# Probing the active fraction of soil microbiomes using BONCAT-FACS

**DOI:** 10.1038/s41467-019-10542-0

**Published:** 2019-06-24

**Authors:** Estelle Couradeau, Joelle Sasse, Danielle Goudeau, Nandita Nath, Terry C. Hazen, Ben P. Bowen, Romy Chakraborty, Rex R. Malmstrom, Trent R. Northen

**Affiliations:** 1 Environmental Genomics and Systems Biology, Lawrence Berkeley National Laboratory, Berkeley, CA USA; 2 Joint Genome Institute, Department of Energy, Walnut Creek, CA USA; 3 University of Tennessee, Oak Ridge National Laboratory, Oak Ridge, TN USA; 4 Earth Science and Environmental Sciences, Lawrence Berkeley National Laboratory, Berkeley, CA USA

**Keywords:** Soil microbiology, Microbial ecology

## Abstract

The ability to link soil microbial diversity to soil processes requires technologies that differentiate active microbes from extracellular DNA and dormant cells. Here, we use BONCAT (bioorthogonal non-canonical amino acid tagging) to measure translationally active cells in soils. We compare the active population of two soil depths from Oak Ridge (Tennessee, USA) and find that a maximum of 25–70% of the extractable cells are active. Analysis of 16S rRNA sequences from BONCAT-positive cells recovered by fluorescence-activated cell sorting (FACS) reveals that the phylogenetic composition of the active fraction is distinct from the total population of extractable cells. Some members of the community are found to be active at both depths independently of their abundance rank, suggesting that the incubation conditions favor the activity of similar organisms. We conclude that BONCAT-FACS is effective for interrogating the active fraction of soil microbiomes in situ and provides a new approach for uncovering the links between soil processes and specific microbial groups.

## Abstract (web summary)

Standard DNA-based analyses of microbial communities cannot distinguish between active microbes and dead or dormant cells. Here, Couradeau et al. use BONCAT (bioorthogonal non-canonical amino acid tagging), flow cytometry, and 16S rRNA gene amplicon sequencing to identify active microbial cells in soils.

## Introduction

Soil communities are composed of thousands of species and reach densities of millions to billions of cells within each gram of material^1,2^. Together, they perform key nutrient cycling functions and, as a collective, are dominant contributors to Earth’s biogeochemical cycles^3^. Next generation sequencing enables a detailed examination of the microbial taxa inhabiting soils^4^, and allows for comparisons across a large sets of samples with the aim of pinpointing the drivers of the microbial diversity^3,5,6^. Such comparative studies reveal patterns of diversity that emerge in soils, especially in terms of correlation with edaphic factors, such as pH^7^, soil texture^8^ or moisture content^9^, or biological factors, such as species-species interaction, life strategy^10^ or rank abundance^6^. Recent reports have suggested that a large fraction, possibly up to ~40%, of the microbial diversity retrieved from soils by molecular methods might come from dead cells or extracellular DNA^11^, and that up to >95% of cells may be dormant at a given point in time depending on the studies^12–14^. Thus, it is challenging to extrapolate links between soil processes and community composition using traditional screening methods^15^. Complementary technologies are needed to distinguish between active cells driving soil processes and the inactive cells that do not^15^.

Active microorganisms have been identified previously using stable isotope probing (SIP) or bromodeoxyuridine (BrdU) labeling. SIP encompasses a series of methods that involve the incorporation of heavy isotopes into newly synthetized DNA and its separation on a density gradient^16^. SIP using labeled ^13^C compounds has shed light onto how the soil microbiome metabolizes certain molecules of interest such as cellulose^17^. Although SIP has been successfully implemented in soils, it remains technically challenging, labor and cost intensive^18^, and can be confounded by cross-feeding and label dilution effects^19^. BrdU is a thymidine analog that gets incorporated into DNA by cells undergoing replication, enabling DNA immunocapturing using BrdU antibodies^20^. This method has been successfully used in soils to probe active microbes^21,22^, however, this technique also suffers technical difficulties, such as a low labeling efficiency which typically require a the large amount of biological material to obtain sufficient amount of labeled DNA for sequencing^15^. Some newer approaches have coupled SIP to single cell analysis using Raman microspectroscopy or NanoSIMS to track metabolically active or newly formed cells^23^ by labeling them with H_2_^18^O^24^, but these methods currently have relatively low throughput and provide limited phylogenetic resolution.

Recently, bioorthogonal non-canonical amino acid tagging (BONCAT) was used to characterize active microbial aggregates from marine sediments^25,26^. This approach uses a relatively fast procedure and small amounts of material, attributes that make it appealing for probing soils. Communities are incubated with homopropargylglycine (HPG), a water soluble analog of methionine containing an alkyne group, which is incorporated into newly synthesized proteins^27,28^. Fluorescent dyes are then conjugated to HPG-containing proteins using an azide-alkyne “click chemistry” reaction^27^. As a consequence, cells that were translationally active during the incubation are fluorescently labeled and can be specifically recovered using fluorescence-activated cell sorting (FACS)^26^. Compared to other methods of activity labeling, BONCAT is faster and less laborious, with the “click chemistry” reaction taking less than 2 h. Perhaps more importantly, BONCAT labels newly made proteins and therefore does not rely on cell division and DNA synthesis to occur, facilitating short term incubations (minutes to hours) and interrogation of slowly dividing cells.

Here, we report the successful use of BONCAT to probe active members of the soil microbiome, as well as the integration of BONCAT with FACS cell sorting and sequencing of the active soil cells. For these studies we incubated soils from the Oak Ridge Field Research Site (ORFRS) site with HPG, and sorted labeled cells using FACS. The composition of the active community was determined through 16S rRNA gene amplicon sequencing. The results were compared with the composition of the total soil community as well as to the ~700 isolates collected from the same field site. These analyses reveal that a large fraction of the active extractable microbes had close relatives among the local isolates collection and among major soil taxa identified in a recent global soil survey^29^.

## Results and discussion

### HPG is actively incorporated by cells in situ

**Fig. 1:**
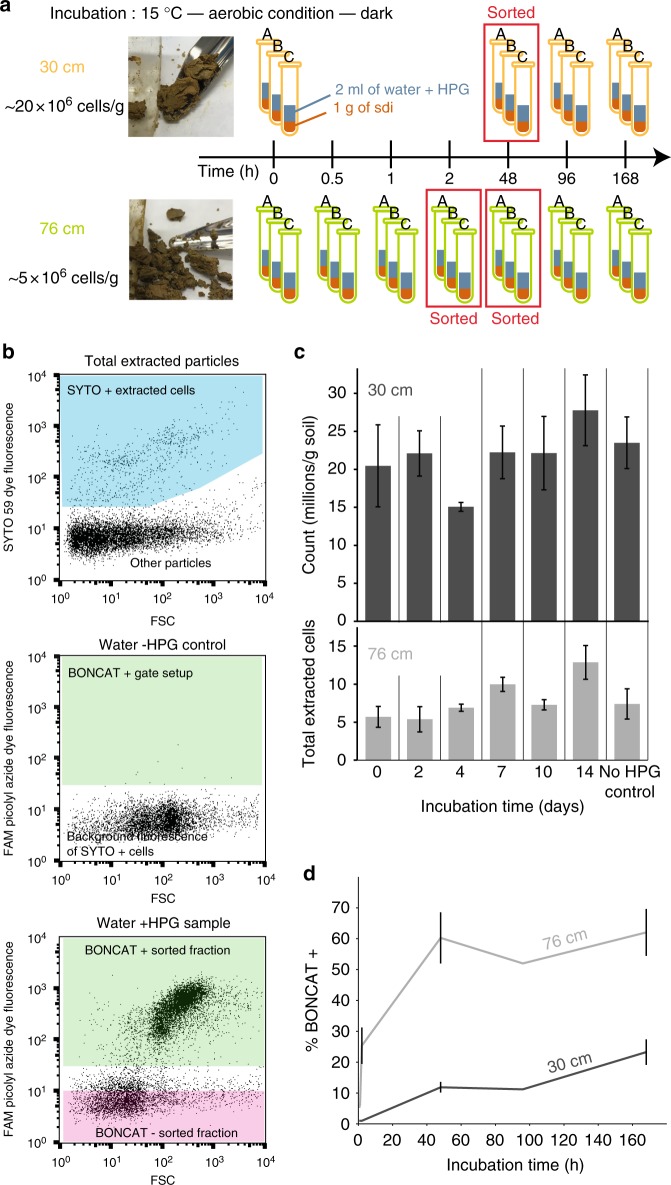
Performing BONCAT-FACS on soil samples. **a** Details of the incubation conditions of the 76 cm and 30 cm soil samples. Samples were incubated in triplicate and three time points were sorted. **b** The gate drawing was done in two steps, first the cells were separated from the background particles based on their DNA dye staining SYTO59 fluorescence (Ex: 640 nm/Em: [655–685 nm]), as pictured by the blue gate on the top panel. The SYTO+ cells were further analyzed for their BONCAT fluorescence with the FAM Picolyl dye (Ex: 488 nm/Em: 530 nm). The middle panel shows an example of a control sample that was water incubated and clicked (water –HPG control), the BONCAT gate (in green) was set such that less than 0.5 % of events would fall in that gate (false positive). The bottom panel is an example of how the BONCAT+ and BONCAT – gates where set in a HPG incubated sample. Note that the green gate is the same than in the control sample. **c** Total extracted cell counts over time showing ~20 million cells per gram at 30 cm and ~5 million cells per gram at 76 cm. **d** Temporal dynamics of BONCAT+ (express as a percent of the extractable cells) labeling for the 30 cm and the 76 cm sample. Error bars represent standard deviation (*n* = 3)

We evaluated the utility of BONCAT for identifying translationally active cells from soil systems consisting of a highly heterogeneous matrix, and we coupled BONCAT with FACS to detect and recover individual active cells, as opposed to microbial aggregates consisting of hundreds of cells^26^. Soil samples were collected at the ORFRS in Oak Ridge, TN, USA and were horizontally cored at 30 cm and 76 cm below surface for the analyses of two distinct communities (Fig. 1a). The 30 cm soil had more quartz and less mica than the 76 cm sample that was composed of more clay (Supplementary Fig. 1). None of these samples had detectable amount of methionine based on LC-MS and therefore it is unlikely that there was significant competition from methionine for incorporation of HPG (Supplementary Fig. 2).

To confirm that HPG was actively incorporated by the cells in situ, we performed a killed control experiment on the 76 cm soil with duplicate samples for each treatment condition. Cells were either fixed before or after incubation with HPG by the addition of paraformaldehyde (3% final concentration). Cells fixed prior to HPG incubation did not acquire fluorescence following the click reactions. Similarly, unfixed cells incubated without HPG also did not acquire fluorescence (See Fig. 1b for an example of how sort gates were determined). In contrast, both unfixed cells and cells fixed after HPG incubation acquired a distinct green fluorescence signal corresponding to the azide dye addition to the BONCAT labeled cells. The fraction of fluorescent cells and the per-cell fluorescence intensities were comparable between unfixed and post-incubation fixed cells, although the general shape of the analyzed events clouds differed in the cytograms (Supplementary Fig. 3, Supplementary table 2). This confirmed that HPG was only incorporated by active cells and that fixation was not required for the cycloaddition of the BONCAT azide fluorescent dye, as previously reported^26,28^. Therefore, all additional experiments were performed with unfixed cells to avoid the negative impacts of paraformaldehyde fixation on subsequent PCR amplification of 16S rRNA genes^30^.

### Comparison of the extractable cells to the total soil community

Although the soil was directly transferred from the soil core and not agitated during the incubation, cells needed to be detached from the soil matrix following incubation with HPG and captured on a 0.2 µm filter (see methods, Supplementary Table 3) for subsequent click reaction and FACS. We evaluated the impact on community composition introduced by this disaggregation step as it would filter out the extracellular DNA^11^ and cells passing through a 0.2 µm filter. The disaggregation step could also impact community composition if some groups detach preferentially from the soil aggregates. Thus, we compared the 16S rRNA composition of DNA extracted of bulk soil with the composition of detached cells captured on the 0.2 µm filter.

**Fig. 2:**
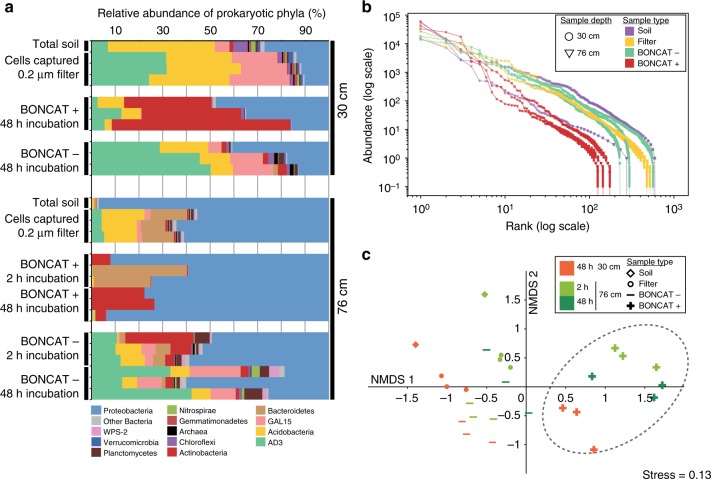
Composition of total community and sorted fractions (16S rRNA gene sequencing). **a** Microbial diversity displayed at the phylum level for all samples analyzed. **b** Rank vs. abundance (absolute abundance from the rarefied OTU table at an even depth of 81000 sequences, see “methods”) plot in log–log scale of the libraries of averaged biological replicates, standard deviations are displayed as error bars (*n* = 3). **c** NMDS ordination of the Bray Curtis pairwise distance of all libraries. 95% confidence ellipse is displayed on the BONCAT+ group of samples. “Bulk Soil” samples are libraries constructed from total DNA extracted from soil, “Filter” samples are DNA extracted from all cells detached from soil and captured on a 0.2 µm filter, BONCAT+ and BONCAT− libraries were constructed from corresponding cell sorted samples

The microbial community structure retrieved from the total soil DNA purification at 30 cm and at 76 cm differed at the phylum level (Fig. 2a and Supplementary Fig. 5). For example, the 30 cm soil was dominated by Acidobacteria as well as candidate phlya AD3 and GAL15. The 76 cm soil was largely dominated by Proteobacteria with a higher fraction of Bacteroidetes than found in the 30 cm soil. At the feature level (also called exact sequence variant (ESV), i.e., sequences denoised and clustered at 100% similarity), at both depths the most abundant feature was an Alphaproteobacterium genus *Aquamicrobium* that accounted for 8.77% and 72.9% of the analyzed sequences from 30 cm full soil and 76 cm full soil, respectively. This feature was only partially captured on the 0.2 µm filters (it represented 2.4% and 3.1% respectively for the 76 cm and the 30 cm filters), which might be explained by (i) a technical bias in determining relative abundance^31^ or (ii) non exclusively, the facts that this taxon exists largely as extracellular DNA in the soil, was not detached efficiently from the soil, or is of small size and not retained by the filter as suggested by the description of *Aquamicrobium* strains^32,33^. The number of operational taxonomic units (OTUs; features clustered at 97% similarity) captured on the filters for the 30 cm sample was half the number retrieved from the total soil, whereas the number captured at 76 cm filters captured greater than or equal to the full soil sample in average (Supplementary Table 1). The OTUs that were present on the 76 cm filter sample and not retrieved in the soil were found in low abundance (<0.1%) and might have come from the rare members of the soil microbiome. As expected the community composition of the extractable cell fraction captured on a filter was not identical to the bulk soil libraries. Since BONCAT labeling and cell sorting was performed on the total cells captured on a filter, referred to as the ‘extractable fraction’ throughout the remainder of the manuscript, results from sorted cells are compared to the extractable fraction unless otherwise noted. It is worth noting that the extractable fraction might also be a better proxy for the intact cellular fraction of the soil microbiome as it filters out extracellular DNA^11^ that can end up in sequencing results of DNA extracted from bulk soil.

### Tracking of the active cell fraction through time using BONCAT

To identify individual active cells within soils, samples from 30 cm and 76 cm were incubated with HPG for up to one week (168 h) with periodic sampling followed by fluorescent labeling. The total number of cells was ~20 million cells g^−1^ soil at 30 cm and ~5 million cells g^−1^ soil at 76 cm (Fig. 1b). Cell population increased over time (Pearson *R* = 0.45, *p* = 0.062 and Pearson *R* = 0.76, *p* < 0.005 at 30 and 76 cm respectively), indicating there was neither acute toxicity leading to massive cell loss nor a stimulation leading to a massive cell population bloom during the incubations. Although higher cell densities have been reported in other soils^34^, our soil samples were oligotrophic (Supplementary Fig. 1), with very low nitrogen (<0.05%), no detectable amount of phosphorus, and a TOC (<0.15%) of the same order of magnitude that bare arid land soils^35^. Moreover, we could only isolate 0.5–33 ng of DNA per g of soil while other soils often yield micrograms of DNA per gram of soil^34^. Altogether, these observations indicate that these soils might only be able to support a small population of cells, and we are therefore confident the extractable cell fraction in this study (10^6^–10^7^ cells per gram of soil, Fig. 1c) represents a large fraction of the soil microbiome.

The fraction of BONCAT+ (Fig. 1b, d) cells increased over time in both soil samples (Fig. S4), with a distinct rate of labeling and fraction of labeled cells detected for both soil samples. For example, cells from the 76 cm soil were labeled quickly (clear BONCAT+ population were visible as early as 30 min incubation) and ~60% of all extractable cells were labeled by 48 h, whereas cells from the 30 cm soil were labeled more slowly (no BONCAT labeling after 1 h) and only ~20% total cells were labeled after 48 h (Fig. 1d and Supplementary Fig. 4). These differences, which were consistent among biological replicates, suggest that the microbial community found at 76 cm was composed primarily of active cells while the community at 30 cm had a larger fraction of inactive cells.

Previous studies found that only a small fraction of cells (as low as 0.1–2%) were active at any giving time^12^, shaping the view that most soil microbes are dormant^13,14^. In this study, roughly 20% or more of the extractable cells we analyzed were active at both depths, and the 76 cm soil reached this value in only 30 min of incubation. The high number of active cells we found could be linked to the fact that we labeled translationally active cells while other techniques usually probe actively dividing cells therefore biasing against slow growers. The incubation conditions, which mimicked a natural event such as a heavy rain that saturates the soil, may have also impacted the fraction of active cells. Similar experiments involving other activity probing methods, with different incubation conditions and soil types will reveal the magnitude of variation that the active fraction undergoes under natural climatic conditions.

### The active fraction is a selected subset of the total community

To determine the identity of extractable active cells, we sequenced the 16S rRNA genes of BONCAT+ cells from 30 and 76 cm soil samples. Specifically, triplicate collections of 35k–75k BONCAT+ cells recovered by FACS (2 h incubation of the 76 cm sample and 48 h incubations of the 76 and 30 cm samples) and characterized using iTag sequencing (Supplementary Table 1). Both soils were sequenced at the 48 h time point as it represents the beginning of the plateau phase of the BONCAT labeling for both cores (Fig. 1d). For the 76 cm soil, the 2 h time point was also sequenced to identify cells that were quickly labeled. Unlabeled cells (BONCAT-) were also sorted and sequenced from these time points (Fig. 1a). In order to compare the BONCAT sorted fractions to the total community at a large scale, we plotted the rank *vs*. abundance of all libraries (Fig. 2b). This plot clearly shows that the BONCAT+ populations were distinct from the rest of the samples, with a steeper slope reflecting a faster drop of diversity at higher ranks. The pattern for BONCAT− samples was similar to the extractable cells (filter samples). In order to assess if this difference was from compositional variation, we computed and ordinated a beta-diversity metric (Bray-Curtis measure of dissimilarity), and ordinated pairwise measures between samples (Fig. 2c). The resulting NMDS plot revealed that all the BONCAT+ fractions from both the 30 and the 76 cm formed a distinct group from the rest of the samples (Adonis, *F* = 2.65, *p* value = 0.001). This analysis supports the observation that the BONCAT− cells fraction resemble the total extractable cells, while the total DNA samples clustered further away. These results also indicate that the pools of BONCAT− cells, although of lower diversity compared to the control total soil and extractable cells, were a random subset of soil community, while the BONCAT+ fraction was clearly composed of a distinct and reproducible subset of the community.

**Fig. 3:**
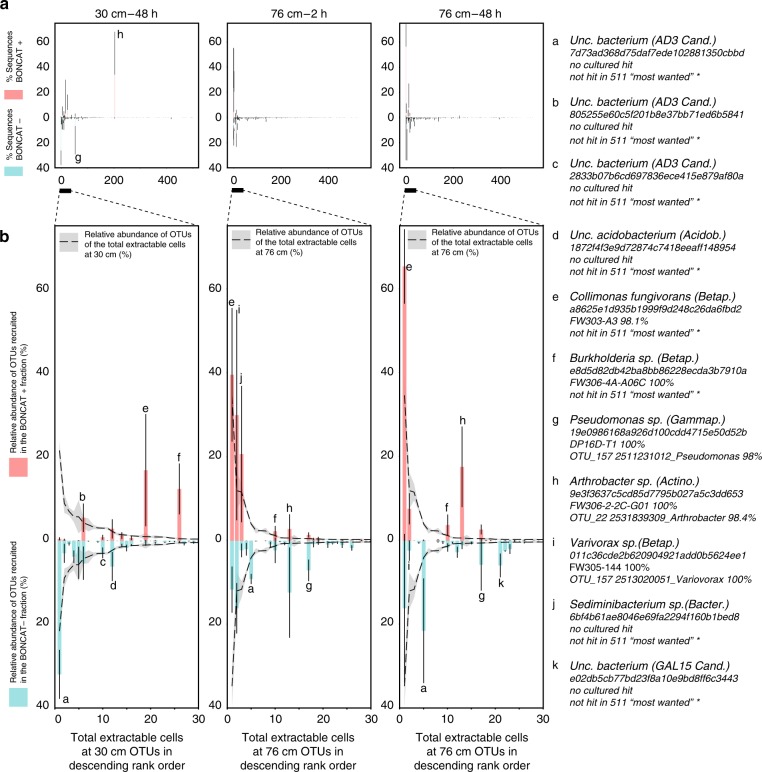
Comparing the composition of the BONCAT+ and BONCAT− populations. **a** Relative abundance (in percent, ±SD, *n* = 3) of OTUs present in the BONCAT+ (red) and BONCAT− (blue) for the 30 cm–48 h incubation (left panel), 76 cm–2 h incubation (middle panel) and 76 cm–48 h incubation (right panel). The OTUs have been ranked in descending order from left to right according to their relative abundance on the filter samples (all cells detached and captured on a filter). **b** Close-up on the 30 most abundant OTUs overlaid with their abundance on the filter samples (dashed line, ±SD shows as gray shading *n* = 3). The most abundant OTUs are indexed from a to k. Their taxonomy, ID, hit in the ENIGMA culture collection and matches to the 511 most abundant soil microbiome^29^ is provided on the right legend panel. Error bars represent standard deviation (*n* = 3)

Analyzing the phylogeny of the BONCAT+ samples at the phylum level, we found that at 30 cm, the extractable active fraction was dominated by Actinobacteria (Fig. 2a and Supplementary Fig. 5), with one *Arthrobacter* OTU encompassing ~51% of the retrieved sequences on average (“h” Fig. 3a), while the 76 cm active population was dominated by Proteobacteria. At the OTU level (features clustered at 97% similarity) (Fig. 3), the BONCAT positives OTU h-e-f were highly active at both 30 cm and 76 cm independently of their abundance in the parent population. For instance, OTU h *Arthrobacter* was only recovered at low abundance (rank 214) from the extractable cells, while it is the most abundant OTU in the BONCAT+ fraction for this sample. In contrast, the most abundant members of the 76 cm community (e.g., OTU a Fig. 3) were BONCAT−, indicating that they did not respond to the incubation condition. These observations suggest that an OTU’s activity could not be predicted from their abundance in the parent community alone.

Although we are confident that the BONCAT+ fraction is composed of translationally active cells, the relative proportion of the OTUs within each library should be interpreted with some caution, as potential biases from PCR when producing the iTags libraries^31,36^ and sorting (in the detachment from the filter and DNA staining steps) may impact estimates of relative abundance. More precisely, a few OTUs account for the majority of the sequences retrieved in the BONCAT fractions while their abundance was lower in the total extractable community. Given that the size of the total extractable population only increased slightly during the first 48 h of incubation, this can be explained by two non-exclusive hypotheses: (i) some technical bias in determining relative abundance or (ii) real growth of certain OTUs balanced by loss of other members. While our experimental design does not allow us to distinguish between microbes that were already active and the ones that became active during the incubation, it seems reasonable to assume that the signal we measured is perhaps a mix of both types. Attempting a comparison of the 76 cm sample extractable BONCAT+ cells after 2 and 48 h of incubation we observe that the OTU j dropped while the OTU e and h increased in relative abundance (Fig. 3). We conclude that BONCAT is a promising method to interrogate through time community dynamic at the OTU level.

Another interesting finding from this study is that the BONCAT+ signal plateaued at around ~4 million active cells per gram of soil independently of the size of the total population. The BONCAT+ plateau may be due to the exhaustion of HPG by the cells or its sorption to the soil particles, or there may have been some resource limit within these samples that controlled the total number of active cells in each soil sample. It is also possible that some active cells were not labeled due to their inability to incorporate HPG. At this point, it is not possible to determine which scenario explains the observed plateau in our study, but the fact that BONCAT+ cells belonged to 251 different OTUs spanning 17 bacterial phyla and accounted for up to ~70% of the extractable cells, suggests, as previously noted^25,26,37^, that HPG is in fact incorporated by a large set of microbial species.

### Comparison of BONCAT+ cells with soil isolates and phylotypes

We examined how the composition of a culture collection generated from the same experimental site, (see “Methods” and Supplementary Data 1) compared to the diversity of the active, and presumably ecologically relevant, fraction of the community. More specifically, we compared 16S rRNA gene sequences of BONCAT+ cells and total cells libraries (both total soil and extractable cells) to 16S rRNA gene sequences from 687 isolates collected from this same location (Supplementary Data 1). Surprisingly, between 77 and 98% of total sequences from BONCAT+ cells shared >97% sequence similarity with the isolates. While the relative abundances should be interpreted with some caution due to potential impact of amplification or sorting bias^31,38^, these results suggest that a large fraction of the active extractable community had close cultured representatives. These findings also indicate the culture collection will be an important resource for exploring connections between soil communities and ecological processes at the Oak Ridge study site.

**Fig. 4:**
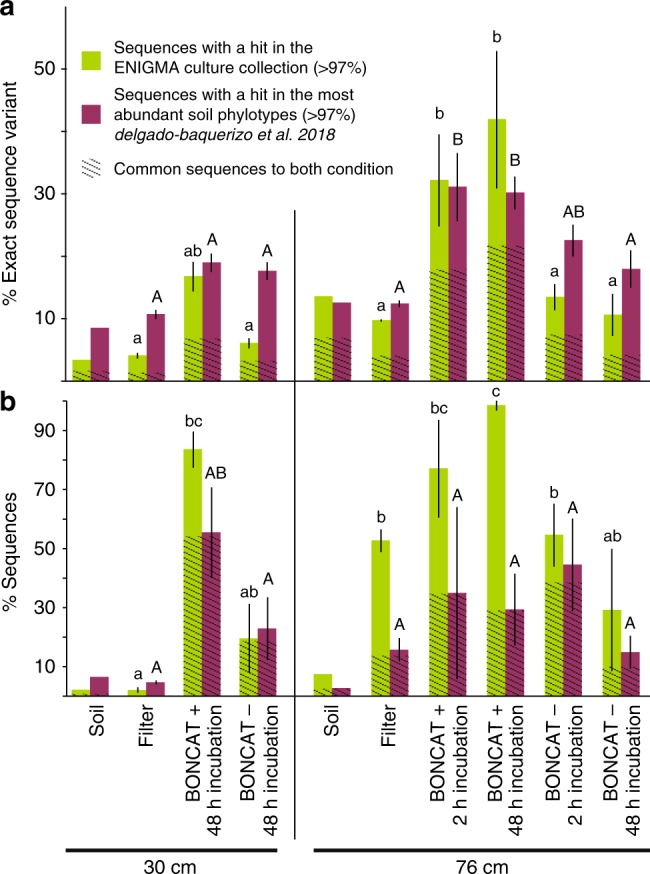
Prevalence of soil isolates and ubiquitous soil OTUs among BONCAT+ cells. **a** Percent features (non-clustered DADA2 results) and **b** percent sequences from the current libraries with a hit (>97% sequence similarity) in the ENIGMA culture collection (this collection contains 697 full-length 16S rRNA gene from strains that were isolated from the same field site as the samples considered in this study) (green), in the set of 511 phylotypes identified as the most abundant by^29^ (yellow) or both (dashed area). Data are average (*n* = 3) ± SD, letters indicate ANOVA post hoc significant differences. “soil” samples are libraries constructed from total DNA extracted from soil, “filter” samples are DNA extracted from all cells detached from soil and captured on a 0.2 µm filter, BONCAT+ and BONCAT- libraries were constructed from corresponding cell sorted samples

The observation that a substantial portion of the active extractable cells have cultured relatives aligns well with the recently published contribution from Delgado-Baquerizo et al.^29^ that identified a list of 511 phylotypes (OTUs with 97% cutoff) encompassing 44% of the microbial diversity of soils worldwide. Among these phylotypes, 45% had a cultured representative, suggesting that cultivation efforts have already yielded to isolate representatives of soil ubiquitous taxa. In order to further compare our dataset with these 511 ubiquitous soil phylotypes, we ran BLAST on a set of representative sequences of our libraries OTUs and recovered the >97% hits (Fig. 4a, b). We found that there was an overlap between the sequences found in our culture collection and from the 511 reference phylotypes^29^. Three of the most abundant BONCAT+ OTUs retrieved belonged to the 511 prominent members of the global atlas for soil microbiome^29^ (e.g., OTU g, h, i Fig. 3, 100% sequence similarity), further supporting the idea that cultured isolates might be particularly relevant to the understanding of the soil microbiome in this study.

## Conclusions

We find BONCAT to be a useful tool for the analysis of the active fraction of soil microbiomes when coupled to fluorescence activated single cell sorting and sequencing. This enables separating active cells from extracellular DNA, dormant microbes, and dead cells. BONCAT can be viewed as a filter that focuses environmental DNA analyses on the active and likely ecologically relevant extractable cell fraction. As with any filter, the BONCAT procedure may also introduce some biases, and it will need to be benchmarked against other activity probing strategies and tested in a larger variety of soil types. Nonetheless, we showed that BONCAT-FACS can be used to track the active cell population dynamics and dissect the behavior of active members at the phylum or OTU level. Our experiments resulted in consistent enrichment of a specific set of organisms in the BONCAT+ fraction, validating the reproducibility of BONCAT-FACS approach. Surprisingly, we found that a large fraction of the extractable cells was active under our incubation conditions (20–60%) and that the sequences from the active population had close representatives in the culture collection established from the same sampling site. Although the biological finding of this study are limited to the specific soils and incubation conditions reported here our data demonstrate that BONCAT labeling can be applied to soil in a feasible, robust and reproducible manner, and could be widely used in future soil microbiome research.

## Methods

### Description of the umbrella project of this study

This study and samples are part of the ENIGMA (Ecosystems Networks Integrated with Genes and Molecular Assemblies) project (http://enigma.lbl.gov/), a multi-PIs DOE SFA (Department of Energy Science Focus Area). The ENIGMA field site has already been studied^39^ and a culture collection of isolates from this exact field site was made available to this study, it is referred as the “the culture collection” in the main text (Supplementary File).

### Samples collection and incubation condition

Two 4 cm diameter sample soil cores were collected horizontally from Oak Ridge, TN (GPS 35.941133, −84.336504) on 24 January 2017 from a silt loam area. A vertical trench was made and a first core was taken at 30 cm depth while the second one was collected at 76 cm depth. Both cores were shipped cooled and where stored in the dark at 4 °C until processing. At the time of the experiment (within 1–3 months after collection) a piece of ~1 g of soil was sampled from the distal part of the core under sterile conditions for each replicate and placed into a 14 ml polystyrene dual positions snap cap that was kept in the upper position allowing gas exchange through the incubation. Each replicate was incubated with 2 ml of 50 µM L-homopropargylglycine (HPG, Click Chemistry Tools, Scottsdale, AZ, USA) in sterile water (DEPC diethyl pyrocarbonate treated filter sterilized water, pH 7) at 15 °C in the dark, no mixing procedure was applied (i.e., we did not make a soil slurry) and the headspace was 12 ml. This temperature was chosen because it is the average surface temperature at the field site. The incubation was done aerobically because data from the field indicate the soil is aerobic above 1 m in this area (T. Hazen, personal communication). Two milliliter was enough to fully submerge the 1 g of soil used for each replicate, this level of hydration ensured that all the soil pores were completely flooded and that there was no diffusion limitation of HPG. The addition of 2 ml diluted the soil solutes and might represent a field event corresponding to a heavy rain capable of flooding the soil. Control samples were incubated under the same conditions with water but without HPG (water –HPG control). The full incubation design can be found in Fig. 1a. At the end of the incubation period (spanning 0.5–168 h) 5 ml of 0.02% Tween® 20 (Sigma-Aldrich, ST Louis, MO, USA) in phosphate saline buffer (1X PBS) was added to each tube (already containing 2 ml of HPG solution and 1 g of soil) and further vortexed at maximum speed for 5 min (Vortex-Genie 2, Scientific Industries, Inc., Bohemia, NY, USA) in order to detach cells from the soil particles. Culture tubes were then centrifuged at 500 × *g* for 55 min (centrifuge 5810R, Eppendorf, Hamburg, Germany) and the supernatant containing the detached cells was aliquoted in 700 µl aliquots and frozen right away at −20 °C in 10% glycerol (Sigma-Aldrich, ST Louis, MO, USA) dissolved in PBS until further processing. The amount of supernatant collected per aliquot was chosen based on preliminary data that indicated that this amount was optimal to sort the target number of cells downstream.

### Determining background fluorescent labeling in BONCAT

We performed a killed control experiments to validate the active incorporation of HPG and fluorescent labeling by cells by fixing duplicate soil samples from the 76 cm with 3% paraformaldehyde (PFA, Sigma-Aldrich, ST Louis MO, USA) for 1 h at RT. The aim of this experiment was to confirm that the cells needed to actively incorporate HPG to be labelled and that the simple diffusion of HPG into cells would not create artefactual signal^25,26^. Tests involving PFA were used as controls during methods development, and all sequencing results and were generated from unfixed sample.

We performed the PFA fixation (PFA, Sigma-Aldrich, ST Louis MO, USA) either prior incubation with HPG or right after. A set of samples was first fixed with 3% paraformaldehyde for 1 h at RT, while for another set PFA was spiked post incubation. The details of the incubation conditions can be found in Supplementary Table 2. These killed controls were compared to other live controls that were incubated without HPG in order to measure non-specific fluorescent labeling of cells. The killed controls and the no HPG controls went through the click chemistry reaction (see below) and their fluorescence in the BONCAT dye channel measured to determine the background fluorescence of the samples. Incubation times were 2 h and 48 h. This set of sample was handled as previously described, cells were detached from the soil and frozen stock in 10% glycerol were kept at −20 °C until further evaluation of HPG incorporation, see below.

### Soil properties, mineral and organic composition of the soils

Bulk X-ray powder diffraction was used to analyze the mineralogical composition of the soils cores. Powdered samples were loaded on an autosampler in a Rigaku SmartLab X-ray diffractometer (Rigaku, The Woodlands, TX, USA), using a Bragg-Brentano geometry in a theta-theta configuration. Data were collected from 4° to 70° of 2θ, using Cu Kα radiation. After manual identification of the phases present, a Rietveld refinement was performed to obtain their weight fractions, using the software MAUD^40^.

The soil chemistry analyses were performed by the UC Davis Analytical lab (https://anlab.ucdavis.edu/). Total carbon and total nitrogen were measure by the combustion method as described by the AOAC Official Method 272.43. The TOC was measured the same way after removal of carbonate via acid fumigation^41^. Soil nitrate and extractable ammonium where determined by the flow injection analyzer method^42,43^. The extractable phosphate (under detection limit of 1ppm for our samples) was measured by the Olsen-P method^44^, this method measures the bioavailable inorganic phosphate (orthophosphate).

### Click reaction - BONCAT stain

A volume of 700 µl of frozen cells of each sample were allowed to thaw at 4 °C for ~1 h. In the meantime, the click-reaction mixture was prepared by mixing the dye premix with the reaction buffer. This premix consisted of 5 µl copper sulfate (CuSO_4_ 100 µM final concentration), of 10 µl tris-hydroxypropyltriazolylmethylamine (THPTA, 500 µM final concentration), and of 3.3 µl (FAM picolyl azide dye, 5 µM final concentration). The mix was incubated 3 min in the dark before being mixed with the reaction buffer, which was made of 50 µl sodium ascorbate freshly prepared in 1X PBS at 5 mM final concentration and 50 µl of aminoguanidine HCl freshly prepared in 1X PBS at 5 mM final concentration and 880 µl of 1X PBS. All reagents were purchased from Click Chemistry Tools (Click Chemistry Tools, Scottsdale, AZ, USA). Once thawed, the cells were captured on a 0.2 µm GTTP isopore™ 25 mm diameter filter (MilliporeSigma, Burlington, MA, USA) and rinsed with 7 ml 1X PBS. The filter was then placed on a glass slide and 80 µl of the click reaction mixture was quickly added before covering the filter with a coverslip to avoid excess oxygen during the click reaction. The slides were incubated in the dark for 30 min and each filter was then thoroughly washed three times in a succession of three baths of 20 ml 1X PBS for 5 min each. The filters were finally transferred to 5 ml tubes (BD-Falcon 5 ml round bottom tube with snap cap, Corning^TM^, Corning, NY, USA) with 2 ml of 0.02% Tween® 20 in PBS, with the cells facing inwards and vortexed at maximum speed for 5 min to detach the cells. The tubes were incubated for 20 min at 25 °C, and subsequently stored at 4 °C. Before being loaded onto the cell sorter (BD-Influx^TM^, BD Biosciences, San Jose, CA, USA), the samples were filtered through a 35 µm filter (BD-falcon 5 ml tube with cell strainer cap, Corning^TM^, Corning, NY, USA). Each set of experiment included water incubated samples (water –HPG control) that were clicked along with each set of samples, the fluorescence of the water incubated samples in the BONCAT dye channel was used to define the BONCAT staining background of each single click reaction.

### Flow cytometer, cell count, and cell sorting

For the cell counts, the cells were prepared the exact same way as described above, but the click reaction was omitted and the cells detached from the soil were stained 1X SYBR^TM^ (ThermoFisher Scientific, Invitrogen, Eugene OR, USA). For the evaluation of the BONCAT stained samples, cells were counterstained with the SYTO^TM^ 59 (ThermoFisher Scientific, Invitrogen, Eugene OR, USA) DNA dye for 5 min at RT at 0.5 µM. The cell sorter (BD-Influx^TM^, BD Biosciences, San Jose, CA, USA) was setup to capture the FAM picolyl azide dye (excitation = 490 nm/emission = 510 nm) in the green channel off a 488 nm blue laser and the counter DNA stain (excitation = 622 nm, emission = 645 nm) in the red channel off of a 630 nm red laser. A first gate was drawn on the SYTO positive (SYTO+) particles, under the assumption that this would capture the cells. SYTO+ events accounted for 0.1–5% of the events depending on the samples, most of the events being abiotic, most probably clays or other minerals (Supplementary Fig. 1). The BONCAT positive (BONCAT+) and BONCAT negative (BONCAT−) where further gated as a sub-fraction of the SYTO+ cells based on the BONCAT dye fluorescence. The no HPG control sample that went through click reaction steps along with the labeled samples was used to define the level of background BONCAT stain fluorescence, the BONCAT− gate was drawn under that line and BONCAT+ gate to ensure less than 0.5% false positives (Fig. 1b). The percent of BONCAT+ determined for a time course for both the 30 cm and the 76 cm sample guided the sorting decisions. We decided to sort three biological replicates at two incubation time points for the 76 cm sample (2 h and 48 h) and three biological replicates at one time point for the 30 cm sample (48 h). A total of 35–75 k cells (the target number was 75 k but some samples had too low cell counts or too low labelled cell counts, see Supplementary table 1 for detailed counts) were sorted in parallel for the BONCAT+ and BONCAT− gates into a 96 well plate. Plates were frozen at −80 °C until processing.

### Total DNA extraction from soil and filters

In order to compare sorted cells to the soil microbiome, total purified DNA was prepared from the soil cores and the extractable cells captured on a 0.2 GTTP isopore™ 25 mm filter (MilliporeSigma, Burlington, MA, USA). We used the Qiagen-MoBio Power soil DNA kit (Qiagen, Hilden, Germany) following the manufacturer instructions, except for the lysis step that was performed by shaking the tubes at 30 Hz for 10 min in a tissue homogenizer (TissueLyser II, Qiagen, Hilden, Germany).

### Libraries preparation and sequencing

In order to pellet the sorted cells, the 96 well plates were centrifuged at 7200 × *g* for 60 min at 10 °C. The plates were further centrifuged upside-down for 20 s at 60 × *g* to remove supernatant. The pelleted cells were lysed using PrepGEM (zyGEM, Charlottesville, VA, USA) chemical lysis in 2 µl reactions following manufacturer’s recommendation. 0.2 µl of 10X Green buffer, 0.02 µl of PrepGEM, 0.02 µl of lysozyme and 1.8 µl of water were added to each well. Note that six empty wells were submitted to PrepGEM lysis and library construction to account for potential contaminant. The plates were then placed in a thermocycler for 30 min at 37 °C and 30 min at 75 °C. The iTag PCR was performed directly on the cell lysate following the JGI standard operating protocol (https://jgi.doe.gov/user-program-info/pmo-overview/protocols-sample-preparation-information/). Briefly, the V4 region of the 16S rRNA gene was amplified using the universal primer set 515F (GTGYCAGCMGCCGCGGTAA), 806R (GGACTACNVGGGTWTCTAAT)^45^. The adapter sequences, linkers and barcode were on the reverse primer. The 16S rRNA gene PCR was performed in a final volume of 25 µl (10 µl of the 5 Prime master mix, 0.5 µl of the forward primer (at 10 µM), 1.5 µl of the reverse primer (at 3.3 µM), 0.44 µl of BSA, 10.5 µl of water and 2 µl of cell lysate). The PCR condition was as follows: after an initial denaturation step at 94 °C for 3 min, 30 PCR cycles occurred consisting on a 45 s denaturation step at 94 °C followed by a 1 min annealing step at 50 °C and a 1.5 min elongation step at 72 °C. A final elongation step of 10 min at 72 °C was further added to finish all incomplete target sequences. The V4 region of the 16S rRNA gene from the total DNA extracted from the soil and from the cells enriched on filters were also amplified using the same PCR condition. The PCR products were cleaned using the Agencourt AMpure XP beads solution (Beckman Coulter Life Sciences, Indianapolis, IN, USA) to remove excess primers and primer dimers. PCR products were incubated with 80% (v/v) beads for 5 min at 25 °C before being placed on a magnetic holder (MagWell™ Magnetic Separator 96, EdgeBio, San Jose, CA, USA). The supernatant was removed and the beads were washed with 70% v/v ethanol three times before being resuspended in 11 µl of water. The total DNA extracts were processed in parallel, the only difference being that the iTag PCR was performed in 50 µl final volume and the PCR product was resuspended in 16 µl water after the bead clean-up step. PCR products were run on a High Sensitivity DNA assay Bioanalyzer chip (2100 Bioanalyser, Agilent, Santa Clara, CA, USA) to confirm fragment size and concentration. PCR products were pooled to an equimolar concentration and run on the Illumina MiSeq platform (Illumina, San Diego, CA, USA). Sequences data have been archived under the Bioproject ID PRJNA475109 at the NCBI.

### Sequences processing

The sequences were processed using Qiime2 v2017.9^46^. The sequences were imported in qiime2 using the *fastq manifest* format. Sequences were further denoised, the primer trimmed (20 nucleotides from each side) and paired using DADA2^47^ as implemented in the Qiime *dada2 denoise-paired* plug-in. This step also included a chimera check using the *consensus* method. The output was a table of 4063 features (also called exact sequence variant (ESV)) of 6,419,059 sequences. 130 features had at least one hit in one of the six no template controls and were not considered for further analysis. The filtered table had 6,110,776 sequences gathered into 3933 features with a median value of 205,167 sequences per sample. The features were further clustered into operational taxonomic units (OTUs) at a threshold of 97% similarity using the *vsearch cluster-features-de-novo* plug-in. The clustered OTU table had 1533 OTUs in total. The absolute number of OTUs in 16S rRNA genes analyses can vary by up to three orders of magnitude depending on the technique used^48^, DADA2 is known to return a more conservative number than the previously widely used upfront clustering methods by decreasing the number of false positives^47^. This relatively low OTU count is also consistent with the very low level of organics (carbon and nitrogen) in these soils, which total organic carbon (TOC) are comparable to un-colonized arid lands where microbial diversity is known to be reduced^49^. The taxonomy of the representative sequences was assigned using the *feature-classifier classify-sklearn* plug-in (https://data.qiime2.org/2018.2/common/gg-13-8-99-515-806-nb-classifier.qza). This classifier was trained on the Greengenes database 13_8 99% trimmed to the amplified region (V4 515F/806R). If the classifier could not assign the representative sequences at the phylum, then they were manually checked on the most up-to-date Silva SINA alignment service (https://www.arb-silva.de/aligner/) and the Silva classification was retained. The OTU table with assigned taxonomy was used to build the bar graph at the phylum level and all downstream analyses. Bray Curtis pairwise distance beta-diversity metric was computed on the OTU table and the obtained triangular distance matrix was ordinated using NMDS. The OTU table was further rarefied to an even sequence depth of 81,000, the rarefied OTU table was used to construct the rank-abundance plot. OTUs in each library were sorted according to their abundance using the average method where a group of similar values gets the average rank value for the group; the abundance was plotted in log scale against the log rank value in descending order.

### Comparison with reference dataset

We compared our iTag data with the 697 full-length 16S rRNA gene of the ENIGMA Project’s existing culture collection from this field site and with the 511 16S rRNA gene sequences of the most abundant and widespread soil microbiome members, retrieved from Delgado-Baquerizo et al.^29^. We performed a nucleotide BLAST of one representative sequence per feature against the ENIGMA isolate database or the “511 most wanted soil phylotypes”^29^ database using Geneious R9^©^. A cutoff of >97% similarity was used to determine if a sequence from our dataset had a match in the ENIGMA isolate database and/or the “511 most wanted soil phylotypes” database.

### LC-MS soil metabolomics

Triplicates of 2 g of soils from 30 cm and 70 cm were extracted using 8 ml of LCMS grade water and incubated 1 h on an overhead shaker at 4 °C. Aqueous extractable components were collected by removal of insoluble material with centrifugation at 3220 × *g* for 15 min at 4 °C, filtration of supernatants through a 0.45 µm PVDF syringe filter (MilliporeSigma, Burlington, MA, USA), followed by lyophilization of filtrates to remove water (Labconco 7670521, Kansas City, MO, USA). Dried samples were then resuspended in 500 µl of LCMS grade methanol, bath sonicated at 25 °C for 15 min, and then clarified by filtration through 0.2 µm PVDF microcentrifugal filtration devices (1000 × *g*, 2 min, 25 °C). Methanol extracts were spiked with an internal standard mix (^13^C,^15^N universally labeled amino acids, 767964, Sigma-Aldrich, USA, which included canonical amino acids, including methionine, at a final concentration of 10 µM each). Metabolites in extracts were chromatographically separated using hydrophilic liquid interaction chromatography on a SeQuant 5 µm, 150 × 2.1 mm, 200 Å zic-HILIC column (1.50454.0001, Millipore) and detected with a Q Exactive Hybrid Quadrupole-Orbitrap Mass Spectrometer equipped with a HESI-II source probe (ThermoFisher Scientific). Chromatographic separations were done by an Agilent 1290 series HPLC system, used with a column temperature at 40 °C, sample storage was set at 4 °C and injection volume at 6 µl. A gradient of mobile phase A (5 mM ammonium acetate in water) and B (5 mM ammonium acetate, 95% v/v acetonitrile in water) was used for metabolite retention and elution as follows: column equilibration at 0.45 mL4 5 ml min^−1^ in 100% B for 1.5 min, followed by a linear gradient at 0.45 5 ml min^−1^ to 35% A over 13.5 min, a linear gradient to 0.6 mL 5 ml min^−1^ and to 100% A over 3 min, a hold at 0.6 6 5 ml min^−1^ and 100% A for 5 min followed by a linear gradient to 0.45 5 ml min^−1^ and 100% B over 2 min and re-equilibration for an additional 7 min. Each sample was injected twice: once for analysis in positive ion mode and once for analysis in negative ion mode. The mass spectrometer source was set with a sheath gas flow of 55, aux gas flow of 20 and sweep gas flow of 2 (arbitrary units), spray voltage of |±3| kV, and capillary temperature of 400 °C. Ions were detected by the Q Exactive’s data dependent MS2 Top2 method, with the two highest abundance precursory ions (2.0 *m/z* isolation window, 17,500 resolution, 1e5 AGC target, 2.0 *m/z* isolation window, stepped normalized collisions energies of 10, 20 and 30 eV) selected from a full MS pre-scan (70–1050 *m/z*, 70,000 resolution, 3e6 AGC target, 100 ms maximum ion transmission) with dd settings at 1e3 minimum AGC target, charges excluded above |3| and a 10 s dynamic exclusion window. Internal and external standards were included for quality control purposes, with blank injections between every unique sample. QC mix was injected at the start and end of the injection sequence to ensure the stability of the signal through time and consisted of 30 compounds spanning a large range of *m/z*, RT and detectable in both positive and negative mode. Extracted ion chromatograms for internal standard compounds were evaluated using MZmine version 2.26^50^ to ensure consistency between injections. Samples were analyzed using Metabolite Atlas^50^ (https://github.com/biorack/metatlas). Briefly, a retention time corrected compound library generated by linear regression comparison of QC standards against an in house retention time (RT)-*m/z*-MSMS library of reference compounds analyzed using the same LCMS methods was used for compound identification in samples where measured RT, *m/z* and fragmentation spectra were compared with library predicted RT, theoretical m/z, library detected adducts and library MSMS fragmentation spectra. Compounds identification were retained when peak intensity was >1e4, retention time difference from predicted was <1 min, *m/z* was <20 ppm from theoretical, expected adduct was detected and at least one ion fragment matched the library spectra and were more abundant in at least one sample as compared to the average value + 1 SD of the extraction controls. Only eight compounds met these criteria; average peak heights from the extracted ion chromatograms are reported in Fig. S5. The signal was overall very low owing to the low amount of organics in these soils. We checked for the presence of methionine manually using MZmine version 2.26^32^ and confirmed that there were no detectable amount of methionine in any of the sample analyzed. Metabolomics data has been deposited JGI genome portal #1207416 along with the analysis file #1207417.

### Reporting summary

Further information on research design is available in the Nature Research Reporting Summary linked to this article.

## Supplementary information

Supplementary information

Description of Additional Supplementary Files

Supplementary Data 1

Reporting Summary

Source Data

## Data Availability

The 16S rRNA gene sequences from the libraries constructed for this study have been deposited to Genebank under the Bioproject ID PRJNA475109 [https://www.ncbi.nlm.nih.gov/bioproject/PRJNA475109]. The 16S rRNA genes from the ENIGMA culture collection are included in Supplementary Data 1. The raw flow cytometer data collected are displayed in Supplementary Figs. 4 and 5. The LCMS data and analysis are publicly available from the Joint Genome Institute Genome Portal: https://genome.jgi.doe.gov/portal/201CAT_FD/201CAT_FD.info.html. The data file is #1207416 and the analysis file is #1207417. Source data for Figs. 1c, d, 2b, 3 and 4, and Supplementary Figs. 1b and 2c, are provided as a Source Data file.
