# Supplementary material for: Probing the active fraction of soil microbiomes using BONCAT-FACS
Source: Nat Commun. 2019 Jun 24;10:2770. doi: 10.1038/s41467-019-10542-0 (PMC6591230; doi:10.1038/s41467-019-10542-0)
Supplement: Supplementary file 1 — Supplementary information [file 41467_2019_10542_MOESM1_ESM.pdf]

Supplementary information

Probing the active fraction of soil microbiomes using BONCAT-FACS

Couradeau et al.

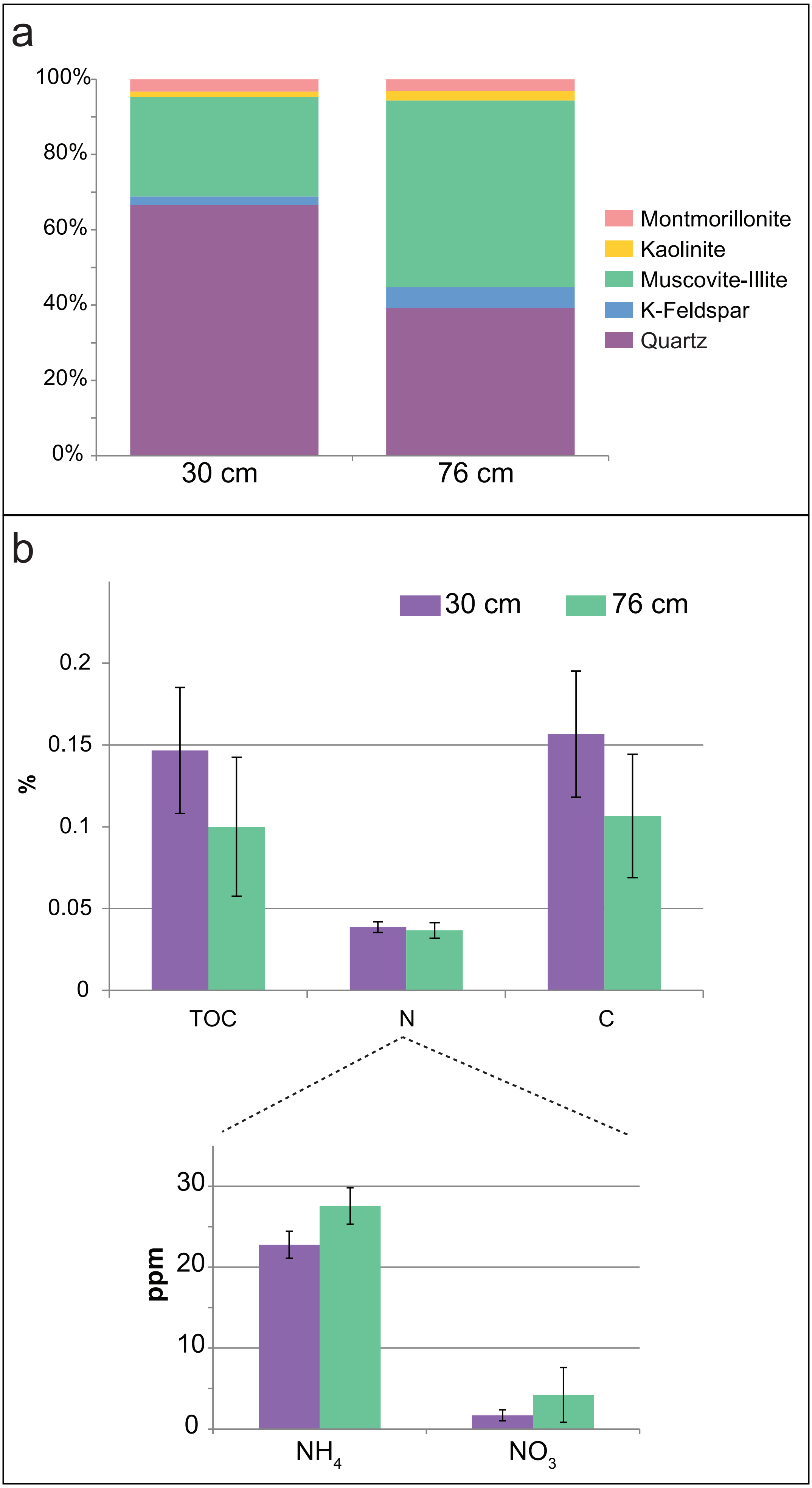

Supplementary Figure 1

Soils properties (a) Mineral composition (b) Total C, TOC, Total N, ammonium and nitrate concentration Error bars represent standard deviation (n=3). Ppm, parts per million.

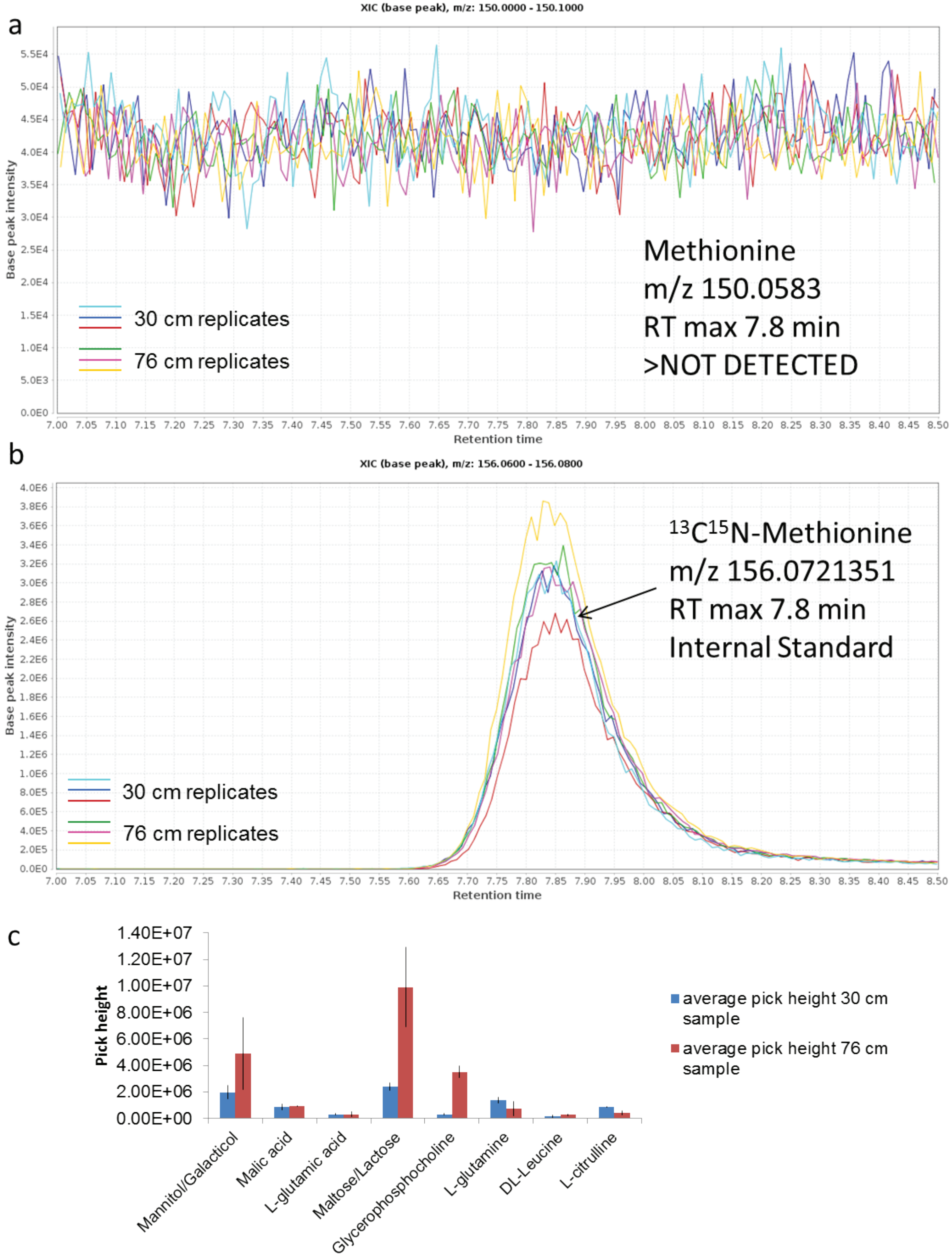

Supplementary Figure 2

LC-MS analysis of the full soil water extract showing that (a) there is no detectable amount of methionine, although (b) the spiked labeled heavy methionine was easily detected in all samples. (c) Peak height of the 8 compounds that passed our identification criteria (see methods). Data are means ± SD (n=3).

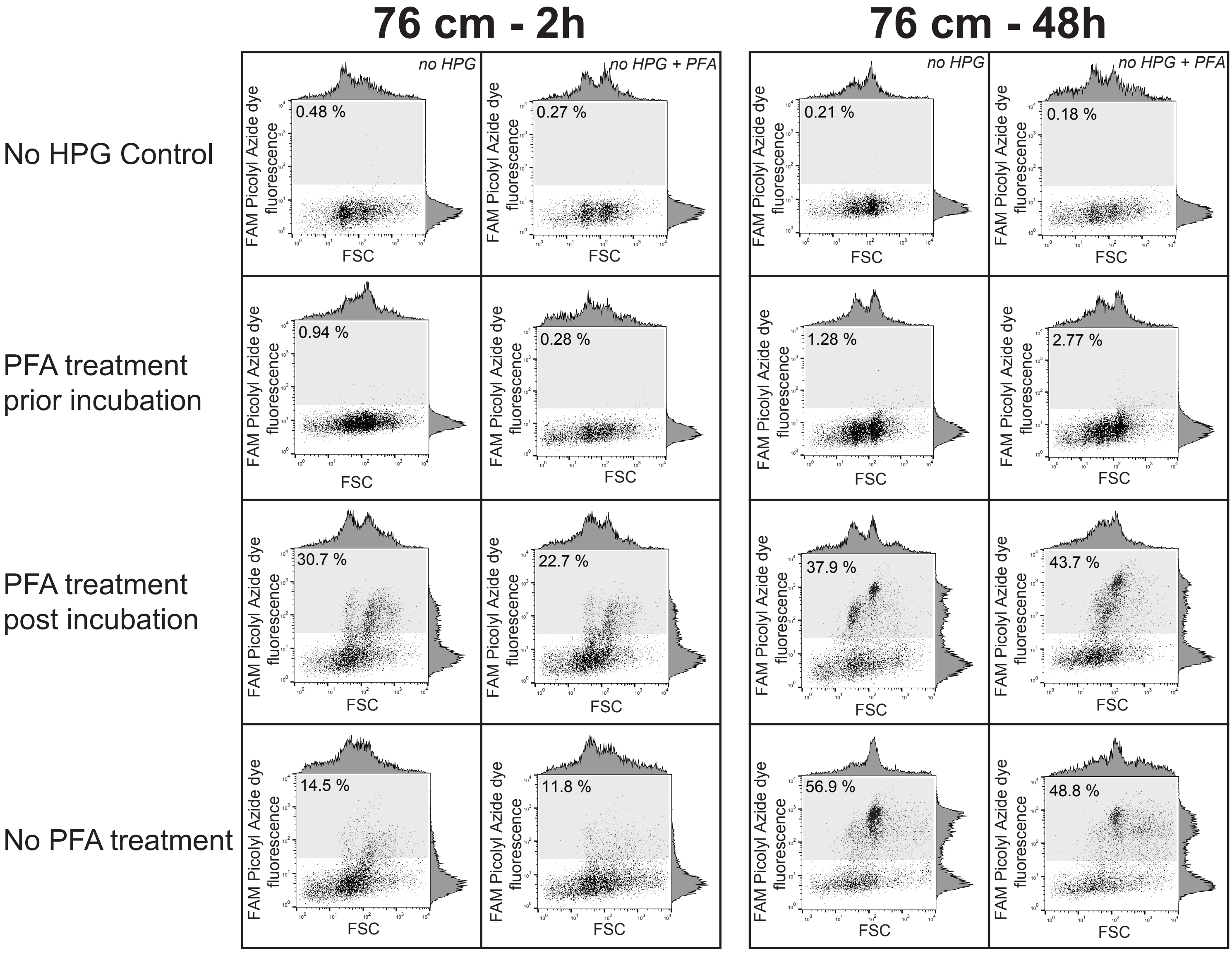

Supplementary Figure 3

BONCAT labeling of fixed and unfixed cells. Cells stained with SYTO dye plotted according to their forward scatter signal (FSC, x-axis) and BONCAT fluorescence, y-axis) in log-log scale. The distribution of the events along the x and y-axis is shown respectively on the density plot on the top and on the right of each graph. The BONCAT+ cells gate is displayed as a gray box in each plot, and the percent cells in the BONCAT+ gate is indicated in the top left corner of the box. The left two columns are biological replicates from 76 cm soil incubated for 2 h, and the right two for 48 h. Each row of panels corresponds to a different treatment, the first row being control samples without HPG (with or without PFA fixation), the second corresponds to samples that were pre-treated with PFA before incubation, while the third corresponds to samples were cells were fixed with PFA after incubation. The last row corresponds samples that were not fixed, i.e the same treatment used for when sorting and sequencing BONCAT+ and BONCAT– cells.

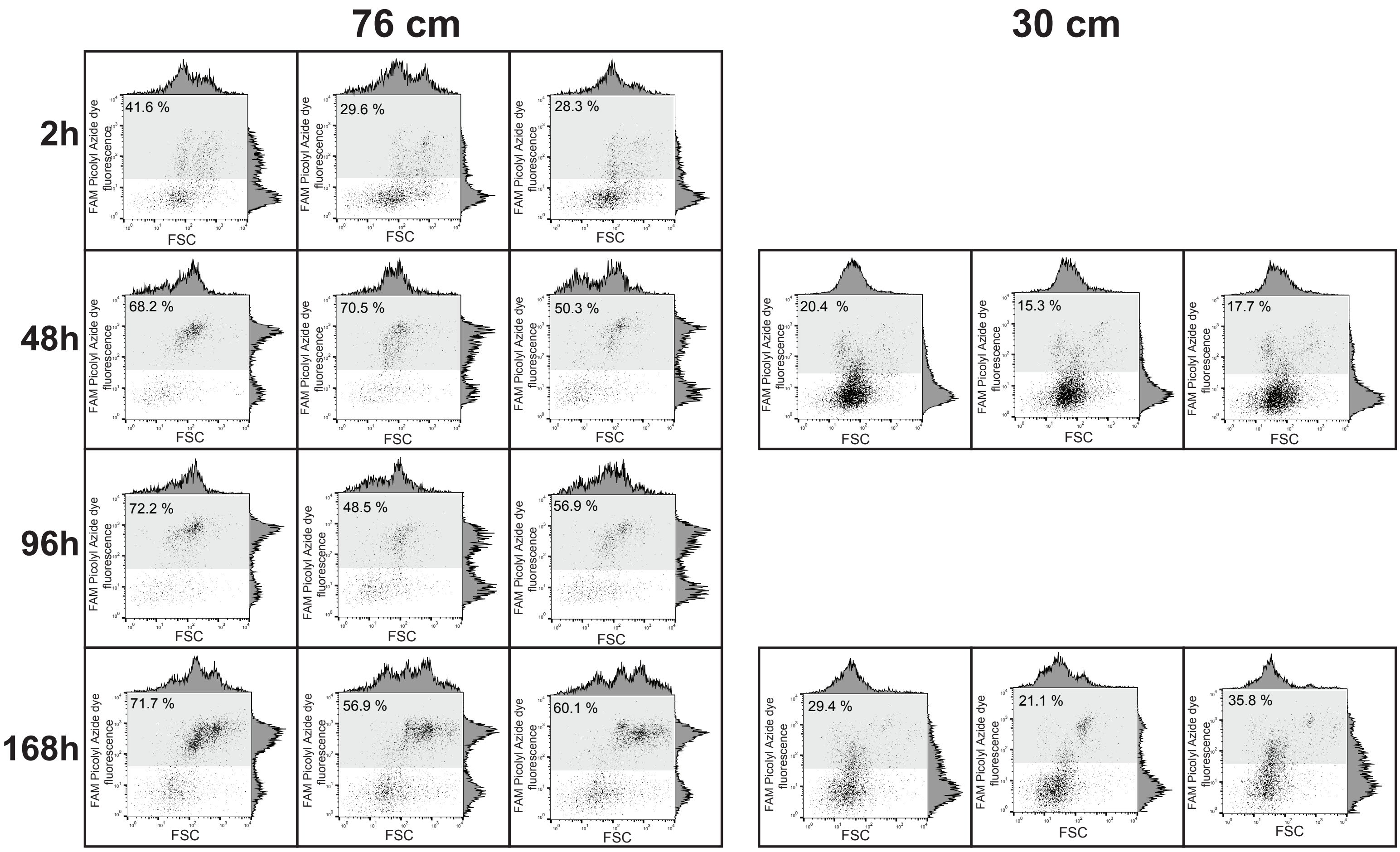

Supplementary Figure 4

BONCAT labelling over time of the 30 cm and the 76 cm samples. Cells stained with SYTO dye plotted according to their forward scatter signal (FSC, x-axis) and BONCAT fluorescence (Ex: 488nm/Em: 530nm, y-axis) in log-log scale. The distribution of the events along the x and y-axis is shown respectively on the density plot on the top and on the right of each graph. The left three columns are biological replicates from 76 cm and the right three from 30 cm soil. Each row corresponds to an incubation time (2 h to 168 h). The BON-CAT+ cells gate is displayed as a gray box in each plot, and the percent cells in the BONCAT+ gate is indicated in the top left corner of the box.

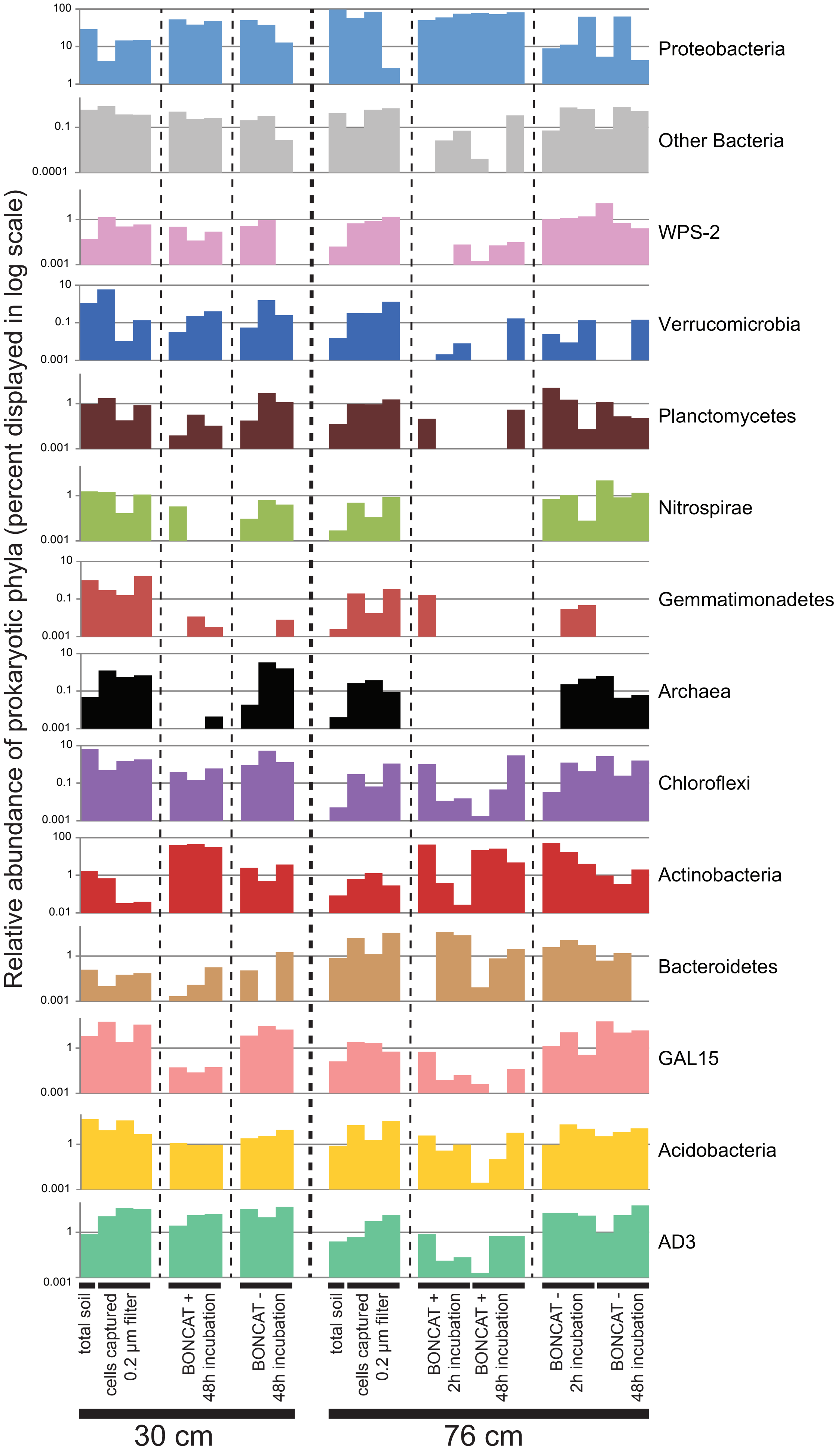

Supplementary Figure 5

Relative abundance of prokaryotic phyla displayed in log scale. The data displayed are the same than the Figure 2A, however they are plotted phylum by phylum and in log scale therefore allowing a better comparison of the low abundance phyla.

Supplementary table 1: Sample description, pooling strategy and alpha diversity metric of the iTag libraries

Sample ID	Sample depth	Incubation time	Type	Number of cells pooled	Frequency* per sample before filtering contaminants	Frequency* per sample without contaminant	ESV** count	OTU count (97% sim. cluster)
30cm_48h_A_BONCAT+	30 cm	48h	BONCAT +	50000	258672	257606	138	97
30cm_48h_A_BONCAT-	30 cm	48h	BONCAT -	75000	140530	136389	77	51
30cm_48h_B_BONCAT+	30 cm	48h	BONCAT +	75000	236375	234877	203	144
30cm_48h_B_BONCAT-	30 cm	48h	BONCAT -	75000	82431	81186	171	123
30cm_48h_D_BONCAT+	30 cm	48h	BONCAT +	75000	279152	278404	178	126
30cm_48h_D_BONCAT-	30 cm	48h	BONCAT -	75000	155187	89480	139	105
76cm_2h_A_BONCAT+	76 cm	2h	BONCAT +	35000	163551	163183	194	131
76cm_2h_A_BONCAT-	76 cm	2h	BONCAT -	50000	122347	120373	74	53
76cm_2h_B_BONCAT+	76 cm	2h	BONCAT +	65000	240574	240285	199	143
76cm_2h_B_BONCAT-	76 cm	2h	BONCAT -	75000	154938	152259	55	42
76cm_2h_C_BONCAT+	76 cm	2h	BONCAT +	73000	149499	149499	268	187
76cm_2h_C_BONCAT-	76 cm	2h	BONCAT -	75000	115293	115293	166	109
76cm_48h_A_BONCAT+	76 cm	48h	BONCAT +	75000	736900	736231	161	129
76cm_48h_A_BONCAT-	76 cm	48h	BONCAT -	45000	146726	130866	57	38
76cm_48h_B_BONCAT+	76 cm	48h	BONCAT +	75000	257443	257311	403	269
76cm_48h_B_BONCAT-	76 cm	48h	BONCAT -	35000	127528	113817	126	87
76cm_48h_C_BONCAT+	76 cm	48h	BONCAT +	50000	484680	482851	613	441
76cm_48h_C_BONCAT-	76 cm	48h	BONCAT -	75000	164342	160961	135	99
76cm_A_filter	76 cm	-	FILTER	-	305866	305866	528	381
76cm_B_filter	76 cm	-	FILTER	-	336054	336054	561	421
76cm_C_filter	76 cm	-	FILTER	-	175457	175457	373	281
30cm_A_filter	30 cm	-	FILTER	-	327415	327415	567	406
30cm_B_filter	30 cm	-	FILTER	-	260298	260298	412	296
30cm_C_filter	30 cm	-	FILTER	-	141273	141273	338	246
76cm_total_soil	76 cm	-	SOIL	-	349341	349341	390	324
30cm_total_soil	30 cm	-	SOIL	-	314201	314201	916	620

*Number of sequences after QC and denoising, note that DADA2 removed all singletons

**ESV: Exact Sequence Variant

Supplementary table 2: Experimental design and cell recovery rate from the killed control (PFA treated) samples

Sample ID	Pre-incubation treatment	Incubation (2h)	Post-incubation treatment	total extracted cell count per g of soil	average recovery per g of soil
pre_incubation_PFA_2h_A	PFA	HPG 50 µM		3.7E+07	**2.8E+07**
pre_incubation_PFA_2h_B	PFA	HPG 50 µM		1.9E+07	
PFA_water_control	PFA	water		NA	NA
post_incubation_PFA_2h_A		HPG 50 µM	PFA	1.6E+07	**1.6E+07**
post_incubation_PFA_2h_B		HPG 50 µM	PFA	1.5E+07	
HPG_2h_A		HPG 50 µM		2.7E+07	**2.8E+07**
HPG_2h_B		HPG 50 µM		2.9E+07	
water control		water		NA	NA
pre_incubation_PFA_48h_A	PFA	HPG 50 µM		1.7E+07	**2.1E+07**
pre_incubation_PFA_48h_B	PFA	HPG 50 µM		2.4E+07	
PFA_water_control	PFA	water		NA	NA
post_incubation_PFA_48h_A		HPG 50 µM	PFA	1.9E+07	**2.1E+07**
post_incubation_PFA_48h_B		HPG 50 µM	PFA	2.4E+07	
HPG_48h_A		HPG 50 µM		2.2E+07	**2.5E+07**
HPG_48h_B		HPG 50 µM		2.7E+07	
water control		water		NA	NA

Supplementary table 3: Cells recovery rate in PBS according to cell extraction method (from 30 cm soil)

	Vortex (5min)	Sonication (5min)*	0.02% Tween addition	0.22 µm filter capture**	Recovery (cell per g of soil)
**1**	x				1.19E+07
**2**	x	x			1.99E+07
**3**	x		x		2.57E+07
**4**	x	x	x		2.62E+07
**5**	x		x	x	1.53E+07
**6**	x	x	x	x	1.99E+07

**The sonication (5 min in sonication bath) step did not improve the cell recovery rate so this step was omitted in the final protocol*

***After being capture on a 0.22 µm filter the cells were released by vortexing the filter for 5 min in PBS + 0.02% Tween*
