# Supplementary material for: Probing the active fraction of soil microbiomes using BONCAT-FACS
Source: Nat Commun. 2019 Jun 24;10:2770. doi: 10.1038/s41467-019-10542-0 (PMC6591230; doi:10.1038/s41467-019-10542-0)
Supplement: Supplementary file 2 — Description of Additional Supplementary Files [file 41467_2019_10542_MOESM2_ESM.pdf]

Description of Additional Supplementary Files

File Name: Supplementary Data 1

Description: Partial 16S Ribosomal RNA genes sequences from the ENIGMA culture collection.
