# Supplementary material for: Probing the active fraction of soil microbiomes using BONCAT-FACS
Source: Nat Commun. 2019 Jun 24;10:2770. doi: 10.1038/s41467-019-10542-0 (PMC6591230; doi:10.1038/s41467-019-10542-0)
Supplement: Supplementary file 3 — Supplementary Data 1 [file 41467_2019_10542_MOESM3_ESM.docx]

**Partial 16S ribosomal gene sequences from the ENIGMA collection**

>FW305-3

ATGCAGTCGAGCGGTAGAGAGGTGCTTGCACCTCTTGAGAGCGGCGGACGGGTGAGTAATGCCTAGGAATCTGCCTGGTAGTGGGGGATAACGTTCGGAAACGGACGCTAATACCGCATACGTCCTACGGGAGAAAGCAGGGGACCTTCGGGCCTTGCGCTATCAGATGAGCCTAGGTCGGATTAGCTAGTTGGTGGGGTAATGGCTCACCAAGGCGACGATCCGTAACTGGTCTGAGAGGATGATCAGTCACACTGGAACTGAGACACGGTCCAGACTCCTACGGGAGGCAGCAGTGGGGAATATTGGACAATGGGCGAAAGCCTGATCCAGCCATGCCGCGTGTGTGAAGAAGGTCTTCGGATTGTAAAGCACTTTAAGTTGGGAGGAAGGGTACTTACCTAATACGTGAGTATTTTGACGTTACCGACAGAATAAGCACCGGCTAACTCTGTGCCAGCAGCCGCGGTAATACAGAGGGTGCAAGCGTTAATCGGAATTACTGGGCGTAAAGCGCGCGTAGGTGGTTCGTTAAGTTGGATGTGAAATCCCCGGGCTCAACCTGGGAACTGCATCCAAAACTGGCGAGCTAGAGTATGGTAGAGGGTGGTGGAATTTCCTGTGTAGCGGTGAAATGCGTAGATATAGGAAGGAACACCAGTGGCGAAGGCGACCACCTGGACTGATACTGACACTGAGGTGCGAAAGCGTGGGGAGCAAACAGGATTAGATACCCTGGTAGTCCACGCCGTAAACGATGTCAACTAGCCGTTGGGAGCCTTGAGCTCTTAGTGGCGCAGCTAACGCATTAAGTTGACCGCCTGGGGAGTACGGCCGCAAGGTTAAAACTCAAATGAATTGACGGGGGCCCGCACAAGCGGTGGAGCATGTGGTTTAATTCGAAGCAACGCGAAGAACCTTACCAGGCCTTGACATCCAATGAACTTTCCAGAGATGGATTGGTGCCTTCGGGAACATTGAGACAGGTGCTGCATGGCTGTCGTCAGCTCGTGTCGTGAGATGTTGGGTTAAGTCCCGTAACGAGCGCAACCCTTGTCCTTAGTTACCAGCACGTAATGGTGGGCACTCTAAGGAGACTGCCGGTGACAAACCGGAGGAAGGTGGGGATGACGTCAAGTCATCATGGCCCTTACGGCCTGGGCTACACACGTGCTACAATGGTCGGTACAGAGGGTTGCCAAGCCGCGAGGTGGAGCTAATCCCACAAAACCGATCGTAGTCCGGATCGCAGTCTGCAACTCGACTGCGTGAAGTCGGAATCGCTAGTAATCGCGAATCAGAATGTCGCGGTGAATACGTTCCCGGGCCTTGTACACACCGCCCGTCACACCATGGGAGTGGGTTGCACCAGAAGTAGCTAGTATAACCTTCGGGAGGACG

>FW305-12

TCCTGGCGGCGAGTGGCGAACGGGTGAGTAATACATCGGAACGTGCCCAATCGTGGGGGATAACGCAGCGAAAGCTGTGCTAATACCGCATACGATCTACGGATGAAAGCAGGGGATCGCAAGACCTTGCGCGAATGGAGCGGCCGATGGCAGATTAGGTAGTTGGTGAGGTAAAGGCTCACCAAGCCTTCGATCTGTAGCTGGTCTGAGAGGACGACCAGCCACACTGGGACTGAGACACGGCCCAGACTCCTACGGGAGGCAGCAGTGGGGAATTTTGGACAATGGGCGCAAGCCTGATCCAGCCATGCCGCGTGCAGGATGAAGGCCTTCGGGTTGTAAACTGCTTTTGTACGGAACGAAACGGCCTTTTCTAATAAAGAGGGCTAATGACGGTACCGTAAGAATAAGCACCGGCTAACTACGTGCCAGCAGCCGCGGTAATACGTAGGGTGCAAGCGTTAATCGGAATTACTGGGCGTAAAGCGTGCGCAGGCGGTTATGTAAGACAGTTGTGAAATCCCCGGGCTCAACCTGGGAACTGCATCTGTGACTGCATAGCTAGAGTACGGTAGAGGGGGATGGAATTCCGCGTGTAGCAGTGAAATGCGTAGATATGCGGAGGAACACCGATGGCGAAGGCAATCCCCTGGACCTGTACTGACGCTCATGCACGAAAGCGTGGGGAGCAAACAGGATTAGATACCCTGGTAGTCCACGCCCTAAACGATGTCAACTGGTTGTTGGGAATTCACTTTCTCAGTAACGAAGCTAACGCGTGAAGTTGACCGCCTGGGGAGTACGGCCGCAAGGTTGAAACTCAAAGGAATTGACGGGGACCCGCACAAGCGGTGGATGATGTGGTTTAATTCGATGCAACGCGAAAAACCTTACCCACCTTTGACATGTACGGAATTCGCCAGAGATGGCTTAGTGCTCGAAAGAGAACCGTAACACAGGTGCTGCATGGCTGTCGTCAGCTCGTGTCGTGAGATGTTGGGTTAAGTCCCGCAACGAGCGCAACCCTTGTCATTAGTTGCTACATTTAGTTGGGCACTCTAATGAGACTGCCGGTGACAAACCGGAGGAAGGTGGGGATGACGTCAAGTCCTCATGGCCCTTATAGGTGGGGCTACACACGTCATACAATGGCTGGTACAAAGGGTTGCCAACCCGCGAGGGGGAGCTAATCCCATAAAACCAGTCGTAGTCCGGATCGCAGTCTGCAACTCGACTGCGTGAAGTCGGAATCGCTAGTAATCGTGGATCAGAATGTCACGGTGAATACGTTCCCGGGTCTTGTACACACCGCCCGTCACACCATGGGAGCGGGTTCTGCCAGAAGTAGTTAGC

>FW305-17

CGGGAGCTTGCTCCTTGATTCAGCGGCGGACGGGTGAGTAATGCCTAGGAATCTGCCTGGTAGTGGGGGACAACGTTTCGAAAGGAACGCTAATACCGCATACGTCCTACGGGAGAAAGCAGGGGACCTTCGGGCCTTGCGCTATCAGATGAGCCTAGGTCGGATTAGCTAGTAGGTGAGGTAATGGCTCACCTAGGCGACGATCCGTAACTGGTCTGAGAGGATGATCAGTCACACTGGAACTGAGACACGGTCCAGACTCCTACGGGAGGCAGCAGTGGGGAATATTGGACAATGGGCGAAAGCCTGATCCAGCCATGCCGCGTGTGTGAAGAAGGTCTTCGGATTGTAAAGCACTTTAAGTTGGGAGGAAGGGCAGTAAGCTAATACCTTGCTGTTTTGACGTTACCGACAGAATAAGCACCGGCTAACTCTGTGCCAGCAGCCGCGGTAATACAGAGGGTGCAAGCGTTAATCGGAATTACTGGGCGTAAAGCGCGCGTAGGTGGTTCGTTAAGTTGGATGTGAAAGCCCCGGGCTCAACCTGGGAACTGCATCCAAAACTGGCGAGCTAGAGTATGGTAGAGGGTGGGTGGAAATTTCCTGTGTAGCGGTGAAATGCGTAGATATAGGAAGGAACACCAGTGGCGAAGGCGACCACCTGGACTGATACTGACACTGAGGTGCGAAAGCGTGGGGAGCAAACAGGATTAGATACCCTGGTAGTCCACGCCGTAAACGATGTCAACTAGCCGTTGGAATCCTTGAGATTTTAGTGGCGCAGCTAACGCATTAAGTTGACCGCCTGGGGAGTACGGCCGCAAGGTTAAAACTCAAATGAATTGACGGGGGCCCGCACAAGCGGTGGAGCATGTGGTTTAATTCGAAGCAACGCGAAGAACCTTACCAGGCCTTGACATGCAGAGAACTTTCCAGAGATGGATTGGTGCCTTCGGGAACTCTGACACAGGTGCTGCATGGCTGTCGTCAGCTCGTGTCGTGAGATGTTGGGTTAAGTCCCGTAACGAGCGCAACCCTTGTCCTTAGTTACCAGCACGTTATGGTGGGCACTCTAAGGAGACTGCCGGTGACAAACCGGAGGAAGGTGGGGATGACGTCAAGTCATCATGGCCCTTACGGCCTGGGCTACACACGTGCTACAATGGTCGGTACAGAGGGTTGCCAAGCCGCGAGGTGGAGCTAATCTCACAAAACCGATCGTAGTCCGGATCGCAGTCTGCAACTCGACTGCGTGAAGTCGGAATCGCTAGTAATCGCAAATCAGAATGTTGCGGTGAATACGTTCCCGGGCCTTGTACACACCGCCCGTCACACCATGGGAGTGGGTTGCACCAGAAGTAGCTAGTCTAACCTTC

>FW305-19

GCAGTCGAACGGCAGCACGGGCTTCGGCCTGGTGGCGAGTGGCGAACGGGTGAGTAATACATCGGAACGTGCCCTGTAGTGGGGGATAACTAGTCGAAAGATTAGCTAATACCGCATACGACCTGAGGGTGAAAGCGGGGGACCGGTAACGGCCTCGCGCTACGAGAGCGGGCCGCGGTAATACGTAGGGTGCGAGCGTTNATCGGAATTACTGGGCGTAAAGCGTGCGCMSGCGGTTTTGTAAGACAGGCGTGAAATCCCCGAGCTCAACTTGGGAATGGCGCTTGTGACTGCAAGGCTAGAGTATGTCAGAGGGGGGTAGAATTCCACGTGTAGCAGTGAAATGCGTAGAGATGTGGAGGAATACCGATGGCGAAGGCAGCCCCCTGGGACGTCACTGACGCTCATGCACGAAAGCGTGGGGAGCAAACAGGATTAGATACCCTGGTAGTCCACGCCCTAAACGATGTCAACTAGTTGTTGGGGATTCATTTCTTCAGTAACGTAGCTAACGCGTGAAGTTGACCGCCTGGGGAGTACGGTCGCAAGATTAAAACTCAAAGGAATTGACGGGGACCCGCACAAGCGGTGGATGATGTGGATTAATTCGATGCAACGCGAAAAACCTTACCTACCCTTGACATGCCACTAACGAAGCAGAGATGCATNAGGTGCCCGAAAGGGAAAGTGGACACAGGTGCTGCATGGCNGTCGTCAGCTCGTGTCGTGAGATGTTGGGTTAAGTCCCGCAACGAGCGCAACCCTTGTCTCTAGTTGCTACGAAAGGGCACTCTAGAGAGACTGCCGGTGACAAACCGGAGGAAGGTGGGGATGACGTCAAGTCCTCATGGCCCTTATGGGTAGGGCTTCACACGTCATACAATGGTGCGTACAGAGGGTTGCCAACCCGCGAGGGGGAGCTAATCCCAGAAAACGCATCGTAGTCCGGATCGTAGTCTGCAACTCGACTACGTGAAGCNGGAATCGCTNGTAATCGCGGATCAGCATGCCGCGGTGAATACGTTCCCGGGTCTTGTACACACCGCCCGTCACACCATGGGAGTGGGTTTTGCCAGAAGTAGTTAGCCTAACCGC

>FW305-20

GCACGGGTACTTGTACCTGGTGGCGAGCGGCGGACGGGTGAGTAATGCCTAGGAATCTGCCTGGTAGTGGGGGATAACGCTCGGAAACGGACGCTAATACCGCATACGTCCTACGGGAGAAAGCAGGGGACCTTCGGGCCTTGCGCTATCAGATGAGCCTAGGTCGGATTAGCTAGTTGGTGAGGTAATGGCTCACCAAGGCGACGATCCGTAACTGGTCTGAGAGGATGATCAGTCATCACTGGAACTGAGACACGGTCCAGACTCCTACGGGAGGCAGCAGTGGGGAATATTGGACAATGGGCGAAAGCCTGATCCAGCCATGCCGCGTGTGTGAAGAAGGTCTTCGGATTGTAAAGCACTTTAAGTTGGGAGGAAGGGCATTTACCTAATACGTAAGTGTTTTGACGTTACCGACAGAATAAGCACCGGCTAACTCTGTGCCAGCAGCCGCGGTAATACAGAGGGTGCAAGCGTTAATCGGAATTACTGGGCGTAAAGCGCGCGTAGGTGGTTCGTTAAGTTGGATGTGAAATCCCCGGGCTCAACCTGGGAACTGCATTCAAAACTGTCGAGCTAGAGTATGGTAGAGGGTGGGTGGAAATTTCCTGTGTAGCGGTGAAATGCGTAGATATAGGAAGGAACACCAGTGGCGAAGGCGACCACCTGGACTGATACTGACACTGAGGTGCGAAAGCGTGGGGAGCAAACAGGATTAGATACCCTGGTAGTCCACGCCGTAAACGATGTCAACTAGCCGTTGGGAGCCTTGAGCTCTTAGTGGCGCAGCTAACGCATTAAGTTGACCGCCTGGGGAGTACGGCCGCAAGGTTAAAACTCAAATGAATTGACGGGGGCCCGCACAAGCGGTGGAGCATGTGGTTTAATTCGAAGCAACGCGAAGAACCTTACCAGGCCTTGACATCCAATGAACTTTCCAGAGATGGATTGGTGCCTTCGGGAACATTGAGACAGGTGCTGCATGGCTGTCGTCAGCTCGTGTCGTGAGATGTTGGGTTAAGTCCCGTAACGAGCGCAACCCTTGTCCTTAGTTACCAGCACGTAATGGTGGGCACTCTAAGGAGACTGCCGGTGACAAACCGGAGGAAGGTGGGGATGACGTCAAGTCATCATGGCCCTTACGGCCTGGGCTACACACGTGCTACAATGGTCGGTACAGAGGGTTGCCAAGCCGCGAGGTGGAGCTAATCCCAGAAAACCGATCGTAGTCCGGATCGCAGTCTGCAACTCGACTGCGTGAAGTTGGAATCGCTNGTAATCGCGAATCAGAATGTCGCGGTGAATACGTTCCCGGGCCTTGTACACACCGCCCGTCACACCATGGGAGTGGGTTGCACCAGAAGTAGCNTAGTCTAACCTTCGGGAGGAC

>FW305-20A

GCAGTCGAACGGCAGCACGGGCTTCGGCCTGGTGGCGAGTGGCGAACGGGTGAGTAATACATCGGAACGTGCCCTGTAGTGGGGGATAACTAGTCGAAAGATTAGCTAATACCGCATACGACCTGAGGGTGAAAGCGGGGGACCGCAAGGCCTCGCGCTACAGGAGCGGCCGATGTCTGATTAGCTAGTTGGTGGGGTAAGAGCCTACCAAGGCGACGATCAGTAGCTGGTCTGAGAGGACGATCAGCCACACTGGGACTGAGACACGGCCCAGACTCCTACGGGAGGCAGCAGTGGGGAATTTTGGACAATGGGGGCAACCCTGATCCAGCAATGCCGCGTGTGTGAAGAAGGCCTTCGGGTTGTAAAGCACTTTTGTCCGGAAAGAAATGGCTCTGGTTAATACCCGGGGTCGATGACGGTACCGGAAGAATAAGCACCGGCTAACTACGTGCCAGCAGCCGCGGTAATACGTAGGGTGCGAGCGTTAATCGGAATTACTGGGCGTAAAGCGTGCGCAGGCGGTTTTGTAAGACAGGCGTGAAATCCCCGAGCTCAACTTGGGAATGGCGCTTGTGACTGCAAGGCTAGAGTATGTCAGAGGGGGGTAGAATTCCACGTGTAGCAGTGAAATGCGTAGAGATGTGGAGGAATACCGATGGCGAAGGCAGCCCCCTGGGACGTCACTGACGCTCATGCACGAAAGCGTGGGGAGCAAACAGGATTAGATACCCTGGTAGTCCACGCCCTAAACGATGTCAACTAGTTGTTGGGGATTCATTTCTTCAGTAACGTAGCTAACGCGTGAAGTTGACCGCCTGGGGAGTACGGTCGCAAGATTAAAACTCAAAGGAATTGACGGGGACCCGCACAAGCGGTGGATGATGTGGATTAATTCGATGCAACGCGAAAAACCTTACCTACCCTTGACATGCCACTAACGAAGCAGAGATGCATTAGGTGCCCGAAAGGGAAAGTGGACACAGGTGCTGCATGGCTGTCGTCAGCTCGTGTCGTGAGATGTTGGGTTAAGTCCCGCAACGAGCGCAACCCTTGTCTCTAGTTGCTACGAAAGGGCACTCTAGAGAGACTGCCGGTGACAAACCGGAGGAAGGTGGGGATGACGTCAAGTCCTCATGGCCCTTATGGGTAGGGCTTCACACGTCATACAATGGTGCGTACAGAGGGTTGCCAACCCGCGAGGGGGAGCTAATCCCAGAAAACGCATCGTAGTCCGGATCGTAGTCTGCAACTCGACTACGTGAAGCCGGAATCGCTAGTAATCGCGGATCAGCATGCCGCGGTGAATACGTTCCCGGGTCTTGTACACACCGCCCGTCACACCATGGGAGTGGGTTTTGCCAGAAGTAGTTAGCCTAACCGCAAGGA

>FW305-20B

GCAGTCGAACGGCAGCGCGGGAGCAATCCTGGCGGCGAGTGGCGAACGGGTGAGTAATACATCGGAACGTGCCCAATCGTGGGGGATAACGCAGCGAAAGCTGTGCTAATACCGCATACGATCTACGGATGAAAGCAGGGGATCGCAAGACCTTGCGCGAATGGAGCGGCCGATGGCAGATTAGGTAGTTGGTGAGGTAAAGGCTCACCAAGCCTTCGATCTGTAGCTGGTCTGAGAGGACGACCAGCCACACTGGGACTGAGACACGGCCCAGACTCCTACGGGAGGCAGCAGTGGGGAATTTTGGACAATGGGCGCAAGCCTGATCCAGCCATGCCGCGTGCAGGATGAAGGCCTTCGGGTTGTAAACTGCTTTTGTACGGAACGAAACGGCCTTTTCTAATAAAGAGGGCTAATGACGGTACCGTAAGAATAAGCACCGGCTAACTACGTGCCAGCAGCCGCGGTAATACGTAGGGTGCAAGCGTTAATCGGAATTACTGGGCGTAAAGCGTGCGCAGGCGGTTATGTAAGACAGTTGTGAAATCCCCGGGCTCAACCTGGGAACTGCATCTGTGACTGCATAGCTAGAGTACGGTAGAGGGGGGATGGAATTCCGCGTGTAGCAGTGAAATGCGTAGATATGCGGAGGAACACCGATGGCGAAGGCAATCCCCTGGACCTGTACTGACGCTCATGCACGAAAGCGTGGGGAGCAAACAGGATTAGATACCCTGGTAGTCCACGCCCTAAACGATGTCAACTGGGTTGTTGGGAATTCACTTTCTCAGTAACGAAGCTAACGCGTGAAGTTGACCGCCTGGGGAGTACGGCCGCAAGGTTGAAACTCAAAGGAATTGACGGGGACCCGCACAAGCGGTGGATGATGTGGTTTAATTCGATGCAACGCGAAAAACCTTACCCACCTTTTGACATGTACGGAATTCGCCAGAGATGGCGTAGTGCTCGAAAGAGAACCGTAACACAGGTGCTGCATGGCTGTCGTCAGCTCGTGTCGTGAGATGTTTGGGGTTAAGTCCCGCAACGAGCGCAACCCTTTGTCATTAGTTGCTACATTTAGTTGGGCACTCTAATGAGACTGCCGGTGACAAACCGGAGGAAGGTGGGGATGACGTCAAGTCCTCATGGCCCTTATAGGTGGGGCTACACACGTCATACAATGGCTGGTACAAAGGGTTGCCAACCCGCGAGGGGGAGCTAATCCCATAAAACCAGTCGTAGTCCGGATCGCAGTCTGCAACTCGACTGCGTGAAGTCGGAATCGCTAGTAATCGTGGATCAGAATGTCACGGTGAATACGTTCCCGGGTCTTGTACACACCGCCCGTCACACCATGGGAGCGGGTTCTGCCAGAAGTAGTTAGCTTAACCGCAAGGA

>FW305-21

TGCAGTCGAACGGCAGCACGGGAGCAATCCTGGTGGCGAGTGGCGAACGGGTGAGTAATACATCGGAACGTGCCCTGTCGTGGGGGATAACTAGTCGAAAGATTAGCTAATACCGCATACGACCTGAGGGTGAAAGCGGGGGACCGTAAGGCCTCGCGCGATAGGAGCGGCCGATGTCTGATTAGCTAGTTGGTGGGGTAAAGGCCCACCAAGGCGACGATCAGTAGCTGGTCTGAGAGGACGATCAGCCACACTGGGACTGAGACACGGCCCAGACTCCTACGGGAGGCAGCAGTGGGGAATTTTGGACAATGGGGGCAACCCTGATCCAGCAATGCCGCGTGTGTGAAGAAGGCCTTCGGGTTGTAAAGCACTTTTGTCCGGAAAGAAATCCCTTGCTCTAATACAGCGGGGGGGATGACGGTACCGGAAGAATAAGCACCGGCTAACTACGTGCCAGCAGCCGCGGTAATACGTAGGGTGCGAGCGTTAATCGGAATTACTGGGCGTAAAGCGTGCGCAGGCGGTTTTGTAAGACAGGCGTGAAATCCCCGAGCTCAACTTGGGAATGGCGCTTGTGACTGCAAGGCTAGAGTATGTCAGAGGGGGGTAGAATTCCACGTGTAGCAGTGAAATGCGTAGAGATGTGGAGGAATACCGATGGCGAAGGCAGCCCCCTGGGACGTCACTGACGCTCATGCACGAAAGCGTGGGGAGCAAACAGGATTAGATACCCTGGTAGTCCACGCCCTAAACGATGTCAACTAGTTGTTGGGGATTCATTTCTTCAGTAACGTAGCTAACGCGTGAAGTTGACCGCCTGGGGAGTACGGTCGCAAGATTAAAACTCAAAGGAATTGACGGGGACCCGCACAAGCGGTGGATGATGTGGATTAATTCGATGCAACGCGAAAAACCTTACCTACCCTTGACATGCCACTAACGAAGCAGAGATGCATTAGGTGCCCGAAAGGGAAAGTGGACACAGGTGCTGCATGGCTGTCGTCAGCTCGTGTCGTGAGATGTTGGGTTAAGTCCCGCAACGAGCGCAACCCTTGTCTTTAGTTGCTACGCAAGAGCACTCTAGAGAGACTGCCGGTGACAAACCGGAGGAAGGTGGGGATGACGTCAAGTCCTCATGGCCCTTATGGGTAGGGCTTCACACGTCATACAATGGTGCGTACAGAGGGTTGCCAACCCGCGAGGGGGAGCTAATCCCAGAAAACGCATCGTAGTCCGGATCGTAGTCTGCAACTCGACTACGTGAAGCCGGAATCGCTAGTAATCGCGGATCAGCATGCCGCGGTGAATACGTTCCCGGGTCTTGTACACACCGCCCGTCACACCATGGGAGTGGGTTTTGCCAGAAGTAGTTAGCCTAACCGCAAGGA

>FW305-22

CGACGGCAGCACGGGAGCAATCCTGGTGGCGAGTGGCGAACGGGTGAGTAATACATCGGAACGTGCCCTGTCGTGGGGGATAACTAGTCGAAAGATTAGCTAATACCGCATACGACCTGAGGGTGAAAGCGGGGGACCGTAAGGCCTCGCGCGATAGGAGCGGCCGATGTCTGATTAGCTAGTTGGTGGGGTAAAGGCCCACCAAGGCGACGATCAGTAGCTGGTCTGAGAGGACGATCAGCCACACTGGGACTGAGACACGGCCCAGACTCCTACGGGAGGCAGCAGTGGGGAATTTTGGACAATGGGGGCAACCCTGATCCAGCAATGCCGCGTGTGTGAAGAAGGCCTTCGGGTTGTAAAGCACTTTTGTCCGGAAAGAAATCCCTTGCTCTAATACAGCGGGGGGGATGACGGTACCGGAAGAATAAGCACCGGCTAACTACGTGCCAGCAGCCGCGGTAATACGTAGGGTGCGAGCGTTAATCGGAATTACTGGGCGTAAAGCGTGCGCAGGCGGTTTTGTAAGACAGGCGTGAAATCCCCGAGCTCAACTTGGGAATGGCGCTTGTGACTGCAAGGCTAGAGTATGTCAGAGGGGGGTAGAATTCCACGTGTAGCAGTGAAATGCGTAGAGATGTGGAGGAATACCGATGGCGAAGGCAGCCCCCTGGGACGTCACTGACGCTCATGCACGAAAGCGTGGGGAGCAAACAGGATTAGATACCCTGGTAGTCCACGCCCTAAACGATGTCAACTAGTTGTTGGGGATTCATTTCTTCAGTAACGTAGCTAACGCGTGAAGTTGACCGCCTGGGGAGTACGGTCGCAAGATTAAAACTCAAAGGAATTGACGGGGACCCGCACAAGCGGTGGATGATGTGGATTAATTCGATGCAACGCGAAAAACCTTACCTACCCTTGACATGCCACTAACGAAGCAGAGATGCATTAGGTGCCCGAAAGGGAAAGTGGACACAGGTGCTGCATGGCTGTCGTCAGCTCGTGTCGTGAGATGTTGGGTTAAGTCCCGCAACGAGCGCAACCCTTGTCTTTAGTTGCTACGCAAGAGCACTCTAGAGAGACTGCCGGTGACAAACCGGAGGAAGGTGGGGATGACGTCAAGTCCTCATGGCCCTTATGGGTAGGGCTTCACACGTCATACAATGGTGCGTACAGAGGGTTGCCAACCCGCGAGGGGGAGCTAATCCCAGAAAACGCATCGTAGTCCGGATCGTAGTCTGCAACTCGACTACGTGAAGCCGGAATCGCTAGTAATCGCGGATCAGCATGCCGCGGTGAATACGTTCCCGGGTCTTGTACACACCGCCCGTCACACCATGGGAGTGGGTTTTGCCAGAAGTAGTTAGCCTAACCGCAAGGA

>FW305-23

GCAGTCGAACGGCAGCACGGGCTTCGGCCTGGTGGCGAGTGGCGAACGGGTGAGTAATACATCGGAACGTGCCCTGTAGTGGGGGATAACTAGTCGAAAGATTAGCTAATACCGCATACGACCTGAGGGTGAAAGCGGGGGACCGCAAGGCCTCGCGCTACAGGAGCGGCCGATGTCTGATTAGCTAGTTGGTGGGGTAAAAGCCTACCAAGGCGACGATCAGTAGCTGGTCTGAGAGGACGATCAGCCACACTGGGACTGAGACACGGCCCAGACTCCTACGGGAGGCAGCAGTGGGGAATTTTGGACAATGGGGGCAACCCTTGATCCAGCAATGCCGCGTGTGTGAAGAAGGCCTTCGGGTTGTAAAGCACTTTTGTCCGGAAAGAAATGGCTCTGGTTAATACCCGGGGTCGATGACGGTACCGGAAGAATAAGCACCGGCTAACTACGTGCCAGCAGCCGCGGTAATACGTAGGGTGCGAGCGTTAATCGGAATTACTGGGCGTAAAGCGTGCGCAGGCGGTTTTGTAAGACAGGCGTGAAATCCCCGAGCTCAACTTGGGAATGGCGCTTGTGACTGCAAGGCTAGAGTATGTCAGAGGGGGGTAGAATTCCACGTGTAGCAGTGAAATGCGTAGAGATGTGGAGGAATACCGATGGCGAAGGCAGCCCCCTGGGACGTCACTGACGCTCATGCACGAAAGCGTGGGGAGCAAACAGGATTAGATACCCTGGTAGTCCACGCCCTAAACGATGTCAACTAGTTGTTGGGGATTCATTTCTTCAGTAACGTAGCTAACGCGTGAAGTTGACCGCCTGGGGAGTACGGTCGCAAGATTAAAACTCAAAGGAATTGACGGGGACCCGCACAAGCGGTGGATGATGTGGATTAATTCGATGCAACGCGAAAAACCTTACCTACCCTTGACATGCCACTAACGAAGCAGAGATGCATTAGGTGCCCGAAAGGGAAAGTGGACACAGGTGCTGCATGGCTGTCGTCAGCTCGTGTCGTGAGATGTTGGGTTAAGTCCCGCAACGAGCGCAACCCTTGTCTCTAGTTGCTACGAAAGGGCACTCTAGAGAGACTGCCGGTGACAAACCGGAGGAAGGTGGGGATGACGTCAAGTCCTCATGGCCCTTATGGGTAGGGCTTCACACGTCATACAATGGTGCGTACAGAGGGTTGCCAACCCGCGAGGGGGAGCTAATCCCAGAAAACGCATCGTAGTCCGGATCGTAGTCTGCAACTCGACTACGTGAAGCCGGAATCGCTAGTAATCGCGGATCAGCATGCCGCGGTGAATACGTTCCCGGGTCTTGTACACACCGCCCGTCACACCATGGGAGTGGGTTTTGCCAGAAGTAGTTAGCCTAACCGCAAGGA

>FW305-25

TGCAGTCGAGCGGTAGAGAGGTGCTTGCACCTCTTGAGAGCGGCGGACGGGTGAGTAATGCCTAGGAATCTGCCTGGTAGTGGGGGATAACGTTCGGAAACGGACGCTAATACCGCATACGTCCTACGGGAGAAAGCAGGGGACCTTCGGGCCTTGCGCTATCAGATGAGCCTAGGTCGGATTAGCTAGTTGGTGGGGTAATGGCTCACCAAGGCGACGATCCGTAACTGGTCTGAGAGGATGATCAGTCACACTGGAACTGAGACACGGTCCAGACTCCTACGGGAGGCAGCAGTGGGGAATATTGGACAATGGGCGAAAGCCTGATCCAGCCATGCCGCGTGTGTGAAGAAGGTCTTCGGATTGTAAAGCACTTTAAGTTGGGAGGAAGGGTACTTACCTAATACGTGAGTATTTTGACGTTACCGACAGAATAAGCACCGGCTAACTCTGTGCCAGCAGCCGCGGTAATACAGAGGGTGCAAGCGTTAATCGGAATTACTGGGCGTAAAGCGCGCGTAGGTGGTTCGTTAAGTTGGATGTGAAATCCCCGGGCTCAACCTGGGAACTGCATCCAAAACTGGCGAGCTAGAGTATGGTAGAGGGTGGTGGAATTTCCTGTGTAGCGGTGAAATGCGTAGATATAGGAAGGAACACCAGTGGCGAAGGCGACCACCTGGACTGATACTGACACTGAGGTGCGAAAGCGTGGGGAGCAAACAGGATTAGATACCCTGGTAGTCCACGCCGTAAACGATGTCAACTAGCCGTTGGGAGCCTTGAGCTCTTAGTGGCGCAGCTAACGCATTAAGTTGACCGCCTGGGGAGTACGGCCGCAAGGTTAAAACTCAAATGAATTGACGGGGGCCCGCACAAGCGGTGGAGCATGTGGTTTAATTCGAAGCAACCGCGAAGAACCTTACCAGGCCTTGACATCCAATGAACTTTCCAGAGATGGATTGGTGCCTTCGGGAACATTGAGACAGGTGCTGCATGGCTGTCGTCAGCTCGTGTCGTGAGATGTTGGGTTAAGTCCCGTAACGAGCGCAACCCTTGTCCTTAGTTACCAGCACGTAATGGTGGGCACTCTAAGGAGACTGCCGGTGACAAACCGGAGGAAGGTGGGGATGACGTCAAGTCATCATGGCCCTTACGGCCTGGGCTACACACGTGCTACAATGGTCGGTACAGAGGGTTGCCAAGCCGCGAGGTGGAGCTAATCCCACAAAACCGATCGTAGTCCGGATCGCAGTCTGCAACTCGACTGCGTGAAGTTGGAATCGCTAGTAATCGCGAATCAGAATGTCGCGGTGAATACGTTCCCGGGCCTTGTACACACCGCCCGTCACACCATGGGAGTGGGTTGCACCAGAAGTAGCTAGTCTAACCTTCGGGAGGAC

>FW305-29A

GCAGTCGAACGGTAACAGGTTAAGCTGACGAGTGGCGAACGGGTGAGTAATGTATCGGAACGTGCCCAGTCGTGGGGGATAACTGCTCGAAAGAGCAGCTAATACCGCATACGACCTGAGGGTGAAAGCGGGGGATCGCAAGACCTCGCGCGATTGGAGCGGCCGATATCAGATTAGGTAGTTGGTGGGGTAAAGGCTCACCAAGCCAACGATCTGTAGCTGGTCTGAGAGGACGACCAGCCACACTGGGACTGAGACACGGCCCAGACTCCTACGGGAGGCAGCAGTGGGGAATTTTGGACAATGGACGCAAGTCTGATCCAGCCATGCCGCGTGCGGGAAGAAGGCCTTCGGGTTGTAAACCGCTTTTGTCAGGGAAGAAAAGGTTCTGGTTAATACCTGGGACTCATGACGGTACCTGAAGAATAAGCACCGGCTAAACTACGTGCCAGCAGCCGCGGTAATACGTAGGGTGCAAGCGTTAATCGGAATTACTGGGCGTAAAGCGTGCGCAGGCGGTTATGCAAGACAGAGGTGAAATCCCCGGGCTCAACCTGGGAACTGCCTTTGTGACTGCATAGCTAGAGTACGGTAGAGGGGGGATGGAATTCCGCGTGTAGCAGTGAAATGCGTAGATATGCGGAGGAACACCGATGGCGAAGGCAATCCCCTGGACCTGTACTGACGCTCATGCACGAAAGCGTGGGGAGCAAACAGGATTAGATACCCTGGTAGTCCACGCCCTAAACGATGTCAACTGGTTGTTGGGGAGGGTTTCTTCTCAGTAACGAAGCTAACGCGTGAAGTTGACCGCCTGGGGAGTACGGCCGCAAGGTTGAAACTCAAAGGAATTGACGGGGACCCGCACAAGCGGTGGATGATGTGGGTTTAATTCGATGCAACGCGAAAAACCTTACCTACCCTTGACATGCCAGGAATCCTGAAGAGATTTGGGAGTGCTCGAAAGAGAGCCTGGACACAGGTGCTGCATGGCCGTCGTCAGCTCGTGTCGTGAGATGTTGGGTTAAGTCCCGCAACGAGCGCAACCCTTGTCATTAGTTGCTACGAAAGGGCACTCTAATGAGACTGCCGGTGACAAACCGGAGGAAGGTGGGGATGACGTCAGGTCATCATGGCCCTTATGGGTAGGGCTACACACGTCATACAATGGCCGGGACAGAGGGCTGCCAACCCGCGAGGGGGAGCTAATCCCAGAAACCCGGTCGTAGTCCGGATCGTAGTCTGCAACTCGACTGCGTGAAGTCGGAATCGCTAGTAATCGCGGATCAGCTTGCCGCGGTGAATACGTTCCCGGGTCTTGTACACACCGCCCGTCACACCATGGGAGCGGGTTCTGCCAGAAGTAGTTAGCCTAACCGCAAGG

>FW305-29B

GCAGTCGAACGGTAACAGGTTAAGCTGACGAGTGGCGAACGGGTGAGTAATGTATCGGAACGTGCCCAGTCGTGGGGGATAACTGCTCGAAAGAGCAGCTAATACCGCATACGACCTGAGGGTGAAAGCGGGGGATCGCAAGACCTCGCGCGATTGGAGCGGCCGATATCAGATTAGGTAGTTGGTGGGGTAAAGGCTCACCAAGCCAACGATCTGTAGCTGGTCTGAGAGGACGACCAGCCACACTGGGACTGAGACACGGCCCAGACTCCTACGGGAGGCAGCAGTGGGGAATTTTGGACAATGGACGCAAGTCTGATCCAGCCATGCCGCGTGCGGGAAGAAGGCCTTCGGGTTGTAAACCGCTTTTGTCAGGGAAGAAAAGGTTCTGGTTAATACCTGGGACTCATGACGGTACCTGAAGAATAAGCACCGGCTAACTACGTGCCAGCAGCCGCGGTAATACGTAGGGTGCAAGCGTTAATCGGAATTACTGGGCGTAAAGCGTGCGCAGGCGGTTATGCAAGACAGAGGTGAAATCCCCGGGCTCAACCTGGGAACTGCCTTTGTGACTGCATAGCTAGAGTACGGTAGAGGGGGGATGGAATTCCGCGTGTAGCAGTGAAATGCGTAGATATGCGGAGGAACACCGATGGCGAAGGCAATCCCCTGGACCTGTACTGACGCTCATGCACGAAAGCGTGGGGAGCAAACAGGATTAGATACCCTGGTAGTCCACGCCCTAAACGATGTCAACTGGTTGTTGGGGAGGGTTTCTTCTCAGTAACGAAGCTAACGCGTGAAGTTGACCGCCTGGGGAGTACGGCCGCAAGGTTGAAACTCAAAGGAATTGACGGGGACCCGCACAAGCGGTGGATGATGTGGTTTAATTCGATGCAACGCGAAAAACCTTACCTACCCTTGACATGCCAGGAATCCTGAAGAGATTTGGGAGTGCTCGAAAGAGAGCCTGGACACAGGTGCTGCATGGCCGTCGTCAGCTCGTGTCGTGAGATGTTGGGTTAAGTCCCGCAACGAGCGCAACCCTTGTCATTAGTTGCTACGAAAGGGCACTCTAATGAGACTGCCGGTGACAAACCGGAGGAAGGTGGGGATGACGTCAGGTCATCATGGCCCTTATGGGTAGGGCTACACACGTCATACAATGGCCGGGACAGAGGGCTGCCAACCCGCGAGGGGGAGCTAATCCCAGAAACCCGGTCGTAGTCCGGATCGTAGTCTGCAACTCGACTGCGTGAAGTTGGAATCGCTAGTAATCGCGGATCAGCTTGCCGCGGTGAATACGTTCCCGGGTCTTGTACACACCGCCCGTCACACCATGGGAGCGGGTTCTGCCAGAAGTAGTTAGCCTAACCGCAAGGGGGGCGAT

>FW305-35

CAGTCGAACGGCAGCACAGGGCTTCGGCCTGGTGGCGAGTGGCGAACGGGTGAGTAATACATCGGAACGTGCCCTGTAGTGGGGGATAACTAGTCGAAAGATTAGCTAATACCGCATACGACCTGAGGGTGAAAGCGGGGGACCGGTNNGNGCCTCGCNCTACGAGAGCNNNNNNTNTCNGANTANCTTTTTNGNGGGGTAANNNCCNNCNNGNGNNNNNATNNNNNNNNGGTCAGAGANGANNATNNNCCACTGNGNNANNGANACNNNGCCCACACNCCNANGGGNGGCNNCAGTGGGGAATTTTGGMCAATGGGGGCAACCCNTGATNCCNNCAATGCCGCGTGTGNGAAAGAAGGCCTTNNGGGTTGTAAAGCNTTTTTNTCCGGAAAGAAANTGGCTTTGGTTAATACCCGGGGNNCGATGACGGTACCGGGAAGAATAAGCACCGGCTNAATTACGTGCCAGCAGCCGCGGTAATACGTAGGGTGCGAGCGTTAATCGGAATTACTGGGCGTAAAGCGTGCGCCGGCGGTTTTGTAAGACAGGCGTGAAATCCCCGAGCTCAACTTGGGAATGGCGCTTGTGACTGCAAGGCTAGAGTATGTCAGAGGGGGGTAGAATTCCACGTGTAGCAGTGAAATGCGTAGAGATGTGGAGGAATACCGATGGCGAAGGCAGCCCCCTGGGACGTCACTGACGCTCATGCACGAAAGCGTGGGGAGCAAACAGGATTAGATACCCTGGTAGTCCACGCCCTAAACGATGTCAACTAGTTGTTGGGGATTCATTTCTTCAGTAACGTAGCTAACGCGTGAAGTTGACCGCCTGGGGAGTACGGTCGCAAGATTAAAACTCAAAGGAATTGACGGGGACCCGCACAAGCGGTGGATGATGTGGATTAATTCGATGCAACGCGAAAAACCTTACCTACCCTTGACATGCCACTAACGAAGCAGAGATGCATTAGGTGCCCGAAAGGGAAAGTGGACACAGGTGCTGCATGGCTGTCGTCAGCTCGTGTCGTGAGATGTTGGGTTAAGTCCCGCAACGAGCGCAACCCTTGTCTCTAGTTGCTACGAAAGGGCACTCTAGAGAGACTGCCGGTGACAAACCGGAGGAAGGTGGGGATGACGTCAAGTCCTCATGGCCCTTATGGGTAGGGCTTCACACGTCATACAATGGTGCGTACAGAGGGTTGCCAACCCGCGAGGGGGAGCTAATCCCAGAAAACGCATCGTAGTCCGGATCGTAGTCTGCAACTCGACTACGTGAAGCCGGAATCGCTGTAATCGCGGATCAGCATGCCGCGGTGAATACGTTCCCGGGTCTTGTACACACCGCCCGTCACACCATGGGAGTGGGTTTTGCCAGAAGTAGTTAGCCTAACCGC

>FW305-42

AAATGCAGTCGAGCGGATGACGGGAGCTTGCTCCTTGATTCAGCGGCGGACGGGTGAGTAATGCCTAGGAATCTGCCTGGTAGTGGGGGACAACGTTTCGAAAGGAACGCTAATACCGCATACGTCCTACGGGAGAAAGCAGGGGACCTTCGGGCCTTGCGCTATCAGATGAGCCTAGGTCGGATTAGCTAGTAGGTGAGGTAATGGCTCACCTAGGCGACGATCCGTAACTGGTCTGAGAGGATGATCAGTCACACTGGAACTGAGACACGGTCCAGACTCCTACGGGAGGCAGCAGTGGGGAATATTGGACAATGGGCGAAAGCCTGATCCAGCCATGCCGCGTGTGTGAAGAAGGTCTTCGGATTGTAAAGCACTTTAAGTTGGGAGGAAGGGCAGTAAGCTAATACCTTGCTGTTTTGACGTTACCGACAGAATAAGCACCGGCTAACTCTGTGCCAGCAGCCGCGGTAATACAGAGGGTGCAAGCGTTAATCGGAATTACTGGGCGTAAAGCGCGCGTAGGTGGTTCGTTAAGTTGGATGTGAAAGCCCCGGGCTCAACCTGGGAACTGCATCCAAAACTGGCGAGCTAGAGTATGGTAGAGGGTGGGTGGAAATTTCCTGTGGTAGCGGTGAAATGCGTAGATATNGGAAGGAACACCAGTGGCGAAGGCGACCACCTGGACTGATACTGACACTGAGGTGCGAAAGCGTGGGGAGCAAACAGGATTAGATACCCTGGTAGTCCACGCCGTAAACGATGTCAACTAGCCGTTGGAATCCTTGAGATTTTAGTGGCGCAGCTAACGCATTAAGTTGACCGCCTGGGGAGTACGGCCGCAAGGTTAAAACTCAAATGAATTGACGGGGGCCCGCACAAGCGGTGGAGCATGTGGTTTAATTCGAAGCAACGCGAAGAACCTTACCAGGCCTTGACATGCAGAGAACTTTCCAGAGATGGATTGGTGCCTTCGGGAACTCTGACACAGGTGCTGCATGGCTGTCGTCAGCTCGTGTCGTGAGATGTTGGGTTAAGTCCCGTAACGAGCGCAACCCTTGTCCTTAGTTACCAGCACGTTATGGTGGGCACTCTAAGGAGACTGCCGGTGACAAACCGGAGGAAGGTGGGGATGACGTCAAGTCATCATGGCCCTTACGGCCTGGGCTACACACGTGCTACAATGGTCGGTACAGAGGGTTGCCAAGCCGCGAGGTGGAGCTAATCTCACAAAACCGATCGTAGTCCGGATCGCAGTCTGCAACTCGACTGCGTGAAGTCGGAATCGCTAGTAATCGCAAATCAGAATGTTGCGGTGAATACGTTCCCGGGCCTTGTACACACCGCCCGTCACACCATGGGAGTGGGTTGCACCAGAAGTAGCTAGTCTAACCTTCGGGAGGAC

>FW305-43

GACGGCAGCACGGGCTTCGGCCTGGTGGCGAGTGGCGAACGGGTGAGTAATACATCGGAACGTGCCCTGTAGTGGGGGATAACTAGTCGAAAGATTAGCTAATACCGCAACGACCTGAGGGTGAAAGCGGGGGACCGCAAGGCCTCGCGCTACAGGAGCGGCCGATGTCTGATTAGCTAGTTGGTGGGGTAAAAGCCTACCAAGGCGACGATCAGTAGCTGGTCTGAGAGGACGATCAGCCACACTGGGACTGAGACACGGCCCAGACTCCTACGGGAGGCAGCAGTGGGGAATTTTGGACAATGGGGGCAACCCTGATCCAGCAATGCCGCGTGTGTGAAGAAGGCCTTCGGGTTGTAAAGCACTTTTGTCCGGAAAGAAATGGCTCTGGTTAATACCCGGGGTCGATGACGGTACCGGAAGAATAAGCACCGGCTAACTACGTGCCAGCAGCCGCGGTAATACGTAGGGTGCGAGCGTTAATCGGAATTACTGGGCGTAAAGCGTGCGCAGGCGGTTTTGTAAGACAGGCGTGAAATCCCCGAGCTCAACTTGGGAATGGCGCTTGTGACTGCAAGGCTAGAGTATGTCAGAGGGGGGTAGAATTCCACGTGTAGCAGTGAAATGCGTAGAGATGTGGAGGAATACCGATGGCGAAGGCAGCCCCCTGGGACGTCACTGACGCTCATGCACGAAAGCGTGGGGAGCAAACAGGATTAGATACCCTGGTAGTCCACGCCCTAAACGATGTCAACTAGTTGTTGGGGATTCATTTCTTCAGTAACGTAGCTAACGCGTGAAGTTGACCGCCTGGGGAGTACGGTCGCAAGATTAAAACTCAAAGGAATTGACGGGGACCCGCACAAGCGGTGGATGATGTGGATTAATTCGATGCAACGCGAAAAACCTTACCTACCCTTGACATGCCACTAACGAAGCAGAGATGCATTAGGTGCCCGAAAGGGAAAGTGGACACAGGTGCTGCATGGCTGTCGTCAGCTCGTGTCGTGAGATGTTGGGTTAAGTCCCGCAACGAGCGCAACCCTTGTCTCTAGTTGCTACGAAAGGGCACTCTAGAGAGACTGCCGGTGACAAACCGGAGGAAGGTGGGGATGACGTCAAGTCCTCATGGCCCTTATGGGTAGGGCTTCACACGTCATACAATGGTGCGTACAGAGGGTTGCCAACCCGCGAGGGGGAGCTAATCCCAGAAAACGCATCGTAGTCCGGATCGTAGTCTGCAACTCGACTACGTGAAGCCGGAATCGCTAGTAATCGCGGATCAGCATGCCGCGGTGAATACGTTCCCGGGTCTTGTACACACCGCCCGTCACACCATGGGAGTGGGTTTTGCCAGAAGTAGTTAGCCTAACCGCAAGGA

>FW305-44A

TGCAGTCGAGCGGTAAGGCCTTTCGGGGTACACGAGCGGCGAACGGGTGAGTAACACGTGGGTGATCTGCCCTGCACTTCGGGATAAGCCTGGGAAACTGGGTCTAATACCGGATATGACCTCAGGTTGCATGACTTGGGGTGGAAAGATTTATCGGTGCAGGATGGGCCCGCGGCCTATCAGCTTGTTGGTGGGGTAATGGCCTACCAAGGCGACGACGGGTAGCCGACCTGAGAGGGTGACCGGCCACACTGGGACTGAGACACGGCCCAGACTCCTACGGGAGGCAGCAGTGGGGAATATTGCACAATGGGCGAAAGCCTGATGCAGCGACGCCGCGTGAGGGATGACGGCCTTCGGGTTGTAAACCTCTTTCAGCAGGGACGAAGCGCAAGTGACGGTACCTGCAGAAGAAGCACCGGCTAACTACGTGCCAGCAGCCGCGGTAATACGTAGGGTGCAAGCGTTGTCCGGAATTACTGGGCGTAAAGAGTTCGTAGGCGGTTTGTCGCGTCGTTTGTGAAAACCAGCAGCTCAACTGCTGGCTTGCAGGCGATACGGGCAGACTTGAGTACTGCAGGGGAGACTGGAATTCCTGGTGTAGCGGTGAAATGCGCAGATATCAGGAGGAACACCGGTGGCGAAGGCGGGTCTCTGGGCAGTAACTGACGCTGAGGAACGAAAGCGTGGGTAGCGAACAGGATTAGATACCCTGGTAGTCCACGCCGTAAACGGTGGGCGCTAGGGTGTGGGTTCCTTCCACGGAATCCGTGCCGTAGCTAACGCATTAAGCGCCCCGCCTGGGGGAGTACGGCCGCAAGGCTAAAACTCAAAGGAATTGACGGGGGGCCCGCACAAGCGGCGGAGCATGTGGATTAATTCGATGCAACGCGAAGAACCTTACCTGGNGTTTGACATATACCGGAAAGCTGCAGAGATGTGGCCCCCCCTTGTGGTCCGGTATACAGGTGGTGCATGGGCTGTCGTCAGCTCGTGTCGTGAGATGTTGGGTTAAGTCCCGCAACGAGCGCAACCCCTATCTTATGTTGCCAGCACGTTATGGTGGGGACTCGTAAGAGACTGCCGGGGTCAACTCGGAGGAAGGTGGGGACGACGTCAAGTCATCATGCCCCTTATGTCCAGGGCTTCACACATGCTACAATGGCCAGTACAGAGGGCTGCGAGACCGTGAGGTGGAGCGAATCCCTTAAAGCTGGTCTCAGTTCGGATCGGGGTCTGCAACTCGACCCCGTGAAGTNGGAGTCGCTAGTAATCGCAGATCAGCAACGCTGCGGTGAATACGTTCCCGGGCCTTGTACACACCGCCCGTCACGTCATGAAAGTCGGTAACACCCGAAGCCGGTGGCTTAACCCCC

>FW305-44B

CTTTCGGGGTACACGAGCGGCGAACGGGTGAGTAACACGTGGGTGATCTGCCCTGCACTTCGGGATAAGCCTGGGAAACTGGGTCTAATACCGGATATGACCTCAGGTTGCATGACTTGGGGTGGAAAGATTTATCGGTGCAGGATGGGCCCGCGGCCTATCAGCTTGTTGGTGGGGTAATGGCCTACCAAGGCGACGACGGGTAGCCGACCTGAGAGGGTGACCGGCCACACTGGGACTGAGACACGGCCCAGACTCCTACGGGAGGCAGCAGTGGGGAATATTGCACAATGGGCGAAAGCCTGATGCAGCGACGCCGCGTGAGGGATGACGGCCTTCGGGTTGTAAACCTCTTTCAGCAGGGACGAAGCGCAAGTGACGGTACCTGCAGAAGAAGCACCGGCTAACTACGTGCCAGCAGCCGCGGTAATACGTAGGGTGCAAGCGTTGTCCGGAATTACTGGGCGTAAAGAGTTCGTAGGCGGTTTGTCGCGTCGTTTGTGAAAACCAGCAGCTCAACTGCTGGCTTGCAGGCGATACGGGCAGACTTGAGTACTGCAGGGGAGACTGGAATTCCTGGTGTAGCGGTGAAATGCGCAGATATCAGGAGGAACACCGGTGGCGAAGGCGGGTCTCTGGGCAGTAACTGACGCTGAGGAACGAAAGCGTGGGTAGCGAACAGGATTAGATACCCTGGTAGTCCACGCCGTAAACGGTGGGCGCTAGGTGTGGGTTCCTTCCACGGAATCCGTGCCGTAGCTAACGCATTAAGCGCCCCGCCTGGGGGAGTACGGCCGCAAGGCTAAAACTCAAAGGAATTGACGGGGGCCCGCACAAGCGGCGGAGCATGTGGATTAATTCGATGCAACGCGAAGAACCTTACCTGGGTTTGACATATACCGGAAAGCTGCAAGAGATGTGGCCCCCCCTTGTGGTCGGTATACAGGTGGTGCATGGCTGTCGTCAGCCTCGTGTCGTGAGATGTTGGGTTAAGTCCCGCAACGAGCGCAACCCCTATCTTATGTTGCCAGCACGTTATGGTGGGGACTCGTAAGAGACTGCCGGGGTCAACTCGGAGGAAGGTGGGGACGACGTCAAGTCATCATGCCCCTTATGTCCAGGGCTTCACACATGCTACAATGGCCAGTACAGAGGGCTGCGAGACCGTGAGGTGGAGCGAATCCCTTAAAGCTGGTCTCAGTTCGGATCGGGGTCTGCAACTCGACCCCGTGAAGTCGGAGTCGCTAGTAATCGCAGATCAGCAACGCTGCGGTGAATACGTTCCCGGGCCTTGTACACACCGCCCGTCACGTCATGAAAGTCGGTAACACCCGAAGCCGGTGGCTTA

>FW305-45

GCAGTCGAACGGCAGCACGGGCTTCGGCCTGGTGGCGAGTGGCGAACGGGTGAGTAATACATCGGAACGTGCCCTGTAGTGGGGGATAACTAGTCGAAAGATTAGCTAATACCGCATACGACCTGAGGGTGAAAGCGGGGGACCGCAAGGCCTCGCGCTACAGGAGCGGCCGATGTCTGATTAGCTAGTTGGTGGGGTAAAAGCCTACCAAGGCGACGATCAGTAGCTGGTCTGAGAGGACGATCAGCCACACTGGGACTGAGACACGGCCCAGACTCCTACGGGAGGCAGCAGTGGGGAATTTTGGACAATGGGGGCAACCCTGATCCAGCAATGCCGCGTGTGTGAAGAAGGCCTTCGGGTTGTAAAGCACTTTTGTCCGGAAAGAAATGGCTCTGGTTAATACCCGGGGTCGATGACGGTACCGGAAGAATAAGCACCGGCTAACTACGTGCCAGCAGCCGCGGTAATACGTAGGGTGCGAGCGTTAATCGGAATTACTGGGCGTAAAGCGTGCGCAGGCGGTTTTGTAAGACAGGCGTGAAATCCCCGAGCTCAACTTGGGAATGGCGCTTGTGACTGCAAGGCTAGAGTATGTCAGAGGGGGGTAGAATTCCACGTGTAGCAGTGAAATGCGTAGAGATGTGGAGGAATACCGATGGCGAAGGCAGCCCCCTGGGACGTCACTGACGCTCATGCACGAAAGCGTGGGGAGCAAACAGGATTAGATACCCTGGTAGTCCACGCCCTAAACGATGTCAACTAGTTGTTGGGGATTCATTTCTTCAGTAACGTAGCTAACGCGTGAAGTTGACCGCCTGGGGAGTACGGTCGCAAGATTAAAACTCAAAGGAATTGACGGGGACCCGCACAAGCGGTGGATGATGTGGATTAATTCGATGCAACGCGAAAAACCTTACCTACCCTTGACATGCCACTAACGAAGCAGAGATGCATTAGGTGCCCGAAAGGGAAAGTGGACACAGGTGCTGCATGGCCGTCGTCAGCTCGTGTCGTGAGATGTTGGGTTAAGTCCCGCAACGAGCGCAACCCTTGTCTCTAGTTGCTACGAAAGGGCACTCTAGAGAGACTGCCGGTGACAAACCGGAGGAAGGTGGGGATGACGTCAAGTCCTCATGGCCCTTATGGGTAGGGCTTCACACGTCATACAATGGTGCGTACAGAGGGTTGCCAACCCGCGAGGGGGAGCTAATCCCAGAAAACGCATCGTAGTCCGGATCGTAGTCTGCAACTCGACTACGTGAAGCCGGAATCGCTAGTAATCGCGGATCAGCATGCCGCGGTGAATACGTTCCCGGGTCTTGTACACACCGCCCGTCACACCATGGGAGTGGGTTTTGCCAGAAGTAGTTAGCCTAACCGCAAGGAGGGCGATATC

>FW305-47A

GCAGTCGAACGGCAGCACCGGGCTTCGGCCTGGTGGCGAGTGGCGAACGGGTGAGTAATACATCGGAACGTGCCCTGTAGTGGGGGATAACTAGTCGAAAGATTAGCTAATACCGCATACGACCTGAGGGTGAAAGCGGGGGACCGCAAGGCCTCGCGCTACAGGAGCGGCCGATGTCTGATTAGCTAGTTGGTGGGGTAAAAGCCTACCAAGGCGACGATCAGTAGCTGGTCTGAGAGGACGATCAGCCACACTGGGACTGAGACACGGCCCAGACTCCTACGGGAGGCAGCAGTGGGGAATTTTGGACAATGGGGGCAACCCTGATCCAGCAATGCCGCGTGTGTGAAGAAGGCCTTCGGGTTGTAAAGCACTTTTGTCCGGAAAGAAATGGCTCTGGTTAATACCCGGGGTCGATGACGGTACCGGAAGAATAAGCACCGGCTAACTACGTGCCAGCAGCCGCGGTAATACGTAGGGTGCGAGCGTTAATCGGAATTACTGGGCGTAAAGCGTGCGCAGGCGGTTTTGTAAGACAGGCGTGAAATCCCCGAGCTCAACTTGGGAATGGCGCTTGTGACTGCAAGGCTAGAGTATGTCAGAGGGGGGTAGAATTCCACGTGTAGCAGTGAAATGCGTAGAGATGTGGAGGAATACCGATGGCGAAGGCAGCCCCCTGGGACGTCACTGACGCTCATGCACGAAAGCGTGGGGAGCAAACAGGATTAGATACCCTGGTAGTCCACGCCCTAAACGATGTCAACTAGTTGTTGGGGATTCATTTCTTCAGTAACGTAGCTAACGCGTGAAGTTGACCGCCTGGGGAGTACGGTCGCAAGATTAAAACTCAAAGGAATTGACGGGGACCCGCACAAGCGGTGGATGATGTGGATTAATTCGATGCAACGCGAAAAACCTTACCTACCCTTGACATGCCACTAACGAAGCAGAGATGCATTAGGTGCCCGAAAGGGAAAGTGGACACAGGTGCTGCATGGCTGTCGTCAGCTCGTGTCGTGAGATGTTGGGTTAAGTCCCGCAACGAGCGCAACCCTTGTCTCTAGTTGCTACGAAAGGGCACTCTAGAGAGACTGCCGGTGACAAACCGGAGGAAGGTGGGGATGACGTCAAGTCCTCATGGCCCTTATGGGTAGGGCNTTCACACGTCATACAATGGTGCGTACAGAGGGTTGCCAACCCGCGAGGGGGAGCTAATCCCAGAAAACGCATCGTAGTCCGGATCGTAGTCTGCAACTCGACTACGTGAAGCCGGAATCGCTAGTAATCGCGGATCAGCATGCCGCGGTGAATACGTTCCCGGGTCTTGTACACACCGCCCGTCACACCATGGGAGTGGGTTTTGCCAGAAGTAGTTAGCCT

>FW305-47B

TGCAGTCGAGCGGCAGCACGGGTACTTGTACCTGGTGGCGAGCGGCGGACGGGTGAGTAATGCCTAGGAATCTGCCTGGTAGTGGGGGATAACGCTCGGAAACGGACGCTAATACCGCATACGTCCTACGGGAGAAAGCAGGGGACCTTCGGGCCTTGCGCTATCAGATGAGCCTAGGTCGGATTAGCTAGTTGGTGAGGTAATGGCTCACCAAGGCGACGATCCGTAACTGGTCTGAGAGGATGATCAGTCACACTGGAACTGAGACACGGTCCAGACGTCCTACGGGAGGCAGCAGTGGGGAATATTGGACAATGGGCGAAAGCCTGATCCAGCCATGCCGCGTGTGTGAAGAAGGTCTTCGGATTGTAAAGCACTTTAAGTTGGGAGGAAGGGCATTTACCTAATACGTAAGTGTTTTGACGTTACCGACAGAATAAGCACCGGCTAACTCTGTGCCAGCAGCCGCGGTAATACAGAGGGTGCAAGCGTTAATCGGAATTACTGGGCGTAAAGCGCGCGTAGGTGGTTCGTTAAGTTGGATGTGAAATCCCCGGGCTCAACCTGGGAACTGCATTCAAAACTGTCGAGCTAGAGTATGGTAGAGGGTGGGTGGAATTTCCTGTGTAGCGGTGAAATGCGTAGATATAGGAAGGAACACCAGTGGCGAAGGCGACCACCTGGACTGATACTGACACTGAGGTGCGAAAGCGTGGGGAGCAAACAGGATTAGATACCCTGGTAGTCCACGCCGTAAACGATGTCAACTAGCCGTTGGGAGCCTTGAGCTCTTAGTGGCGCAGCTAACGCATTAAGTTGACCGCCTGGGGAGTACGGCCGCAAGGTTAAAACTCAAATGAATTGACGGGGGCCCGCACAAGCGGTGGAGCATGTGGTTTAATTCGAAGCAACGCGAAGAACCTTACCAGGCCTTGACATCCAATGAACTTTCCAGAGATGGATTGGTGCCTTCGGGAACATTGAGACAGGTGCTGCATGGCTGTCGTCAGCTCGTGTCGTGAGATGTTGGGTTAAGTCCCGTAACGAGCGCAACCCTTGTCCTTAGTTACCAGCACGTAATGGTGGGCACTCTAAGGAGACTGCCGGTGACAAACCGGAGGAAGGTGGGGATGACGTCAAGTCATCATGGCCCTTACGGCCTGGGCTACACACGTGCTACAATGGTCGGTACAGAGGGTTGCCAAGCCGCGAGGTGGAGCTAATCCCAGAAAACCGATCGTAGTCCGGATCGCAGTCTGCAACTCGACTGCGTGAAGTCGGAATCGCTAGTAATCGCGAATCAGAATGTCGCGGTGAATACGTTCCCGGGCCTTGTACACACCGCCCGTCACACCATGGGAGTGGGTTGCACCAGAAGTAGCTAGTCTAACCTTCGGGAGGACGG

>FW305-53

TGCAGTCGAGCGGCAGCACGGGTACTTGTACCTGGTGGCGAGCGGCGGACGGGTGAGTAATGCCTAGGAATCTGCCTGGTAGTGGGGGATAACGTTCGGAAACGAACGCTAATACCGCATACGTCCTACGGGAGAAAGCAGGGGACCTTCGGGCCTTGCGCTATCAGATGAGCCTAGGTCGGATTAGCTAGTTGGTGAGGTAATGGCTCACCAAGGCGACGATCCGTAACTGGTCTGAGAGGATGATCAGTCACACTGGAACTGAGACACGGTCCAGACTCCTACGGGAGGCAGCAGTGGGGAATATTGGACAATGGGCGAAAGCCTGATCCAGCCATGCCGCGTGTGTGAAGAAGGTCTTCGGATTGTAAAGCACTTTAAGTTGGGAGGAAGGGCAGTTACCTAATACGTAATTGTTTTGACGTTACCGACAGAATAAGCACCGGCTAACTCTGTGCCAGCAGCCGCGGTAATACAGAGGGTGCAAGCGTTAATCGGAATTACTGGGCGTAAAGCGCGCGTAGGTGGTTCGTTAAGTTGGATGTGAAATCCCCGGGCTCAACCTGGGAACTGCATTCAAAACTGTCGAGCTAGAGTATGGTAGAGGGTGGGTGGAATTTTCCTGTGGTAGCGGTGAAATGCGTAGATATAGGAAGGAACACCAGTGGCGAAGGCGACCACCTGGACTGATACTGACACTGAGGTGCGAAAGCGTGGGGAGCAAACAGGATTAGATACCCTGGTAGTCCACGCCGTAAACGATGTCAACTAGCCGTTGGGAGCCTTGAGCTCTTAGTGGCGCAGCTAACGCATTAAGTTGACCGCCTGGGGAGTACGGCCGCAAGGTTAAAACTCAAATGAATTGACGGGGGCCCGCACAAGCGGTGGAGCATGTGGTTTAATTCGAAGCAACGCGAAGAACCTTACCAGGCCTTGACATCCAATGAACTTTCCAGAGATGGATTGGTGCCTTCGGGAACATTGAGACAGGTGCTGCATGGCTGTCGTCAGCTCGTGTCGTGAGATGTTGGGTTAAGTCCCGTAACGAGCGCAACCCTTGTCCTTAGTTACCAGCACGTAATGGTGGGCACTCTAAGGAGACTGCCGGTGACAAACCGGAGGAAGGTGGGGATGACGTCAAGTCATCATGGCCCTTACGGCCTGGGCTACACACGTGCTACAATGGTCGGTACAGAGGGTTGCCAAGCCGCGAGGTGGAGCTAATCCCATAAAACCGATCGTAGTCCGGATCGCAGTCTGCAACTCGACTGCGTGAAGTCGGAATCGCTAGTAATCGCGAATCAGAATGTCGCGGTGAATACGTTCCCGGGCCTTGTACACACCGCCCGTCACACCATGGGAGTGGGTTGCACCAGAAGTAGCTAGTCTA

>FW305-57

CACGGGTACTTGTACCTGGTGGCGAGCGGCGGACGGGTGAGTAATGCCTAGGAATCTGCCTGGTAGTGGGGGATAACGTTCGGAAACGAACGCTAATACCGCATACGTCCTACGGGAGAAAGCAGGGGACCTTCGGGCCTTGCGCTATCAGATGAGCCTAGGTCGGATTAGCTAGTTGGTGAGGTAATGGCTCACCAAGGCGACGATCCGTAACTGGTCTGAGAGGATGATCAGTCACACTGGAACTGAGACACGGTCCAGACTCCTACGGGAGGCAGCAGTGGGGAATATTGGACAATGGGCGAAAGCCTGATCCAGCCATGCCGCGTGTGTGAAGAAGGTCTTCGGATTGTAAAGCACTTTAAGTTGGGAGGAAGGGCAGTTACCTAATACGTAATTGTTTTGACGTTACCGACAGAATAAGCACCGGCTAACTCTGTGCCAGCAGCCGCGGTAATACAGAGGGTGCAAGCGTTAATCGGAATTACTGGGCGTAAAGCGCGCGTAGGTGGTTCGTTAAGTTGGATGTGAAATCCCCGGGCTCAACCTGGGAACTGCATTCAAAACTGTCGAGCTAGAGTATGGTAGAGGGTGGGTGGAATTTCCTGTGTAGCGGTGAAATGCGTAGATATAGGAAGGAACACCAGTGGCGAAGGCGACCACCTGGACTGATACTGACACTGAGGTGCGAAAGCGTGGGGAGCAAACAGGATTAGATACCCTGGTAGTCCACGCCGTAAACGATGTCAACTAGCCGTTGGGAGCCTTGAGCTCTTAGTGGCGCAGCTAACGCATTAAGTTGACCGCCTGGGGAGTACGGCCGCAAGGTTAAAACTCAAATGAATTGACGGGGGCCCGCACAAGCGGTGGAGCATGTGGTTTAATTCGAAGCAACGCGAAGAACCTTACCAGGCCTTGACATCCAATGAACTTTCCAGAGATGGATTGGTGCCTTCGGGAACATTGAGACAGGTGCTGCATGGCTGTCGTCAGCTCGTGTCGTGAGATGTTGGGTTAAGTCCCGTAACGAGCGCAACCCTTGTCCTTAGTTACCAGCACGTAATGGTGGGCACTCTAAGGAGACTGCCGGTGACAAACCGGAGGAAGGTGGGGATGACGTCAAGTCATCATGGCCCTTACGGCCTGGGCTACACACGTGCTACAATGGTCGGTACAGAGGGTTGCCAAGCCGCGAGGTGGAGCTAATCCCATAAAACCGATCGTAGTCCGGATCGCAGTCTGCAACTCGACTGCGTGAAGTCGGAATCGCTAGTAATCGCGAATCAGAATGTCGCGGTGAATACGTTCCCGGGCCTTGTACACACCGCCCGTCACACCATGGGAGTGGGTTGCACCAGAAGTAG

>FW305-62

TGCAGTCGAGCGGCAGCACGGGTACTTGTACCTGGTGGCGAGCGGCGGACGGGTGAGTAATGCCTAGGAATCTGCCTGGTAGTGGGGGATAACGTTCGGAAACGAACGCTAATACCGCATACGTCCTACGGGAGAAAGCAGGGGACCTTCGGGCCTTGCGCTATCAGATGAGCCTAGGTCGGATTAGCTAGTTGGTGAGGTAATGGCTCACCAAGGCGACGATCCGTAACTGGTCTGAGAGGATGATCAGTCACACTGGAACTGAGACACGGTCCAGACTCCTACGGGAGGCAGCAGTGGGGAATATTGGACAATGGGCGAAAGCCTGATCCAGCCATGCCGCGTGTGTGAAGAAGGTCTTCGGATTGTAAAGCACTTTAAGTTGGGAGGAAGGGCAGTTACCTAATACGTAATTGTTTTGACGTTACCGACAGAATAAGCACCGGCTAACTCTGTGCCAGCAGCCGCGGTAATACAGAGGGTGCAAGCGTTAATCGGAATTACTGGGCGTAAAGCGCGCGTAGGTGGTTCGTTAAGTTGGATGTGAAATCCCCGGGCTCAACCTGGGAACTGCATTCAAAACTGTCGAGCTAGAGTATGGTAGAGGGTGGGTGGAATTTCCTGTGTAGCGGTGAAATGCGTAGATATAGGAAGGAACACCAGTGGCGAAGGCGACCACCTGGACTGATACTGACACTGAGGTGCGAAAGCGTGGGGAGCAAACAGGATTAGATACCCTGGTAGTCCACGCCGTAAACGATGTCAACTAGCCGTTGGGAGCCTTGAGCTCTTAGTGGCGCAGCTAACGCATTAAGTTGACCGCCTGGGGAGTACGGCCGCAAGGTTAAAACTCAAATGAATTGACGGGGGCCCGCACAAGCGGTGGAGCATGTGGTTTAATTCGAAGCAACGCGAAGAACCTTACCAGGCCTTGACATCCAATGAACTTTCCAGAGATGGATTGGTGCCTTCGGGAACATTGAGACAGGTGCTGCATGGCTGTCGTCAGCTCGTGTCGTGAGATGTTGGGTTAAGTCCCGTAACGAGCGCAACCCTTGTCCTTAGTTACCAGCACGTAATGGTGGGCACTCTAAGGAGACTGCCGGATGACAAACCGGAGGAAGGTGGGGATGACGGCTCAAGTCATCATGGCCCTTACGGCCTGGGCTACACACGTGCTACAATGGTCGGTACAGAGGGTTGCCAAGCCGCGAGGTGGAGCTAATCCCATAAAACCGATCGTAGTCCGGATCGCAGTCTGCAACTCGACTGCGTGAAGTCGGAATCGCTAGTAATCGCGAATCAGAATGTCGCGGTGAATACGTTCCCGGGCCTTGTACACACCGCCCGTCACACCATGGGAGTGGGTTGCACCAGAAGTAGCTAGTCTA

>FW305-63

GGGGCAACCTGGCGGCGAGTGGCGAACGGGTGAGTAATATATCGGAACGTACCCTGGAGTGGGGGATAACGTAGCGAAAGTTACGCTAATACCGCATACGATCTAAGGATGAAAGTGGGGGATCGCAAGACCTCATGCTCCTGGAGCGGCCGATATCTGATTAGCTAGTTGGTGGGGTAAAGGCCTACCAAGGCATCGATCAGTAGCTGGTCTGAGAGGACGACCAGCCACACTGGAACTGAGACACGGTCCAGACTCCTACGGGAGGCAGCAGTGGGGAATTTTGGACAATGGGCGCAAGCCTGATCCAGCAATGCCGCGTGAGTGAAGAAGGCCTTCGGGTTGTAAAGCTCTTTTGTCAGGGAAGAAACGGTGGAGGCTAATATCCTTTGCTAATGACGGTACCTGAAGAATAAGCACCGGCTAACTACGTGCCAGCAGCCGCGGTAATACGTAGGGTGCAAGCGTTAATCGGAATTACTGGGCGTAAAGCGTGCGCAGGCGGTTTTGTAAGTCTGTCGTGAAATCCCCGGGCTCAACCTGGGAATTGCGATGGAGACTGCAAGGCTAGAATCTGGCAGAGGGGGGTAGAATTCCACGTGTAGCAGTGAAATGCGTAGATATGTGGAGGAACACCGATGGCGAAGGCAGCCCCCTGGGCTAAGATTGACGCTCATGCACGAAAGCGTGGGGAGCAAACAGGATTAGATACCCTGGTAGTCCACGCCCTAAACGATGTCTACTAGTTGTCGGGTCTTAATTGACTTGGTAACGCAGCTAACGCGTGAAGTAGACCGCCTGGGGAGTACGGTCGCAAGATTAAAACTCAAAGGAATTGACGGGGACCCGCACAAGCGGTGGATGATGTGGATTAATTCGATGCAACGCGAAAAACCTTACCTACCCTTGACATGGCAGGAATCCCGGAGAGATCTGGGAGTGCTCGAAAGAGAACCTGCACACAGGTGCTGCATGGCTGTCGTCAGCTCGTGTCGTGAGATGTTGGGTTAAGTCCCGCAACGAGCGCAACCCTTGTCATTAGTTGCTACGAAAGAGCACTCTAATGAGACTGCCGGTGACAAACCGGAGGAAGGTGGGGATGACGTCAAGTCCTCATGGCCCTTATGGGTAGGGCTTCACACGTCATACAATGGTACATACAGAGGGCCGCCAACCCGCGAGGGGGAGCTAATCCCAGAAAGTGTATCGTAGTCCGGATTGTAGTCTGCAACTCGACTGCATGAAGTTGGAATCGCTAGTAATCGCGGATCAGCATGTCGCGGTGAATACGTTCCCGGGTCTTGTACACACCGCCCGTCACACCATGGGAGCGGGTTTTACCAGAAGTAGGTAG

>FW305-67

ACGGGTACTTGTACCTGGTGGCGAGCGGCGGACGGGTGAGTAATGCCTAGGAATCTGCCTGGTAGTGGGGGATAACGTTCGGAAACGAACGCTAATACCGCATACGTCCTACGGGAGAAAGCAGGGGACCTTCGGGCCTTGCGCTATCAGATGAGCCTAGGTCGGATTAGCTAGTTGGTGAGGTAATGGCTCACCAAGGCGACGATCCGTAACTGGTCTGAGAGGATGATCAGTCACACTGGAACTGAGACACGGTCCAGACTCCTACGGGAGGCAGCAGTGGGGAATATTGGACAATGGGCGAAAGCCTGATCCAGCCATGCCGCGTGTGTGAAGAAGGTCTTCGGATTGTAAAGCACTTTAAGTTGGGAGGAAGGGCAGTTACCTAATACGTAATTGTTTTGACGTTACCGACAGAATAAGCACCGGCTAACTCTGTGCCAGCAGCCGCGGTAATACAGAGGGTGCAAGCGTTAATCGGAATTACTGGGCGTAAAGCGCGCGTAGGTGGTTCGTTAAGTTGGATGTGAAATCCCCGGGCTCAACCTGGGAACTGCATTCAAAACTGTCGAGCTAGAGTATGGTAGAGGGTGGGTGGAATTTCCTGTGTAGCGGTGAAATGCGTAGATATAGGAAGGAACACCAGTGGCGAAGGCGACCACCTGGACTGATACTGACACTGAGGTGCGAAAGCGTGGGGAGCAAACAGGATTAGATACCCTGGTAGTCCACGCCGTAAACGATGTCAACTAGCCGTTGGGAGCCTTGAGCTCTTAGTGGCGCAGCTAACGCATTAAGTTGACCGCCTGGGGAGTACGGCCGCAAGGTTAAAACTCAAATGAATTGACGGGGGCCCGCACAAGCGGTGGAGCATGTGGTTTAATTCGAAGCAACGCGAAGAACCTTACCAGGCCTTGACATCCAATGAACTTTCCAGAGATGGATTGGTGCCTTCGGGAACATTGAGACAGGTGCTGCATGGCTGTCGTCAGCTCGTGTCGTGAGATGTTGGGTTAAGTCCCGTAACGAGCGCAACCCTTGTCCTTAGTTACCAGCACGTAATGGTGGGCACTCTAAGGAGACTGCCGGTGACAAACCGGAGGAAGGTGGGGATGACGTCAAGTCATCATGGCCCTTACCGGCCTGGGCTACACACGTGCTACCAATGGTCGGTACAGAGGGTTGCCAAGCCGCGAGGTGGAGCTAATCCCATAAAACCGATCGTAGTCCGGATCGCAGTCTGCAACTCGACTGCGTGAAGTCGGAATCGCTAGTAATCGCGAATCAGAATGTCGCGGTGAATACGTTCCCGGGCCTTGTACACACCGCCCGTCACACCATGGGAGTGGGTTGCACCAGAAGTAGCTAGT

>FW305-74

CCTTTCGGGGTACACGAGCGGCGAACGGGTGAGTAACACGTGGGTGATCTGCCCTGCACTTCGGGATAAGCCTGGGAAACTGGGTCTAATACCGGATATGACCTCAGGTTGCATGACTTGGGGTGGAAAGATTTATCGGTGCAGGATGGGCCCGCGGCCTATCAGCTTGTTGGTGGGGTAATGGCCTACCAAGGCGACGACGGGTAGCCGACCTGAGAGGGTGACCGGCCACACTGGGACTGAGACACGGCCCAGACTCCTACGGGAGGCAGCAGTGGGGAATATTGCACAATGGGCGAAAGCCTGATGCAGCGACGCCGCGTGAGGGATGACGGCCTTCGGGTTGTAAACCTCTTTCAGCAGGGACGAAGCGCAAGTGACGGTACCTGCAGAAGAAGCACCGGCTAACTACGTGCCAGCAGCCGCGGTAATACGTAGGGTGCAAGCGTTGTCCGGAATTACTGGGCGTAAAGAGTTCGTAGGCGGTTTGTCGCGTCGTTTGTGAAAACCAGCAGCTCAACTGCTGGCTTGCAGGCGATACGGGCAGACTTGAGTACTGCAGGGGAGACTGGAATTCCTGGTGTAGCGGTGAAATGCGCAGATATCAGGAGGAACACCGGTGGCGAAGGCGGGTCTCTGGGCAGTAACTGACGCTGAGGAACGAAAGCGTGGGTAGCGAACAGGATTAGATACCCTGGTAGTCCACGCCGTAAACGGTGGGCGCTAGGTGTGGGTTCCTTCCACGGAATCCGTGCCGTAGCTAACGCATTAAGCGCCCCGCCTGGGGAGTACGGCCGCAAGGCTAAAACTCAAAGGAATTGACGGGGGCCCGCACAAGCGGCGGAGCATGTGGATTAATTCGATGCAACGCGAAGAACCTTACCTGGGTTTGACATATACCGGAAAGCTGCAGAGATGTGGCCCCCCTTGTGGTCGGTATACAGGTGGTGCATGGCTGTCGTCAGCTCGTGTCGTGAGATGTTGGGTTAAGTCCCGCAACGAGCGCAACCCCTATCTTATGTTGCCAGCACGTTATGGTGGGGACTCGTAAGAGACTGCCGGGGTCAACTCGGAGGAAGGTGGGGACGACGTCAAGTCATCATGCCCCTTATGTCCAGGGCTTCACACATGCTACAATGGCCAGTACAGAGGGCTGCGAGACCGTGAGGTGGAGCGAATCCCTTAAAGCTGGTCTCAGTTCGGATCGGGGTCTGCAACTCGACCCCGTGAAGTNGGAGTCGCTAGTAATCGCAGATCAGCAACGCTGCGGTGAATACGTTCCCGGGCCTTGTACACACCGCCCGTCACGTCATGAAAGTCGGTAACACCCGAAGCCGGTGGCTT

>FW305-82A

GCAGTCGAACGGCAGCACGGGCTTCGGCCTGGTGGCGAGTGGCGAACGGGTGAGTAATACATCGGAACGTGCCCTGTAGTGGGGGATAACTAGTCGAAAGATTAGCTAATACCGCATACGACCTGAGGGTGAAAGCGGGGGACCGCAAGGCCTCGCGCTACAGGAGCGGCCGATGTCTGATTAGCTAGTTGGTGGGGTAAAAGCCTACCAAGGCGACGATCAGTAGCTGGTCTGAGAGGACGATCAGCCACACTGGGACTGAGACACGGCCCAGACTCCTACGGGAGGCAGCAGTGGGGAATTTTGGACAATGGGGGCAACCCTGATCCAGCAATGCCGCGTGTGTGAAGAAGGCCTTCGGGTTGTAAAGCACTTTTGTCCGGAAAGAAATGGCTCTGGTTAATACCCGGGGTCGATGACGGTACCGGAAGAATAAGCACCGGCTAACTACGTGCCAGCAGCCGCGGTAATACGTAGGGTGCGAGCGTTAATCGGAATTACTGGGCGTAAAGCGTGCGCAGGCGGTTTTGTAAGACAGGCGTGAAATCCCCGAGCTCAACTTGGGAATGGCGCTTGTGACTGCAAGGCTAGAGTATGTCAGAGGGGGGTAGAATTCCACGTGTAGCAGTGAAATGCGTAGAGATGTGGAGGAATACCGATGGCGAAGGCAGCCCCCTGGGACGTCACTGACGCTCATGCACGAAAGCGTGGGGAGCAAACAGGATTAGATACCCTGGTAGTCCACGCCCTAAACGATGTCAACTAGTTGTTGGGGATTCATTTCTTCAGTAACGTAGCTAACGCGTGAAGTTGACCGCCTGGGGAGTACGGTCGCAAGATTAAAACTCAAAGGAATTGACGGGGACCCGCACAAGCGGTGGATGATGTGGATTAATTCGATGCAACGCGAAAAACCTTACCTACCCTTGACATGCCACTAACGAAGCAGAGATGCATTAGGTGCCCGAAAGGGAAAGTGGACACAGGTGCTGCATGGCTGTCGTCAGCTCGTGTCGTGAGATGTTGGGTTAAGTCCCGCAACGAGCGCAACCCTTGTCTCTANAAGGTTGCTACGAAAGGGCACTCTAGAGAGACTGCCGGTGACAAACCGGAGGAAGGTGGGGATGACGTCAAGTCCTCATGGCCCTTATGGGTAGGGCTTCACACGTCATACAATGGTGCGTACAGAGGGTTGCCAACCCGCGAGGGGGAGCTAATCCCAGAAAACGCATCGTAGTCCGGATCGTAGTCTGCAACTCGACTACGTGAAGCGGAATCGCTAGTAATCGCGGATCAGCATGCCGCGGTGAATACGTTCCCGGGTCTTGTACACACCGCCCGTCACACCATGGGAGTGGGTTTTGCCAGAAGTAGTTAGCCTAACCGCAAGGAGGGCGAT

>FW305-82B

GCAGTCGAACGGCAGCACGGGCTTCGGCCTGGTGGCGAGTGGCGAACGGGTGAGTAATACATCGGAACGTGCCCTGTAGTGGGGGATAACTAGTCGAAAGATTAGCTAATACCGCATACGACCTGAGGGTGAAAGCGGGGGACCGCAAGGCCTCGCGCTACAGGAGCGGCCGATGTCTGATTAGCTAGTTGGTGGGGTAAAAGCCTACCAAGGCGACGATCAGTAGCTGGTCTGAGAGGACGATCAGCCACACTGGGACTGAGACACGGCCCAGACTCCTACGGGAGGCAGCAGTGGGGAATTTTGGACAATGGGGGCAACCCTGATCCAGCAATGCCGCGTGTGTGAAGAAGGCCTTCGGGTTGTAAAGCACTTTTGTCCGGAAAGAAATGGCTCTGGTTAATACCCGGGGTCGATGACGGTACCGGAAGAATAAGCACCGGCTAACTACGTGCCAGCAGCCGCGGTAATACGTAGGGTGCGAGCGTTAATCGGAATTACTGGGCGTAAAGCGTGCGCAGGCGGTTTTGTAAGACAGGCGTGAAATCCCCGAGCTCAACTTGGGAATGGCGCTTGTGACTGCAAGGCTAGAGTATGTCAGAGGGGGGTAGAATTCCACGTGTAGCAGTGAAATGCGTAGAGATGTGGAGGAATACCGATGGCGAAGGCAGCCCCCTGGGACGTCACTGACGCTCATGCACGAAAGCGTGGGGAGCAAACAGGATTAGATACCCTGGTAGTCCACGCCCTAAACGATGTCAACTAGTTGTTGGGGATTCATTTCTTCAGTAACGTAGCTAACGCGTGAAGTTGACCGCCTGGGGAGTACGGTCGCAAGATTAAAACTCAAAGGAATTGACGGGGACCCGCACAAGCGGTGGATGATGTGGATTAATTCGATGCAACGCGAAAAACCTTACCTACCCTTGACATGCCACTAACGAAGCAGAGATGCATTAGGTGCCCGAAAGGGAAAGTGGACACAGGTGCTGCATGGCTGTCGTCAGCTCGTGTCGTGAGATGTTGGGTTAAGTCCCGCAACGAGCGCAACCCTTGTCTCTANAAGGTTGCTACGAAAGGGCACTCTAGAGAGACTGCCGGTGACAAACCGGAGGAAGGTGGGGATGACGTCAAGTCCTCATGGCCCTTATGGGTAGGGCTTCACACGTCATACAATGGTGCGTACAGAGGGTTGCCAACCCGCGAGGGGGAGCTAATCCCAGAAAACGCATCGTAGTCCGGATCGTAGTCTGCAACTCGACTACGTGAAGCGGAATCGCTAGTAATCGCGGATCAGCATGCCGCGGTGAATACGTTCCCGGGTCTTGTACACACCGCCCGTCACACCATGGGAGTGGGTTTTGCCAGAAGTAGTTAGCCTAACCGCAAGGAGGGCGAT

>FW305-82C

GCAGTCGAANGGCAGCACGGGAGCAATCCTGGTGGCGAGTGGCGAACGGGTGAGTAATACATCGGAACGTGCCCTGTCGTGGGGGATAACTAGTCGAAAGATTAGCTAATACCGCATACGACCTGAGGGTGAAAGCGGGGGACCGTAAGGCCTCGCGCGATAGGAGCGGCCGATGTCTGATTAGCTAGTTGGTGGGGTAAAGGCCCACCAAGGCGACGATCAGTAGCTGGTCTGAGAGGACGATCAGCCACACTGGGACTGAGACACGGCCCCAGACTCCTACGGGAGGCAGCAGTGGGGAATTTTGGACAATGGGGGCAACCCTGATCCAGCAATGCCGCGTGTGTGAAGAAGGCCTTCGGGTTGTAAAGCACTTTTGTCCGGAAAGAAATCCCCTTGCTCTTAATACAGCGGGGGGATGACGGTACCGGAAGAATAAGCACCGGCTAACTACGTGCCAGCAGCCGCGGTAATACGTAGGGTGCGAGCGTTAATCGGAATTACTGGGCGTAAAGCGTGCGCAGGCGGTTTTGTAAGACAGGCGTGAAATCCCCGAGCTCAACTTGGGAATGGCGCTTGTGACTGCAAGGCTAGAGTATGTCAGAGGGGGGTAGAATTCCACGTGTAGCAGTGAAATGCGTAGAGATGTGGAGGAATACCGATGGCGAAGGCAGCCCCCTGGGACGTCACTGACGCTCATGCACGAAAGCGTGGGGAGCAAACAGGATTAGATACCCTGGTAGTCCACGCCCTAAACGATGTCAACTAGTTGTTGGGGATTCATTTCTTCAGTAACGTAGCTAACGCGTGAAGTTGACCGCCTGGGGAGTACGGTCGCAAGATTAAAACTCAAAGGAATTGACGGGGACCCGCACAAGCGGTGGATGATGTGGATTAATTCGATGCAACGCGAAAAACCTTACCTACCCTTGACATGCCACTAACGAAGCAGAGATGCATCAGGTGCCCGAAAGGGAAAGTGGACACAGGTGCTGCATGGCTGTCGTCAGCTCGTGTCGTGAGATGTTGGGTTAAGTCCCGCAACGAGCGCAACCCTTGTCTTTAGTTGCTACGCAAGAGCACTCTAGAGAGACTGCCGGTGACAAACCGGAGGAAGGTGGGGATGACGTCAAGTCCTCATGGCCCTTATGGGTAGGGCTTCACACGTCATACAATGGTGCGTACAGAGGGTTGCCAACCCGCGAGGGGGAGCTAATCCCAGAAAACGCATCGTAGTCCGGATCGTAGTCTGCAACTCGACTACGTGAAGCCGGAATCGCTAGTAATCGCGGATCAGCATGCCGCGGTGAATACGTTCCCGGGTCTTGTACACACCGCCCGTCACACCATGGGAGTGGGTTTTGCCAGAAGTAGTTAGCCTAACCGCAAGGA

>FW305-89A

GCGCGGGGCAACCTGGCGGCGAGTGGCGAACGGGTGAGTAATATATCGGAACGTACCCTGGAGTGGGGGATAACGTAGCGAAAGTTACGCTAATACCGCAACGATCTAAGGATGAAAGTGGGGGATCGCAAGACCTCATGCTCCTGGAGCGGCCGATATCTGATTAGCTAGTTGGTGGGGTAAAGGCCTACCAAGGCATCGATCAGTAGCTGGTCTGAGAGGACGACCAGCCACACTGGAACTGAGACACGGTCCAGACTCCTACGGGAGGCAGCAGTGGGGAATTTTGGACAATGGGCGCAAGCCTGATCCAGCAATGCCGCGTGAGTGAAGAAGGCCTTCGGGTTGTAAAGCTCTTTTGTCAGGGAAGAAACGGTGGAGGCTAATATCCTCTGCTAATGACGGTACCTGAAGAATAAGCACCGGCTAACTACGTGCCAGCAGCCGCGGTAATACGTAGGGTGCAAGCGTTAATCGGAATTACTGGGCGTAAAGCGTGCGCAGGCGGTTTTGTAAGTCTGTCGTGAAATCCCCGGGCTCAACCTGGGAATTGCGATGGAGACTGCAAGGCTAGAATCTGGCAGAGGGGGGTAGAATTCCACGTGTAGCAGTGAAATGCGTAGATATGTGGAGGAACACCGATGGCGAAGGCAGCCCCCTGGGCTAAGATTGACGCTCATGCACGAAAGCGTGGGGAGCAAACAGGATTAGATACCCTGGTAGTCCACGCCCTAAACGATGTCTACTAGTTGTCGGGTCTTAATTGACTTGGTAACGCAGCTAACGCGTGAAGTAGACCGCCTGGGGAGTACGGTCGCAAGATTAAAACTCAAAGGAATTGACGGGGACCCGCACAAGCGGTGGATGATGTGGATTAATTCGATGCAACGCGAAAAACCTTACCTACCCTTGACATGGCAGGAATCCCGGAGAGATTTGGGAGTGCTCGAAAGAGAACCTGCACACAGGTGCTGCATGGCTGTCGTCAGCTCGTGTCGTGAGATGTTGGGTTAAGTCCCGCAACGAGCGCAACCCTTGTCATTAGTTGCTACGAAAGAGCACTCTAATGAGACTGCCGGTGACAAACCGGAGGAAGGTGGGGATGACGTCAAGTCCTCATGGCCCTTATGGGTAGGGCTTCACACGTCATACAATGGTACATACAGAGGGCCGCCAACCCGCGAGGGGGAGCTAATCCCAGAAAGTGTATCGTAGTCCGGATTGTAGTCTGCAACTCGACTGCATGAAGTGGAATCGCTAGTAATCGCGGATCAGCATGTCGCGGTGAATACGTTCCCGGGTCTTGTACACACCGCCCGTCACACCATGGGAGCGGGTTTTACCAGAAGTAGGTAGCTTAACC

>FW305-97A

CACGGGAGCAATCCTGGTGGCGAGTGGCGAACGGGTGAGTAATACATCGGAACGTGCCCTGTCGTGGGGGATAACTAGTCGAAAGATTAGCTAATACCGCATACGACCTGAGGGTGAAAGCGGGGGACCGTAAGGCCTCGCGCGATAGGAGCGGCCGATGTCTGATTAGCTAGTTGGTGGGGTAAAGGCCCACCAAGGCGACGATCAGTAGCTGGTCTGAGAGGACGATCAGCCACACTGGGACTGAGACACGGCCCAGACTCCTACGGGAGGCAGCAGTGGGGAATTTTGGACAATGGGGGCAACCCTGATCCAGCAATGCCGCGTGTGTGAAGAAGGCCTTCGGGTTGTAAAGCACTTTTGTCCGGAAAGAAATCCCTTGCTCTTAATACGGCGGGGGGATGACGGTACCGGAAGAATAAGCACCGGCTAACTACGTGCCAGCAGCCGCGGTAATACGTAGGGTGCGAGCGTTAATCGGAATTACTGGGCGTAAAGCGTGCGCAGGCGGTTTTGTAAGACAGGCGTGAAATCCCCGAGCTCAACTTGGGAATGGCGCTTGTGACTGCAAGGCTAGAGTATGTCAGAGGGGGGTAGAATTCCACGTGTAGCAGTGAAATGCGTAGAGATGTGGAGGAATACCGATGGCGAAGGCAGCCCCCTGGGACGTCACTGACGCTCATGCACGAAAGCGTGGGGAGCAAACAGGATTAGATACCCTGGTAGTCCACGCCCTAAACGATGTCAACTAGTTGTTGGGGATTCATTTCTTCAGTAACGTAGCTAACGCGTGAAGTTGACCGCCTGGGGAGTACGGTCGCAAGATTAAAACTCAAAGGAATTGACGGGGACCCGCACAAGCGGTGGATGATGTGGATTAATTCGATGCAACGCGAAAAACCTTACCTACCCTTGACATGCCACTAACGAAGCAGAGATGCATCAGGTGCCCGAAAGGGAAAGTGGACACAGGTGCTGCATGGCTGTCGTCAGCTCGTGTCGTGAGATGTTGGGTTAAGTCCCGCAACGAGCGCAACCCTTGTCTTTAGTTGCTACGCAAGAGCACTCTAGAGAGACTGCCGGTGACAAACCGGAGGAAGGTGGGGATGACGTCAAGTCCTCATGGCCCTTATGGGTAGGGCTTCACACGTCATACAATGGTGCGTACAGAGGGTTGCCAACCCGCGAGGGGGAGCTAATCCCAGAAAACGCATCGTAGTCCGGATCGTAGTCTGCAACTCGACTACGTGAAGCCGGAATCGCTAGTAATCGCGGATCAGCATGCCGCGGTGAATACGTTCCCGGGTCTTGTACACACCGCCCGTCACACCATGGGAGTGGGTTTTGCCAGAAGTAGTTAGCCTAACCGCAAGGAGGGCGA

>FW305-97B

TGCAGTCGAANGGCAGCGCGGGAGCAATCCTGGCGGCGAGTGGCGAACGGGTGAGTAATACATCGGAACGTGCCCAATCGTGGGGGATAACGCAGCGAAAGCTGTGCTAATACCGCANACGATCTACGGATGAAAGCAGGGGATCGCAAGACCTTGCGCGAATGGAGCGGCCGATGGCAGATTAGGTAGTTGGTGAGGTAAAGGCTCACCAAGCCTTCGATCTGTAGCTGGTCTGAGAGGACGACCAGCCACACTGGGACTGAGACACGGCCCAGACTCCTACGGGAGGCAGCAGTGGGGAATTTTGGACAATGGGCGCAAGCCTGATCCAGCCATGCCGCGTGCAGGATGAAGGCCTTCGGGTTGTAAACTGCTTTTGTACGGAACGAAACGGCCTTTTCTAATAAAGAAGGCTAATGACGGTACCGTAAGAATAAGCACCGGCTAACTACGTGCCAGCAGCCGCGGTAATACGTAGGGTGCAAGCGTTAATCGGAATTACTGGGCGTAAAGCGTGCGCAGGCGGTAATGTAAGACAGTTGTGAAATCCCCGGGCTCAACCTGGGAACTGCATCTGTGACTGCATTGCTGGAGTACGGCAGAGGGGGATGGAATTCCGCGTGTAGCAGTGAAATGCGTAGATATGCGGAGGAACACCGATGGCGAAGGCAATCCCCTGGGCCTGTACTGACGCTCATGCACGAAAGCGTGGGGAGCAAACAGGATTAGATACCCTGGTAGTCCACGCCCTAAACGATGTCAACTGGTTGTTGGGAATTCACTTTCTCAGTAACGAAGCTAACGCGTGAAGTTGACCGCCTGGGGAGTACGGCCGCAAGGTTGAAACTCAAAGGAATTGACGGGGACCCGCACAAGCGGTGGATGATGTGGGTTTAATTCGATGCAACGCGAAAAACCTTACCCACCTTTGACATGTACGGAATTCGCCAGAGATGGCTTAGTGCTCGAAAGAGAGCCGTAACACAGGTGCTGCATGGCTTTCGTCAGCTCGTGTCGTGAGATGTTGGGTTAAGTCCCGCAACGAGCGCAACCCTTGTCATTAGTTGCTACATTTAGTTGGGCACTCTAATGAGACTGCCGGTGACAAACCGGAGGAAGGTGGGGATGACGTCAAGTCCTCATGGCCCTTATAGGTGGGGCTACACACGTCATACAATGGCTGGTACAAAGGGTTGCCAACCCGCGAGGGGGAGCTAATCCCATAAAACCAGTCGTAGTCCGGATCGCAGTCTGCAACTCGACTGCGTGAAGTTGGAATCGCTAGTAATCGTGGATCAGAATGTCACGGTGAATACGTTCCCGGGTCTTGTACACACCGCCCGTCACACCATGGGAGCGGGTTCTGCCAGAAGTAGTTAGCTTAACCGCAAGGA

>FW305-98

GCAGTCGAACGGCAGCGCGGGAGCAATCCTGGCGGCGAGTGGCGAACGGGTGAGTAATACATCGGAACGTGCCCAATCGTGGGGGATAACGCAGCGAAAGCTGTGCTAATACCGCATACGATCTACGGATGAAAGCAGGGGATCGCAAGACCTTGCGCGAATGGAGCGGCCGATGGCAGATTAGGTAGTTGGTGAGGTAAAGGCTCACCAAGCCTTCGATCTGTAGCTGGTCTGAGAGGACGACCAGCCACACTGGGACTGAGACACGGCCCAGACTCCTACGGGAGGCAGCAGTGGGGAATTTTGGACAATGGGCGCAAGCCTGATCCAGCCATGCCGCGTGCAGGATGAAGGCCTTCGGGTTGTAAACTGCTTTTGTACGGAACGAAACGGTCTTTTCTAATAAAGAAGGCTAATGACGGTACCGTAAGAATAAGCACCGGCTAACTACGTGCCAGCAGCCGCGGTAATACGTAGGGTGCAAGCGTTAATCGGAATTACTGGGCGTAAAGCGTGCGCAGGCGGTAATGTAAGACAGTTGTGAAATCCCCGGGCTCAACCTGGGAACTGCATCTGTGACTGCATTGCTGGAGTACGGCAGAGGGGGATGGAATTCCGCGTGTAGCAGTGAAATGCGTAGATATGCGGAGGAACACCGATGGCGAAGGCAATCCCCTGGGCCTGTACTGACGCTCATGCACGAAAGCGTGGGGAGCAAACAGGATTAGATACCCTGGTAGTCCACGCCCTAAACGATGTCAACTGGTTGTTGGGAATTCACTTTCTCAGTAACGAAGCTAACGCGTGAAGTTGACCGCCTGGGGAGTACGGCCGCAAGGTTGAAACTCAAAGGAATTGACGGGGACCCGCACAAGCGGTGGATGATGGGGGTTTAATTCGATGCAACGCGAAAAACCTTACCCACCTTTGACATGTACGGAATTCGCCAGAGATGGCTTAAGTGCTCGAAAGAGAACCGTAACACAGGTGCTGCATGGCTGTCGTCAGCTCGTGTCGTGAGATGTTGGGTTAAGTCCCGCAACGAGCGCAACCCTTGTCATTAGTTGCTACATTTAGTTGGGCACTCTAATGAGACTGCCGGTGACAAACCGGAGGAAGGTGGGGATGACGTCAAGTCCTCATGGCCCTTATAGGTGGGGCTACACACGTCATACAATGGCTGGTACAAAGGGTTGCCAACCCGCGAGGGGGAGCTAATCCCATAAAACCAGTCGTAGTCCGGATCGCAGTCTGCAACTCGACTGCGTGAAGTCGGAATCGCTAGTAATCGTGGATCAGAATGTCACGGTGAATACGTTCCCGGGTCTTGTACACACCGCCCGTCACACCATGGGAGCGGGTTCTGCCAGAAGTAGTTAGCTTAACCGCAAGGAGG

>FW305-114

GCAGTCGAACGGCAGCGCGGGGCAACCTGGCGGCGAGTGGCGAACGGGTGAGTAATATATCGGAACATACCCTAGAGTGGGGGATAACGTAGCGAAAGTTACGCTAATACCGCATACGATCTGAGGATGAAAGCGGGGGATCGCAAGACCTCGTGCTCATGGAGTGGCCGATATCTGATTAGCTAGTTGGTAGGGTAAAAGCCTACCAAGGCGACGATCAGTAGCTGGTTTGAGAGAACGACCAGCCACACTGGAACTGAGACACGGTCCAGACTCCTACGGGAGGCAGCAGTGGGGAATTTTGGACAATGGGGGCAACCCTGATCCAGCAATGCCGCGTGAGTGAAGAAGGCCTTCGGGTTGTAAAGCTCTTTTGTCAGGGAAGAAACGGTGAGTTCTAATACAACTTGCTAATGACGGTACCTGAAGAATAAGCACCGGCTAACTACGTGCCAGCAGCCGCGGTAATACGTAGGGTGCAAGCGTTAATCGGAATTACTGGGCGTAAAGCGTGCGCAGGCGGTTTTATAAGTCTGATGTGAAATCCCCGGGCTCAACCTGGGAACTGCATTGGAGACTGTAAGGCTAGAGTGTGTCAGAGGGGGGTAGAATTCCACGTGTAGCAGTGAAATGCGTAGATATGTGGAGGAATACCGATGGCGAAGGCAGCCCCCTGGGATAACACTGACGCTCATGCACGAAAGCGTGGGGAGCAAACAGGATTAGATACCCTGGTAGTCCACGCCCTAAACGATGTCTACTAGTTGTCGGGTCTTAATTGACTTGGTAACGCAGCTAACGCGTGAAGTAGACCGCCTGGGGAGTACGGTCGCAAGATTAAAACTCAAAGGAATTGACGGGGACCCGCACAAGCGGTGGATGATGTGGATTAATTCGATGCAACGCGAAAAACCTTACCTACCCTTGACATGGAAGGAATCCCGAAGAGATTTGGGAGTGCTCGAAAGAGAACCTTTACACAGGTGCTGCATGGCTGTCCGTCAGCTCGTGTCGTGAGATGTTGGGTTAAGTCCCGCAACGAGCGCAACCCTTGTCATTAGTTGCTACGAAAGGGCACTCTAATGAGACTGCCGGTGACAAACCGGAGGAAGGTGGGGATGACGTCAAGTCCTCATGGCCCTTATGGGTAGGGCTTCACACGTCATACAATGGTACATACAGAGGGCCGCCAACCCGCGAGGGGGAGCTAATCCCAGAAAGTGTATCGTAGTCCGGATTGTAGTCTGCAACTCGACTACATGAAGTTGGAATCGCTAGTAATCGCGGATCAGCATGTCGCGGTGAATACGTTCCCGGGTCTTGTACACACCGCCCGTCACACCATGGGAGCGGGTTCTGCCAGAAGTAGTTAGCTTAACCGCAAGGAGGGCGA

>FW305-116

CAGTCGAACGGCAGCGCGGGAGCAATCCTGGCGGCGAGTGGCGAACGGGTGAGTAATACATCGGAACGTGCCCAATCGTGGGGGATAACGCAGCGAAAGCTGTGCTAATACCGCATACGATCTACGGATGAAAGCAGGGGATCGCAAGACCTTGCGCGAATGGAGCGGCCGATGGCAGATTAGGTAGTTGGTGAGGTAAAGGCTCACCAAGCCTTCGATCTGTAGCTGGTCTGAGAGGACGACCAGCCACACTGGGACTGAGACACGGCCCAGACTCCTACGGGAGGCAGCAGTGGGGAATTTTGGACAATGGGCGCAAGCCTGATCCAGCCATGCCGCGTGCAGGATGAAGGCCTTCGGGTTGTAAACTGCTTTTGTACGGAACGAAACGGCCTTTTCTAATAAAGAGGGCTAATGACGGTACCGTAAGAATAAGCACCGGCTAACTACGTGCCAGCAGCCGCGGTAATACGTAGGGTGCAAGCGTTAATCGGAATTACTGGGCGTAAAGCGTGCGCAGGCGGTTATGTAAGACAGTTGTGAAATCCCCGGGCTCAACCTGGGAACTGCATCTGTGACTGCATAGCTAGAGTACGGTAGAGGGGGATGGAATTCCGCGTGTAGCAGTGAAATGCGTAGATATGCGGAGGAACACCGATGGCGAAGGCAATCCCCTGGACCTGTACTGACGCTCATGCACGAAAGCGTGGGGAGCAAACAGGATTAGATACCCTGGTAGTCCACGCCCTAAACGATGTCAACTGGTTGTTGGGAATTCACTTTCTCAGTAACGAAGCTAACGCGTGAAGTTGACCGCCTGGGGGAGTACGGCCGCAAGGTTGAAACTCAAAGGAATTGACGGGGACCCGCACAAGCGGTGGATGATGTGGTTTAATTCGATGCAACGCGAAAAACCTTACCCACCTTTGACATGTACGGAATTCGCCAGAGATGGCTTAGTGCTCGAAAGAGAACCGTAACACAGGTGCTGCATGGCTGTCGTCAGCTCGTGTCGTGAGATGTTGGGTTAAGTCCCGCAACGAGCGCAACCCTTGTCATTAGTTGCTACATTTAGTTGGGCACTCTAATGAGACTGCCGGTGACAAACCGGAGGAAGGTGGGGATGACGTCAAGTCCTCATGGCCCTTATAGGTGGGGCTACACACGTCATACAATGGCTGGTACAAAGGGTTGCCAACCCGCGAGGGGGAGCTAATCCCATAAAACCAGTCGTAGTCCGGATCGCAGTCTGCAACTCGACTGCGTGAAGTCGGAATCGCTAGTAATCGTGGATCAGAATGTCACGGTGAATACGTTCCCGGGTCTTGTACACACCGCCCGTCACACCATGGGAGCGGGTTCTGCCAGAAGTAGTTAGCTTAACCGCAAGGAGGGCGA

>FW305-127

AATGCAGTCGAGCGGANGACGGGAGCTTGCTCCTTGATTCAGCGGCGGACGGGTGAGTAATGCCTAGGAATCTGCCTGGTAGTGGGGGACAACGTTTCGAAAGGAACGCTAATACCGCATACGTCCTACGGGAGAAAGCAGGGGACCTTCGGGCCTTGCGCTATCAGATGAGCCTAGGTCGGATTAGCTAGTAGGTGAGGTAATGGCTCACCTAGGCGACGATCCGTAACTGGTCTGAGAGGATGATCAGTCACACTGGAACTGAGACACGGTCCAGACTCCTACGGGAGGCAGCAGTGGGGAATATTGGACAATGGGCGAAAGCCTGATCCAGCCATGCCGCGTGTGTGAAGAAGGTCTTCGGATTGTAAAGCACTTTAAGTTGGGAGGAAGGGCAGTAAGCTAATACCTTGCTGTTTTGACGTTACCGACAGAATAAGCACCGGCTAACTCTGTGCCAGCAGCCGCGGTAATACAGAGGGTGCAAGCGTTAATCGGAATTACTGGGCGTAAAGCGCGCGTAGGTGGTTCGTTAAGTTGGATGTGAAAGCCCCGGGCTCAACCTGGGAACTGCATCCAAAACTGGCGAGCTAGAGTATGGTAGAGGGTGGTGGAATTTCCTGTGTAGCGGTGAAATGCGTAGATATAGGAAGGAACACCAGTGGCGAAGGCGACCACCTGGACTGATACTGACACTGAGGTGCGAAAGCGTGGGGAGCAAACAGGATTAGATACCCTGGTAGTCCACGCCGTAAACGATGTCAACTAGCCGTTGGAATCCTTGAGATTTTAGTGGCGCAGCTAACGCATTAAGTTGACCGCCTGGGGAGTACGGCCGCAAGGTTAAAACTCAAATGAATTGACGGGGGCCCGCACAAGCGGTGGAGCATGTGGTTTAATTCGAAGCAACGCGAAGAACCTTACCAGGCCTTGACATGCAGAGAACTTTCCAGAGATGGATTGGTGCCTTCGGGAACTCTGACACAGGTGCTGCATGGCTGTCGTCAGCTCGTGTCGTGAGATGTTGGGTTAAGTCCCGTAACGAGCGCAACCCTTGTCCTTAGTTACCAGCACGTTATGGTGGGCACTCTAAGGAGACTGCCGGTGACAAACCGGAGGAAGGTGGGGATGACGTCAAGTCATCATGGCCCTTACGGCCTGGGCTACACACGTGCTACAATGGTCGGTACAGAGGGTTGCCAAGCCGCGAGGTGGAGCTAATCTCACAAAACCGATCGTAGTCCGGATCGCAGTCTGCAACTCGACTGCGTGAAGTTGGAATCGCTAGTAATCGCAAATCAGAATGTTGCGGTGAATACGTTCCCGGGCCTTGTACACACCGCCCGTCACACCATGGGAGTGGGNTGCACCAGAAGTAGCTAGTCTNACCTTCNGGAGGACGG

>FW305-128

TGCAGTCGAACGGCAGCACGGAGCTTGCTCTGGTGGCGAGTGGCGAACGGGTGAGTAATATATCGGAACGTACCCTGGAGTGGGGGATAACGTAGCGAAAGTTACGCTAATACCGCATACGATCTACGGATGAAAGTGGGGGATCGCAAGACCTCATGCTCGTGGAGCGGCCGATATCTGATTAGCTAGTTGGTAGGGTAAAAGCCTACCAAGGCATCGATCAGTAGCTGGTCTGAGAGGACGACCAGCCACACTGGAACTGAGACACGGTCCAGACTCCTACGGGAGGCAGCAGTGGGGAATTTTGGACAATGGGCGAAAGCCTGATCCAGCAATGCCGCGTGAGTGAAGAAGGCCTTCGGGTTGTAAAGCTCTTTTGTCAGGGAAGAAACGGTGAGAGCTAATATCTCTTGCTAATGACGGTACCTGAAGAATAAGCACCGGCTAACTACGTGCCAGCAGCCGCGGTAATACGTAGGGTGCAAGCGTTAATCGGAATTACTGGGCGTAAAGCGTGCGCAGGCGGTTTTGTAAGTCTGATGTGAAATCCCCGGGCTCAACCTGGGAATTGCATTGGAGACTGCAAGGCTAGAATCTGGCAGAGGGGGGTAGAATTCCACGTGTAGCAGTGAAATGCGTAGATATGTGGAGGAACACCGATGGCGAAGGCAGCCCCCTGGGTCAAGATTGACGCTCATGCACGAAAGCGTGGGGAGCAAACAGGATTAGATACCCTGGTAGTCCACGCCCTAAACGATGTCTACTAGTTGTCGGGTCTTAATTGACTTGGTAACGCAGCTAACGCGTGAAGTAGACCGCCTGGGGAGTACGGTCGCAAGATTAAAACTCAAAGGAATTGACGGGGACCCGCACAAGCGGTGGATGATGTGGATTAATTCGATGCAACGCGAAAAACCTTACCTACCCTTGACATGGCTGGAATCCCCGAGAGATTGGGGAGTGCTCGAAAGAGAACCAGTACACAGGTGCTGCATGGCTGTCGTCAGCTCGTGTCGTGAGATGTTGGGTTAAGTCCCGCAACGAGCGCAACCCTTGTCATTAGTTGCTACGAAAGGGCACTCTAATGAGACTGCCGGTGACAAACCGGAGGAAGGTGGGGATGACGTCAAGTCCTCATGGCCCTTATGGGTAGGGCTTCACACGTCATACAATGGTACATACAGAGCGCCGCCAACCCGCGAGGGGGAGCTAATCGCAGAAAGTGTATCGTAGTCCGGATTGTAGTCTGCAACTCGACTGCATGAAGTTGGAATCGCTAGTAATCGCGGATCAGCATGTCGCGGTGAATACGTTCCCGGGTCTTGTACACACCGCCCGTCACACCATGGGAGCGGGTTTTACCAGAAGTAGGTAGCTTAACC

>FW305-130

GCAGTCGAGCGGTAAGGCCTTTCGGGGTACACGAGCGGCGAACGGGTGAGTAACACGTGGGTGATCTGCCCTGCACTTCGGGATAAGCCTGGGAAACTGGGTCTAATACCGGATATGACCTCAGGTTGCATGACTTGGGGTGGAAAGATTTATCGGTGCAGGATGGGCCCGCGGCCTATCAGCTTGTTGGTGGGGTAATGGCCTACCAAGGCGACGACGGGTAGCCGACCTGAGAGGGTGACCGGCCACACTGGGACTGAGACACGGCCCAGACTCCTACGGGAGGCAGCAGTGGGGAATATTGCACAATGGGCGAAAGCCTGATGCAGCGACGCCGCGTGAGGGATGACGGCCTTCGGGTTGTAAACCTCTTTCAGCAGGGACGAAGCGCAAGTGACGGTACCTGCAGAAGAAGCACCGGCTAACTACGTGCCAGCAGCCGCGGTAATACGTAGGGTGCAAGCGTTGTCCGGAATTACTGGGCGTAAAGAGTTCGTAGGCGGTTTGTCGCGTCGTTTGTGAAAACCAGCAGCTCAACTGCTGGCTTGCAGGCGATACGGGCAGACTTGAGTACTGCAGGGGAGACTGGAATTCCTGGTGTAGCGGTGAAATGCGCAGATATCAGGAGGAACACCGGTGGCGAAGGCGGGTCTCTGGGCAGTAACTGACGCTGAGGAACGAAAGCGTGGGTAGCGAACAGGATTAGATACCCTGGTAGTCCACGCCGTAAACGGTGGGCGCTAGGTGTGGGTTCCTTCCACGGAATCCGTGCCGTAGCTAACGCATTAAGCGCCCCGCCTGGGGAGTACGGCCGCAAGGCTAAAACTCAAAGGAATTGACGGGGGCCCGCACAAGCGGCGGAGCATGTGGATTAATTCGATGCAACGCGAAGAACCTTACCTGGGGTTTGACATATACCGGAAAGCTGCAGAGATGTGGCCCCCCTTGTGGTCGGTATACAGGTGGTGCATGGCTGTCGTCAGCTCGTGTCGTGAGATGTTGGGTTAAGTCCCGCAACGAGCGCAACCCCTATCTTATGTTGCCAGCACGTTATGGTGGGGACTCGTAAGAGACTGCCGGGGTCAACTCGGAGGAAGGTGGGGACGACGTCAAGTCATCATGCCCCTTATGTCCAGGGCTTCACACATGCTACAATGGCCAGTACAGAGGGCTGCGAGACCGTGAGGTGGAGCGAATCCCTTAAAGCTGGTCTCAGTTCGGATCGGGGTCTGCAACTCGACCCCGTGAAGTNGGAGTCGCTAGTAATCGCAGATCAGCAACGCTGCGGTGAATACGTTCCCGGGCCTTGTACACACCGCCCGTCACGTCATGAAAGTCGGTAACACCCGAAGCCGGTGGCT

>FW305-141

CGGTAACGCGGGGCAACCTGGCGACGAGTGGCGAACGGGTGAGTAAAGCATCGGAACGTGCCCAGTAGTGGGGGATAGCCCGGCGAAAGCCGGATTAATACCGCATACGACCTACGGGTGAAAGGGGGGGATCGCAAGACCTCTCGCTATTGGAGCGGCCGATGTCAGATTAGCTAGTTGGTGGGGTAAAGGCCTACCAAGGCGACGATCTGTAGCTGGTCTGAGAGGACGACCAGCCACACTGGGACTGAGACACGGCCCAGACTCCTACGGGAGGCAGCAGTGGGGAATTTTGGACAATGGACGCAAGTCTGATCCAGCCATGCCGCGTGCGGGAAGAAGGCCTTCGGGTTGTAAACCGCTTTTGTCAGGGAAGAAATCTTCTGGGTTAACACCTCGGGAGGATGACGGTACCTGAAGAATAAGCACCGGCTAACTACGTGCCAGCAGCCGCGGTAATACGTAGGGTGCGAGCGTTAATCGGAATTACTGGGCGTAAAGCGTGCGCAGGCGGTTATATAAGACAGAGGTGAAATCCCCGGGCTCAACCTGGGAACTGCCTTTGTGACTGTATAGCTTGAGTGCGGCAGAGGGGGATGGAATTCCGCGTGTAGCAGTGAAATGCGTAGATATGCGGAGGAACACCGATGGCGAAGGCAATCCCCTGGGCCTGCACTGACGCTCATGCACGAAAGCGTGGGGAGCAAACAGGATTAGATACCCTGGTAGTCCACGCCCTAAACGATGTCAACTGGTTGTTGGGAAGGTTCCTTCTCAGTAACGAAGCTAACGCGTGAAGTTGACCGCCTGGGGAGTACGGCCGCAAGGTTGAAACTCAAAGGAATTGACGGGGACCCGCACAAGCGGTGGATGATGTGGTTTAATTCGATGCAACGCGAAAAACCTTACCTACCCTTGACATGTCTGGAATCCTGAAGAGATTTGGGAGTGCTCGAAAGAGAGCCAGAACACAGGTGCTGCATGGCCGTCGTCAGCTCGTGTCGTGAGATGTTGGGTTAAGTCCCGCAACGAGCGCAACCCTTGTCATTAGTTGCTACGAAAGGGCACTCTAATGAGACTGCCGGTGACAAACCGGAGGAAGGTGGGGATGACGTCAGGTCCTCATGGCCCTTATGGGTAGGGCTACACACGTCATACAATGGCCGGTACAGAGGGCTGCCAACCCGCGAGGGGGAGCCAATCCCAGAAAACCGGTCGTAGTCCGGATCGCAGTCTGCAACTCGACTGCGTGAAGTCGGAATCGCTAGTAATCGCGGATCAGCTTGCCGCGGTGAATACGTTCCCGGGTCTTGTACACACCGCCCGTCACACCATGGGAGCGGGTTCTGCCAGAAGTAGTTAGCCTAACCGCAAGGAGGGCGA

>FW305-A1

GGCCCTTCGGGGTACTCGAGTGGCGAACGGGTGAGTAACACGTGGGTGATCTGCCCTGCACTTTGGGATAAGCCTGGGAAACTGGGTCTAATACCGAATATGACCACGCTCTTCATGGGGTGTGGTGGAAAGCTTTTGCGGTGTGGGATGGGCCCGCGGCCTATCAGCTTGTTGGTGGGGTAATGGCCTACCAAGGCGACGACGGGTAGCCGGCCTGAGAGGGTGACCGGCCACACTGGGACTGAGATACGGCCCAGACTCCTACGGGAGGCAGCAGTGGGGAATATTGCACAATGGGCGCAAGCCTGATGCAGCGACGCCGCGTGAGGGATGACGGCCTTCGGGTTGTAAACCTCTTTCACCAGGGACGAAGCGCAAGTGACGGTACCTGGAGAAGAAGCACCGGCCAACTACGTGCCAGCAGCCGCGGTAATACGTAGGGTGCGAGCGTTGTCCGGAATTACTGGGCGTAAAGAGCTCGTAGGTGGTTTGTCGCGTTGTTCGTGAAAACTCACAGCTTAACTGTGGGCGTGCGGGCGATACGGGCAGACTAGAGTACTGCAGGGGAGACTGGAATTCCTGGTGTAGCGGTGGAATGCGCAGATATCAGGAGGAACACCGGTGGCGAAGGCGGGTCTCTGGGCAGTAACTGACGCTGAGGAGCGAAAGCGTGGGGAGCGAACAGGATTAGATACCCTGGTAGTCCACGCCGTAAACGGTGGGTACTAGGTGTGGGTTCCTTCCTTGGGATCCGTGCCGTAGCTAACGCATTAAGTACCCCGCCTGGGGAGTACGGCCGCAAGGCTAAAACTCAAAGGAATTGACGGGGGGCCCGCACAAGCGGCGGAGCATGTGGATTAATTCGATGCAACGCGAAGAACCTTACCTGGGTTTGACATGCACAGGACGCTGGTAGAGATATCAGTTCCCTTGTGGCCTGTGTGCAGGTGGTGCATGGCTGTCGTCAGCTCGTGTCGTGAGATGTTGGGTTAAGTCCCGCAACGAGCGCAACCCCTATCTTATGTTGCCAGCGCGTTATGGCGGGGACTCGTAAGAGACTGCCGGGGTCAACTCGGAGGAAGGTGGGGATGACGTCAAGTCATCATGCCCCTTATGTCCAGGGCTTCACACATGCTACAATGGCCGGTACAAAGGGCTGCGAATCCGCGAGGTGGAGCGAATCCCTTGAAAGCCGGTCTCAGTTCGGATCGGGGTCTGCAACTCGACCCCGTGAAGTGGAGTCGCTAGTAATCGCAGATCAGCAACGCTGCGGTGAATACGTTCCCGGGCCTTGTACTACACCGCCCGTCACGTCATG

>FW305-E1

GCACTTTTGGGATAAGCCTGGGAAACTGGGTCTAATACCGAATATGACCACGCTCTTTCATGGGGTGTGGTGGAAAGCTTTTGCGGTGTGGGATGGGCCCGCGGCCTATCAGCTTGTTGGTGGGGTAATGGCCTACCAAGGCGACGACGGGTAGCCGGCCTGAGAGGGTGACCGGCCACACTGGGACTGAGATACGGCCCAGACTCCTACGGGAGGCAGCAGTGGGGAATATTGCACAATGGGCGCAAGCCTGATGCAGCGACGCCGCGTGAGGGATGACGGCCTTCGGGTTGTAAACCTCTTTCACCAGGGACGAAGCGCAAGTGACGGTACCTGGAGAAGAAGCACCGGCCAACTACGTGCCAGCAGCCGCGGTAATACGTAGGGTGCGAGCGTTGTCCGGAATTACTGGGCGTAAAGAGCTCGTAGGTGGTTTGTCGCGTTGTTCGTGAAAACTCACAGCTTAACTGTGGGCGTGCGGGCGATACGGGCAGACTAGAGTACTGCAGGGGAGACTGGAATTCCTGGTGTAGCGGTGGAATGCGCAGATATCAGGAGGAACACCGGTGGCGAAGGCGGGTCTCTGGGCAGTAACTGACGCTGAGGAGCGAAAGCGTGGGGAGCGAACAGGATTAGATACCCTGGTAGTCCACGCCGTAAACGGTGGGTACTAGGTGTGGGTTCCTTCCTTGGGATCCGTGCCGTAGCTAACGCATTAAGTACCCCGCCTGGGGAGTACGGCCGCAAGGCTAAAACTCAAAGGAATTGACGGGGGCCCGCACAAGCGGCGGAGCATGTGGATTAATTCGATGCAACGCGAAGAACCTTACCTGGGTTTGACATGCACAGGACGCTGGTAGAGATATCAGTTCCCTTGTGGCCTGTGTGCAGGTGGTGCATGGCTGTCGTCAGCTCGTGTCGTGAGATGTTGGGTTAAGTCCCGCAACGAGCGCAACCCCTATCTTATGTTGCCAGCGCGTTATGGCGGGGACTCGTAAGAGACTGCCGGGGTCAACTCGGAGGAAGGTGGGGATGACGTCAAGTCATCATGCCCCTTATGTCCAGGGCTTCACACATGCTACAATGGCCGGTACAAAGGGCTGCGAATCCGCGAGGTGGAGCGAATCCCTTGAAAGCCGGTCTCAGTTCGGATCGGGGTCTGCAACTCGACCCCGTGAAGTGGAGTCGCTAGTAATCGCAGATCAGCAACGCTGCGGTGAAATACGTTCCCGGGCCTTGTACAACACCGCCCGTC

>FW305-E2

GACTTGCTCCTTGATCAGCGGCGGACGGGTGAGTAATGCCTAGGAATCTGCCTGGTAGTGGGGGACAACGTTTCGAAAGGAACGCTAATACCGCATACGTCCTACGGGAGAAAGCAGGGGACCTTCGGGCCTTGCGCTATCAGATGAGCCTAGGTCGGATTAGCTAGTTGGTGGGGTAATGGCTCACCAAGGCGACGATCCGTAACTGGTCTGAGAGGATGATCAGTCACACTGGAACTGAGACACGGTCCAGACTCCTACGGGAGGCAGCAGTGGGGAATATTGGACAATGGGCGAAAGCCTGATCCAGCCATGCCGCGTGTGTGAAGAAGGTCTTCGGATTGTAAAGCACTTTAAGTTGGGAGGAAGGGCAGTAAGTNAATACCTTGCTGTTTTGACGTTACCGACAGAATAAGCACCGGCTAACTCTGTGCCAGCAGCCGCGGTAATACAGAGGGTGCAAGCGTTAATCGGAATTACTGGGCGTAAAGCGCGCGTAGGTGGTTTGTTAAGTTGGATGTGAAAGCCCCGGGCTCAACCTGGGAACTGCATCCAAAACTGGCAAGCTAGAGTACGGTAGAGGGTGGTGGAATTTCCTGTGTAGCGGTGAAATGCGTAGATATAGGAAGGAACACCAGTGGCGAAGGCGACCACCTGGACTGATACTGACACTGAGGTGCGAAAGCGTGGGGAGCAAACAGGATTAGATACCCTGGTAGTCCACGCCGTAAACGATGTCAACTAGCCGTTGGAATCCTTGAGATTTTAGTGGCGCAGCTAACGCATTAAGTTGACCGCCTGGGGAGTACGGCCGCAAGGTTAAAACTCAAATGAATTGACGGGGGCCCGCACAAGCGGTGGAGCATGTGGTTTAATTCGAAGCAACGCGAAGAACCTTACCAGGCCTTGACATGCAGAGAACTTTCCAGAGATGGATTGGTGCCTTCGGGAACTCTGACACAGGTGCTGCATGGCTGTCGTCAGCTCGTGTCGTGAGATGTTGGGTTAAGTCCCGTAACGAGCGCAACCCTTGTCCTTAGTTACCAGCACGTTATGGTGGGCACTCTAAGGAGACTGCCGGTGACAAACCGGAGGAAGGTGGGGATGACGTCAAGTCATCATGGCCCTTACGGCCTGGGCTACACACGTGCTACAATGGTCGGTACAGAGGGTTGCCAAGCCGCGAGGTGGAGCTAATCTCACAAAACCGATCGTAGTCCGGATCGCAGTCTGCAACTCGACTGCGTGAAGTCGGAATCGCTAGTAATCGCGAATCAGAATGTCGCGGTGTAATACGTTCCCGGGCCTTGTACACACCGCCCGTCACACC

>FW305-E3

GGCAGCGCGGGAGCAATCCTGGCGGCGAGTGGCGAACGGGTGAGTAATACATCGGAACGTGCCCAATCGTGGGGGATAACGCAGCGAAAGCTGTGCTAATACCGCATACGATCTACGGATGAAAGCAGGGGATCGCAAGACCTTGCGCGAATGGAGCGGCCGATGGCAGATTAGGTAGTTGGTGAGGTAAAGGCTCACCAAGCCTTCGATCTGTAGCTGGTCTGAGAGGACGACCAGCCACACTGGGACTGAGACACGGCCCAGACTCCTACGGGAGGCAGCAGTGGGGAATTTTGGACAATGGGCGCAAGCCTGATCCAGCCATGCCGCGTGCAGGATGAAGGCCTTCGGGTTGTAAACTGCTTTTGTACGGAACGAAACGGCCTTTTCTAATAAAGAGGGCTAATGACGGTACCGTAAGAATAAGCACCGGCTAACTACGTGCCAGCAGCCGCGGTAATACGTAGGGTGCAAGCGTTAATCGGAATTACTGGGCGTAAAGCGTGCGCAGGCGGTTATGTAAGACAGTTGTGAAATCCCCGGGCTCAACCTGGGAACTGCATCTGTGACTGCATAGCTAGAGTACGGTAGAGGGGGGATGGAATTCCGCGTGTAGCAGTGAAATGCGTAGATATGCGGAGGAACACCGATGGCGAAGGCAATCCCCTGGACCTGTACTGACGCTCATGCACGAAAGCGTGGGGAGCAAACAGGATTAGATACCCTGGTAGTCCACGCCCTAAACGATGTCAACTGGTTGTTGGGAATTCACTTTCTCAGTAACGAAGCTAACGCGTGAAGTTGACCGCCTGGGGAGTACGGCCGCAAGGTTGAAACTCAAAGGAATTGACGGGGACCCGCACAAGCGGTGGATGATGTGGTTTAATTCGATGCAACGCGAAAAACCTTACCCACCTTTGACATGTACGGAATTCGCCAGAGATGGCTTAGTGCTCGAAAGAGAACCGTAACACAGGTGCTGCATGGCTGTCGTCAGCTCGTGTCGTGAGATGTTGGGTTAAGTCCCGCAACGAGCGCAACCCTTGTCATTAGTTGCTACATTTAGTTGGGCACTCTAATGAGACTGCCGGTGACAAACCGGAGGAAGGTGGGGATGACGTCAAGTCCTCATGGCCCTTATAGGTGGGGCTACACACGTCATACAATGGCTGGTACAAAGGGTTGCCAACCCGCGAGGGGGAGCTAATCCCATAAAACCAGTCGTAGTCCGGATCGCAGTCTGCAACTCGACTGCGTGAAGTCGGAATCGCTAGTAATCGTGGATCAGAATGTCACGGTGAATACGTTCCCGGGTCTTGTACACACCGCCCGTCACACCATGGGAGCGGGTTCTGCCAGAAGTAGTTAGCTTAACCGCAAGGA

>FW305-F1

GGCAGCGCGGGAGCAATCCCTGGCGGCGAGTGGCGAACGGGTGAGTAATACATCGGAACGTGCCCAATCGTGGGGGATAACGCAGCGAAAGCTGTGCTAATACCGCATACGATCTACGGATGAAAGCAGGGGATCGCAAGACCTTGCGCGAATGGAGCGGCCGATGGCAGATTAGGTAGTTGGTGAGGTAAAGGCTCACCAAGCCTTCGATCTGTAGCTGGTCTGAGAGGACGACCAGCCACACTGGGACTGAGACACGGCCCAGACTCCTACGGGAGGCAGCAGTGGGGAATTTTGGACAATGGGCGCAAGCCTGATCCAGCCATGCCGCGTGCAGGATGAAGGCCTTCGGGTTGTAAACTGCTTTTGTACGGAACGAAACGGCCTTTTCTAATAAAGAGGGCTAATGACGGTACCGTAAGAATAAGCACCGGCTAACTACGTGCCAGCAGCCGCGGTAATACGTAGGGTGCAAGCGTTAATCGGAATTACTGGGCGTAAAGCGTGCGCAGGCGGTTATGTAAGACAGTTGTGAAATCCCCGGGCTCAACCTGGGAACTGCATCTGTGACTGCATAGCTAGAGTACGGTAAAGGGGGAATGGAATTCCGCGTGTAGCATTGAAATGCGTAGATATGCGGAGGAACACCGATGGCGAAGGCAATCCCCTGGACCTGTACTGACGCTCATGCACGAAAGCGTGGGGAGCAAACAGGATTAGATACCCTGGTAGTCCACGCCCTAAACGATGTCAACTGGTTGTTGGGAATTCACTTTCTCAGTAACGAAGCTAACGCGTGAAGTTGACCGCCTGGGGAGTACGGCCGCAAGGTTGAAACTCAAAGGAATTGACGGGGACCCGCACAAGCGGTGGATGATGTGGTTTAATTCGATGCAACGCGAAAAACCTTACCCACCTTTGACATGTACGGAATTCGCCAGAGATGGCTTAGTGCTCGAAAGAGAACCGTAACACAGGTGCTGCATGGCTGTCGTCAGCTCGTGTCGTGAGATGTTGGGTTAAGTCCCGCAACGAGCGCAACCCTTGTCATTAGTTGCTACATTTAGTTGGGCACTCTAATGAGACTGCCGGTGACAAACCGGAGGAAGGTGGGGATGACGTCAAGTCCTCATGGCCCTTATAGGTGGGGCTACACACGTCATACAATGGCTGGTACAAAGGGTTGCCAACCCGCGAGGGGGAGCTAATCCCATAAAACCAGTCGTAGTCCGGATCGCAGTCTGCGAACTCGACTGCGTGAAAGTCGGAATCGCTAGTAATCGTGGATCAGAAATGTCACGGTGTAATACGTTCCCGGGTTCTTGTACACACCGCCCGTCACACTCATGGAAGACGGGTTCTGCCAGAAAGTAGTTAGCTAACCGCAAGGAGGGCGATATCTT

>FW305-F2

CGGCAGCGCGGGAGCAAATCCTGGCGGCGAGTGGCGAACGGGTGAGTAATACATCGGAACGTGCCCAATCGTGGGGGATAACGCAGCGAAAGCTGTGCTAATACCGCATACGATCTACGGATGAAAGCAGGGGATCGCAAGACCTTGCGCGAATGGAGCGGCCGATGGCAGATTAGGTAGTTGGTGAGGTAAAGGCTCACCAAGCCTTCGATCTGTAGCTGGTCTGAGAGGACGACCAGCCACACTGGGACTGAGACACGGCCCAGACTCCTACGGGAGGCAGCAGTGGGGAATTTTGGACAATGGGCGCAAGCCTGATCCAAGCCATGCCGCGTGCAGGATGAAGGCCTTCGGGTTGTAAACTGCTTTTGTACGGAACGAAACGGCCTTTTCTAATAAAGAAGGCTAATGACGGTACCGTAAGAATAAGCACCGGCTAACTACGTGCCAGCAGCCGCGGTAATACGTAGGGTGCAAGCGTTAATCGGAATTACTGGGCGTAAAGCGTGCGCAGGCGGTAATGTAAGACAGTTGTGAAATCCCCGGGCTCAACCTGGGAACTGCATCTGTGACTGCATTGCTGGAGTACGGCAGAGGGGGATGGAATTCCGCGTGTAGCAGTGAAATGCGTAGATATGCGGAGGAACACCGATGGCGAAGGCAATCCCCTGGGCCTGTACTGACGCTCATGCACGAAAGCGTGGGGAGCAAACAGGATTAGATACCCTGGTAGTCCACGCCCTAAACGATGTCAACTGGTTGTTGGGAATTCACTTTCTCAGTAACGAAGCTAACGCGTGAAGTTGACCGCCTGGGGAGTACGGCCGCAAGGTTGAAACTCAAAGGAATTGACGGGGACCCGCACAAGCGGTGGATGATGTGGTTTAATTCGATGCAACGCGAAAAACCTTACCCACCTTTGACATGTACGGAATTCGCCAGAGATGGCTTAGTGCTCGAAAGAGAGCCGTAACACAGGTGCTGCATGGCTGTCGTCAGCTCGTGTCGTGAGATGTTGGGTTAAGTCCCGCAACGAGGCGCAACCCTTGTCATTAGTTGCTACATTTAGTTGGGCACTCTAATGAGACTGCCGGTGACAAACCGGAGGAAGGTGGGGATGACGTCAAGTCCTCATGGCCCTTATAGGTGGGGCTACACACGTCATACAATGGCTGGTACAAAGGGTTGCCAACCCGCGAGGGGGAGCTAATCCCATAAAACCAGTCGTAGTCCGGATCGCAGTCTGCAACTCGACTGCGTGAAGTCGGAATCGCTAGTAATCGTGGATCAGAATGTCACGGTGAATACGTTCCCGGGTCTTGTACACACCGCCCGTCACACCATGGGAGCGGGTTCTGCCAGAAGTAGTTAGCTTAACCGCAAGGAGGGCGATA

>FW305-F3

ACGGCAGCGCGGGAGCAATCCTGGCGGCGAGTGGCGAACGGGTGAGTAATACATCGGAACGTGCCCAATCGTGGGGGATAACGCAGCGAAAGCTGTGCTAATACCGCATACGATCTACGGATGAAAGCAGGGGATCGCAAGACCTTGCGCGAATGGAGCGGCCGATGGCAGATTAGGTAGTTGGTGAGGTAAAGGCTCACCAAGCCTTCGATCTGTAGCTGGTCTGAGAGGACGACCAGCCACACTGGGACTGAGACACGGCCCAGACTCCTACGGGAGGCAGCAGTGGGGAATTTTGGACAATGGGCGCAAGCCTGATCCAGCCATGCCGCGTGCAGGATGAAGGCCTTCGGGTTGTAAACTGCTTTTGTACGGAACGAAACGGCCTTTTCTAATAAAGAAGGCTAATGACGGTACCGTAAGAATAAGCACCGGCTAACTACGTGCCAGCAGCCGCGGTAATACGTAGGGTGCAAGCGTTAATCGGAATTACTGGGCGTAAAGCGTGCGCAGGCGGTAATGTAAGACAGTTGTGAAATCCCCGGGCTCAACCTGGGAACTGCATCTGTGACTGCATTGCTGGAGTACGGCAGAGGGGGATGGAATTCCGCGTGTAGCAGTGAAATGCGTAGATATGCGGAGGAACACCGATGGCGAAGGCAATCCCCTGGGCCTGTACTGACGCTCATGCACGAAAGCGTGGGAGCAAACAGGATTAGATACCCTGGTAGTCCACGCCCTAAACGATGTCAACTGGTTGTTGGGAATTCACTTTCTCAGTAACGAAGCTAACGCGTGAAGTTGACCGCCTGGGGAGTACGGCCGCAAGGTTGAAACTCAAAGGAATTTGACGGGGACCCGCACAAGCGGTGGATGATGTGGTTTAATTCGATGCAACGCGAAAAACCTTTACCCACCTTTGACATGTACGGAATTCGCCAGAGATGGCTTAGTGCTCGAAAGAGAACCGTAACACAGGTGCTGCATGGCTGTCGTCAGCTCGTGTCGTGAGATGTTGGGTTAAGTCCCGCAACGAGCGCAACCCTTGTCATTAGTTGCTACATTTAGTTGGGCACTCTAATGAGACTGCCGGTGACAAACCGGAGGAAGGTGGGGATGACGTCAAGTCCTCATGGCCCTTATAGGTGGGGCTACACACGTCATACAATGGCTGGTACAAAGGGTTGCCAACCCGCGAGGGGGAGCTAATCCCATAAAACCAGTCGTAGTCCGGATCGCAGTCTGC

>FW305-F4

GGCGAACGGGTGAGTAATACATCGGAACGTGCCCAATCGTGGGGGATAACGCAGCGAAAGCTGTGCTAATACCGCATACGATCTACGGATGAAAGCAGGGGATCGCAAGACCTTGCGCGAATGGAGCGGCCGATGGCAGATTAGGTAGTTGGTGAGGTAAAGGCTCACCAAGCCTTCGATCTGTAGCTGGTCTGAGAGGACGACCAGCCACACTGGGACTGAGACACGGCCCAGACTCCTACGGGAGGCAGCAGTGGGGAATTTTGGACAATGGGCGCAAGCCTGATCCAGCCATGCCGCGTGCAGGATGAAGGCCTTCGGGTTGTAAACTGCTTTTGTACGGAACGAAACGGCCTTTTCTAATAAAGAAGGCTAATGACGGTACCGTAAGAATAAGCACCGGCTNAACTACGTGCCAGCAGCCGCGGTAATACGTAGGGTGCAAGCGTTAATCGGAATTACTGGGCGTAAAGCGTGCGCAGGCGGTAATGTAAGACAGTTGTGAAATCCCCGGGGCTCAACCTGGGAACTGCATCTGTGACTGCATTGCTGGAGTACGGCAGAGGGGGGATGGAATTCCGCGTGTAGCAGTGAAATGCGTAGATATGCGGGAGGAACACCGATGGGCGAAGGCAATTCCCCTGGGCCTGTACTGACGGCTCATGCACGAAAAGCGTGGGGGAGCAAAACAGGATTAGATACCCTTGGTAGTCCCACGCCCTAAAACGATGTCAAACTGGGTTGTTGGGGAATTCACTTTTCTCAGTAACGAAAGCTAACGGCGTGAAAGTTGACCCGCCTGGGGGAGTACCGGCCCGCAAAGGTTGAAAACTCAAAAGGGAATTTGACGGGGGACCCCGCACAAAGCGGGTGGGATGATGTGGTTTTTAATTCCGATGGCAACGGCGAAAAAACCTTTACCCCACCCTTTGAACATGTACGGGAATTCGCCAAGAGAATGGCTTAGTGGCNTCGAAAAGAGAAACCGTAACAAACCAGGTGCTTGCATGGCTGTTCGTCAAGCTCGNTGTTCGTGAGAAATGTTGGGGGTTAAGTCNCNCGCAAACGAANGCGCAAACCCCTTGTCCATTAGTTGCTAAACATTTAGNTNTGGGCAAACTCCTAATGAGAAACTNGCCGGTGACAAAAACCGGAGGAAAAGGTNGGGGNANTGACGTCAAAAGTCNCNTNCATGGNCCCCTTATAGNGNTGGGGCTANCNANCACGTCNANTACNAATGGCTGGTACNNAAAAGGGTTGCCAAAACCCGCGANGGGNGGAAGCTAATCCCATAAAACCCCAGTCGTAGTCCNNGGATCGCCAGTCTGCNAACTCGACTGCGTGAAGNNGGAATCGCTAGTAATCGTNGNNCAGNATGTCACGGTGNATACGTTCCCGGGTCTTGTANNCNCCGCCCGTCNCNCCATGGGAGCGGGTTCTGCCAGAAGTAGTTAGCTTAACCGCAAGGA

>FW305-F5

CGGCAGCGCGGGAGCAATCCTGGCGGCGAGTGGCGAACGGGTGAGTAATACATCGGAACGTGCCCAATCGTGGGGGATAACGCAGCGAAAGCTGTGCTAATACCGCATACGATCTACGGATGAAAGCAGGGGATCGCAAGACCTTGCGCGAATGGAGCGGCCGATGGCAGATTAGGTAGTTGGTGAGGTAAAGGCTCACCAAGCCTTCGATCTGTAGCTGGTCTGAGAGGACGACCAGCCACACTGGGACTGAGACACGGCCCAGACTCCTACGGGAGGCAGCAGTGGGGAATTTTGGACAATGGGCGCAAGCCTGATCCAGCCATGCCGCGTGCAGGATGAAGGCCTTCGGGTTGTAAACTGCTTTTGTACGGAACGAAACGGCCTTTTCTAATAAAGAGGGCTAATGACGGTACCGTAAGAATAAGCACCGGCTAACTACGTGCCAGCAGCCGCGGTAATACGTAGGGTGCAAGCGTTAATCGGAATTACTGGGCGTAAAGCGTGCGCAGGCGGTTATGTAAGACAGTTGTGAAATCCCCGGGCTCAACCTGGGAACTGCATCTGTGACTGCATAGCTAGAGTACGGTAAAGAGGGGATGGAATTCCGCGTGTAGCAGTGAAATGCGTAGATATGCGGAGGAACACCGATGGCGAAGGCAATCCCCTGGACCTGTACTGACGCTCATGCACGAAAGCGTGGGGAGCAAACAGGATTAGATACCCTGGTAGTCCACGCCCTAAACGATGTCAACTGGTTGTTGGGAATTCACTTTCTCAGTAACGAAGCTAACGCGTGAAGTTGACCGCCTGGGGAGTACGGCCGCAAGGTTGAAACTCAAAGGAATTGACGGGGACCCGCACAAGCGGTGGATGATGTGGTTTAATTCGATGCAACGCGAAAAACCTTACCCACCTTTGACATGTACGGAATTCGCCAGAGATGGCGTAGTGCTCGAAAGAGAACCGTAACACAGGTGCTGCATGGCTGTCGTCAGCTCGTGTCGTGAGATGTTGGGTTAAGTCCCGCAACGAGCGCAACCCTTGTCATTAGTTGCTACATTTAGTTGGGCACTCTAATGAGACTGCCGGTGACAAACCGGAGGAAGGTGGGGATGACGTCAAGTCCTCATGGCCCTTATAGGTGGGGCTACACACGTCATACAATGGCTGGTACAAAGGGTTGCCAACCCGCGAGGGGGAGCTAATCCCATAAAACCAGTCGTAGTCCGGATCGCAGTCTGCAACTCGACTGCGTTAAGTCGGAATCGCTAGTAATCGTGGATCNNNNTGTCACGGTGAATACGTTCCCGGGTCTTGTACACACCGCCCGTCACACCATGGGAGCGGGTTCTGCCAGAAGTAGTTAGCTAACCGCAAGGAGGCGATA

>FW305-F6

GCTAATACCGGATAAGCCCTTACGGGGAAAGATTTATCGCCGAAAGATCGGCCCGCGTCTGATTAGCTAGTTGGTGAGGTAATGGCTCACCAAGGCGACGATCAGTAGCTGGTCTGAGAGGATGATCAGCCACATTGGGACTGAGACACGGCCCAAACTCCTACGGGAGGCAGCAGTGGGGAATATTGGACAATGGGGGCAACCCTGATCCAGCCATGCCGCGTGAGTGATGAAGGCCCTAGGGTTGTAAAGCTCTTTTGTGCGGGAAGATAATGACGGTACCGCAAGAATAAGCCCCGGCTAACTTCGTGCCAGCAGCCGCGGTAATACGAAGGGGGCTAGCGTTGCTCGGAATCACTGGGCGTAAAGGGTGCGTAGGCGGGTCTTTAAGTCAGGGGTGAAATCCTGGAGCTCAACTCCAGAACTGCCTTTGATACTGAAGATCTTGAGTTCGGGAGAGGTGAGTGGAACTGCGAGTGTAGAGGTGAAATTCGTAGATATTCGCAAGAACACCAGTGGCGAAGGCGGCTCACTGGCCCGATACTGACGCTGAGGCACGAAAGCGTGGGGAGCAAACAGGATTAGATACCCTGGTAGTCCACGCCGTAAACGATGAATGCCAGCCGTTAGTGGGTTTACTCACTAGTGGCGCAGCTAACGCTTTAAGCATTCCGCCTGGGGGAGTACGGTCGCAAGATTAAAACTCAAAGGAATTGACGGGGGCCCGCACAAGCGGTGGAGCATGTGGTTTAATTCGACGCAACGCGCAGAACCTTACCAGCCCTTGACATGTCCAGGACCGGTCCGCAGAGATGTGACCTTCTCTTTCGGAGCCTGGAGCACAGGTGCTGCATGGGCTGTCCGTCAGCTCGTGTCGTGAGATGTTGGGTTAAGTCCCGCAAACGAGCGCAACCCCCGTCCTTAGTTGCTACCATTTAGTTGAGCACTCTAAGGAGACTGCCGGTGATAAGCCGCGAGGAAGGTGGGGATGACGTCAAGTCCTCATGGCCCTTACGGGCTGGGCTACACACGTGCTACAATGGCGGTGACAATGGGATGCTAAGGGGCGACCCTTCGCAAATCTCAAAAAGCCGTCTCAGTTCGGATTGGGCTCTGCAACTCGAGCCCATGAAGTTGGAATCGCTAGTAATCGTGGATCAGCACGCCACGGTGTATACGTTCCCGGGCCTTGTACACACCGCCCGTCACACCATGGGAGTTGGTTTTACCTGAAGACGGTGCGCTAACCCGCAANGGAGGCAGCC

>FW305-F7

TGCAGTCGAACGGCAGCACGGGCTTTCGGCCTGGTGGCGAGTGGCGAACGGGTGAGTAATACATCGGAACGTGCCCTGTAGTGGGGGATAACTAGTCGAAAGATTAGCTAATACCGCATACGACCTGAGGGTGAAAGCGGGGGACCGTAAGGCCTCGCGCTACAGGAGCGGCCGATGTCTGATTAGCTAGTTGGTGGGGTAAAAGCCTACCAAGGCGACGATCAGTAGCTGGTCTGAGAGGACGATCAGCCACACTGGGACTGAGACACGGCCCAGACTCCTACGGGAGGCAGCAGTGGGGAATTTTGGACAATGGGGGCAACCCTGATCCAGCAATGCCGCGTGTGTGAAGAAGGCCTTCGGGTTGTAAAGCACTTTTGTCCGGAAAGAAATGGCTCTGGTTAATACCCGGGGTCGATGACGGTACCGGAAGAATAAGCACCGGCTAACTACGTGCCAGCAGCCGCGGTAATACGTAGGGTGCGAGCGTTAATCGGAATTACTGGGGCGTAAAGCGTGCGCAGGCGGTTTTTGTAAGACAGGCGTGAAATCCCCCGAGCTCAACTTGGGAATGGCGCTTGTGACTGCAAGGGCTAGAGTATGTCAGAGGGGGGTAGAATTCCACGTGTAGCAGTGAAATGCGTAGAGATGTGGAGGAATACCGATGGCGAAGGCAGCCCCCTGGGACGTCACTGACGCTCATGCACGAAAGCGTGGGGAGCAAACAGGATTAGATACCCTGGTAGTCCACGCCCTAAACGATGTCAACTAGTTGTTGGGGATTCATTTCTTCAGTAACGTAGCTAACGCGTGAAGTTGACCGCCTGGGGAGTACGGTCGCAAGATTAAAACTCAAAGGAATTGACGGGGACCCGCACAAGCGGTGGATGATGTGGATTAATTCGATGCAACGCGAAAAACCTTACCTACCCTTGACATGCCACTAACGAAGCAGAGATGCATTAGGTGCCCGAAAGGGAAAGTGGACACAGGTGCTGCATGGCTGTCGTCAGCTCGTGTCGTGAGATGTTGGGTTAAGTCCCGCAACGAGCGCAACCCTTGTCTCTAGTTGCTACGAAAGGGCACTCTAGAGAGACTGCCGGTGACAAACCGGAGGAAGGTGGGGATGACGTCAAGTCCTCATGGCCCTTATGGGTAGGGCTTCACACGTCATACAATGGTGCGTACAGAGGGTTGCCAACCCGCGAGGGGGAGCTAATCCCAGAAAACGCATCGTAGTCCGGATCGTAGTCTGCAACTCGACTACGTGAAGCTGGAATCGCTAGTAATCGCGGATCAGCATGCCGCGGTGTAATACGTTCCCGGGTCTTGTACACACCGCCCGTCACACC

>FW305-F8

GGCAGCGCGGGAGCAATCCCTGGCGGCGAGTGGCGAACGGGTGAGTAATACATCGGAACGTGCCCAATCGTGGGGGATAACGCAGCGAAAGCTGTGCTAATACCGCATACGATCTACGGATGAAAGCAGGGGATCGCAAGACCTTGCGCGAATGGAGCGGCCGATGGCAGATTAGGTAGTTGGTGAGGTAAAGGCTCACCAAGCCTTCGATCTGTAGCTGGTCTGAGAGGACGACCAGCCACACTGGGACTGAGACACGGCCCAGACTCCTACGGGAGGCAGCAGTGGGGAATTTTGGACAATGGGCGCAAGCCTGATCCAGCCATGCCGCGTGCAGGATGAAGGCCTTCGGGTTGTAAACTGCTTTTGTACGGAACGAAACGGCCTTTTTCTAATAAAGAGGGCTAATGACGGTACCGTAAGAATAAGCACCGGCTAAACTACGTGCCAGCAGCCGCGGTAATACGTAGGGTGCAAGCGTTAATCGGAATTACTGGGCGTAAAGCGTGCGCAGGCGGTTATGTAAGACAGTTGTGAAATCCCCGGGCTCAACCTGGGAACTGCATCTGTGACTGCATAGCTAGAGTACGGTAGAGGGGGGATGGAATTCCGCGTGTAGCAGTGAAATGCGTAGATATGCGGAGGAACACCGATGGCGAAGGCAATCCCCTGGACCTGTACTGACGCTCATGCACGAAAGCGTGGGGAGCAAACAGGATTAGATACCCTGGTAGTCCACGCCCTAAACGATGTCAACTGGTTGTTGGGAATTCACTTTCTCAGTAACGAAGCTAACGCGTGAAGTTGACCGCCTGGGGAGTACGGCCGCAAGGTTGAAACTCAAAGGAATTGACGGGGACCCGCACAAGCGGTGGATGATGTGGTTTAATTCGATGCAACGCGAAAAACCTTACCCACCTTTGACATGTACGGAATTCGCCAGAGATGGCTTAGTGCTCGAAAGAGAACCGTAACACAGGTGCTGCATGGCTGTCGTCAGCTCGTGTCGTGAGATGTTGGGTTAAGTCCCGCAACGAGCGCAACCCTTGTCATTAGTTGCTACATTTAGTTGGGCACTCTAATGAGACTGCCGGTGACAAACCGGAGGAAGGTGGGGATGACGTCAAGTCCTCATGGCCCTTATAGGTGGGGCTACACACGTCATACAATGGCTGGTACAAAGGGTTGCCAACCCGCGAGGGGGAGCTAATCCCATAAAACCAGTCGTAGTCCGGATCGCAGTCTGCAACTCGACTGCGTGAAGTCGGAATCGCTAGTAATCGTGGATCAGAATGTCACGGTGAATACGTTCCCGGGTCTTGTACACACCGCCCGTCACACCATGGGAGCGGGTTCTGCCAGAAGTAGTTAGCTTAACCGCAAGGAGGGCGA

>FW305-F9

GTGGCGAACGGGTGAGTAATACATCGGAACGTGCCCAATCGTGGGGGATAACGCAGCGAAAGCTGTGCTAATACCGCATACGATCTACGGATGAAAGCAGGGGATCGCAAGACCTTGCGCGAATGGAGCGGCCGATGGCAGATTAGGTAGTTGGTGAGGTAAAGGCTCACCAAGCCTTCGATCTGTAGCTGGTCTGAGAGGACGACCAGCCACACTGGGACTGAGACACGGCCCAGACTCCTACGGGAGGCAGCAGTGGGGAATTTTGGACAATGGGCGCAAGCCTGATCCAGCCATGCCGCGTGCAGGATGAAGGCCTTCGGGTTGTAAACTGCTTTTGTACGGAACGAAACGGCCTTTTCTAATAAAGAGGGCTAATGACGGTACCGTAAGAATAAGCACCGGCTAACTACGTGCCAGCAGCCGCGGTAATACGTAGGGTGCAAGCGTTAATCGGAATTACTGGGCGTAAAGCGTGCGCAGGCGGTTATGTAAGACAGTTGTGAAATCCCCGGGCTCAACCTGGGAACTGCATCTGTGACTGCATAGCTAGAGTACGGTAGAGGGGGGATGGAATTCCGCGTGTAGCAGTGAAATGCGTAGATATGCGGAGGAACACCGATGGCGAAGGCAATCCCCTGGACCTGTACTGACGCTCATGCACGAAAGCGTGGGGAGCAAACAGGATTAGATACCCTGGTAGTCCACGCCCTAAACGATGTCAACTGGTTGTTGGGAATTCACTTTCTCAGTAACGAAGCTAACGCGTGAAGTTGACCGCCTGGGGAGTACGGCCGCAAGGTTGAAACTCAAAGGAATTGACGGGGACCCGCACAAGCGGTGGATGATGTGGTTTAATTCGATGCAACGCGAAAAACCTTACCCACCTTTGACATGTACGGAATTCGCCAGAGATGGCTTAGTGCTCGAAAGAGAACCGTAACACAGGTGCTGCATGGCTGTCGTCAGCTCGTGTCGTGAGATGTTGGGTTAAGTCCCGCAACGAGCGCAACCCTTGTCATTAGTTGCTACATTTAGTTGGGCACTCTAATGAGACTGCCGGTGACAAACCGGAGGAAGGTGGGGATGACGTCAAGTCCTCATGGCCCTTATAGGTGGGGCTACACACGTCATACAATGGCTGGTACAAAGGGTTGCCAACCCGCGAGGGGGAGCTAATCCCATAAAACCAGTCGTAGTCCGGATCGCAGTCTGCAACTCGACTGCGTGAAGTCGGAATCGCTAGTAATCGTGGATCAGAATGTCACGGTGAATACGTTCCCGGGTCTTGTACACACCGCCCGTCACACCATGGGAGCGGGTTCTGCC

>FW305-F10

CGGGATAAGCCTGGGAAACTGGGTCTAATACCGGATATGACCTCAGGTTGCATGACTTGGGGTGGAAAGATTTATCGGTGCAGGATGGGCCCGCGGCCTATCAGCTTGTTGGTGGGGTAATGGCCTACCAAGGCGACGACGGGTAGCCGACCTGAGAGGGTGACCGGCCACACTGGGACTGAGACACGGCCCAGACTCCTACGGGAGGCAGCAGTGGGGAATATTGCACAATGGGCGAAAGCCTGATGCAAGCGACGCCGCGTGAGGGATGACGGCCTTCGGGTTGTAAACCTCTTTCAGCAGGGACGAAGCGCAAGTGACGGTACCTGCAGAAGAAGCACCGGCTAACTACGTGCCAGCAGCCGCGGTAATACGTAGGGTGCAAGCGTTGTCCGGAATTACTGGGCGTAAAGAGTTCGTAGGCGGTTTGTCGCGTCGTTTGTGAAAACCAGCAGCTCAACTGCTGGCTTGCAGGCGATACGGGCAGACTTGAGTACTGCAGGGGAGACTGGAATTCCTGGTGTAGCGGTGAAATGCGCAGATATCAGGAGGAACACCGGTGGCGAAGGCGGGTCTCTGGGCAGTAACTGACGCTGAGGAACGAAAGCGTGGGTAGCGAACAGGATTAGATACCCTGGTAGTCCACGCCGTAAACGGTGGGCGCTAGGGTGTGGGTTCCTTCCACGGAATCCGTGCCGTAGCTAACGCATTAAGCGCCCCGCCTGGGGGAGTACGGGCCGCAAGGCTAAAACTCAAAGGAATTGACGGGGGGCCCGCACAAGCGGCGGAGCATGTGGATTAATTCGATGCAACGCGAAGAACCTTACCTGGGTTTGACATATACCGGAAAGCTGCAGAGATGTGGCCCCCCTTGTGGTCGGTATACAGGTGGTGCATGGCTGTCGTCAGCTCGTGTCGTGAGATGTTGGGTTAAGTCCCGCAACGAGCGCAACCCCTATCTTATGTTGCCAGCACGTTATGGTGGGGACTCGTAAGAGACTGCCGGGGTCAACTCGGAGGAAGGTGGGGACGACGTCAAGTCATCATGCCCCTTATGTCCAGGGCTTCACACATGCTACAATGGCCAGTACAGAGGGCTGCGAGACCGTGAGGTGGAGCGAATCCCTTAAAGCTGGTCTCAGTTCGGATCGGGGTCTGCAACTCGACCCCGTGAAGTTGGAGTCGCTAGTAATCGCAGATCAGCAACGCTGCGGTGAATACGTTCCCGGGCCTTGTACACACCGCCCGTCACGTCATGAAAGTCGGTAACACCCGAAGCCGGTGGCTTAACCCC

>FW305-F11

CACGAGCGGCGAACGGGTGAGTAACACGTGGGTGATCTGCCCTGCACTTCGGGATAAGCCTGGGAAACTGGGTCTAATACCGGATATGACCTCCTATCGCATGGTGGGTGGTGGAAAGATTTATCGGTGCAGGATGGGCCCGCGGCCTATCAGCTTGTTGGTGGGGTAATGGCCTACCAAGGCGACGACGGGTAGCCGACCTGAGAGGGTGACCGGCCACACTGGGACTGAGACACGGCCCAGACTCCTACGGGAGGCAGCAGTGGGGAATATTGCACAATGGGCGAAAGCCTGATGCAAGCGACGCCGCGTGAGGGATGACGGCCTTCGGGGTTGTAAACCTCTTTCAGCAGGGACGAAGCGCAAGTGACGGTACCTGCAGAAGAAGCACCGGCTAACTACGTGCCAGCAGCCGCGGTAATACGTAGGGTGCAAGCGTTGTCCGGAATTACTGGGCGTAAAGAGTTCGTAGGCGGTTTGTCGCGTCGTTTGTGAAAACCAGCAGCTCAACTGCTGGCTTGCAGGCGATACGGGCAGACTTGAGTACTGCAGGGGAGACTGGAATTCCTGGTGTAGCGGTGAAATGCGCAGATATCAGGAGGAACACCGGTGGCGAAGGCGGGTCTCTGGGCAGTAACTGACGCTGAGGAACGAAAGCGTGGGTAGCGAACAGGATTAGATACCCTGGTAGTCCACGCCGTAAACGGTGGGCGCTAGGTGTGGGTTCCTTCCACGGAATCCGTGCCGTAGCTAACGCATTAAGCGCCCCGCCTGGGGAGTACGGCCGCAAGGCTAAAACTCAAAGGAATTGACGGGGGCCCGCACAAGCGGCGGAGCATGTGGATTAATTCGATGCAACGCGAAGAACCTTACCTGGGTTTGACATATACCGGAAAGCTGCAGAGATGTGGCCCCCCTTGTGGTCGGTATACAGGTGGTGCATGGCTGTCGTCAGCTCGTGTCGTGAGATGTTGGGTTAAGTCCCGCAACGAGCGCAACCCCTATCTTATGTTGCCAGCACGTTATGGTGGGGACTCGTAAGAGACTGCCGGGGTCAACTCGGAGGAAGGTGGGGACGACGTCAAGTCATCATGCCCCTTATGTCCAGGGCTTCACACATGCTACAATGGCCAGTACAGAGGGCTGCGAGACCGTGAGGTGGAGCGAATCCCTTAAAGCTGGTCTCAGTTCGGATCGGGGTCTGCAACTCGACCCCGTGAAGTTGGAGTCGCTAGTAATCGCAGATCAGCAACGCTGCGGTGAATACGTTCCCGGGCCTTGTACACACCGCCCGTCACGTCATGAAAGTCGGTAACACCCGA

>FW305-F12

ATGCAGTCGACGGCAGCGCGGGAAGCAATCCTGGCGGCGAGTGGCGAACGGGTGAGTAATACATCGGAACGTGCCCAATCGTGGGGGATAACGCAGCGAAAGCTGTGCTAATACCGCATACGATCTACGGATGAAAGCAGGGGATCGCAAGACCTTGCGCGAATGGAGCGGCCGATGGCAGATTAGGTAGTTGGTGAGGTAAAGGCTCACCAAGCCTTCGATCTGTAGCTGGTCTGAGAGGACGACCAGCCACACTGGGACTGAGACACGGCCCAGACTCCTACGGGAGGCAGCAGTGGGGAATTTTGGACAATGGGCGCAAGCCTGATCCAGCCATGCCGCGTGCAGGATGAAGGCCTTCGGGTTGTAAACTGCTTTTGTACGGAACGAAACGGCCTTTTCTAATAAAGAGGGCTAATGACGGTACCGTAAGAATAAGCACCGGCTAACTACGTGCCAGCAGCCGCGGTAATACGTAGGGTGCAAGCGTTAATCGGAATTACTGGGCGTAAAGCGTGCGCAGGCGGTTATGTAAGACAGTTGTGAAATCCCCGGGGCTCAACCTGGGAACTGCATCTGTGACTGCATAGCTAGAGTACGGTAGAGGGGGATGGAATTCCGCGTGTAGCAGTGAAATGCGTAGATATGCGGAGGAACACCGANNGGCGAAGGCAATCCCCCGGGACCTGTACTGACGCTCATGCACGAAAGCGTGGGGAGCAAACAGGATTAGATACCCTGGTAGTCCACGCCCTTAAACGATGTCAAACTGGTTGTTGGGAATTCACTTTCTCAGTAACGAAGCTAACGCGTGAAGTTGACCGCCTGGGGGAGTACGGCCGCAAAGGTTGAAACTCAAAAGGAATTGACGGGGACCCGCACAAAGCGGTGGATGATGTGGTTTAATTCGATGCAACGCGAAAAAACCTTACCCCACCTTTGACATGTACGGAATTCGCCAGAGATGGCTTAGTGCTCGAAAGAGAACCGTAACACCAGGTGCTGCATGGCTGTCGTCAGCTCGTGTCGTGAGATGTTGGGTTAAGTCCCGCAACGAGCGCAACCCTTGTCATTAGTTGCTACATTTAGTTGGGCACTCTAATGAGACTGCCGGTGACAAACCGGAGGAAGGTGGGGATGACGTCAAGTCCTCATGGCCCTTATAGGTGGGGCTACACACGTCATACAATGGCTGGTACAAAGGGTTGCCAACCCGCGAGGGGGAGCTAATCCCATAAAACCAGTCGTAGTCCGGATCGCAGTCTGCTTACTCGACTGCGTGTAAGTCGGAATCGCTAGTAATCGT

>FW305-F13

CACTCTGGGACAAGCCCTGGAAACGGGGTCTAATACCGGATAACACTCTGTCCCGCATGGGACGGGGTTGAAAGCTCCGGCGGTGAAGGATGAGCCCGCGGCCTATCAGCTTGTTGGTGGGGTAATGGCCTACCAAGGCGACGACGGGTAGCCGGCCTGAGAGGGCGACCGGCCACACTGGGACTGAGACACGGCCCAGACTCCTACGGGAGGCAGCAGTGGGGAATATTGCACAATGGGCGAAAGCCTGATGCAGCGACGCCGCGTGAGGGATGACGGCCTTCGGGTTGTAAACCTCTTTCAGCAGGGAAGAAGCGAAAGTGACGGTACCTGCAGAAGAAGCGCCGGCTAACTACGTGCCAGCAGCCGCGGTAATACGTAGGGCGCAAGCGTTGTCCGGAATTATTGGGCGTAAAGAGCTCGTAGGCGGCTTGTCACGTCGGATGTGAAAGCCCGGGGCTTAACCCCGGGTCTGCATTCGATACGGGCTAGCTAGAGTGTGGTAGGGGAGATCGGAATTCCTGGTGTAGCGGTGAAATGCGCAGATATCAGGAGGAACACCGGTGGCGAAGGCGGATCTCTGGGCCATTACTGACGCTGAGGAGCGAAAGCGTGGGGAGCGAACAGGATTAGATACCCTGGTAGTCCACGCCGTAAACGTTGGGAACTAGGTGTTGGCGACATTCCACGTCGTCGGTGCCGCAGCTAACGCATTAAGTTCCCCGCCTGGGGAGTACGGCCGCAAGGCTAAAACTCAAAGGAATTGACGGGGGCCCGCACAAGCAGCGGAGCATGTGGCTTAATTCGACGCAACGCGAAGAACCTTACCAAGGCTTGACATATACCGGAAAGCATCAGAGATGGTGCCCCCCTTGTGGTCGGTATACAGGTGGTGCATGGCTGTCGTCAGCTCGTGTCGTGAGATGTTGGGTTAAGTCCCGCAACGAGCGCAACCCTTGTTCTGTGTTGCCAGCATGCCCTTCGGGGTGATGGGGACTCACAGGAGACTGCCGGGGTCAACTCGGAGGAAGGTGGGGACGACGTCAAGTCATCATGCCCCTTATGTCTTGGGCTGCACACGTGCTACAATGGCCGGTACAATGAGCTGCGATGCCGCGAGGCGGAGCGAATCTCAAAAAGCCGGTCTCAGTTCGGATTGGGGTCTGCAACTCGACCCCATGAAGTCGGAGTTGCTAGTAATCGCAGATCAGCATTGCTGCGGTGAATACGTTCCCGGGCCTTGTACACACCGCCCGTCACGTCACGAAAGTCGGTAACACCCGAAGCCGGGGTCCCAACCCC

>FW305-F14

ACACGAGCGGCGAACGGGTGAGTAACACGTGGGTGATCTGCCCTGCACTTCGGGATAAGCCTGGGAAACTGGGTCTAATACCGGATATGACCTCAGGTTGCATGACTTGGGGTGGAAAGATTTATCGGTGCAGGATGGGCCCGCGGCCTATCAGCTTGTTGGTGGGGTAATGGCCTACCAAGGCGACGACGGGTAGCCGACCTGAGAGGGTGACCGGCCACACTGGGACTGAGACACGGCCCAGACTCCTACGGGAGGCAGCAGTGGGGAATATTGCACAATGGGCGAAAGCCTGATGCAGCGACGCCGCGTGAGGGATGACGGCCTTCGGGTTGTAAACCTCTTTCAGCAGGGACGAAGCGCAAGTGACGGTACCTGCAGAAGAAGCACCGGCTAACTACGTGCCAGCAGCCGCGGTAATACGTAGGGTGCAAGCGTTGTCCGGAATTACTGGGCGTAAAGAGTTCGTAGGCGGTTTGTCGCGTCGTTTGTGAAAACCAGCAGCTCAACTGCTGGCTTGCAGGCGATACGGGCAGACTTGAGTACTGCAGGGGAGACTGGAATTCCTGGTGTAGCGGTGAAATGCGCAGATATCAGGAGGAACACCGGTGGCGAAGGCGGGTCTCTGGGCAGTAACTGACGCTGAGGAACGAAAGCGTGGGTAGCGAACAGGATTAGATACCCTGGTAGTCCACGCCGTAAACGGTGGGCGCTAGGGTGTGGGTTCCTTCCACGGAATCCGTGCCGTAGCTAACGCATTAAGCGCCCCGCCTGGGGGAGTACGGGCCGCAAGGCTAAAACTCAAAGGAATTGACGGGGGGCCCGCACAAGCGGCGGAGCATGTGGATTAATTCGATGCAACGCGAAGAACCTTACCTGGGGTTTGACATATACCGGGAAAAGCTTGCAGAGATGTGGCCCCCCTTGTGGTCGGTATACAGGTGGTGCATGGCTGTCGTCAGCTCGTGTCGTGAGATGTTGGGTTAAGTCCCGCAACGAGCGCAACCCCTATCTTATGTTGCCAGCACGTTATGGTGGGGACTCGTAAGAGACTGCCGGGGTCAACTCGGAGGAAGGTGGGGACGACGTCAAGTCATCATGCCCCTTATGTCCAGGGCTTCACACATGCTACAATGGCCAGTACAGAGGGCTGCGAGACCGTGAGGTGGAGCGAATCCCTTAAAGCTGGTCTCAGTTCGGATCGGGGTCTGCAACTCGACCCCGTGAAGTTGGAGTCGCTAGTAATCGCAGATCAGCAACGCTGCGGTGAATACGTTCCCGGGCCTTGTACACACCGCCCGTCACGTCATGAAAGTCGGTAACACCC

>GW456-11-11-14-LB1

GCAGTCGAGCGGCAGCACGGGTACTTGTACCTGGTGGCGAGCGGCGGACGGGTGAGTAATGCCTAGGAATCTGCCTGGTAGTGGGGGATAACGCTCGGAAACGGACGCTAATACCGCATACGTCCTACGGGAGAAAGCAGGGGACCTTCGGGCCTTGCGCTATCAGATGAGCCTAGGTCGGATTAGCTAGTTGGTGAGGTAATGGCTCACCAAGGCGACGATCCGTAACTGGTCTGAGAGGATGATCAGTCACACTGGAACTGAGACACGGTCCAGACTCCTACGGGAGGCAGCAGTGGGGAATATTGGACAATGGGCGAAAGCCTGATCCAGCCATGCCGCGTGTGTGAAGAAGGTCTTCGGATTGTAAAGCACTTTAAGTTGGGAGGAAGGGCAGTTACCTAATACGTATCTGTTTTGACGTTACCGACAGAATAAGCACCGGCTAACTCTGTGCCAGCAGCCGCGGTAATACAGAGGGTGCAAGCGTTAATCGGAATTACTGGGCGTAAAGCGCGCGTAGGTGGTTTGTTAAGTTGGATGTGAAATCCCCGGGCTCAACCTGGGAACTGCATTCAAAACTGACAAGCTAGAGTATGGTAGAGGGTGGTGGAATTTCCTGTGTAGCGGTGAAATGCGTAGATATAGGAAGGAACACCAGTGGCGAAGGCGACCACCTGGACTGATACTGACACTGAGGTGCGAAAGCGTGGGGAGCAAACAGGATTAGATACCCTGGTAGTCCACGCCGTAAACGATGTCAACTAGCCGTTGGGAGCCTTGAGCTCTTAGTGGCGCAGCTAACGCATTAAGTTGACCGCCTGGGGAGTACGGCCGCAAGGTTAAAACTCAAATGAATTGACGGGGGCCCGCACAAGCGGTGGAGCATGTGGTTTAATTCGAAGCAACGCGAAGAACCTTACCAGGCCTTGACATCCAATGAACTTTCCAGAGATGGATTGGTGCCTTCGGGAACATTGAGACAGGTGCTGCATGGCTGTCGTCAGCTCGTGTCGTGAGATGTTGGGTTAAGTCCCGTAACGAGCGCAACCCTTGTCCTTAGTTACCAGCACGNNATGGTGGGCACTCTAAGGAGACTGCCGGTGACAAACCGGAGGAAGGTGGGGATGACGTCAAGTCATCATGGCCCTTACGGCCTGGGCTACACACGTGCTACAATGGTCGGTACAGAGGGTTGCCAAGCCGCGAGGTGGAGCTAATCCCAGAAAACCGATCGTAGTCCGGATCGCAGTCTGCAACTCGACTGCGTGAAGTCGGAATCGCTAGTAATCGCGAATCAGAATGTCGCGGTGAATACGTTCCCGGGCCTTGTACACACCGCCCGTCACACCATGGGAGTGGGTTGCACCAGAAGTAGCTAGTC

>GW456-11-11-14-LB2

TACTTGTACCTGGTGGCGAGCGGCGGACGGGTGAGTAATGCCTAGGAATCTGCCTAGTAGTGGGGGATAACGTCCGGAAACGGGCGCTAATACCGCATACGTCCTACGGGAGAAAGTGGGGGATCTTCGGACCTCACGCTATTAGATGAGCCTAGGTCGGATTAGCTAGTTGGTGAGGTAATGGCTCACCAAGGCGACGATCCGTAACTGGTCTGAGAGGATGATCAGTCACACTGGAACTGAGACACGGTCCAGACTCCTACGGGAGGCAGCAGTGGGGAATATTGGACAATGGGCGAAAGCCTGATCCAGCCATGCCGCGTGTGTGAAGAAGGTCTTCGGATTGTAAAGCACTTTAAGTTGGGAGGAAGGGCAGTTACCTAATACGTGATTGTTTTGACGTTACCGACAGAATAAGCACCGGCTAACTCTGTGCCAGCAGCCGCGGTAATACAGAGGGTGCAAGCGTTAATCGGAATTACTGGGCGTAAAGCGCGCGTAGGTGGTTTGTTAAGTTGGATGTGAAAGCCCCGGGCTCAACCTGGGAACTGCATCCAAAACTGGCAAGCTAGAGTATGGTAGAGGGTGGTGGAATTTCCTGTGTAGCGGTGAAATGCGTAGATATAGGAAGGAACACCAGTGGCGAAGGCGACCACCTGGACTGATACTGACACTGAGGTGCGAAAGCGTGGGGAGCAAACAGGATTAGATACCCTGGTAGTCCACGCCGTAAACGATGTCAACTAGCCGTTGGGAGCCTTGAGCTCTTAGTGGCGCAGCTAACGCATTAAGTTGACCGCCTGGGGAGTACGGCCGCAAGGTTAAAACTCAAATGAATTGACGGGGGCCCGCACAAGCGGTGGAGCATGTGGTTTAATTCGAAGCAACGCGAAGAACCTTACCAGGCCTTGACATCCAATGAACTTTCTAGAGATAGATTGGTGCCTTCGGGAACATTGAGACAGGTGCTGCATGGCTGTCGTCAGCTCGTGTCGTGAGATGTTGGGTTAAGTCCCGTAACGAGCGCAACCCTTGTCCTTAGTTACCAGCACGTTATGGTGGGCACTCTAAGGAGACTGCCGGTGACAAACCGGAGGAAGGTGGGGATGACGTCAAGTCATCATGGCCCTTACGGCCTGGGCTACACACGTGCTACAATGGTCGGTACAAAGGGTTGCCAAGCCGCGAGGTGGAGCTAATCCCATAAAACCGATCGTAGTCCGGATCGCAGTCTGCAACTCGACTGCGTGAAGTCGGAATCGCTAGTAATCGCGAATCAGAATGTCGCGGTGAATACGTTCCCGGGCCTTGTACACACCGCCCGTCACACCAT

>GW456-11-11-14-LB4

GCAGTCGAGGGGTATAGTTCTTCGGAACTAGAGACCGGCGCACGGGTGCGTAACGCGTATGCAATCTACCTTTTACAGAGGGATAGCCCAGAGAAATTTGGATTAATACCTCATAGCATAGCAATCTCGCATGAGATCACTATTAAAGTCACAACGGTAAAAGATGAGCATGCGTCCCATTAGCTAGTTGGTAAGGTAACGGCTTACCAAGGCTACGATGGGTAGGGGTCCTGAGAGGGAGATCCCCCACACTGGTACTGAGACACGGACCAGACTCCTACGGGAGGCAGCAGTGAGGAATATTGGACAATGGGCGCAAGCCTGATCCAGCCATGCCGCGTGCAGGATGACGGTCCTATGGATTGTAAACTGCTTTTATACGAGAAGAAACACTCCAACGTGTTGGAGCTTGACGGTATCGTAAGAATAAGGATCGGCTAACTCCGTGCCAGCAGCCGCGGTAATACGGAGGATCCAAGCGTTATCCGGAATCATTGGGTTTAAAGGGTCCGTAGGCGGTTTAGTAAGTCAGTGGTGAAAGCCCATCGCTCAACGGTGGAACGGCCATTGATACTGCTAAACTTGAATTATTAGGAAGTAACTAGAATATGTAGTGTAGCGGTGAAATGCTTAGAGATTACATGGAATACCAATTGCGAAGGCAGGTTACTACTAATGGATTGACGCTGATGGACGAAAGCGTGGGTAGCGAACAGGATTAGATACCCTGGTAGTCCACGCCGTAAACGATGGATACTAGCTGTTGGGAGCAATCTCAGTGGCTAAGCGAAAGTGATAAGTATCCCACCTGGGGAGTACGTTCGCAAGAATGAAACTCAAAGGAATTGACGGGGGCCCGCACAAGCGGTGGAGCATGTGGTTTAATTCGATGATACGCGAGGAACCTTACCAAGGCTTAAATGTAGATTGACCGGTTTGGAAACAGATCTTTCGCAAGACAATTTACAAGGTGCTGCATGGTTGTCGTCAGCTCGTGCCGTGAGGTGTCAGGTTAAGTCCTATAACGAGCGCAACCCCTGTTGTTAGTTGCCAGCGAGTCATGTCGGGAACTCTAACAAGACTGCCAGTGCAAACTGTGAGGAAGGTGGGGATGACGTCAAATCATCACGGCCCTTACGCCTTGGGCTACACACGTGCTACAATGGCCGGTACAGAGAGCAGCCACTGGGCGACCAGGAGCGAATCTATAAAACCGGTCACAGTTCGGATCGGAGTCTGCAACTCGACTCCGTGAAGCTGGAATCGCTAGTAATCGGATATCAGCCATGATCCGGTGAATACGTTCCCGGGCCTTGTACACACCGCCCGTCAAGCCATGGAAAGC

>GW456-11-11-14-TSB2

ATGCAGTCGAGCGGCAGCACGGGTACTTGTACCTGGTGGCGAGCGGCGGACGGGTGAGTAATGCCTAGGAATCTGCCTGGTAGTGGGGGATAACGCTCGGAAACGGACGCTAATACCGCATACGTCCTACGGGAGAAAGCAGGGGACCTTCGGGCCTTGCGCTATCAGATGAGCCTAGGTCGGATTAGCTAGTTGGTGAGGTAATGGCTCACCAAGGCGACGATCCGTAACTGGTCTGAGAGGATGATCAGTCACACTGGAACTGAGACACGGTCCAGACTCCTACGGGAGGCAGCAGTGGGGAATATTGGACAATGGGCGAAAGCCTGATCCAGCCATGCCGCGTGTGTGAAGAAGGTCTTCGGATTGTAAAGCACTTTAAGTTGGGAGGAAGGGCAGTTACCTAATACGTATCTGTTTTGACGTTACCGACAGAATAAGCACCGGCTAACTCTGTGCCAGCAGCCGCGGTAATACAGAGGGTGCAAGCGTTAATCGGAATTACTGGGCGTAAAGCGCGCGTAGGTGGTTTGTTAAGTTGGATGTGAAATCCCCGGGCTCAACCTGGGAACTGCATTCAAAACTGACAAGCTAGAGTATGGTAGAGGGTGGTGGAATTTCCTGTGTAGCGGTGAAATGCGTAGATATAGGAAGGAACACCAGTGGCGAAGGCGACCACCTGGACTGATACTGACACTGAGGTGCGAAAGCGTGGGGAGCAAACAGGATTAGATACCCTGGTAGTCCACGCCGTAAACGATGTCAACTAGCCGTTGGGAGCCTTGAGCTCTTAGTGGCGCAGCTAACGCATTAAGTTGACCGCCTGGGGAGTACGGCCGCAAGGTTAAAACTCAAATGAATTGACGGGGGCCCGCACAAGCGGTGGAGCATGTGGTTTAATTCGAAGCAACGCGAAGAACCTTACCAGGCCTTGACATCCAATGAACTTTCCAGAGATGGATTGGTGCCTTCGGGAACATTGAGACAGGTGCTGCATGGCTGTCGTCAGCTCGTGTCGTGAGATGTTGGGTTAAGTCCCGTAACGAGCGCAACCCTTGTCCTTAGTTACCAGCACGTNATGGTGGGCACTCTAAGGAGACTGCCGGTGACAAACCGGAGGAAGGTGGGGATGACGTCAAGTCATCATGGCCCTTACGGCCTGGGCTACACACGTGCTACAATGGTCGGTACAGAGGGTTGCCAAGCCGCGAGGTGGAGCTAATCCCAGAAAACCGATCGTAGTCCGGATCGCAGTCTGCAACTCGACTGCGTGAAGTCGGAATCGCTAGTAATCGCGAATCAGAATGTCGCGGTGAATACGTTCCCGGGCCTTGTACACACCGCCCGTCACACCATGGGAGTGGGTTGCACCAGAAGTAGCTAG

>GW456-11-11-14-TSB4

GTTGCTTGCAACCATGAGACCGGCGCACGGGTGCGTAACGCGTATGCAACCTACCTTCATCTGGGGGATAGCCCGGAGAAATCCGGATTAATACCGCATAAAATCACAGTACGGCATCGTACAATGATCAAACATTTATGGGATGGAGATGGGCATGCGTGTCATTAGTTAGTTGGCGGGGTAACGGCCCACCAAGACCGCGATGACTAGGGGATCTGAGAGGATGACCCCCCACACTGGTACTGAGACACGGACCAGACTCCTACGGGAGGCAGCAGTAAGGAATATTGGTCAATGGGGGCAACCCTGAACCAGCCATGCCGCGTGCAGGAAGACGGCCCTCTGGGTTGTAAACTGCTTTTATTCGGGAATAAACCTGCTTACGTGTAAGCAGCTGAATGTACCGAAGGAATAAGGATCGGCTAACTCCGTGCCAGCAGCCGCGGTAATACGGAGGATCCAAGCGTTATCCGGATTTATTGGGTTTAAAGGGTGCGTAGGCGGCCTGTTAAGTCAGGGGTGAAAGACGGTAGCTCAACTATCGCAGTGCCCTTGATACTGATGGGCTTGAATGGACTAGAGGTAGGCGGAATGTGACAAGTAGCGGTGAAATGCATAGATATGTCACAGAACACCGATTGCGAAGGCAGCTTACTATGGTCTTATTGACGCTGAGGCACGAAAGCGTGGGGATCAAACAGGATTAGATACCCTGGTAGTCCACGCCCTAAACGATGAATACTCGCTGTTGGCGATATACAGTCAGCGGCTAAGCGAAAGCGTTAAGTATTCCACCTGGGGAGTACGCCCGCAAGGGTGAAACTCAAAGGAATTGACGGGGGCCCGCACAAGCGGAGGAGCATGTGGTTTAATTCGATGATACGCGAGGAACCTTACCCGGGCTTGAAAGTTAGTGAAGTATCCAGAGATGGATGCGTCCGCAAGGACACGAAACTAGGTGCTGCATGGCTGTCGTCAGCTCGTGCCGTGAGGTGTTGGGTTAAGTCCCGCAACGAGCGCAACCCCTATGTCTAGTTGCCAGCACGTAATGGTGGGGACTCTAGACAGACTGCCTGTGCAAACAGAGAGGAAGGAGGGGACGACGTCAAGTCATCATGGCCCTTACGTCCGGGGCTACACACGTGCTACAATGGATGGTACAGAGGGCAGCAAGCTGGCAACAGCAAGCAAATCTCAAAAAGCCATTCACAGTTCGGATTGGGGTCTGCAACTCGACCCCATGAAGTTGGATTCGCTAGTAATCGCATATCAGCAATGATGCGGTGAATACGTTCCCGGGCCTTGTACACACCGCCCGTCAAGCCATGGAAGTTGGGGGTACCTAAAGTACG

>GW456-11-11-14-TSB5

CTTCGGATGCTGACGAGTGGCGAACGGGTGAGTAATACATCGGAACGTGCCCGAGAGTGGGGGATAACGAGGCGAAAGCTTTGCTAATACCGCATACGATCTCAGGATGAAAGCAGGGGACCGCAAGGCCTTGCGCTCACGGAGCGGCCGATGGCAGATTAGGTAGTTGGTGGGATAAAAGCTTACCAAGCCGACGATCTGTAGCTGGTCTGAGAGGACGACCAGCCACACTGGGACTGAGACACGGCCCAGACTCCTACGGGAGGCAGCAGTGGGGAATTTTGGACAATGGGCGCAAGCCTGATCCAGCCATGCCGCGTGCAGGATGAAGGCCTTCGGGTTGTAAACTGCTTTTGTACGGAACGAAAAGACTCCTTCTAATAAAGGGGGTCCATGACGGTACCGTAAGAATAAGCACCGGCTAACTACGTGCCAGCAGCCGCGGTAATACGTAGGGTGCGAGCGTTAATCGGAATTACTGGGCGTAAAGCGTGCGCAGGCGGTTATATAAGACAGATGTGAAATCCCCGGGCTCAACCTGGGAACTGCATTTGTGACTGTATAGCTAGAGTACGGTAGAGGGGGATGGAATTCCGCGTGTAGCAGTGAAATGCGTAGATATGCGGAGGAACACCGATGGCGAAGGCAATCCCCTGGACCTGTACTGACGCTCATGCACGAAAGCGTGGGGAGCAAACAGGATTAGATACCCTGGTAGTCCACGCCCTAAACGATGTCAACTGGTTGTTGGGTCTTCACTGACTCAGTAACGAAGCTAACGCGTGAAGTTGACCGCCTGGGGAGTACGGCCGCAAGGTTGAAACTCAAAGGAATTGACGGGGACCCGCACAAGCGGTGGATGATGTGGTTTAATTCGATGCAACGCGAAAAACCTTACCCACCTTTGACATGTATGGAATCCTTTAGAGATAGAGGAGTGCTCGAAAGAGAGCCATAACACAGGTGCTGCATGGCTGTCGTCAGCTCGTGTCGTGAGATGTTGGGTTAAGTCCCGCAACGAGCGCAACCCTTGCCATTAGTTGCTACGAAAGGGCACTCTAATGGGACTGCCGGTGACAAACCGGAGGAAGGTGGGGATGACGTCAAGTCCTCATGGCCCTTATAGGTGGGGCTACACACGTCATACAATGGCTGGTACAGAGGGTTGCCAACCCGCGAGGGGGAGCCAATCCCATAAAGCCAGTCGTAGTCCGGATCGCAGTCTGCAACTCGACTGCGTGAAGTCGGAATCGCTAGTAATCGCGGATCAGAATGTCGCGGTGAATACGTTCCCGGGTCTTGTACACACCGCCCGTCACACCATGGGAGCGGGTTCTGCCAGAAGTAGT

>GW456-11-11-14-TSB8

AAGGTGCTGACGAGTGGCGAACGGGTGAGTAATGCATCGGAACGTGCCCAGTCGTGGGGGATAACGCAGCGAAAGCTGCGCTAATACCGCATACGATCTATGGATGAAAGCGGGGGACCGTAAGGCCTCGCGCGATTGGAGCGGCCGATGTCAGATTAGGTAGTTGGTGGGGTAAAGGCTCACCAAGCCGACGATCTGTAGCTGGTCTGAGAGGACGACCAGCCACACTGGGACTGAGACACGGCCCAGACTCCTACGGGAGGCAGCAGTGGGGAATTTTGGACAATGGGCGCAAGCCTGATCCAGCAATGCCGCGTGCAGGAAGAAGGCCTTCGGGTTGTAAACTGCTTTTGTACGGAACGAAAAGGCTCTGGTTAATACCTGGGGCTCATGACGGTACCGTAAGAATAAGCACCGGCTAACTACGTGCCAGCAGCCGCGGTAATACGTAGGGTGCAAGCGTTAATCGGAATTACTGGGCGTAAAGCGTGCGCAGGCGGTTTTGTAAGACAGGCGTGAAATCCCCGGGCTCAACCTGGGAATTGCGCTTGTGACTGCAAGGCTGGAGTGCGGCAGAGGGGGATGGAATTCCGCGTGTAGCAGTGAAATGCGTAGATATGCGGAGGAACACCGATGGCGAAGGCAATCCCCTGGGCCTGCACTGACGCTCATGCACGAAAGCGTGGGGAGCAAACAGGATTAGATACCCTGGTAGTCCACGCCCTAAACGATGTCAACTGGTTGTTGGGTCTCTTCTGACTCAGTAACGAAGCTAACGCGTGAAGTTGACCGCCTGGGGAGTACGGCCGCAAGGTTGAAACTCAAAGGAATTGACGGGGACCCGCACAAGCGGTGGATGATGTGGTTTAATTCGATGCAACGCGAAAAACCTTACCCACCTTTGACATGTACGGAATTTGCCAGAGATGGCTTAGTGCTCGAAAGAGAGCCGTAACACAGGTGCTGCATGGCTGTCGTCAGCTCGTGTCGTGAGATGTTGGGTTAAGTCCCGCAACGAGCGCAACCCTTGTCATTAGTTGCTACGAAAGGGCACTCTAATGAGACTGCCGGTGACAAACCGGAGGAAGGTGGGGATGACGTCAAGTCCTCATGGCCCTTATAGGTGGGGCTACACACGTCATACAATGGCTGGTACAAAGGGTTGCCAACCCGCGAGGGGGAGCTAATCCCATAAAGCCAGTCGTAGTCCGGATCGCAGTCTGCAACTCGACTGCGTGAAGTCGGAATCGCTAGTAATCGTGGATCAGCATGTCACGGTGAATACGTTCCCGGGTCTTGTACACACCGCCCGTCACACCATGGGAGCGGGTCTCGCCAGAAGTAGTTAGCCTAACCGCAAGGA

>GW456-12-1-14-LB1

ACGGGTACTTGTACCTGGTGGCGAGCGGCGGACGGGTGAGTAATGCCTAGGAATCTGCCTGGTAGTGGGGGATAACGCTCGGAAACGGACGCTAATACCGCATACGTCCTACGGGAGAAAGCAGGGGACCTTCGGGCCTTGCGCTATCAGATGAGCCTAGGTCGGATTAGCTAGTTGGTGAGGTAATGGCTCACCAAGGCGACGATCCGTAACTGGTCTGAGAGGATGATCAGTCACACTGGAACTGAGACACGGTCCAGACTCCTACGGGAGGCAGCAGTGGGGAATATTGGACAATGGGCGAAAGCCTGATCCAGCCATGCCGCGTGTGTGAAGAAGGTCTTCGGATTGTAAAGCACTTTAAGTTGGGAGGAAGGGCAGTTACCTAATACGTATCTGTTTTGACGTTACCGACAGAATAAGCACCGGCTAACTCTGTGCCAGCAGCCGCGGTAATACAGAGGGTGCAAGCGTTAATCGGAATTACTGGGCGTAAAGCGCGCGTAGGTGGTTTGTTAAGTTGGATGTGAAATCCCCGGGCTCAACCTGGGAACTGCATTCAAAACTGACAAGCTAGAGTATGGTAGAGGGTGGTGGAATTTCCTGTGTAGCGGTGAAATGCGTAGATATAGGAAGGAACACCAGTGGCGAAGGCGACCACCTGGACTGATACTGACACTGAGGTGCGAAAGCGTGGGGAGCAAACAGGATTAGATACCCTGGTAGTCCACGCCGTAAACGATGTCAACTAGCCGTTGGGAGCCTTGAGCTCTTAGTGGCGCAGCTAACGCATTAAGTTGACCGCCTGGGGAGTACGGCCGCAAGGTTAAAACTCAAATGAATTGACGGGGGCCCGCACAAGCGGTGGAGCATGTGGTTTAATTCGAAGCAACGCGAAGAACCTTACCAGGCCTTGACATCCAATGAACTTTCCAGAGATGGATTGGTGCCTTCGGGAACATTGAGACAGGTGCTGCATGGCTGTCGTCAGCTCGTGTCGTGAGATGTTGGGTTAAGTCCCGTAACGAGCGCAACCCTTGTCCTTAGTTACCAGCACGNNATGGTGGGCACTCTAAGGAGACTGCCGGTGACAAACCGGAGGAAGGTGGGGATGACGTCAAGTCATCATGGCCCTTACGGCCTGGGCTACACACGTGCTACAATGGTCGGTACAGAGGGTTGCCAAGCCGCGAGGTGGAGCTAATCCCAGAAAACCGATCGTAGTCCGGATCGCAGTCTGCAACTCGACTGCGTGAAGTCGGAATCGCTAGTAATCGCGAATCAGAATGTCGCGGTGAATACGTTCCCGGGCCTTGTACACACCGCCCGTCACACCATGGGAGTGGGTTGCACCAGAAGTAGCTAGTCTA

>GW456-12-1-14-LB2

CTTGCTCCTGGATTCAGCGGCGGACGGGTGAGTAATGCCTAGGAATCTGCCTGNNNGTGGGGGACAACGTTTCGAAAGGAACGCTAATACCGCATACGTCCTACGGGAGAAAGCAGGGGACCTTCGGGCCTTGCGCTATCAGATGAGCCTAGGTCGGATTAGCTAGTTGGTGAGGTAATGGCTCACCAAGGCGACGATCCGTAACTGGTCTGAGAGGATGATCAGTCACACTGGAACTGAGACACGGTCCAGACTCCTACGGGAGGCAGCAGTGGGGAATATTGGACAATGGGCGAAAGCCTGATCCAGCCATGCCGCGTGTGTGAAGAAGGTCTTCGGATTGTAAAGCACTTTAAGTTGGGAGGAAGGGCAGTAAATTAATACTTTGCTGTTTTGACGTTACCGACAGAATAAGCACCGGCTAACTCTGTGCCAGCAGCCGCGGTAATACAGAGGGTGCAAGCGTTAATCGGAATTACTGGGCGTAAAGCGCGCGTAGGTGGTTCGTTAAGTTGGATGTGAAATCCCCGGGCTCAACCTGGGAACTGCATCCAAAACTGGCGAGCTAGAGTATGGTAGAGGGTGGTGGAATTTCCTGTGTAGCGGTGAAATGCGTAGATATAGGAAGGAACACCAGTGGCGAAGGCGACCACCTGGACTGATACTGACACTGAGGTGCGAAAGCGTGGGGAGCAAACAGGATTAGATACCCTGGTAGTCCACGCCGTAAACGATGTCAACTAGCCGTTGGGAGCCTTGAGCTCTTAGTGGCGCAGCTAACGCATTAAGTTGACCGCCTGGGGAGTACGGCCGCAAGGTTAAAACTCAAATGAATTGACGGGGGCCCGCACAAGCGGTGGAGCATGTGGTTTAATTCGAAGCAACGCGAAGAACCTTACCAGGCCTTGACATCCAATGAACTTTCCAGAGATGGATTGGTGCCTTCGGGAACATTGARACAGGTGCTGCATGGSTGTCGTCASCTCGTGTCGTGAGATGTTGGGTTAAGTCCCGTAACGAGCGCAACCCTTGTCCTTAGTTACCAGCACGTAATGGTGGGCACTCTAAGGAGACTGCCGGTGACAAACCGGAGGAAGGTGGGGATGACGTCAAGTCATCATGGCCCTTACGGCCTGGGCTACACACGTGCTACAATGGTCGGTACAAAGGGTTGCCAAGCCGCGAGGTGGAGCTAATCCCATAAAACCGATCGTAGTCCGGATCGCAGTCTGCAACTCGACTGCGTGAAGTCGGAATCGCTAGTAATCGCGAATCAGAATGTCGCGGTGAATACGTTCCCGGGCCTTGTACACACCGCCCGTCACACCATGGGAGTGGGTTGCACCAGAAGTAGCTAGTCTAACC

>GW456-12-1-14-LB3

GCTGACGAGTGGCGAACGGGTGAGTAATACATCGGAACGTGCCCGAGAGTGGGGGATAACGAGGCGAAAGCTTTGCTAATACCGCATACGATCTCAGGATGAAAGCAGGGGACCGCAAGGCCTTGCGCTCACGGAGCGGCCGATGGCAGATTAGGTAGTTGGTGGGATAAAAGCTTACCAAGCCGACGATCTGTAGCTGGTCTGAGAGGACGACCAGCCACACTGGGACTGAGACACGGCCCAGACTCCTACGGGAGGCAGCAGTGGGGAATTTTGGACAATGGGCGCAAGCCTGATCCAGCCATGCCGCGTGCAGGATGAAGGCCTTCGGGTTGTAAACTGCTTTTGTACGGAACGAAAAGACTCCTTCTAATAAAGGGGGTCCATGACGGTACCGTAAGAATAAGCACCGGCTAACTACGTGCCAGCAGCCGCGGTAATACGTAGGGTGCGAGCGTTAATCGGAATTACTGGGCGTAAAGCGTGCGCAGGCGGTTATATAAGACAGATGTGAAATCCCCGGGCTCAACCTGGGAACTGCATTTGTGACTGTATAGCTAGAGTACGGTAGAGGGGGATGGAATTCCGCGTGTAGCAGTGAAATGCGTAGATATGCGGAGGAACACCGATGGCGAAGGCAATCCCCTGGACCTGTACTGACGCTCATGCACGAAAGCGTGGGGAGCAAACAGGATTAGATACCCTGGTAGTCCACGCCCTAAACGATGTCAACTGGTTGTTGGGTCTTCACTGACTCAGTAACGAAGCTAACGCGTGAAGTTGACCGCCTGGGGAGTACGGCCGCAAGGTTGAAACTCAAAGGAATTGACGGGGACCCGCACAAGCGGTGGATGATGTGGTTTAATTCGATGCAACGCGAAAAACCTTACCCACCTTTGACATGTATGGAATCCTTTAGAGATAGAGGAGTGCTCGAAAGAGAGCCATAACACAGGTGCTGCATGGCTGTCGTCAGCTCGTGTCGTGAGATGTTGGGTTAAGTCCCGCAACGAGCGCAACCCTTGCCATTAGTTGCTACGAAAGGGCACTCTAATGGGACTGCCGGTGACAAACCGGAGGAAGGTGGGGATGACGTCAAGTCCTCATGGCCCTTATAGGTGGGGCTACACACGTCATACAATGGCTGGTACAGAGGGTTGCCAACCCGCGAGGGGGAGCCAATCCCATAAAGCCAGTCGTAGTCCGGATCGCAGTCTGCAACTCGACTGCGTGAAGTCGGAATCGCTAGTAATCGCGGATCAGAATGTCGCGGTGAATACGTTCCCGGGTCTTGTACACACCGCCCGTCACACCATGGGAGCGGGTTCTGCCAGAAGTAGTTAGCC

>GW456-12-1-14-TSB1

ATGCAGTCGAGCGGCAGCACGGGTACTTGTACCTGGTGGCGAGCGGCGGACGGGTGAGTAATGCCTAGGAATCTGCCTGGTAGTGGGGGATAACGCTCGGAAACGGACGCTAATACCGCATACGTCCTACGGGAGAAAGCAGGGGACCTTCGGGCCTTGCGCTATCAGATGAGCCTAGGTCGGATTAGCTAGTTGGTGAGGTAATGGCTCACCAAGGCGACGATCCGTAACTGGTCTGAGAGGATGATCAGTCACACTGGAACTGAGACACGGTCCAGACTCCTACGGGAGGCAGCAGTGGGGAATATTGGACAATGGGCGAAAGCCTGATCCAGCCATGCCGCGTGTGTGAAGAAGGTCTTCGGATTGTAAAGCACTTTAAGTTGGGAGGAAGGGCAGTTACCTAATACGTATCTGTTTTGACGTTACCGACAGAATAAGCACCGGCTAACTCTGTGCCAGCAGCCGCGGTAATACAGAGGGTGCAAGCGTTAATCGGAATTACTGGGCGTAAAGCGCGCGTAGGTGGTTTGTTAAGTTGGATGTGAAATCCCCGGGCTCAACCTGGGAACTGCATTCAAAACTGACAAGCTAGAGTATGGTAGAGGGTGGTGGAATTTCCTGTGTAGCGGTGAAATGCGTAGATATAGGAAGGAACACCAGTGGCGAAGGCGACCACCTGGACTGATACTGACACTGAGGTGCGAAAGCGTGGGGAGCAAACAGGATTAGATACCCTGGTAGTCCACGCCGTAAACGATGTCAACTAGCCGTTGGGAGCCTTGAGCTCTTAGTGGCGCAGCTAACGCATTAAGTTGACCGCCTGGGGAGTACGGCCGCAAGGTTAAAACTCAAATGAATTGACGGGGGCCCGCACAAGCGGTGGAGCATGTGGTTTAATTCGAAGCAACGCGAAGAACCTTACCAGGCCTTGACATCCAATGAACTTTCCAGAGATGGATTGGTGCCTTCGGGAACATTGAGACAGGTGCTGCATGGCTGTCGTCAGCTCGTGTCGTGAGATGTTGGGTTAAGTCCCGTAACGAGCGCAACCCTTGTCCTTAGTTACCAGCACGTNATGGTGGGCACTCTAAGGAGACTGCCGGTGACAAACCGGAGGAAGGTGGGGATGACGTCAAGTCATCATGGCCCTTACGGCCTGGGCTACACACGTGCTACAATGGTCGGTACAGAGGGTTGCCAAGCCGCGAGGTGGAGCTAATCCCAGAAAACCGATCGTAGTCCGGATCGCAGTCTGCAACTCGACTGCGTGAAGTCGGAATCGCTAGTAATCGCGAATCAGAATGTCGCGGTGAATACGTTCCCGGGCCTTGTACACACCGCCCGTCACACCATGGGAGTGGGTTGCACCAGAAGTAGCTAGTCT

>GW456-12-1-14-TSB4

CGGATCCTTCGGGATTAGTGGCGGACGGGTGAGTAACACGTGGGAACGTGCCCTTTGGTTCGGAACAACTCAGGGAAACTTGAGCTAATACCGGATGTGCCCTTCGGGGGAAAGATTTATCGCCATTGGAGCGGCCCGCGTAGGATTAGCTAGTTGGTGGGGTAAAGGCCCACCAAGGCTACGATCCTTAGCTGGTCTGAGAGGATGATCAGCCACATTGGGACTGAGACACGGCCCAAACTCCTACGGGAGGCAGCAGTGGGGAATCTTGCGCAATGGGCGAAAGCCTGACGCAGCCATGCCGCGTGAATGATGAAGGTCTTAGGATTGTAAAATTCTTTCACCGGGGACGATAATGACGGTACCCGGAGAAGAAGCCCCGGCTAACTTCGTGCCAGCAGCCGCGGTAATACGAAGGGGGCTAGCGTTGCTCGGAATTACTGGGCGTAAAGGGAGCGTAGGCGGACTGTTTAGTCAGAGGTGAAAGCCCAGGGCTCAACCTTGGAATTGCCTTTGATACTGGCAGTCTTGAGTACGGAAGAGGTATGTGGAACTCCGAGTGTAGAGGTGAAATTCGTAGATATTCGGAAGAACACCAGTGGCGAAGGCGACATACTGGTCCGTTACTGACGCTGAGGCTCGAAAGCGTGGGGAGCAAACAGGATTAGATACCCTGGTAGTCCACGCCGTAAACGATGAGTGCTAGTTGTCGGCATGCATGCATGTCGGTGACGCAGCTAACGCATTAAGCACTCCGCCTGGGGAGTACGGTCGCAAGATTAAAACTCAAAGGAATTGACGGGGGCCCGCACAAGCGGTGGAGCATGTGGTTTAATTCGAAGCAACGCGCAGAACCTTACCACCTTTTGACATGCCCGGACCACCAGAGAGATCTGGCTTTCCCTTCGGGGACTGGGACACAGGTGCTGCATGGCTGTCGTCAGCTCGTGTCGTGAGATGTTGGGTTAAGTCCCGCAACGAGCGCAACCCTCGCGATTAGTTGCCATCAGGTTTGGCTGGGCACTCTAATCGTACTGCCGGARTTAATCCGGAGGAAGGCGGGGATGACGTCAAGTCCTCATGGCCCTTACAAGGTGGGCTACACACGTGCTACAATGGCGACTACAGAGGGCTGCAATCCCGCGAGGGGGAGCCAATCCCTAAAAGTCGTCTCAGTTCGGATTGTTCTCTGCAACTCGAGAGCATGAAGTTGGAATCGCTAGTAATCGCGGATCAGCATGCCGCGGTGAATACGTTCCCGGGCCTTGTACACACCGCCCGTCACACCATGGGAGTTGGCTTTACCCGAAGGCGCTGCGCTAACCGCAAGGAGGCAGGCG

>GW456-12-1-14-TSB6

GGGAGCTTGCTCCTGAATTCAGCGGCGGACGGGTGAGTAATGCCTAGGAATCTGCCTGGTAGTGGGGGACAACGTTTCGAAAGGAACGCTAATACCGCATACGTCCTACGGGAGAAAGCAGGGGACCTTCGGGCCTTGCGCTATCAGATGAGCCTAGGTCGGATTAGCTAGTTGGTGAGGTAATGGCTCACCAAGGCGACGATCCGTAACTGGTCTGAGAGGATGATCAGTCACACTGGAACTGAGACACGGTCCAGACTCCTACGGGAGGCAGCAGTGGGGAATATTGGACAATGGGCGAAAGCCTGATCCAGCCATGCCGCGTGTGTGAAGAAGGTCTTCGGATTGTAAAGCACTTTAAGTTGGGAGGAAGGGTTGTAGATTAATACTCTGCAATTTTGACGTTACCGACAGAATAAGCACCGGCTAACTCTGTGCCAGCAGCCGCGGTAATACAGAGGGTGCAAGCGTTAATCGGAATTACTGGGCGTAAAGCGCGCGTAGGTGGTTCGTTAAGTTGGATGTGAAATCCCCGGGCTCAACCTGGGAACTGCATCCAAAACTGGCGAGCTAGAGTATGGTAGAGGGTGGTGGAATTTCCTGTGTAGCGGTGAAATGCGTAGATATAGGAAGGAACACCAGTGGCGAAGGCGACCACCTGGACTGATACTGACACTGAGGTGCGAAAGCGTGGGGAGCAAACAGGATTAGATACCCTGGTAGTCCACGCCGTAAACGATGTCAACTAGCCGTTGGGAGCCTTGAGCTCTTAGTGGCGCAGCTAACGCATTAAGTTGACCGCCTGGGGAGTACGGCCGCAAGGTTAAAACTCAAATGAATTGACGGGGGCCCGCACAAGCGGTGGAGCATGTGGTTTAATTCGAAGCAACGCGAAGAACCTTACCAGGCCTTGACATCCAATGAACTTTCCAGAGATGGATTGGTGCCTTCGGGAACATTGAGACAGGTGCTGCATGGCTGTCGTCAGCTCGTGTCGTGAGATGTTGGGTTAAGTCCCGTAACGAGCGCAACCCTTGTCCTTAGTTACCAGCACGTTATGGTGGGCACTCTAAGGAGACTGCCGGTGACAAACCGGAGGAAGGTGGGGATGACGTCAAGTCATCATGGCCCTTACGGCCTGGGCTACACACGTGCTACAATGGTCGGTACAAAGGGTTGCCAAGCCGCGAGGTGGAGCTAATCCCATAAAACCGATCGTAGTCCGGATCGCAGTCTGCAACTCGACTGCGTGAAGTCGGAATCGCTAGTAATCGCGAATCAGAATGTCGCGGTGAATACGTTCCCGGGCCTTGTACACACCGCCCGTCACACCATGGGAGTGGGTTGCACCAGAAGTAGCTAGTCTAACC

>GW456-12-10-14-LB1

CGGTAACAGGTCTTCGGATGCTGACGAGTGGCGAACGGGTGAGTAATACATCGGAACGTGCCCGAGAGTGGGGGATAACGAGGCGAAAGCTTTGCTAATACCGCATACGATCTCAGGATGAAAGCAGGGGACCGCAAGGCCTTGCGCTCACGGAGCGGCCGATGGCAGATTAGGTAGTTGGTGGGATAAAAGCTTACCAAGCCGACGATCTGTAGCTGGTCTGAGAGGACGACCAGCCACACTGGGACTGAGACACGGCCCAGACTCCTACGGGAGGCAGCAGTGGGGAATTTTGGACAATGGGCGCAAGCCTGATCCAGCCATGCCGCGTGCAGGATGAAGGCCTTCGGGTTGTAAACTGCTTTTGTACGGAACGAAAAGACTCCTTCTAATAAAGGGGGTCCATGACGGTACCGTAAGAATAAGCACCGGCTAACTACGTGCCAGCAGCCGCGGTAATACGTAGGGTGCGAGCGTTAATCGGAATTACTGGGCGTAAAGCGTGCGCAGGCGGTTATATAAGACAGATGTGAAATCCCCGGGCTCAACCTGGGAACTGCATTTGTGACTGTATAGCTAGAGTACGGTAGAGGGGGATGGAATTCCGCGTGTAGCAGTGAAATGCGTAGATATGCGGAGGAACACCGATGGCGAAGGCAATCCCCTGGACCTGTACTGACGCTCATGCACGAAAGCGTGGGGAGCAAACAGGATTAGATACCCTGGTAGTCCACGCCCTAAACGATGTCAACTGGTTGTTGGGTCTTCACTGACTCAGTAACGAAGCTAACGCGTGAAGTTGACCGCCTGGGGAGTACGGCCGCAAGGTTGAAACTCAAAGGAATTGACGGGGACCCGCACAAGCGGTGGATGATGTGGTTTAATTCGATGCAACGCGAAAAACCTTACCCACCTTTGACATGTATGGAATCCTTTAGAGATAGAGGAGTGCTCGAAAGAGAGCCATAACACAGGTGCTGCATGGCTGTCGTCAGCTCGTGTCGTGAGATGTTGGGTTAAGTCCCGCAACGAGCGCAACCCTTGCCATTAGTTGCTACGAAAGGGCACTCTAATGGGACTGCCGGTGACAAACCGGAGGAAGGTGGGGATGACGTCAAGTCCTCATGGCCCTTATAGGTGGGGCTACACACGTCATACAATGGCTGGTACAGAGGGTTGCCAACCCGCGAGGGGGAGCCAATCCCATAAAGCCAGTCGTAGTCCGGATCGCAGTCTGCAACTCGACTGCGTGAAGTCGGAATCGCTAGTAATCGCGGATCAGAATGTCGCGGTGAATACGTTCCCGGGTCTTGTACACACCGCCCGTCACACCATGGGAGCGGGTTCTGCCAGAAGTAGTTAGCCTAACCGCAAGGAGGGCGA

>GW456-12-10-14-LB2

TACTTGTACCTGGTGGCGAGCGGCGGACGGGTGAGTAATGCCTAGGAATCTGCCTGGTAGTGGGGGATAACGCTCGGAAACGGACGCTAATACCGCATACGTCCTACGGGAGAAAGCAGGGGACCTTCGGGCCTTGCGCTATCAGATGAGCCTAGGTCGGATTAGCTAGTTGGTGAGGTAATGGCTCACCAAGGCGACGATCCGTAACTGGTCTGAGAGGATGATCAGTCACACTGGAACTGAGACACGGTCCAGACTCCTACGGGAGGCAGCAGTGGGGAATATTGGACAATGGGCGAAAGCCTGATCCAGCCATGCCGCGTGTGTGAAGAAGGTCTTCGGATTGTAAAGCACTTTAAGTTGGGAGGAAGGGCAGTTACCTAATACGTATCTGTTTTGACGTTACCGACAGAATAAGCACCGGCTAACTCTGTGCCAGCAGCCGCGGTAATACAGAGGGTGCAAGCGTTAATCGGAATTACTGGGCGTAAAGCGCGCGTAGGTGGTTTGTTAAGTTGGATGTGAAATCCCCGGGCTCAACCTGGGAACTGCATTCAAAACTGACAAGCTAGAGTATGGTAGAGGGTGGTGGAATTTCCTGTGTAGCGGTGAAATGCGTAGATATAGGAAGGAACACCAGTGGCGAAGGCGACCACCTGGACTGATACTGACACTGAGGTGCGAAAGCGTGGGGAGCAAACAGGATTAGATACCCTGGTAGTCCACGCCGTAAACGATGTCAACTAGCCGTTGGGAGCCTTGAGCTCTTAGTGGCGCAGCTAACGCATTAAGTTGACCGCCTGGGGAGTACGGCCGCAAGGTTAAAACTCAAATGAATTGACGGGGGCCCGCACAAGCGGTGGAGCATGTGGTTTAATTCGAAGCAACGCGAAGAACCTTACCAGGCCTTGACATCCAATGAACTTTCCAGAGATGGATTGGTGCCTTCGGGAACATTGAGACAGGTGCTGCATGGCTGTCGTCAGCTCGTGTCGTGAGATGTTGGGTTAAGTCCCGTAACGAGCGCAACCCTTGTCCTTAGTTACCAGCACGTNATGGTGGGCACTCTAAGGAGACTGCCGGTGACAAACCGGAGGAAGGTGGGGATGACGTCAAGTCATCATGGCCCTTACGGCCTGGGCTACACACGTGCTACAATGGTCGGTACAGAGGGTTGCCAAGCCGCGAGGTGGAGCTAATCCCAGAAAACCGATCGTAGTCCGGATCGCAGTCTGCAACTCGACTGCGTGAAGTCGGAATCGCTAGTAATCGCGAATCAGAATGTCGCGGTGAATACGTTCCCGGGCCTTGTACACACCGCCCGTCACACCATGGGAGTGGGTTGCACCAGAAGTAGCTAGTCTAA

>GW456-12-10-14-LB3

CTTCGGACTTAGTGGCGGACGGGTGAGTAACACGTGGGAACGTGCCTTTAGGTTCGGAATAACTCAGGGAAACTTGTGCTAATACCGAATGTGCCCTTCGGGGGAAAGATTTATCGCCTTTAGAGCGGCCCGCGTCTGATTAGCTAGTTGGTGAGGTAAAGGCTCACCAAGGCGACGATCAGTAGCTGGTCTGAGAGGATGATCAGCCACATTGGGACTGAGACACGGCCCAAACTCCTACGGGAGGCAGCAGTGGGGAATCTTGCGCAATGGGCGAAAGCCTGACGCAGCCATGCCGCGTGAATGATGAAGGTCTTAGGATTGTAAAATTCTTTCACCGGGGACGATAATGACGGTACCCGGAGAAGAAGCCCCGGCTAACTTCGTGCCAGCAGCCGCGGTAATACGAAGGGGGCTAGCGTTGCTCGGAATTACTGGGCGTAAAGGGAGCGTAGGCGGACATTTAAGTCAGGGGTGAAATCCCGGGGCTCAACCTCGGAATTGCCTTTGATACTGGGTGTCTTGAGTGTGAGAGAGGTATGTGGAACTCCGAGTGTAGAGGTGAAATTCGTAGATATTCGGAAGAACACCAGTGGCGAAGGCGACATACTGGCTCATTACTGACGCTGAGGCTCGAAAGCGTGGGGAGCAAACAGGATTAGATACCCTGGTAGTCCACGCCGTAAACGATGATTGCTAGTTGTCGGGATGCATGCATTTCGGTGACGCAGCTAACGCATTAAGCAATCCGCCTGGGGAGTACGGTCGCAAGATTAAAACTCAAAGGAATTGACGGGGGCCCGCACAAGCGGTGGAGCATGTGGTTTAATTCGAAGCAACGCGCAGAACCTTACCACCTTTTGACATGCCCGGACCGCCACAGAGATGTGGCTTTCTCTTCGGAGACTGGGACACAGGTGCTGCATGGCTGTCGTCAGCTCGTGTCGTGAGATGTTGGGTTAAGTCCCGCAACGAGCGCAACCCTCGCCATTAGTTGCCATCATTCAGTTGGGAACTCTAATGGGACTGCCGGTGCTAAGCCGGAGGAAGGTGGGGATGACGTCAAGTCCTCATGGCCCTTACAGGGTGGGCTACACACGTGCTACAATGGCGACTACAGAGGGTTAATCCTTAAAAGTCGTCTCAGTTCGGATTGTCCTCTGCAACTCGAGGGCATGAAGTTGGAATCGCTAGTAATCGCGGATCAGCATGCCGCGGTGAATACGTTCCCGGGCCTTGTACACACCGCCCGTCACACCATGGGAGTTGGTTCTACCCGAAGGCGCTGCGC

>GW456-12-10-14-TSB1

TCTAGTGGCGCACGGGTGCGTAACGCGTGGGAATCTGCCCTTGGGTTCGGAATAACTTCGGGAAACTGAAGCTAATACCGGATGATGACGAAAGTCCAAAGATTTATCGCCCAGGGATGAGCCCGCGTAGGATTAGCTAGTTGGTGGGGTAAAGGCTCACCAAGGCAACGATCCTTAGCTGGTCTGAGAGGATGATCAGCCACACTGGGACTGAGACACGGCCCAGACTCCTACGGGAGGCAGCAGTAGGGAATATTGGACAATGGGCGAAAGCCTGATCCAGCAATGCCGCGTGAGTGATGAAGGCCTTAGGGTTGTAAAGCTCTTTTACCCGAGATGATAATGACAGTATCGGGAGAATAAGCTCCGGCTAACTCCGTGCCAGCAGCCGCGGTAATACGGAGGGAGCTAGCGTTGTTCGGAATTACTGGGCGTAAAGCGCACGTAGGCGGCGATTTAAGTCAGAGGTGAAAGCCCGGGGCTCAACCCCGGAACTGCCTTTGAGACTGGATTGCTAGAATCTTGGAGAGGCGAGTGGAATTCCGAGTGTAGAGGTGAAATTCGTAGATATTCGGAAGAACACCAGTGGCGAAGGCGGCTCGCTGGACAAGTATTGACGCTGAGGTGCGAAAGCGTGGGGAGCAAACAGGATTAGATACCCTGGTAGTCCACGCCGTAAACGATGATAACTAGCTGCCGGGGCACATGGTGTTTCGGTGGCGCAGCTAACGCATTAAGTTATCCGCCTGGGGAGTACGGTCGCAAGATTAAAACTCAAAGGAATTGACGGGGGCCTGCACAAGCGGTGGAGCATGTGGTTTAATTCGAAGCAACGCGCAGAACCTTACCAGCGTTTGACATCCTCATCGCGGATTTCAGAGATGATTTCCTTCAGTTCGGCTGGATGARTGACAGGTGCTGCATGGCTGTCGTCAGCTCGTGTCSTGAGATGTTGGGTTAAGTCCCGCAACGAGCGCAACCCTCGCCTTTAGTTGCCAGCATTTAGTTGGGTACTCTAAAGGAACCGCCGGTGATAAGCCGGAGGAAGGTGGGGATGACGTCAAGTCCTCATGGCCCTTACGCGCTGGGCTACACACGTGCTACAATGGCGACTACAGTGGGCTGCAACCGTGCGAGCGGTAGCTAATCTCCAAAAGTCGTCTCAGTTCGGATTGTTCTCTGCAACTCGAGAGCATGAAGGCGGAATCGCTAGTAATCGCGGATCAGCATGCCGCGGTGAATACGTTCCCAGGCCTTGTACACACCGCCCGTCACACCATGGGATTTGGATTCACCCGAAGGCA

>GW456-12-10-14-TSB2

GCAGTCGAACGAACTCTTCGGAGTTAGTGGCGGACGGGTGAGTAACACGTGGGAACGTGCCTTTAGGTTCGGAATAACTCAGGGAAACTTGTGCTAATACCGAATGTGCCCTTCGGGGGAAAGATTTATCGCCTTTAGAGCGGCCCGCGTCTGATTAGCTAGTTGGTGAGGTAAAGGCTCACCAAGGCGACGATCAGTAGCTGGTCTGAGAGGATGATCAGCCACATTGGGACTGAGACACGGCCCAAACTCCTACGGGAGGCAGCAGTGGGGAATCTTGCGCAATGGGCGAAASCCTGACGCANCCATGCCGCGTGAATGATGAAGGTNTTAGGATTGTAAAANTCTTTCACCGGGGACGATAATGACGGTACCCGGAGAAGAAGCCCCGGCTAACTTCGTGCCAGCAGCCGCGGTAATACGAAGGGGGCTAGCGTTGCTCGGAATTACTGGGCGTAAAGGGAGCGTAGGCGGACATTTAAGTCAGGGGTGAAATCCCGGGGCTCAACCTCGGAATTGCCTTTGATACTGGGTGTCTTGAGTATGAGAGAGGTGTGTGGAACTCCGAGTGTAGAGGTGAAATTCGTAGATATTCGGAAGAACACCAGTGGCGAAGGCGACACACTGGCTCATTACTGACGCTGAGGCTCGAAAGCGTGGGGAGCAAACAGGATTAGATACCCTGGTAGTCCACGCCGTAAACGATGATTGCTAGTTGTCGGGATGCATGCATTTCGGTGACGCAGCTAACGCATTAAGCAATCCGCCTGGGGAGTACGGTCGCAAGATTAAAACTCAAAGGAATTGACGGGGGCCCGCACAAGCGGTGGAGCATGTGGTTTAATTCGAAGCAACGCGCAGAACCTTACCACCTTTTGACATGCCTGGACCGCCAGAGAGATCTGGCTTTCCCTTCGGGGACTAGGACACAGGTGCTGCATGGCTGTCGTCAGCTCGTGTCGTGAGATGTTGGGTTAAGTCCCGCAACGAGCGCAACCCTCGCCATTAGTTGCCATCATTTAGTTGGGAACTCTAATGGGACTGCCGGTGCTAAGCCGGAGGAAGGTGGGGATGACGTCAAGTCCTCATGGCCCTTACAGGGTGGGCTACACACGTGCTACAATGGCGACTACAGAGGGTTAATCCTTAAAAGTCGTCTCAGTTCGGATTGTCCTCTGCAACTCGAGGGCATGAAGTTGGAATCGCTAGTAATCGCGGATCAGCATGCCGCGGTGAATACGTTCCCGGGCCTTGTACACACCGCCCGTCACACCATGGGAGTTGGTTCTACCCGAAGGCGCTGCGCTGACCGCAAGGAGGC

>GW456-12-10-14-TSB4

GTGCTGACGAGTGGCGAACGGGTGAGTAATGCATCGGAACGTGCCCAGTCGTGGGGGATAACGCAGCGAAAGCTGCGCTAATACCGCATACGATCTATGGATGAAAGCGGGGGACCGTAAGGCCTCGCGCGATTGGAGCGGCCGATGTCAGATTAGGTAGTTGGTGGGGTAAAGGCTCACCAAGCCGACGATCTGTAGCTGGTCTGAGAGGACGACCAGCCACACTGGGACTGAGACACGGCCCAGACTCCTACGGGAGGCAGCAGTGGGGAATTTTGGACAATGGGCGCAAGCCTGATCCAGCAATGCCGCGTGCAGGAAGAAGGCCTTCGGGTTGTAAACTGCTTTTGTACGGAACGAAAAGGCTCTGGTTAATACCTGGGGCTCATGACGGTACCGTAAGAATAAGCACCGGCTAACTACGTGCCAGCAGCCGCGGTAATACGTAGGGTGCAAGCGTTAATCGGAATTACTGGGCGTAAAGCGTGCGCAGGCGGTTTTGTAAGACAGGCGTGAAATCCCCGGGCTCAACCTGGGAATTGCGCTTGTGACTGCAAGGCTGGAGTGCGGCAGAGGGGGATGGAATTCCGCGTGTAGCAGTGAAATGCGTAGATATGCGGAGGAACACCGATGGCGAAGGCAATCCCCTGGGCCTGCACTGACGCTCATGCACGAAAGCGTGGGGAGCAAACAGGATTAGATACCCTGGTAGTCCACGCCCTAAACGATGTCAACTGGTTGTTGGGTCTCTTCTGACTCAGTAACGAAGCTAACGCGTGAAGTTGACCGCCTGGGGAGTACGGCCGCAAGGTTGAAACTCAAAGGAATTGACGGGGACCCGCACAAGCGGTGGATGATGTGGTTTAATTCGATGCAACGCGAAAAACCTTACCCACCTTTGACATGTACGGAATTTGCCAGAGATGGCTTAGTGCTCGAAAGAGAGCCGTAACACAGGTGCTGCATGGCTGTCGTCAGCTCGTGTCGTGAGATGTTGGGTTAAGTCCCGCAACGAGCGCAACCCTTGTCATTAGTTGCTACGAAAGGGCACTCTAATGAGACTGCCGGTGACAAACCGGAGGAAGGTGGGGATGACGTCAAGTCCTCATGGCCCTTATAGGTGGGGCTACACACGTCATACAATGGCTGGTACAAAGGGTTGCCAACCCGCGAGGGGGAGCTAATCCCATAAAGCCAGTCGTAGTCCGGATCGCAGTCTGCAACTCGACTGCGTGAAGTCGGAATCGCTAGTAATCGTGGATCAGCATGTCACGGTGAATACGTTCCCGGGTCTTGTACACACCGCCCGTCACACCATGGGAGCGGGTCTCGCCAGAAGTAGTTAGCCTAA

>GW456-12-10-14-TSB6

TACTTGTACCTGGTGGCGAGCGGCGGACGGGTGAGTAATGCCTAGGAATCTGCCTGGTAGTGGGGGATAACGCTCGGAAACGGACGCTAATACCGCATACGTCCTACGGGAGAAAGCAGGGGACCTTCGGGCCTTGCGCTATCAGATGAGCCTAGGTCGGATTAGCTAGTTGGTGAGGTAATGGCTCACCAAGGCGACGATCCGTAACTGGTCTGAGAGGATGATCAGTCACACTGGAACTGAGACACGGTCCAGACTCCTACGGGAGGCAGCAGTGGGGAATATTGGACAATGGGCGAAAGCCTGATCCAGCCATGCCGCGTGTGTGAAGAAGGTCTTCGGATTGTAAAGCACTTTAAGTTGGGAGGAAGGGCAGTTACCTAATACGTATCTGTTTTGACGTTACCGACAGAATAAGCACCGGCTAACTCTGTGCCAGCAGCCGCGGTAATACAGAGGGTGCAAGCGTTAATCGGAATTACTGGGCGTAAAGCGCGCGTAGGTGGTTTGTTAAGTTGGATGTGAAATCCCCGGGCTCAACCTGGGAACTGCATTCAAAACTGACAAGCTAGAGTATGGTAGAGGGTGGTGGAATTTCCTGTGTAGCGGTGAAATGCGTAGATATAGGAAGGAACACCAGTGGCGAAGGCGACCACCTGGACTGATACTGACACTGAGGTGCGAAAGCGTGGGGAGCAAACAGGATTAGATACCCTGGTAGTCCACGCCGTAAACGATGTCAACTAGCCGTTGGGAGCCTTGAGCTCTTAGTGGCGCAGCTAACGCATTAAGTTGACCGCCTGGGGAGTACGGCCGCAAGGTTAAAACTCAAATGAATTGACGGGGGCCCGCACAAGCGGTGGAGCATGTGGTTTAATTCGAAGCAACGCGAAGAACCTTACCAGGCCTTGACATCCAATGAACTTTCCAGAGATGGATTGGTGCCTTCGGGAACATTGAGACAGGTGCTGCATGGCTGTCGTCAGCTCGTGTCGTGAGATGTTGGGTTAAGTCCCGTAACGAGCGCAACCCTTGTCCTTAGTTACCAGCACGTNATGGTGGGCACTCTAAGGAGACTGCCGGTGACAAACCGGAGGAAGGTGGGGATGACGTCAAGTCATCATGGCCCTTACGGCCTGGGCTACACACGTGCTACAATGGTCGGTACAGAGGGTTGCCAAGCCGCGAGGTGGAGCTAATCCCAGAAAACCGATCGTAGTCCGGATCGCAGTCTGCAACTCGACTGCGTGAAGTCGGAATCGCTAGTAATCGCGAATCAGAATGTCGCGGTGAATACGTTCCCGGGCCTTGTACACACCGCCCGTCACACCATGGGAGTGGGTTGCACCAGAAGTAGCTAG

>GW456-12-10-14-TSB7

CTCTTCGGAGTTAGTGGCGCACGGGTGCGTAACGCGTGGGAATCTGCCCTTGGGTACGGAATAACTCAGAGAAATTTGTGCTAATACCGTATAATGTCTTCGGACCAAAGATTTATCGCCCAAGGATGAGCCCGCGTAAGATTAGCTAGTTGGTGAGGTAAAAGCTCACCAAGGCGACGATCTTTAGCTGGTCTGAGAGGATGATCAGCCACACTGGGACTGAGACACGGCCCAGACTCCTACGGGAGGCAGCAGTGGGGAATATTGGACAATGGGCGAAAGCCTGATCCAGCAATGCCGCGTGAGTGATGAAGGCCTTAGGGTTGTAAAGCTCTTTTACCCGGGATGATAATGACAGTACCGGGAGAATAAGCTCCGGCTAACTCCGTGCCAGCAGCCGCGGTAATACGGAGGGAGCTAGCGTTGTTCGGAATTACTGGGCGTAAAGCGCGCGTAGGCGGTTTTTTAAGTCAGAGGTGAAAGCCCGGGGCTCAACCCCGGAATAGCCTTTGAAACTGGAAAACTTGAATCTTGGAGAGGTCAGTGGAATTCCGAGTGTAGAGGTGAAATTCGTAGATATTCGGAAGAACACCAGTGGCGAAGGCGACTGACTGGACAAGTATTGACGCTGAGGTGCGAAAGCGTGGGGAGCAAACAGGATTAGATACCCTGGTAGTCCACGCCGTAAACGATGATAACTATCTGTCCGGGCTCATAGAGCTTGGGTGGAGCAGCTAACGCATTAAGTTATCCGCCTGGGGAGTACGGTCGCAAGATTAAAACTCAAAGGAATTGACGGGGGCCTGCACAAGCGGTGGAGCATGTGGTTTAATTCGAAGCAACGCGCAGAACCTTACCAGCGTTTGACATCCTGATCGCGGATTAGAGAGATCTTTTCCTTCAGTTCGGCTGGATCAGTGACAGGTGCTGCATGGCTGTCGTCAGCTCGTGTCGTGAGATGTTGGGTTAAGTCCCGCAACGAGCGCAACCCTCATCCCTAGTTGCCATCATTTAGTTGGGCACTCTATGGAAACTGCCGGTGATAAGCCGGAGGAAGGTGGGGATGACGTCAAGTCCTCATGGCCCTTACACGCTGGGCTACACACGTGCTACAATGGCAACTACAGTGGGCAGCAACCTCGCGAGGGGTAGCTAATCTCCAAAAGTTGTCTCAGTTCGGATTGTTCTCTGCAACTCGAGAGCATGAAGGCGGAATCGCTAGTAATCGCGGATCAGCATGCCGCGGTGAATACGTTCCCAGGCCTTGTACACACCGCCCGTCACACCATGGGAGTTGGTTTCACCCGAAGGCAGTGCTCTAA

>GW456-12-10-14-TSB8

TCTAGTGGCGCACGGGTGCGTAACGCGTGGGAATCTGCCCTTGGGTTCGGAATAACTTCGGGAAACTGAAGCTAATACCGGATGATGACGAAAGTCCAAAGATTTATCGCCCAGGGATGAGCCCGCGTAGGATTAGCTAGTTGGTGGGGTAAAGGCTCACCAAGGCAACGATCCTTAGCTGGTCTGAGAGGATGATCAGCCACACTGGGACTGAGACACGGCCCAGACTCCTACGGGAGGCAGCAGTAGGGAATATTGGACAATGGGCGAAAGCCTGATCCAGCAATGCCGCGTGAGTGATGAAGGCCTTAGGGTTGTAAAGCTCTTTTACCCGAGATGATAATGACAGTATCGGGAGAATAAGCTCCGGCTAACTCCGTGCCAGCAGCCGCGGTAATACGGAGGGAGCTAGCGTTGTTCGGAATTACTGGGCGTAAAGCGCACGTAGGCGGCGATTTAAGTCAGAGGTGAAAGCCCGGGGCTCAACCCCGGAACTGCCTTTGAGACTGGATTGCTAGAATCTTGGAGAGGCGAGTGGAATTCCGAGTGTAGAGGTGAAATTCGTAGATATTCGGAAGAACACCAGTGGCGAAGGCGGCTCGCTGGACAAGTATTGACGCTGAGGTGCGAAAGCGTGGGGAGCAAACAGGATTAGATACCCTGGTAGTCCACGCCGTAAACGATGATAACTAGCTGCCGGGGCACATGGTGTTTCGGTGGCGCAGCTAACGCATTAAGTTATCCGCCTGGGGAGTACGGTCGCAAGATTAAAACTCAAAGGAATTGACGGGGGCCTGCACAAGCGGTGGAGCATGTGGTTTAATTCGAAGCAACGCGCAGAACCTTACCAGCGTTTGACATCCTCATCGCGGATTTCAGAGATGATTTCCTTCAGTTCGGCTGGATGAGTGACAGGTGCTGCATGGCTGTCGTCAGCTCGTGTCGTGAGATGTTGGGTTAAGTCCCGCAACGAGCGCAACCCTCGCCTTTAGTTGCCAGCATTTAGTTGGGTACTCTAAAGGAACCGCCGGTGATAAGCCGGAGGAAGGTGGGGATGACGTCAAGTCCTCATGGCCCTTACGCGCTGGGCTACACACGTGCTACAATGGCGACTACAGTGGGCTGCAACCGTGCGAGCGGTAGCTAATCTCCAAAAGTCGTCTCAGTTCGGATTGTTCTCTGCAACTCGAGAGCATGAAGGCGGAATCGCTAGTAATCGCGGATCAGCATGCCGCGGTGAATACGTTCCCAGGCCTTGTACACACCGCCCGTCACACCATGGGATTTGGATTCACCCGAAGGCACTGCG

>GW458-11-11-14-LB1

GGGTACTTGTACCTGGTGGCGAGCGGCGGACGGGTGAGTAATGCCTAGGAATCTGCCTGGTAGTGGGGGATAACGCTCGGAAACGGACGCTAATACCGCATACGTCCTACGGGAGAAAGCAGGGGACCTTCGGGCCTTGCGCTATCAGATGAGCCTAGGTCGGATTAGCTAGTTGGTGAGGTAATGGCTCACCAAGGCGACGATCCGTAACTGGTCTGAGAGGATGATCAGTCACACTGGAACTGAGACACGGTCCAGACTCCTACGGGAGGCAGCAGTGGGGAATATTGGACAATGGGCGAAAGCCTGATCCAGCCATGCCGCGTGTGTGAAGAAGGTCTTCGGATTGTAAAGCACTTTAAGTTGGGAGGAAGGGCAGTTACCTAATACGTATCTGTTTTGACGTTACCGACAGAATAAGCACCGGCTAACTCTGTGCCAGCAGCCGCGGTAATACAGAGGGTGCAAGCGTTAATCGGAATTACTGGGCGTAAAGCGCGCGTAGGTGGTTTGTTAAGTTGGATGTGAAATCCCCGGGCTCAACCTGGGAACTGCATTCAAAACTGACAAGCTAGAGTATGGTAGAGGGTGGTGGAATTTCCTGTGTAGCGGTGAAATGCGTAGATATAGGAAGGAACACCAGTGGCGAAGGCGACCACCTGGACTGATACTGACACTGAGGTGCGAAAGCGTGGGGAGCAAACAGGATTAGATACCCTGGTAGTCCACGCCGTAAACGATGTCAACTAGCCGTTGGGAGCCTTGAGCTCTTAGTGGCGCAGCTAACGCATTAAGTTGACCGCCTGGGGAGTACGGCCGCAAGGTTAAAACTCAAATGAATTGACGGGGGCCCGCACAAGCGGTGGAGCATGTGGTTTAATTCGAAGCAACGCGAAGAACCTTACCAGGCCTTGACATCCAATGAACTTTCCAGAGATGGATTGGTGCCTTCGGGAACATTGAGACAGGTGCTGCATGGCTGTCGTCAGCTCGTGTCGTGAGATGTTGGGTTAAGTCCCGTAACGAGCGCAACCCTTGTCCTTAGTTACCAGCACGTNNTGGTGGGCACTCTAAGGAGACTGCCGGTGACAAACCGGAGGAAGGTGGGGATGACGTCAAGTCATCATGGCCCTTACGGCCTGGGCTACACACGTGCTACAATGGTCGGTACAGAGGGTTGCCAAGCCGCGAGGTGGAGCTAATCCCAGAAAACCGATCGTAGTCCGGATCGCAGTCTGCAACTCGACTGCGTGAAGTCGGAATCGCTAGTAATCGCGAATCAGAATGTCGCGGTGAATACGTTCCCGGGCCTTGTACACACCGCCCGTCACACCATGGGAGTGGGTTGCACCAGAAGTAGCTAGTC

>GW458-11-11-14-LB2

GGCAGCGCGGGAGCAATCCTGGCGGCGAGTGGCGAACGGGTGAGTAATACATCGGAACGTGCCCAATCGTGGGGGATAACGCAGCGAAAGCTGTGCTAATACCGCATACGATCTACGGATGAAAGCAGGGGATCGCAAGACCTTGCGCGAATGGAGCGGCCGATGGCAGATTAGGTAGTTGGTGGGGTAAAGGCTCACCAAGCCTTCGATCTGTAGCTGGTCTGAGAGGACGACCAGCCACACTGGGACTGAGACACGGCCCAGACTCCTACGGGAGGCAGCAGTGGGGAATTTTGGACAATGGGCGAAAGCCTGATCCAGCCATGCCGCGTGCAGGATGAAGGCCTTCGGGTTGTAAACTGCTTTTGTACGGAACGAAACGGTCTTTTCTAATACAGAAGGCTAATGACGGTACCGTAAGAATAAGCACCGGCTAACTACGTGCCAGCAGCCGCGGTAATACGTAGGGTGCAAGCGTTAATCGGAATTACTGGGCGTAAAGCGTGCGCAGGCGGTTATGTAAGACAGTTGTGAAATCCCCGGGCTCAACCTGGGAACTGCATCTGTGACTGCATAGCTAGAGTACGGTAGAGGGGGATGGAATTCCGCGTGTAGCAGTGAAATGCGTAGATATGCGGAGGAACACCGATGGCGAAGGCAATCCCCTGGACCTGTACTGACGCTCATGCACGAAAGCGTGGGGAGCAAACAGGATTAGATACCCTGGTAGTCCACGCCCTAAACGATGTCAACTGGTTGTTGGGTCTTCACTGACTCAGTAACGAAGCTAACGCGTGAAGTTGACCGCCTGGGGAGTACGGCCGCAAGGTTGAAACTCAAAGGAATTGACGGGGACCCGCACAAGCGGTGGATGATGTGGTTTAATTCGATGCAACGCGAAAAACCTTACCCACCTTTGACATGTACGGAATTCGCCAGAGATGGCTTAGTGCTCGAAAGAGAACCGTAACACAGGTGCTGCATGGCTGTCGTCAGCTCGTGTCGTGAGATGTTGGGTTAAGTCCCGCAACGAGCGCAACCCTTGTCATTAGTTGCTACATTCAGTTGGGCACTCTAATGAGACTGCCGGTGACAAACCGGAGGAAGGTGGGGATGACGTCAAGTCCTCATGGCCCTTATAGGTGGGGCTACACACGTCATACAATGGCTGGTACAAAGGGTTGCCAACCCGCGAGGGGGAGCTAATCCCATAAAACCAGTCGTAGTCCGGATCGCAGTCTGCAACTCGACTGCGTGAAGTCGGAATCGCTAGTAATCGTGGATCAGAATGTCACGGTGAATACGTTCCCGGGTCTTGTACACACCGCCCGTCACACCATGGGAGCGGGTTCTGCCAGAAGTAGTT

>GW458-11-11-14-TSB1

TGCAGTCGAGCGGATGACAGGAGCTTGCTCCTGAATTCAGCGGCGGACGGGTGAGTAATGCCTAGGAATCTGCCTGGTAGTGGGGGACAACGTTTCGAAAGGAACGCTAATACCGCATACGTCCTACGGGAGAAAGCAGGGGACCTTCGGGCCTTGCGCTATCAGATGAGCCTAGGTCGGATTAGCTAGTTGGTGAGGTAATGGCTCACCAAGGCGACGATCCGTAACTGGTCTGAGAGGATGATCAGTCACACTGGAACTGAGACACGGTCCAGACTCCTACGGGAGGCAGCAGTGGGGAATATTGGACAATGGGCGAAAGCCTGATCCAGCCATGCCGCGTGTGTGAAGAAGGTCTTCGGATTGTAAAGCACTTTAAGTTGGGAGGAAGGGCATTAACCTAATACGTTAGTGTTTTGACGTTACCGACAGAATAAGCACCGGCTAACTCTGTGCCAGCAGCCGCGGTAATACAGAGGGTGCAAGCGTTAATCGGAATTACTGGGCGTAAAGCGCGCGTAGGTGGTTTGTTAAGTTGGATGTGAAAGCCCCGGGCTCAACCTGGGAACTGCATTCAAAACTGACAAGCTAGAGTATGGTAGAGGGTGGTGGAATTTCCTGTGTAGCGGTGAAATGCGTAGATATAGGAAGGAACACCAGTGGCGAAGGCGACCACCTGGACTGATACTGACACTGAGGTGCGAAAGCGTGGGGAGCAAACAGGATTAGATACCCTGGTAGTCCACGCCGTAAACGATGTCAACTAGCCGTTGGGAGCCTTGAGCTCTTAGTGGCGCAGCTAACGCATTAAGTTGACCGCCTGGGGAGTACGGCCGCAAGGTTAAAACTCAAATGAATTGACGGGGGCCCGCACAAGCGGTGGAGCATGTGGTTTAATTCGAAGCAACGCGAAGAACCTTACCAGGCCTTGACATCCAATGAACTTTCCAGAGATGGATTGGTGCCTTCGGGAACATTGAGACAGGTGCTGCATGGCTGTCGTCAGCTCGTGTCGTGAGATGTTGGGTTAAGTCCCGTAACGAGCGCAACCCTTGTCCTTAGTTACCAGCACGTCATGGTGGGCACTCTAAGGAGACTGCCGGTGACAAACCGGAGGAAGGTGGGGATGACGTCAAGTCATCATGGCCCTTACGGCCTGGGCTACACACGTGCTACAATGGTCGGTACAGAGGGTTGCCAAGCCGCGAGGTGGAGCTAATCCCACAAAACCGATCGTAGTCCGGATCGCAGTCTGCAACTCGACTGCGTGAAGTCGGAATCGCTAGTAATCGCGAATCAGAATGTCGCGGTGAATACGTTCCCGGGCCTTGTACACACCGCCCGTCACACCATGGGAGTGGGTTGCACCAGAAGTAGCTA

>GW458-11-11-14-TSB2

TGCAGTCGAACGGCAGCGCGGGAGCAATCCTGGCGGCGNAGTGGCGAACGGGTGAGTAATACATCGGAACGTGCCCAATCGTGGGGGATAACGCAGCGAAAGCTGTGCTAATACCGCATACGATCTACGGATGAAAGCAGGGGATCGCAAGACCTTGCGCGAATGGAGCGGCCGATGGCAGATTAGGTAGTTGGTGGGGTAAAGGCTCACCAAGCCTTCGATCTGTAGCTGGTCTGAGAGGACGACCAGCCACACTGGGACTGAGACACGGCCCAGACTCCTACGGGAGGCAGCAGTGGGGAATTTTGGACAATGGGCGAAAGCCTGATCCAGCCATGCCGCGTGCAGGATGAAGGCCTTCGGGTTGTAAACTGCTTTTGTACGGAACGAAACGGTCTTTTCTAATACAGAAGGCTAATGACGGTACCGTAAGAATAAGCACCGGCTAACTACGTGCCAGCAGCCGCGGTAATACGTAGGGTGCAAGCGTTAATCGGAATTACTGGGCGTAAAGCGTGCGCAGGCGGTTATGTAAGACAGTTGTGAAATCCCCGGGCTCAACCTGGGAACTGCATCTGTGACTGCATAGCTAGAGTACGGTAGAGGGGGATGGAATTCCGCGTGTAGCAGTGAAATGCGTAGATATGCGGAGGAACACCGATGGCGAAGGCAATCCCCTGGACCTGTACTGACGCTCATGCACGAAAGCGTGGGGAGCAAACAGGATTAGATACCCTGGTAGTCCACGCCCTAAACGATGTCAACTGGTTGTTGGGTCTTCACTGACTCAGTAACGAAGCTAACGCGTGAAGTTGACCGCCTGGGGAGTACGGCCGCAAGGTTGAAACTCAAAGGAATTGACGGGGACCCGCACAAGCGGTGGATGATGTGGTTTAATTCGATGCAACGCGAAAAACCTTACCCACCNTTTGACATGTACGGAATTCGCCAGAGATGGCTTAGTGCTCGAAAGAGAACCGTAACACAGGTGCTGCATGGCTGTCGTCAGCTCGTGTCGTGAGATGTTGGGTTAAGTCCCGCAACGAGCGCAACCCTTGTCATTAGTTGCTACATTCAGTTGGGCACTCTAATGAGACTGCCGGTGACAAACCGGAGGAAGGTGGGGATGACGTCAAGTCCTCATGGCCCTTATAGGTGGGGCTACACACGTCATACAATGGCTGGTACAAAGGGTTGCCAACCCGCGAGGGGGAGCTAATCCCATAAAACCAGTCGTAGTCCGGATCGCAGTCTGCAACTCGACTGCGTGAAGTCGGAATCGCTAGTAATCGTGGATCAGAATGTCACGGTGAATACGTTCCCGGGTCTTGTACACACCGCCCGTCACACCATGGGAGCGGGTTCTGCCAGAAGTAGTTAGCTTAACCGCAA

>GW458-11-11-14-TSB3

GGAGCTTGCTCTGGTGGCGAGTGGCGAACGGGTGAGTAATATATCGGAACGTACCCTGGAGTGGGGGATAACGTAGCGAAAGTTACGCTAATACCGCATACGATCTACGGATGAAAGTGGGGGATCGCAAGACCTCATGCTCGTGGAGCGGCCGATATCTGATTAGCTAGTTGGTAGGGTAAAAGCCTACCAAGGCATCGATCAGTAGCTGGTCTGAGAGGACGACCAGCCACACTGGAACTGAGACACGGTCCAGACTCCTACGGGAGGCAGCAGTGGGGAATTTTGGACAATGGGCGAAAGCCTGATCCAGCAATGCCGCGTGAGTGAAGAAGGCCTTCGGGTTGTAAAGCTCTTTTGTCAGGGAAGAAACGGTGAGGGCTAATATCTCTTGCTAATGACGGTACCTGAAGAATAAGCACCGGCTAACTACGTGCCAGCAGCCGCGGTAATACGTAGGGTGCAAGCGTTAATCGGAATTACTGGGCGTAAAGCGTGCGCAGGCGGTTTTGTAAGTCTGATGTGAAATCCCCGGGCTCAACCTGGGAATTGCATTGGAGACTGCAAGGCTAGAATCTGGCAGAGGGGGGTAGAATTCCACGTGTAGCAGTGAAATGCGTAGATATGTGGAGGAACACCGATGGCGAAGGCAGCCCCCTGGGTCAAGATTGACGCTCATGCACGAAAGCGTGGGGAGCAAACAGGATTAGATACCCTGGTAGTCCACGCCCTAAACGATGTCTACTAGTTGTCGGGTCTTAATTGACTTGGTAACGCAGCTAACGCGTGAAGTAGACCGCCTGGGGAGTACGGTCGCAAGATTAAAACTCAAAGGAATTGACGGGGACCCGCACAAGCGGTGGATGATGTGGATTAATTCGATGCAACGCGAAAAACCTTACCTACCCTTGACATGGCTGGAATCCCCGAGAGATTGGGGAGTGCTCGAAAGAGAACCAGTACACAGGTGCTGCATGGCTGTCGTCAGCTCGTGTCGTGAGATGTTGGGTTAAGTCCCGCAACGAGCGCAACCCTTGTCATTAGTTGCTACGAAAGGGCACTCTAATGAGACTGCCGGTGACAAACCGGAGGAAGGTGGGGATGACGTCAAGTCCTCATGGCCCTTATGGGTAGGGCTTCACACGTCATACAATGGTACATACAGAGCGCCGCCAACCCGCGAGGGGGAGCTAATCGCAGAAAGTGTATCGTAGTCCGGATTGTAGTCTGCAACTCGACTGCATGAAGTTGGAATCGCTAGTAATCGCGGATCAGCATGTCGCGGTGAATACGTTCCCGGGTCTTGTACACACCGCCCGTCACACCATGGGAGCGGG

>GW458-11-17-14-LB1

TCGAGCGGCAGCACGGGTACTTGTACCTGGTGGCGAGCGGCGGACGGGTGAGTAATGCCTAGGAATCTGCCTGGTAGTGGGGGATAACGCTCGGAAACGGACGCTAATACCGCATACGTCCTACGGGAGAAAGCAGGGGACCTTCGGGCCTTGCGCTATCAGATGAGCCTAGGTCGGATTAGCTAGTTGGTGAGGTAATGGCTCACCAAGGCGACGATCCGTAACTGGTCTGAGAGGATGATCAGTCACACTGGAACTGAGACACGGTCCAGACTCCTACGGGAGGCAGCAGTGGGGAATATTGGACAATGGGCGAAAGCCTGATCCAGCCATGCCGCGTGTGTGAAGAAGGTCTTCGGATTGTAAAGCACTTTAAGTTGGGAGGAAGGGCAGTTACCTAATACGTATCTGTTTTGACGTTACCGACAGAATAAGCACCGGCTAACTCTGTGCCAGCAGCCGCGGTAATACAGAGGGTGCAAGCGTTAATCGGAATTACTGGGCGTAAAGCGCGCGTAGGTGGTTTGTTAAGTTGGATGTGAAATCCCCGGGCTCAACCTGGGAACTGCATTCAAAACTGACAAGCTAGAGTATGGTAGAGGGTGGTGGAATTTCCTGTGTAGCGGTGAAATGCGTAGATATAGGAAGGAACACCAGTGGCGAAGGCGACCACCTGGACTGATACTGACACTGAGGTGCGAAAGCGTGGGGAGCAAACAGGATTAGATACCCTGGTAGTCCACGCCGTAAACGATGTCAACTAGCCGTTGGGAGCCTTGAGCTCTTAGTGGCGCAGCTAACGCATTAAGTTGACCGCCTGGGGAGTACGGCCGCAAGGTTAAAACTCAAATGAATTGACGGGGGCCCGCACAAGCGGTGGAGCATGTGGTTTAATTCGAAGCAACGCGAAGAACCTTACCAGGCCTTGACATCCAATGAACTTTCCAGAGATGGATTGGTGCCTTCGGGAACATTGAGACAGGTGCTGCATGGCTGTCGTCAGCTCGTGTCGTGAGATGTTGGGTTAAGTCCCGTAACGAGCGCAACCYTTGTCCTTAGTTACCAGCACGTCATGGTGGGCACTCTAAGGAGACTGCCGGTGACAAACCGGAGGAAGGTGGGGATGACGTCAAGTCATCATGGCCCTTACGGCCTGGGCTACACACGTGCTACAATGGTCGGTACAGAGGGTTGCCAAGCCGCGAGGTGGAGCTAATCCCAGAAAACCGATCGTAGTCCGGATCGCAGTCTGCAACTCGACTGCGTGAAGTCGGAATCGCTAGTAATCGCGAATCAGAATGTCGCGGTGAATACGTTCCCGGGCCTTGTACACACCGCCCGTCACACCATGGGAGTGGG

>GW458-11-17-14-LB2

CAGTCGAGCGGCAGCACGGGTACTTGTACCTGGTGGCGAGCGGCGGACGGGTGAGTAATGCCTAGGAATCTGCCTGGTAGTGGGGGATAACGCTCGGAAACGGACGCTAATACCGCATACGTCCTACGGGAGAAAGCAGGGGACCTTCGGGCCTTGCGCTATCAGATGAGCCTAGGTCGGATTAGCTAGTTGGTGAGGTAATGGCTCACCAAGGCGACGATCCGTAACTGGTCTGAGAGGATGATCAGTCACACTGGAACTGAGACACGGTCCAGACTCCTACGGGAGGCAGCAGTGGGGAATATTGGACAATGGGCGAAAGCCTGATCCAGCCATGCCGCGTGTGTGAAGAAGGTCTTCGGATTGTAAAGCACTTTAAGTTGGGAGGAAGGGCAGTTACCTAATACGTATCTGTTTTGACGTTACCGACAGAATAAGCACCGGCTAACTCTGTGCCAGCAGCCGCGGTAATACAGAGGGTGCAAGCGTTAATCGGAATTACTGGGCGTAAAGCGCGCGTAGGTGGTTTGTTAAGTTGGATGTGAAATCCCCGGGCTCAACCTGGGAACTGCATTCAAAACTGACAAGCTAGAGTATGGTAGAGGGTGGTGGAATTTCCTGTGTAGCGGTGAAATGCGTAGATATAGGAAGGAACACCAGTGGCGAAGGCGACCACCTGGACTGATACTGACACTGAGGTGCGAAAGCGTGGGGAGCAAACAGGATTAGATACCCTGGTAGTCCACGCCGTAAACGATGTCAACTAGCCGTTGGGAGCCTTGAGCTCTTAGTGGCGCAGCTAACGCATTAAGTTGACCGCCTGGGGAGTACGGCCGCAAGGTTAAAACTCAAATGAATTGACGGGGGCCCGCACAAGCGGTGGAGCATGTGGTTTAATTCGAAGCAACGCGAAGAACCTTACCAGGCCTTGACATCCAATGAACTTTCCAGAGATGGATTGGTGCCTTCGGGAACATTGAGACAGGTGCTGCATGGCTGTCGTCAGCTCGTGTCGTGAGATGTTGGGTTAAGTCCCGTAACGAGCGCAACCCTTGTCCTTAGTTACCAGCACGTCATGGTGGGCACTCTAAGGAGACTGCCGGTGACAAACCGGAGGAAGGTGGGGATGACGTCAAGTCATCATGGCCCTTACGGCCTGGGCTACACACGTGCTACAATGGTCGGTACAGAGGGTTGCCAAGCCGCGAGGTGGAGCTAATCCCAGAAAACCGATCGTAGTCCGGATCGCAGTCTGCAACTCGACTGCGTGAAGTCGGAATCGCTAGTAATCGCGAATCAGAATGTCGCGGTGAATACGTTCCCGGGCCTTGTACACACCGCCCGTCACACCATGGGAGTGGGTTGCACCAGAAGTAGCTAGTCTA

>GW458-11-17-14-R2A1

CGGCAGCGCGGGAGCAATCCTGGCGGCGAGTGGCGAACGGGTGAGTAATACATCGGAACGTGCCCAATCGTGGGGGATAACGCAGCGAAAGCTGTGCTAATACCGCATACGATCTACGGATGAAAGCAGGGGATCGCAAGACCTTGCGCGAATGGAGCGGCCGATGGCAGATTAGGTAGTTGGTGGGGTAAAGGCTCACCAAGCCTTCGATCTGTAGCTGGTCTGAGAGGACGACCAGCCACACTGGGACTGAGACACGGCCCAGACTCCTACGGGAGGCAGCAGTGGGGAATTTTGGACAATGGGCGAAAGCCTGATCCAGCCATGCCGCGTGCAGGATGAAGGCCTTCGGGTTGTAAACTGCTTTTGTACGGAACGAAACGGTCTTTTCTAATACAGAAGGCTAATGACGGTACCGTAAGAATAAGCACCGGCTAAYTACGTGCCAGCAGCCGCGGTAATACGTAGGGTGCAAGCGTTAATCGGAATTACTGGGCGTAAAGCGTGCGCAGGCGGTTATGTAAGACAGTTGTGAAATCCCCGGGCTCAACCTGGGAACTGCATCTGTGACTGCATAGCTAGAGTACGGTAGAGGGGGATGGAATTCCGCGTGTAGCAGTGAAATGCGTAGATATGCGGAGGAACACCGATGGCGAAGGCAATCCCCTGGACCTGTACTGACGCTCATGCACGAAAGCGTGGGGAGCAAACAGGATTAGATACCCTGGTAGTCCACGCCCTAAACGATGTCAACTGGTTGTTGGGTCTTCACTGACTCAGTAACGAAGCTAACGCGTGAAGTTGACCGCCTGGGGAGTACGGCCGCAAGGTTGAAACTCAAAGGAATTGACGGGGACCCGCACAAGCGGTGGATGATGKGGTTTAATTCGATGCAACGCGAAAAACCTTACCCACCTTTGACATGTACGGAATTCGCCAGAGATGGCTTAGTGCTCGAAAGAGAACCGTAACACAGGTGCTGCATGGCTGTCGTCAGCTCGTGTCGTGAGATGTTGGGTTAAGTCCCGCAACGAGCGCAACCCTTGTCATTAGTTGCTACATTCAGTTGGGCACTCTAATGAGACTGCCGGTGACAAACCGGAGGAAGGTGGGGATGACGTCAAGTCCTCATGGCCCTTATAGGTGGGGCTACACACGTCATACAATGGCTGGTACAAAGGGTTGCCAACCCGCGAGGGGGAGCTAATCCCATAAAACCAGTCGTAGTCCGGATCGCAGTCTGCAACTCGACTGCGTGAAGTCGGAATCGCTAGTAATCGTGGATCAGAATGTCACGGTGAATACGTTCCCGGGTCTTGTACACACCGCCCGTCACACCATGGGAGCGGGTTCTGCCAGAAGTAGTTAGCTTAACCGCAAGGAGGG

>GW458-11-17-14-TSB1

CGAGCGGCAGCACGGGTACTTGTACCTGGTGGCGAGCGGCGGACGGGTGAGTAATGCCTAGGAATCTGCCTGGTAGTGGGGGATAACGCTCGGAAACGGACGCTAATACCGCATACGTCCTACGGGAGAAAGCAGGGGACCTTCGGGCCTTGCGCTATCAGATGAGCCTAGGTCGGATTAGCTAGTTGGTGAGGTAATGGCTCACCAAGGCGACGATCCGTAACTGGTCTGAGAGGATGATCAGTCACACTGGAACTGAGACACGGTCCAGACTCCTACGGGAGGCAGCAGTGGGGAATATTGGACAATGGGCGAAAGCCTGATCCAGCCATGCCGCGTGTGTGAAGAAGGTCTTCGGATTGTAAAGCACTTTAAGTTGGGAGGAAGGGCAGTTACYTAATACGTATCTGTTTTGACGTTACCGACAGAATAAGCACCGGCTAACTCTGTGCCAGCAGCCGCGGTAATACAGAGGGTGCAAGCGTTAATCGGAATTACTGGGCGTAAAGCGCGCGTAGGTGGTTTGTTAAGTTGGATGTGAAATCCCCGGGCTCAACCTGGGAACTGCATTCAAAACTGACAAGCTAGAGTATGGTAGAGGGTGGTGGAATTTCCTGTGTAGCGGTGAAATGCGTAGATATAGGAAGGAACACCAGTGGCGAAGGCGACCACCTGGACTGATACTGACACTGAGGTGCGAAAGCGTGGGGAGCAAACAGGATTAGATACCCTGGTAGTCCACGCCGTAAACGATGTCAACTAGCCGTTGGGAGCCTTGAGCTCTTAGTGGCGCAGCTAACGCATTAAGTTGACCGCCTGGGGAGTACGGCCGCAAGGTTAAAACTCAAATGAATTGACGGGGGCCCGCACAAGCGGTGGAGCATGTGGTTTAATTCGAAGCAACGCGAARAACCTTACCAGGCCTTGACATCCAATGAACTTTCCAGAGATGGATTGGTGCCTTCGGGAACATTGARACAGGTGCTGCATGGCTGTCSTCAGCTCSTGTCGTGAGATGTTGGGTTAAGTCCCGTAACGAGCGCAACCCTTGTCCTTAGTTACCAGCACGTCATGGTGGGCACTCTAAGGAGACTGCCGGTGACAAACCGGAGGAAGGTGGGGATGACGTCAAGTCATCATGGCCCTTACGGCCTGGGCTACACACGTGCTACAATGGTCGGTACAGAGGGTTGCCAAGCCGCGAGGTGGAGCTAATCCCAGAAAACCGATCGTAGTCCGGATCGCAGTCTGCAACTCGACTGCGTGAAGTCGGAATCGCTAGTAATCGCGAATCAGAATGTCGCGGTGAATACGTTCCCGGGCCTTGTACACACCGCCCGTCACACCATGGGAGTGGGTTGCACCAGAAGTAGCTAGTCTA

>GW458-11-17-14-TSB2

TCGAACGGCAGCGCGGGAGCAATCCTGGCGGCGAGTGGCGAACGGGTGAGTAATACATCGGAACGTGCCCAATCGTGGGGGATAACGCAGCGAAAGCTGTGCTAATACCGCATACGATCTACGGATGAAAGCAGGGGATCGCAAGACCTTGCGCGAATGGAGCGGCCGATGGCAGATTAGGTAGTTGGTGGGGTAAAGGCTCACCAAGCCTTCGATCTGTAGCTGGTCTGAGAGGACGACCAGCCACACTGGGACTGAGACACGGCCCAGACTCCTACGGGAGGCAGCAGTGGGGAATTTTGGACAATGGGCGAAAGCCTGATCCAGCCATGCCGCGTGCAGGATGAAGGCCTTCGGGTTGTAAACTGCTTTTGTACGGAACGAAACGGTCTTTTCTAATACAGAAGGCTAATGACGGTACCGTAAGAATAAGCACCGGCTAACTACGTGCCAGCAGCCGCGGTAATACGTAGGGTGCAAGCGTTAATCGGAATTACTGGGCGTAAAGCGTGCGCAGGCGGTTATGTAAGACAGTTGTGAAATCCCCGGGCTCAACCTGGGAACTGCATCTGTGACTGCATAGCTAGAGTACGGTAGAGGGGGATGGAATTCCGCGTGTAGCAGTGAAATGCGTAGATATGCGGAGGAACACCGATGGCGAAGGCAATCCCCTGGACCTGTACTGACGCTCATGCACGAAAGCGTGGGGAGCAAACAGGATTAGATACCCTGGTAGTCCACGCCCTAAACGATGTCAACTGGTTGTTGGGTCTTCACTGACTCAGTAACGAAGCTAACGCGTGAAGTTGACCGCCTGGGGAGTACGGCCGCAAGGTTGAAACTCAAAGGAATTGACGGGGACCCGCACAAGCGGTGGATGATGTGGTTTAATTCGATGCAACGCGAAAAACCTTACCCACYTTTGACATGTACGGAATTCGCCAGAGATGGCTTAKTGCTCGAAAGAGAACCGTAACACAGGTGCTGCATGGCTGTCGTCAGCTCGTGTCGTGAGATGTTGGGTTAAGTCCCGCAACGAGCGCAACCCTTGTCATTAGTTGCTACATTCAGTTGGGCACTCTAATGAGACTGCCGGTGACAAACCGGAGGAAGGTGGGGATGACGTCAAGTCCTCATGGCCCTTATAGGTGGGGCTACACACGTCATACAATGGCTGGTACAAAGGGTTGCCAACCCGCGAGGGGGAGCTAATCCCATAAAACCAGTCGTAGTCCGGATCGCAGTCTGCAACTCGACTGCGTGAAGTCGGAATCGCTAGTAATCGTGGATCAGAATGTCACGGTGAATACGTTCCCGGGTCTTGTACACACCGCCCGTCACACCATGGGAGCGGGTTCTGCCAGAAGTAGTTAGCTTAACCGCAAGGAGGGC

>GW458-11-26-14-LB1

GTCGAACGGCAGCGCGGGAGCAATCCTGGCGGCGAGTGGCGAACGGGTGAGTAATACATCGGAACGTGCCCAATCGTGGGGGATAACGCAGCGAAAGCTGTGCTAATACCGCATACGATCTACGGATGAAAGCAGGGGATCGCAAGACCTTGCGCGAATGGAGCGGCCGATGGCAGATTAGGTAGTTGGTGGGGTAAAGGCTCACCAAGCCTTCGATCTGTAGCTGGTCTGAGAGGACGACCAGCCACACTGGGACTGAGACACGGCCCAGACTCCTACGGGAGGCAGCAGTGGGGAATTTTGGACAATGGGCGAAAGCCTGATCCAGCCATGCCGCGTGCAGGATGAAGGCCTTCGGGTTGTAAACTGCTTTTGTACGGAACGAAACGGTCTTTTCTAATACAGAAGGCTAATGACGGTACCGTAAGAATAAGCACCGGCTAACTACGTGCCAGCAGCCGCGGTAATACGTAGGGTGCAAGCGTTAATCGGAATTACTGGGCGTAAAGCGTGCGCAGGCGGTTATGTAAGACAGTTGTGAAATCCCCGGGCTCAACCTGGGAACTGCATCTGTGACTGCATAGCTAGAGTACGGTAGAGGGGGATGGAATTCCGCGTGTAGCAGTGAAATGCGTAGATATGCGGAGGAACACCGATGGCGAAGGCAATCCCCTGGACCTGTACTGACGCTCATGCACGAAAGCGTGGGGAGCAAACAGGATTAGATACCCTGGTAGTCCACGCCCTAAACGATGTCAACTGGTTGTTGGGTCTTCACTGACTCAGTAACGAAGCTAACGCGTGAAGTTGACCGCCTGGGGAGTACGGCCGCAAGGTTGAAACTCAAAGGAATTGACGGGGACCCGCACAAGCGGTGGATGATGTGGTTTAATTCGATGCAACGCGAAAAACCTTACCCACCTTTGACATGTACGGAATTCGCCAGAGATGGCTTAGTGCTCGAAAGAGAACCGTAACACAGGTGCTGCATGGCTGTCGTCAGCTCGTGTCGTGAGATGTTGGGTTAAGTCCCGCAACGAGCGCAACCCTTGTCATTAGTTGCTACATTCAGTTGGGCACTCTAATGAGACTGCCGGTGACAAACCGGAGGAAGGTGGGGATGACGTCAAGTCCTCATGGCCCTTATAGGTGGGGCTACACACGTCATACAATGGCTGGTACAAAGGGTTGCCAACCCGCGAGGGGGAGCTAATCCCATAAAACCAGTCGTAGTCCGGATCGCAGTCTGCAACTCGACTGCGTGAAGTCGGAATCGCTAGTAATCGTGGATCAGAATGTCACGGTGAATACGTTCCCGGGTCTTGTACACACCGCCCGTCACACCATGGGAGCGGGTTCTGCCAGAAGTAGTTAGC

>GW458-11-26-14-LB2

GAGTGGCGAACGGGTGAGTAATATATCGGAACGTNCCCAANNGTGGGGGATAACGTAGCGAAAGTNNNGCTAATACCGCATACGATCTACGGATGAAAGCAGGGGATCTTNGNACCTTGNGCTCCTGGAGCGGCCGATNNCTGATTAGNNAGTTGGTGAGGTAAAGGCTCACCAAGGCNNCGATNNGTAGCTGGTCTGAGAGGACGACCAGCCACACTGGAACTGAGACACGGTCCAGACTCCTACGGGAGGCAGCAGTGGGGAATTTTGGACAATGGGCGCAAGCCTGATCCAGCCATGCCGCGTGAGTGANGAAGGCCTTCGGGTTGTAAANNNCTTTTGTNNGGAANGAAACGGNNTNGTCTAATATCCATTCNTAATGACGGTACCTGAAGAATAAGCACCGGCTAACTACGTGCCAGCAGCCGCGGTAATACGTAGGGTGCAAGCGTTAATCGGAATTACTGGGCGTAAAGCGTGCGCAGGCGGTTTTGTAAGTCWGACGTGAAATCCCCGGGCTCAACCTGGGAATTGCGTTGGAGACTGCANNGCTAGAATCTGGCAGAGGGGGATAGAATTCCACGTGTAGCAGTGAAATGCGTAGANATGTGGAGGAACACCGATGGCGAAGGCAGTCCCCTGGRTCAAGATTGACGCTCATGCACGAAAGCGTGGGGAGCAAACAGGATTAGATACCCTGGTAGTCCACGCCCTAAACGATGTCTACTAGTTGTCGGGTTTTAATTAACTTGGTAACGCAGCTAACGCGTGAAGTAGACCGCCTGGGGAGTACGGTCGCAAGATTNAAACTCAAAGGAATTGACGGGGACCCGCACAAGCGGTGGATGATGTGGATTAATTCGATGCAACGCGAAAAACCTTACCNACCCTTGACATGTACGGAATNNCNNAGAGANNTGGCAGTGCTCGAAAGAGAACCGTAACACAGGTGCTGCATGGCTGTCGTCAGCTCGTGTCGTGAGATGTTGGGTTAAGTCCCGCAACGAGCGCAACCCTTGTCATTAGTTGCTACATTCAGTTGGGCACTCTAATGAGACTGCCGGTGACAAACCGGAGGAAGGTGGGGATGACGTCAAGTCCTCATGGCCCTTATGGGTAGGGCTTCACACGTCATACAATGGTACATACAGAGGGCCGCCAACCCGCGAGGGGGAGCTAATCCCAGAAANNCTNTCGTAGTCCGGATCGNAGTCTGCAACTCGACTACGTGAAGGTGGAATCGCTAGTAATCGCGGATCAGCATGCCGCGGTGAATACGTTCCCGGGTCTTGTACACACCGCCCGTCACACCATGGGAGCGG

>GW458-11-26-14-LB3

CAGCACGGGTACTTGTACCTGGTGGCGAGCGGCGGACGGGTGAGTAATGCCTAGGAATCTGCCTGGTAGTGGGGGATAACGCTCGGAAACGGACGCTAATACCGCATACGTCCTACGGGAGAAAGCAGGGGACCTTCGGGCCTTGCGCTATCAGATGAGCCTAGGTCGGATTAGCTAGTTGGTGAGGTAATGGCTCACCAAGGCGACGATCCGTAACTGGTCTGAGAGGATGATCAGTCACACTGGAACTGAGACACGGTCCAGACTCCTACGGGAGGCAGCAGTGGGGAATATTGGACAATGGGCGAAAGCCTGATCCAGCCATGCCGCGTGTGTGAAGAAGGTCTTCGGATTGTAAAGCACTTTAAGTTGGGAGGAAGGGCAGTTACCTAATACGTATCTGTTTTGACGTTACCGACAGAATAAGCACCGGCTAACTCTGTGCCAGCAGCCGCGGTAATACAGAGGGTGCAAGCGTTAATCGGAATTACTGGGCGTAAAGCGCGCGTAGGTGGTTTGTTAAGTTGGATGTGAAATCCCCGGGCTCAACCTGGGAACTGCATTCAAAACTGACAAGCTAGAGTATGGTAGAGGGTGGTGGAATTTCCTGTGTAGCGGTGAAATGCGTAGATATAGGAAGGAACACCAGTGGCGAAGGCGACCACCTGGACTGATACTGACACTGAGGTGCGAAAGCGTGGGGAGCAAACAGGATTAGATACCCTGGTAGTCCACGCCGTAAACGATGTCAACTAGCCGTTGGGAGCCTTGAGCTCTTAGTGGCGCAGCTAACGCATTAAGTTGACCGCCTGGGGAGTACGGCCGCAAGGTTAAAACTCAAATGAATTGACGGGGGCCCGCACAAGCGGTGGAGCATGTGGTTTAATTCGAAGCAACGCGAAGAACCTTACCAGGCCTTGACATCCAATGAACTTTCCAGAGATGGATTGGTGCCTTCGGGAACATTGAGACAGGTGCTGCATGGCTGTCGTCAGCTCGTGTCGTGAGATGTTGGGTTAAGTCCCGTAACGAGCGCAACCCTTGTCCTTAGTTACCAGCACGTNATGGTGGGCACTCTAAGGAGACTGCCGGTGACAAACCGGAGGAAGGTGGGGATGACGTCAAGTCATCATGGCCCTTACGGCCTGGGCTACACACGTGCTACAATGGTCGGTACAGAGGGTTGCCAAGCCGCGAGGTGGAGCTAATCCCAGAAAACCGATCGTAGTCCGGATCGCAGTCTGCAACTCGACTGCGTGAAGTCGGAATCGCTAGTAATCGCGAATCAGAATGTCGCGGTGAATACGTTCCCGGGCCTTGTACACACCGCCCGTCACACCATGGGAG

>GW458-11-26-14-LB4

GTCGAGCGGATGAAGGGAGCTTGCTCCTGAATTCAGCGGCGGACGGGTGAGTAATGCCTAGGAATCTGCCTGGTAGTGGGGGACAACGTTTCGAAAGGAACGCTAATACCGCATACGTCCTACGGGAGAAAGCAGGGGACCTTCGGGCCTTGCGCTATCAGATGAGCCTAGGTCGGATTAGCTAGTTGGTGAGGTAATGGCTCACCAAGGCGACGATCCGTAACTGGTCTGAGAGGATGATCAGTCACACTGGAACTGAGACACGGTCCAGACTCCTACGGGAGGCAGCAGTGGGGAATATTGGACAATGGGCGAAAGCCTGATCCAGCCATGCCGCGTGTGTGAAGAAGGTCTTCGGATTGTAAAGCACTTTAAGTTGGGAGGAAGGGTTGTAGATTAATACTCTGCAATTTTGACGTTACCGACAGAATAAGCACCGGCTAACTCTGTGCCAGCAGCCGCGGTAATACAGAGGGTGCAAGCGTTAATCGGAATTACTGGGCGTAAAGCGCGCGTAGGTGGTTCGTTAAGTTGGATGTGAAATCCCCGGGCTCAACCTGGGAACTGCATCCAAAACTGGCGAGCTAGAGTATGGTAGAGGGTGGTGGAATTTCCTGTGTAGCGGTGAAATGCGTAGATATAGGAAGGAACACCAGTGGCGAAGGCGACCACCTGGACTGATACTGACACTGAGGTGCGAAAGCGTGGGGAGCAAACAGGATTAGATACCCTGGTAGTCCACGCCGTAAACGATGTCAACTAGCCGTTGGGAGCCTTGAGCTCTTAGTGGCGCAGCTAACGCATTAAGTTGACCGCCTGGGGAGTACGGCCGCAAGGTTAAAACTCAAATGAATTGACGGGGGCCCGCACAAGCGGTGGAGCATGTGGTTTAATTCGAAGCAACGCGAAGAACCTTACCAGGCCTTGACATCCAATGAACTTTCCAGAGATGGATTGGTGCCTTCGGGAACATTGAGACAGGTGCTGCATGGCTGTCGTCAGCTCGTGTCGTGAGATGTTGGGTTAAGTCCCGTAACGAGCGCAACCCTTGTCCTTAGTTACCAGCACGTAATGGTGGGCACTCTAAGGAGACTGCCGGTGACAAACCGGAGGAAGGTGGGGATGACGTCAAGTCATCATGGCCCTTACGGCCTGGGCTACACACGTGCTACAATGGTCGGTACAAAGGGTTGCCAAGCCGCGAGGTGGAGCTAATCCCATAAAACCGATCGTAGTCCGGATCGCAGTCTGCAACTCGACTGCGTGAAGTCGGAATCGCTAGTAATCGCGAATCAGAATGTCGCGGTGAATACGTTCCCGGGCCTTGTACACACCGCCCGTCACACCATGGGAGTGGGTTGCACCAGAAGTAG

>GW458-11-26-14-TSB1

GGGAGCTTGCTCCTGAATTCAGCGGCGGACGGGTGAGTAATGCCTAGGAATCTGCCTGGTAGTGGGGGACAACGTTTCGAAAGGAACGCTAATACCGCATACGTCCTACGGGAGAAAGCAGGGGACCTTCGGGCCTTGCGCTATCAGATGAGCCTAGGTCGGATTAGCTAGTTGGTGAGGTAATGGCTCACCAAGGCGACGATCCGTAACTGGTCTGAGAGGATGATCAGTCACACTGGAACTGAGACACGGTCCAGACTCCTACGGGAGGCAGCAGTGGGGAATATTGGACAATGGGCGAAAGCCTGATCCAGCCATGCCGCGTGTGTGAAGAAGGTCTTCGGATTGTAAAGCACTTTAAGTTGGGAGGAAGGGTTGTAGATTAATACTCTGCAATTTTGACGTTACCGACAGAATAAGCACCGGCTAACTCTGTGCCAGCAGCCGCGGTAATACAGAGGGTGCAAGCGTTAATCGGAATTACTGGGCGTAAAGCGCGCGTAGGTGGTTCGTTAAGTTGGATGTGAAATCCCCGGGCTCAACCTGGGAACTGCATCCAAAACTGGCGAGCTAGAGTATGGTAGAGGGTGGTGGAATTTCCTGTGTAGCGGTGAAATGCGTAGATATAGGAAGGAACACCAGTGGCGAAGGCGACCACCTGGACTGATACTGACACTGAGGTGCGAAAGCGTGGGGAGCAAACAGGATTAGATACCCTGGTAGTCCACGCCGTAAACGATGTCAACTAGCCGTTGGGAGCCTTGAGCTCTTAGTGGCGCAGCTAACGCATTAAGTTGACCGCCTGGGGAGTACGGCCGCAAGGTTAAAACTCAAATGAATTGACGGGGGCCCGCACAAGCGGTGGAGCATGTGGTTTAATTCGAAGCAACGCGAAGAACCTTACCAGGCCTTGACATCCAATGAACTTTCCAGAGATGGATTGGTGCCTTCGGGAACATTGAGACAGGTGCTGCATGGCTGTCGTCAGCTCGTGTCGTGAGATGTTGGGTTAAGTCCCGTAACGAGCGCAACCCTTGTCCTTAGTTACCAGCACGTAATGGTGGGCACTCTAAGGAGACTGCCGGTGACAAACCGGAGGAAGGTGGGGATGACGTCAAGTCATCATGGCCCTTACGGCCTGGGCTACACACGTGCTACAATGGTCGGTACAAAGGGTTGCCAAGCCGCGAGGTGGAGCTAATCCCATAAAACCGATCGTAGTCCGGATCGCAGTCTGCAACTCGACTGCGTGAAGTCGGAATCGCTAGTAATCGCGAATCAGAATGTCGCGGTGAATACGTTCCCGGGCCTTGTACACACCGCCCGTCACACCATGGGAGTGGGTTGCACCAGAAGTAGC

>GW458-11-26-14-TSB2

CGCGGGAGCAATCCTGGCGGCGAGTGGCGAACGGGTGAGTAATACATCGGAACGTGCCCAATCGTGGGGGATAACGCAGCGAAAGCTGTGCTAATACCGCATACGATCTACGGATGAAAGCAGGGGATCGCAAGACCTTGCGCGAATGGAGCGGCCGATGGCAGATTAGGTAGTTGGTGGGGTAAAGGCTCACCAAGCCTTCGATCTGTAGCTGGTCTGAGAGGACGACCAGCCACACTGGGACTGAGACACGGCCCAGACTCCTACGGGAGGCAGCAGTGGGGAATTTTGGACAATGGGCGAAAGCCTGATCCAGCCATGCCGCGTGCAGGATGAAGGCCTTCGGGTTGTAAACTGCTTTTGTACGGAACGAAACGGTCTTTTCTAATACAGAAGGCTAATGACGGTACCGTAAGAATAAGCACCGGCTAACTACGTGCCAGCAGCCGCGGTAATACGTAGGGTGCAAGCGTTAATCGGAATTACTGGGCGTAAAGCGTGCGCAGGCGGTTATGTAAGACAGTTGTGAAATCCCCGGGCTCAACCTGGGAACTGCATCTGTGACTGCATAGCTAGAGTACGGTAGAGGGGGATGGAATTCCGCGTGTAGCAGTGAAATGCGTAGATATGCGGAGGAACACCGATGGCGAAGGCAATCCCCTGGACCTGTACTGACGCTCATGCACGAAAGCGTGGGGAGCAAACAGGATTAGATACCCTGGTAGTCCACGCCCTAAACGATGTCAACTGGTTGTTGGGTCTTCACTGACTCAGTAACGAAGCTAACGCGTGAAGTTGACCGCCTGGGGAGTACGGCCGCAAGGTTGAAACTCAAAGGAATTGACGGGGACCCGCACAAGCGGTGGATGATGTGGTTTAATTCGATGCAACGCGAAAAACCTTACCCACCTTTGACATGTACGGAATTCGCCAGAGATGGCTTAGTGCTCGAAAGAGAACCGTAACACAGGTGCTGCATGGCTGTCGTCAGCTCGTGTCGTGAGATGTTGGGTTAAGTCCCGCAACGAGCGCAACCCTTGTCATTAGTTGCTACATTCAGTTGGGCACTCTAATGAGACTGCCGGTGACAAACCGGAGGAAGGTGGGGATGACGTCAAGTCCTCATGGCCCTTATAGGTGGGGCTACACACGTCATACAATGGCTGGTACAAAGGGTTGCCAACCCGCGAGGGGGAGCTAATCCCATAAAACCAGTCGTAGTCCGGATCGCAGTCTGCAACTCGACTGCGTGAAGTCGGAATCGCTAGTAATCGTGGATCAGAATGTCACGGTGAATACGTTCCCGGGTCTTGTACACACCGCCCGTCACACCATGGGAGCGGGTTCTGCCAGAAGTAG

>GW458-11-26-14-TSB3

ACGGGTACTTGTACCTGGTGGCGAGCGGCGGACGGGTGAGTAATGCCTAGGAATCTGCCTGGTAGTGGGGGATAACGCTCGGAAACGGACGCTAATACCGCATACGTCCTACGGGAGAAAGCAGGGGACCTTCGGGCCTTGCGCTATCAGATGAGCCTAGGTCGGATTAGCTAGTTGGTGAGGTAATGGCTCACCAAGGCGACGATCCGTAACTGGTCTGAGAGGATGATCAGTCACACTGGAACTGAGACACGGTCCAGACTCCTACGGGAGGCAGCAGTGGGGAATATTGGACAATGGGCGAAAGCCTGATCCAGCCATGCCGCGTGTGTGAAGAAGGTCTTCGGATTGTAAAGCACTTTAAGTTGGGAGGAAGGGCAGTTACCTAATACGTATCTGTTTTGACGTTACCGACAGAATAAGCACCGGCTAACTCTGTGCCAGCAGCCGCGGTAATACAGAGGGTGCAAGCGTTAATCGGAATTACTGGGCGTAAAGCGCGCGTAGGTGGTTTGTTAAGTTGGATGTGAAATCCCCGGGCTCAACCTGGGAACTGCATTCAAAACTGACAAGCTAGAGTATGGTAGAGGGTGGTGGAATTTCCTGTGTAGCGGTGAAATGCGTAGATATAGGAAGGAACACCAGTGGCGAAGGCGACCACCTGGACTGATACTGACACTGAGGTGCGAAAGCGTGGGGAGCAAACAGGATTAGATACCCTGGTAGTCCACGCCGTAAACGATGTCAACTAGCCGTTGGGAGCCTTGAGCTCTTAGTGGCGCAGCTAACGCATTAAGTTGACCGCCTGGGGAGTACGGCCGCAAGGTTAAAACTCAAATGAATTGACGGGGGCCCGCACAAGCGGTGGAGCATGTGGTTTAATTCGAAGCAACGCGAAGAACCTTACCAGGCCTTGACATCCAATGAACTTTCCAGAGATGGATTGGTGCCTTCGGGAACATTGAGACAGGTGCTGCATGGCTGTCGTCAGCTCGTGTCGTGAGATGTTGGGTTAAGTCCCGTAACGAGCGCAACCCTTGTCCTTAGTTACCAGCACGNNNTGGTGGGCACTCTAAGGAGACTGCCGGTGACAAACCGGAGGAAGGTGGGGATGACGTCAAGTCATCATGGCCCTTACGGCCTGGGCTACACACGTGCTACAATGGTCGGTACAGAGGGTTGCCAAGCCGCGAGGTGGAGCTAATCCCAGAAAACCGATCGTAGTCCGGATCGCAGTCTGCAACTCGACTGCGTGAAGTCGGAATCGCTAGTAATCGCGAATCAGAATGTCGCGGTGAATACGTTCCCGGGCCTTGTACACACCGCCCGTCACACCATGGGAGTGGGTTGCACCAGAAGTAGCTAGTC

>GW458-11-26-14-TSB4

ATGCTGACGAGTGGCGAACGGGTGAGTAATACATCGGAACGTGCCCGATCGTGGGGGATAACGGAGCGAAAGCTTTGCTAATACCGCATACGATCTACGGATGAAAGCAGGGGACCGCAAGGCCTTGCGCGGACGGAGCGGCCGATGGCAGATTAGGTAGTTGGTGGGATAAAAGCTTACCAAGCCGACGATCTGTAGCTGGTCTGAGAGGACGACCAGCCACACTGGGACTGAGACACGGCCCAGACTCCTACGGGAGGCAGCAGTGGGGAATTTTGGACAATGGGCGCAAGCCTGATCCAGCCATGCCGCGTGCAGGATGAAGGCCTTCGGGTTGTAAACTGCTTTTGTACGGAACGAAAAGACTCTTTCTAATAAAGAGGGTCCATGACGGTACCGTAAGAATAAGCACCGGCTAACTACGTGCCAGCAGCCGCGGTAATACGTAGGGTGCAAGCGTTAATCGGAATTACTGGGCGTAAAGCGTGCGCAGGCGGTTATGTAAGACAGATGTGAAATCCCCGGGCTCAACCTGGGAACTGCATTTGTGACTGCATAGCTAGAGTACGGCAGAGGGGGATGGAATTCCGCGTGTAGCAGTGAAATGCGTAGATATGCGGAGGAACACCGATGGCGAAGGCAATCCCCTGGGCCTGTACTGACGCTCATGCACGAAAGCGTGGGGAGCAAACAGGATTAGATACCCTGGTAGTCCACGCCCTAAACGATGTCAACTGGTTGTTGGGTCTTCACTGACTCAGTAACGAAGCTAACGCGTGAAGTTGACCGCCTGGGGAGTACGGCCGCAAGGTTGAAACTCAAAGGAATTGACGGGGACCCGCACAAGCGGTGGATGATGTGGTTTAATTCGATGCAACGCGAAAAACCTTACCCACCTTTGACATGTACGGAATCCTTTAGAGATAGAGGAGTGCTCGAAAGAGAACCGTAACACAGGTGCTGCATGGCTGTCGTCAGCTCGTGTCGTGAGATGTTGGGTTAAGTCCCGCAACGAGCGCAACCCTTGCCATTAGTTGCTACGAAAGGGCACTCTAATGGGACTGCCGGTGACAAACCGGAGGAAGGTGGGGATGACGTCAAGTCCTCATGGCCCTTATAGGTGGGGCTACACACGTCATACAATGGCTGGTACAGAGGGTTGCCAACCCGCGAGGGGGAGCCAATCCCATAAAGCCAGTCGTAGTCCGGATCGCAGTCTGCAACTCGACTGCGTGAAGTCGGAATCGCTAGTAATCGCGGATCAGAATGTCGCGGTGAATACGTTCCCGGGTCTTGTACACACCGCCCGTCACACCATGGGAGCGGGTTCTGCCA

>GW458-11-26-14-TSB5

TCTTCGGAGTTAGTGGCGGACGGGTGAGTAACACGTGGGAACGTGCCTTTAGGTTCGGAATAACTCAGGGAAACTTGTGCTAATACCGAATGTGCCCTTCGGGGGAAAGATTTATCGCCTTTAGAGCGGCCCGCGTCTGATTAGCTAGTTGGTGAGGTAAAGGCTCACCAAGGCGACGATCAGTAGCTGGTCTGAGAGGATGATCAGCCACATTGGGACTGAGACACGGCCCAAACTCCTACGGGAGGCAGCAGTGGGGAATCTTGCGCAATGGGCGAAAGCCTGACGCAGCCATGCCGCGTGAATGATGAAGGTCTTAGGATTGTAAAATTCTTTCAGTAGGGACGATAATGACGGTACCTACAGAAGAAGCCCCGGCTAACTTCGTGCCAGCAGCCGCGGTAATACGAAGGGGGCTAGCGTTGCTCGGAATTACTGGGCGTAAAGGGAGCGTAGGCGGACATTTAAGTCAGGGGTGAAATCCCGGGGCTCAACCTCGGAATTGCCTTTGATACTGGGTGTCTTGAGTGTGAGAGAGGTATGTGGAACTCCGAGTGTAGAGGTGAAATTCGTAGATATTCGGAAGAACACCAGTGGCGAAGGCGACATACTGGCTCATTACTGACGCTGAGGCTCGAAAGCGTGGGGAGCAAACAGGATTAGATACCCTGGTAGTCCACGCCGTAAACGATGATTGCTAGTTGTCGGGATGCATGCATTTCGGTGACGCAGCTAACGCATTAAGCAATCCGCCTGGGGAGTACGGTCGCAAGATTAAAACTCAAAGGAATTGACGGGGGCCCGCACAAGCGGTGGAGCATGTGGTTTAATTCGAAGCAACGCGCAGAACCTTACCACCTTTTGACATGCCTGGACCGCCAGAGAGATCTGGCTTTCCCTTCGGGGACTAGGACACAGGTGCTGCATGGCTGTCGTCAGCTCGTGTCGTGAGATGTTGGGTTAAGTCCCGCAACGAGCGCAACCCTCGCCATTAGTTGCCATCATTTAGTTGGGAACTCTAATGGGACTGCCGGTGCTAAGCCGGAGGAAGGTGGGGATGACGTCAAGTCCTCATGGCCCTTACAGGGTGGGCTACACACGTGCTACAATGGCGACTACAGAGGGTTAATCCTTAAAAGTCGTCTCAGTTCGGATTGTCCTCTGCAACTCGAGGGCATGAAGTTGGAATCGCTAGTAATCGCGGATCAGCATGCCGCGGTGAATACGTTCCCGGGCCTTGTACACACCGCCCGTCACACCATGGGAGTTGGTTCTACCCGAAGGCGCTGCG

>GW458-12-2-14-LB1

CAATCCTGGCGGCGAGTGGCGAACGGGTGAGTAATACATCGGAACGTGCCCAATCGTGGGGGATAACGCAGCGAAAGCTGTGCTAATACCGCATACGATCTACGGATGAAAGCAGGGGATCGCAAGACCTTGCGCGAATGGAGCGGCCGATGGCAGATTAGGTAGTTGGTGGGGTAAAGGCTCACCAAGCCTTCGATCTGTAGCTGGTCTGAGAGGACGACCAGCCACACTGGGACTGAGACACGGCCCAGACTCCTACGGGAGGCAGCAGTGGGGAATTTTGGACAATGGGCGAAAGCCTGATCCAGCCATGCCGCGTGCAGGATGAAGGCCTTCGGGTTGTAAACTGCTTTTGTACGGAACGAAACGGTCTTTTCTAATACAGAAGGCTAATGACGGTACCGTAAGAATAAGCACCGGCTAACTACGTGCCAGCAGCCGCGGTAATACGTAGGGTGCAAGCGTTAATCGGAATTACTGGGCGTAAAGCGTGCGCAGGCGGTTATGTAAGACAGTTGTGAAATCCCCGGGCTCAACCTGGGAACTGCATCTGTGACTGCATAGCTAGAGTACGGTAGAGGGGGATGGAATTCCGCGTGTAGCAGTGAAATGCGTAGATATGCGGAGGAACACCGATGGCGAAGGCAATCCCCTGGACCTGTACTGACGCTCATGCACGAAAGCGTGGGGAGCAAACAGGATTAGATACCCTGGTAGTCCACGCCCTAAACGATGTCAACTGGTTGTTGGGTCTTCACTGACTCAGTAACGAAGCTAACGCGTGAAGTTGACCGCCTGGGGAGTACGGCCGCAAGGTTGAAACTCAAAGGAATTGACGGGGACCCGCACAAGCGGTGGATGATGTGGTTTAATTCGATGCAACGCGAAAAACCTTACCCACCTTTGACATGTACGGAATTCGCCAGAGATGGCTTAGTGCTCGAAAGAGAACCGTAACACAGGTGCTGCATGGCTGTCGTCAGCTCGTGTCGTGAGATGTTGGGTTAAGTCCCGCAACGAGCGCAACCCTTGTCATTAGTTGCTACATTCAGTTGGGCACTCTAATGAGACTGCCGGTGACAAACCGGAGGAAGGTGGGGATGACGTCAAGTCCTCATGGCCCTTATAGGTGGGGCTACACACGTCATACAATGGCTGGTACAAAGGGTTGCCAACCCGCGAGGGGGAGCTAATCCCATAAAACCAGTCGTAGTCCGGATCGCAGTCTGCAACTCGACTGCGTGAAGTCGGAATCGCTAGTAATCGTGGATCAGAATGTCACGGTGAATACGTTCCCGGGTCTTGTACACACCGCCCGTCACACCATGGGAGCGGGTTCTGCCAGAAGTAGTTAGC

>GW458-12-2-14-LB2

GCAGTCGAGCGGCAGCACGGGTACTTGTACCTGGTGGCGAGCGGCGGACGGGTGAGTAATGCCTAGGAATCTGCCTGGTAGTGGGGGATAACGCTCGGAAACGGACGCTAATACCGCATACGTCCTACGGGAGAAAGCAGGGGACCTTCGGGCCTTGCGCTATCAGATGAGCCTAGGTCGGATTAGCTAGTTGGTGAGGTAATGGCTCACCAAGGCGACGATCCGTAACTGGTCTGAGAGGATGATCAGTCACACTGGAACTGAGACACGGTCCAGACTCCTACGGGAGGCAGCAGTGGGGAATATTGGACAATGGGCGAAAGCCTGATCCAGCCATGCCGCGTGTGTGAAGAAGGTCTTCGGATTGTAAAGCACTTTAAGTTGGGAGGAAGGGCAGTTACCTAATACGTATCTGTTTTGACGTTACCGACAGAATAAGCACCGGCTAACTCTGTGCCAGCAGCCGCGGTAATACAGAGGGTGCAAGCGTTAATCGGAATTACTGGGCGTAAAGCGCGCGTAGGTGGTTTGTTAAGTTGGATGTGAAATCCCCGGGCTCAACCTGGGAACTGCATTCAAAACTGACAAGCTAGAGTATGGTAGAGGGTGGTGGAATTTCCTGTGTAGCGGTGAAATGCGTAGATATAGGAAGGAACACCAGTGGCGAAGGCGACCACCTGGACTGATACTGACACTGAGGTGCGAAAGCGTGGGGAGCAAACAGGATTAGATACCCTGGTAGTCCACGCCGTAAACGATGTCAACTAGCCGTTGGGAGCCTTGAGCTCTTAGTGGCGCAGCTAACGCATTAAGTTGACCGCCTGGGGAGTACGGCCGCAAGGTTAAAACTCAAATGAATTGACGGGGGCCCGCACAAGCGGTGGAGCATGTGGTTTAATTCGAAGCAACGCGAAGAACCTTACCAGGCCTTGACATCCAATGAACTTTCCAGAGATGGATTGGTGCCTTCGGGAACATTGAGACAGGTGCTGCATGGCTGTCGTCAGCTCGTGTCGTGAGATGTTGGGTTAAGTCCCGTAACGAGCGCAACCCTTGTCCTTAGTTACCAGCACGNNATGGTGGGCACTCTAAGGAGACTGCCGGTGACAAACCGGAGGAAGGTGGGGATGACGTCAAGTCATCATGGCCCTTACGGCCTGGGCTACACACGTGCTACAATGGTCGGTACAGAGGGTTGCCAAGCCGCGAGGTGGAGCTAATCCCAGAAAACCGATCGTAGTCCGGATCGCAGTCTGCAACTCGACTGCGTGAAGTCGGAATCGCTAGTAATCGCGAATCAGAATGTCGCGGTGAATACGTTCCCGGGCCTTGTACACACCGCCCGTCACACCATGGGAGTGGGTTGCACCAGAAGTAGCTAGT

>GW458-12-2-14-TSB1

AAGTCGAGCGGCAGCACGGGTACTTGTACCTGGTGGCGAGCGGCGGACGGGTGAGTAATGCCTAGGAATCTGCCTGGTAGTGGGGGATAACGCTCGGAAACGGACGCTAATACCGCATACGTCCTACGGGAGAAAGCAGGGGACCTTCGGGCCTTGCGCTATCAGATGAGCCTAGGTCGGATTAGCTAGTTGGTGAGGTAATGGCTCACCAAGGCGACGATCCGTAACTGGTCTGAGAGGATGATCAGTCACACTGGAACTGAGACACGGTCCAGACTCCTACGGGAGGCAGCAGTGGGGAATATTGGACAATGGGCGAAAGCCTGATCCAGCCATGCCGCGTGTGTGAAGAAGGTCTTCGGATTGTAAAGCACTTTAAGTTGGGAGGAAGGGCAGTTACCTAATACGTATCTGTTTTGACGTTACCGACAGAATAAGCACCGGCTAACTCTGTGCCAGCAGCCGCGGTAATACAGAGGGTGCAAGCGTTAATCGGAATTACTGGGCGTAAAGCGCGCGTAGGTGGTTTGTTAAGTTGGATGTGAAATCCCCGGGCTCAACCTGGGAACTGCATTCAAAACTGACAAGCTAGAGTATGGTAGAGGGTGGTGGAATTTCCTGTGTAGCGGTGAAATGCGTAGATATAGGAAGGAACACCAGTGGCGAAGGCGACCACCTGGACTGATACTGACACTGAGGTGCGAAAGCGTGGGGAGCAAACAGGATTAGATACCCTGGTAGTCCACGCCGTAAACGATGTCAACTAGCCGTTGGGAGCCTTGAGCTCTTAGTGGCGCAGCTAACGCATTAAGTTGACCGCCTGGGGAGTACGGCCGCAAGGTTAAAACTCAAATGAATTGACGGGGGCCCGCACAAGCGGTGGAGCATGTGGTTTAATTCGAAGCAACGCGAAGAACCTTACCAGGCCTTGACATCCAATGAACTTTCCAGAGATGGATTGGTGCCTTCGGGAACATTGAGACAGGTGCTGCATGGCTGTCGTCAGCTCGTGTCGTGAGATGTTGGGTTAAGTCCCGTAACGAGCGCAACCCTTGTCCTTAGTTACCAGCACGTNATGGTGGGCACTCTAAGGAGACTGCCGGTGACAAACCGGAGGAAGGTGGGGATGACGTCAAGTCATCATGGCCCTTACGGCCTGGGCTACACACGTGCTACAATGGTCGGTACAGAGGGTTGCCAAGCCGCGAGGTGGAGCTAATCCCAGAAAACCGATCGTAGTCCGGATCGCAGTCTGCAACTCGACTGCGTGAAGTCGGAATCGCTAGTAATCGCGAATCAGAATGTCGCGGTGAATACGTTCCCGGGCCTTGTACACACCGCCCGTCACACCATGGGAGTGGGTTGCACCAGAAGTAGCTAGTCTA

>GW458-12-2-14-TSB2

TGCAGTCGAACGGTGTAGCAATACATAGTGGCGCACGGGTGCGTAACACGTATGCAACCTACCTTATACTTAAGGATAGCCTTTGGAAACGGAGATTAATACTTAATGGTACATTGATTTGGCATCAGATTAATGTTAAAGATTTATTGGTATAAGATGGGCATGCGTCCAATTAGTTAGTTGGTGAGGTAACGGCTCACCAAGACGATGATTGGTAGGGGAACTGAGAGGTTAATCCCCCACACTGGCACTGAGATACGGGCCAGACTCCTACGGGAGGCAGCAGTAGGGAATATTGGGCAATGGAGGCAACTCTGACCCAGCCATGCCGCGTGCAGGATGAAGGCGTTATGCGTTGTAAACTGCTTTTATATAGGAAGAAACAGACCTTGCGAGGTAAATTGACGGTACTATATGAATAAGCACCGGCTAACTCCGTGCCAGCAGCCGCGGTAATACGGAGGGTGCAAGCGTTGTCCGGATTTATTGGGTTTAAAGGGTGCGTAGGCGGTTTATTAAGTCAGTGGTGAAAGACGGTCGCTCAACGATTGCAGTGCCATTGATACTGGTAGACTTGAGTATGATTGAGGTAGCTGGAATGGATAGTGTAGCGGTGAAATGCATAGATATTATCCAGAACACCAATTGCGTAGGCAAGTTACTAAGTCATAACTGACGCTGATGCACGAAAGTGTGGGTATCAAACAGGATTAGATACCCTGGTAGTCCACACTGTAAACGATGATAACTAACTGTTGGCTTTCGGGTCAGTGGTACAGAGAAATCGTTAAGTTATCCACCTGGGGAGTACGCCGGCAACGGTGAAACTCAAAGGAATTGACGGGGGTCCGCACAAGCGGTGGAGCATGTGGTTTAATTCGATGATACGCGAGGAACCTTACCTAGGCTAGAATGTGAAGGAATGTATCAGAAATGGTGCAGTCAGCAATGACCTGAAACAAGGTGCTGCATGGCTGTCGTCAGCTCGTGCCGTGAGGTGTTGGGTTAAGTCCCGCAACGAGCGCAACCCCTGTTGTTAGTTGCCATCAGGTAATGCTGGGAACTCTAACAAGACTGCCTACGCAAGTAGAGAGGAAGGAGGGGACGACGTCAAGTCATCATGGCCCTTACGCCTAGGGCAACACACGTGCTACAATGGACGGTACAGCAGGTCGCTATGTGGTAACACAATGCCAATCTTGAAAGCCGTTCTCAGTTCGGATTGGGGTCTGCAACTCGACCCTATGAAGCTGGAATCGCTAGTAATCGGATATCAGCTATGATCCGGTGAATACGTTCCCGGACCTTGTACACACCGCCCGTCAAGCCATGAAAGTCTGGTAGACCTAAAGC

>GW458-12-2-14-TSB3

CGGCAGCGCGGGAGCAATCCTGGCGGCGAGTGGCGAACGGGTGAGTAATACATCGGAACGTGCCCAATCGTGGGGGATAACGCAGCGAAAGCTGTGCTAATACCGCATACGATCTACGGATGAAAGCAGGGGATCGCAAGACCTTGCGCGAATGGAGCGGCCGATGGCAGATTAGGTAGTTGGTGGGGTAAAGGCTCACCAAGCCTTCGATCTGTAGCTGGTCTGAGAGGACGACCAGCCACACTGGGACTGAGACACGGCCCAGACTCCTACGGGAGGCAGCAGTGGGGAATTTTGGACAATGGGCGAAAGCCTGATCCAGCCATGCCGCGTGCAGGATGAAGGCCTTCGGGTTGTAAACTGCTTTTGTACGGAACGAAACGGTCTTTTCTAATACAGAAGGCTAATGACGGTACCGTAAGAATAAGCACCGGCTAACTACGTGCCAGCAGCCGCGGTAATACGTAGGGTGCAAGCGTTAATCGGAATTACTGGGCGTAAAGCGTGCGCAGGCGGTTATGTAAGACAGTTGTGAAATCCCCGGGCTCAACCTGGGAACTGCATCTGTGACTGCATAGCTAGAGTACGGTAGAGGGGGATGGAATTCCGCGTGTAGCAGTGAAATGCGTAGATATGCGGAGGAACACCGATGGCGAAGGCAATCCCCTGGACCTGTACTGACGCTCATGCACGAAAGCGTGGGGAGCAAACAGGATTAGATACCCTGGTAGTCCACGCCCTAAACGATGTCAACTGGTTGTTGGGTCTTCACTGACTCAGTAACGAAGCTAACGCGTGAAGTTGACCGCCTGGGGAGTACGGCCGCAAGGTTGAAACTCAAAGGAATTGACGGGGACCCGCACAAGCGGTGGATGATGTGGTTTAATTCGATGCAACGCGAAAAACCTTACCCACCTTTGACATGTACGGAATTCGCCAGAGATGGCTTAGTGCTCGAAAGAGAACCGTAACACAGGTGCTGCATGGCTGTCGTCAGCTCGTGTCGTGAGATGTTGGGTTAAGTCCCGCAACGAGCGCAACCCTTGTCATTAGTTGCTACATTCAGTTGGGCACTCTAATGAGACTGCCGGTGACAAACCGGAGGAAGGTGGGGATGACGTCAAGTCCTCATGGCCCTTATAGGTGGGGCTACACACGTCATACAATGGCTGGTACAAAGGGTTGCCAACCCGCGAGGGGGAGCTAATCCCATAAAACCAGTCGTAGTCCGGATCGCAGTCTGCAACTCGACTGCGTGAAGTCGGAATCGCTAGTAATCGTGGATCAGAATGTCACGGTGAATACGTTCCCGGGTCTTGTACACACCGCCCGTCACACCATGGGAGCGGGTTCTGCCAGAAG

>GW458-12-9-14-LB1

GCAGTCGAGCGGCAGCACGGGTACTTGTACCTGGTGGCGAGCGGCGGACGGGTGAGTAATGCCTAGGAATCTGCCTGGTAGTGGGGGATAACGCTCGGAAACGGACGCTAATACCGCATACGTCCTACGGGAGAAAGCAGGGGACCTTCGGGCCTTGCGCTATCAGATGAGCCTAGGTCGGATTAGCTAGTTGGTGAGGTAATGGCTCACCAAGGCGACGATCCGTAACTGGTCTGAGAGGATGATCAGTCACACTGGAACTGAGACACGGTCCAGACTCCTACGGGAGGCAGCAGTGGGGAATATTGGACAATGGGCGAAAGCCTGATCCAGCCATGCCGCGTGTGTGAAGAAGGTCTTCGGATTGTAAAGCACTTTAAGTTGGGAGGAAGGGCAGTTACCTAATACGTATCTGTTTTGACGTTACCGACAGAATAAGCACCGGCTAACTCTGTGCCAGCAGCCGCGGTAATACAGAGGGTGCAAGCGTTAATCGGAATTACTGGGCGTAAAGCGCGCGTAGGTGGTTTGTTAAGTTGGATGTGAAATCCCCGGGCTCAACCTGGGAACTGCATTCAAAACTGACAAGCTAGAGTATGGTAGAGGGTGGTGGAATTTCCTGTGTAGCGGTGAAATGCGTAGATATAGGAAGGAACACCAGTGGCGAAGGCGACCACCTGGACTGATACTGACACTGAGGTGCGAAAGCGTGGGGAGCAAACAGGATTAGATACCCTGGTAGTCCACGCCGTAAACGATGTCAACTAGCCGTTGGGAGCCTTGAGCTCTTAGTGGCGCAGCTAACGCATTAAGTTGACCGCCTGGGGAGTACGGCCGCAAGGTTAAAACTCAAATGAATTGACGGGGGCCCGCACAAGCGGTGGAGCATGTGGTTTAATTCGAAGCAACGCGAAGAACCTTACCAGGCCTTGACATCCAATGAACTTTCCAGAGATGGATTGGTGCCTTCGGGAACATTGNAGACAGGTGCTGCATGGCTGTCGTCAGCTCGTGTCGTGAGATGTTGGGTTAAGTCCCGTAACGAGCGCAACCCTTGTCCTTAGTTACCAGCACGTNATGGTGGGCACTCTAAGGAGACTGCCGGTGACAAACCGGAGGAAGGTGGGGATGACGTCAAGTCATCATGGCCCTTACGGCCTGGGCTACACACGTGCTACAATGGTCGGTACAGAGGGTTGCCAAGCCGCGAGGTGGAGCTAATCCCAGAAAACCGATCGTAGTCCGGATCGCAGTCTGCAACTCGACTGCGTGAAGTCGGAATCGCTAGTAATCGCGAATCAGAATGTCGCGGTGAATACGTTCCCGGGCCTTGTACACACCGCCCGTCACACCATGGGAGTGGGTTGCACCAGAAGTAGCTAGTCT

>GW458-12-9-14-LB2

CGCGGGAGCAATCCTGGCGGCGAGTGGCGAACGGGTGAGTAATACATCGGAACGTGCCCAATCGTGGGGGATAACGCAGCGAAAGCTGTGCTAATACCGCATACGATCTACGGATGAAAGCAGGGGATCGCAAGACCTTGCGCGAATGGAGCGGCCGATGGCAGATTAGGTAGTTGGTGGGGTAAAGGCTCACCAAGCCTTCGATCTGTAGCTGGTCTGAGAGGACGACCAGCCACACTGGGACTGAGACACGGCCCAGACTCCTACGGGAGGCAGCAGTGGGGAATTTTGGACAATGGGCGAAAGCCTGATCCAGCCATGCCGCGTGCAGGATGAAGGCCTTCGGGTTGTAAACTGCTTTTGTACGGAACGAAACGGTCTTTTCTAATACAGAAGGCTAATGACGGTACCGTAAGAATAAGCACCGGCTAACTACGTGCCAGCAGCCGCGGTAATACGTAGGGTGCAAGCGTTAATCGGAATTACTGGGCGTAAAGCGTGCGCAGGCGGTTATGTAAGACAGTTGTGAAATCCCCGGGCTCAACCTGGGAACTGCATCTGTGACTGCATAGCTAGAGTACGGTAGAGGGGGATGGAATTCCGCGTGTAGCAGTGAAATGCGTAGATATGCGGAGGAACACCGATGGCGAAGGCAATCCCCTGGACCTGTACTGACGCTCATGCACGAAAGCGTGGGGAGCAAACAGGATTAGATACCCTGGTAGTCCACGCCCTAAACGATGTCAACTGGTTGTTGGGTCTTCACTGACTCAGTAACGAAGCTAACGCGTGAAGTTGACCGCCTGGGGAGTACGGCCGCAAGGTTGAAACTCAAAGGAATTGACGGGGACCCGCACAAGCGGTGGATGATGTGGTTTAATTCGATGCAACGCGAAAAACCTTACCCACCTTTGACATGTACGGAATTCGCCAGAGATGGCTTAGTGCTCGAAAGAGAACCGTAACACAGGTGCTGCATGGCTGTCGTCAGCTCGTGTCGTGAGATGTTGGGTTAAGTCCCGCAACGAGCGCAACCCTTGTCATTAGTTGCTACATTCAGTTGGGCACTCTAATGAGACTGCCGGTGACAAACCGGAGGAAGGTGGGGATGACGTCAAGTCCTCATGGCCCTTATAGGTGGGGCTACACACGTCATACAATGGCTGGTACAAAGGGTTGCCAACCCGCGAGGGGGAGCTAATCCCATAAAACCAGTCGTAGTCCGGATCGCAGTCTGCAACTCGACTGCGTGAAGTCGGAATCGCTAGTAATCGTGGATCAGAATGTCACGGTGAATACGTTCCCGGGTCTTGTACACACCGCCCGTCACACCATGGGAGCGGGTTCTGCCAGAAGTAGTTAGC

>GW458-12-9-14-TSB1

GCAGTCGAACGGCAGCGCGGGAGCAATCCTGGCGGCGAGTGGCGAACGGGTGAGTAATACATCGGAACGTGCCCAATCGTGGGGGATAACGCAGCGAAAGCTGTGCTAATACCGCATACGATCTACGGATGAAAGCAGGGGATCGCAAGACCTTGCGCGAATGGAGCGGCCGATGGCAGATTAGGTAGTTGGTGGGGTAAAGGCTCACCAAGCCTTCGATCTGTAGCTGGTCTGAGAGGACGACCAGCCACACTGGGACTGAGACACGGCCCAGACTCCTACGGGAGGCAGCAGTGGGGAATTTTGGACAATGGGCGAAAGCCTGATCCAGCCATGCCGCGTGCAGGATGAAGGCCTTCGGGTTGTAAACTGCTTTTGTACGGAACGAAACGGTCTTTTCTAATACAGAAGGCTAATGACGGTACCGTAAGAATAAGCACCGGCTAACTACGTGCCAGCAGCCGCGGTAATACGTAGGGTGCAAGCGTTAATCGGAATTACTGGGCGTAAAGCGTGCGCAGGCGGTTATGTAAGACAGTTGTGAAATCCCCGGGCTCAACCTGGGAACTGCATCTGTGACTGCATAGCTAGAGTACGGTAGAGGGGGATGGAATTCCGCGTGTAGCAGTGAAATGCGTAGATATGCGGAGGAACACCGATGGCGAAGGCAATCCCCTGGACCTGTACTGACGCTCATGCACGAAAGCGTGGGGAGCAAACAGGATTAGATACCCTGGTAGTCCACGCCCTAAACGATGTCAACTGGTTGTTGGGTCTTCACTGACTCAGTAACGAAGCTAACGCGTGAAGTTGACCGCCTGGGGAGTACGGCCGCAAGGTTGAAACTCAAAGGAATTGACGGGGACCCGCACAAGCGGTGGATGATGTGGTTTAATTCGATGCAACGCGAAAAACCTTACCCACCTTTGACATGTACGGAATTCGCCAGAGATGGCTTAGTGCTCGAAAGAGAACCGTAACACAGGTGCTGCATGGCTGTCGTCAGCTCGTGTCGTGAGATGTTGGGTTAAGTCCCGCAACGAGCGCAACCCTTGTCATTAGTTGCTACATTCAGTTGGGCACTCTAATGAGACTGCCGGTGACAAACCGGAGGAAGGTGGGGATGACGTCAAGTCCTCATGGCCCTTATAGGTGGGGCTACACACGTCATACAATGGCTGGTACAAAGGGTTGCCAACCCGCGAGGGGGAGCTAATCCCATAAAACCAGTCGTAGTCCGGATCGCAGTCTGCAACTCGACTGCGTGAAGTCGGAATCGCTAGTAATCGTGGATCAGAATGTCACGGTGAATACGTTCCCGGGTCTTGTACACACCGCCCGTCACACCATGGGAGCGGGTTCTGCCAGAAGTAGTTAGC

>GW458-12-9-14-TSB2

ATGCAGTCGAGCGGCAGCACGGGTACTTGTACCTGGTGGCGAGCGGCGGACGGGTGAGTAATGCCTAGGAATCTGCCTGGTAGTGGGGGATAACGCTCGGAAACGGACGCTAATACCGCATACGTCCTACGGGAGAAAGCAGGGGACCTTCGGGCCTTGCGCTATCAGATGAGCCTAGGTCGGATTAGCTAGTTGGTGAGGTAATGGCTCACCAAGGCGACGATCCGTAACTGGTCTGAGAGGATGATCAGTCACACTGGAACTGAGACACGGTCCAGACTCCTACGGGAGGCAGCAGTGGGGAATATTGGACAATGGGCGAAAGCCTGATCCAGCCATGCCGCGTGTGTGAAGAAGGTCTTCGGATTGTAAAGCACTTTAAGTTGGGAGGAAGGGCAGTTACCTAATACGTATCTGTTTTGACGTTACCGACAGAATAAGCACCGGCTAACTCTGTGCCAGCAGCCGCGGTAATACAGAGGGTGCAAGCGTTAATCGGAATTACTGGGCGTAAAGCGCGCGTAGGTGGTTTGTTAAGTTGGATGTGAAATCCCCGGGCTCAACCTGGGAACTGCATTCAAAACTGACAAGCTAGAGTATGGTAGAGGGTGGTGGAATTTCCTGTGTAGCGGTGAAATGCGTAGATATAGGAAGGAACACCAGTGGCGAAGGCGACCACCTGGACTGATACTGACACTGAGGTGCGAAAGCGTGGGGAGCAAACAGGATTAGATACCCTGGTAGTCCACGCCGTAAACGATGTCAACTAGCCGTTGGGAGCCTTGAGCTCTTAGTGGCGCAGCTAACGCATTAAGTTGACCGCCTGGGGAGTACGGCCGCAAGGTTAAAACTCAAATGAATTGACGGGGGCCCGCACAAGCGGTGGAGCATGTGGTTTAATTCGAAGCAACGCGAAGAACCTTACCAGGCCTTGACATCCAATGAACTTTCCAGAGATGGATTGGTGCCTTCGGGAACATTGAGACAGGTGCTGCATGGCTGTCGTCAGCTCGTGTCGTGAGATGTTGGGTTAAGTCCCGTAACGAGCGCAACCCTTGTCCTTAGTTACCAGCACGNNATGGTGGGCACTCTAAGGAGACTGCCGGTGACAAACCGGAGGAAGGTGGGGATGACGTCAAGTCATCATGGCCCTTACGGCCTGGGCTACACACGTGCTACAATGGTCGGTACAGAGGGTTGCCAAGCCGCGAGGTGGAGCTAATCCCAGAAAACCGATCGTAGTCCGGATCGCAGTCTGCAACTCGACTGCGTGAAGTCGGAATCGCTAGTAATCGCGAATCAGAATGTCGCGGTGAATACGTTCCCGGGCCTTGTACACACCGCCCGTCACACCATGGGAGTGGGTTGCACCAGAAGTAGCTAGTCTAACCTTTCGG

>GW460-11-11-14-LB1

GGCCGCAAGGTGCTGACGAGTGGCGAACGGGTGAGTAATGCATCGGAACGTGCCCAGTCGTGGGGGATAACGCAGCGAAAGCTGCGCTAATACCGCATACGATCTATGGATGAAAGCGGGGGACCGTAAGGCCTCGCGCGATTGGAGCGGCCGATGTCAGATTAGGTAGTTGGTGGGGTAAAGGCTCACCAAGCCGACGATCTGTAGCTGGTCTGAGAGGACGACCAGCCACACTGGGACTGAGACACGGCCCAGACTCCTACGGGAGGCAGCAGTGGGGAATTTTGGACAATGGGCGCAAGCCTGATCCAGCAATGCCGCGTGCAGGAAGAAGGCCTTCGGGTTGTAAACTGCTTTTGTACGGAACGAAAAGGCTCTGGTTAATACCTGGGGCTCATGACGGTACCGTAAGAATAAGCACCGGCTAACTACGTGCCAGCAGCCGCGGTAATACGTAGGGTGCAAGCGTTAATCGGAATTACTGGGCGTAAAGCGTGCGCAGGCGGTTTTGTAAGACAGGCGTGAAATCCCCGGGCTCAACCTGGGAATTGCGCTTGTGACTGCAAGGCTGGAGTGCGGCAGAGGGGGATGGAATTCCGCGTGTAGCAGTGAAATGCGTAGATATGCGGAGGAACACCGATGGCGAAGGCAATCCCCTGGGCCTGCACTGACGCTCATGCACGAAAGCGTGGGGAGCAAACAGGATTAGATACCCTGGTAGTCCACGCCCTAAACGATGTCAACTGGTTGTTGGGTCTCTTCTGACTCAGTAACGAAGCTAACGCGTGAAGTTGACCGCCTGGGGAGTACGGCCGCAAGGTTGAAACTCAAAGGAATTGACGGGGACCCGCACAAGCGGTGGATGATGTGGTTTAATTCGATGCAACGCGAAAAACCTTACCCACCTTTGACATGTACGGAATTTGCCAGAGATGGCTTAGTGCTCGAAAGAGAGCCGTAACACAGGTGCTGCATGGCTGTCGTCAGCTCGTGTCGTGAGATGTTGGGTTAAGTCCCGCAACGAGCGCAACCCTTGTCATTAGTTGCTACGAAAGGGCACTCTAATGAGACTGCCGGTGACAAACCGGAGGAAGGTGGGGATGACGTCAAGTCCTCATGGCCCTTATAGGTGGGGCTACACACGTCATACAATGGCTGGTACAAAGGGTTGCCAACCCGCGAGGGGGAGCTAATCCCATAAAGCCAGTCGTAGTCCGGATCGCAGTCTGCAACTCGACTGCGTGAAGTCGGAATCGCTAGTAATCGTGGATCAGCATGTCACGGTGAATACGTTCCCGGGTCTTGTACACACCGCCCGTCACACCATGGGAGCGGGTCTCGCCAGAAGTAGTTAGCCTAACCG

>GW460-11-11-14-LB4

GGTCTTCGGATGCTGACGAGTGGCGAACGGGTGAGTAATACATCGGAACGTGCCCGAGAGTGGGGGATAACGAAGCGAAAGCTTTGCTAATACCGCATACGATCTCAGGATGAAAGCAGGGGACCGCAAGGCCTTGCGCTCACGGAGCGGCCGATGGCAGATTAGGTAGTTGGTGGGATAAAAGCTTACCAAGCCGACGATCTGTAGCTGGTCTGAGAGGACGACCAGCCACACTGGGACTGAGACACGGCCCAGACTCCTACGGGAGGCAGCAGTGGGGAATTTTGGACAATGGGCGCAAGCCTGATCCAGCCATGCCGCGTGCAGGATGAAGGCCTTCGGGTTGTAAACTGCTTTTGTACGGAACGAAAAGACTCTGGTTAATACCTGGGGTCCATGACGGTACCGTAAGAATAAGCACCGGCTAACTACGTGCCAGCAGCCGCGGTAATACGTAGGGTGCGAGCGTTAATCGGAATTACTGGGCGTAAAGCGTGCGCAGGCGGTTATATAAGACAGATGTGAAATCCCCGGGCTCAACCTGGGAACTGCATTTGTGACTGTATAGCTAGAGTACGGCAGAGGGGGATGGAATTCCGCGTGTAGCAGTGAAATGCGTAGATATGCGGAGGAACACCGATGGCGAAGGCAATCCCCTGGGCCTGTACTGACGCTCATGCACGAAAGCGTGGGGAGCAAACAGGATTAGATACCCTGGTAGTCCACGCCCTAAACGATGTCAACTGGTTGTTGGGTCTTCACTGACTCAGTAACGAAGCTAACGCGTGAAGTTGACCGCCTGGGGAGTACGGCCGCAAGGTTGAAACTCAAAGGAATTGACGGGGACCCGCACAAGCGGTGGATGATGTGGTTTAATTCGATGCAACGCGAAAAACCTTACCCACCTTTGACATGTACGGAATCCTTTAGAGATAGAGGAGTGCTCGAAAGAGAACCGTAACACAGGTGCTGCATGGCTGTCGTCAGCTCGTGTCGTGAGATGTTGGGTTAAGTCCCGCAACGAGCGCAACCCTTGTCATTAGTTGCTACATTCAGTTGGGCACTCTAATGAGACTGCCGGTGACAAACCGGAGGAAGGTGGGGATGACGTCAAGTCCTCATGGCCCTTATAGGTGGGGCTACACACGTCATACAATGGCTGGTACAGAGGGTTGCCAACCCGCGAGGGGGAGCCAATCCCATAAAGCCAGTCGTAGTCCGGATCGCAGTCTGCAACTCGACTGCGTGAAGTCGGAATCGCTAGTAATCGCGGATCAGAATGTCGCGGTGAATACGTTCCCGGGTCTTGTACNNCACCGCCCGTCACAC

>GW460-11-11-14-LB5

GCTTGCTGGATATGAAAGTGGCGCACGGGTGCGTAACGCGTATGCAACCTACCTTTATCTGGGGGATAGCCCGGAGAAATCCGGATTAATACCGCATAAAATCACAGGATGGCATTATCCAATGATCAAACATTTATGGGATGAAGATGGGCATGCGTGTCATTAGCTAGTTGGCGGGGTAACGGCCCACCAAGGCGACGATGACTAGGGGATCTGAGAGGATGGCCCCCCACACTGGTACTGAGACACGGACCAGACTCCTACGGGAGGCAGCAGTAAGGAATATTGGTCAATGGAGGCAACTCTGAACCAGCCATGCCGCGTGCAGGAAGACTGCCCTATGGGTTGTAAACTGCTTTTATCCGGGAATAAACCTATTTACGTGTAAATAGCTGAATGTACCGGAAGAATAAGGATCGGCTAACTCCGTGCCAGCAGCCGCGGTAATACGGAGGATCCAAGCGTTATCCGGATTTATTGGGTTTAAAGGGTGCGTAGGCGGCCTGTTAAGTCAGGGGTGAAAGACGGTAGCTCAACTATCGCAGTGCCCTTGATACTGATGGGCTTGAATGGACTAGAGGTAGGCGGAATGAGACAAGTAGCGGTGAAATGCATAGATATGTCTCAGAACACCGATTGCGAAGGCAGCTTACTATGGTCTTATTGACGCTGAGGCACGAAAGCGTGGGGATCAAACAGGATTAGATACCCTGGTAGTCCACGCCCTAAACGATGAACACTCGCTGTTGGCGATACACAGTCAGCGGCTAAGCGAAAGCGTTAAGTGTTCCACCTGGGGAGTACGCTCGCAAGAGTGAAACTCAAAGGAATTGACGGGGGCCCGCACAAGCGGAGGAGCATGTGGTTTAATTCGATGATACGCGAGGAACCTTACCCGGGCTTGAAAGTTAGTGAATGATCTAGAGATAGATCAGTGAGCAATCACACGAAACTAGGTGCTGCATGGCTGTCGTCAGCTCGTGCCGTGAGGTGTTGGGTTAAGTCCCGCAACGAGCGCAACCCCTATGTTTAGTTGCCAGCATGTAATGATGGGGACTCTAAACAGACTGCCTGTGCAAACAGAGAGGAAGGAGGGGACGACGTCAAGTCATCATGGCCCTTACGTCCGGGGCTACACACGTGCTACAATGGATGGTACAGAGGGCAGCTAGCCGGCAACGGTATGCGAATCTCACAAAGCCATTCACAGTTCGGATTGGGGTCTGCAACTCGACCCCATGAAGTTGGATTCGCTAGTAATCGCGTATCAGCAATGACGCGGTGAATACGTTCCCGGGCCTTGTACACACCGCCCGTCAAGCCATGGAAGTTGGGGGTACCT

>GW460-11-11-14-LB6

CTTGCTCTGGTGGCGAGTGGCGAACGGGTGAGTAATATATCGGAACGTACCCTGGAGTGGGGGATAACGTAGCGAAAGTTACGCTAATACCGCATACGATCTACGGATGAAAGTGGGGGATCGCAAGACCTCATGCTCGTGGAGCGGCCGATATCTGATTAGCTAGTTGGTAGGGTAAAAGCCTACCAAGGCATCGATCAGTAGCTGGTCTGAGAGGACGACCAGCCACACTGGAACTGAGACACGGTCCAGACTCCTACGGGAGGCAGCAGTGGGGAATTTTGGACAATGGGCGAAAGCCTGATCCAGCAATGCCGCGTGAGTGAAGAAGGCCTTCGGGTTGTAAAGCTCTTTTGTCAGGGAAGAAACGGTGAGAGCTAATATCTCTTGCTAATGACGGTACCTGAAGAATAAGCACCGGCTAACTACGTGCCAGCAGCCGCGGTAATACGTAGGGTGCAAGCGTTAATCGGAATTACTGGGCGTAAAGCGTGCGCAGGCGGTTTTGTAAGTCTGATGTGAAATCCCCGGGCTCAACCTGGGAATTGCATTGGAGACTGCAAGGCTAGAATCTGGCAGAGGGGGGTAGAATTCCACGTGTAGCAGTGAAATGCGTAGATATGTGGAGGAACACCGATGGCGAAGGCAGCCCCCTGGGTCAAGATTGACGCTCATGCACGAAAGCGTGGGGAGCAAACAGGATTAGATACCCTGGTAGTCCACGCCCTAAACGATGTCTACTAGTTGTCGGGTCTTAATTGACTTGGTAACGCAGCTAACGCGTGAAGTAGACCGCCTGGGGAGTACGGTCGCAAGATTAAAACTCAAAGGAATTGACGGGGACCCGCACAAGCGGTGGATGATGTGGATTAATTCGATGCAACGCGAAAAACCTTACCTACCCTTGACATGGCTGGAATCCCCGAGAGATTGNGGGAGTGCTCGAAAGAGAACCAGTACACAGGTGCTGCATGGCTGTCGTCAGCTCGTGTCGTGAGATGTTGGGTTAAGTCCCGCAACGAGCGCAACCCTTGTCATTAGTTGCTACGAAAGGGCACTCTAATGAGACTGCCGGTGACAAACCGGAGGAAGGTGGGGATGACGTCAAGTCCTCATGGCCCTTATGGGTAGGGCTTCACACGTCATACAATGGTACATACAGAGCGCCGCCAACCCGCGAGGGGGAGCTAATCGCAGAAAGTGTATCGTAGTCCGGATTGTAGTCTGCAACTCGACTGCATGAAGTTGGAATCGCTAGTAATCGCGGATCAGCATGTCGCGGTGAATACGTTCCCGGGTCTTGTACACACCGCCCGTCACACCATGGGAGCGGGTTTTACCAGAAGTAGGTAGC

>GW460-11-11-14-LB11

GTCGAGCGGCAGCACGGGTACTTGTACCTGGTGGCGAGCGGCGGACGGGTGAGTAATGCCTAGGAATCTGCCTGGTAGTGGGGGATAACGCTCGGAAACGGACGCTAATACCGCATACGTCCTACGGGAGAAAGCAGGGGACCTTCGGGCCTTGCGCTATCAGATGAGCCTAGGTCGGATTAGCTAGTTGGTGAGGTAATGGCTCACCAAGGCGACGATCCGTAACTGGTCTGAGAGGATGATCAGTCACACTGGAACTGAGACACGGTCCAGACTCCTACGGGAGGCAGCAGTGGGGAATATTGGACAATGGGCGAAAGCCTGATCCAGCCATGCCGCGTGTGTGAAGAAGGTCTTCGGATTGTAAAGCACTTTAAGTTGGGAGGAAGGGCAGTTACCTAATACGTATCTGTTTTGACGTTACCGACAGAATAAGCACCGGCTAACTCTGTGCCAGCAGCCGCGGTAATACAGAGGGTGCAAGCGTTAATCGGAATTACTGGGCGTAAAGCGCGCGTAGGTGGTTTGTTAAGTTGGATGTGAAATCCCCGGGCTCAACCTGGGAACTGCATTCAAAACTGACAAGCTAGAGTATGGTAGAGGGTGGTGGAATTTCCTGTGTAGCGGTGAAATGCGTAGATATAGGAAGGAACACCAGTGGCGAAGGCGACCACCTGGACTGATACTGACACTGAGGTGCGAAAGCGTGGGGAGCAAACAGGATTAGATACCCTGGTAGTCCACGCCGTAAACGATGTCAACTAGCCGTTGGGAGCCTTGAGCTCTTAGTGGCGCAGCTAACGCATTAAGTTGACCGCCTGGGGAGTACGGCCGCAAGGTTAAAACTCAAATGAATTGACGGGGGCCCGCACAAGCGGTGGAGCATGTGGTTTAATTCGAAGCAACGCGAAGAACCTTACCAGGCCTTGACATCCAATGAACTTTCCAGAGATGGATTGGTGCCTTCGGGAACATTGAGACAGGTGCTGCATGGCTGTCGTCAGCTCGTGTCGTGAGATGTTGGGTTAAGTCCCGTAACGAGCGCAACCCTTGTCCTTAGTTACCAGCACGTNATGGTGGGCACTCTAAGGAGACTGCCGGTGACAAACCGGAGGAAGGTGGGGATGACGTCAAGTCATCATGGCCCTTACGGCCTGGGCTACACACGTGCTACAATGGTCGGTACAGAGGGTTGCCAAGCCGCGAGGTGGAGCTAATCCCAGAAAACCGATCGTAGTCCGGATCGCAGTCTGCAACTCGACTGCGTGAAGTCGGAATCGCTAGTAATCGCGAATCAGAATGTCGCGGTGAATACGTTCCCGGGCCTTGTACACACCGCCCGTCACACCATGGGAGTGGGTTGCACCAGAAGTAGCTAG

>GW460-11-11-14-TSB1

GCTTGCTCTGGTGGCGAGTGGCGAACGGGTGAGTAATATATCGGAACGTACCCTGGAGTGGGGGATAACGTAGCGAAAGTTACGCTAATACCGCATACGATCTACGGATGAAAGTGGGGGATCGCAAGACCTCATGCTCGTGGAGCGGCCGATATCTGATTAGCTAGTTGGTAGGGTAAAAGCCTACCAAGGCATCGATCAGTAGCTGGTCTGAGAGGACGACCAGCCACACTGGAACTGAGACACGGTCCAGACTCCTACGGGAGGCAGCAGTGGGGAATTTTGGACAATGGGCGAAAGCCTGATCCAGCAATGCCGCGTGAGTGAAGAAGGCCTTCGGGTTGTAAAGCTCTTTTGTCAGGGAAGAAACGGTGAGAGCTAATATCTCTTGCTAATGACGGTACCTGAAGAATAAGCACCGGCTAACTACGTGCCAGCAGCCGCGGTAATACGTAGGGTGCAAGCGTTAATCGGAATTACTGGGCGTAAAGCGTGCGCAGGCGGTTTTGTAAGTCTGATGTGAAATCCCCGGGCTCAACCTGGGAATTGCATTGGAGACTGCAAGGCTAGAATCTGGCAGAGGGGGGTAGAATTCCACGTGTAGCAGTGAAATGCGTAGATATGTGGAGGAACACCGATGGCGAAGGCAGCCCCCTGGGTCAAGATTGACGCTCATGCACGAAAGCGTGGGGAGCAAACAGGATTAGATACCCTGGTAGTCCACGCCCTAAACGATGTCTACTAGTTGTCGGGTCTTAATTGACTTGGTAACGCAGCTAACGCGTGAAGTAGACCGCCTGGGGAGTACGGTCGCAAGATTAAAACTCAAAGGAATTGACGGGGACCCGCACAAGCGGTGGATGATGTGGATTAATTCGATGCAACGCGAAAAACCTTACCTACCCTTGACATGGCTGGAATCCCCGAGAGATTGGGGAGTGCTCGAAAGAGAACCAGTACACAGGTGCTGCATGGCTGTCGTCAGCTCGTGTCGTGAGATGTTGGGTTAAGTCCCGCAACGAGCGCAACCCTTGTCATTAGTTGCTACGAAAGGGCACTCTAATGAGACTGCCGGTGACAAACCGGAGGAAGGTGGGGATGACGTCAAGTCCTCATGGCCCTTATGGGTAGGGCTTCACACGTCATACAATGGTACATACAGAGCGCCGCCAACCCGCGAGGGGGAGCTAATCGCAGAAAGTGTATCGTAGTCCGGATTGTAGTCTGCAACTCGACTGCATGAAGTTGGAATCGCTAGTAATCGCGGATCAGCATGTCGCGGTGAATACGTTCCCGGGTCTTGTACACACCGCCCGTCACACCATGGGAGCGGGTTTTACCAGAAGTAGGTAGC

>GW460-11-11-14-TSB3

ACACGAGTGGCGCACGGGTGAGTAACGCGTGGATATCTGCCTTTTGGTTCGGAATAACTCCGGGAAACTGGAGCTAATACCGGATGGTTCCTACGGGATAAAGATTTATCGCCAAAAGATGAGTCCGCGTACGATTAGCTAGTTGGTGAGGTAATGGCTCACCAAGGCGACGATCGTTAGCTGGTCTGAGAGGACGATCAGCCACACTGGGACTGAGACACGGCCCAGACTCCTACGGGAGGCAGCAGTGGGGAATATTGGACAATGGGGGCAACCCTGATCCAGCCATGCCGCGTGAGTGATGAAGGCCTTCGGGTTGTAAAGCTCTTTTGGCGGGGACGATGATGACGGTACCCGCAGAATAAGCCCCGGCTAACTTCGTGCCAGCAGCCGCGGTAAGACGAAGGGGGCTAGCGTTGTTCGGAATTACTGGGCGTAAAGCGAGTGTAGGTGGTTGTCATAGTCAGGTGTGAAAGCCTTGAGCTCAACTCAAGAAATGCACTTGATACTGGGCGACTAGAGGACCGGAGAGGATAGTGGAATTCCCAGTGTAGTGGTGAAATACGTAGATATTGGGAAGAACACCAGTGGCGAAGGCGGCTATCTGGACGGTTTCTGACACTAAGACTCGAAAGCGTGGGGAGCAAACAGGATTAGATACCCTGGTAGTCCACGCCGTAAACGATGGGTGCTAGACGTTGGCGAGCTTGCTCGTCAGTGTCGCAGCTAACGCGTTAAGCACCCCGCCTGGGGAGTACGGCCGCAAGGTTGAAACTCAAAGGAATTGACGGGGGCCCGCACAAGCGGTGGAGCATGTGGTTCAATTCGACGCAACGCGCAGAACCTTACCAGCCCTTGACATGTGACTCGCCGGCTCCAGAGATGGAGCCTTCGGTTCGGCCGGAGTCAGCACAGGTGCTGCATGGCTGTCGTCAGCTCGTGTCGTGAGATGTTGGGTTAAGTCCCGCAACGAGCGCAACCCTCGTCTCCAGTTGCCATCAGGTTAAGCTGGGCACTTTGGAGAAACTGCCGGTGACAAGCCGGAGGAAGGTGGGGATGACGTCAAGTCCTCATGGCCCTTACGGGCTGGGCTACACACGTGCTACAATGGCGGTGACAGTGGGATGCGAAGGGGCGACCCGGAGCCGATCCCCAAAAACCGTCCCAGTTCGGATTGCACTCTGCAACTCGAGTGCATGAAGTTGGAATCGCTAGTAATCGCGGATCAGCATGCCGCGGTGAATACGTTCCCGGGCCTTGTACACACCGCCCGTCACGCCATGGGAGTTGGTTCTACCCGAAAACGGTGCG

>GW460-11-11-14-TSB4

GCTTGCTGGATATGAAAGTGGCGCACGGGTGCGTAACGCGTATGCAACCTACCTTTATCTGGGGGATAGCCCGGAGAAATCCGGATTAATACCGCATAAAATCACAGGATGGCATTATCCAATGATCAAACATTTATGGGATGAAGATGGGCATGCGTGTCATTAGCTAGTTGGCGGGGTAACGGCCCACCAAGGCGACGATGACTAGGGGATCTGAGAGGATGGCCCCCCACACTGGTACTGAGACACGGACCAGACTCCTACGGGAGGCAGCAGTAAGGAATATTGGTCAATGGAGGCAACTCTGAACCAGCCATGCCGCGTGCAGGAAGACTGCCCTATGGGTTGTAAACTGCTTTTATCCGGGAATAAACCTATTTACGTGTAAATAGCTGAATGTACCGGAAGAATAAGGATCGGCTAACTCCGTGCCAGCAGCCGCGGTAATACGGAGGATCCAAGCGTTATCCGGATTTATTGGGTTTAAAGGGTGCGTAGGCGGCCTGTTAAGTCAGGGGTGAAAGACGGTAGCTCAACTATCGCAGTGCCCTTGATACTGATGGGCTTGAATGGACTAGAGGTAGGCGGAATGAGACAAGTAGCGGTGAAATGCATAGATATGTCTCAGAACACCGATTGCGAAGGCAGCTTACTATGGTCTTATTGACGCTGAGGCACGAAAGCGTGGGGATCAAACAGGATTAGATACCCTGGTAGTCCACGCCCTAAACGATGAACACTCGCTGTTGGCGATACACAGTCAGCGGCTAAGCGAAAGCGTTAAGTGTTCCACCTGGGGAGTACGCTCGCAAGAGTGAAACTCAAAGGAATTGACGGGGGCCCGCACAAGCGGAGGAGCATGTGGTTTAATTCGATGATACGCGAGGAACCTTACCCGGGCTTGAAAGTTAGTGAATGATCTAGAGATAGATCAGTGAGCAATCACACGAAACTAGGTGCTGCATGGCTGTCGTCAGCTCGTGCCGTGAGGTGTTGGGTTAAGTCCCGCAACGAGCGCAACCCCTATGTTTAGTTGCCAGCATGTAATGATGGGGACTCTAAACAGACTGCCTGTGCAAACAGAGAGGAAGGAGGGGACGACGTCAAGTCATCATGGCCCTTACGTCCGGGGCTACACACGTGCTACAATGGATGGTACAGAGGGCAGCTAGCCGGCAACGGTATGCGAATCTCACAAAGCCATTCACAGTTCGGATTGGGGTCTGCAACTCGACCCCATGAAGTTGGATTCGCTAGTAATCGCGTATCAGCAATGACGCGGTGAATACGTTCCCGGGCCTTGTACACACCGCCCGTCAAGCCATGGAAGTTGGGGGTACCTAAAGTA

>GW460-11-11-14-TSB5

CTAGTGGCGCACGGGTGCGTAACGCGTGGGAATCTACCCTTGGGTTCGGAATAACTTCGGGAAACTGAAGCTAATACCGGATGATGTCGAAAGACCAAAGATTTATCGCCCAGGGATGAGCCCGCGTAGGATTAGCTAGTTGGTGGGGTAAAGGCTCACCAAGGCAACGATCCTTAGCTGGTCTGAGAGGATGATCAGCCACACTGGGACTGAGACACGGCCCAGACTCCTACGGGAGGCAGCAGTAGGGAATATTGGACAATGGGGGCAACCCTGATCCAGCAATGCCGCGTGAGTGATGAAGGCCTTAGGGTTGTAAAGCTCTTTTACCCGAGATGATAATGACAGTATCGGGAGAATAAGCTCCGGCTAACTCCGTGCCAGCAGCCGCGGTAATACGGAGGGAGCTAGCGTTGTTCGGAATTACTGGGCGTAAAGCGCACGTAGGCGGCGATTTAAGTCAGAGGTGAAAGCCCGGGGCTCAACCCCGGAACTGCCTTTGAGACTGGATTGCTAGAATCTTGGAGAGGCGAGTGGAATTCCGAGTGTAGAGGTGAAATTCGTAGATATTCGGAAGAACACCAGTGGCGAAGGCGGCTCGCTGGACAAGTATTGACGCTGAGGTGCGAAAGCGTGGGGAGCAAACAGGATTAGATACCCTGGTAGTCCACGCCGTAAACGATGATAACTAGCTGCCGGGGCACATGGTGTTTCGGTGGCGCAGCTAACGCATTAAGTTATCCGCCTGGGGAGTACGGTCGCAAGATTAAAACTCAAAGGAATTGACGGGGGCCTGCACAAGCGGTGGAGCATGTGGTTTAATTCGAAGCAACGCGCAGAACCTTACCAGCGTTTGACATCCTCATCGCGATTTCCAGAGATGGATTTCTTCAGTTCGGCTGGATGAGTGACAGGTGCTGCATGGCTGTCGTCAGCTCGTGTCGTGAGATGTTGGGTTAAGTCCCGCAACGAGCGCAACCCTCGCCTTTAGTTGCCAGCATTTGGTTGGGTACTCTAAAGGAACCGCCGGTGATAAGCCGGAGGAAGGTGGGGATGACGTCAAGTCCTCATGGCCCTTACGCGCTGGGCTACACACGTGCTACAATGGCGACTACAGTGGGCTGCAACCGTGCGAGCGGTAGCTAATCTCCAAAAGTCGTCTCAGTTCGGATTGTTCTCTGCAACTCGAGAGCATGAAGGCGGAATCGCTAGTAATCGCGGATCAGCATGCCGCGGTGAATACGTTCCCAGGCCTTGTACACACCGCCCGTCACACCATGGGATTTGGATTCACCCGAAGGCAC

>GW460-11-11-14-TSB6

AGACCTTCGGGTCTAGTGGCGCACGGGTGCGTAACGCGTGGGAATCTACCCTTGGGTTCGGAATAACGTCGGGAAACTGACGCTAATACCGGATGATGACGAAAGTCCAAAGATTTATCGCCCAGGGATGAGCCCGCGTAGGATTAGCTAGTTGGTGGGGTAAAGGCTCACCAAGGCTACGATCCTTAGCTGGTCTGAGAGGATGATCAGCCACACTGGGACTGAGACACGGCCCAGACTCCTACGGGAGGCAGCAGTAGGGAATATTGGACAATGGGGGCAACCCTGATCCAGCAATGCCGCGTGAGTGATGAAGGCCTTAGGGTTGTAAAGCTCTTTTACCCGAGATGATAATGACAGTATCGGGAGAATAAGCTCCGGCTAACTCCGTGCCAGCAGCCGCGGTAATACGGAGGGAGCTAGCGTTGTTCGGAATTACTGGGCGTAAAGCGCACGTAGGCGGCGATTTAAGTCAGAGGTGAAAGCCCGGGGCTCAACCCCGGAACTGCCTTTGAGACTGGATTGCTAGAATCTTGGAGAGGCGGGTGGAATTCCGAGTGTAGAGGTGAAATTCGTAGATATTCGGAAGAACACCAGTGGCGAAGGCGGCCCGCTGGACAAGTATTGACGCTGAGGTGCGAAAGCGTGGGGAGCAAACAGGATTAGATACCCTGGTAGTCCACGCCGTAAACGATGATAACTAGCTGCTGGGGCACATGGTGTTTCAGTGGCGCAGCTAACGCATTAAGTTATCCGCCTGGGGAGTACGGTCGCAAGATTAAAACTCAAAGGAATTGACGGGGGCCTGCACAAGCGGTGGAGCATGTGGTTTAATTCGAAGCAACGCGCAGAACCTTACCAACGTTTGACATCCCTATCGCGGATCGTGGAGACACTTTCCTTCAGTTCGGCTGGATAGGTGACAGGTGCTGCATGGCTGTCGTCAGCTCGTGTCGTGAGATGTTGGGTTAAGTCCCGCAACGAGCGCAACCCTCGCCTTTAGTTGCCATCATTTAGTTGGGTACTCTAAAGGAACCGCCGGTGATAAGCCGGAGGAAGGTGGGGATGACGTCAAGTCCTCATGGCCCTTACGCGTTGGGCTACACACGTGCTACAATGGCGACTACAGTGGGCAGCCACTCCGCGAGGAGGAGCTAATCTCCAAAAGTCGTCTCAGTTCGGATCGTTCTCTGCAACTCGAGAGCGTGAAGGCGGAATCGCTAGTAATCGCGGATCAGCATGCCGCGGTGAATACGTTCCCAGGCCTTGTACACACCGCCCGTCACACCATGGGAGTTGGATTCACTCGAAGGCGTTGAGCTAACCGC

>GW460-11-11-14-TSB7

GGATGCTGACGAGTGGCGAACGGGTGAGTAATACATCGGAACGTGCCCGAGAGTGGGGGATAACGAAGCGAAAGCTTTGCTAATACCGCATACGATCTCAGGATGAAAGCAGGGGACCGCAAGGCCTTGCGCTCACGGAGCGGCCGATGGCAGATTAGGTAGTTGGTGGGATAAAAGCTTACCAAGCCGACGATCTGTAGCTGGTCTGAGAGGACGACCAGCCACACTGGGACTGAGACACGGCCCAGACTCCTACGGGAGGCAGCAGTGGGGAATTTTGGACAATGGGCGCAAGCCTGATCCAGCCATGCCGCGTGCAGGATGAAGGCCTTCGGGTTGTAAACTGCTTTTGTACGGAACGAAAAGACTCTGGTTAATACCTGGGGTCCATGACGGTACCGTAAGAATAAGCACCGGCTAACTACGTGCCAGCAGCCGCGGTAATACGTAGGGTGCGAGCGTTAATCGGAATTACTGGGCGTAAAGCGTGCGCAGGCGGTTATATAAGACAGATGTGAAATCCCCGGGCTCAACCTGGGAACTGCATTTGTGACTGTATAGCTAGAGTACGGCAGAGGGGGATGGAATTCCGCGTGTAGCAGTGAAATGCGTAGATATGCGGAGGAACACCGATGGCGAAGGCAATCCCCTGGGCCTGTACTGACGCTCATGCACGAAAGCGTGGGGAGCAAACAGGATTAGATACCCTGGTAGTCCACGCCCTAAACGATGTCAACTGGTTGTTGGGTCTTCACTGACTCAGTAACGAAGCTAACGCGTGAAGTTGACCGCCTGGGGAGTACGGCCGCAAGGTTGAAACTCAAAGGAATTGACGGGGACCCGCACAAGCGGTGGATGATGTGGTTTAATTCGATGCAACGCGAAAAACCTTACCCACCTTTGACATGTACGGAATCCTTTAGAGATAGAGGAGTGCTCGAAAGAGAACCGTAACACAGGTGCTGCATGGCTGTCGTCAGCTCGTGTCGTGAGATGTTGGGTTAAGTCCCGCAACGAGCGCAACCCTTGTCATTAGTTGCTACATTCAGTTGGGCACTCTAATGAGACTGCCGGTGACAAACCGGAGGAAGGTGGGGATGACGTCAAGTCCTCATGGCCCTTATAGGTGGGGCTACACACGTCATACAATGGCTGGTACAGAGGGTTGCCAACCCGCGAGGGGGAGCCAATCCCATAAAGCCAGTCGTAGTCCGGATCGCAGTCTGCAACTCGACTGCGTGAAGTCGGAATCGCTAGTAATCGCGGATCAGAATGTCGCGGTGAATACGTTCCCGGGTCTTGTACACACCGCCCGTCACACCATGGGAGCGGGTTCTGCCAGAAGTAGTTAGCCTAA

>GW460-12-1-14-LB1

CAATCCTGGCGGCGAGTGGCGAACGGGTGAGTAATACATCGGAACGTGCCCAATCGTGGGGGATAACGCAGCGAAAGCTGTGCTAATACCGCATACGATCTACGGATGAAAGCAGGGGATCGCAAGACCTTGCGCGAATGGAGCGGCCGATGGCAGATTAGGTAGTTGGTGAGGTAAAGGCTCACCAAGCCTTCGATCTGTAGCTGGTCTGAGAGGACGACCAGCCACACTGGGACTGAGACACGGCCCAGACTCCTACGGGAGGCAGCAGTGGGGAATTTTGGACAATGGGCGAAAGCCTGATCCAGCCATGCCGCGTGCAGGATGAAGGCCTTCGGGTTGTAAACTGCTTTTGTACGGAACGAAACGGTCTTTTCTAATACAGAAGGCTAATGACGGTACCGTAAGAATAAGCACCGGCTAACTACGTGCCAGCAGCCGCGGTAATACGTAGGGTGCAAGCGTTAATCGGAATTACTGGGCGTAAAGCGTGCGCAGGCGGTTATGTAAGACAGTTGTGAAATCCCCGGGCTCAACCTGGGAACTGCATCTGTGACTGCATAGCTAGAGTACGGTAGAGGGGGATGGAATTCCGCGTGTAGCAGTGAAATGCGTAGATATGCGGAGGAACACCGATGGCGAAGGCAATCCCCTGGACCTGTACTGACGCTCATGCACGAAAGCGTGGGGAGCAAACAGGATTAGATACCCTGGTAGTCCACGCCCTAAACGATGTCAACTGGTTGTTGGGTCTTCACTGACTCAGTAACGAAGCTAACGCGTGAAGTTGACCGCCTGGGGAGTACGGCCGCAAGGTTGAAACTCAAAGGAATTGACGGGGACCCGCACAAGCGGTGGATGATGTGGTTTAATTCGATGCAACGCGAAAAACCTTACCCACCTTTGACATGTACGGAATTCGCCAGAGATGGCTTAGTGCTCGAAAGAGAACCGTAACACAGGTGCTGCATGGCTGTCGTCAGCTCGTGTCGTGAGATGTTGGGTTAAGTCCCGCAACGAGCGCAACCCTTGTCATTAGTTGCTACATTCAGTTGGGCACTCTAATGAGACTGCCGGTGACAAACCGGAGGAAGGTGGGGATGACGTCAAGTCCTCATGGCCCTTATAGGTGGGGCTACACACGTCATACAATGGCTGGTACAAAGGGTTGCCAACCCGCGAGGGGGAGCTAATCCCATAAAACCAGTCGTAGTCCGGATCGCAGTCTGCAACTCGACTGCGTGAAGTCGGAATCGCTAGTAATCGTGGATCAGAATGTCACGGTGAATACGTTCCCGGGTCTTGTACACACCGCCCGTCACACCATGGGAGCGGGTTCTGCCAGAAGTAG

>GW460-12-1-14-LB2

GGTCTTCGGATGCTGACGAGTGGCGAACGGGTGAGTAATACATCGGAACGTGCCCGAGAGTGGGGGATAACGAAGCGAAAGCTTTGCTAATACCGCATACGATCTCAGGATGAAAGCAGGGGACCGCAAGGCCTTGCGCTCACGGAGCGGCCGATGGCAGATTAGGTAGTTGGTGGGATAAAAGCTTACCAAGCCGACGATCTGTAGCTGGTCTGAGAGGACGACCAGCCACACTGGGACTGAGACACGGCCCAGACTCCTACGGGAGGCAGCAGTGGGGAATTTTGGACAATGGGCGCAAGCCTGATCCAGCCATGCCGCGTGCAGGATGAAGGCCTTCGGGTTGTAAACTGCTTTTGTACGGAACGAAAAGACTCTGGTTAATACCTGGGGTCCATGACGGTACCGTAAGAATAAGCACCGGCTAACTACGTGCCAGCAGCCGCGGTAATACGTAGGGTGCGAGCGTTAATCGGAATTACTGGGCGTAAAGCGTGCGCAGGCGGTTATATAAGACAGATGTGAAATCCCCGGGCTCAACCTGGGAACTGCATTTGTGACTGTATAGCTAGAGTACGGCAGAGGGGGATGGAATTCCGCGTGTAGCAGTGAAATGCGTAGATATGCGGAGGAACACCGATGGCGAAGGCAATCCCCTGGGCCTGTACTGACGCTCATGCACGAAAGCGTGGGGAGCAAACAGGATTAGATACCCTGGTAGTCCACGCCCTAAACGATGTCAACTGGTTGTTGGGTCTTCACTGACTCAGTAACGAAGCTAACGCGTGAAGTTGACCGCCTGGGGAGTACGGCCGCAAGGTTGAAACTCAAAGGAATTGACGGGGACCCGCACAAGCGGTGGATGATGTGGTTTAATTCGATGCAACGCGAAAAACCTTACCCACCTTTGACATGTACGGAATCCTTTAGAGATAGAGGAGTGCTCGAAAGAGAACCGTAACACAGGTGCTGCATGGCTGTCGTCAGCTCGTGTCGTGAGATGTTGGGTTAAGTCCCGCAACGAGCGCAACCCTTGTCATTAGTTGCTACATTCAGTTGGGCACTCTAATGAGACTGCCGGTGACAAACCGGAGGAAGGTGGGGATGACGTCAAGTCCTCATGGCCCTTATAGGTGGGGCTACACACGTCATACAATGGCTGGTACAGAGGGTTGCCAACCCGCGAGGGGGAGCCAATCCCATAAAGCCAGTCGTAGTCCGGATCGCAGTCTGCAACTCGACTGCGTGAAGTCGGAATCGCTAGTAATCGCGGATCAGAATGTCGCGGTGAATACGTTCCCGGGTCTTGTACACACCGCCCGTCACACCATGGGAGCGGGTTCTGCCAGAAGTAGTTAG

>GW460-12-1-14-LB3

GCAGTCGAGCGGCAGCACGGGTACTTGTACCTGGTGGCGAGCGGCGGACGGGTGAGTAATGCCTAGGAATCTGCCTGGTAGTGGGGGATAACGCTCGGAAACGGACGCTAATACCGCATACGTCCTACGGGAGAAAGCAGGGGACCTTCGGGCCTTGCGCTATCAGATGAGCCTAGGTCGGATTAGCTAGTTGGTGAGGTAATGGCTCACCAAGGCGACGATCCGTAACTGGTCTGAGAGGATGATCAGTCACACTGGAACTGAGACACGGTCCAGACTCCTACGGGAGGCAGCAGTGGGGAATATTGGACAATGGGCGAAAGCCTGATCCAGCCATGCCGCGTGTGTGAAGAAGGTCTTCGGATTGTAAAGCACTTTAAGTTGGGAGGAAGGGCAGTTACCTAATACGTATCTGTTTTGACGTTACCGACAGAATAAGCACCGGCTAACTCTGTGCCAGCAGCCGCGGTAATACAGAGGGTGCAAGCGTTAATCGGAATTACTGGGCGTAAAGCGCGCGTAGGTGGTTTGTTAAGTTGGATGTGAAATCCCCGGGCTCAACCTGGGAACTGCATTCAAAACTGACAAGCTAGAGTATGGTAGAGGGTGGTGGAATTTCCTGTGTAGCGGTGAAATGCGTAGATATAGGAAGGAACACCAGTGGCGAAGGCGACCACCTGGACTGATACTGACACTGAGGTGCGAAAGCGTGGGGAGCAAACAGGATTAGATACCCTGGTAGTCCACGCCGTAAACGATGTCAACTAGCCGTTGGGAGCCTTGAGCTCTTAGTGGCGCAGCTAACGCATTAAGTTGACCGCCTGGGGAGTACGGCCGCAAGGTTAAAACTCAAATGAATTGACGGGGGCCCGCACAAGCGGTGGAGCATGTGGTTTAATTCGAAGCAACGCGAAGAACCTTACCAGGCCTTGACATCCAATGAACTTTCCAGAGATGGATTGGTGCCTTCGGGAACATTGAGACAGGTGCTGCATGGCTGTCGTCAGCTCGTGTCGTGAGATGTTGGGTTAAGTCCCGTAACGAGCGCAACCCTTGTCCTTAGTTACCAGCACGTNATGGTGGGCACTCTAAGGAGACTGCCGGTGACAAACCGGAGGAAGGTGGGGATGACGTCAAGTCATCATGGCCCTTACGGCCTGGGCTACACACGTGCTACAATGGTCGGTACAGAGGGTTGCCAAGCCGCGAGGTGGAGCTAATCCCAGAAAACCGATCGTAGTCCGGATCGCAGTCTGCAACTCGACTGCGTGAAGTCGGAATCGCTAGTAATCGCGAATCAGAATGTCGCGGTGAATACGTTCCCGGGCCTTGTACACACCGCCCGTCACACCATGGGAGT

>GW460-12-1-14-LB5

CTTGCTCTGGTGGCGAGTGGCGAACGGGTGAGTAATATATCGGAACGTACCCTGGAGTGGGGGATAACGTAGCGAAAGTTACGCTAATACCGCATACGATCTAAGGATGAAAGTGGGGGATCGCAAGACCTCATGCTCGTGGAGCGGCCGATATCTGATTAGCTAGTTGGTAGGGTAAAAGCCTACCAAGGCATCGATCAGTAGCTGGTCTGAGAGGACGACCAGCCACACTGGAACTGAGACACGGTCCAGACTCCTACGGGAGGCAGCAGTGGGGAATTTTGGACAATGGGCGAAAGCCTGATCCAGCAATGCCGCGTGAGTGAAGAAGGCCTTCGGGTTGTAAAGCTCTTTTGTCAGGGAAGAAACGGTGAGAGCTAATATCTCTTGCTAATGACGGTACCTGAAGAATAAGCACCGGCTAACTACGTGCCAGCAGCCGCGGTAATACGTAGGGTGCAAGCGTTAATCGGAATTACTGGGCGTAAAGCGTGCGCAGGCGGTTTTGTAAGTCTGATGTGAAATCCCCGGGCTCAACCTGGGAATTGCATTGGAGACTGCAAGGCTAGAATCTGGCAGAGGGGGGTAGAATTCCACGTGTAGCAGTGAAATGCGTAGATATGTGGAGGAACACCGATGGCGAAGGCAGCCCCCTGGGTCAAGATTGACGCTCATGCACGAAAGCGTGGGGAGCAAACAGGATTAGATACCCTGGTAGTCCACGCCCTAAACGATGTCTACTAGTTGTCGGGTCTTAATTGACTTGGTAACGCAGCTAACGCGTGAAGTAGACCGCCTGGGGAGTACGGTCGCAAGATTAAAACTCAAAGGAATTGACGGGGACCCGCACAAGCGGTGGATGATGTGGATTAATTCGATGCAACGCGAAAAACCTTACCTACCCTTGACATGGCTGGAATCCCCGAGAGATTGGGGAGTGCTCGAAAGAGAACCAGTACACAGGTGCTGCATGGCTGTCGTCAGCTCGTGTCGTGAGATGTTGGGTTAAGTCCCGCAACGAGCGCAACCCTTGTCATTAGTTGCTACGAAAGGGCACTCTAATGAGACTGCCGGTGACAAACCGGAGGAAGGTGGGGATGACGTCAAGTCCTCATGGCCCTTATGGGTAGGGCTTCACACGTCATACAATGGTACATACAGAGCGCCGCCAACCCGCGAGGGGGAGCTAATCGCAGAAAGTGTATCGTAGTCCGGATTGTAGTCTGCAACTCGACTGCATGAAGTTGGAATCGCTAGTAATCGCGGATCAGCATGTCGCGGTGAATACGTTCCCGGGTCTTGTACACACCGCCCGTCACACCATGGGAGCGGGTTTTACCAGAAGTAGGTAGC

>GW460-12-1-14-TSB1

CTTCGGATGCTGACGAGTGGCGAACGGGTGAGTAATACATCGGAACGTGCCCGAGAGTGGGGGATAACGAAGCGAAAGCTTTGCTAATACCGCATACGATCTCAGGATGAAAGCAGGGGACCGCAAGGCCTTGCGCTCACGGAGCGGCCGATGGCAGATTAGGTAGTTGGTGGGATAAAAGCTTACCAAGCCGACGATCTGTAGCTGGTCTGAGAGGACGACCAGCCACACTGGGACTGAGACACGGCCCAGACTCCTACGGGAGGCAGCAGTGGGGAATTTTGGACAATGGGCGCAAGCCTGATCCAGCCATGCCGCGTGCAGGATGAAGGCCTTCGGGTTGTAAACTGCTTTTGTACGGAACGAAAAGACTCTGGTTAATACCTGGGGTCCATGACGGTACCGTAAGAATAAGCACCGGCTAACTACGTGCCAGCAGCCGCGGTAATACGTAGGGTGCGAGCGTTAATCGGAATTACTGGGCGTAAAGCGTGCGCAGGCGGTTATATAAGACAGATGTGAAATCCCCGGGCTCAACCTGGGAACTGCATTTGTGACTGTATAGCTAGAGTACGGCAGAGGGGGATGGAATTCCGCGTGTAGCAGTGAAATGCGTAGATATGCGGAGGAACACCGATGGCGAAGGCAATCCCCTGGGCCTGTACTGACGCTCATGCACGAAAGCGTGGGGAGCAAACAGGATTAGATACCCTGGTAGTCCACGCCCTAAACGATGTCAACTGGTTGTTGGGTCTTCACTGACTCAGTAACGAAGCTAACGCGTGAAGTTGACCGCCTGGGGAGTACGGCCGCAAGGTTGAAACTCAAAGGAATTGACGGGGACCCGCACAAGCGGTGGATGATGTGGTTTAATTCGATGCAACGCGAAAAACCTTACCCACCTTTGACATGTACGGAATCCTTTAGAGATAGAGGAGTGCTCGAAAGAGAACCGTAACACAGGTGCTGCATGGCTGTCGTCAGCTCGTGTCGTGAGATGTTGGGTTAAGTCCCGCAACGAGCGCAACCCTTGTCATTAGTTGCTACATTCAGTTGGGCACTCTAATGAGACTGCCGGTGACAAACCGGAGGAAGGTGGGGATGACGTCAAGTCCTCATGGCCCTTATAGGTGGGGCTACACACGTCATACAATGGCTGGTACAGAGGGTTGCCAACCCGCGAGGGGGAGCCAATCCCATAAAGCCAGTCGTAGTCCGGATCGCAGTCTGCAACTCGACTGCGTGAAGTCGGAATCGCTAGTAATCGCGGATCAGAATGTCGCGGTGAATACGTTCCCGGGTCTTGTACACACCGCCCGTCACACCATGGGAGCGGGTTCTGCCAGAAGTAGTTAGCCTAACCGTAAGG

>GW460-12-1-14-TSB2

GTCGAACGATGACCTCCAGCTTGCTGGGGTGATTAGTGGCGAACGGGTGAGTAACACGTGAGTAACCTGCCCTTAACTCTGGGATAAGCCTGGGAAACTGGGTCTAATACCGGATATGACTCCTCATCGCATGGTGGGGGGTGGAAAGCTTTTTGTGGTTTTGGATGGACTCGCGGCCTATCAGCTTGTTGGTGAGGTAATGGCTCACCAAGGCGACGACGGGTAGCCGGCCTGAGAGGGTGACCGGCCACACTGGGACTGAGACACGGCCCAGACTCCTACGGGAGGCAGCAGTGGGGAATATTGCACAATGGGCGAAAGCCTGATGCAGCGACGCCGCGTGAGGGATGACGGCCTTCGGGTTGTAAACCTCTTTCAGTAGGGAAGAAGCGAAAGTGACGGTACCTGCAGAAGAAGCGCCGGCTAACTACGTGCCAGCAGCCGCGGTAATACGTAGGGCGCAAGCGTTATCCGGAATTATTGGGCGTAAAGAGCTCGTAGGCGGTTTGTCGCGTCTGCCGTGAAAGTCCGGGGCTCAACTCCGGATCTGCGGTGGGTACGGGCAGACTAGAGTGATGTAGGGGAGACTGGAATTCCTGGTGTAGCGGTGAAATGCGCAGATATCAGGAGGAACACCGATGGCGAAGGCAGGTCTCTGGGCATTAACTGACGCTGAGGAGCGAAAGCATGGGGAGCGAACAGGATTAGATACCCTGGTAGTCCATGCCGTAAACGTTGGGCACTAGGTGTGGGGGACATTCCACGTTTTCCGCGCCGTAGCTAACGCATTAAGTGCCCCGCCTGGGGAGTACGGCCGCAAGGCTAAAACTCAAAGGAATTGACGGGGGCCCGCACAAGCGGCGGAGCATGCGGATTAATTCGATGCAACGCGAAGAACCTTACCAAGGCTTGACATGGGCCGGACCGGGCTGGAAACAGTCCTTCCCCTTTGGGGCCGGTTCACAGGTGGTGCATGGTTGTCGTCAGCTCGTGTCGTGAGATGTTGGGTTAAGTCCCGCAACGAGCGCAACCCTCGTTCCATGTTGCCAGCGCGTAATGGCGGGGACTCATGGGAGACTGCCGGGGTCAACTCGGAGGAAGGTGGGGACGACGTCAAATCATCATGCCCCTTATGTCTTGGGCTTCACGCATGCTACAATGGCCGGTACAAAGGGTTGCGATACTGTGAGGTGGAGCTAATCCCAAAAAGCCGGTCTCAGTTCGGATTGGGGTCTGCAACTCGACCCCATGAAGTCGGAGTCGCTAGTAATCGCAGATCAGCAACGCTGCGGTGAATACGTTCCCGGGCCTTGTACACACCGCCCGTCAAGTCACGAAAGTTGGTAACACCCGAAGCCGGTGGCCT

>GW460-12-1-14-TSB5

GCTTGCTCCGCTGACGAGTGGCGAACGGGTGAGTAATATATCGGAACGTACCCAAGAGTGGGGGATAACGTAGCGAAAGTTACGCTAATACCGCATACGATCTAAGGATGAAAGCAGGGGACCGCAAGGCCTTGTGCTCCTGGAGCGGCCGATATCTGATTAGCTAGTTGGTAGGGTAAAGGCCTACCAAGGCATCGATCAGTAGCTGGTCTGAGAGGACGACCAGCCACACTGGAACTGAGACACGGTCCAGACTCCTACGGGAGGCAGCAGTGGGGAATTTTGGACAATGGGCGCAAGCCTGATCCAGCAATGCCGCGTGAGTGAAGAAGGCCTTCGGGTTGTAAAGCTCTTTTGTCAGGGAAGAAACGGGTGAGGCTAATATCNNCATCTAATGACGGTACCTGAAGAATAAGCACCGGCTAACTACGTGCCAGCAGCCGCGGTAATACGTAGGGTGCAAGCGTTAATCGGAATTACTGGGCGTAAAGCGTGCGCAGGCGGTTTTGTAAGTCTGTCGTGAAATCCCCGGGCTCAACCTGGGAATGGCGATGGAGACTGCAAGGCTAGAATCTGGCAGAGGGGGGTAGAATTCCACGTGTAGCAGTGAAATGCGTAGAGATGTGGAGGAACACCGATGGCGAAGGCAGCCCCCTGGGTCAAGATTGACGCTCATGCACGAAAGCGTGGGGAGCAAACAGGATTAGATACCCTGGTAGTCCACGCCCTAAACGATGTCTACTAGTTGTCGGGTCTTAATTGACTTGGTAACGCAGCTAACGCGTGAAGTAGACCGCCTGGGGAGTACGGTCGCAAGATTAAAACTCAAAGGAATTGACGGGGACCCGCACAAGCGGTGGATGATGTGGATTAATTCGATGCAACGCGAAAAACCTTACCTACCCTTGACATGGAAGGAATCCCCGAGAGATTGGGGAGTGCCCGAAAGGGAACCTTTACACAGGTGCTGCATGGCTGTCGTCAGCTCGTGTCGTGAGATGTTGGGTTAAGTCCCGCAACGAGCGCAACCCTTGTCATTAGTTGCTACGAAAGGGCACTCTAATGAGACTGCCGGTGACAAACCGGAGGAAGGTGGGGATGACGTCAAGTCCTCATGGCCCTTATGGGTAGGGCTTCACACGTCATACAATGGTACATACAGAGGGCCGCCAACCCGCGAGGGGGAGCTAATCCCAGAAAGTGTATCGTAGTCCGGATTGTAGTCTGCAACTCGACTGCATGAAGTTGGAATCGCTAGTAATCGCGGATCAGCATGTCGCGGTGAATACGTTCCCGGGTCTTGTACACACCGCCCGTCACACCATGGGAGCGGGTTTTACCAGAAGTAGGTAG

>GW460-12-1-14-TSB6

ATGCAGTCGAGCGGCAGCACGGGTACTTGTACCTGGTGGCGAGCGGCGGACGGGTGAGTAATGCCTAGGAATCTGCCTGGTAGTGGGGGATAACGCTCGGAAACGGACGCTAATACCGCATACGTCCTACGGGAGAAAGCAGGGGACCTTCGGGCCTTGCGCTATCAGATGAGCCTAGGTCGGATTAGCTAGTTGGTGAGGTAATGGCTCACCAAGGCGACGATCCGTAACTGGTCTGAGAGGATGATCAGTCACACTGGAACTGAGACACGGTCCAGACTCCTACGGGAGGCAGCAGTGGGGAATATTGGACAATGGGCGAAAGCCTGATCCAGCCATGCCGCGTGTGTGAAGAAGGTCTTCGGATTGTAAAGCACTTTAAGTTGGGAGGAAGGGCAGTTACCTAATACGTATCTGTTTTGACGTTACCGACAGAATAAGCACCGGCTAACTCTGTGCCAGCAGCCGCGGTAATACAGAGGGTGCAAGCGTTAATCGGAATTACTGGGCGTAAAGCGCGCGTAGGTGGTTTGTTAAGTTGGATGTGAAATCCCCGGGCTCAACCTGGGAACTGCATTCAAAACTGACAAGCTAGAGTATGGTAGAGGGTGGTGGAATTTCCTGTGTAGCGGTGAAATGCGTAGATATAGGAAGGAACACCAGTGGCGAAGGCGACCACCTGGACTGATACTGACACTGAGGTGCGAAAGCGTGGGGAGCAAACAGGATTAGATACCCTGGTAGTCCACGCCGTAAACGATGTCAACTAGCCGTTGGGAGCCTTGAGCTCTTAGTGGCGCAGCTAACGCATTAAGTTGACCGCCTGGGGAGTACGGCCGCAAGGTTAAAACTCAAATGAATTGACGGGGGCCCGCACAAGCGGTGGAGCATGTGGTTTAATTCGAAGCAACGCGAAGAACCTTACCAGGCCTTGACATCCAATGAACTTTCCAGAGATGGATTGGTGCCTTCGGGAACATTGAGACAGGTGCTGCATGGCTGTCGTCAGCTCGTGTCGTGAGATGTTGGGTTAAGTCCCGTAACGAGCGCAACCCTTGTCCTTAGTTACCAGCACGTNATGGTGGGCACTCTAAGGAGACTGCCGGTGACAAACCGGAGGAAGGTGGGGATGACGTCAAGTCATCATGGCCCTTACGGCCTGGGCTACACACGTGCTACAATGGTCGGTACAGAGGGTTGCCAAGCCGCGAGGTGGAGCTAATCCCAGAAAACCGATCGTAGTCCGGATCGCAGTCTGCAACTCGACTGCGTGAAGTCGGAATCGCTAGTAATCGCGAATCAGAATGTCGCGGTGAATACGTTCCCGGGCCTTGTACACACCGCCCGTCACACCATGGGAGTGGGTTGCACCAGAAGTAGCTAGTCTAACC

>GW460-12-10-14-LB1

GCAGTCGAGCGGCAGCACGGGTACTTGTACCTGGTGGCGAGCGGCGGACGGGTGAGTAATGCCTAGGAATCTGCCTGGTAGTGGGGGATAACGCTCGGAAACGGACGCTAATACCGCATACGTCCTACGGGAGAAAGCAGGGGACCTTCGGGCCTTGCGCTATCAGATGAGCCTAGGTCGGATTAGCTAGTTGGTGAGGTAATGGCTCACCAAGGCGACGATCCGTAACTGGTCTGAGAGGATGATCAGTCACACTGGAACTGAGACACGGTCCAGACTCCTACGGGAGGCAGCAGTGGGGAATATTGGACAATGGGCGAAAGCCTGATCCAGCCATGCCGCGTGTGTGAAGAAGGTCTTCGGATTGTAAAGCACTTTAAGTTGGGAGGAAGGGCAGTTACCTAATACGTATCTGTTTTGACGTTACCGACAGAATAAGCACCGGCTAACTCTGTGCCAGCAGCCGCGGTAATACAGAGGGTGCAAGCGTTAATCGGAATTACTGGGCGTAAAGCGCGCGTAGGTGGTTTGTTAAGTTGGATGTGAAATCCCCGGGCTCAACCTGGGAACTGCATTCAAAACTGACAAGCTAGAGTATGGTAGAGGGTGGTGGAATTTCCTGTGTAGCGGTGAAATGCGTAGATATAGGAAGGAACACCAGTGGCGAAGGCGACCACCTGGACTGATACTGACACTGAGGTGCGAAAGCGTGGGGAGCAAACAGGATTAGATACCCTGGTAGTCCACGCCGTAAACGATGTCAACTAGCCGTTGGGAGCCTTGAGCTCTTAGTGGCGCAGCTAACGCATTAAGTTGACCGCCTGGGGAGTACGGCCGCAAGGTTAAAACTCAAATGAATTGACGGGGGCCCGCACAAGCGGTGGAGCATGTGGTTTAATTCGAAGCAACGCGAAGAACCTTACCAGGCCTTGACATCCAATGAACTTTCCAGAGATGGATTGGTGCCTTCGGGAACATTGAGACAGGTGCTGCATGGCTGTCGTCAGCTCGTGTCGTGAGATGTTGGGTTAAGTCCCGTAACGAGCGCAACCCTTGTCCTTAGTTACCAGCACGTNATGGTGGGCACTCTAAGGAGACTGCCGGTGACAAACCGGAGGAAGGTGGGGATGACGTCAAGTCATCATGGCCCTTACGGCCTGGGCTACACACGTGCTACAATGGTCGGTACAGAGGGTTGCCAAGCCGCGAGGTGGAGCTAATCCCAGAAAACCGATCGTAGTCCGGATCGCAGTCTGCAACTCGACTGCGTGAAGTCGGAATCGCTAGTAATCGCGAATCAGAATGTCGCGGTGAATACGTTCCCGGGCCTTGTACACACCGCCCGTCACACCATGGGAGTGGGTTGCACCAGAAGTAGCTAGTCT

>GW460-12-10-14-LB2

CGAACTCTTCGGAGTTAGTGGCGGACGGGTGAGTAACACGTGGGAACGTGCCTTTAGGTTCGGAATAACTCAGGGAAACTTGTGCTAATACCGAATGTGCCCTTCGGGGGAAAGATTTATCGCCTTTAGAGCGGCCCGCGTCTGATTAGCTAGTTGGTGAGGTAAAGGCTCACCAAGGCGACGATCAGTAGCTGGTCTGAGAGGATGATCAGCCACATTGGGACTGAGACACGGCCCAAACTCCTACGGGAGGCAGCAGTGGGGAATCTTGCGCAATGGGCGAAAGCCTGACGCAGCCATGCCGCGTGAATGATGAAGGTCTTAGGATTGTAAAATTCTTTCACCGGGGACGATAATGACGGTACCCGGAGAAGAAGCCCCGGCTAACTTCGTGCCAGCAGCCGCGGTAATACGAAGGGGGCTAGCGTTGCTCGGAATTACTGGGCGTAAAGGGAGCGTAGGCGGACATTTAAGTCAGGGGTGAAATCCCGGGGCTCAACCTCGGAATTGCCTTTGATACTGGGTGTCTTGAGTATGAGAGAGGTGTGTGGAACTCCGAGTGTAGAGGTGAAATTCGTAGATATTCGGAAGAACACCAGTGGCGAAGGCGACACACTGGCTCATTACTGACGCTGAGGCTCGAAAGCGTGGGGAGCAAACAGGATTAGATACCCTGGTAGTCCACGCCGTAAACGATGATTGCTAGTTGTCGGGATGCATGCATTTCGGTGACGCAGCTAACGCATTAAGCAATCCGCCTGGGGAGTACGGTCGCAAGATTAAAACTCAAAGGAATTGACGGGGGCCCGCACAAGCGGTGGAGCATGTGGTTTAATTCGAAGCAACGCGCAGAACCTTACCACCTTTTGACATGCCTGGACCGCCAGAGAGATCTGGCTTTCCCTTCGGGGACTAGGACACAGGTGCTGCATGGCTGTCGTCAGCTCGTGTCGTGAGATGTTGGGTTAAGTCCCGCAACGAGCGCAACCCTCGCCATTAGTTGCCATCATTTAGTTGGGAACTCTAATGGGACTGCCGGTGCTAAGCCGGAGGAAGGTGGGGATGACGTCAAGTCCTCATGGCCCTTACAGGGTGGGCTACACACGTGCTACAATGGCGACTACAGAGGGTTAATCCTTAAAAGTCGTCTCAGTTCGGATTGTCCTCTGCAACTCGAGGGCATGAAGTTGGAATCGCTAGTAATCGCGGATCAGCATGCCGCGGTGAATACGTTCCCGGGCCTTGTACACACCGCCCGTCACACCATGGGAGTTGGTTCTACCCGAAGGCGCTGCGCTGA

>GW460-12-10-14-LB3

GCAGTCGAGCGGGCCCTTCGGGGTCAGCGGCGGACGGGTGAGTAACGCGTGGGAACGTGCCTTCTGGTTCGGAATAACCCTGGGAAACTAGGGCTAATACCGGATACGCCCTTATGGGGAAAGGTTTACTGCCGGAAGATCGGCCCGCGTCTGATTAGCTAGTTGGTGGGGTAACGGCCTACCAAGGCGACGATCAGTAGCTGGTCTGAGAGGATGATCAGCCACACTGGGACTGAGACACGGCCCAGACTCCTACGGGAGGCAGCAGTGGGGAATATTGGACAATGGGCGCAAGCCTGATCCAGCCATGCCGCGTGAGTGATGAAGGCCTTAGGGTTGTAAAGCTCTTTTATCCGGGACGATAATGACGGTACCGGAGGAATAAGCCCCGGCTAACTTCGTGCCAGCAGCCGCGGTAATACGAAGGGGGCTAGCGTTGCTCGGAATCACTGGGCGTAAAGGGCGCGTAGGCGGCGTTTTAAGTCGGGGGTGAAAGCCTGTGGCTCAACCACAGAATGGCCTTCGATACTGGGACGCTTGAGTATGGTAGAGGTTGGTGGAACTGCGAGTGTAGAGGTGAAATTCGTAGATATTCGCAAGAACACCGGTGGCGAAGGCGGCCAACTGGACCATTACTGACGCTGAGGCGCGAAAGCGTGGGGAGCAAACAGGATTAGATACCCTGGTAGTCCACGCCGTAAACGATGAATGCCAGCTGTTGGGGTGCTTGCACCGCAGTAGCGCAGCTAACGCTTTGAGCATTCCGCCTGGGGAGTACGGTCGCAAGATTAAAACTCAAAGGAATTGACGGGGGCCCGCACAAGCGGTGGAGCATGTGGTTTAATTCGAAGCAACGCGCAGAACCTTACCATCCTTTGACATGGCGTGTTACCCAGAGAGATTTGGGGTCCACTTCGGTGGCGCGCACACAGGTGCTGCATGGCTGTCGTCAGCTCGTGTCGTGAGATGTTGGGTTAAGTCCCGCAACGAGCGCAACCCACGTCCTTAGTTGCCATCATTCAGTTGGGCACTCTAGGGAGACTGCCGGTGATAAGCCGCGAGGAAGGTGTGGATGACGTCAAGTCCTCATGGCCCTTACGGGATGGGCTACACACGTGCTACAATGGCGGTGACAGTGGGACGCGAAGGAGCGATCTGGAGCAAATCCCCAAAAGCCGTCTCAGTTCGGATTGCACTCTGCAACTCGAGTGCATGAAGGCGGAATCGCTAGTAATCGTGGATCAGCATGCCACGGTGAATACGTTCCCGGGCCTTGTACACACCGCCCGTCACACCATGGGAGTTGGTCTTACCCGACGGCGCTGCGCC

>GW460-12-10-14-LB4

TCTAGTGGCGCACGGGTGCGTAACGCGTGGGAATCTGCCCTTGGGTTCGGAATAACTCGCCGAAAGGCGTGCTAATACCGGATGATGTCGTAAGACCAAAGATTTATCGCCCAAGGATGAGCCCGCGTAAGATTAGGTAGTTGGTGAGGTAAAGGCTCACCAAGCCGACGATCTTTAGCTGGTCTGAGAGGATGATCAGCCACACTGGGACTGAGACACGGCCCAGACTCCTACGGGAGGCAGCAGTGGGGAATATTGGACAATGGGCGAAAGCCTGATCCAGCAATGCCGCGTGAGTGATGAAGGCCTTAGGGTTGTAAAGCTCTTTTACCCGGGATGATAATGACAGTACCGGGAGAATAAGCTCCGGCTAACTCCGTGCCAGCAGCCGCGGTAATACGGAGGGAGCTAGCGTTGTTCGGAATTACTGGGCGTAAAGCGCACGTAGGCGGCTTTGTAAGTCAGAGGTGAAAGCCTGGAGCTCAACTCCAGAACTGCCTTTGAGACTGCATCGCTTGAATCCAGGAGAGGTGAGTGGAATTCCGAGTGTAGAGGTGAAATTCGTAGATATTCGGAAGAACACCAGTGGCGAAGGCGGCTCACTGGACTGGTATTGACGCTGAGGTGCGAAAGCGTGGGGAGCAAACAGGATTAGATACCCTGGTAGTCCACGCCGTAAACGATGATAACTAGCTGTCCGGGCACTTGGTGCTTGGGTGGCGCAGCTAACGCATTAAGTTATCCGCCTGGGGAGTACGGTCGCAAGATTAAAACTCAAAGGAATTGACGGGGGCCTGCACAAGCGGTGGAGCATGTGGTTTAATTCGAAGCAACGCGCAGAACCTTACCAGCGTTTGACATGGTAGGACGACTTCCAGAGATGGATTTCTTCCCTTCGGGGACCTACACACAGGTGCTGCATGGCTGTCGTCAGCTCGTGTCGTGAGATGTTGGGTTAAGTCCCGCAACGAGCGCAACCCTCGCCTTTAGTTACCATCATTTAGTTGGGTACTCTAAAGGAACCGCCGGTGATAAGCCGGAGGAAGGTGGGGATGACGTCAAGTCCTCATGGCCCTTACGCGCTGGGCTACACACGTGCTACAATGGCAACTACAGTGGGCAGCAATCCCGCGAGGGTGAGCTAATCTCCAAAAGTTGTCTCAGTTCGGATTGTACTCTGCAACTCGAGTGCATGAAGGCGGAATCGCTAGTAATCGCGGATCAGCATGCCGCGGTGAATACGTTCCCAGGCCTTGTACACACCGCCCGTCACACC

>GW460-12-10-14-LB6

TTCGGGGTACTCGAGTGGCGAACGGGTGAGTAACACGTGGGTGATCTGCCCTGCACTTTGGGATAAGCCTGGGAAACTGGGTCTAATACCGAATATGACCACGCTCTTCATGGGGTGTGGTGGAAAGCTTTTGCGGTGTGGGATGGGCCCGCGGCCTATCAGCTTGTTGGTGGGGTAATGGCCTACCAAGGCGACGACGGGTAGCCGGCCTGAGAGGGTGACCGGCCACACTGGGACTGAGATACGGCCCAGACTCCTACGGGAGGCAGCAGTGGGGAATATTGCACAATGGGCGCAAGCCTGATGCAGCGACGCCGCGTGAGGGATGACGGCCTTCGGGTTGTAAACCTCTTTCACCAGGGACGAAGCGCAAGTGACGGTACCTGGAGAAGAAGCACCGGCCAACTACGTGCCAGCAGCCGCGGTAATACGTAGGGTGCGAGCGTTGTCCGGAATTACTGGGCGTAAAGAGCTCGTAGGTGGTTTGTCGCGTTGTTCGTGAAAACTCACAGCTTAACTGTGGGCGTGCGGGCGATACGGGCAGACTAGAGTACTGCAGGGGAGACTGGAATTCCTGGTGTAGCGGTGGAATGCGCAGATATCAGGAGGAACACCGGTGGCGAAGGCGGGTCTCTGGGCAGTAACTGACGCTGAGGAGCGAAAGCGTGGGGAGCGAACAGGATTAGATACCCTGGTAGTCCACGCCGTAAACGGTGGGTACTAGGTGTGGGTTCCTTCCTTGGGATCCGTGCCGTAGCTAACGCATTAAGTACCCCGCCTGGGGAGTACGGCCGCAAGGCTAAAACTCAAAGGAATTGACGGGGGCCCGCACAAGCGGCGGAGCATGTGGATTAATTCGATGCAACGCGAAGAACCTTACCTGGGTTTGACATGCACAGGACGCTGGTAGAGATATCAGTTCCCTTGTGGCCTGTGTGCAGGTGGTGCATGGCTGTCGTCAGCTCGTGTCGTGAGATGTTGGGTTAAGTCCCGCAACGAGCGCAACCCCTATCTTATGTTGCCAGCGCGTTATGGCGGGGACTCGTAAGAGACTGCCGGGGTCAACTCGGAGGAAGGTGGGGATGACGTCAAGTCATCATGCCCCTTATGTCCAGGGCTTCACACATGCTACAATGGCCGGTACAAAGGGCTGCGAATCCGCGAGGTGGAGCGAATCCCTTGAAAGCCGGTCTCAGTTCGGATCGGGGTCTGCAACTCGACCCCGTGAAGTTGGAGTCGCTAGTAATCGCAGATCAGCAACGCTGCGGTGAATACGTTCCCGGGCCTTGTACACACCGCCCGTCACGTCATGAAAGTCGGTAACACCCGAAG

>GW460-12-10-14-TSB1

TGCTTGCACCTCTTGAGAGCGGCGGACGGGTGAGTAATGCCTAGGAATCTGCCTGGTAGTGGGGGATAACGTTCGGAAACGGACGCTAATACCGCATACGTCCTACGGGAGAAAGCAGGGGACCTTCGGGCCTTGCGCTATCAGATGAGCCTAGGTCGGATTAGCTAGTTGGTGAGGTAATGGCTCACCAAGGCGACGATCCGTAACTGGTCTGAGAGGATGATCAGTCACACTGGAACTGAGACACGGTCCAGACTCCTACGGGAGGCAGCAGTGGGGAATATTGGACAATGGGCGAAAGCCTGATCCAGCCATGCCGCGTGTGTGAAGAAGGTCTTCGGATTGTAAAGCACTTTAAGTTGGGAGGAAGGGTACTTACCTAATACGTGAGTATTTTGACGTTACCGACAGAATAAGCACCGGCTAACTCTGTGCCAGCAGCCGCGGTAATACAGAGGGTGCAAGCGTTAATCGGAATTACTGGGCGTAAAGCGCGCGTAGGTGGTTCGTTAAGTTGAATGTGAAATCCCCGGGCTCAACCTGGGAACTGCATCCAAAACTGGCGAGCTAGAGTATGGTAGAGGGTGGTGGAATTTCCTGTGTAGCGGTGAAATGCGTAGATATAGGAAGGAACACCAGTGGCGAAGGCGACCACCTGGACTGATACTGACACTGAGGTGCGAAAGCGTGGGGAGCAAACAGGATTAGATACCCTGGTAGTCCACGCCGTAAACGATGTCAACTAGCCGTTGGGAGCCTTGAGCTCTTAGTGGCGCAGCTAACGCATTAAGTTGACCGCCTGGGGAGTACGGCCGCAAGGTTAAAACTCAAATGAATTGACGGGGGCCCGCACAAGCGGTGGAGCATGTGGTTTAATTCGAAGCAACGCGAAGAACCTTACCAGGCCTTGACATCCAATGAACTTTCCAGAGATGGATTGGTGCCTTCGGGAACATTGAGACAGGTGCTGCATGGCTGTCGTCAGCTCGTGTCGTGAGATGTTGGGTTAAGTCCCGTAACGAGCGCAACCCTTGTCCTTAGTTACCAGCACGTAATGGTGGGCACTCTAAGGAGACTGCCGGTGACAAACCGGAGGAAGGTGGGGATGACGTCAAGTCATCATGGCCCTTACGGCCTGGGCTACACACGTGCTACAATGGTCGGTACAGAGGGTTGCCAAGCCGCGAGGTGGAGCTAATCCCATAAAACCGATCGTAGTCCGGATCGCAGTCTGCAACTCGACTGCGTGAAGTCGGAATCGCTAGTAATCGCGAATCAGAATGTCGCGGTGAATACGTTCCCGGGCCTTGTACACACCGCCCGTCACACCATGGGAGTGGGTTGCACCAGAAGTAGCTAGTCTA

>GW460-12-10-14-TSB2

CTANANATGCAGTCGGACGAACTCTTCGGAGTTAGTGGCGGACGGGTGAGTAACACGTGGGAACGTGCCTTTAGGTTCGGAATAACTCAGGGAAACTTGTGCTAATACCGAATGTGCCCTTCGGGGGAAAGATTTATCGCCTTTAGAGCGGCCCGCGTCTGATTAGCTAGTTGGTGAGGTAAAGGCTCACCAAGGCGACGATCAGTAGCTGGTCTGAGAGGATGATCAGCCACATTGGGACTGAGACACGGCCCAAACTCCTACGGGAGGCAGCAGTGGGGAATCTTGCGCAATGGGCGAAAGCCTGACGCAGCCATGCCGCGTGAATGATGAAGGTCTTAGGATTGTAAAATTCTTTCACCGGGGACGATAATGACGGTACCCGGAGAAGAAGCCCCGGCTAACTTCGTGCCAGCAGCCGCGGTAATACGAAGGGGGCTAGCGTTGCTCGGAATTACTGGGCGTAAAGGGAGCGTAGGCGGACATTTAAGTCAGGGGTGAAATCCCGGGGCTCAACCTCGGAATTGCCTTTGATACTGGGTGTCTTGAGTATGAGAGAGGTGTGTGGAACTCCGAGTGTAGAGGTGAAATTCGTAGATATTCGGAAGAACACCAGTGGCGAAGGCGACACACTGGCTCATTACTGACGCTGAGGCTCGAAAGCGTGGGGAGCAAACAGGATTAGATACCCTGGTAGTCCACGCCGTAAACGATGATTGCTAGTTGTCGGGATGCATGCATTTCGGTGACGCAGCTAACGCATTAAGCAATCCGCCTGGGGAGTACGGTCGCAAGATTAAAACTCAAAGGAATTGACGGGGGCCCGCACAAGCGGTGGAGCATGTGGTTTAATTCGAAGCAACGCGCAGAACCTTACCACCTTTTGACATGCCTGGACCGCCAGAGAGATCTGGCTTTCCCTTCGGGGACTAGGACACAGGTGCTGCATGGCTGTCGTCAGCTCGTGTCGTGAGATGTTGGGTTAAGTCCCGCAACGAGCGCAACCCTCGCCATTAGTTGCCATCATTTAGTTGGGAACTCTAATGGGACTGCCGGTGCTAAGCCGGAGGAAGGTGGGGATGACGTCAAGTCCTCATGGCCCTTACAGGGTGGGCTACACACGTGCTACAATGGCGACTACAGAGGGTTAATCCTTAAAAGTCGTCTCAGTTCGGATTGTCCTCTGCAACTCGAGGGCATGAAGTTGGAATCGCTAGTAATCGCGGATCAGCATGCCGCGGTGAATACGTTCCCGGGCCTTGTACACACCGCCCGTCACACCATGGGAGTTGGTTCTACCCGAAGGCGCTGCGCT

>GW460-12-10-14-TSB4

GCTTGCTGGTGGATTAGTGGCGAACGGGTGAGTAACACGTGAGTAACCTGCCCTTGACTCTGGGATAAGCCTGGGAAACTGGGTCTAATACCGGATATGACTGATCATCGCATGGTGGTTGGTGGAAAGCTTTTGTGGTTTTGGATGGACTCGCGGCCTATCAGCTTGTTGGTGGGGTAATGGCCTACCAAGGCGACGACGGGTAGCCGGCCTGAGAGGGTGACCGGCCACACTGGGACTGAGACACGGCCCAGACTCCTACGGGAGGCAGCAGTGGGGAATATTGCACAATGGGCGAAAGCCTGATGCAGCGACGCCGCGTGAGGGATGACGGCCTTCGGGTTGTAAACCTCTTTCAGTAGGGAAGAAGCGTAAGTGACGGTACCTGCAGAAGAAGCGCCGGCTAACTACGTGCCAGCAGCCGCGGTAATACGTAGGGCGCAAGCGTTATCCGGAATTATTGGGCGTAAAGAGCTCGTAGGCGGTTTGTCGCGTCTGCCGTGAAAGTCCGGGGCTCAACTCCGGATCTGCGGTGGGTACGGGCAGACTAGAGTGATGTAGGGGAGACTGGAATTCCTGGTGTAGCGGTGAAATGCGCAGATATCAGGAGGAACACCGATGGCGAAGGCAGGTCTCTGGGCATTAACTGACGCTGAGGAGCGAAAGCATGGGGAGCGAACAGGATTAGATACCCTGGTAGTCCATGCCGTAAACGTTGGGCACTAGGTGTGGGGGACATTCCACGTTTTCCGCGCCGTAGCTAACGCATTAAGTGCCCCGCCTGGGGAGTACGGCCGCAAGGCTAAAACTCAAAGGAATTGACGGGGGCCCGCACAAGCGGCGGAGCATGCGGATTAATTCGATGCAACGCGAAGAACCTTACCAAGGCTTGACATGAACCAGACCGGGCTGGAAACAGTCCTTCCCCTTTGGGGTTGGTTTACAGGTGGTGCATGGTTGTCGTCAGCTCGTGTCGTGAGATGTTGGGTTAAGTCCCGCAACGAGCGCAACCCTCGTTCCATGTTGCCAGCGGGTAGTGCCGGGGACTCATGGGAGACTGCCGGGGTCAACTCGGAGGAAGGTGGGGACGACGTCAAATCATCATGCCCCTTATGTCTTGGGCTTCACGCATGCTACAATGGCCGGTACAAAGGGTTGCGATACTGTGAGGTGGAGCTAATCCCAAAAAGCCGGTCTCAGTTCGGATTGGGGTCTGCAACTCGACCCCATGAAGTCGGAGTCGCTAGTAATCGCAGATCAGCAACGCTGCGGTGAATACGTTCCCGGGCCTTGTACACACCGCCCGTCAAGTCACGAAAGTTGGTAACACCCGAAGCCG

>GW460-12-1-14-TSB3

CTACTTCTGGCAGAACCCGCTCCCATGGTGTGACGGGCGGTGTGTACAAGACCCGGGAACGTATTCACCGTGACATTCTGATCCACGATTACTAGCGATTCCGACTTCACGCAGTCGAGTTGCAGACTGCGATCCGGACTACGACTGGTTTTATGGGATTAGCTCCCCCTCGCGGGTTGGCAACCCTTTGTACCAGCCATTGTATGACGTGTGTAGCCCCACCTATAAGGGCCATGAGGACTTGACGTCATCCCCACCTTCCTCCGGTTTGTCACCGGCAGTCTCATTAGAGTGCCCAACTGAATGTAGCAACTAATGACAAGGGTTGCGCTCGTTGCGGGACTTAACCCAACATCTCACGACACGAGCTGACGACAGCCATGCAGCACCTGTGTTACGGTTCTCTTTCGAGCACTAAGCCATCTCTGGCGAATTCCGTACATGTCAAAGGTGGGTAAGGTTTTTCGCGTTGCATCGAATTAAACCACATCATCCACCGCTTGTGCGGGTCCCCGTCAATTCCTTTGAGTTTCAACCTTGCGGCCGTACTCCCCAGGCGGTCAACTTCACGCGTTAGCTTCGTTACTGAGTCAGTGAAGACCCAACAACCAGTTGACATCGTTTAGGGCGTGGACTACCAGGGTATCTAATCCTGTTTGCTCCCCACGCTTTCGTGCATGAGCGTCAGTACAGGTCCAGGGGATTGCCTTCGCCATCGGTGTTCCTCCGCATATCTACGCATTTCACTGCTACACGCGGAATTCCATCCCCCTCTACCGTACTCTAGCTATGCAGTCACAGATGCAGTTCCCAGGTTGAGCCCGGGGATTTCACAACTGTCTTACATAACCGCCTGCGCACGCTTTACGCCCAGTAATTCCGATTAACGCTTGCACCCTACGTATTACCGCGGCTGCTGGCACGTAGTTAGCCGGTGCTTATTCTTACGGTACCGTCATTA

>FW305-3-2-15-A-LB1

TGCAGTCGAACGGTAACAGGCCGCAAGGTGCTGACGAGTGGCGAACGGGTGAGTAATGCATCGGAACGTGCCCAGTCGTGGGGGATAACGCAGCGAAAGCTGCGCTAATACCGCATACGATCTATGGATGAAAGCGGGGGACCGTAAGGCCTCGCGCGATTGGAGCGGCCGATGTCAGATTAGGTAGTTGGTGGGGTAAAGGCTCACCAAGCCGACGATCTGTAGCTGGTCTGAGAGGACGACCAGCCACACTGGGACTGAGACACGGCCCAGACTCCTACGGGAGGCAGCAGTGGGGAATTTTGGACAATGGGCGCAAGCCTGATCCAGCAATGCCGCGTGCAGGAAGAAGGCCTTCGGGTTGTAAACTGCTTTTGTACGGAACGAAAAGGCTCTGGTTAATACCTGGGGCACATGACGGTACCGTAAGAATAAGCACCGGCTAACTACGTGCCAGCAGCCGCGGTAATACGTAGGGTGCAAGCGTTAATCGGAATTACTGGGCGTAAAGCGTGCGCAGGCGGTTTTGTAAGACAGGCGTGAAATCCCCGGGCTCAACCTGGGAATTGCGCTTGTGACTGCAAGGCTGGAGTGCGGCAGAGGGGGATGGAATTCCGCGTGTAGCAGTGAAATGCGTAGATATGCGGAGGAACACCGATGGCGAAGGCAATCCCCTGGGCCTGCACTGACGCTCATGCACGAAAGCGTGGGGAGCAAACAGGATTAGATACCCTGGTAGTCCACGCCCTAAACGATGTCAACTGGTTGTTGGGTCTCTTCTGACTCAGTAACGAAGCTAACGCGTGAAGTTGACCGCCTGGGGAGTACGGCCGCAAGGTTGAAACTCAAAGGAATTGACGGGGACCCGCACAAGCGGTGGATGATGTGGTTTAATTCGATGCAACGCGAAAAACCTTACCCACCTTTGACATGTACGGAATTTGCCAGAGATGGCTTAGTGCTCGAAAGAGAGCCGTAACACAGGTGCTGCATGGCTGTCGTCAGCTCGTGTCGTGAGATGTTGGGTTAAGTCCCGCAACGAGCGCAACCCTTGTCATTAGTTGCTACGAAAGGGCACTCTAATGAGACTGCCGGTGACAAACCGGAGGAAGGTGGGGATGACGTCAAGTCCTCATGGCCCTTATAGGTGGGGCTACACACGTCATACAATGGCTGGTACAAAGGGTTGCCAACCCGCGAGGGGGAGCTAATCCCATAAAGCCAGTCGTAGTCCGGATCGCAGTCTGCAACTCGACTGCGTGAAGTCGGAATCGCTAGTAATCGTGGATCAGCATGTCACGGTGAATACGTTCCCGGGTCTTGTACACACCGCCCGTCACACCATGGGAGCGGGTCTCGCCAGAAGTAGTTAGCC

>FW305-3-2-15-A-LB2

TGCAAGTCGAGCGGTAGAGAGAAGCTTGCTTCTCTTGAGAGCGGCGGACGGGTGAGTAATGCCTAGGAATCTGCCTGGTAGTGGGGGATAACGTTCGGAAACGAACGCTAATACCGCATACGTCCTACGGGAGAAAGCAGGGGACCTTCGGGCCTTGCGCTATCAGATGAGCCTAGGTCGGATTAGCTAGTTGGTGAGGTAATGGCTCACCAAGGCGACGATCCGTAACTGGTCTGAGAGGATGATCAGTCACACTGGAACTGAGACACGGTCCAGACTCCTACGGGAGGCAGCAGTGGGGAATATTGGACAATGGGCGAAAGCCTGATCCAGCCATGCCGCGTGTGTGAAGAAGGTCTTCGGATTGTAAAGCACTTTAAGTTGGGAGGAAGGGCAGTTACCTAATACGTGATTGTTTTGACGTTACCGACAGAATAAGCACCGGCTAACTCTGTGCCAGCAGCCGCGGTAATACAGAGGGTGCAAGCGTTAATCGGAATTACTGGGCGTAAAGCGCGCGTAGGTGGTTTGTTAAGTTGGATGTGAAATCCCCGGGCTCAACCTGGGAACTGCATTCAAAACTGACTGACTAGAGTATGGTAGAGGGTGGTGGAATTTCCTGTGTAGCGGTGAAATGCGTAGATATAGGAAGGAACACCAGTGGCGAAGGCGACCACCTGGACTAATACTGACACTGAGGTGCGAAAGCGTGGGGAGCAAACAGGATTAGATACCCTGGTAGTCCACGCCGTAAACGATGTCAACTAGCCGTTGGAAGCCTTGAGCTTTTAGTGGCGCAGCTAACGCATTAAGTTGACCGCCTGGGGAGTACGGCCGCAAGGTTAAAACTCAAATGAATTGACGGGGGCCCGCACAAGCGGTGGAGCATGTGGTTTAATTCGAAGCAACGCGAAGAACCTTACCAGGCCTTGACATCCAATGAACTTTCCAGAGATGGATTGGTGCCTTCGGGAACATTGAGACAGGTGCTGCATGGCTGTCGTCAGCTCGTGTCGTGAGATGTTGGGTTAAGTCCCGTAACGAGCGCAACCCTTGTCCTTAGTTACCAGCACGTCATGGTGGGCACTCTAAGGAGACTGCCGGTGACAAACCGGAGGAAGGTGGGGATGACGTCAAGTCATCATGGCCCTTACGGCCTGGGCTACACACGTGCTACAATGGTCGGTACAGAGGGTTGCCAAGCCGCGAGGTGGAGCTAATCCCATAAAACCGATCGTAGTCCGGATCGCAGTCTGCAACTCGACTGCGTGAAGTCGGAATCGCTAGTAATCGCGAATCAGAATGTCGCGGTGAATACGTTCCCGGGCCTTGTACACACCGCCCGTCACACCATGGGAGTGGGTTGCACCAGAAGTAGCTAGTCTAACCTTC

>FW305-3-2-15-A-R2A1

ATGCAGTCGAGCGGCAGCACGGGTACTTGTACCTGGTGGCGAGCGGCGGACGGGTGAGTAATGCCTAGGAATCTGCCTGGTAGTGGGGGATAACGCTCGGAAACGGACGCTAATACCGCATACGTCCTACGGGAGAAAGCAGGGGACCTTCGGGCCTTGCGCTATCAGATGAGCCTAGGTCGGATTAGCTAGTTGGTGAGGTAATGGCTCACCAAGGCGACGATCCGTAACTGGTCTGAGAGGATGATCAGTCACACTGGAACTGAGACACGGTCCAGACTCCTACGGGAGGCAGCAGTGGGGAATATTGGACAATGGGCGAAAGCCTGATCCAGCCATGCCGCGTGTGTGAAGAAGGTCTTCGGATTGTAAAGCACTTTAAGTTGGGAGGAAGGGCAGTTACCTAATACGTATCTGTTTTGACGTTACCGACAGAATAAGCACCGGCTAACTCTGTGCCAGCAGCCGCGGTAATACAGAGGGTGCAAGCGTTAATCGGAATTACTGGGCGTAAAGCGCGCGTAGGTGGTTTGTTAAGTTGGATGTGAAATCCCCGGGCTCAACCTGGGAACTGCATTCAAAACTGACAAGCTAGAGTATGGTAGAGGGTGGTGGAATTTCCTGTGTAGCGGTGAAATGCGTAGATATAGGAAGGAACACCAGTGGCGAAGGCGACCACCTGGACTGATACTGACACTGAGGTGCGAAAGCGTGGGGAGCAAACAGGATTAGATACCCTGGTAGTCCACGCCGTAAACGATGTCAACTAGCCGTTGGGAGCCTTGAGCTCTTAGTGGCGCAGCTAACGCATTAAGTTGACCGCCTGGGGAGTACGGCCGCAAGGTTAAAACTCAAATGAATTGACGGGGGCCCGCACAAGCGGTGGAGCATGTGGTTTAATTCGAAGCAACGCGAAGAACCTTACCAGGCCTTGACATCCAATGAACTTTCCAGAGATGGATTGGTGCCTTCGGGAACATTGAGACAGGTGCTGCATGGCTGTCGTCAGCTCGTGTCGTGAGATGTTGGGTTAAGTCCCGTAACGAGCGCAACCCTTGTCCTTAGTTACCAGCACGNNNTGGTGGGCACTCTAAGGAGACTGCCGGTGACAAACCGGAGGAAGGTGGGGATGACGTCAAGTCATCATGGCCCTTACGGCCTGGGCTACACACGTGCTACAATGGTCGGTACAGAGGGTTGCCAAGCCGCGAGGTGGAGCTAATCCCAGAAAACCGATCGTAGTCCGGATCGCAGTCTGCAACTCGACTGCGTGAAGTCGGAATCGCTAGTAATCGCGAATCAGAATGTCGCGGTGAATACGTTCCCGGGCCTTGTACACACCGCCCGTCACACCATGGGAGTGGGTTGCACCAGAAGTAGCTAGTCTAACC

>FW305-3-2-15-A-R2A2

AGTCGAGCGGTAGAGAGAAGCTTGCTTCTCTTGAGAGCGGCGGACGGGTGAGTAATGCCTAGGAATCTGCCTGGTAGTGGGGGATAACGTTCGGAAACGAACGCTAATACCGCATACGTCCTACGGGAGAAAGCAGGGGACCTTCGGGCCTTGCGCTATCAGATGAGCCTAGGTCGGATTAGCTAGTTGGTGAGGTAATGGCTCACCAAGGCGACGATCCGTAACTGGTCTGAGAGGATGATCAGTCACACTGGAACTGAGACACGGTCCAGACTCCTACGGGAGGCAGCAGTGGGGAATATTGGACAATGGGCGAAAGCCTGATCCAGCCATGCCGCGTGTGTGAAGAAGGTCTTCGGATTGTAAAGCACTTTAAGTTGGGAGGAAGGGCAGTTACCTAATACGTGATTGTTTTGACGTTACCGACAGAATAAGCACCGGCTAACTCTGTGCCAGCAGCCGCGGTAATACAGAGGGTGCAAGCGTTAATCGGAATTACTGGGCGTAAAGCGCGCGTAGGTGGTTTGTTAAGTTGGATGTGAAATCCCCGGGCTCAACCTGGGAACTGCATTCAAAACTGACTGACTAGAGTATGGTAGAGGGTGGTGGAATTTCCTGTGTAGCGGTGAAATGCGTAGATATAGGAAGGAACACCAGTGGCGAAGGCGACCACCTGGACTAATACTGACACTGAGGTGCGAAAGCGTGGGGAGCAAACAGGATTAGATACCCTGGTAGTCCACGCCGTAAACGATGTCAACTAGCCGTTGGAAGCCTTGAGCTTTTAGTGGCGCAGCTAACGCATTAAGTTGACCGCCTGGGGAGTACGGCCGCAAGGTTAAAACTCAAATGAATTGACGGGGGCCCGCACAAGCGGTGGAGCATGTGGTTTAATTCGAAGCAACGCGAAGAACCTTACCAGGCCTTGACATCCAATGAACTTTCCAGAGATGGATTGGTGCCTTCGGGAACATTGAGACAGGTGCTGCATGGCTGTCGTCAGCTCGTGTCGTGAGATGTTGGGTTAAGTCCCGTAACGAGCGCAACCCTTGTCCTTAGTTACCAGCACGTCATGGTGGGCACTCTAAGGAGACTGCCGGTGACAAACCGGAGGAAGGTGGGGATGACGTCAAGTCATCATGGCCCTTACGGCCTGGGCTACACACGTGCTACAATGGTCGGTACAGAGGGTTGCCAAGCCGCGAGGTGGAGCTAATCCCATAAAACCGATCGTAGTCCGGATCGCAGTCTGCAACTCGACTGCGTGAAGTCGGAATCGCTAGTAATCGCGAATCAGAATGTCGCGGTGAATACGTTCCCGGGCCTTGTACACACCGCCCGTCACACCATGGGAGTGGGTTGCACCAGAAGTAGCTAGTCTAACCT

>FW305-3-2-15-A-TSA1

TGCAGTCGAGCGGTAGAGAGAAGCTTGCTTCTCTTGAGAGCGGCGGACGGGTGAGTAATGCCTAGGAATCTGCCTGGTAGTGGGGGATAACGTTCGGAAACGAACGCTAATACCGCATACGTCCTACGGGAGAAAGCAGGGGACCTTCGGGCCTTGCGCTATCAGATGAGCCTAGGTCGGATTAGCTAGTTGGTGAGGTAATGGCTCACCAAGGCGACGATCCGTAACTGGTCTGAGAGGATGATCAGTCACACTGGAACTGAGACACGGTCCAGACTCCTACGGGAGGCAGCAGTGGGGAATATTGGACAATGGGCGAAAGCCTGATCCAGCCATGCCGCGTGTGTGAAGAAGGTCTTCGGATTGTAAAGCACTTTAAGTTGGGAGGAAGGGCAGTTACCTAATACGTGATTGTTTTGACGTTACCGACAGAATAAGCACCGGCTAACTCTGTGCCAGCAGCCGCGGTAATACAGAGGGTGCAAGCGTTAATCGGAATTACTGGGCGTAAAGCGCGCGTAGGTGGTTTGTTAAGTTGGATGTGAAATCCCCGGGCTCAACCTGGGAACTGCATTCAAAACTGACTGACTAGAGTATGGTAGAGGGTGGTGGAATTTCCTGTGTAGCGGTGAAATGCGTAGATATAGGAAGGAACACCAGTGGCGAAGGCGACCACCTGGACTAATACTGACACTGAGGTGCGAAAGCGTGGGGAGCAAACAGGATTAGATACCCTGGTAGTCCACGCCGTAAACGATGTCAACTAGCCGTTGGAAGCCTTGAGCTTTTAGTGGCGCAGCTAACGCATTAAGTTGACCGCCTGGGGAGTACGGCCGCAAGGTTAAAACTCAAATGAATTGACGGGGGCCCGCACAAGCGGTGGAGCATGTGGTTTAATTCGAAGCAACGCGAAGAACCTTACCAGGCCTTGACATCCAATGAACTTTCCAGAGATGGATTGGTGCCTTCGGGAACATTGAGACAGGTGCTGCATGGCTGTCGTCAGCTCGTGTCGTGAGATGTTGGGTTAAGTCCCGTAACGAGCGCAACCCTTGTCCTTAGTTACCAGCACGTCATGGTGGGCACTCTAAGGAGACTGCCGGTGACAAACCGGAGGAAGGTGGGGATGACGTCAAGTCATCATGGCCCTTACGGCCTGGGCTACACACGTGCTACAATGGTCGGTACAGAGGGTTGCCAAGCCGCGAGGTGGAGCTAATCCCATAAAACCGATCGTAGTCCGGATCGCAGTCTGCAACTCGACTGCGTGAAGTCGGAATCGCTAGTAATCGCGAATCAGAATGTCGCGGTGAATACGTTCCCGGGCCTTGTACACACCGCCCGTCACACCATGGGAGTGGGTTGCACCAGAAGTAGCTAGTCTAACCTTC

>FW305-3-2-15-A-TSA2

TGCAGTCGAACGGTAACAGGTCTTCGGATGCTGACGAGTGGCGAACGGGTGAGTAATACATCGGAACGTGCCCGATCGTGGGGGATAACGGAGCGAAAGCTTTGCTAATACCGCATACGATCTACGGATGAAAGCAGGGGACCGCAAGGCCTTGCGCGGACGGAGCGGCCGATGGCAGATTAGGTAGTTGGTGGGATAAAAGCTTACCAAGCCGACGATCTGTAGCTGGTCTGAGAGGACGACCAGCCACACTGGGACTGAGACACGGCCCAGACTCCTACGGGAGGCAGCAGTGGGGAATTTTGGACAATGGGCGAAAGCCTGATCCAGCCATGCCGCGTGCAGGATGAAGGCCTTCGGGTTGTAAACTGCTTTTGTACGGAACGAAAAGACTCTTTCTAATAAAGAGGGTCCATGACGGTACCGTAAGAATAAGCACCGGCTAACTACGTGCCAGCAGCCGCGGTAATACGTAGGGTGCAAGCGTTAATCGGAATTACTGGGCGTAAAGCGTGCGCAGGCGGTTATGTAAGACAGATGTGAAATCCCCGGGCTCAACCTGGGAACTGCATTTGTGACTGCATAGCTAGAGTACGGCAGAGGGGGATGGAATTCCGCGTGTAGCAGTGAAATGCGTAGATATGCGGAGGAACACCGATGGCGAAGGCAATCCCCTGGGCCTGTACTGACGCTCATGCACGAAAGCGTGGGGAGCAAACAGGATTAGATACCCTGGTAGTCCACGCCCTAAACGATGTCAACTGGTTGTTGGGTCTTCACTGACTCAGTAACGAAGCTAACGCGTGAAGTTGACCGCCTGGGGAGTACGGCCGCAAGGTTGAAACTCAAAGGAATTGACGGGGACCCGCACAAGCGGTGGATGATGTGGTTTAATTCGATGCAACGCGAAAAACCTTACCCACCTTTGACATGTACGGAATCCTTTAGAGATAGAGGAGTGCTCGAAAGAGAACCGTAACACAGGTGCTGCATGGCTGTCGTCAGCTCGTGTCGTGAGATGTTGGGTTAAGTCCCGCAACGAGCGCAACCCTTGCCATTAGTTGCTACGAAAGGGCACTCTAATGGGACTGCCGGTGACAAACCGGAGGAAGGTGGGGATGACGTCAAGTCCTCATGGCCCTTATAGGTGGGGCTACACACGTCATACAATGGCTGGTACAGAGGGTTGCCAACCCGCGAGGGGGAGCCAATCCCATAAAGCCAGTCGTAGTCCGGATCGCAGTCTGCAACTCGACTGCGTGAAGTCGGAATCGCTAGTAATCGCGGATCAGAATGTCGCGGTGAATACGTTCCCGGGTCTTGTACACACCGCCCGTCACACCATGGGAGCGGGTTCTGCCAGAAGTAGTTAGCCTAACCGCAAGGAGGGCG

>FW305-3-2-15-A-TSA3

TGCAGTCGAGCGGTAGAGAGAAGCTTGCTTCTCTTGAGAGCGGCGGACGGGTGAGTAATGCCTAGGAATCTGCCTGGTAGTGGGGGATAACGTTCGGAAACGAACGCTAATACCGCATACGTCCTACGGGAGAAAGCAGGGGACCTTCGGGCCTTGCGCTATCAGATGAGCCTAGGTCGGATTAGCTAGTTGGTGAGGTAATGGCTCACCAAGGCGACGATCCGTAACTGGTCTGAGAGGATGATCAGTCACACTGGAACTGAGACACGGTCCAGACTCCTACGGGAGGCAGCAGTGGGGAATATTGGACAATGGGCGAAAGCCTGATCCAGCCATGCCGCGTGTGTGAAGAAGGTCTTCGGATTGTAAAGCACTTTAAGTTGGGAGGAAGGGCAGTTACCTAATACGTGATTGTTTTGACGTTACCGACAGAATAAGCACCGGCTAACTCTGTGCCAGCAGCCGCGGTAATACAGAGGGTGCAAGCGTTAATCGGAATTACTGGGCGTAAAGCGCGCGTAGGTGGTTTGTTAAGTTGGATGTGAAATCCCCGGGCTCAACCTGGGAACTGCATTCAAAACTGACTGACTAGAGTATGGTAGAGGGTGGTGGAATTTCCTGTGTAGCGGTGAAATGCGTAGATATAGGAAGGAACACCAGTGGCGAAGGCGACCACCTGGACTAATACTGACACTGAGGTGCGAAAGCGTGGGGAGCAAACAGGATTAGATACCCTGGTAGTCCACGCCGTAAACGATGTCAACTAGCCGTTGGAAGCCTTGAGCTTTTAGTGGCGCAGCTAACGCATTAAGTTGACCGCCTGGGGAGTACGGCCGCAAGGTTAAAACTCAAATGAATTGACGGGGGCCCGCACAAGCGGTGGAGCATGTGGTTTAATTCGAAGCAACGCGAAGAACCTTACCAGGCCTTGACATCCAATGAACTTTCCAGAGATGGATTGGTGCCTTCGGGAACATTGAGACAGGTGCTGCATGGCTGTCGTCAGCTCGTGTCGTGAGATGTTGGGTTAAGTCCCGTAACGAGCGCAACCCTTGTCCTTAGTTACCAGCACGTCATGGTGGGCACTCTAAGGAGACTGCCGGTGACAAACCGGAGGAAGGTGGGGATGACGTCAAGTCATCATGGCCCTTACGGCCTGGGCTACACACGTGCTACAATGGTCGGTACAGAGGGTTGCCAAGCCGCGAGGTGGAGCTAATCCCATAAAACCGATCGTAGTCCGGATCGCAGTCTGCAACTCGACTGCGTGAAGTCGGAATCGCTAGTAATCGCGAATCAGAATGTCGCGGTGAATACGTTCCCGGGCCTTGTACACACCGCCCGTCACACCATGGGAGTGGGTTGCACCAGAAGTAGCTAGTCTA

>FW305-3-2-15-B-LB1

TGCAGTCGAACGGCAGCGCGGGAGCAATCCTGGCGGCGAGTGGCGAACGGGTGAGTAATACATCGGAACGTGCCCAATCGTGGGGGATAACGCAGCGAAAGCTGTGCTAATACCGCATACGATCTACGGATGAAAGCAGGGGATCGCAAGACCTTGCGCGAATGGAGCGGCCGATGGCAGATTAGGTAGTTGGTGAGGTAAAGGCTCACCAAGCCTTCGATCTGTAGCTGGTCTGAGAGGACGACCAGCCACACTGGGACTGAGACMCGGCCCAGACTCCTACGGGAGGCAGCAGTGGGGAATTTTGGACAATGGGCGAAAGCCTGATCCAGCCATGCCGCGTGCAGGATGAAGGCCTTCGGGTTGTAAACTGCTTTTGTACGGAACGAAACGGTCTTTTCTAATACAGAAGGCTAATGACGGTACCGTAAGAATAAGCACCGGCTAACTACGTGCCAGCAGCCGCGGTAATACGTAGGGTGCAAGCGTTAATCGGAATTACTGGGCGTAAAGCGTGCGCAGGCGGTTATGTAAGACAGTTGTGAAATCCCCGGGCTCAACCTGGGAACTGCATCTGTGACTGCATAGCTAGAGTACGGTAGAGGGGGATGGAATTCCGCGTGTAGCAGTGAAATGCGTAGATATGCGGAGGAACACCGATGGCGAAGGCAATCCCCTGGACCTGTACTGACGCTCATGCACGAAAGCGTGGGGAGCAAACAGGATTAGATACCCTGGTAGTCCACGCCCTAAACGATGTCAACTGGTTGTTGGGTCTTCACTGACTCAGTAACGAAGCTAACGCGTGAAGTTGACCGCCTGGGGAGTACGGCCGCAAGGTTGAAACTCAAAGGAATTGACGGGGACCCGCACAAGCGGTGGATGATGTGGTTTAATTCGATGCAACGCGAAAAACCTTACCCACCTTTGACATGTACGGAATTCGCCAGAGATGGCTTAGTGCTCGAAAGAGAACCGTAACACAGGTGCTGCATGGCTGTCGTCAGCTCGTGTCGTGAGATGTTGGGTTAAGTCCCGCAACGAGCGCAACCCTTGTCATTAGTTGCTACATTCAGTTGGGCACTCTAATGAGACTGCCGGTGACAAACCGGAGGAAGGTGGGGATGACGTCAAGTCCTCATGGCCCTTATAGGTGGGGCTACACACGTCATACAATGGCTGGTACAAAGGGTTGCCAACCCGCGAGGGGGAGCTAATCCCATAAAACCAGTCGTAGTCCGGATCGCAGTCTGCAACTCGACTGCGTGAAGTCGGAATCGCTAGTAATCGTGGATCAGAATGTCACGGTGAATACGTTCCCGGGTCTTGTACACACCGCCCGTCACACCATGGGAGCGGGTTCTGCCAGAAGTAGTTAGCTTAACCGCAAGGAGGGCGAT

>FW305-3-2-15-B-LB2

GCAGTCGAACGGCAGCGCGGGAGCAATCCTGGCGGCGAGTGGCGAACGGGTGAGTAATACATCGGAACGTGCCCAATCGTGGGGGATAACGCAGCGAAAGCTGTGCTAATACCGCATACGATCTACGGATGAAAGCAGGGGATCGCAAGACCTTGCGCGAATGGAGCGGCCGATGGCAGATTAGGTAGTTGGTGAGGTAAAGGCTCACCAAGCCTTCGATCTGTAGCTGGTCTGAGAGGACGACCAGCCACACTGGGACTGAGACACGGCCCAGACTCCTACGGGAGGCAGCAGTGGGGAATTTTGGACAATGGGCGAAAGCCTGATCCAGCCATGCCGCGTGCAGGATGAAGGCCTTCGGGTTGTAAACTGCTTTTGTACGGAACGAAACGGTCTTTTCTAATACAGAAGGCTAATGACGGTACCGTAAGAATAAGCACCGGCTAACTACGTGCCAGCAGCCGCGGTAATACGTAGGGTGCAAGCGTTAATCGGAATTACTGGGCGTAAAGCGTGCGCAGGCGGTTATGTAAGACAGTTGTGAAATCCCCGGGCTCAACCTGGGAACTGCATCTGTGACTGCATAGCTAGAGTACGGTAGAGGGGGATGGAATTCCGCGTGTAGCAGTGAAATGCGTAGATATGCGGAGGAACACCGATGGCGAAGGCAATCCCCTGGACCTGTACTGACGCTCATGCACGAAAGCGTGGGGAGCAAACAGGATTAGATACCCTGGTAGTCCACGCCCTAAACGATGTCAACTGGTTGTTGGGTCTTCACTGACTCAGTAACGAAGCTAACGCGTGAAGTTGACCGCCTGGGGAGTACGGCCGCAAGGTTGAAACTCAAAGGAATTGACGGGGACCCGCACAAGCGGTGGATGATGTGGTTTAATTCGATGCAACGCGAAAAACCTTACCCACCTTTGACATGTACGGAATTCGCCAGAGATGGCTTAGTGCTCGAAAGAGAACCGTAACACAGGTGCTGCATGGCTGTCGTCAGCTCGTGTCGTGAGATGTTGGGTTAAGTCCCGCAACGAGCGCAACCCTTGTCATTAGTTGCTACATTCAGTTGGGCACTCTAATGAGACTGCCGGTGACAAACCGGAGGAAGGTGGGGATGACGTCAAGTCCTCATGGCCCTTATAGGTGGGGCTACACACGTCATACAATGGCTGGTACAAAGGGTTGCCAACCCGCGAGGGGGAGCTAATCCCATAAAACCAGTCGTAGTCCGGATCGCAGTCTGCAACTCGACTGCGTGAAGTCGGAATCGCTAGTAATCGTGGATCAGAATGTCACGGTGAATACGTTCCCGGGTCTTGTACACACCGCCCGTCACACCATGGGAGCGGGTTCTGCCAGAAGTAGTTAGCTTAACCGCAAGGAGG

>FW305-3-2-15-B-TSA1

GCAGTCGANCGGCAGCGCGGGAGCAATCCTGGCGGCGAGTGGCGAACGGGTGAGTAATACATCGGAACGTGCCCAATCGTGGGGGATAACGCAGCGAAAGCTGTGCTAATACCGCATACGATCTACGGATGAAAGCAGGGGATCGCAAGACCTTGCGCGAATGGAGCGGCCGATGGCAGATTAGGTAGTTGGTGAGGTAAAGGCTCACCAAGCCTTCGATCTGTAGCTGGTCTGAGAGGACGACCAGCCACACTGGGACTGAGACACGGCCCAGACTCCTACGGGAGGCAGCAGTGGGGAATTTTGGACAATGGGCGAAAGCCTGATCCAGCCATGCCGCGTGCAGGATGAAGGCCTTCGGGTTGTAAACTGCTTTTGTACGGAACGAAACGGTCTTTTCTAATACAGAAGGCTAATGACGGTACCGTAAGAATAAGCACCGGCTAACTACGTGCCAGCAGCCGCGGTAATACGTAGGGTGCAAGCGTTAATCGGAATTACTGGGCGTAAAGCGTGCGCAGGCGGTTATGTAAGACAGTTGTGAAATCCCCGGGCTCAACCTGGGAACTGCATCTGTGACTGCATAGCTAGAGTACGGTAGAGGGGGATGGAATTCCGCGTGTAGCAGTGAAATGCGTAGATATGCGGAGGAACACCGATGGCGAAGGCAATCCCCTGGACCTGTACTGACGCTCATGCACGAAAGCGTGGGGAGCAAACAGGATTAGATACCCTGGTAGTCCACGCCCTAAACGATGTCAACTGGTTGTTGGGTCTTCACTGACTCAGTAACGAAGCTAACGCGTGAAGTTGACCGCCTGGGGAGTACGGCCGCAAGGTTGAAACTCAAAGGAATTGACGGGGACCCGCACAAGCGGTGGATGATGTGGTTTAATTCGATGCAACGCGAAAAACCTTACCCACCTTTGACATGTACGGAATTCGCCAGAGATGGCTTAGTGCTCGAAAGAGAACCGTAACACAGGTGCTGCATGGCTGTCGTCAGCTCGTGTCGTGAGATGTTGGGTTAAGTCCCGCAACGAGCGCAACCCTTGTCATTAGTTGCTACATTCAGTTGGGCACTCTAATGAGACTGCCGGTGACAAACCGGAGGAAGGTGGGGATGACGTCAAGTCCTCATGGCCCTTATAGGTGGGGCTACACACGTCATACAATGGCTGGTACAAAGGGTTGCCAACCCGCGAGGGGGAGCTAATCCCATAAAACCAGTCGTAGTCCGGATCGCAGTCTGCAACTCGACTGCGTGAAGTCGGAATCGCTAGTAATCGTGGATCAGAATGTCACGGTGAATACGTTCCCGGGTCTTGTACACACCGCCCGTCACACCATGGGAGCGGGTTCTGCCAGAAGTAGTTAGCTTA

>FW305-3-2-15-B-TSA2

TGCAGTCGAACGGCAGCGCGGGAGCAATCCTGGCGGCGAGTGGCGAACGGGTGAGTAATACATCGGAACGTGCCCAATCGTGGGGGATAACGCAGCGAAAGCTGTGCTAATACCGCATACGATCTACGGATGAAAGCAGGGGATCGCAAGACCTTGCGCGAATGGAGCGGCCGATGGCAGATTAGGTAGTTGGTGAGGTAAAGGCTCACCAAGCCTTCGATCTGTAGCTGGTCTGAGAGGACGACCAGCCACACTGGGACTGAGACACGGCCCAGACTCCTACGGGAGGCAGCAGTGGGGAATTTTGGACAATGGGCGAAAGCCTGATCCAGCCATGCCGCGTGCAGGATGAAGGCCTTCGGGTTGTAAACTGCTTTTGTACGGAACGAAACGGTCTTTTCTAATACAGAAGGCTAATGACGGTACCGTAAGAATAAGCACCGGCTAACTACGTGCCAGCAGCCGCGGTAATACGTAGGGTGCAAGCGTTAATCGGAATTACTGGGCGTAAAGCGTGCGCAGGCGGTTATGTAAGACAGTTGTGAAATCCCCGGGCTCAACCTGGGAACTGCATCTGTGACTGCATAGCTAGAGTACGGTAGAGGGGGATGGAATTCCGCGTGTAGCAGTGAAATGCGTAGATATGCGGAGGAACACCGATGGCGAAGGCAATCCCCTGGACCTGTACTGACGCTCATGCACGAAAGCGTGGGGAGCAAACAGGATTAGATACCCTGGTAGTCCACGCCCTAAACGATGTCAACTGGTTGTTGGGTCTTCACTGACTCAGTAACGAAGCTAACGCGTGAAGTTGACCGCCTGGGGAGTACGGCCGCAAGGTTGAAACTCAAAGGAATTGACGGGGACCCGCACAAGCGGTGGATGATGTGGTTTAATTCGATGCAACGCGAAAAACCTTACCCACCTTTGACATGTACGGAATTCGCCAGAGATGGCTTAGTGCTCGAAAGAGAACCGTAACACAGGTGCTGCATGGCTGTCGTCAGCTCGTGTCGTGAGATGTTGGGTTAAGTCCCGCAACGAGCGCAACCCTTGTCATTAGTTGCTACATTCAGTTGGGCACTCTAATGAGACTGCCGGTGACAAACCGGAGGAAGGTGGGGATGACGTCAAGTCCTCATGGCCCTTATAGGTGGGGCTACACACGTCATACAATGGCTGGTACAAAGGGTTGCCAACCCGCGAGGGGGAGCTAATCCCATAAAACCAGTCGTAGTCCGGATCGCAGTCTGCAACTCGACTGCGTGAAGTCGGAATCGCTAGTAATCGTGGATCAGAATGTCACGGTGAATACGTTCCCGGGTCTTGTACACACCGCCCGTCACACCATGGGAGCGGGTTCTGCCAGAAGTAGTTAGCTTA

>FW305-3-2-15-B-TSA3

TGCAGTCGAACGGCAGCACGGGCTTCGGCCTGGTGGCGAGTGGCGAACGGGTGAGTAATACATCGGAACGTGCCCTGTAGTGGGGGATAACTAGTCGAAAGATTAGCTAATACCGCATACGACCTGAGGGTGAAAGCGGGGGACCGCAAGGCCTCGCGCTACAGGAGCGGCCGATGTCTGATTAGCTAGTTGGTGGGGTAAAGGCCTACCAAGGCGACGATCAGTAGNTGGTCTGAGAGGACGATCAGCCACACTGGGACTGAGACACGGCCCNNACTCCTACGGGAGGCAGCAGTGGGGAATTTTGGACAATGGGGGCAACCCTGATCCAGCAATGCCGCGTGTGTGAAGAAGGCCTTCGGGTTGTAAAGCACTTTTGTCCGGAAAGAAATGGCTCTGGTTAATACCCGGGGTCGATGACGGTACCGGAAGAATAAGCACCGGCTAACTACGTGCCAGCAGCCGCGGTAATACGTAGGGTGCGAGCGTTAATCGGAATTACTGGGCGTAAAGCGTGCGCAGGCGGTTTTGTAAGACAGGCGTGAAATCCCCGAGCTCAACTTGGGAATGGCGCTTGTGACTGCAAGGCTAGAGTATGTCAGAGGGGGGTAGAATTCCACGTGTAGCAGTGAAATGCGTAGAGATGTGGAGGAATACCGATGGCGAAGGCAGCCCCCTGGGACGTCACTGACGCTCATGCACGAAAGCGTGGGGAGCAAACAGGATTAGATACCCTGGTAGTCCACGCCCTAAACGATGTCAACTAGTTGTTGGGGATTCATTTCTTCAGTAACGTAGCTAACGCGTGAAGTTGACCGCCTGGGGAGTACGGTCGCAAGATTAAAACTCAAAGGAATTGACGGGGACCCGCACAAGCGGTGGATGATGTGGATTAATTCGATGCAACGCGAAAAACCTTACCTACCCTTGACATGCCACTAACGAAGCAGAGATGCATCAGGTGCCCGAAAGGGAAAGTGGACACAGGTGCTGCATGGCTGTCGTCAGCTCGTGTCGTGAGATGTTGGGTTAAGTCCCGCAACGAGCGCAACCCTTGTCTCTAGTTGCTACGAAAGGGCACTCTAGAGAGACTGCCGGTGACAAACCGGAGGAAGGTGGGGATGACGTCAAGTCCTCATGGCCCTTATGGGTAGGGCTTCACACGTCATACAATGGTGCGTACAGAGGGTTGCCAACCCGCGAGGGGGAGCTAATCCCAGAAAACGCATCGTAGTCCGGATCGTAGTCTGCAACTCGACTACGTGAAGCTGGAATCGCTAGTAATCGCGGATCAGCATGCCGCGGTGAATACGTTCCCGGGTCTTGTACACACCGCCCGTCACACCATGGGAGTGGGTTTTGCCAGAAGTAGTTAGCCT

>FW305-3-2-15-C-LB1

TGCAGTCGAGCGGTAGAGAGAAGCTTGCTTCTCTTGAGAGCGGCGGACGGGTGAGTAATGCCTAGGAATCTGCCTGGTAGTGGGGGATAACGTTCGGAAACGAACGCTAATACCGCATACGTCCTACGGGAGAAAGCAGGGGACCTTCGGGCCTTGCGCTATCAGATGAGCCTAGGTCGGATTAGCTAGTTGGTGAGGTAATGGCTCACCAAGGCGACGATCCGTAACTGGTCTGAGAGGATGATCAGTCACACTGGAACTGAGACACGGTCCAGACTCCTACGGGAGGCAGCAGTGGGGAATATTGGACAATGGGCGAAAGCCTGATCCAGCCATGCCGCGTGTGTGAAGAAGGTCTTCGGATTGTAAAGCACTTTAAGTTGGGAGGAAGGGCAGTTACCTAATACGTGATTGTTTTGACGTTACCGACAGAATAAGCACCGGCTAACTCTGTGCCAGCAGCCGCGGTAATACAGAGGGTGCAAGCGTTAATCGGAATTACTGGGCGTAAAGCGCGCGTAGGTGGTTTGTTAAGTTGGATGTGAAATCCCCGGGCTCAACCTGGGAACTGCATTCAAAACTGACTGACTAGAGTATGGTAGAGGGTGGTGGAATTTCCTGTGTAGCGGTGAAATGCGTAGATATAGGAAGGAACACCAGTGGCGAAGGCGACCACCTGGACTAATACTGACACTGAGGTGCGAAAGCGTGGGGAGCAAACAGGATTAGATACCCTGGTAGTCCACGCCGTAAACGATGTCAACTAGCCGTTGGAAGCCTTGAGCTTTTAGTGGCGCAGCTAACGCATTAAGTTGACCGCCTGGGGAGTACGGCCGCAAGGTTAAAACTCAAATGAATTGACGGGGGCCCGCACAAGCGGTGGAGCATGTGGTTTAATTCGAAGCAACGCGAAGAACCTTACCAGGSCTTGACATCCAATGAACTTTCCAGAGATGGATTGGTGCCTTCGGGAACATTGAGACAGGTGCTGCATGGCTGTCGTCAGCTCGTGTCGTGAGATGTTGGGTTAAGTCCCGTAACGAGCGCAACCCTTGTCCTTAGTTACCAGCACGTCATGGTGGGCACTCTAAGGAGACTGCCGGTGACAAACCGGAGGAAGGTGGGGATGACGTCAAGTCATCATGGCCCTTACGGCCTGGGCTACACACGTGCTACAATGGTCGGTACAGAGGGTTGCCAAGCCGCGAGGTGGAGCTAATCCCATAAAACCGATCGTAGTCCGGATCGCAGTCTGCAACTCGACTGCGTGAAGTCGGAATCGCTAGTAATCGCGAATCAGAATGTCGCGGTGAATACGTTCCCGGGCCTTGTACACACCGCCCGTCACACCATGGGAGTGGGTTGCACCAGAAGTAGCTAGTCTAACCTTCGGGNGGACG

>FW305-3-2-15-C-LB2

TGCAGTCGAACGGCAGCACGGGCTTCGGCCTGGTGGCGAGTGGCGAACGGGTGAGTAATACATCGGAACGTGCCCTGTAGTGGGGGATAACTAGTCGAAAGATTAGCTAATACCGCATACGACCTGAGGGTGAAAGCGGGGGACCGCAAGGCCTCGCGCTACAGGAGCGGCCGATGTCTGATTAGNTAGTTGGTGGGGTAAAGGCCTACCAAGGCGANNNTCAGTAGCTGGNCTGAGAGGACGATCAGCCACACTGGGACTGAGACMCGGCCCASACTCCTACGGGAGGCAGCAGTGGGGAATTTTGGACAATGGGGGCAACCCTGATCCAGCAATGCCGCGTGTGTGAAGAAGGCCTTCGGGTTGTAAAGCACTTTTGTCCGGAAAGAAATGGCTCTGGTTAATACCCGGGGTCGATGACGGTACCGGAAGAATAAGCACCGGCTAACTACGTGCCAGCAGCCGCGGTAATACGTAGGGTGCGAGCGTTAATCGGAATTACTGGGCGTAAAGCGTGCGCAGGCGGTTTTGTAAGACAGGCGTGAAATCCCCGAGCTCAACTTGGGAATGGCGCTTGTGACTGCAAGGCTAGAGTATGTCAGAGGGGGGTAGAATTCCACGTGTAGCAGTGAAATGCGTAGAGATGTGGAGGAATACCGATGGCGAAGGCAGCCCCCTGGGACGTCACTGACGCTCATGCACGAAAGCGTGGGGAGCAAACAGGATTAGATACCCTGGTAGTCCACGCCCTAAACGATGTCAACTAGTTGTTGGGGATTCATTTCTTCAGTAACGTAGCTAACGCGTGAAGTTGACCGCCTGGGGAGTACGGTCGCAAGATTAAAACTCAAAGGAATTGACGGGGACCCGCACAAGCGGTGGATGATGTGGATTAATTCGATGCAACGCGAAAAACCTTACCTACCCTTGACATGCCACTAACGAAGCAGAGATGCATCAGGTGCCCGAAAGGGAAAGTGGACACAGGTGCTGCATGGCTGTCGTCAGCTCGTGTCGTGAGATGTTGGGTTAAGTCCCGCAACGAGCGCAACCCTTGTCTCTAGTTGCTACGAAAGGGCACTCTAGAGAGACTGCCGGTGACAAACCGGAGGAAGGTGGGGATGACGTCAAGTCCTCATGGCCCTTATGGGTAGGGCTTCACACGTCATACAATGGTGCGTACAGAGGGTTGCCAACCCGCGAGGGGGAGCTAATCCCAGAAAACGCATCGTAGTCCGGATCGTAGTCTGCAACTCGACTACGTGAAGCTGGAATCGCTAGTAATCGCGGATCAGCATGCCGCGGTGAATACGTTCCCGGGTCTTGTACACACCGCCCGTCACACCATGGGAGTGGGTTTTGCCAGAAGTAGTTAGCCTAACCGCAAGGAGGGCGAT

>FW305-3-2-15-C-LB3

GCTACACATGCAGTCGAGCGGTAGAGAGAAGCTTGCTTCTCTTGAGAGCGGCGGACGGGTGAGTAATGCCTAGGAATCTGCCTGGTAGTGGGGGATAACGTTCGGAAACGAACGCTAATACCGCATACGTCCTACGGGAGAAAGCAGGGGACCTTCGGGCCTTGCGCTATCAGATGAGCCTAGGTCGGATTAGCTAGTTGGTGAGGTAATGGCTCACCAAGGCGACGATCCGTAACTGGTCTGAGAGGATGATCAGTCACACTGGAACTGAGACACGGTCCAGACTCCTACGGGAGGCAGCAGTGGGGAATATTGGACAATGGGCGAAAGCCTGATCCAGCCATGCCGCGTGTGTGAAGAAGGTCTTCGGATTGTAAAGCACTTTAAGTTGGGAGGAAGGGCAGTTACCTAATACGTGATTGTTTTGACGTTACCGACAGAATAAGCACCGGCTAACTCTGTGCCAGCAGCCGCGGTAATACAGAGGGTGCAAGCGTTAATCGGAATTACTGGGCGTAAAGCGCGCGTAGGTGGTTTGTTAAGTTGGATGTGAAATCCCCGGGCTCAACCTGGGAACTGCATTCAAAACTGACTGACTAGAGTATGGTAGAGGGTGGTGGAATTTCCTGTGTAGCGGTGAAATGCGTAGATATAGGAAGGAACACCAGTGGCGAAGGCGACCACCTGGACTAATACTGACACTGAGGTGCGAAAGCGTGGGGAGCAAACAGGATTAGATACCCTGGTAGTCCACGCCGTAAACGATGTCAACTAGCCGTTGGAAGCCTTGAGCTTTTAGTGGCGCAGCTAACGCATTAAGTTGACCGCCTGGGGAGTACGGCCGCAAGGTTAAAACTCAAATGAATTGACGGGGGCCCGCACAAGCGGTGGAGCATGTGGTTTAATTCGAAGCAACGCGAAGAACCTTACCAGGCCTTGACATCCAATGAACTTTCCAGAGATGGATTGGTGCCTTCGGGAACATTGAGACAGGTGCTGCATGGCTGTCGTCAGCTCGTGTCGTGAGATGTTGGGTTAAGTCCCGTAACGAGCGCAACCCTTGTCCTTAGTTACCAGCACGTCATGGTGGGCACTCTAAGGAGACTGCCGGTGACAAACCGGAGGAAGGTGGGGATGACGTCAAGTCATCATGGCCCTTACGGCCTGGGCTACACACGTGCTACAATGGTCGGTACAGAGGGTTGCCAAGCCGCGAGGTGGAGCTAATCCCATAAAACCGATCGTAGTCCGGATCGCAGTCTGCAACTCGACTGCGTGAAGTCGGAATCGCTAGTAATCGCGAATCAGAATGTCGCGGTGAATACGTTCCCGGGCCTTGTACACACCGCCCGTCACACCATGGGAGTGGGTTGCACCAGAAGTAGCTAGTCTAACCTTCGGGNGGACGGTN

>FW305-3-2-15-C-R2A1

GCAGTCGAGCGGTAGAGAGAAGCTTGCTTCTCTTGAGAGCGGCGGACGGGTGAGTAATGCCTAGGAATCTGCCTGGTAGTGGGGGATAACGTTCGGAAACGAACGCTAATACCGCATACGTCCTACGGGAGAAAGCAGGGGACCTTCGGGCCTTGCGCTATCAGATGAGCCTAGGTCGGATTAGCTAGTTGGTGAGGTAATGGCTCACCAAGGCGACGATCCGTAACTGGTCTGAGAGGATGATCAGTCACACTGGAACTGAGACACGGTCCAGACTCCTACGGGAGGCAGCAGTGGGGAATATTGGACAATGGGCGAAAGCCTGATCCAGCCATGCCGCGTGTGTGAAGAAGGTCTTCGGATTGTAAAGCACTTTAAGTTGGGAGGAAGGGCAGTTACCTAATACGTGATTGTTTTGACGTTACCGACAGAATAAGCACCGGCTAACTCTGTGCCAGCAGCCGCGGTAATACAGAGGGTGCAAGCGTTAATCGGAATTACTGGGCGTAAAGCGCGCGTAGGTGGTTTGTTAAGTTGGATGTGAAATCCCCGGGCTCAACCTGGGAACTGCATTCAAAACTGACTGACTAGAGTATGGTAGAGGGTGGTGGAATTTCCTGTGTAGCGGTGAAATGCGTAGATATAGGAAGGAACACCAGTGGCGAAGGCGACCACCTGGACTAATACTGACACTGAGGTGCGAAAGCGTGGGGAGCAAACAGGATTAGATACCCTGGTAGTCCACGCCGTAAACGATGTCAACTAGCCGTTGGAAGCCTTGAGCTTTTAGTGGCGCAGCTAACGCATTAAGTTGACCGCCTGGGGAGTACGGCCGCAAGGTTAAAACTCAAATGAATTGACGGGGGCCCGCACAAGCGGTGGAGCATGTGGTTTAATTCGAAGCAACGCGAAGAACCTTACCAGGCCTTGACATCCAATGAACTTTCCAGAGATGGATTGGTGCCTTCGGGAACATTGAGACAGGTGCTGCATGGCTGTCGTCAGCTCGTGTCGTGAGATGTTGGGTTAAGTCCCGTAACGAGCGCAACCCTTGTCCTTAGTTACCAGCACGTCATGGTGGGCACTCTAAGGAGACTGCCGGNGACAAACCGGAGGAAGGTGGGGATGACGTCNAGTCATCATNNCCCTTACGGCCNGGGCTACACANGNGCTACAATGGTCGGTNCAGAGGGTTGCCAAGCCGCGAGGTGGAGCTAATCCCATAAAACCGATCGTAGTCCGGATCGCAGTCTGCAACTCGACTGNGTGAAGTCGGAATCGCTAGTAATCGCGAATCAGAATGTCGCGGTGAATACGTTCCCGGGCCTTGTNNACACCGCCCGTCACACCATGGGAGTGGGTTGCACCAGAAG

>FW305-3-2-15-C-TSA1

TGCAGTCGAACGGCAGCACGGGCTTCGGCCTGGTGGCGAGTGGCGAACGGGTGAGTAATACATCGGAACGTGCCCTGTAGTGGGGGATAACTAGTCGAAAGATTAGCTAATACCGCATACGACCTGAGGGTGAAAGCGGGGGACCGCAAGGCCTCGCGCTACAGGAGCGGCCGATGTNTGATTAGCTAGTTGGTGGGGTAAAGGCCTACCAAGGCGACGATCAGTAGCTGGNCTGAGAGGACGATCAGCCACACTGGGACTGAGACACGGCCCNNACTCCTACGGGAGGCAGCAGTGGGGAATTTTGGACAATGGGGGCAACCCTGATCCAGCAATGCCGCGTGTGTGAAGAAGGCCTTCGGGTTGTAAAGCACTTTTGTCCGGAAAGAAATGGCTCTGGTTAATACCCGGGGTCGATGACGGTACCGGAAGAATAAGCACCGGCTAACTACGTGCCAGCAGCCGCGGTAATACGTAGGGTGCGAGCGTTAATCGGAATTACTGGGCGTAAAGCGTGCGCAGGCGGTTTTGTAAGACAGGCGTGAAATCCCCGAGCTCAACTTGGGAATGGCGCTTGTGACTGCAAGGCTAGAGTATGTCAGAGGGGGGTAGAATTCCACGTGTAGCAGTGAAATGCGTAGAGATGTGGAGGAATACCGATGGCGAAGGCAGCCCCCTGGGACGTCACTGACGCTCATGCACGAAAGCGTGGGGAGCAAACAGGATTAGATACCCTGGTAGTCCACGCCCTAAACGATGTCAACTAGTTGTTGGGGATTCATTTCTTCAGTAACGTAGCTAACGCGTGAAGTTGACCGCCTGGGGAGTACGGTCGCAAGATTAAAACTCAAAGGAATTGACGGGGACCCGCACAAGCGGTGGATGATGTGGATTAATTCGATGCAACGCGAAAAACCTTACCTACCCTTGACATGCCACTAACGAAGCAGAGATGCATCAGGTGCCCGAAAGGGAAAGTGGACACAGGTGCTGCATGGCTGTCGTCAGCTCGTGTCGTGAGATGTTGGGTTAAGTCCCGCAACGAGCGCAACCCTTGTCTCTAGTTGCTACGAAAGGGCACTCTAGAGAGACTGCCGGTGACAAACCGGAGGAAGGTGGGGATGACGTCAAGTCCTCATGGCCCTTATGGGTAGGGCTTCACACGTCATACAATGGTGCGTACAGAGGGTTGCCAACCCGCGAGGGGGAGCTAATCCCAGAAAACGCATCGTAGTCCGGATCGTAGTCTGCAACTCGACTACGTGAAGCTGGAATCGCTAGTAATCGCGGATCAGCATGCCGCGGTGAATACGTTCCCGGGTCTTGTACACACCGCCCGTCACACCATGGGAGTGGGTTTTGCCAGAAGTAGTTAGCCTAAC

>FW305-3-2-15-C-TSA2

TGCAAGTCGAGCGGTAGAGAGAAGCTTGCTTCTCTTGAGAGCGGCGGACGGGTGAGTAATGCCTAGGAATCTGCCTGGTAGTGGGGGATAACGTTCGGAAACGAACGCTAATACCGCATACGTCCTACGGGAGAAAGCAGGGGACCTTCGGGCCTTGCGCTATCAGATGAGCCTAGGTCGGATTAGCTAGTTGGTGAGGTAATGGCTCACCAAGGCGACGATCCGTAACTGGTCTGAGAGGATGATCAGTCACACTGGAACTGAGACACGGTCCAGACTCCTACGGGAGGCAGCAGTGGGGAATATTGGACAATGGGCGAAAGCCTGATCCAGCCATGCCGCGTGTGTGAAGAAGGTCTTCGGATTGTAAAGCACTTTAAGTTGGGAGGAAGGGCAGTTACCTAATACGTGATTGTTTTGACGTTACCGACAGAATAAGCACCGGCTAACTCTGTGCCAGCAGCCGCGGTAATACAGAGGGTGCAAGCGTTAATCGGAATTACTGGGCGTAAAGCGCGCGTAGGTGGTTTGTTAAGTTGGATGTGAAATCCCCGGGCTCAACCTGGGAACTGCATTCAAAACTGACTGACTAGAGTATGGTAGAGGGTGGTGGAATTTCCTGTGTAGCGGTGAAATGCGTAGATATAGGAAGGAACACCAGTGGCGAAGGCGACCACCTGGACTAATACTGACACTGAGGTGCGAAAGCGTGGGGAGCAAACAGGATTAGATACCCTGGTAGTCCACGCCGTAAACGATGTCAACTAGCCGTTGGAAGCCTTGAGCTTTTAGTGGCGCAGCTAACGCATTAAGTTGACCGCCTGGGGAGTACGGCCGCAAGGTTAAAACTCAAATGAATTGACGGGGGCCCGCACAAGCGGTGGAGCATGTGGTTTAATTCGAAGCAACGCGAAGAACCTTACCAGGCCTTGACATCCAATGAACTTTCCAGAGATGGATTGGTGCCTTCGGGAACATTGAGACAGGTGCTGCATGGCTGTCGTCAGCTCGTGTCGTGAGATGTTGGGTTAAGTCCCGTAACGAGCGCAACCCTTGTCCTTAGTTACCAGCACGTCATGGTGGGCACTCTAAGGAGACTGCCGGTGACAAACCGGAGGAAGGTGGGGATGACGTCAAGTCATCATGGCCCTTACGGCCTGGGCTACACACGTGCTACAATGGTCGGTACAGAGGGTTGCCAAGCCGCGAGGTGGAGCTAATCCCATAAAACCGATCGTAGTCCGGATCGCAGTCTGCAACTCGACTGCGTGAAGTCGGAATCGCTAGTAATCGCGAATCAGAATGTCGCGGTGAATACGTTCCCGGGCCTTGTACACACCGCCCGTCACACCATGGGAGTGGGTTGCACCAGAAGTAGCTAGTC

>FW305-3-2-15-C-TSA3

ATGCAGTCGAGCGGTAGAGAGAAGCTTGCTTCTCTTGAGAGCGGCGGACGGGTGAGTAATGCCTAGGAATCTGCCTGGTAGTGGGGGATAACGTTCGGAAACGAACGCTAATACCGCATACGTCCTACGGGAGAAAGCAGGGGACCTTCGGGCCTTGCGCTATCAGATGAGCCTAGGTCGGATTAGCTAGTTGGTGAGGTAATGGCTCACCAAGGCGACGATCCGTAACTGGTCTGAGAGGATGATCAGTCACACTGGAACTGAGACACGGTCCAGACTCCTACGGGAGGCAGCAGTGGGGAATATTGGACAATGGGCGAAAGCCTGATCCAGCCATGCCGCGTGTGTGAAGAAGGTCTTCGGATTGTAAAGCACTTTAAGTTGGGAGGAAGGGCAGTTACCTAATACGTGATTGTTTTGACGTTACCGACAGAATAAGCACCGGCTAACTCTGTGCCAGCAGCCGCGGTAATACAGAGGGTGCAAGCGTTAATCGGAATTACTGGGCGTAAAGCGCGCGTAGGTGGTTTGTTAAGTTGGATGTGAAATCCCCGGGCTCAACCTGGGAACTGCATTCAAAACTGACTGACTAGAGTATGGTAGAGGGTGGTGGAATTTCCTGTGTAGCGGTGAAATGCGTAGATATAGGAAGGAACACCAGTGGCGAAGGCGACCACCTGGACTAATACTGACACTGAGGTGCGAAAGCGTGGGGAGCAAACAGGATTAGATACCCTGGTAGTCCACGCCGTAAACGATGTCAACTAGCCGTTGGAAGCCTTGAGCTTTTAGTGGCGCAGCTAACGCATTAAGTTGACCGCCTGGGGAGTACGGCCGCAAGGTTAAAACTCAAATGAATTGACGGGGGCCCGCACAAGCGGTGGAGCATGTGGTTTAATTCGAAGCAACGCGAAGAACCTTACCAGGCCTTGACATCCAATGAACTTTCCAGAGATGGATTGGTGCCTTCGGGAACATTGAGACAGGTGCTGCATGGCTGTCGTCAGCTCGTGTCGTGAGATGTTGGGTTAAGTCCCGTAACGAGCGCAACCCTTGTCCTTAGTTACCAGCACGTCATGGTGGGCACTCTAAGGAGACTGCCGGTGACAAACCGGAGGAAGGTGGGGATGACGTCAAGTCATCATGGCCCTTACGGCCTGGGCTACACACGTGCTACAATGGTCGGTACAGAGGGTTGCCAAGCCGCGAGGTGGAGCTAATCCCATAAAACCGATCGTAGTCCGGATCGCAGTCTGCAACTCGACTGCGTGAAGTCGGAATCGCTAGTAATCGCGAATCAGAATGTCGCGGTGAATACGTTCCCGGGCCTTGTACACACCGCCCGTCACACCATGGGAGTGGGTTGCACCAGAAGTAGCTAGTCTAACCTTC

>FW305-3-2-15-D-LB1

TGCAGTCGAACGGCAGCACGGGAGCAATCCTGGTGGCGAGTGGCGAACGGGTGAGTAATACATCGGAACGTGCCCTGTCGTGGGGGATAACTAGTCGAAAGATTAGCTAATACCGCATACGACCTGAGGGTGAAAGCGGGGGACCGCAAGGCCTCGCGCGATAGGAGCGGCCGATGTCTGATTAGCTAGTTGGTGGGGTAAAGGCCCACCAAGGCGACGATCAGTAGCTGGTCTGAGAGGACGATCAGCCACACTGGGACTGAGACACGGCCCAGACTCCTACGGGAGGCAGCAGTGGGGAATTTTGGACAATGGGGGCAACCCTGATCCAGCAATGCCGCGTGTGTGAAGAAGGCCTTCGGGTTGTAAAGCACTTTTGTCCGGAAAGAAATCCCTTGCCCTAATACGGCGGGGGGATGACGGTACCGGAAGAATAAGCACCGGCTAACTACGTGCCAGCAGCCGCGGTAATACGTAGGGTGCGAGCGTTAATCGGAATTACTGGGCGTAAAGCGTGCGCAGGCGGTTTTGTAAGACAGGCGTGAAATCCCCGAGCTCAACTTGGGAATGGCGCTTGTGACTGCAAGGCTAGAGTATGTCAGAGGGGGGTAGAATTCCACGTGTAGCAGTGAAATGCGTAGAGATGTGGAGGAATACCGATGGCGAAGGCAGCCCCCTGGGACGTCACTGACGCTCATGCACGAAAGCGTGGGGAGCAAACAGGATTAGATACCCTGGTAGTCCACGCCCTAAACGATGTCAACTAGTTGTTGGGGATTCATTTCTTCAGTAACGTAGCTAACGCGTGAAGTTGACCGCCTGGGGAGTACGGTCGCAAGATTAAAACTCAAAGGAATTGACGGGGACCCGCACAAGCGGTGGATGATGTGGATTAATTCGATGCAACGCGAAAAACCTTACCTACCCTTGACATGCCACTAACGAAGCARAGATGCATCAGGTGCCCGAAAGGGAAAGTGGACACAGGTGCTGCATGGCTGTCGTCAGCTCGTGTCGTGAGATGTTGGGTTAAGTCCCGCAACGAGCGCAACCCTTGTCTTTAGTTGCTACGCAAGAGCACTCTARAGAGACTGCCGGTGACAAACCGGAGGAANGGTGGGGATGACGTCAAGTCCTCATGGCCCTTATGGGTAGGGCTTCACACGTCATACAATGGTGCGTACAGAGGGTTGCCAACCCGCGAGGGGGAGCCAATCCCAGAAAACGCATCGTAGTCCGGATCGTAGTCTGCAACTCGACTACGTGAAGCTGGAATCGCTAGTAATCGCGGATCAGCATGCCGCGGTGAATACGTTCCCGGGTCTTGTACACACCGCCCGTCACACCATGGGAGTGGGTTTTGCCAGAAGTAGTTAGCCTAACCGCAAGGAGGGCGA

>FW305-3-2-15-D-LB2

TGCAGTCGAACGGCAGCGCGGGAGCAATCCTGGCGGCGAGTGGCGAACGGGTGAGTAATACATCGGAACGTGCCCAATCGTGGGGGATAACGCAGCGAAAGCTGTGCTAATACCGCATACGATCTACGGATGAAAGCAGGGGATCGCAAGACCTTGCGCGAATGGAGCGGCCGATGGCAGATTAGGTAGTTGGTGAGGTAAAGGCTCACCAAGCCTTCGATCTGTAGCTGGTCTGAGAGGACGACCAGCCACACTGGGACTGAGACACGGCCCAGACTCCTACGGGAGGCAGCAGTGGGGAATTTTGGACAATGGGCGCAAGCCTGATCCAGCCATGCCGCGTGCAGGATGAAGSCCTTCGGGTTGTAAACTGCTTTTGTACGGAACGAAACGGTCTTTTCTAATAAAGAAGGCTAATGACGGTACCGTAAGAATAAGCACCGGCTAACTACGTGCCAGCAGCCGCGGTAATACGTAGGGTGCAAGCGTTAATCGGAATTACTGGGCGTAAAGCGTGCGCAGGCGGTAATGTAAGACAGTTGTGAAATCCCCGGGCTCAACCTGGGAACTGCATCTGTGACTGCATTGCTGGAGTACGGCAGAGGGGGATGGAATTCCGCGTGTAGCAGTGAAATGCGTAGATATGCGGAGGAACACCGATGGCGAAGGCAATCCCCTGGGCCTGTACTGACGCTCATGCACGAAAGCGTGGGGAGCAAACAGGATTAGATACCCTGGTAGTCCACGCCCTAAACGATGTCAACTGGTTGTTGGGAATTCACTTTCTCAGTAACGAAGCTAACGCGTGAAGTTGACCGCCTGGGGAGTACGGCCGCAAGGTTGAAACTCAAAGGAATTGACGGGGACCCGCACAAGCGGTGGATGATGTGGTTTAATTCGATGCAACGCGAAAAACCTTACCCACCTTTGACATGTACGGAATTCGCCAGAGATGGCTTAGTGCTCGAAAGAGAACCGTAACACAGGTGCTGCATGGCTGTCGTCAGCTCGTGTCGTGAGATGTTGGGTTAAGTCCCGCAACGAGCGCAACCCTTGTCATTAGTTGCTACATTTAGTTGGGCACTCTAATGAGACTGCCGGTGACAAACCGGAGGAAGGTGGGGATGACGTCAAGTCCTCATGGCCCTTATAGGTGGGGCTACACACGTCATACAATGGCTGGTACAAAGGGTTGCCAACCCGCGAGGGGGAGCTAATCCCATAAAACCAGTCGTAGTCCGGATCGCAGTCTGCAACTCGACTGCGTGAAGTCGGAATCGCTAGTAATCGTGGATCAGAATGTCACGGTGAATACGTTCCCGGGTCTTGTACACACCGCCCGTCACACCATGGGAGCGGGTTCTGCCAGAAGTAGTTAGCTTAACCGCAAGGAGGG

>FW305-3-2-15-D-TSA1

TGCAGTCGAACGGCAGCACGGGCTTCGGCCTGGTGGCGAGTGGCGAACGGGTGAGTAATACATCGGAACGTGCCCTGTAGTGGGGGATAACTAGTCGAAAGATTAGCTAATACCGCATACGACCTGAGGGTGAAAGCGGGGGACCGCAAGGCCTCGCGCTACAGGAGCGGCCGATGTCTGATTAGCTAGTNGGTGGGGTAAAGGCCTACCAAGGCGACGATCAGTAGCTGGTCTGAGAGGACGATCAGCCACACTGGGACTGAGACACGGCCCANACTCCTACGGGAGGCAGCAGTGGGGAATTTTGGACAATGGGGGCAACCCTGATCCAGCAATGCCGCGTGTGTGAAGAAGGCCTTCGGGTTGTAAAGCACTTTTGTCCGGAAAGAAATGGCTCTGGTTAATACCCGGGGTCGATGACGGTACCGGAAGAATAAGCACCGGCTAACTACGTGCCAGCAGCCGCGGTAATACGTAGGGTGCGAGCGTTAATCGGAATTACTGGGCGTAAAGCGTGCGCAGGCGGTTTTGTAAGACAGGCGTGAAATCCCCGAGCTCAACTTGGGAATGGCGCTTGTGACTGCAAGGCTAGAGTATGTCAGAGGGGGGTAGAATTCCACGTGTAGCAGTGAAATGCGTAGAGATGTGGAGGAATACCGATGGCGAAGGCAGCCCCCTGGGACGTCACTGACGCTCATGCACGAAAGCGTGGGGAGCAAACAGGATTAGATACCCTGGTAGTCCACGCCCTAAACGATGTCAACTAGTTGTTGGGGATTCATTTCTTCAGTAACGTAGCTAACGCGTGAAGTTGACCGCCTGGGGAGTACGGTCGCAAGATTAAAACTCAAAGGAATTGACGGGGACCCGCACAAGCGGTGGATGATGTGGATTAATTCGATGCAACGCGAAAAACCTTACCTACCCTTGACATGCCACTAACGAAGCAGAGATGCATCAGGTGCCCGAAAGGGAAAGTGGACACAGGTGCTGCATGGSTGTCGTCAGCTCKTGTCGTGAGATGTTGGGTTAAGTCCCGCAACGAGCGCAACCCTTGTCTCTAGTTGCTACGAAAGGGCACTCTAGAGAGACTGCCGGTGACAAACCGGAGGAAGGTGGGGATGACGTCAAGTCCTCATGGCCCTTATGGGTAGGGCTTCACACGTCATACAATGGTGCGTACAGAGGGTTGCCAACCCGCGAGGGGGAGCTAATCCCAGAAAACGCATCGTAGTCCGGATCGTAGTCTGCAACTCGACTACGTGAAGCTGGAATCGCTAGTAATCGCGGATCAGCATGCCGCGGTGAATACGTTCCCGGGTCTTGTACACACCGCCCGTCACACCATGGGAGTGGGTTTTGCCAGAAGTAGTTAGCCTAA

>FW305-3-2-15-D-TSA2

TGCAGTCGAACGGCAGCGCGGGAGCAATCCTGGCGGCGAGTGGCGAACGGGTGAGTAATACATCGGAACGTGCCCAATCGTGGGGGATAACGCAGCGAAAGCTGTGCTAATACCGCATACGATCTACGGATGAAAGCAGGGGATCGCAAGACCTTGCGCGAATGGAGCGGCCGATGGCAGATTAGGTAGTTGGTGAGGTAAAGGCTCACCAAGCCTTCGATCTGTAGCTGGTCTGAGAGGACGACCAGCCACACTGGGACTGAGACACGGCCCAGACTCCTACGGGAGGCAGCAGTGGGGAATTTTGGACAATGGGCGCAAGCCTGATCCAGCCATGCCGCGTGCAGGATGAAGGCCTTCGGGTTGTAAACTGCTTTTGTACGGAACGAAACGGTCTTTTCTAATAAAGAAGGCTAATGACGGTACCGTAAGAATAAGCACCGGCTAACTACGTGCCAGCAGCCGCGGTAATACGTAGGGTGCAAGCGTTAATCGGAATTACTGGGCGTAAAGCGTGCGCAGGCGGTAATGTAAGACAGTTGTGAAATCCCCGGGCTCAACCTGGGAACTGCATCTGTGACTGCATTGCTGGAGTACGGCAGAGGGGGATGGAATTCCGCGTGTAGCAGTGAAATGCGTAGATATGCGGAGGAACACCGATGGCGAAGGCAATCCCCTGGGCCTGTACTGACGCTCATGCACGAAAGCGTGGGGAGCAAACAGGATTAGATACCCTGGTAGTCCACGCCCTAAACGATGTCAACTGGTTGTTGGGAATTCACTTTCTCAGTAACGAAGCTAACGCGTGAAGTTGACCGCCTGGGGAGTACGGCCGCAAGGTTGAAACTCAAAGGAATTGACGGGGACCCGCACAAGCGGTGGATGATGTGGTTTAATTCGATGCAACGCGAAAAACCTTACCCACCTTTGACATGTACGGAATTCGCCAGAGATGGCTTAGTGCTCGAAAGAGAACCGTAACACAGGTGCTGCATGGCTGTCGTCAGCTCGTGTCGTGAGATGTTGGGTTAAGTCCCGCAACGAGCGCAACCCTTGTCATTAGTTGCTACATTTAGTTGGGCACTCTAATGAGACTGCCGGTGACAAACCGGAGGAAGGTGGGGATGACGTCAAGTCCTCATGGCCCTTATAGGTGGGGCTACACACGTCATACAATGGCTGGTACAAAGGGTTGCCAACCCGCGAGGGGGAGCTAATCCCATAAAACCAGTCGTAGTCCGGATCGCAGTCTGCAACTCGACTGCGTGAAGTCGGAATCGCTAGTAATCGTGGATCAGAATGTCACGGTGAATACGTTCCCGGGTCTTGTACACACCGCCCGTCACACCATGGGAGCGGGTTCTGCCAGAAGTAGTTAGCTTAA

>FW305-3-2-15-E-LB1

TGCAGTCGAACGGCAGCACAGTAAGAGCTTGCTCTTANGGGTGGCGAGTGGCGGACGGGTGAGGAATACATCGGAATCTACTTTTTCGTGGGGGATAACGTAGGGAAACTTACGCTAATACCGCATACGACCTACGGGTGAAAGCAGGGGACCTTCGGGCCTTGCGCGATTGAATGAGCCGATGTCGGATTAGCTAGTTGGCGGGGTAAAGGCCCACCAAGGCGACGATCCGTAGCTGGTCTGAGAGGATGATCAGCCACACTGGAACTGAGACACGGTCCAGACTCCTACGGGAGGCAGCAGTGGGGAATATTGGACAATGGGCGCAAGCCTGATCCAGCCATACCGCGNGGGTGAAGAANGCCTTCGGGTTGTAAAGNCCTTTTGTTGGGNAAGAAATCCAGCCGGTTAATACCTGGTTGGGATGACGGTACCCAAAGAATAAGCACCGGCTAACTTCGTGCCAGCAGCCGCGGTAATACGAAGGGTGCAAGCGTTACTCGGAATTACTGGGCGTAAAGCGTGCGTAGGTGGTTGTTTAAGTCTGTTGTGAAAGCCCTGGGCTCAACCTGGGAACTGCAGTGGAAACTGGACGACTAGAGTGTGGTAGAGGGTAGCGGAATTCCTGGTGTAGCAGTGAAATGCGTAGAGATCAGGAGGAACATCCATGGCGAAGGCAGCTACCTGGACCAACACTGACACTGAGGCACGAAAGCGTGGGGAGCAAACAGGATTAGATACCCTGGTAGTCCACGCCCTAAACGATGCGAACTGGATGTTGGGTGCAATTTGGCACGCAGTATCGAAGCTAACGCGTTAAGTTCGCCGCCTGGGGAGTACGGTCGCAAGACTGAAACTCAAAGGAATTGACGGGGGCCCGCACAAGCGGTGGAGTATGTGGTTTAATTCRATGCAACGCGAAGAACCTTACCTGGCCTTGACRTGTCGAGAACTTTCCAGAGATGGATTGGTGCCTTCGGGAACTCGAACACAGGTGCTGCATGGCTGTCGTCAGCTCGTGTCGTGAGATGTTGGGTTAAGTCCCGCAACGAGCGCAACCCTTGTCCTTAGTTGCCAGCACGTAATGGTGGGAACTCTAAGGAGACCGCCGGTGACAAACCGGAGGAAGGTGGGGATGACGTCAAGTCATCATGGCCCTTACGGCCAGGGCTACACACGTACTACAATGGTAGGGACAGAGGGCTGCAAGCCGGCGACGGTAAGCCAATCCCAGAAACCCTATCTCAGTCCGGATTGGAGTCTGCAACTCGACTCCATGAAGTCGGAATCGCTAGTAATCGCAGATCAGCATTGCTGCGGTGAATACGTTCCCGGGCCTTGTACACACCGCCCGTCACACCATGGGAGTTTGTTGCACCAGAAGCAGGTAGCTTAACCTTC

>FW305-3-2-15-E-R2A1

TGCAGTCGAGCGGATGAAGGAGCTTGCTCCTGGATTCAGCGGCGGACGGGTGAGTAATGCCTAGGAATCTGCCTGGTAGTGGGGGACAACGTTTCGAAAGGAACGCTAATACCGCATACGTCCTACGGGAGAAAGCAGGGGACCTTCGGGCCTTGCGCTATCAGATGAGCCTAGGTCGGATTAGCTAGTTGGTGAGGTAATGGCTCACCAAGGCGACGATCCGTAACTGGTCTGAGAGGATGATCAGTCACACTGGAACTGAGACACGGTCCAGACTCCTACGGGAGGCAGCAGTGGGGAATATTGGACAATGGGCGAAAGCCTGATCCAGCCATGCCGCGTGTGTGAAGAAGGTCTTCGGATTGTAAAGCACTTTAAGTTGGGAGGAAGGGTTGTAGATTAATACTCTGCAATTTTGACGTTACCGACAGAATAAGCACCGGCTAACTCTGTGCCAGCAGCCGCGGTAATACAGAGGGTGCAAGCGTTAATCGGAATTACTGGGCGTAAAGCGCGCGTAGGTGGTTTGTTAAGTTGGATGTGAAATCCCCGGGCTCAACCTGGGAACTGCATCCAAAACTGGCAAGCTAGAGTATGGTAGAGGGTGGTGGAATTTCCTGTGTAGCGGTGAAATGCGTAGATATAGGAAGGAACACCAGTGGCGAAGGCGACCACCTGGACTGATACTGACACTGAGGTGCGAAAGCGTGGGGAGCAAACAGGATTAGATACCCTGGTAGTCCACGCCGTAAACGATGTCAACTAGCCGTTGGGAGCCTTGAGCTCTTAGTGGCGCAGCTAACGCATTAAGTTGACCGCCTGGGGAGTACGGCCGCAAGGTTAAAACTCAAATGAATTGACGGGGGCCCGCACAAGCGGTGGAGCATGTGGTTTAATTCGAAGCAACGCGAAGAACCTTACCAGGCCTTGACATCCAATGAACTTTCCAGAGATGGATTGGTGCCTTCGGGAACATTGAGACAGGTGCTGCATGGCTGTCGTCAGCTCGTGTCGTGAGATGTTGGGTTAAGTCCCGTAACGAGCGCAACCCTTGTCCTTAGTTACCAGCACGTAATGGTGGGCACTCTAAGGAGACTGCCGGTGACAAACCGGAGGAAGGTGGGGATGACGTCAAGTCATCATGGCCCTTACGGCCTGGGCTACACACGTGCTACAATGGTCGGTACAGAGGGTTGCCAAGCCGCGAGGTGGAGCTAATCCCACAAAACCGATCGTAGTCCGGATCGCAGTCTGCAACTCGACTGCGTGAAGTCGGAATCGCTAGTAATCGCGAATCAGAATGTCGCGGTGAATACGTTCCCGGGCCTTGTACACACCGCCCGTCACACCATGGGAGTGGGTTGCACCAGAAGTAGC

>FW305-3-2-15-E-R2A2

TGCAGTCGANCGATAGTGAAGGGCTTGCTCTTCACGAAAGTGGCGCACGGGTGCGTAACGCGTATGCAACCTACCTTAATCAGGGGGATAGCCCGAAGAAATTCGGATTAACACCGCATAAAATCACAGTACAGCATTGTACAATGATCAAATATTTATAGGATTAAGATGGGCATGCGTGTCATTAGTTAGTTGGCGAGGTAACGGCTCACCAAGACCACGATGACTAGGGGATCTGAGAGGATGACCCCCCACACTGGTACTGAGACACGGACCAGACTCCTACGGGAGGCAGCAGTAAGGAATATTGGTCAATGGAGGCAACTCTGAACCAGCCATGCCGCGTGCAGGAAGACAGCCCTCTGGGTCGTAAACTGCTTTTATTCGGGAATAAACCTACTTACGTGTAAGTAGCTGAATGTACCGAAGGAATAAGGATCGGCTAACTCCGTGCCAGCAGCCGCGGTAATACGGAGGATCCAAGCGTTATCCGGATTTATTGGGTTTAAAGGGTGCGTAGGCGGCTTATTAAGTCAGGGGTGAAAGACGGTGGCTCAACCATCGCAGTGCCCTTGATACTGATGAGCTTGAATACACTAGAGGTAGGCGGAATGTGACAAGTAGCGGTGAAATGCATAGATATGTCACAGAACACCGATTGCGAAGGCAGCTTACTATGGTGTCATTGACGCTGAGGCACGAAAGCGTGGGGATCAAACAGGATTAGATACCCTGGTAGTCCACGCCCTAAACGATGAATACTCGCTGTTAGCGATACACAGTTAGCGGCTAAGCGAAAGCGTTAAGTATTCCACCTGGGGAGTACGCCCGCAAGGGTGAAACTCAAAGGAATTGACGGGGGCCCGCACAAGCGGAGGAGCATGTGGTTTAATTCGATGATACGCGAGGAACCTTACCCGGGCTTGAAAGTTAGTGAATGATCTAGAGATAGGTCAGTGAGCAATCACACGAAACTAGGTGCTGCATGGCTGTCGTCAGCTCGTGCCGTGAGGTGTTGGGTTAAGTCCCGCAACGAGCGCAACCCCTATGTTTAGTTGCCAGCACGTTAAGGTGGGGACTCTAAACAGACTGCCTGTGCAAACAGAGAGGAAGGAGGGGACGACGTCAAGTCATCATGGCCCTTACGTCCGGGGCTACACACGTGCTACAATGGATGGTACAGAGGGCAGCAAGCTGGCAACAGCAAGCAAATCTCAAAAAGCCATTCACAGTTCGGATAGAGGTCTGCAACTCGACCTCTTGAAGTTGGATTCGCTAGTAATCGCGTATCAGCAATGACGCGGTGAATACGTTCCCGGGCCTTGTACACACCGCCCGTCAAGCCATGGAAGTTGGGGGTACCTAAAGTATGTAACCGCAAGGAGC

>FW305-3-2-15-E-R2A3

TGCAGTCGAACGGCAGCACGGGAGCAATCCTGGTGGCGAGTGGCGAACGGGTGAGTAATACATCGGAACGTGCCCTGTCGTGGGGGATAACTAGTCGAAAGATTAGCTAATACCGCATACGACCTGAGGGTGAAAGCGGGGGACCGTAAGGCCTCGCGCGATAGGAGCGGCCGATGTCTGATTAGCTAGTTGGTGGGGTAAAGGCCCACCAAGGCGACGATCAGTAGCTGGTCTGAGAGGACGATCAGCCACACTGGGACTGAGACACGGCCCAGACTCCTACGGGAGGCAGCAGTGGGGAATTTTGGACAATGGGGGCAACCCTGATCCAGCAATGCCGCGTGTGTGAAGAAGGCCTTCGGGTTGTAAAGCACTTTTGTCCGGAAAGAAATCCCTTGCTCTAATACAGCGGGGGGATGACGGTACCGGAAGAATAAGCACCGGCTAACTACGTGCCAGCAGCCGCGGTAATACGTAGGGTGCGAGCGTTAATCGGAATTACTGGGCGTAAAGCGTGCGCAGGCGGTTTTGTAAGACAGGCGTGAAATCCCCGAGCTCAACTTGGGAATGGCGCTTGTGACTGCAAGGCTAGAGTATGTCAGAGGGGGGTAGAATTCCACGTGTAGCAGTGAAATGCGTAGAGATGTGGAGGAATACCGATGGCGAAGGCAGCCCCCTGGGACGTCACTGACGCTCATGCACGAAAGCGTGGGGAGCAAACAGGATTAGATACCCTGGTAGTCCACGCCCTAAACGATGTCAACTAGTTGTTGGGGATTCATTTCTTCAGTAACGTAGCTAACGCGTGAAGTTGACCGCCTGGGGAGTACGGTCGCAAGATTAAAACTCAAAGGAATTGACGGGGACCCGCACAAGCGGTGGATGATGTGGATTAATTCGATGCAACGCGAAAAACCTTACCTACCCTTGACATGCCACTAACGAAGCAGAGATGCATTAGGTGCCCGAAAGRGAAAGTGGACACAGGTGCTGCATGGCTGTCGTCAGCTCGTGTCGTGAGATGTTGGGTTAAGTCCCGCAACGAGCGCAACCCTTGTCTTTAGTTGCTACGCAAGAGCACTCTARAGAGACTGCCGGTGACAAACCGGAGGAAGGTGGGGATGACGTCAAGTCCTCATGGCCCTTATGGGTAGGGCTTCACACGTCATACAATGGTGCGTACAGAGGGTTGCCAACCCGCGAGGGGGAGCTAATCCCAGAAAACGCATCGTAGTCCGGATCGTAGTCTGCAACTCGACTACGTGAAGCTGGAATCGCTAGTAATCGCGGATCAGCATGCCGCGGTGAATACGTTCCCGGGTCTTGTACACACCGCCCGTCACACCATGGGAGTGGGTTTTGCCAGAAGTAGTTAGCCT

>FW305-3-2-15-E-TSA1

GGAGCTTGCTCCTGGATTCAGCGGCGGACGGGTGAGTAATGCCTAGGAATCTGCCTGGTAGTGGGGGACAACGTTTCGAAAGGAACGCTAATACCGCATACGTCCTACGGGAGAAAGCAGGGGACCTTCGGGCCTTGCGCTATCAGATGAGCCTAGGTCGGATTAGCTAGTTGGTGAGGTAATGGCTCACCAAGGCGACGATCCGTAACTGGTCTGAGAGGATGATCAGTCACACTGGAACTGAGACACGGTCCAGACTCCTACGGGAGGCAGCAGTGGGGAATATTGGACAATGGGCGAAAGCCTGATCCAGCCATGCCGCGTGTGTGAAGAAGGTCTTCGGATTGTAAAGCACTTTAAGTTGGGAGGAAGGGTTGTAGATTAATACTCTGCAATTTTGACGTTACCGACAGAATAAGCACCGGCTAACTCTGTGCCAGCAGCCGCGGTAATACAGAGGGTGCAAGCGTTAATCGGAATTACTGGGCGTAAAGCGCGCGTAGGTGGTTTGTTAAGTTGGATGTGAAATCCCCGGGCTCAACCTGGGAACTGCATCCAAAACTGGCAAGCTAGAGTATGGTAGAGGGTGGTGGAATTTCCTGTGTAGCGGTGAAATGCGTAGATATAGGAAGGAACACCAGTGGCGAAGGCGACCACCTGGACTGATACTGACACTGAGGTGCGAAAGCGTGGGGAGCAAACAGGATTAGATACCCTGGTAGTCCACGCCGTAAACGATGTCAACTAGCCGTTGGGAGCCTTGAGCTCTTAGTGGCGCAGCTAACGCATTAAGTTGACCGCCTGGGGAGTACGGCCGCAAGGTTAAAACTCAAATGAATTGACGGGGGCCCGCACAAGCGGTGGAGCATGTGGTTTAATTCGAAGCAACGCGAAGAACCTTACCAGGCCTTGACATCCAATGAACTTTCCAGAGATGGATTGGTGCCTTCGGGAACATTGAGACAGGTGCTGCATGGCTGTCGTCAGCTCGTGTCGTGAGATGTTGGGTTAAGTCCCGTAACGAGCGCAACCCTTGTCCTTAGTTACCAGCACGTAATGGTGGGCACTCTAAGGAGACTGCCGGTGACAAACCGGAGGAAGGTGGGGATGACGTCAAGTCATCATGGCCCTTACGGCCTGGGCTACACACGTGCTACAATGGTCGGTACAGAGGGTTGCCAAGCCGCGAGGTGGAGCTAATCCCACAAAACCGATCGTAGTCCGGATCGCAGTCTGCAACTCGACTGCGTGAAGTCGGAATCGCTAGTAATCGCGAATCAGAATGTCGCGGTGAATACGTTCCCGGGCCTTGTACACACCGCCCGTCACACCATGGGAGTGGGTTGCACCAGAAGTAGCTAGTCTAACCTTC

>FW305-3-2-15-E-TSA2

TGCAGTCGAGCGGATGAAGGAGCTTGCTCCTGGATTCAGCGGCGGACGGGTGAGTAATGCCTAGGAATCTGCCTGGTAGTGGGGGACAACGTTTCGAAAGGAACGCTAATACCGCATACGTCCTACGGGAGAAAGCAGGGGACCTTCGGGCCTTGCGCTATCAGATGAGCCTAGGTCGGATTAGCTAGTTGGTGAGGTAATGGCTCACCAAGGCGACGATCCGTAACTGGTCTGAGAGGATGATCAGTCACACTGGAACTGAGACACGGTCCAGACTCCTACGGGAGGCAGCAGTGGGGAATATTGGACAATGGGCGAAAGCCTGATCCAGCCATGCCGCGTGTGTGAAGAAGGTCTTCGGATTGTAAAGCACTTTAAGTTGGGAGRAAGGGTTGTAGATTAATACTCTGCAATTTTGACGTTACCGACAGAATAAGCACCGGCTAACTCTGTGCCAGCAGCCGCGGTAATACAGAGGGTGCAAGCGTTAATCGGAATTACTGGGCGTAAAGCGCGCGTAGGTGGTTTGTTAAGTTGGATGTGAAATCCCCGGGCTCAACCTGGGAACTGCATCCAAAACTGGCAAGCTAGAGTATGGTAGAGGGTGGTGGAATTTCCTGTGTAGCGGTGAAATGCGTAGATATAGGAAGGAACACCAGTGGCGAAGGCGACCACCTGGACTGATACTGACACTGAGGTGCGAAAGCGTGGGGAGCAAACAGGATTAGATACCCTGGTAGTCCACGCCGTAAACGATGTCAACTAGCCGTTGGGAGCCTTGAGCTCTTAGTGGCGCAGCTAACGCATTAAGTTGACCGCCTGGGGAGTACGGCCGCAAGGTTAAAACTCAAATGAATTGACGGGGGCCCGCACAAGCGGTGGAGCATGTGGTTTAATTCGAAGCAACGCGAAGAACCTTACCAGGCCTTGACATCCAATGAACTTTCCAGAGATGGATTGGTGCCTTCGGGAACATTGAGACAGGTGCTGCATGGCTGTCGTCAGCTCGTGTCGTGAGATGTTGGGTTAAGTCCCGTAACGAGCGCAACCCTTGTCCTTAGTTACCAGCACGTAATGGTGGGCACTCTAAGGAGACTGCCGGTGACAAACCGGAGGAAGGTGGGGATGACGTCAAGTCATCATGGCCCTTACGGCCTGGGCTACACACGTGCTACAATGGTCGGTACAGAGGGTTGCCAAGCCGCGAGGTGGAGCTAATCCCACAAAACCGATCGTAGTCCGGATCGCAGTCTGCAACTCGACTGCGTGAAGTCGGAATCGCTAGTAATCGCGAATCAGAATGTCGCGGTGAATACGTTCCCGGGCCTTGTACACACCGCCCGTCACACCATGGGAGTGGGTTGCACCAGAAGTAGCTAGTCTAACCNTCGGGAGGAC

>FW305-3-2-15-E-TSA3

TGCAGTCGNNCGGCAGCGCGGGAGCAATCCTGGCGGCGAGTGGCGAACGGGTGAGTAATACATCGGAACGTGCCCAATCGTGGGGGATAACGCAGCGAAAGCTGTGCTAATACCGCATACGATCTACGGATGAAAGCAGGGGATCGCAAGACCTTGCGCGAATGGAGCGGCCGATGGCAGATTAGGTAGTTGGTGAGGTAAAGGCTCACCAAGCCTTCGATCTGTAGCTGGTCTGAGAGGACGACCAGCCACACTGGGACTGAGACACGGCCCAGACTCCTACGGGAGGCAGCAGTGGGGAATTTTGGACAATGGGCGAAAGCCTGATCCAGCCATGCCGCGTGCAGGATGAAGGCCTTCGGGTTGTAAACTGCTTTTGTACGGAACGAAACGGTCTTTTCTAATACAGAAGGCTAATGACGGTACCGTAAGAATAAGCACCGGCTAACTACGTGCCAGCAGCCGCGGTAATACGTAGGGTGCAAGCGTTAATCGGAATTACTGGGCGTAAAGCGTGCGCAGGCGGTTATGTAAGACAGTTGTGAAATCCCCGGGCTCAACCTGGGAACTGCATCTGTGACTGCATAGCTAGAGTACGGTAGAGGGGGATGGAATTCCGCGTGTAGCAGTGAAATGCGTAGATATGCGGAGGAACACCGATGGCGAAGGCAATCCCCTGGACCTGTACTGACGCTCATGCACGAAAGCGTGGGGAGCAAACAGGATTAGATACCCTGGTAGTCCACGCCCTAAACGATGTCAACTGGTTGTTGGGTCTTCACTGACTCAGTAACGAAGCTAACGCGTGAAGTTGACCGCCTGGGGAGTACGGCCGCAAGGTTGAAACTCAAAGGAATTGACGGGGACCCGCACAAGCGGTGGATGATGTGGTTTAATTCGATGCAACGCGAAAAACCTTACCCACCTTTGACATGTACGGAATTCGCCAGAGATGGCTTAGTGCTCGAAAGAGAGCCGTAACACAGGTGCTGCATGGCTGTCGTCAGCTCGTGTCGTGAGATGTTGGGTTAAGTCCCGCAACGAGCGCAACCCTTGTCATTAGTTGCTACATTCAGTTGGGCACTCTAATGAGACTGCCGGTGACAAACCGGAGGAAGGTGGGGATGACGTCAAGTCCTCATGGCCCTTATAGGTGGGGCTACACACGTCATACAATGGCTGGTACAAAGGGTTGCCAACCCGCGAGGGGGAGCTAATCCCATAAAACCAGTCGTAGTCCGGATCGCAGTCTGCAACTCGACTGCGTGAAGTCGGAATCGCTAGTAATCGTGGATCAGAATGTCACGGTGAATACGTTCCCGGGTCTTGTACACACCGCCCGTCACACCATGGGAGCGGGTTC

>FW305-3-2-15-E-TSA4

CATGCAGTCGAGCGGATGAAGGAGCTTGCTCCTGGATTCAGCGGCGGACGGGTGAGTAATGCCTAGGAATCTGCCTGGTAGTGGGGGACAACGTTTCGAAAGGAACGCTAATACCGCATACGTCCTACGGGAGAAAGCAGGGGACCTTCGGGCCTTGCGCTATCAGATGAGCCTAGGTCGGATTAGCTAGTTGGTGAGGTAATGGCTCACCAAGGCGACGATCCGTAACTGGTCTGAGAGGATGATCAGTCACACTGGAACTGAGACACGGTCCAGACTCCTACGGGAGGCAGCAGTGGGGAATATTGGACAATGGGCGAAAGCCTGATCCAGCCATGCCGCGTGTGTGAAGAAGGTCTTCGGATTGTAAAGCACTTTAAGTTGGGAGGAAGGGTTGTAGATTAATACTCTGCAATTTTGACGTTACCGACAGAATAAGCACCGGCTAACTCTGTGCCAGCAGCCGCGGTAATACAGAGGGTGCAAGCGTTAATCGGAATTACTGGGCGTAAAGCGCGCGTAGGTGGTTTGTTAAGTTGGATGTGAAATCCCCGGGCTCAACCTGGGAACTGCATCCAAAACTGGCAAGCTAGAGTATGGTAGAGGGTGGTGGAATTTCCTGTGTAGCGGTGAAATGCGTAGATATAGGAAGGAACACCAGTGGCGAAGGCGACCACCTGGACTGATACTGACACTGAGGTGCGAAAGCGTGGGGAGCAAACAGGATTAGATACCCTGGTAGTCCACGCCGTAAACGATGTCAACTAGCCGTTGGGAGCCTTGAGCTCTTAGTGGCGCAGCTAACGCATTAAGTTGACCGCCTGGGGAGTACGGCCGCAAGGTTAAAACTCAAATGAATTGACGGGGGCCCGCACAAGCGGTGGAGCATGTGGTTTAATTCGAAGCAACGCGAAGAACCTTACCAGGCCTTGACATCCAATGAACTTTCCAGAGATGGATTGGTGCCTTCGGGAACATTGAGACAGGTGCTGCATGGCTGTCGTCAGCTCGTGTCGTGAGATGTTGGGTTAAGTCCCGTAACGAGCGCAACCCTTGTCCTTAGTTACCAGCACGTAATGGTGGGCACTCTAAGGAGACTGCCGGTGACAAACCGGAGGAAGGTGGGGATGACGTCAAGTCATCATGGCCCTTACGGCCTGGGCTACACACGTGCTACAATGGTCGGTACAGAGGGTTGCCAAGCCGCGAGGTGGAGCTAATCCCACAAAACCGATCGTAGTCCGGATCGCAGTCTGCAACTCGACTGCGTGAAGTCGGAATCGCTAGTAATCGCGAATCAGAATGTCGCGGTGAATACGTTCCCGGGCCTTGTACACACCGCCCGTCACACCATGGGAGTGGGTTGCACCAGAAGTAGCTAGTCTAACCTTCGGGAGGAC

>FW305-3-2-15-F-LB1

TGCAGTCGAACGGTAACAGGCCGCAAGGTGCTGACGAGTGGCGAACGGGTGAGTAATGCATCGGAACGTGCCCAGTCGTGGGGGATAACGCAGCGAAAGCTGCGCTAATACCGCATACGATCTATGGATGAAAGCGGGGGACCGTAAGGCCTCGCGCGATTGGAGCGGCCGATGTCAGATTAGGTAGTTGGTGGGGTAAAGGCTCACCAAGCCGACGATCTGTAGCTGGTCTGAGAGGACGACCAGCCACACTGGGACTGAGACACGGCCCAGACTCCTACGGGAGGCAGCAGTGGGGAATTTTGGACAATGGGCGCAAGCCTGATCCAGCAATGCCGCGTGCAGGAAGAAGGCCTTCGGGTTGTAAACTGCTTTTGTACGGAACGAAAAGGCTCTGGTTAATACCTGGGGCACATGACGGTACCGTAAGAATAAGCACCGGCTAACTACGTGCCAGCAGCCGCGGTAATACGTAGGGTGCAAGCGTTAATCGGAATTACTGGGCGTAAAGCGTGCGCAGGCGGTTTTGTAAGACAGGCGTGAAATCCCCGGGCTCAACCTGGGAATTGCGCTTGTGACTGCAAGGCTGGAGTGCGGCAGAGGGGGATGGAATTCCGCGTGTAGCAGTGAAATGCGTAGATATGCGGAGGAACACCGATGGCGAAGGCAATCCCCTGGGCCTGCACTGACGCTCATGCACGAAAGCGTGGGGAGCAAACAGGATTAGATACCCTGGTAGTCCACGCCCTAAACGATGTCAACTGGTTGTTGGGTCTCTTCTGACTCAGTAACGAAGCTAACGCGTGAAGTTGACCGCCTGGGGAGTACGGCCGCAAGGTTGAAACTCAAAGGAATTGACGGGGACCCGCACAAGCGGTGGATGATGTGGTTTAATTCGATGCAACGCGAAAAACCTTACCCACCTTTGACATGTACGGAATTTGCCAGAGATGGCTTAGTGCTCGAAAGAGAGCCGTAACACAGGTGCTGCATGGCTGTCGTCAGCTCGTGTCGTGAGATGTTGGGTTAAGTCCCGCAACGAGCGCAACCCTTGTCATTAGTTGCTACGAAAGGGCACTCTAATGAGACTGCCGGTGACAAACCGGAGGAAGGTGGGGATGACGTCAAGTCCTCATGGCCCTTATAGGTGGGGCTACACACGTCATACAATGGCTGGTACAAAGGGTTGCCAACCCGCGAGGGGGAGCTAATCCCATAAAGCCAGTCGTAGTCCGGATCGCAGTCTGCAACTCGACTGCGTGAAGTCGGAATCGCTAGTAATCGTGGATCAGCATGTCACGGTGAATACGTTCCCGGGTCTTGTACACACCGCCCGTCACACCATGGGAGCGGGTCTCGCCAGAAGTAGTTAGCCTAACCGCAAGGAGGGCGA

>FW305-3-2-15-F-LB2

CTTGCTTGGTGGATCAGTGGCGAACGGGTGAGTAACACGTGAGCAACCTGCCCTGGACTCTGGGATAAGCGCTGGAAACGGTGTCTAATACTGGATATGAGCTCTCATCGCATGGTGGGGGTTGGAAAGATTTTTCGGTCTGGGATGGGCTCGCGGCCTATCAGCTTGTTGGTGAGGTAATGGCTCACCAAGGCGTCGACGGGTAGCCGGCCTGAGAGGGTGACCGGCCACACTGGGACTGAGACACGGCCCAGACTCCTACGGGAGGCAGCAGTGGGGAATATTGCACAATGGGCGGAAGCCTGATGCAGCAACGCCGCGTGAGGGATGACGGCCTTCGGGTTGTAAACCTCTTTTAGCAGGGAAGAAGCGAGAGTGACGGTACCTGCAGAAAAAGCGCCGGCTAACTACGTGCCAGCAGCCGCGGTAATACGTAGGGCGCAAGCGTTATCCGGAATTATTGGGCGTAAAGAGCTCGTAGGCGGTTTGTCGCGTCTGCTGTGAAATCCCGAGGCTCAACCTCGGGCCTGCAGTGGGTACGGGCAGACTAGAGTGCGGTAGGGGAGATTGGAATTCCTGGTGTAGCGGTGGAATGCGCAGATATCAGGAGGAACACCGATGGCGAAGGCAGATCTCTGGGCCGTAACTGACGCTGAGGAGCGAAAGGGTGGGGAGCAAACAGGCTTAGATACCCTGGTAGTCCACCMCGTAAACGTTGGGAACTAGTTGTGGGGACCATTCCACGGTTTCCGTGACGCAGCTAACGCATTAAGTTCCCCGCCTGGGGAGTACGGCCGCAAGGCTAAAACTCARAGGAATTGACGGGGACCCGCACAAGCGGCGGAGCATGCGGATTAATTCGATGCAACGCGAAGAACCTTACCAAGGCTTGACATACACCAGAACACCGTASAAATACGGGACTCTTTGGACACTGGTGAACAGGTGGTGCATGGTTGTCGYCAGCTCGTGTCKTGAGATGTTGGGTTAAKTCCCGCAMCGAGCGCAACCCTCGTTCTATGTTGCCAGCACGTAATGGTGGGAACTCATGGGATACTGCCGGGGTCAACTCGGAGGAAGGTGGGGATGACGTCAAATCATCATGCCCCTTATGTCTTGGGCTTCACGCATGCTACAATGGCCGGTACAAAGGGCTGCAATACCGTGAGGTGGAGCGAATCCCAAAAAGCCGGTCCCAGTTCGGATTGAGGTCTGCAACTCGACCTCATGAAGTCGGAGTCGCTAGTAATCGCAGATCAGCAACGCTGCGGTGAATACGTTCCCGGGTCTTGTACACACCGCCCGTCAAGTCATGAAAGTCGGTAACACCTGAAGCCGGTGGCCCAACCC

>FW305-3-2-15-F-LB3

TGCAGTCGAACGGCAGCACGGGAGCAATCCTGGTGGCGAGTGGCGAACGGGTGAGTAATACATCGGAACGTGCCCTGTCGTGGGGGATAACTAGTCGAAAGATTAGCTAATACCGCATACGACCTGAGGGTGAAAGCGGGGGACCGTAAGGCCTCGCGCGATAGGAGCGGCCGATGTCTGATTAGCTAGTTGGTGGGGTAAAGGCCCACCAAGGCGACGATCAGTAGCTGGTCTGAGAGGACGATCAGCCACACTGGGACTGAGACACGGCCCAGACTCCTACGGGAGGCAGCAGTGGGGAATTTTGGACAATGGGGGCAACCCTGATCCAGCAATGCCGCGTGTGTGAAGAAGGCCTTCGGGTTGTAAAGCACTTTTGTCCGGAAAGAAATCCCTTGCTCTAATACAGCGGGGGGATGACGGTACCGGAAGAATAAGCACCGGCTAACTACGTGCCAGCAGCCGCGGTAATACGTAGGGTGCGAGCGTTAATCGGAATTACTGGGCGTAAAGCGTGCGCAGGCGGTTTTGTAAGACAGGCGTGAAATCCCCGAGCTCAACTTGGGAATGGCGCTTGTGACTGCAAGGCTAGAGTATGTCAGAGGGGGGTAGAATTCCACGTGTAGCAGTGAAATGCGTAGAGATGTGGAGGAATACCGATGGCGAAGGCAGCCCCCTGGGACGTCACTGACGCTCATGCACGAAAGCGTGGGGAGCAAACAGGATTAGATACCCTGGTAGTCCACGCCCTAAACGATGTCAACTAGTTGTTGGGGATTCATTTCTTCAGTAACGTAGCTAACGCGTGAAGTTGACCGCCTGGGGAGTACGGTCGCAAGATTAAAACTCAAAGGAATTGACGGKGACCCGCACAAGCGGTGGATGATGTGGATTAATTCGATGCAWCGCGAAAAACCTTACCTACYYTTGACATGCCACTAACGAAGCAGAGATGCATTAGGTGNCCGAAANGGAAAGTGGACACAGGTGCTGCATGGCTGTCGTCAGCTCGTGTCGTGAGATGTTGGGTTAAGTCCCGCAACGAGCGCAACCCTTGTCTTTAGTTGCTACGCAAGAGCACTCTAGAGAGACTGCCGGTGACAAACCGGAGGAAGGTGGGGATGACGTCAAGTCCTCATGGCCCTTATGGGTAGGGCTTCACACGTCATACAATGGTGCGTACAGAGGGTTGCCAACCCGCGAGGGGGAGCTAATCCCAGAAAACGCATCGTAGTCCGGATCGTAGTCTGCAACTCGACTACGTGAAGCTGGAATCGCTAGTAATCGCGGATCAGCATGCCGCGGTGAATACGTTCCCGGGTCTTGTACACACCGCCCGTCACACCATGGGAGTGGGTTTTGCCAGAAGTAGTTAGCCTAACCGCAAGGAGGGCG

>FW305-3-2-15-F-LB4

ACGGGAGCAATCCTGGTGGCGAGTGGCGAACGGGTGAGTAATACATCGGAACGTGCCCTGTCGTGGGGGATAACTAGTCGAAAGATTAGCTAATACCGCATACGACCTGAGGGTGAAAGCGGGGGACCGTAAGGCCTCGCGCGATAGGAGCGGCCGATGTCTGATTAGCTAGTTGGTGGGGTAAAGGCCCACCAAGGCGACGATCAGTAGCTGGTCTGAGAGGACGATCAGCCACACTGGGACTGAGACACGGCCCAGACTCCTACGGGAGGCAGCAGTGGGGAATTTTGGACAATGGGGGCAACCCTGATCCAGCAATGCCGCGTGTGTGAAGAAGGCCTTCGGGTTGTAAAGCACTTTTGTCCGGAAAGAAATCCCTTGCTCTAATACAGCGGGGGGATGACGGTACCGGAAGAATAAGCACCGGCTAACTACGTGCCAGCAGCCGCGGTAATACGTAGGGTGCGAGCGTTAATCGGAATTACTGGGCGTAAAGCGTGCGCAGGCGGTTTTGTAAGACAGGCGTGAAATCCCCGAGCTCAACTTGGGAATGGCGCTTGTGACTGCAAGGCTAGAGTATGTCAGAGGGGGGTAGAATTCCACGTGTAGCAGTGAAATGCGTAGAGATGTGGAGGAATACCGATGGCGAAGGCAGCCCCCTGGGACGTCACTGACGCTCATGCACGAAAGCGTGGGGAGCAAACAGGATTAGATACCCTGGTAGTCCACGCCCTAAACGATGTCAACTAGTTGTTGGGGATTCATTTCTTCAGTAACGTAGCTAACGCGTGAAGTTGACCGCCTGGGGAGTACGGTCGCAAGATTAAAACTCAAAGGAATTGACGGGGACCCGCACAAGCGGTGGATGATGTGGATTAATTCGATGCAACGCGAAAAACCTTACCTACCCTTGACATGCCACTAACGAAGCAGAGATGCATCAGGTGCCCGAAAGGGAAAGTGGACACAGGTGCTGCATGGCTGTCGTCAGCTCGTGTCGTGAGATGTTGGGTTAAGTCCCGCAACGAGCGCAACCCTTGTCTTTAGTTGCTACGCAAGARCACTCTAGAGAGACTGCCGGTGACAAACCGGAGGAAGGTGGGGATGACGTCAAGTCCTCATGGCCCTTATGGGTAGGGCTTCACACGTCATACAATGGTGCGTACAGAGGGTTGCCAACCCGCGAGGGGGAGCTAATCCCAGAAAACGCATCGTAGTCCGGATCGTAGTCTGCAACTCGACTACGTGAAGCTGGAATCGCTAGTAATCGCGGATCAGCATGCCGCGGTGAATACGTTCCCGGGTCTTGTACACACCGCCCGTCACACCATGGGAGTGGGTTTTGCCAGAAGTAGTTAGCCTAACCGCAAGGAGGGCG

>FW305-3-2-15-F-R2A1

TGCAGTCGANCGGTGAAGCCNAGCTTGCTTGGTGGATCAGTGGCGAACGGGTGAGTAACACGTGAGCAACCTGCCCTGGACTCTGGGATAAGCGCTGGAAACGGTGTCTAATACTGGATATGAGCTCTCATCGCATGGTGGGGGTTGGAAAGATTTTTCGGTCTGGGATGGGCTCGCGGCCTATCAGCTTGTTGGTGAGGTAATGGCTCACCAAGGCGTCGACGGGTAGCCGGCCTGAGAGGGTGACCGGCCACACTGGGACTGAGACACGGCCCAGACTCCTACGGGAGGCAGCAGTGGGGAATATTGCACAATGGGCGGAAGCCTGATGCAGCAACGCCGCGTGAGGGATGACGGCCTTCGGGTTGTAAACCTCTTTTAGCAGGGAAGAAGCGAGAGTGACGGTACCTGCAGAAAAAGCGCCGGCTAACTACGTGCCAGCAGCCGCGGTAATACGTAGGGCGCAAGCGTTATCCGGAATTATTGGGCGTAAAGAGCTCGTAGGCGGTTTGTCGCGTCTGCTGTGAAATCCCGAGGCTCAACCTCGGGCCTGCAGTGGGTACGGGCAGACTAGAGTGCGGTAGGGGAGATTGGAATTCCTGGTGTAGCGGTGGAATGCGCAGATATCAGGAGGAACACCGATGGCGAAGGCAGATCTCTGGGCCGTAACTGACGCTGAGGAGCGAAAGGGTGGGGAGCAAACAGGCTTAGATACCCTGGTAGTCCACCCCGTAAACGTTGGGAACTAGTTGTGGGGACCATTCCACGGTTTCCGTGACGCAGCTAACGCATTAAGTTCCCCGCCTGGGGAGTACGGCCGCAAGGCTAAAACTCAAAGGAATTGACGGGGACCCGCACAAGCGGCGGAGCATGCGGATTAATTCGATGCAACGCGAAGAACCTTACCAAGGCTTGACATACACCAGAACACCGTAGAAATACGGGACTCTTTGGACACTGGTGAACAGGTGGTGCATGGTTGTCGTCAGCTCGTGTCGTGAGATGTTGGGTTAAGTCCCGCAACGAGCGCAACCCTCGTTCTATGTTGCCAGCACGTAATGGTGGGAACTCATGGGATACTGCCGGGGTCAACTCGGAGGAAGGTGGGGATGACGTCAAATCATCATGCCCCTTATGTCTTGGGCTTCACGCATGCTACAATGGCCGGTACAAAGGGCTGCAATACCGTGAGGTGGAGCGAATCCCAAAAAGCCGGTCCCAGTTCGGATTGAGGTCTGCAACTCGACCTCATGAAGTCGGAGTCGCTAGTAATCGCAGATCAGCAACGCTGCGGTGAATACGTTCCCGGGTCTTGTACACACCGCCCGTCAAGTCATGAAAGTCGGTAACACCTGAAGCCGGTGGCCCAA

>FW305-3-2-15-F-R2A2

GCAGTCGAACGGCAGCACGGGAGCAATCCTGGTGGCGAGTGGCGAACGGGTGAGTAATACATCGGAACGTGCCCTGTCGTGGGGGATAACTAGTCGAAAGATTAGCTAATACCGCATACGACCTGAGGGTGAAAGCGGGGGACCGTAAGGCCTCGCGCGATAGGAGCGGCCGATGTCTGATTAGCTAGTTGGTGGGGTAAAGGCCCACCAAGGCGACGATCAGTAGCTGGTCTGAGAGGACGATCAGCCACACTGGGACTGAGACACGGCCCAGAYTCCTACGGGAGGCAGCAGTGGGGAATTTTGGACAATGGGGGCAACCCTGATCCAGCAATGCCGCGTGTGTGAAGAAGGCCTTCGGGTTGTAAAGCACTTTTGTCCGGAAAGAAATCCCTTGCTCTAATACAGCGGGGGGATGACGGTACCGGAAGAATAAGCACCGGCTAACTACGTGCCAGCAGCCGCGGTAATACGTAGGGTGCGAGCGTTAATCGGAATTACTGGGCGTAAAGCGTGCGCAGGCGGTTTTGTAAGACAGGCGTGAAATCCCCGAGCTCAACTTGGGAATGGCGCTTGTGACTGCAAGGCTAGAGTATGTCAGAGGGGGGTAGAATTCCACGTGTAGCAGTGAAATGCGTAGAGATGTGGAGGAATACCGATGGCGAAGGCAGCCCCCTGGGACGTCACTGACGCTCATGCACGAAAGCGTGGGGAGCAAACAGGATTAGATACCCTGGTAGTCCACGCCCTAAACGATGTCAACTAGTTGTTGGGGATTCATTTCTTCAGTAACGTAGCTAACGCGTGAAGTTGACCGCCTGGGGAGTACGGTCGCAAGATTAAAACTCAAAGGAATTGACGGGGACCCGCACAAGCGGTGGATGATGTGGATTAATTCGATGCAACGCGAAAAACCTTACCTACCCTTGACATGCCACTAACGAAGCAGAGATGCATCAGGTGCCCGAAAGGGAAAGTGGACACAGGTGCTGCATGGCTGTCGTCAGCTCGTGTCGTGAGATGTTGGGTTAAGTCCCGCAACGAGCGCAACCCTTGTCTTTAGTTGCTACGCAAGAGCACTCTAGAGAGACTGCCGGTGACAAACCGGAGGAAGGTGGGGATGACGTCAAGTCCTCATGGCCCTTATGGGTAGGGCTTCACACGTCATACAATGGTGCGTACAGAGGGTTGCCAACCCGCGAGGGGGAGCTAATCCCAGAAAACGCATCGTAGTCCGGATCGTAGTCTGCAACTCGACTACGTGAAGCTGGAATCGCTAGTAATCGCGGATCAGCATGCCGCGGTGAATACGTTCCCGGGTCTTGTACACACCGCCCGTCACACCATGGGAGTGGGTTTTGCCAGAAGTAGTTAGCCTAA

>FW305-3-2-15-F-R2A3

TGCAGTCGAACGGTAACAGGCCGCAAGGTGCTGACGAGTGGCGAACGGGTGAGTAATGCATCGGAACGTGCCCAGTCGTGGGGGATAACGCAGCGAAAGCTGCGCTAATACCGCATACGATCTATGGATGAAAGCGGGGGACCGTAAGGCCTCGCGCGATTGGAGCGGCCGATGTCAGATTAGGTAGTTGGTGGGGTAAAGGCTCACCAAGCCGACGATCTGTAGCTGGTCTGAGAGGACGACCAGCCACACTGGGACTGAGACACGGCCCAGACTCCTACGGGAGGCAGCAGTGGGGAATTTTGGACAATGGGCGCAAGCCTGATCCAGCAATGCCGCGTGCAGGAAGAAGGCCTTCGGGTTGTAAACTGCTTTTGTACGGAACGAAAAGGCTCTGGTTAATACCTGGGGCACATGACGGTACCGTAAGAATAAGCACCGGCTAACTACGTGCCAGCAGCCGCGGTAATACGTAGGGTGCAAGCGTTAATCGGAATTACTGGGCGTAAAGCGTGCGCAGGCGGTTTTGTAAGACAGGCGTGAAATCCCCGGGCTCAACCTGGGAATTGCGCTTGTGACTGCAAGGCTGGAGTGCGGCAGAGGGGGATGGAATTCCGCGTGTAGCAGTGAAATGCGTAGATATGCGGAGGAACACCGATGGCGAAGGCAATCCCCTGGGCCTGCACTGACGCTCATGCACGAAAGCGTGGGGAGCAAACAGGATTAGATACCCTGGTAGTCCACGCCCTAAACGATGTCAACTGGTTGTTGGGTCTCTTCTGACTCAGTAACGAAGCTAACGCGTGAAGTTGACCGCCTGGGGAGTACGGCCGCAAGGTTGAAACTCAAAGGAATTGACGGGGACCCGCACAAGCGGTGGATGATGTGGTTTAATTCGATGCAACGCGAAAAACCTTACCCACCTTTGACATGTACGGAATTTGCCAGAGATGGCTTAGTGCTCGAAAGAGAGCCGTAACACAGGTGCTGCATGGCTGTCGTCAGCTCGTGTCGTGAGATGTTGGGTTAAGTCCCGCAACGAGCGCAACCCTTGTCATTAGTTGCTACGAAAGGGCACTCTAATGAGACTGCCGGTGACAAACCGGAGGAAGGTGGGGATGACGTCAAGTCCTCATGGCCCTTATAGGTGGGGCTACACACGTCATACAATGGCTGGTACAAAGGGTTGCCAACCCGCGAGGGGGAGCTAATCCCATAAAGCCAGTCGTAGTCCGGATCGCAGTCTGCAACTCGACTGCGTGAAGTCGGAATCGCTAGTAATCGTGGATCAGCATGTCACGGTGAATACGTTCCCGGGTCTTGTACACACCGCCCGTCACACCATGGGAGCGGGTCTCGCCAGAAGTAGTTAGCCTAACCGCAAGGAGGGCGAT

>FW305-3-2-15-F-R2A4

TGCAGTCGAACGGTAACAGGTCTTCGGATGCTGACGAGTGGCGAACGGGTGAGTAATACATCGGAACGTGCCCGATCGTGGGGGATAACGGAGCGAAAGCTTTGCTAATACCGCATACGATCTACGGATGAAAGCAGGGGACCGCAAGGCCTTGCGCGGACGGAGCGGCCGATGGCAGATTAGGTAGTTGGTGGGATAAAAGCTTACCAAGCCGACGATCTGTAGCTGGTCTGAGAGGACGACCAGCCACACTGGGACTGAGACACGGCCCAGACTCCTACGGGAGGCAGCAGTGGGGAATTTTGGACAATGGGCGAAAGCCTGATCCAGCCATGCCGCGTGCAGGATGAAGGCCTTCGGGTTGTAAACTGCTTTTGTACGGAACGAAAAGACTCTTTCTAATAAAGAGGGTCCATGACGGTACCGTAAGAATAAGCACCGGCTAACTACGTGCCAGCAGCCGCGGTAATACGTAGGGTGCAAGCGTTAATCGGAATTACTGGGCGTAAAGCGTGCGCAGGCGGTTATGTAAGACAGATGTGAAATCCCCGGGCTCAACCTGGGAACTGCATTTGTGACTGCATAGCTAGAGTACGGCAGAGGGGGATGGAATTCCGCGTGTAGCAGTGAAATGCGTAGATATGCGGAGGAACACCGATGGCGAAGGCAATCCCCTGGGCCTGTACTGACGCTCATGCACGAAAGCGTGGGGAGCAAACAGGATTAGATACCCTGGTAGTCCACGCCCTAAACGATGTCAACTGGTTGTTGGGTCTTCACTGACTCAGTAACGAAGCTAACGCGTGAAGTTGACCGCCTGGGGAGTACGGCCGCAAGGTTGAAACTCAAAGGAATTGACGGGGACCCGCACAAGCGGTGGATGATGTGGTTTAATTCGATGCAACGCGAAAAACCTTACCCACCTTTGACATGTACGGAATCCTTTAGAGATAGAGGAGTGCTCGAAAGAGAACCGTAACACAGGTGCTGCATGGCTGTCGTCAGCTCGTGTCGTGAGATGTTGGGTTAAGTCCCGCAACGAGCGCAACCCTTGCCATTAGTTGCTACGAAAGGGCACTCTAATGGGACTGCCGGTGACAAACCGGAGGAAGGTGGGGATGACGTCAAGTCCTCATGGCCCTTATAGGTGGGGCTACACACGTCATACAATGGCTGGTACAGAGGGTTGCCAACCCGCGAGGGGGAGCCAATCCCATAAAGCCAGTCGTAGTCCGGATCGCAGTCTGCAACTCGACTGCGTGAAGTCGGAATCGCTAGTAATCGCGGATCAGAATGTCGCGGTGAATACGTTCCCGGGTCTTGTACACACCGCCCGTCACACCATGGGAGCGGGTTCTGCCAGAAGTAGTTAGCCTAA

>FW305-3-2-15-F-TSA1

TGCAGTCGAGCGGTAGAGAGAAGCTTGCTTCTCTTGAGAGCGGCGGACGGGTGAGTAATGCCTAGGAATCTGCCTGGTAGTGGGGGATAACGTTCGGAAACGGACGCTAATACCGCATACGTCCTACGGGAGAAAGCAGGGGACCTTCGGGCCTTGCGCTATCAGATGAGCCTAGGTCGGATTAGCTAGTTGGTGGGGTAATGGCTCACCAAGGCGACGATCCGTAACTGGTCTGAGAGGATGATCAGTCACACTGGAACTGAGACACGGTCCAGACTCCTACGGGAGGCAGCAGTGGGGAATATTGGACAATGGGCGAAAGCCTGATCCAGCCATGCCGCGTGTGTGAAGAAGGTCTTCGGATTGTAAAGCACTTTAAGTTGGGAGGAAGGGCAGTTACCTAATACGTGATTGTTTTGACGTTACCGACAGAATAAGCACCGGCTAACTCTGTGCCAGCAGCCGCGGTAATACAGAGGGTGCAAGCGTTAATCGGAATTACTGGGCGTAAAGCGCGCGTAGGTGGTTTGTTAAGTTGGATGTGAAATCCCCGGGCTCAACCTGGGAACTGCATTCAAAACTGACTGACTAGAGTATGGTAGAGGGTGGTGGAATTTCCTGTGTAGCGGTGAAATGCGTAGATATAGGAAGGAACACCAGTGGCGAAGGCGACCACCTGGACTGATACTGACACTGAGGTGCGAAAGCGTGGGGAGCAAACAGGATTAGATACCCTGGTAGTCCACGCCGTAAACGATGTCAACTAGCCGTTGGGAGCCTTGAGCTCTTAGTGGCGCAGCTAACGCATTAAGTTGACCGCCTGGGGAGTACGGCCGCAAGGTTAAAACTCAAATGAATTGACGGGGGCCCGCACAAGCGGTGGAGCATGTGGTTTAATTCGAAGCAACGCGAAGAACCTTACCAGGCCTTGACATCCAATGAACTTTCTAGAGATAGATTGGTGCCTTCGGGAACATTGAGACAGGTGCTGCATGGCTGTCGTCAGCTCGTGTCGTGAGATGTTGGGTTAAGTCCCGTAACGAGCGCAACCCTTGTCCTTAGTTACCAGCACGTAATGGTGGGCACTCTAAGGAGACTGCCGGTGACAAACCGGAGGAAGGTGGGGATGACGTCAAGTCATCATGGCCCTTACGGCCTGGGCTACACACGTGCTACAATGGTCGGTACAGAGGGTTGCCAAGCCGCGAGGTGGAGCTAATCCCATAAAACCGATCGTAGTCCGGATCGCAGTCTGCAACTCGACTGCGTGAAGTCGGAATCGCTAGTAATCGCGAATCAGAATGTCGCGGTGAATACGTTCCCGGGCCTTGTACACACCGCCCGTCACACCATGGGAGTGGGTTGCACCAGAAGTAGCTAGTC

>FW305-3-2-15-F-TSA2

TGCAGTCGANCGGTGAAGCCNAGCTTGCTTGGTGGATCAGTGGCGAACGGGTGAGTAACACGTGAGCAACCTGCCCTGGACTCTGGGATAAGCGCTGGAAACGGTGTCTAATACTGGATATGAGCTCTCATCGCATGGTGGGGGTTGGAAAGATTTTTCGGTCTGGGATGGGCTCGCGGCCTATCAGCTTGTTGGTGAGGTAATGGCTCACCAAGGCGTCGACGGGTAGCCGGCCTGAGAGGGTGACCGGCCACACTGGGACTGAGACACGGCCCAGACTCCTACGGGAGGCAGCAGTGGGGAATATTGCACAATGGGCGGAAGCCTGATGCAGCAACGCCGCGTGAGGGATGNCGGCCTTCGGGTTGTAANCCTCTTTTAGCAGGGAAGAAGCGAGAGTGACGGTACCTGCAGAAAAAGCGCCGGCTAACTACGTGCCAGCAGCCGCGGTAATACGTAGGGCGCAAGCGTTATCCGGAATTATTGGGCGTAAAGAGCTCGTAGGCGGTTTGTCGCGTCTGCTGTGAAATCCCGAGGCTCAACCTCGGGCCTGCAGTGGGTACGGGCAGACTAGAGTGCGGTAGGGGAGATTGGAATTCCTGGTGTAGCGGTGGAATGCGCAGATATCAGGAGGAACACCGATGGCGAAGGCAGATCTCTGGGCCGTAACTGACGCTGAGGAGCGAAAGGGTGGGGAGCAAACAGGCTTAGATACCCTGGTAGTCCACCCCGTAAACGTTGGGAACTAGTTGTGGGGACCATTCCACGGTTTCCGTGACGCAGCTAACGCATTAAGTTCCCCGCCTGGGGAGTACGGCCGCAAGGCTAAAACTCAAAGGAATTGACGGGGACCCGCACAAGCGGCGGAGCATGCGGATTAATTCGATGCAACGCGAAGAACCTTACCAAGGCTTGACATACACCAGAACACCGTAGAAATACGGGACTCTTTGGACACTGGTGAACAGGTGGTGCATGGTTGTCGTCAGCTCGTGTCGTGAGATGTTGGGTTAAGTCCCGCAACGAGCGCAACCCTCGTTCTATGTTGCCAGCACGTAATGGTGGGAACTCATGGGATACTGCCGGGGTCAACTCGGAGGAAGGTGGGGATGACGTCAAATCATCATGCCCCTTATGTCTTGGGCTTCACGCATGCTACAATGGCCGGTACAAAGGGCTGCAATACCGTGAGGTGGAGCGAATCCCAAAAAGCCGGTCCCAGTTCGGATTGAGGTCTGCAACTCGACCTCATGAAGTCGGAGTCGCTAGTAATCGCAGATCAGCAACGCTGCGGTGAATACGTTCCCGGGTCTTGTACACACCGCCCGTCAAGTCATGAAAGTCGGTAACACCTGAAGCCGGTGGCCCAACCC

>FW305-3-2-15-F-TSA3

TGCAGTCGAACGGTAACAGGCCGCAAGGTGCTGACGAGTGGCGAACGGGTGAGTAATGCATCGGAACGTGCCCAGTCGTGGGGGATAACGCAGCGAAAGCTGCGCTAATACCGCATACGATCTATGGATGAAAGCGGGGGACCGTAAGGCCTCGCGCGATTGGAGCGGCCGATGTCAGATTAGGTAGTTGGTGGGGTAAAGGCTCACCAAGCCGACGATCTGTAGCTGGTCTGAGAGGACGACCAGCCACACTGGGACTGAGACACGGCCCAGACTCCTACGGGAGGCAGCAGTGGGGAATTTTGGACAATGGGCGCAAGCCTGATCCAGCAATGCCGCGTGCAGGAAGAAGGCCTTCGGGTTGTAAACTGCTTTTGTACGGAACGAAAAGGCTCTGGTTAATACCTGGGGCACATGACGGTACCGTAAGAATAAGCACCGGCTAACTACGTGCCAGCAGCCGCGGTAATACGTAGGGTGCAAGCGTTAATCGGAATTACTGGGCGTAAAGCGTGCGCAGGCGGTTTTGTAAGACAGGCGTGAAATCCCCGGGCTCAACCTGGGAATTGCGCTTGTGACTGCAAGGCTGGAGTGCGGCAGAGGGGGATGGAATTCCGCGTGTAGCAGTGAAATGCGTAGATATGCGGAGGAACACCGATGGCGAAGGCAATCCCCTGGGCCTGCACTGACGCTCATGCACGAAAGCGTGGGGAGCAAACAGGATTAGATACCCTGGTAGTCCACGCCCTAAACGATGTCAACTGGTTGTTGGGTCTCTTCTGACTCAGTAACGAAGCTAACGCGTGAAGTTGACCGCCTGGGGAGTACGGCCGCAAGGTTGAAACTCAAAGGAATTGACGGGGACCCGCACAAGCGGTGGATGATGTGGTTTAATTCGATGCAACGCGAAAAACCTTACCCACCTTTGACATGTACGGAATTTGCCAGAGATGGCTTAGTGCTCGAANAGAGAGCCGTAACACAGGTGCTGCATGGCTGTCGTCAGCTCGTGTCGTGAGATGTTGGGTTAAGTCCCGCAACGAGCGCAACCCTTGTCATTAGTTGCTACGAAAGGGCACTCTAATGAGACTGCCGGTGACAAACCGGAGGAAGGTGGGGATGACGTCAAGTCCTCATGGCCCTTATAGGTGGGGCTACACACGTCATACAATGGCTGGTACAAAGGGTTGCCAACCCGCGAGGGGGAGCTAATCCCATAAAGCCAGTCGTAGTCCGGATCGCAGTCTGCAACTCGACTGCGTGAAGTCGGAATCGCTAGTAATCGTGGATCAGCATGTCACGGTGAATACGTTCCCGGGTCTTGTACACACCGCCCGTCACACCATGGGAGCGGGTCTCGCCAGAAGTAGTTAGCCTAACCGCAAGGAGGGC

>FW305-3-2-15-F-TSA4

GGGAGCAATCCTGGTGGCGAGTGGCGAACGGGTGAGTAATACATCGGAACGTGCCCTGTCGTGGGGGATAACTAGTCGAAAGATTAGCTAATACCGCATACGACCTGAGGGTGAAAGCGGGGGACCGTAAGGCCTCGCGCGATAGGAGCGGCCGATGTCTGATTAGCTAGTTGGTGGGGTAAAGGCCCACCAAGGCGACGATCAGTAGCTGGTCTGAGAGGACGATCAGCCACACTGGGACTGAGACACGGCCCAGACTCCTACGGGAGGCAGCAGTGGGGAATTTTGGACAATGGGGGCAACCCTGATCCAGCAATGCCGCGTGKGTGAAGAAGGCCTTCGGGTTGTAAAGCACTTTTGTCCGGAAAGAAATCCCTTGCTCTAATACAGCGGGGGGATGACGGTACCGGAAGAATAAGCACCGGCTAACTACGTGCCAGCAGCCGCGGTAATACGTAGGGTGCGAGCGTTAATCGGAATTACTGGGCGTAAAGCGTGCGCAGGCGGTTTTGTAAGACAGGCGTGAAATCCCCGAGCTCAACTTGGGAATGGCGCTTGTGACTGCAAGGCTAGAGTATGTCAGAGGGGGGTAGAATTCCACGTGTAGCAGTGAAATGCGTAGAGATGTGGAGGAATACCGATGGCGAAGGCAGCCCCCTGGGACGTCACTGACGCTCATGCACGAAAGCGTGGGGAGCAAACAGGATTAGATACCCTGGTAGTCCACGCCCTAAACGATGTCAACTAGTTGTTGGGGATTCATTTCTTCAGTAACGTAGCTAACGCGTGAAGTTGACCGCCTGGGGAGTACGGTCGCAAGATTAAAACTCAAAGGAATTGACGGGGACCCGCACAAGCGGTGGATGATGTGGATTAATTCGATGCAACGCGAAAAACCTTACCTACCCTTGACATGCCACTAACGAAGCAGAGATGCATTAGGTGCCCGAAAGGGAAAGTGGACACAGGTGCTGCATGGCTGTCGTCAGCTCGTGTCGTGAGATGTTGGGTTAAGTCCCGCAACGAGCGCAACCCTTGTCTTTAGTTGCTACGCAAGAGCACTCTAGAGAGACTGCCGGTGACAAACCGGAGGAAGGTGGGGATGACGTCAAGTCCTCATGGCCCTTATGGGTAGGGCTTCACACGTCATACAATGGTGCGTACAGAGGGTTGCCAACCCGCGAGGGGGAGCTAATCCCAGAAAACGCATCGTAGTCCGGATCGTAGTCTGCAACTCGACTACGTGAAGCTGGAATCGCTAGTAATCGCGGATCAGCATGCCGCGGTGAATACGTTCCCGGGTCTTGTACACACCGCCCGTCACACCATGGGAGTGGGNNTTGCCAGAAGTAGTTAGCCTAACCGCAAGGAGGGCGAT

>FW305-3-2-15-F-TSA5

TGCAGTCGAACGGTGAAGCCNAGCTTGCTTGGTGGATCAGTGGCGAACGGGTGAGTAACACGTGAGCAACCTGCCCTGGACTCTGGGATAAGCGCTGGAAACGGTGTCTAATACTGGATATGAGCTCTCATCGCATGGTGGGGGTTGGAAAGATTTTTCGGTCTGGGATGGGCTCGCGGCCTATCAGCTTGTTGGTGAGGTAATGGCTCACCAAGGCGTCGACGGGTAGCCGGCCTGAGAGGGTGACCGGCCACACTGGGACTGAGACACGGCCCAGACTCCTACGGGAGGCAGCAGTGGGGAATATTGCACAATGGGCGGAAGCCTGATGCAGCAACGCCGCGTGAGGGATGACGGCCTTCGGGTTGTAAACCTCTTTTAGCAGGGAAGAAGCGAGAGTGACGGTACCTGCAGAAAAAGCGCCGGCTAACTACGTGCCAGCAGCCGCGGTAATACGTAGGGCGCAAGCGTTATCCGGAATTATTGGGCGTAAAGAGCTCGTAGGCGGTTTGTCGCGTCTGCTGTGAAATCCCGAGGCTCAACCTCGGGCCTGCAGTGGGTACGGGCAGACTAGAGTGCGGTAGGGGAGATTGGAATTCCTGGTGTAGCGGTGGAATGCGCAGATATCAGGAGGAACACCGATGGCGAAGGCAGATCTCTGGGCCGTAACTGACGCTGAGGAGCGAAAGGGTGGGGAGCAAACAGGCTTAGATACCCTGGTAGTCCACCCCGTAAACGTTGGGAACTAGTTGTGGGGACCATTCCACGGTTTCCGTGACGCAGCTAACGCATTAAGTTCCCCGCCTGGGGAGTACGGCCGCAAGGCTAAAACTCAAAGGAATTGACGGGGACCCGCACAAGCGGCGGAGCATGCGGATTAATTCGATGCAACGCGAAGAACCTTACCAAGGCTTGACATACACCAGAACACCGTAGAAATACGGGACTCTTTGGACACTGGTGAACAGGTGGTGCATGGTTGTCGTCAGCTCGTGTCGTGAGATGTTGGGTTAAGTCCCGCAACGAGCGCAACCCTCGTTCTATGTTGCCAGCACGTAATGGTGGGAACTCATGGGATACTGCCGGGGTCAACTCGGAGGAAGGTGGGGATGACGTCAAATCATCATGCCCCTTATGTCTTGGGCTTCACGCATGCTACAATGGCCGGTACAAAGGGCTGCAATACCGTGAGGTGGAGCGAATCCCAAAAAGCCGGTCCCAGTTCGGATTGAGGTCTGCAACTCGACCTCATGAAGTCGGAGTCGCTAGTAATCGCAGATCAGCAACGCTGCGGTGAATACGTTCCCGGGTCTTGTACACACCGCCCGTCAAGTCATGAAAGTCGGTAACACCTGAAGCCGGTGGCCCAA

>FW305-3-2-15-F-TSA6

TGCAGTCGAACGGTAACAGGCCGCAAGGTGCTGACGAGTGGCGAACGGGTGAGTAATGCATCGGAACGTGCCCAGTCGTGGGGGATAACGCAGCGAAAGCTGCGCTAATACCGCATACGATCTATGGATGAAAGCGGGGGACCGTAAGGCCTCGCGCGATTGGAGCGGCCGATGTCAGATTAGGTAGTTGGTGGGGTAAAGGCTCACCAAGCCGACGATCTGTAGCTGGTCTGAGAGGACGACCAGCCACACTGGGACTGAGACACGGCCCAGACTCCTACGGGAGGCAGCAGTGGGGAATTTTGGACAATGGGCGCAAGCCTGATCCAGCAATGCCGCGTGCAGGAAGAAGGCCTTCGGGTTGTAAACTGCTTTTGTACGGAACGAAAAGGCTCTGGTTAATACCTGGGGCACATGACGGTACCGTAAGAATAAGCACCGGCTAACTACGTGCCAGCAGCCGCGGTAATACGTAGGGNGCAAGCGTTAATCGGAATTACTGGGCGTAAAGCGTGCGCAGGCGGTTTTGTAAGACAGGCGTGNAATCCCCGGGCTCAACCTGGGAATTGCGCTTGTGACTGCAAGGCTGGAGTGCGGCAGAGGGGGATGGAATTCCGCGTGTAGCAGTGAAATGCGTAGATATGCGGAGGAACACCGATGGCGAAGGCAATCCCCTGGGCCTGCACTGACGCTCATGCACGAAAGCGTGGGGAGCAAACAGGATTAGATACCCTGGTAGTCCACGCCCTAAACGATGTCAACTGGTTGTTGGGTCTCTTCTGACTCAGTAACGAAGCTAACGCGTGAAGTTGACCGCCTGGGGAGTACGGCCGCAAGGTTGAAACTCAAAGGAATTGACGGGGACCCGCACAAGCGGTGGATGATGTGGTTTAATTCGATGCAACGCGAAAAACCTTACCCACCTTTGACATGTACGGAATTTGCCAGAGATGGCTTAGTGCTCGAAAGAGAGCCGTAACACAGGTGCTGCATGGCTGTCGTCAGCTCGTGTCGTGAGATGTTGGGTTAAGTCCCGCAACGAGCGCAACCCTTGTCATTAGTTGCTACGAAAGGGCACTCTAATGAGACTGCCGGTGACAAACCGGAGGAAGGTGGGGATGACGTCAAGTCCTCATGGCCCTTATAGGTGGGGCTACMCMCGTCATACAATGGCTGGTACAAAGGGTTGCCAACCCGCGAGGGGGAGCTAATCCCATAAAGCCAGTCGTAGTCCGGATCGCAGTCTGCAACTCGACTGCGTGAAGTCGGAATCGCTAGTAATCGTGGATCAGCATGTCACGGTGAATACGTTCCCGGGTCTTGTACACACCGCCCGTCACACCATGGGAGCGGGTCTCGCCAGAAGTAGTTA

>FW305-3-2-15-F-TSA7

TGCAGTCGGACGAAGGCTTCGGCCTTAGTGGCGCACGGGTGCGTAACGCGTGGGAATCTACCCTTGGGTTCGGAATAACAGTTAGAAATGACTGCTAATACCGGATGATGACGTAAGTCCAAAGATTTATCGCCCAAGGATGAGCCCGCGTAGGATTAGCTAGTTGGTGAGGTAAAGGCTCACCAAGGCGACGATCCTTAGCTGGTCTGAGAGGATGATCAGCCACACTGGGACTGAGACACGGCCCAGACTCCTACGGGAGGCAGCAGTGGGGAATATTGGACAATGGGCGAAAGCCTGATCCAGCAATGCCGCGTGAGTGATGAAGGCCTTAGGGTTGTAAAGCTCTTTTACCCGGGATGATAATGACAGTACCGGGAGAATAAGCTCCGGCTAACTCCGTGCCAGCAGCCGCGGTAATACGGAGGGAGCTAGCGTTGTTCGGAATTACTGGGCGTAAAGCGCACGTAGGCGGCTTTGTAAGTTAGAGGTGAAAGCCTGGAGCTTAACTCCAGAACTGCCTTTAAGACTGCATCGCTTGAATCCAGGAGAGGTGAGTGGAATTCCGAGTGTAGAGGTGAAATTCGTAGATATTCGGAAGAACACCAGTGGCGAAGGCGGCTCACTGGACTGGTATTGACGCTGAGGTGCGAAAGCGTGGGGAGCAAACAGGATTAGATACCCTGGTAGTCCACGCCGTAAACGATGATAACTAGCTGTCTGGGCTCATGGAGCTTAGGTGGCGCAGCTAACGCATTAAGTTATCCGCCTGGGGAGTACGGTCGCAAGATTAAAACTCAAAGGAATTGACGGGGGCCTGCACAAGCGGTGGAGCATGTGGTTTAATTCGAAGCAACGCGCAGAACCTTACCAGCGTTTGACATCCTCATCGCGGATTCCAGAGATGGATTCCTTCAGTTCGGCTGGATGAGTGACAGGTGCTGCATGGCTGTCGTCAGCTCGTGTCGTGAGATGTTGGGTTAAGTCCCGCAACGAGCGCAACCCTCGTCCTTAGTTGCCATCATTTAGTTGGGCACTCTAAGGAAACCGCCGGTGATAAGCCGGAGGAAGGTGGGGATGACGTCAAGTCCTCATGGCCCTTACGCGCTGGGCTACACACGTGCTACAATGGCAACTACAGTGGGCAGCAAACCTGCGAGGGTGAGCTAATCTCCAAAAGTTGTCTCAGTTCGGATTGTTCTCTGCAACTCGAGAGCATGAAGGCGGAATCGCTAGTAATCGCGGATCAGCATGCCGCGGTGAATACGTTCCCAGGCCTTGTACANNNCGCCCGTCACACCATGGGAGTTGGATTCACCCGAAGGCGCTGCGCTAA

>DP16D-L5

GGTAACAGGTCTTCGGACGCTGACGAGTGGCGAACGGGTGAGTAATACATCGGAACGTGCCCAGTCGTGGGGGATAACTACTCGAAAGAGTAGCTAATACCGCATACGATCTGAGGATGAAAGCGGGGGACCTTCGGGCCTCGCGCGATTGGAGCGGCCGATGGCAGATTAGGTAGTTGGTGGGATAAAAGCTTACCAAGCCGACGATCTGTAGCTGGTCTGAGAGGACGACCAGCCACACTGGGACTGAGACACGGCCCAGACTCCTACGGGAGGCAGCAGTGGGGAATTTTGGACAATGGGCGAAAGCCTGATCCAGCAATGCCGCGTGCAGGATGAAGGCCTTCGGGTTGTAAACTGCTTTTGTACGGAACGAAAAAGCTCCTTCTAATACAGGGGGCCCATGACGGTACCGTAAGAATAAGCACCGGCTAACTACGTGCCAGCAGCCGCGGTAATACGTAGGGTGCGAGCGTTAATCGGAATTACTGGGCGTAAAGCGTGCGCAGGCGGTTATGTAAGACAGATGTGAAATCCCCGGGCTCAACCTGGGAACTGCATTTGTGACTGCATGGCTAGAGTACGGTAGAGGGGGATGGAATTCCGCGTGTAGCAGTGAAATGCGTAGATATGCGGAGGAACACCGATGGCGAAGGCAATCCCCTGGACCTGTACTGACGCTCATGCACGAAAGCGTGGGGAGCAAACAGGATTAGATACCCTGGTAGTCCACGCCCTAAACGATGTCAACTGGTTGTTGGGAATTAGTTTTCTCAGTAACGAAGCTAACGCGTGAAGTTGACCGCCTGGGGAGTACGGCCGCAAGGTTGAAACTCAAAGGAATTGACGGGGACCCGCACAAGCGGTGGATGATGTGGTTTAATTCGATGCAACGCGAAAAACCTTACCCACCTTTGACATGGCAGGAAGTTTCCAGAGATGGATTCGTGCTCGAAAGAGAACCTGCACACAGGTGCTGCATGGCTGTCGTCAGCTCGTGTCGTGAGATGTTGGGTTAAGTCCCGCAACGAGCGCAACC

>FW602-T3

TGGCTCCTTACGGTTACCCCACCGACTTCGGGTGTTACAAACTCTCGTGGTGTGACGGGCGGTGTGTACAAGGCCCGGGAACGTATTCACCGCGGCATGCTGATCCGCGATTACTAGCGATTCCAGCTTCATGTAGGCGAGTTGCAGCCTACAATCCGAACTGAGAGTGGTTTTATGGGATTGGCTCGACCTCGCGGTTTTGCTGCCCTTTGTACCACCCATTGTAGCACGTGTGTAGCCCAGGTCATAAGGGGCATGATGATTTGACGTCATCCCCACCTTCCTCCGGTTTGTCACCGGCAGTCACCTTAGAGTGCCCAACTGAATGCTGGCAACTAAGATCAAGGGTTGCGCTCGTTGCGGGACTTAACCCAACATCTCACGACACGAGCTGACGACAACCATGCACCACCTGTCACTCTGTCCCCCGAAGGGGAACGCCCTATCTCTAGGGTTGTCAGAGGATGTCAAGACCTGGTAAGGTTCTTCGCGTTGCTTCGAATTAAACCACATGCTCCACCGCTTGTGCGGGCCCCCGTCAATTCCTTTGAGTTTCAGCCTTGCGGCCGTACTCCCCAGGCGGAGTGCTTAATGCGTTTGCTGCAGCACTAAAGGGCGGAAACCCTCTAACACTTATCACTCATCGTTTACGGCGTGGACTACCAGGGTATCTAATCCTGTTTGCTCCCCACGCTTTCGCGCCTCAGTGTCAGTTACAGGCCAAAGAGTCGCCTTCGCCACTGGTGTTCCTCCACATCTCTACGCATTTCACCGCTACACGTGGAATTCCACTCTTCTCTCCTGCACTCAAGTCTCCCAGTTTCCAATGACCCTCCCCGGTTGAGCCGGGGGCTTTCACATCAGACTTAAGAGACCACCTGCGCGCGCTTTACGCCCAATAATTCCGGACAACGCTTGCCACCTACGTATTACCGCGGCTGCTGGCACGTATTTAGCCG

>DP16D-E2

AGTCGAACGGCAGCACGGGAGCAATCCTGGTGGCGAGTGGCGAACGGGTGAGTAATACATCGGAACGTGCCCTGTCGTGGGGGATAACTAGTCGAAAGATTAGCTAATACCGCATACGACCTGAGGGTGAAAGCGGGGGACCGTAAGGCCTCGCGCGATAGGAGCGGCCGATGTCTGATTAGCTAGTTGGTGGGGTAAAGGCCCACCAAGGCGACGATCAGTAGCTGGTCTGAGAGGACGATCAGCCACACTGGGACTGAGACACGGCCCAGACTCCTACGGGAGGCAGCAGTGGGGAATTTTGGACAATGGGGGCAACCCTGATCCAGCAATGCCGCGTGTGTGAAGAAGGCCTTCGGGTTGTAAAGCACTTTTGTCCGGAAAGAAATCCCTTGCCCTAATACGGCGGGGGGATGACGGTACCGGAAGAATAAGCACCGGCTAACTACGTGCCAGCAGCCGCGGTAATACGTAGGGTGCGAGCGTTAATCGGAATTACTGGGCGTAAAGCGTGCGCAGGCGGTTTTGTAAGACAGGCGTGAAATCCCCGAGCTCAACTTGGGAATGGCGCTTGTGACTGCAAGGCTAGAGTATGTCAGAGGGGGGTAGAATTCCACGTGTAGCAGTGAAATGCGTAGAGATGTGGAGGAATACCGATGGCGAAGGCAGCCCCCTGGGACGTCACTGACGCTCATGCACGAAAGCGTGGGGAGCAAACAGGATTAGATACCCTGGTAGTCCACGCCCTAAACGATGTCAACTAGTTGTTGGGGATTCATTTCTTCAGTAACGTAGCTAACGCGTGAAGTTGACCGCCTGGGGAGTACGGTCGCAAGATTAAAACTCAAAGGAATTGACGGGGACCCGCACAAGCGGTGGATGATGTGGATTAATTCGATGCAACGCGNAAAACCTTACCTACCCTTGACATGCCACTAACGAAGCAGAGATGCATCAGGTGCCCGAAAGGGAAAGTGGACACAGGTGCTGCATGGCTGTCGTCAGCTCGTGTCGTGAGATGTTGGGTTAAGTCCCGCAACGAGCGCAACCCTTGTCTTTAGTTGCTACGCAAGAGCACTCTAGAGAGACTGCCGGTGACAAACCGGAGGAAGGTGGGGATGACGTCAAGTCCTCATGGCCCTTATGGGTAGGGCTTCACACGTCATACAATGGTGCGTACAGAGGGTTGCCAACCCGCGAGGGGGAGCTAATCCCAGAAAACGCATCGTAGTCCGGATCGTAGTCTGCAACTCGACTACGTGAAGCTGGAATCGCTAGTAATCGCGGATCAGCATGCCGCGGTGAATACGTTCCCGGGTCTTGTACACACCGCCCGTCACACCATGGGAGTGGGTTTTGCCAGAAGTAGTTAGCCTAACCGCAAGGAGGGCGATACC

>DP16D-E3

CGGCAGCACGGAGCTTGCTCTGGTGGCGAGTGGCGAACGGGTGAGTAATATATCGGAACGTACCCTGGAGTGGGGGATAACGTAGCGAAAGTTACGCTAATACCGCATACGATCTAAGGATGAAAGTGGGGGATCGCAAGACCTCATGCTCGTGGAGCGGCCGATATCTGATTAGCTAGTTGGTAGGGTAAAAGCCTACCAAGGCATCGATCAGTAGCTGGTCTGAGAGGACGACCAGCCACACTGGAACTGAGACACGGTCCAGACTCCTACGGGAGGCAGCAGTGGGGAATTTTGGACAATGGGCGCAAGCCTGATCCAGCAATGCCGCGTGAGTGAAGAAGGCCTTCGGGTTGTAAAGCTCTTTTGTCAGGGAAGAAACGGTGGGAGCTAATATCTCCTGCTAATGACGGTACCTGAAGAATAAGCACCGGCTAACTACGTGCCAGCAGCCGCGGTAATACGTAGGGTGCAAGCGTTAATCGGAATTACTGGGCGTAAAGCGTGCGCAGGCGGTTTTGTAAGTCTGATGTGAAATCCCCGGGCTCAACCTGGGAATTGCATTGGAGACTGCAAGGCTAGAATCTGGCAGAGGGGGGTAGAATTCCACGTGTAGCAGTGAAATGCGTAGATATGTGGAGGAACACCGATGGCGAAGGCAGCCCCCTGGGTCAAGATTGACGCTCATGCACGAAAGCGTGGGGAGCAAACAGGATTAGATACCCTGGTAGTCCACGCCCTAAACGATGTCTACTAGTTGTCGGGTCTTAATTGACTTGGTAACGCAGCTAACGCGTGAAGTAGACCGCCTGGGGAGTACGGTCGCAAGATTAAAACTCAAAGGAATTGACGGGGACCCGCACAAGCGGTGGATGATGTGGATTAATTCGATGCAACGCGAAAAACCTTACCTACCCTTGACATGGCTGGAATCCCCGAGAGATTGGGGAGTGCTCGAAAGAGAACCAGTACACAGGTGCTGCATGGCTGTCGTCAGCTCGTGTCGTGAGATGTTGGGTTAAGTCCCGCAACGAGCGCAACCCTTGTCATTAGTTGCTACGAAAGGGCACTCTAATGAGACTGCCGGTGACAAACCGGAGGAAGGTGGGGATGACGTCAAGTCCTCATGGCCCTTATGGGTAGGGCTTCACACGTCATACAATGGTACATACAGAGCGCCGCCAACCCGCGAGGGGGAGCTAATCGCAGAAAGTGTATCGTAGTCCGGATTGTAGTCTGCAACTCGACTGCATGAAGTTGGAATCGCTAGTAATCGCGGATCAGCATGTCGCGGTGAATACGTTCCCGGGTCTTGTACACACCGCCCGTCACACCATGGGAGCGGGTTTTACCAGAAGTAGGTAGCTTAACCG

>DP16D-R1

AGTCGAGCGGATGAAGAGAGCTTGCTCTCTGATTCAGCGGCGGACGGGTGAGTAATGCCTAGGAATCTGCCTGGTAGTGGGGGACAACGTCTCGAAAGGGACGCTAATACCGCATACGTCCTACGGGAGAAAGCAGGGGACCTTCGGGCCTTGCGCTATCAGATGAGCCTAGGTCGGATTAGCTAGTTGGTGAGGTAATGGCTCACCAAGGCGACGATCCGTAACTGGTCTGAGAGGATGATCAGTCACACTGGAACTGAGACACGGTCCAGACTCCTACGGGAGGCAGCAGTGGGGAATATTGGACAATGGGCGAAAGCCTGATCCAGCCATGCCGCGTGTGTGAAGAAGGTNTTCGGATTGTAAAGCACTTTAAGTTGGGAGGAAGGGTTGTAGATTAATACTCTGCAATTTTGACGTTACCGACAGAATAAGCACCGGCTAACTCTGTGCCAGCAGCCGCGGTAATACAGAGGGTGCAAGCGTTAATCGGAATTACTGGGCGTAAAGCGCGCGTAGGTGGTTTGTTAAGTTGGATGTGAAAGCCCCGGGCTCAACCTGGGAACTGCATTCAAAACTGACAAGCTAGAGTATGGTAGAGGGTGGTGGAATTTCCTGTGTAGCGGTGAAATGCGTAGATATAGGAAGGAACACCAGTGGCGAAGGCGACCACCTGGACTGATACTGACACTGAGGTGCGAAAGCGTGGGGAGCAAACAGGATTAGATACCCTGGTAGTCCACGCCGTAAACGATGTCAACTAGCCGTTGGGAGCCTTGAGCTCTTAGTGGCGCAGCTAACGCATTAAGTTGACCGCCTGGGGAGTACGGCCGCAAGGTTAAAACTCAAATGAATTGACGGGGGCCCGCACAAGCGGTGGAGCATGTGGTTTAATTCGAAGCAACGCGAAGAACCTTACCAGGCCTTGACATCCAATGAACTTTCCAGAGATGGATTGGTGCCTTCGGGAACATTGAGACAGGTGCTGCATGGCTGTCGTCAGCTCGTGTCGTGAGATGTTGGGTTAAGTCCCGTAACGAGCGCAACCCTTGTCCTTAGTTACCAGCACGTTATGGTGGGCACTCTAAGGAGACTGCCGGTGACAAACCGGAGGAAGGTGGGGATGACGTCAAGTCATCATGGCCCTTACGGCCTGGGCTACACACGTGCTACAATGGTCGGTACAGAGGGTTGCCAAGCCGCGAGGTGGAGCTAATCCCATAAAACCGATCGTAGTCCGGATCGCAGTCTGCAACTCGACTGCGTGAAGTCGGAATCGCTAGTAATCGCGAATCAGAATGTCGCGGTGAATACGTTCCCGGGCCTTGTACACACCGCCCGTCACACCATGGGAGTGGGTTGCACCAGAAGTAGCTAGTCTAACCTTC

>DP16D-R6

CGGTAACAGGTCTTCGGATGCTGACGAGTGGCGAACGGGTGAGTAATACATCGGAACGTGCCCGATCGTGGGGGATAACGGAGCGAAAGCTTTGCTAATACCGCATACGATCTACGGATGAAAGCAGGGGACCGCAAGGCCTTGCGCGGACGGAGCGGCCGATGGCAGATTAGGTAGTTGGTGGGATAAAAGCTTACCAAGCCGACGATCTGTAGCTGGTCTGAGAGGACGACCAGCCACACTGGGACTGAGACACGGCCCAGACTCCTACGGGAGGCAGCAGTGGGGAATTTTGGACAATGGGCGAAAGCCTGATCCAGCCATGCCGCGTGCAGGATGAAGGCCTTCGGGTTGTAAACTGCTTTTGTACGGAACGAAAAGACTCTTTCTAATAAAGAGGGTCCATGACGGTACCGTAAGAATAAGCACCGGCTAACTACGTGCCAGCAGCCGCGGTAATACGTAGGGTGCGAGCGTTAATCGGAATTACTGGGCGTAAAGCGTGCGCAGGCGGTTATGCAAGACAGATGTGAAATCCCCGGGCTCAACCTGGGAACTGCATTTGTGACTGCATAGCTAGAGTACGGTAGAGGGGGATGGAATTCCGCGTGTAGCAGTGAAATGCGTAGATATGCGGAGGAACACCGATGGCGAAGGCAATCCCCTGGACCTGTACTGACGCTCATGCACGAAAGCGTGGGGAGCAAACAGGATTAGATACCCTGGTAGTCCACGCCCTAAACGATGTCAACTGGTTGTTGGGTCTTCACTGACTCAGTAACGAAGCTAACGCGTGAAGTTGACCGCCTGGGGAGTACGGCCGCAAGGTTGAAACTCAAAGGAATTGACGGGGACCCGCACAAGCGGTGGATGATGTGGTTTAATTCGATGCAACGCGNAAAACCTTACCCACCTTTGACATGTACGGAATTTGCCAGAGATGGCTTAGTGCTCGAAAGAGAACCGTAACACAGGTGCTGCATGGCTGTCGTCAGCTCGTGTCGTGAGATGTTGGGTTAAGTCCCGCAACGAGCGCAACCCTTGCCATTAGTTGCTACGAAAGGGCACTCTAATGGGACTGCCGGTGACAAACCGGAGGAAGGTGGGGATGACGTCAAGTCCTCATGGCCCTTATAGGTGGGGCTACACACGTCATACAATGGCTGGTACAGAGGGTTGCCAACCCGCGAGGGGGAGCCAATCCCATAAAGCCAGTCGTAGTCCGGATCGCAGTCTGCAACTCGACTGCGTGAAGTCGGAATCGCTAGTAATCGCGGATCAGAATGTCGCGGTGAATACGTTCCCGGGTCTTGTACACACCGCCCGTCACACCATGGGAGCGGGTTCTGCCAGAAGTAGTTAGCCTAACCGCAAGGAGGGCGATAC

>DP16D-T1

GCTACACATGCAGTCGAGCGGATGAGAAGAGCTTGCTCTTCGATTCAGCGGCGGACGGGTGAGTAATGCCTAGGAATCTGCCTGGTAGTGGGGGACAACGTTTCGAAAGGAACGCTAATACCGCATACGTCCTACGGGAGAAAGCAGGGGACCTTCGGGCCTTGCGCTATCAGATGAGCCTAGGTCGGATTAGCTAGTTGGTGAGGTAATGGCTCACCAAGGCGACGATCCGTAACTGGTCTGAGAGGATGATCAGTCACACTGGAACTGAGACACGGTCCAGANTYCTACGGGAGGCAGCAGTGGGGAATATTGGACAATGGGCGAAAGCCTGATCCAGCCATGCCGCGTGTGTGAAGAAGGTCTTCGGATTGTAAAGCACTTTAAGTTGGGAGGAAGGGCATTAACCTAATACGTTAGTGTTTTGACGTTACCGACAGAATAAGCACCGGCTAACTCTGTGCCAGCAGCCGCGGTAATACAGAGGGTGCAAGCGTTAATCGGAATTACTGGGCGTAAAGCGCGCGTAGGTGGTTTGTTAAGTTGGATGTGAAAGCCCCGGGCTCAACCTGGGAACTGCATCCAAAACTGGCAAGCTAGAGTACGGTAGAGGGTGGTGGAATTTCCTGTGTAGCGGTGAAATGCGTAGATATAGGAAGGAACACCAGTGGCGAAGGCGACCACCTGGACTGATACTGACACTGAGGTGCGAAAGCGTGGGGAGCAAACAGGATTAGATACCCTGGTAGTCCACGCCGTAAACGATGTCAACTAGCCGTTGGAATCCTTGAGATTTTAGTGGCGCAGCTAACGCATTAAGTTGACCGCCTGGGGAGTACGGCCGCAAGGTTAAAACTCAAATGAATTGACGGGGGCCCGCACAAGCGGTGGAGCATGTGGTTTAATTCGAAGCAACGCGAAGAACCTTACCAGGCCTTGACATGCAGAGAACTTTCCAGAGATGGATTGGTGCCTTCGGGAACTCTGACACAGGTGCTGCATGGCTGTCGTCAGCTCGTGTCGTGAGATGTTGGGTTAAGTCCCGTAACGAGCGCAACCCTTGTCCTTAGTTACCAGCACGTTATGGTGGGCACTCTAAGGAGACTGCCGGTGACAAACCGGAGGAAGGTGGGGATGACGTCAAGTCATCATGGCCCTTACGGCCTGGGCTACACACGTGCTACAATGGTCGGTACAGAGGGTTGCCAAGCCGCGAGGTGGAGCTAATCTCACAAAACCGATCGTAGTCCGGATCGCAGTCTGCAACTCGACTGCGTGAAGTCGGAATCGCTAGTAATCGCGAATCAGAATGTCGCGGTGAATACGTTCCCGGGCCTTGTACACACCGCCCGTCACACCATGGGAGTGGGTTGCACCAGAAGTAGCTAGTCTAACCT

>FW215-E1

CTACACATGCAGTCGAGCGGATGAAGGAGCTTGCTCCTGGATTCAGCGGCGGACGGGTGAGTAATGCCTAGGAATCTGCCTGGTAGTGGGGGACAACGTTTCGAAAGGAACGCTAATACCGCATACGTCCTACGGGAGAAAGCAGGGGACCTTCGGGCCTTGCGCTATCAGATGAGCCTAGGTCGGATTAGCTAGTTGGTGAGGTAATGGCTCACCAAGGCGACGATCCGTAACTGGTCTGAGAGGATGATCAGTCACACTGGAACTGAGACACGGTCCAGACTCCTACGGGAGGCAGCAGTGGGGAATATTGGACAATGGGCGAAAGCCTGATCCAGCCATGCCGCGTGTGTGAAGAAGGTCTTCGGATTGTAAAGCACTTTAAGTTGGGAGGAAGGGTTGTAGATTAATACTCTGCAATTTTGACGTTACCGACAGAATAAGCACCGGCTAACTCTGTGCCAGCAGCCGCGGTAATACAGAGGGTGCAAGCGTTAATCGGAATTACTGGGCGTAAAGCGCGCGTAGGTGGTTCGTTAAGTTGGATGTGAAATCCCCGGGCTCAACCTGGGAACTGCATCCAAAACTGGCGAGCTAGAGTATGGTAGAGGGTGGTGGAATTTCCTGTGTAGCGGTGAAATGCGTAGATATAGGAAGGAACACCAGTGGCGAAGGCGACCACCTGGACTGATACTGACACTGAGGTGCGAAAGCGTGGGGAGCAAACAGGATTAGATACCCTGGTAGTCCACGCCGTAAACGATGTCAACTAGCCGTTGGGAGCCTTGAGCTCTTAGTGGCGCAGCTAACGCATTAAGTTGACCGCCTGGGGAGTACGGCCGCAAGGTTAAAACTCAAATGAATTGACGGGGGCCCGCACAAGCGGTGGAGCATGTGGTTTAATTCGAAGCAACGCGAAGAACCTTACCAGGCCTTGACATCCAATGAACTTTCCAGAGATGGATTGGTGCCTTCGGGAACATTGAGACAGGTGCTGCATGGCTGTCGTCAGCTCGTGTCGTGAGATGTTGGGTTAAGTCCCGTAACGAGCGCAACCCTTGTCCTTAGTTACCAGCACGTAATGGTGGGCACTCTAAGGAGACTGCCGGTGACAAACCGGARGAAGGTGGGGATGACGTCAAGTCATCATGGCCCTTACGGCCTGGGCTACACACGTGCTACAATGGTCGGTACAAAGGGTTGCCAAGCCGCGAGGTGGAGCTAATCCCATAAAACCGATCGTAGTCCGGATCGCAGTCTGCAACTCGACTGCGTGAAGTCGGAATCGCTAGTAATCGCGAATCAGAATGTCGCGGTGAATACGTTCCCGGGCCTTGTACACACCGCCCGTCACACCATGGGAGTGGGTTGCACCAGAAGTAGCTAGTCTAACCTTCGGGGGGAC

>FW215-L1

TGCAGTCGAGCGGATGAAAGGAGCTTGCTCCTGGATTCAGCGGCGGACGGGTGAGTAATGCCTAGGAATCTGCCTGGTAGTGGGGGACAACGTTTCGAAAGGAACGCTAATACCGCATACGTCCTACGGGAGAAAGCAGGGGACCTTCGGGCCTTGCGCTATCAGATGAGCCTAGGTCGGATTAGCTAGTTGGTGAGGTAATGGCTCACCAAGGCGACGATCCGTAACTGGTCTGAGAGGATGATCAGTCACACTGGAACTGAGACACGGTCCAGACTCCTACGGGAGGCAGCAGTGGGGAATATTGGACAATGGGCGAAAGCCTGATCCAGCCATGCCGCGTGTGTGAAGAAGGTCTTCGGATTGTAAAGCACTTTAAGTTGGGAGGAAGGGTTGTAGATTAATACTCTGCAATTTTGACGTTACCGACAGAATAAGCACCGGCTAACTCTGTGCCAGCAGCCGCGGTAATACAGAGGGTGCAAGCGTTAATCGGAATTACTGGGCGTAAAGCGCGCGTAGGTGGTTCGTTAAGTTGGATGTGAAATCCCCGGGCTCAACCTGGGAACTGCATCCAAAACTGGCGAGCTAGAGTATGGTAGAGGGTGGTGGAATTTCCTGTGTAGCGGTGAAATGCGTAGATATAGGAAGGAACACCAGTGGCGAAGGCGACCACCTGGACTGATACTGACACTGAGGTGCGAAAGCGTGGGGAGCAAACAGGATTAGATACCCTGGTAGTCCACGCCGTAAACGATGTCAACTAGCCGTTGGGAGCCTTGAGCTCTTAGTGGCGCAGCTAACGCATTAAGTTGACCGCCTGGGGAGTACGGCCGCAAGGTTAAAACTCAAATGAATTGACGGGGGCCCGCACAAGCGGTGGAGCATGTGGTTTAATTCGAAGCAACGCGAAGAACCTTACCAGGCCTTGACATCCAATGAACTTTCCAGAGATGGATTGGTGCCTTCGGGAACATTGAGACAGGTGCTGCATGGCTGTCGTCAGCTCGTGTCGTGAGATGTTGGGTTAAGTCCCGTAACGAGCGCAACCCTTGTCCTTAGTTACCAGCACGTAATGGTGGGCACTCTAAGGAGACTGCCGGTGACAAACCGGAGGAAGGTGGGGATGACGTCAAGTCATCATGGCCCTTACGGCCTGGGCTACACACGTGCTACAATGGTCGGTACAAAGGGTTGCCAAGCCGCGAGGTGGAGCTAATCCCATAAAACCGATCGTAGTCCGGATCGCAGTCTGCAACTCGACTGCGTGAAGTCGGAATCGCTAGTAATCGCGAATCAGAATGTCGCGGTGAATACGTTCCCGGGCCTTGTACACACCGCCCGTCACACCATGGGAGTGGGTTGCACCAGAAGTAGCTAGTCTAACCTTCGGGGGGACGGT

>FW215-L2

GCTACACATGCAGTCGAGCGGATGACAGGAGCTTGCTCCTGAATTCAGCGGCGGACGGGTGAGTAATGCCTAGGAATCTGCCTGGTAGTGGGGGACAACGTTTCGAAAGGAACGCTAATACCGCATACGTCCTACGGGAGAAAGCAGGGGACCTTCGGGCCTTGCGCTATCAGATGAGCCTAGGTCGGATTAGCTAGTTGGTGAGGTAATGGCTCACCAAGGCGACGATCCGTAACTGGTCTGAGAGGATGATCAGTCACACTGGAACTGAGACACGGTCCAGAYTCCTACGGGAGGCAGCAGTGGGGAATATTGGACAATGGGCGAAAGCCTGATCCAGCCATGCCGCGTGTGTGAAGAAGGTCTTCGGATTGTAAAGCACTTTAAGTTGGGAGGAAGGGCATTAACCTAATACGTTAGTGTTTTGACGTTACCGACAGAATAAGCACCGGCTAACTCTGTGCCAGCAGCCGCGGTAATACAGAGGGTGCAAGCGTTAATCGGAATTACTGGGCGTAAAGCGCGCGTAGGTGGTTTGTTAAGTTGGATGTGAAAGCCCCGGGCTCAACCTGGGAACTGCATTCAAAACTGACAAGCTAGAGTATGGTAGAGGGTGGTGGAATTTCCTGTGTAGCGGTGAAATGCGTAGATATAGGAAGGAACACCAGTGGCGAAGGCGACCACCTGGACTGATACTGACACTGAGGTGCGAAAGCGTGGGGAGCAAACAGGATTAGATACCCTGGTAGTCCACGCCGTAAACGATGTCAACTAGCCGTTGGGAGCCTTGAGCTCTTAGTGGCGCAGCTAACGCATTAAGTTGACCGCCTGGGGAGTACGGCCGCAAGGTTAAAACTCAAATGAATTGACGGGGGCCCGCACAAGCGGTGGAGCATGTGGTTTAATTCGAAGCAACGCGAAGAACCTTACCAGGCCTTGACATCCAATGAACTTTCCAGAGATGGATTGGTGCCTTCGGGAACATTGAGACAGGTGCTGCATGGCTGTCGTCAGCTCGTGTCGTGAGATGTTGGGTTAAGTCCCGTAACGAGCGCAACCCTTGTCCTTAGTTACCAGCACGTCATGGTGGGCACTCTAAGGAGACTGCCGGTGACAAACCGGAGGAAGGTGGGGATGACGTCAAGTCATCATGGCCCTTACGGCCTGGGCTACACACGTGCTACAATGGTCGGTACAGAGGGTTGCCAAGCCGCGAGGTGGAGCTAATCCCACAAAACCGATCGTAGTCCGGATCGCAGTCTGCAACTCGACTGCGTGAAGTCGGAATCGCTAGTAATCGCGAATCAGAATGTCGCGGTGAATACGTTCCCGGGCCTTGTACACACCGCCCGTCACACCATGGGAGTGGGTTGCACCAGAAGTAGCTAGTCT

>FW215-R2

AGGAGCTTGCTCCTGGATTCAGCGGCGGACGGGTGAGTAATGCCTAGGAATCTGCCTGGTAGTGGGGGACAACGTTTCGAAAGGAACGCTAATACCGCATACGTCCTACGGGAGAAAGCAGGGGACCTTCGGGCCTTGCGCTATCAGATGAGCCTAGGTCGGATTAGCTAGTTGGTGAGGTAATGGCTCACCAAGGCGACGATCCGTAACTGGTCTGAGAGGATGATCAGTCACACTGGAACTGAGACACGGTCCAGACTCCTACGGGAGGCAGNCAGTGGGGAATATTGGACAATGGGCGAAAGCCTGATCCAGCCATGCCGCGTGTGTGAAGAAGGTCTTCGGATTGTAAAGCACTTTAAGTTGGGAGGAAGGGTTGTAGATTAATACTCTGCAATTTTGACGTTACCGACAGAATAAGCACCGGCTAACTCTGTGCCAGCAGCCGCGGTAATACAGAGGGTGCAAGCGTTAATCGGAATTACTGGGCGTAAAGCGCGCGTAGGTGGTTCGTTAAGTTGGATGTGAAATCCCCGGGCTCAACCTGGGAACTGCATCCAAAACTGGCGAGCTAGAGTATGGTAGAGGGTGGTGGAATTTCCTGTGTAGCGGTGAAATGCGTAGATATAGGAAGGAACACCAGTGGCGAAGGCGACCACCTGGACTGATACTGACACTGAGGTGCGAAAGCGTGGGGAGCAAACAGGATTAGATACCCTGGTAGTCCACGCCGTAAACGATGTCAACTAGCCGTTGGGAGCCTTGAGCTCTTAGTGGCGCAGCTAACGCATTAAGTTGACCGCCTGGGGAGTACGGCCGCAAGGTTAAAACTCAAATGAATTGACGGGGGCCCGCACAAGCGGTGGAGCATGTGGTTTAATTCGAAGCAACGCGAAGAACCTTACCAGGCCTTGACATCCAATGAACTTTCCAGAGATGGATTGGTGCCTTCGGGAACATTGAGACAGGTGCTGCATGGCTGTCGTCAGCTCGTGTCGTGAGATGTTGGGTTAAGTCCCGTAACGAGCGCAACCCTTGTCCTTAGTTACCAGCACGTAATGGTGGGCACTCTAAGGAGACTGCCGGTGACAAACCGGAGGAAGGTGGGGATGACGTCAAGTCATCATGGCCCTTACGGCCTGGGCTACACACGTGCTACAATGGTCGGTACAAAGGGTTGCCAAGCCGCGAGGTGGAGCTAATCCCATAAAACCGATCGTAGTCCGGATCGCAGTCTGCAACTCGACTGCGTGAAGTCGGAATCGCTAGTAATCGCGAATCAGAATGTCGCGGTGAATACGTTCCCGGGCCTTGTACACACCGCCCGTCACACCATGGGAGTGGGTTGCACCAGAAGTAGCTAGTCTAACCTTCGGG

>FW215-R3

GCTACACATGCAGTCGAGCGGATGACAGGAGCTTGCTCCTGAATTCAGCGGCGGACGGGTGAGTAATGCCTAGGAATCTGCCTGGTAGTGGGGGACAACGTTTCGAAAGGAACGCTAATACCGCATACGTCCTACGGGAGAAAGCAGGGGACCTTCGGGCCTTGCGCTATCAGATGAGCCTAGGTCGGATTAGCTAGTTGGTGAGGTAATGGCTCACCAAGGCGACGATCCGTAACTGGTCTGAGAGGATGATCAGTCACACTGGAACTGAGACACGGTCCAGACTCCTACGGGAGGCAGCAGTGGGGAATATTGGACAATGGGCGAAAGCCTGATCCAGCCATGCCGCGTGTGTGAAGAAGGTCTTCGGATTGTAAAGCACTTTAAGTTGGGAGGAAGGGCATTAACCTAATACGTTAGTGTTTTGACGTTACCGACAGAATAAGCACCGGCTAACTCTGTGCCAGCAGCCGCGGTAATACAGAGGGTGCAAGCGTTAATCGGAATTACTGGGCGTAAAGCGCGCGTAGGTGGTTTGTTAAGTTGGATGTGAAAGCCCCGGGCTCAACCTGGGAACTGCATTCAAAACTGACAAGCTAGAGTATGGTAGAGGGTGGTGGAATTTCCTGTGTAGCGGTGAAATGCGTAGATATAGGAAGGAACACCAGTGGCGAAGGCGACCACCTGGACTGATACTGACACTGAGGTGCGAAAGCGTGGGGAGCAAACAGGATTAGATACCCTGGTAGTCCACGCCGTAAACGATGTCAACTAGCCGTTGGGAGCCTTGAGCTCTTAGTGGCGCAGCTAACGCATTAAGTTGACCGCCTGGGGAGTACGGCCGCAAGGTTAAAACTCAAATGAATTGACGGGGGCCCGCACAAGCGGTGGAGCATGTGGTTTAATTCGAAGCAACGCGAAGAACCTTACCAGGCCTTGACATCCAATGAACTTTCCAGAGATGGATTGGTGCCTTCGGGAACATTGAGACAGGTGCTGCATGGCTGTCGTCAGCTCGTGTCGTGAGATGTTGGGTTAAGTCCCGTAACGAGCGCAACCCTTGTCCTTAGTTACCAGCACGTCATGGTGGGCACTCTAAGGAGACTGCCGGTGACAAACCGGAGGAAGGTGGGGATGACGTCAAGTCATCATGGCCCTTACGGCCTGGGCTACACACGTGCTACAATGGTCGGTACAGAGGGTTGCCAAGCCGCGAGGTGGAGCTAATCCCACAAAACCGATCGTAGTCCGGATCGCAGTCTGCAACTCGACTGCGTGAAGTCGGAATCGCTAGTAATCGCGAATCAGAATGTCGCGGTGAATACGTTCCCGGGCCTTGTACACACCGCCCGTCACACCATGGGAGTGGGTTGCACCAGAAGTAGCTAGTCTACCTTCGGGAGGACGGT

>FW215-R4

GTCGAGCGGATGAAAGGAGCTTGCTCCTGGATTCAGCGGCGGACGGGTGAGTAATGCCTAGGAATCTGCCTGGTAGTGGGGGACAACGTTTCGAAAGGAACGCTAATACCGCATACGTCCTACGGGAGAAAGCAGGGGACCTTCGGGCCTTGCGCTATCAGATGAGCCTAGGTCGGATTAGCTAGTTGGTGAGGTAATGGCTCACCAAGGCGACGATCCGTAACTGGTCTGAGAGGATGATCAGTCACACTGGAACTGAGACACGGTCCAGACTCCTACGGGAGGCAGCAGTGGGGAATATTGGACAATGGGCGAAAGCCTGATCCAGCCATGCCGCGTGTGTGAAGAAGGTCTTCGGATTGTAAAGCACTTTAAGTTGGGAGGAAGGGTTGTAGATTAATACTCTGCAATTTTGACGTTACCGACAGAATAAGCACCGGCTAACTCTGTGCCAGCAGCCGCGGTAATACAGAGGGTGCAAGCGTTAATCGGAATTACTGGGCGTAAAGCGCGCGTAGGTGGTTCGTTAAGTTGGATGTGAAATCCCCGGGCTCAACCTGGGAACTGCATCCAAAACTGGCGAGCTAGAGTATGGTAGAGGGTGGTGGAATTTCCTGTGTAGCGGTGAAATGCGTAGATATAGGAAGGAACACCAGTGGCGAAGGCGACCACCTGGACTGATACTGACACTGAGGTGCGAAAGCGTGGGGAGCAAACAGGATTAGATACCCTGGTAGTCCACGCCGTAAACGATGTCAACTAGCCGTTGGGAGCCTTGAGCTCTTAGTGGCGCAGCTAACGCATTAAGTTGACCGCCTGGGGAGTACGGCCGCAAGGTTAAAACTCAAATGAATTGACGGGGGCCCGCACAAGCGGTGGAGCATGTGGTTTAATTCGAAGCAACGCGAAGAACCTTACCAGGCCTTGACATCCAATGAACTTTCCAGAGATGGATTGGTGCCTTCGGGAACATTGAGACAGGTGCTGCATGGCTGTCGTCAGCTCGTGTCGTGAGATGTTGGGTTAAGTCCCGTAACGAGCGCAACCCTTGTCCTTAGTTACCAGCACGTAATGGTGGGCACTCTAAGGAGACTGCCGGTGACAAACCGGAGGAAGGTGGGGATGACGTCAAGTCATCATGGCCCTTACGGCCTGGGCTACACACGTGCTACAATGGTCGGTACAAAGGGTTGCCAAGCCGCGAGGTGGAGCTAATCCCATAAAACCGATCGTAGTCCGGATCGCAGTCTGCAACTCGACTGCGTGAAGTCGGAATCGCTAGTAATCGCGAATCAGAATGTCGCGGTGAATACGTTCCCGGGCCTTGTACACACCGCCCGTCACACCATGGGAGTGGGTTGCACCAGAAGTAGCTAGTCTAACCTTCGGGGGGAC

>FW215-T2

ACATGCAGTCGAGCGGATGACAGGAGCTTGCTCCTGAATTCAGCGGCGGACGGGTGAGTAATGCCTAGGAATCTGCCTGGTAGTGGGGGACAACGTTTCGAAAGGAACGCTAATACCGCATACGTCCTACGGGAGAAAGCAGGGGACCTTCGGGCCTTGCGCTATCAGATGAGCCTAGGTCGGATTAGCTAGTTGGTGAGGTAATGGCTCACCAAGGCGACGATCCGTAACTGGTCTGAGAGGATGATCAGTCACACTGGAACTGAGACACGGTCCAGAYTCCTACGGGAGGCAGCAGTGGGGAATATTGGACAATGGGCGAAAGCCTGATCCAGCCATGCCGCGTGTGTGAAGAAGGTCTTCGGATTGTAAAGCACTTTAAGTTGGGAGGAAGGGCATTAACCTAATACGTTAGTGTTTTGACGTTACCGACAGAATAAGCACCGGCTAACTCTGTGCCAGCAGCCGCGGTAATACAGAGGGTGCAAGCGTTAATCGGAATTACTGGGCGTAAAGCGCGCGTAGGTGGTTTGTTAAGTTGGATGTGAAAGCCCCGGGCTCAACCTGGGAACTGCATTCAAAACTGACAAGCTAGAGTATGGTAGAGGGTGGTGGAATTTCCTGTGTAGCGGTGAAATGCGTAGATATAGGAAGGAACACCAGTGGCGAAGGCGACCACCTGGACTGATACTGACACTGAGGTGCGAAAGCGTGGGGAGCAAACAGGATTAGATACCCTGGTAGTCCACGCCGTAAACGATGTCAACTAGCCGTTGGGAGCCTTGAGCTCTTAGTGGCGCAGCTAACGCATTAAGTTGACCGCCTGGGGAGTACGGCCGCAAGGTTAAAACTCAAATGAATTGACGGGGGCCCGCACAAGCGGTGGAGCATGTGGTTTAATTCGAAGCAACGCGAAGAACCTTACCAGGCCTTGACATCCAATGAACTTTCCAGAGATGGATTGGTGCCTTCGGGAACATTGAGACAGGTGCTGCATGGCTGTCGTCAGCTCGTGTCGTGAGATGTTGGGTTAAGTCCCGTAACGAGCGCAACCCTTGTCCTTAGTTACCAGCACGTCATGGTGGGCACTCTAAGGAGACTGCCGGTGACAAACCGGAGGAAGGTGGGGATGACGTCAAGTCATCATGGCCCTTACGGCCTGGGCTACACACGTGCTACAATGGTCGGTACAGAGGGTTGCCAAGCCGCGAGGTGGAGCTAATCCCACAAAACCGATCGTAGTCCGGATCGCAGTCTGCAACTCGACTGCGTGAAGTCGGAATCGCTAGTAATCGCGAATCAGAATGTCGCGGTGAATACGTTCCCGGGCCTTGTACACACCGCCCGTCACACCATGGGAGTGGGTTGCACCAGAAGTAGCTAGTCTAACCTTCGGGAGGACGGT

>FW602-L3

TGCAGTCGAGCGAACTGATTAGAAGCTTGCTTCTATGACGTTAGCGGCGGACGGGTGAGTAACACGTGGGCAACCTGCCTGTAAGACTGGGATAACTTCGGGAAACCGAAGCTAATACCGGATAGGATCTTCTCCTTCATGGGAGATGATTGAAAGATGGTTTCGGCTATCACTTACAGATGGGCCCGCGGTGCATTAGCTAGTTGGTGAGGTAACGGCTCACCAAGGCAACGATGCATAGCCGACCTGAGAGGGTGATCGGCCACACTGGGACTGAGACACGGCCCAGACTCCTACGGGAGGCAGCAGTAGGGAATCTTCCGCAATGGACGAAAGTCTGACGGAGCAACGCCGCGTGAGTGATGAAGGCTTTCGGGTCGTAAAACTCTGTTGTTAGGGAAGAACAAGTMCRAGAGTAACTGCTTGTACCTTGACGGTACCTAACCAGAAAGCCNACGGCTAACTACGTGCCAGCAGCCGCGGTAATACGTAGGTGGCAAGCGTTATCCGGAATTATTGGGCGTAAAGCGCGCGCAGGCGGTTTCTTAAGTCTGATGTGAAAGCCCACGGCTCAACCGTGGAGGGTCATTGGAAACTGGGGAACTTGAGTGCAGAAGAGAAAAGCGGAATTCCACGTGTAGCGGTGAAATGCGTAGAGATGTGGAGGAACACCAGTGGCGAAGGCGGCTTTTTGGTCTGTAACTGACGCTGAGGCGCGAAAGCGTGGGGAGCAAACAGGATTAGATACCCTGGTAGTCCACGCCGTAAACGATGAGTGCTAAGTGTTAGAGGGTTTCCGCCCTTTAGTGCTGCAGCTAACGCATTAAGCACTCCGCCTGGGGAGTACGGTCGCAAGACTGAAACTCAAAGGAATTGACGGGGGCCCGCACAAGCGGTGGAGCATGTGGTTTAATTCGAAGCAACGCGAAGAACCTTACCAGGTCTTGACATCCTCTGACAACTCTAGAGATAGAGCGTTCCCCTTCGGGGGACAGAGTGACAGGTGGTGCATGGTTGTCGTCAGCTCGTGTCGTGAGATGTTGGGTTAAGTCCCGCAACGAGCGCAACCCTTGATCTTAGTTGCCAGCATTTAGTTGGGCACTCTAAGGTGACTGCCGGTGACAAACCGGAGGAAGGTGGGGATGACGTCAAATCATCATGCCCCTTATGACCTGGGCTACACACGTGCTACAATGGATGGTACAAAGGGCTGCAAGACCGCGAGGTCAAGCCAATCCCATAAAACCATTCTCAGTTCGGATTGTAGGCTGCAACTCGCCTACATGAAGCTGGAATCGCTAGTAATCGCGGATCAGCATGCCGCGGTGAATACGTTCCCGGGCCTTGTACACACCGCCCGTCACACCACGAGAGTTTGTAACACCCGAAGTCGGTGGAGTAACCGTAAGGAGCTA

>FW602-L4

TGCAGTCGAGCGAATGGATTAAGAGCTTGCTCTTATGAAGTTAGCGGCGGACGGGTGAGTAACACGTGGGTAACCTGCCCATAAGACTGGGATAACTCCGGGAAACCGGGGCTAATACCGGATAACATTTTGAACTGCATGGTTCGAAATTGAAAGGCGGCTTCGGCTGTCACTTATGGATGGACCCGCGTCGCATTAGCTAGTTGGTGAGGTAACGGCTCACCAAGGCAACGATGCGTAGCCGACCTGAGAGGGTGATCGGCCACACTGGGACTGAGACACGGCCCAGACTCCTACGGGAGGCAGCAGTAGGGAATCTTCCGCAATGGACGAAAGTCTGACGGAGCAACGCCGCGTGAGTGATGAAGGCTTTCGGGTCGTAAAACTCTGTTGTTAGGGAAGAACAAGTGCTAGTTGAATAAGCTGGCACCTTGACGGTACCTAACCAGAAAGCCACGGCTAACTACGTGCCAGCAGCCGCGGTAATACGTAGGTGGCAAGCGTTATCCGGAATTATTGGGCGTAAAGCGCGCGCAGGTGGTTTCTTAAGTCTGATGTGAAAGCCCACGGCTCAACCGTGGAGGGTCATTGGAAACTGGGAGACTTGAGTGCAGAAGAGGAAAGTGGAATTCCATGTGTAGCGGTGAAATGCGTAGAGATATGGAGGAACACCAGTGGCGAAGGCGACTTTCTGGTCTGTAACTGACACTGAGGCGCGAAAGCGTGGGGAGCAAACAGGATTAGATACCCTGGTAGTCCACGCCGTAAACGATGAGTGCTAAGTGTTAGAGGGTTTCCGCCCTTTAGTGCTGAAGTTAACGCATTAAGCACTCCGCCTGGGGAGTACGGCCGCAAGGCTGAAACTCAAAGGAATTGACGGGGGCCCGCACAAGCGGTGGAGCATGTGGTTTAATTCGAAGCAACGCGAAGAACCTTACCAGGTCTTGACATCCTCTGAAAACCCTAGAGATAGGGCTTCTCCTTCGGGAGCAGAGTGACAGGTGGTGCATGGTTGTCGTCAGCTCGTGTCGTGAGATGTKGGGTTAAGTCCCGCAACGAGCGCAACCCTTGATCTTAGTTGCCATCATTAAGTTGGGCACTCTAAGGTGACTGCCGGTGACAAACCGGAGGAAGGTGGGGATGACGTCAAATCATCATGCCCCTTATGACCTGGGCTACACACGTGCTACAATGGACGGTACAAAGAGCTGCAAGACCGCGAGGTGGAGCTAATCTCATAAAACCGTTCTCAGTTCGGATTGTAGGCTGCAACTCGCCTACATGAAGCTGGAATCGCTAGTAATCGCGGATCAGCATGCCGCGGTGAATACGTTCCCGGGCCTTGTACACACCGCCCGTCACACCACGAGAGTTTGTAACACCCGAAGTCGGTGGGGTAACC

>FW602-R5

AGTCGAGCGAATCACTTCGGTGGTTAGCGGCGGACGGGTGAGTAACACGTGGGCAACCTGCCTGTAAGACTGGGATAACACCGGGAAACCGGTGCTAATACCGGATAATCCTTTTCCTCTCATGAGGAAAAGCTGAAAGTCGGTTTCGGCTGACACTTACAGATGGGCCCGCGGCGCATTAGCTAGTTGGTGAGGTAACGGCTCACCAAGGCGACGATGCGTAGCCGACCTGAGAGGGTGATCGGCCACACTGGGACTGAGACACGGCCCAGACTCCTACGGGAGGCAGCAGTAGGGAATCTTCCACAATGGACGAAAGTCTGATGGAGCAACGCCGCGTGAGCGATGAAGGCCTTCGGGTCGTAAAGCTCTGTTGTTAGGGAAGAACAAGTACCGGAGTAACTGCCGGTACCTTGACGGTACCTAACCAGAAAGCCACGGCTAACTACGTGCCAGCAGCCGCGGTAATACGTAGGTGGCAAGCGTTGTCCGGAATTATTGGGCGTAAAGCGCGCGCAGGCGGTCCTTTAAGTCTGATGTGAAAGCCCACGGCTCAACCGTGGAGGGTCATTGGAAACTGGGGGACTTGAGTGCAGAAGAGGAAAGCGGAATTCCACGTGTAGCGGTGAAATGCGTAGAGATGTGGAGGAACACCAGTGGCGAAGGCGGCTTTCTGGTCTGTAACTGACGCTGAGGCGCGAAAGCGTGGGGAGCAAACAGGATTAGATACCCTGGTAGTCCACGCCGTAAACGATGAGTGCTAAGTGTTAGGGGGTTTCCGCCCCTTAGTGCTGCAGCTAACGCATTAAGCACTCCGCCTGGGGAGTACGGCCGCAAGGCTGAAACTCAAAGGAATTGACGGGGGCCCGCACAAGCGGTGGAGCATGTGGTTTAATTCGAAGCAACGCGAAGAACCTTACCAGGTCTTGACATCCTCTGACACTCCTAGAGATAGGACGTTCCCCTTCGGGGGACAGAGTGACAGGTGGTGCATGGTTGTCGTCAGCTCGTGTCGTGAGATGTTGGGTTAAGTCCCGCAACGAGCGCAACCCTTGATCTTAGTTGCCAGCATTYAGTTGGGCACTCTAAGGTGACTGCCGGTGACAAACCGGNAGGAAGGTGGGGATGACGTCAAATCATCATGCCCCTTATGACCTGGGCTACACACGTGCTACAATGGATGGTACAAAGGGCTGCAAGACCGCGAGGTTTAGCCAATCCCATAAAACCATTCTCAGTTCGGATTGCAGGCTGCAACTCGCCTGCATGAAGCCGGAATCGCTAGTAATCGCGGATCAGCATGCCGCGGTGAATACGTTCCCGGGCCTTGTACACACCGCCCGTCACACCACGAGAGTTTGTAACACCCGAAGTCGGTGGGGTAACCGTAAGGAGCCAGCCGCCTA

>MPBC1-4

CGGCAGCACGGGAGCAATCCTGGTGGCGAGTGGCGAACGGGTGAGTAATACATCGGAACGTGCCCTGTCGTGGGGGATAACTAGTCGAAAGATTAGCTAATACCGCATACGACCTGAGGGTGAAAGCGGGGGACCGTAAGGCCTCGCGCGATAGGAGCGGCCGATGTCTGATTAGCTAGTTGGTGGGGTAAAGGCCCACCAAGGCGACGATCAGTAGCTGGTCTGAGAGGACGATCAGCCACACTGGGACTGAGACACGGCCCAGACTCCTACGGGAGGCAGCAGTGGGGAATTTTGGACAATGGGGGCAACCCTGATCCAGCAATGCCGCGTGTGTGAAGAAGGCCTTCGGGTTGTAAAGCACTTTTGTCCGGAAAGAAATCCCTTGCTCTAATACAGCGGGGGGATGACGGTACCGGAAGAATAAGCACCGGCTAACTACGTGCCAGCAGCCGCGGTAATACGTAGGGTGCGAGCGTTAATCGGAATTACTGGGCGTAAAGCGTGCGCAGGCGGTTTTGTAAGACAGGCGTGAAATCCCCGAGCTCAACTTGGGAATGGCGCTTGTGACTGCAAGGCTAGAGTATGTCAGAGGGGGGTAGAATTCCACGTGTAGCAGTGAAATGCGTAGAGATGTGGAGGAATACCGATGGCGAAGGCAGCCCCCTGGGACGTCACTGACGCTCATGCACGAAAGCGTGGGGAGCAAACAGGATTAGATACCCTGGTAGTCCACGCCCTAAACGATGTCAACTAGTTGTTGGGGATTCATTTCTTCAGTAACGTAGCTAACGCGTGAAGTTGACCGCCTGGGGAGTACGGTCGCAAGATTAAAACTCAAAGGAATTGACGGGGACCCGCACAAGCGGTGGATGATGTGGATTAATTCGATGCAACGCGAAAAACCTTACCTACCCTTGACATGCCACTAACGAAGCAGAGATGCATCAGGTGCCCGAAAGGGAAAGTGGACACAGGTGCTGCATGGCTGTCGTCAGCTCGTGTCGTGAGATGTTGGGTTAAGTCCCGCAACGAGCGCAACCCTTGTCTTTAGTTGCTACGCAAGAGCACTCTAGAGAGACTGCCGGTGACAAACCGGAGGAAGGTGGGGATGACGTCAAGTCCTCATGGCCCTTATGGGTAGGGCTTCACACGTCATACAATGGTGCGTACAGAGGGTTGCCAACCCGCGAGGGGGAGCTAATCCCAGAAAACGCATCGTAGTCCGGATCGTAGTCTGCAACTCGACTACGTGAAGCTGGAATCGCTAGTAATCGCGGATCAGCATGCCGCGGTGAATACGTTCCCGGGTCTTGTACACACCGCCCGTCACACCATGGGAGTGGGTTTTGCCAGAAGTAGTTAGCCTAACCGCAAGGAGGGCGATACC

>MPBC4-3

CGGCAGCACGGGAGCAATCCTGGTGGCGAGTGGCGAACGGGTGAGTAATACATCGGAACGTGCCCTGTCGTGGGGGATAACTAGTCGAAAGATTAGCTAATACCGCATACGACCTGAGGGTGAAAGCGGGGGACCGTAAGGCCTCGCGCGATAGGAGCGGCCGATGTCTGATTAGCTAGTTGGTGGGGTAAAGGCCCACCAAGGCGACGATCAGTAGCTGGTCTGAGAGGACGATCAGCCACACTGGGACTGAGACACGGCCCAGACTCCTACGGGAGGCAGCAGTGGGGAATTTTGGACAATGGGGGCAACCCTGATCCAGCAATGCCGCGTGTGTGAAGAAGGCCTTCGGGTTGTAAAGCACTTTTGTCCGGAAAGAAATCCCTTGCCCTAATACGGCGGGGGGATGACGGTACCGGAAGAATAAGCACCGGCTAACTACGTGCCAGCAGCCGCGGTAATACGTAGGGTGCGAGCGTTAATCGGAATTACTGGGCGTAAAGCGTGCGCAGGCGGTTTTGTAAGACAGGCGTGAAATCCCCGAGCTCAACTTGGGAATGGCGCTTGTGACTGCAAGGCTAGAGTATGTCAGAGGGGGGTAGAATTCCACGTGTAGCAGTGAAATGCGTAGAGATGTGGAGGAATACCGATGGCGAAGGCAGCCCCCTGGGACGTCACTGACGCTCATGCACGAAAGCGTGGGGAGCAAACAGGATTAGATACCCTGGTAGTCCACGCCCTAAACGATGTCAACTAGTTGTTGGGGATTCATTTCTTCAGTAACGTAGCTAACGCGTGAAGTTGACCGCCTGGGGAGTACGGTCGCAAGATTAAAACTCAAAGGAATTGACGGGGACCCGCACAAGCGGTGGATGATGTGGATTAATTCGATGCAACGCGAAAAACCTTACCTACCCTTGACATGCCACTAACGAAGCAGAGATGCATTAGGTGCTCGAAAGAGAAAGTGGACACAGGTGCTGCATGGCTGTCGTCAGCTCGTGTCGTGAGATGTTGGGTTAAGTCCCGCAACGAGCGCAACCCTTGTCTTTAGTTGCTACGCAAGAGCACTCTAGAGAGACTGCCGGTGACAAACCGGAGGAAGGTGGGGATGACGTCAAGTCCTCATGGCCCTTATGGGTAGGGCTTCACACGTCATACAATGGTGCGTACAGAGGGTTGCCAACCCGCGAGGGGGAGCTAATCCCAGAAAACGCATCGTAGTCCGGATCGTAGTCTGCAACTCGACTACGTGAAGCTGGAATCGCTAGTAATCGCGGATCAGCATGCCGCGGTGAATACGTTCCCGGGTCTTGTACACACCGCCCGTCACACCATGGGAGTGGGTTTTGCCAGAAGTAGTTAGCCTAACCGCAAGGAGGGCGAT

>MPBC4-4

GCAGCACGGGAGCAATCCTGGTGGCGAGTGGCGAACGGGTGAGTAATACATCGGAACGTGCCCTGTCGTGGGGGATAACTAGTCGAAAGATTAGCTAATACCGCATACGACCTGAGGGTGAAAGCGGGGGACCGTAAGGCCTCGCGCGATAGGAGCGGCCGATGTCTGATTAGCTAGTTGGTGGGGTAAAGGCCCACCAAGGCGACGATCAGTAGCTGGTCTGAGAGGACGATCAGCCACACTGGGACTGAGACACGGCCCAGACTCCTACGGGAGGCAGCAGTGGGGAATTTTGGACAATGGGGGCAACCCTGATCCAGCAATGCCGCGTGTGTGAAGAAGGCCTTCGGGTTGTAAAGCACTTTTGTCCGGAAAGAAATCCCTTGCCCTAATACGGCGGGGGGATGACGGTACCGGAAGAATAAGCACCGGCTAACTACGTGCCAGCAGCCGCGGTAATACGTAGGGTGCGAGCGTTAATCGGAATTACTGGGCGTAAAGCGTGCGCAGGCGGTTTTGTAAGACAGGCGTGAAATCCCCGAGCTCAACTTGGGAATGGCGCTTGTGACTGCAAGGCTAGAGTATGTCAGAGGGGGGTAGAATTCCACGTGTAGCAGTGAAATGCGTAGAGATGTGGAGGAATACCGATGGCGAAGGCAGCCCCCTGGGACGTCACTGACGCTCATGCACGAAAGCGTGGGGAGCAAACAGGATTAGATACCCTGGTAGTCCACGCCCTAAACGATGTCAACTAGTTGTTGGGGATTCATTTCTTCAGTAACGTAGCTAACGCGTGAAGTTGACCGCCTGGGGAGTACGGTCGCAAGATTAAAACTCAAAGGAATTGACGGGGACCCGCACAAGCGGTGGATGATGTGGATTAATTCGATGCAACGCGAAAAACCTTACCTACCCTTGACATGCCACTAACGAAGCAGAGATGCATTAGGTGCTCGAAAGAGAAAGTGGACACAGGTGCTGCATGGCTGTCGTCAGCTCGTGTCGTGAGATGTTGGGTTAAGTCCCGCAACGAGCGCAACCCTTGTCTTTAGTTGCTACGCAAGAGCACTCTAGAGAGACTGCCGGTGACAAACCGGAGGAAGGTGGGGATGACGTCAAGTCCTCATGGCCCTTATGGGTAGGGCTTCACACGTCATACAATGGTGCGTACAGAGGGTTGCCAACCCGCGAGGGGGAGCTAATCCCAGAAAACGCATCGTAGTCCGGATCGTAGTCTGCAACTCGACTACGTGAAGCTGGAATCGCTAGTAATCGCGGATCAGCATGCCGCGGTGAATACGTTCCCGGGTCTTGTACACACCGCCCGTCACACCATGGGAGTGGGTTTTGCCAGAAGTAGTTAGCCTAACCGCAAGGAGGGCGAT

>MPBD4-3

TGCAGTCGAACGGCAGCGCGGGAGCAATCCTGGCGGCGAGTGGCGAACGGGTGAGTAATACATCGGAACGTGCCCAATCGTGGGGGATAACGCAGCGAAAGCTGTGCTAATACCGCATACGATCTACGGATGAAAGCAGGGGATCGCAAGACCTTGCGCGAATGGAGCGGCCGATGGCAGATTAGGTAGTTGGTGAGGTAAAGGCTCACCAAGCCTTCGATCTGTAGCTGGTCTGAGAGGACGACCAGCCACACTGGGACTGAGACACGGCCCAGACTCCTACGGGAGGCAGCAGTGGGGAATTTTGGACAATGGGCGAAAGCCTGATCCAGCCATGCCGCGTGCAGGATGAAGGCCTTCGGGTTGTAAACTGCTTTTGTACGGAACGAAACGGTCTTTTCTAATAAAGAAGGCTAATGACGGTACCGTAAGAATAAGCACCGGCTAACTACGTGCCAGCAGCCGCGGTAATACGTAGGGTGCAAGCGTTAATCGGAATTACTGGGCGTAAAGCGTGCGCAGGCGGTGATGTAAGACAGTTGTGAAATCCCCGGGCTCAACCTGGGAACTGCATCTGTGACTGCATCGCTGGAGTACGGCAGAGGGGGATGGAATTCCGCGTGTAGCAGTGAAATGCGTAGATATGCGGAGGAACACCGATGGCGAAGGCAATCCCCTGGGCCTGTACTGACGCTCATGCACGAAAGCGTGGGGAGCAAACAGGATTAGATACCCTGGTAGTCCACGCCCTAAACGATGTCAACTGGTTGTTGGGTCTTCACTGACTCAGTAACGAAGCTAACGCGTGAAGTTGACCGCCTGGGGAGTACGGCCGCAAGGTTGAAACTCAAAGGAATTGACGGGGACCCGCACAAGCGGTGGATGATGTGGTTTAATTCGATGCAACGCGAAAAACCTTACCCACCTTTGACATGTACGGAATTCGCCAGAGATGGCTTAGTGCTCGAAAGAGAACCGTAACACAGGTGCTGCATGGCTGTCGTCAGCTCGTGTCGTGAGATGTTGGGTTAAGTCCCGCAACGAGCGCAACCCTTGTCATTAGTTGCTACATTCAGTTGGGCACTCTAATGAGACTGCCGGTGACAAACCGGAGGAAGGTGGGGATGACGTCAAGTCCTCATGGCCCTTATAGGTGGGGCTACACACGTCATACAATGGCTGGTACAAAGGGTTGCCAACCCGCGAGGGGGAGCTAATCCCATAAAACCAGTCGTAGTCCGGATCGCAGTCTGCAACTCGACTGCGTGAAGTCGGAATCGCTAGTAATCGTGGATCAGAATGTCACGGTGAATACGTTCCCGGGTCTTGTACACACCGCCCGTCACACCATGGGAGCGGGTTCTGCCAGAAGTAGTTAGCTTAACCGCAAGGAGGGCGAT

>MPBD4-4

CGGCAGCGCGGGAGCAATCCTGGCGGCGAGTGGCGAACGGGTGAGTAATACATCGGAACGTGCCCAATCGTGGGGGATAACGCAGCGAAAGCTGTGCTAATACCGCATACGATCTACGGATGAAAGCAGGGGATCGCAAGACCTTGCGCGAATGGAGCGGCCGATGGCAGATTAGGTAGTTGGTGAGGTAAAGGCTCACCAAGCCTTCGATCTGTAGCTGGTCTGAGAGGACGACCAGCCACACTGGGACTGAGACACGGCCCAGACTCCTACGGGAGGCAGCAGTGGGGAATTTTGGACAATGGGCGAAAGCCTGATCCAGCCATGCCGCGTGCAGGATGAAGGCCTTCGGGTTGTAAACTGCTTTTGTACGGAACGAAACGGTCTTTTCTAATAAAGAAGGCTAATGACGGTACCGTAAGAATAAGCACCGGCTAACTACGTGCCAGCAGCCGCGGTAATACGTAGGGTGCAAGCGTTAATCGGAATTACTGGGCGTAAAGCGTGCGCAGGCGGTGATGTAAGACAGTTGTGAAATCCCCGGGCTCAACCTGGGAACTGCATCTGTGACTGCATCGCTGGAGTACGGCAGAGGGGGATGGAATTCCGCGTGTAGCAGTGAAATGCGTAGATATGCGGAGGAACACCGATGGCGAAGGCAATCCCCTGGGCCTGTACTGACGCTCATGCACGAAAGCGTGGGGAGCAAACAGGATTAGATACCCTGGTAGTCCACGCCCTAAACGATGTCAACTGGTTGTTGGGTCTTCACTGACTCAGTAACGAAGCTAACGCGTGAAGTTGACCGCCTGGGGAGTACGGCCGCAAGGTTGAAACTCAAAGGAATTGACGGGGACCCGCACAAGCGGTGGATGATGTGGTTTAATTCGATGCAACGCGAAAAACCTTACCCACCTTTGACATGTACGGAATTCGCCAGAGATGGCTTAGTGCTCGAAAGAGAACCGTAACACAGGTGCTGCATGGCTGTCGTCAGCTCGTGTCGTGAGATGTTGGGTTAAGTCCCGCAACGAGCGCAACCCTTGTCATTAGTTGCTACATTCAGTTGGGCACTCTAATGAGACTGCCGGTGACAAACCGGAGGAAGGTGGGGATGACGTCAAGTCCTCATGGCCCTTATAGGTGGGGCTACACACGTCATACAATGGCTGGTACAAAGGGTTGCCAACCCGCGAGGGGGAGCTAATCCCATAAAACCAGTCGTAGTCCGGATCGCAGTCTGCAACTCGACTGCGTGAAGTCGGAATCGCTAGTAATCGTGGATCAGAATGTCACGGTGAATACGTTCCCGGGTCTTGTACACACCGCCCGTCACACCATGGGAGCGGGTTCTGCCAGAAGTAGTTAGCTTAACCGCAAGGAGGGCGAT

>MPBD7-2

GGTCTTCGGATGCTGACGAGTGGCGAACGGGTGAGTAATACATCGGAACGTGCCCGAGAGTGGGGGATAACGAAGCGAAAGCTTTGCTAATACCGCATACGATCTCAGGATGAAAGCAGGGGACCGCAAGGCCTTGCGCTCACGGAGCGGCCGATGGCAGATTAGGTAGTTGGTGGGATAAAAGCTTACCAAGCCGACGATCTGTAGCTGGTCTGAGAGGACGACCAGCCACACTGGGACTGAGACACGGCCCAGACTCCTACGGGAGGCAGCAGTGGGGAATTTTGGACAATGGGCGCAAGCCTGATCCAGCCATGCCGCGTGCAGGATGAAGGCCTTCGGGTTGTAAACTGCTTTTGTACGGAACGAAAAGACTCTGGTTAATACCTGGGGTCCATGACGGTACCGTAAGAATAAGCACCGGCTAACTACGTGCCAGCAGCCGCGGTAATACGTAGGGTGCGAGCGTTAATCGGAATTACTGGGCGTAAAGCGTGCGCAGGCGGTTATATAAGACAGATGTGAAATCCCCGGGCTCAACCTGGGAACTGCATTTGTGACTGTATAGCTAGAGTACGGCAGAGGGGGATGGAATTCCGCGTGTAGCAGTGAAATGCGTAGATATGCGGAGGAACACCGATGGCGAAGGCAATCCCCTGGGCCTGTACTGACGCTCATGCACGAAAGCGTGGGGAGCAAACAGGATTAGATACCCTGGTAGTCCACGCCCTAAACGATGTCAACTGGTTGTTGGGTCTTCACTGACTCAGTAACGAAGCTAACGCGTGAAGTTGACCGCCTGGGGAGTACGGCCGCAAGGTTGAAACTCAAAGGAATTGACGGGGACCCGCACAAGCGGTGGATGATGTGGTTTAATTCGATGCAACGCGAAAAACCTTACCCACCTTTGACATGTACGGAATCCTTTAGAGATAGAGGAGTGCTCGAAAGAGAACCGTAACACAGGTGCTGCATGGCTGTCGTCAGCTCGTGTCGTGAGATGTTGGGTTAAGTCCCGCAACGAGCGCAACCCTTGTCATTAGTTGCTACATTCAGTTGGGCACTCTAATGAGACTGCCGGTGACAAACCGGAGGAAGGTGGGGATGACGTCAAGTCCTCATGGCCCTTATAGGTGGGGCTACACACGTCATACAATGGCTGGTACAGAGGGTTGCCAACCCGCGAGGGGGAGCCAATCCCATAAAGCCAGTCGTAGTCCGGATCGCAGTCTGCAACTCGACTGCGTGAAGTCGGAATCGCTAGTAATCGCGGATCAGAATGTCGCGGTGAATACGTTCCCGGGTCTTGTACACACCGCCCGTCACACCATGGGAGCGGGTTCTGCCAGAAGTAGTTAGCCTAACCGCAAGGAGGGCGAT

>MPBE1-2

AGTCGAACGGCAGCACGGGAGCAATCCTGGTGGCGAGTGGCGAACGGGTGAGTAATACATCGGAACGTGCCCTGTCGTGGGGGATAACTAGTCGAAAGATTAGCTAATACCGCATACGACCTGAGGGTGAAAGCGGGGGACCGTAAGGCCTCGCGCGATAGGAGCGGCCGATGTCTGATTAGCTAGTTGGTGGGGTAAAGGCCCACCAAGGCGACGATCAGTAGCTGGTCTGAGAGGACGATCAGCCACACTGGGACTGAGACACGGCCCAGACTCCTACGGGAGGCAGCAGTGGGGAATTTTGGACAATGGGGGCAACCCTGATCCAGCAATGCCGCGTGTGTGAAGAAGGCCTTCGGGTTGTAAAGCACTTTTGTCCGGAAAGAAATCCCCTGCTCTAATACAGCGGGGGGATGACGGTACCGGAAGAATAAGCACCGGCTAACTACGTGCCAGCAGCCGCGGTAATACGTAGGGTGCGAGCGTTAATCGGAATTACTGGGCGTAAAGCGTGCGCAGGCGGTTTTGTAAGACAGGCGTGAAATCCCCGAGCTCAACTTGGGAATGGCGCTTGTGACTGCAAGGCTAGAGTATGTCAGAGGGGGGTAGAATTCCACGTGTAGCAGTGAAATGCGTAGAGATGTGGAGGAATACCGATGGCGAAGGCAGCCCCCTGGGACGTCACTGACGCTCATGCACGAAAGCGTGGGGAGCAAACAGGATTAGATACCCTGGTAGTCCACGCCCTAAACGATGTCAACTAGTTGTTGGGGATTCATTTCTTCAGTAACGTAGCTAACGCGTGAAGTTGACCGCCTGGGGAGTACGGTCGCAAGATTAAAACTCAAAGGAATTGACGGGGACCCGCACAAGCGGTGGATGATGTGGATTAATTCGATGCAACGCGAAAAACCTTACCTACCCTTGACATGCCACTAACGAAGCAGAGATGCATCAGGTGCCCGAAAGGGAAAGTGGACACAGGTGCTGCATGGCTGTCGTCAGCTCGTGTCGTGAGATGTTGGGTTAAGTCCCGCAACGAGCGCAACCCTTGTCTTTAGTTGCTACGCAAGAGCACTCTAGAGAGACTGCCGGTGACAAACCGGAGGAAGGTGGGGATGACGTCAAGTCCTCATGGCCCTTATGGGTAGGGCTTCACACGTCATACAATGGTGCGTACAGAGGGTTGCCAACCCGCGAGGGGGAGCTAATCCCAGAAAACGCATCGTAGTCCGGATCGTAGTCTGCAACTCGACTACGTGAAGCTGGAATCGCTAGTAATCGCGGATCAGCATGCCGCGGTGAATACGTTCCCGGGTCTTGTACACACCGCCCGTCACACCATGGGAGTGGGTTTTGCCAGAAGTAGTTAGCCTAACCGCAAGGAGGGCGAT

>MPBE1-4

ACGGGAGCAATCCTGGTGGCGAGTGGCGAACGGGTGAGTAATACATCGGAACGTGCCCTGTCGTGGGGGATAACTAGTCGAAAGATTAGCTAATACCGCATACGACCTGAGGGTGAAAGCGGGGGACCGTAAGGCCTCGCGCGATAGGAGCGGCCGATGTCTGATTAGCTAGTTGGTGGGGTAAAGGCCCACCAAGGCGACGATCAGTAGCTGGTCTGAGAGGACGATCAGCCACACTGGGACTGAGACACGGCCCAGACTCCTACGGGAGGCAGCAGTGGGGAATTTTGGACAATGGGGGCAACCCTGATCCAGCAATGCCGCGTGTGTGAAGAAGGCCTTCGGGTTGTAAAGCACTTTTGTCCGGAAAGAAATCCCCTGCTCTAATACAGCGGGGGGATGACGGTACCGGAAGAATAAGCACCGGCTAACTACGTGCCAGCAGCCGCGGTAATACGTAGGGTGCGAGCGTTAATCGGAATTACTGGGCGTAAAGCGTGCGCAGGCGGTTTTGTAAGACAGGCGTGAAATCCCCGAGCTCAACTTGGGAATGGCGCTTGTGACTGCAAGGCTAGAGTATGTCAGAGGGGGGTAGAATTCCACGTGTAGCAGTGAAATGCGTAGAGATGTGGAGGAATACCGATGGCGAAGGCAGCCCCCTGGGACGTCACTGACGCTCATGCACGAAAGCGTGGGGAGCAAACAGGATTAGATACCCTGGTAGTCCACGCCCTAAACGATGTCAACTAGTTGTTGGGGATTCATTTCTTCAGTAACGTAGCTAACGCGTGAAGTTGACCGCCTGGGGAGTACGGTCGCAAGATTAAAACTCAAAGGAATTGACGGGGACCCGCACAAGCGGTGGATGATGTGGATTAATTCGATGCAACGCGAAAAACCTTACCTACCCTTGACATGCCACTAACGAAGCAGAGATGCATCAGGTGCCCGAAAGGGAAAGTGGACACAGGTGCTGCATGGCTGTCGTCAGCTCGTGTCGTGAGATGTTGGGTTAAGTCCCGCAACGAGCGCAACCCTTGTCTTTAGTTGCTACGCAAGAGCACTCTAGAGAGACTGCCGGTGACAAACCGGAGGAAGGTGGGGATGACGTCAAGTCCTCATGGCCCTTATGGGTAGGGCTTCACACGTCATACAATGGTGCGTACAGAGGGTTGCCAACCCGCGAGGGGGAGCTAATCCCAGAAAACGCATCGTAGTCCGGATCGTAGTCTGCAACTCGACTACGTGAAGCTGGAATCGCTAGTAATCGCGGATCAGCATGCCGCGGTGAATACGTTCCCGGGTCTTGTACACACCGCCCGTCACACCATGGGAGTGGGTTTTGCCAGAAGTAGTTAGCCTAACCGCAAGGAGGGCGAT

>FW104-L1

GTCGAGCGAATGGATTAAGAGCTTGCTCTTATGAAGTTAGCGGCGGACGGGTGAGTAACACGTGGGTAACCTGCCCATAAGACTGGGATAACTCCGGGAAACCGGGGCTAATACCGGATAACATTTTGAACCGCATGGTTCGAAATTGAAAGGCGGCTTCGGCTGTCACTTATGGATGGACCCGCGTCGCATTAGCTAGTTGGTGAGGTAACGGCTCACCAAGGCAACGATGCGTAGCCGACCTGAGAGGGTGATCGGCCACACTGGGACTGAGACACGGCCCAGACTCCTACGGGAGGCAGCAGTAGGGAATCTTCCGCAATGGACGAAAGTCTGACGGAGCAACGCCGCGTGAGTGATGAAGGCTTTCGGGTCGTAAAACTCTGTTGTTAGGGAAGAACAAGTGCTAGTTGAATAAGCTGGCACCTTGACGGTACCTAACCAGAAAGCCACGGCTAACTACGTGCCAGCAGCCGCGGTAATACGTAGGTGGCAAGCGTTATCCGGAATTATTGGGCGTAAAGCGCGCGCAGGTGGTTTCTTAAGTCTGATGTGAAAGCCCACGGCTCAACCGTGGAGGGTCATTGGAAACTGGGAGACTTGAGTGCAGAAGAGGAAAGTGGAATTCCATGTGTAGCGGTGAAATGCGTAGAGATATGGAGGAACACCAGTGGCGAAGGCGACTTTCTGGTCTGTAACTGACACTGAGGCGCGAAAGCGTGGGGAGCAAACAGGATTAGATACCCTGGTAGTCCACGCCGTAAACGATGAGTGCTAAGTGTTAGAGGGTTTCCGCCCTTTAGTGCTGAAGTTAACGCATTAAGCACTCCGCCTGGGGAGTACGGCCGCAAGGCTGAAACTCAAAGGAATTGACGGGGGCCCGCACAAGCGGTGGAGCATGTGGTTTAATTCGAAGCAACGCGAAGAACCTTACCAGGTCTTGACATCCTCTGACAACCCTAGAGATAGGGCTTCTCCTTCGGGAGCAGAGTGACAGGTGGTGCATGGTTGTCGTCAGCTCGTGTCGTGAGATGTTGGGTTAAGTCCCGCAACGAGCGCAACCCTTGATCTTAGTTGCCATCATTAAGTTGGGCACTCTAAGTGACTGCCGGTGACAAACCGGAGGAAGGTGGGGATGACGTCAAATCATCATGCCCCTTATGACCTGG

>FW104-R3

ACTAACGCCTTCTGGAGCAACCCACTCCCATGGTGTGACGGGCGGTGTGTACAAGGCCCGGGAACGTATTCACCGCGGCATAGCTGATCCGCGATTACTAGCGATTCCGACTTCATGGAGTCGAGTTGCAGACTCCAATCCGGACTGGGATCGGCTTTCTGGGATTGGCTCCACCTCGCGGTATTGCAACCCTCTGTACCGACCATTGTAGTACGTGTGTAGCCCTGGCCGTAAGGGCCATGATGACTTGACGTCATCCCCACCTTCCTCCGGTTTGTCACCGGCAGTCTCCTTAGAGTTCCCACCATTACGTGCTGGCAACTAAGGACAAGGGTTGCGCTCGTTGCGGGACTTAACCCAACATCTCACGACACGAGCTGACGACAGCCATGCAGCACCTGTGTTCCGATTCCCGAAGGCACTCCCGCATCTCTGCAGGATTCCGGACATGTCAAGGCCAGGTAAGGTTCTTCGCGTTGCATCGAATTAAACCACATACTCCACCGCTTGTGCGGGCCCCCGTCAATTCCTTTGAGTTTCAGTCTTGCGACCGTACTCCCCAGGCGGCGAACTTAACGCGTTAGCTTCGACACTGATCTCCGAGTTGAGACCAACATCCAGTTCGCATCGTTTAGGGCGTGGACTACCAGGGTATCTAATCCTGTTTGCTCCCCACGCTTTCGTGCTTCAGCGTCAGTGTTGTCCCAGATGGCCGCCTTCGCCACTGATGTTCCTCCCGATCTCTACGCATTTCACCGCTACACCGGGAATTCCACCATCCTCTGACACACTCTAGCCACCCAGTATCCATCGCCATTCCCAGGTTGAGCCCGGGGATTTCACGACAGACTTAAGTAACCGCCTACGCACCCTTTACGCCCAGTAATTCCGATTAACGCTTGCACCCTTCGTATTACCGCGGCTGCTGGCACGAAGTTAGCCGGTGCTTATTCCTCAGGTACCGTCAGCCCCATAGGGTATTAACCCGTGGTATTTCGCTCCTGATAAAAGTGCTTTACAACCCGAAGGCCTTCTTCACACACGCGGCATTGCTGGATCAGGCTTGCGCCCATTGGCCAATATTCCCCACTGCTGCCTCCCGTAGGAGTCTGGGCCGGGGCTCAGTCCCAGTGTGGCTGATCATCCTCTCAACCAGCTAGCGATCGTCGCCTTGGTGGCCATTACCC

>GW704-F3

AGCGGNANAGAGAAGCTTGCTTCTCTTGAGAGCGGCGGACGGGTGAGTAANGCCTAGGAATCTGNCNGGNAGTGGGGGATAACGTTCGGAAACGGACGCTAATACCGCATACGTCCTACNGGAGAAAGCAGGGGACCTTCNGGCCTTGCGCTATCANATGAGCCTAGGTCGGATTAGCTAGTTGGTGAGGTAATGGNTCACCNAGGCGACGATCCGTAACTGGTCTGANAGGATGATCNGTCNCNCTGGAACTGANACACGGTCCANACTCCTACGGGAGGCNNNNNTGGGGAATATTGGACAATGGGNNAAAGCCTGATCCANCCNTGCCGCGTGTGTGAAGAAGGTCTTCGGATTGTAAAGCACTTTAAGTTGGGAGGAAGGGCAGTTACCTNNNACGTGATTGTTTTGACGTTACCGACAGAATAANCACCGGCTAACTCTGTGCCANCANCCGCGGTAATACAGAGGGTGCAAGCGTTAATCGGAATTACTGGGCGTAAAGCGCGCGTAGGTGGTTAGTTAAGTTGGATGTGAAATCCCCGGGCTCAACCTGGGAACTGCATTCAAAACTGACTGACTAGAGTATGGTAGAGGGTGGTGGAATTTCCTGTGTAGCGGTGAAATGCGTAGATATAGGAAGGAACACCAGTGGCGAAGGCGACCACCTGGACTGATACTGACACTGAGGTGCGAAAGCGTGGGGAGCAAACAGGATTAGATACCCTGGTAGTCCACGCCGTAAACGATGTCAACTAGCCGTTGGGAGCCTTGAGCTCTTAGTGGCGCAGCTAACGCATTAAGTTGACCGCCTGGGGAGTACGGCCGCAAGGTTAAAACTCAANTGAATTGACGGGGGCCCGCACAAGCGGTGGAGCATGTGGTTTAATTCGAAGCAACGCGAAGAACCTTACCAGGCCTTGACATCCAATGAACTTTCTAGAGATAGATTGGTGCCTTCGGGAACATTGAGACAGGTGCTGCATGGCTGTCGTCAGCTCGTGTCGTGAGATGTTGGGTTAAGTCCCGTAACGAGCGCAACCCTTGTCCTTAGTTACCAGCACGTAATGGTGGGCACTCTAAGGAGACTGCCGGTGACAAACCGGAGGAAGGTGGGGATGACGTCAAGTCATCATGGCCCTTACGGCCTGGGCTACACACGTGCTACAATGGTCGGTACAGAGGGTTGCCAAGCCGCGAGGTGGAGCTAATCCCAGAAAACCGATCGTAGTCCGGATCGCAGTCTGCAACTCGACTGCGTGAAGTCGGAATCGCTAGTAATCGCGAATCAGAATGTCGCGGTGAATACGTTCCCGGGCCTTGTACACACCGCCCGTCACACCATGGGAGTGGGTTGCACCAGAAGTAGCTAGTCTAACCNTCGGGGGGACGG

>GW704-F5

CCCCGAAGGTTAGACTAGCTACTTCTGGTGCAACCCACTCCCATGGTGTGACGGGCGGTGTGTACAAGGCCCGGGAACGTATTCACCGCGACATTCTGATTCGCGATTACTAGCGATTCCGACTTCACGCAGTCGAGTTGCAGACTGCGATCCGGACTACGATCGGTTTTCTGGGATTAGCTCCACCTCGCGGCTTGGCAACCCTCTGTACCGACCATTGTAGCACGTGTGTAGCCCAGGCCGTAAGGGCCATGATGACTTGACGTCATCCCCACCTTCCTCCGGTTTGTCACCGGCAGTCTCCTTAGAGTGCCCACCATTACGTGCTGGTAACTAAGGACAAGGGTTGCGCTCGTTACGGGACTTAACCCAACATCTCACGACACGAGCTGACGACAGCCATGCAGCACCTGTCTCAATGTTCCCGAAGGCACCAATCTATCTCTAGAAAGTTCATTGGATGTCAAGGCCTGGTAAGGTTCTTCGCGTTGCTTCGAATTAAACCACATGCTCCACCGCTTGTGCGGGNCCCCGTCAATTCATTTGAGTTTTAACCTTGCGGCCGTACTCCCCAGGCGGTCAACTTAATGCGTTAGCTGCGCCACTAAGAGCTCAAGGCTCCCAACGGCTAGTTGACATCGTTTACGGCGTGGACTACCAGGGTATCTAATCCTGTTTGCTCCCCACGCTTTCGCACCTCAGTGTCAGTATCAGTCCAGGTGGTCGCCTTCGCCACTGGTGTTCCTTCCTATATCTACGCATTTCACCGCTACACAGGAAATTCCACCACCCTCTACCATACTCTAGTCAGTCAGTTTTGAATGCAGTTCCCAGGTTGAGCCCGGGGATTTCACATCCAACTTAACTAACCACCTACGCGCGCTTTACGCCCAGTAATTCCGATTAACGCTTGCACCCTCTGTATTACCGCGGCTGCTGGCACAGAGTTAGCCGGTGCTTATTCTGTCGGTAACGTCAAAACAATCACGTATTAGGTAACTNNNNNNCCTCCCAACTTAAAGTGCTTTACAATCCGAAGACCTTCTTCACACACGCGGCATGGCTGGATCAGGCTTTCGCCCATTGTCCAATATTCCCCACTGCTGCCTCCCGTAGGAGTCTGGACCGTGTCTCAGTTCCAGTGTGACTGATCATCCTCTCAGACCAGTTACGGATCGTCGCCTTGGTGAGCCATTACCTCACCAACTAGCTAATCCGACCTAGGCTCATCTGATAGCGCAAGGCCCGAAGGTCCCCTGCTTTCTCCCGTAGGACGTATGCGGTATTAGCGTCCGTTTCCGAACGTTATCCCCCACTACCAGGCAGATTCCTAGGCATTACTCACCCGTCCGCCGCTCTCAAGAGAAGCAAGCTTCTCTCTACCGCTCGACTGCA

>GW247-E7

TGCAGTCGAACGGTAACAGCACTTCGGTGGCTGACGAGTGGCGAACGGGTGAGTAATACATCGGAACGTGCCCGAGAGTGGGGGATAACGCAGCGAAAGCTGTGCTAATACCGCATACGATCTACGGATGAAAGCAGGGGACCGCAAGGCCTTGCGCTCATGGAGCGGCCGATGGCAGATTAGGTAGTTGGTGGGATAAAAGCTTACCAAGCCGACGATCTGTAGCTGGTCTGAGAGGACGACCAGCCACACTGGGACTGAGACACGGCCCAGACTCCTACGGGAGGCAGCAGTGGGGAATTTTGGACAATGGGCGAAAGCCTGATCCAGCCATGCCGCGTGCAGGATGAAGGCCTTCGGGTTGTAAACTGCTTTTGTACGGAACGAAAAGGTCTTTTCTAATAAAGAAGGCTCATGACGGTACCGTAAGAATAAGCACCGGCTAACTACGTGCCAGCAGCCGCGGTAATACGTAGGGTGCAAGCGTTAATCGGAATTACTGGGCGTAAAGCGTGCGCAGGCGGTTATATAAGACAGATGTGAAATCCCCGGGCTCAACCTGGGAACTGCATTAGTGACTGTATAGCTAGAGTGCGGCAGAGGGGGATGGAATTCCGCGTGTAGCAGTGAAATGCGTAGATATGCGGAGGAACACCGATGGCGAAGGCAATCCCCTGGGCCTGCACTGACGCTCATGCACGAAAGCGTGGGGAGCAAACAGGATTAGATACCCTGGTAGTCCACGCCCTAAACGATGTCAACTGGTTGTTGGGAATTTACTTTCTCAGTAACGAAGCTAACGCGTGAAGTTGACCGCCTGGGGAGTACGGCCGCAAGGTTAAAACTCAAAGGAATTGACGGGGACCCGCACAAGCGGTGGATGATGTGGTTTAATTCGATGCAACGCGNAAAACCTTACCCACCTTTGACATGTATGGAAGATCGCAGAGACGCGATTGTGCTCGAAAGAGAGCCATAACACAGGTGCTGCATGGCTGTCGTCAGCTCGTGTCGTGAGATGTTGGGTTAAGTCCCGCAACGAGCGCAACCCTTGTCATTAGTTGCTACATTTAGTTGGGCACTCTAATGAGACTGCCGGTGACAAACCGGAGGAAGGTGGGGATGACGTCAAGTCCTCATGGCCCTTATAGGTGGGGCTACACACGTCATACAATGGCTGGTACAAAGGGTTGCCAACCCGCGAGGGGGAGCCAATCCCATAAAGCCAGTCGTAGTCCGGATCGCAGTCTGCAACTCGACTGCGTGAAGTCGGAATCGCTAGTAATCGTGGATCAGAATGTCACGGTGAATACGTTCCCGGGTCTTGTACACACCGCCCGTCACACCATGGGAGCGGGTTCTGCCAGAAGTAGGTAGCCTAACCGCAAGGAGGGCGCT

>GW704-E2

TGCAGTCGAGCGGTAGAGAGAAGCTTGCTTCTCTTGAGAGCGGCGGACGGGTGAGTAATGCCTAGGAATCTGCCTGGTAGTGGGGGATAACGTTCGGAAACGGACGCTAATACCGCATACGTCCTACGGGAGAAAGCAGGGGACCTTCGGGCCTTGCGCTATCAGATGAGCCTAGGTCGGATTAGCTAGTTGGTGAGGTAATGGCTCACCAAGGCGACGATCCGTAACTGGTCTGAGAGGATGATCAGTCACACTGGAACTGAGACACGGTCCAGACTCCTACGGGAGGCAGCAGTGGGGAATATTGGACAATGGGCGAAAGCCTGATCCAGCCATGCCGCGTGTGTGAAGAAGGTCTTCGGATTGTAAAGCACTTTAAGTTGGGAGGAANGGCAGTTACCTAATACGTGATTGTTTTGACGTTACCGACAGAATAAGCACCGGCTAACTCTGTGCCAGCAGCCGCGGTAATACAGAGGGTGCAAGCGTTAATCGGAATTACTGGGCGTAAAGCGCGCGTAGGTGGTTAGTTAAGTTGGATGTGAAATCCCCGGGCTCAACCNGGGAACTGCATTCAAAACTGACTGACTAGAGTATGGTAGAGGGTGGTGGAATTTCCTGTGTAGCGGTGAAATGCGTAGATATAGGAAGGAACACCAGTGGCGAAGGCGACCACCTGGACTGATACTGACACTGAGGTGCGAAAGCGTGGGGAGCAAACAGGATTAGATACCCTGGTAGTCCACGCCGTAAACGATGTCAACTAGCCGTTNGGGAGCCTTGAGCTCTTAGTGGCGCAGCTAACGCATTAAGTTGACCGCCTGGGGAGTACGGCCGCAAGGTTAAAACTCAAATGAATTGACGGGGGCCCGCACAAGCGGTGGAGCATGTGGTTTAATTCGAAGCAACGCGAAGAACCTTACCAGGCCTTGACATCCAATGAACTTTCTAGAGATAGATTGGTGCCTTCGGGAACATTGAGACAGGTGCTGCATGGCTGTCGTCAGCTCGTGTCGTGAGATGTTGGGTTAAGTCCCGTAACGAGCGCAACCCTTGTCCTTAGTTACCAGCACGTAATGGTGGGCACTCTAAGGAGACTGCCGGTGACAAACCGGAGGAAGGTGGGGATGACGTCAAGTCATCATGGCCCTTACGGCCTGGGCTACACACGTGCTACAATGGTCGGTACAGAGGGTTGCCAAGCCGCGAGGTGGAGCTAATCCCAGAAAACCGATCGTAGTCCGGATCGCAGTCTGCAACTCGACTGCGTGAAGTCGGAATCGCTAGTAATCGCGAATCAGAATGTCGCGGTGAATACGTTCCCGGGCCTTGTACACACCGCCCGTCACACCATGGGAGTGGGTTGCACCAGAAGTAGCTAGTCTAACCTTCGGGGGGACGGT

>GW704-E3

ACCGTCCCCCCGAAGGTTAGACTAGCTACTTCTGGTGCAACCCACTCCCATGGTGTGACGGGCGGTGTGTACAAGGCCCGGGAACGTATTCACCGCGACATTCTGATTCGCGATTACTAGCGATTCCGACTTCACGCAGTCGAGTTGCAGACTGCGATCCGGACTACGATCGGTTTTCTGGGATTAGCTCCACCTCGCGGCTTGGCAACCCTCTGTACCGACCATTGTAGCACGTGTGTAGCCCAGGCCGTAAGGGCCATGATGACTTGACGTCATCCCCACCTTCCTCCGGTTTGTCACCGGCAGTCTCCTTAGAGTGCCCACCATTACGTGCTGGTAACTAAGGACAAGGGTTGCGCTCGTTACGGGACTTAACCCAACATCTCACGACACGAGCTGACGACAGCCATGCAGCACCTGTCTCAATGTTCCCGAAGGCACCAATCTATCTCTAGAAAGTTCATTGGATGTCAAGGCCTGGTAAGGTTCTTCGCGTTGCTTCGAATTAAACCACATGCTCCACCGCTTGTGCGGGCCCCCGTCAATTCATTTGAGTTTTAACCTTGCGGCCGTACTCCCCAGGCGGTCAACTTAATGCGTTAGCTGCGCCACTAAGAGCTCAAGGCTCCCAACGGCTAGTTGACATCGTTTACGGCGTGGACTACCAGGGTATCTAATCCTGTTTGCTCCCCACGCTTTCGCACCTCAGTGTCAGTATCAGTCCAGGTGGTCGCCTTCGCCACTGGTGTTCCTTCCTATATCTACGCATTTCACCGCTACACAGGAAATTCCACCACCCTCTACCATACTCTAGTCAGTCAGTTTTGAATGCAGTTCCCAGGTTGAGCCCGGGGATTTCACATCCAACTTAACTAACCACCTACGCGCGCTTTACGCCCAGTAATTCCGATTAACGCTTGCACCCTCTGTATTACCGCGGCTGCTGGCACAGAGTTAGCCGGTGCTTATTCTGTCGGTAACGTCAAAACAATCACGTATTAGGTAACNGCCNTTCCTCCCAACTTAAAGTGCTTTACAATCCGAAGACCTTCTTCACACACGCGGCATGGCTGGATCAGGCTTTCGCCCATTGTCCAATATTCCCCACTGCTGCCTCCCGTAGGAGTCTGGACCGTGTCTCAGTTCCAGTGTGACTGATCATCCTCTCAGACCAGTTACGGATCGTCGCCTTGGTGAGCCATTACCTCACCAACTAGCTAATCCGACCTAGGCTCATCTGATAGCGCAAGGCCCGAAGGTCCCCTGCTTTCTCCCGTAGGACGTATGCGGTATTAGCGTCCGTTTCCGAACGTTATCCCCCACTACCAGGCAGATTCCTAGGCATTACTCACCCGTCCGCCGCTCTCAAGAGAAGCAAGCTTCTCTCTACCGCTCGACTGCA

>GW704-F2

CCCCGAAGGTTAGACTAGCTACTTCTGGTGCAACCCACTCCCATGGTGTGACGGGCGGTGTGTACAAGGCCCGGGAACGTATTCACCGCGACATTCTGATTCGCGATTACTAGCGATTCCGACTTCACGCAGTCGAGTTGCAGACTGCGATCCGGACTACGATCGGTTTTCTGGGATTAGCTCCACCTCGCGGCTTGGCAACCCTCTGTACCGACCATTGTAGCACGTGTGTAGCCCAGGCCGTAAGGGCCATGATGACTTGACGTCATCCCCACCTTCCTCCGGTTTGTCACCGGCAGTCTCCTTAGAGTGCCCACCATTACGTGCTGGTAACTAAGGACAAGGGTTGCGCTCGTTACGGGACTTAACCCAACATCTCACGACACGAGCTGACGACAGCCATGCAGCACCTGTCTCAATGTTCCCGAAGGCACCAATCTATCTCTAGAAAGTTCATTGGATGTCAAGGCCTGGTAAGGTTCTTCGCGTTGCTTCGAATTAAACCACATGCTCCACCGCTTGTGCGGGCCCCCGTCAATTCATTTGAGTTTTAACCTTGCGGCCGTACTCCCCAGGCGGTCAACTTAATGCGTTAGCTGCGCCACTAAGAGCTCAAGGCTCCCAACGGCTAGTTGACATCGTTTACGGCGTGGACTACCAGGGTATCTAATCCTGTTTGCTCCCCACGCTTTCGCACCTCAGTGTCAGTATCAGTCCAGGTGGTCGCCTTCGCCACTGGTGTTCCTTCCTATATCTACGCATTTCACCGCTACACAGGAAATTCCACCACCCTCTACCATACTCTAGTCAGTCAGTTTTGAATGCAGTTCCCAGGTTGAGCCCGGGGATTTCACATCCAACTTAACTAACCACCTACGCGCGCTTTACGCCCAGTAATTCCGATTAACGCTTGCACCCTCTGTATTACCGCGGCTGCTGGCACAGAGTTAGCCGGTGCTTATTCTGTCGGTAACGTCAAAACAATCACGTATTAGGTAACTNCCNTTCCTCCCAACTTAAAGTGCTTTACAATCCGAAGACCTTCTTCACACACGCGGCATGGCTGGATCAGGCTTTCGCCCATTGTCCAATATTCCCCACTGCTGCCTCCCGTAGGAGTCTGGACCGTGTCTCAGTTCCAGTGTGACTGATCATCCTCTCAGACCAGTTACGGATCGTCGCCTTGGTGAGCCATTACCTCACCAACTAGCTAATCCGACCTAGGCTCATCTGATAGCGCAAGGCCCGAAGGTCCCCTGCTTTCTCCCGTAGGACGTATGCGGTATTAGCGTCCGTTTCCGAACGTTATCCCCCACTACCAGGCAGATTCCTAGGCATTACTCACCCGTCCGCCGCTCTCAAGAGAAGCAAGCTTCTCTCTACCGCTCGACTGCA

>GW247-E2

TGCAGTCGAACGGTAACAGCACTTCGGTGGCTGACGAGTGGCGAACGGGTGAGTAATACATCGGAACGTGCCCGAGAGTGGGGGATAACGCAGCGAAAGCTGTGCTAATACCGCATACGATCTACGGATGAAAGCAGGGGACCGCAAGGCCTTGCGCTCATGGAGCGGCCGATGGCAGATTAGGTAGTTGGTGGGATAAAAGCTTACCAAGCCGACGATCTGTAGCTGGTCTGAGAGGACGACCAGCCACACTGGGACTGAGACACGGCCCAGACTCCTACGGGAGGCAGCAGTGGGGAATTTTGGACAATGGGCGAAAGCCTGATCCAGCCATGCCGCGTGCAGGATGAAGGCCTTCGGGTTGTAAACTGCTTTTGTACGGAACGAAAAGGTCTTTTCTAATAAAGAAGGCTCATGACGGTACCGTAAGAATAAGCACCGGCTAACTACGTGCCAGCAGCCGCGGTAATACGTAGGGTGCAAGCGTTAATCGGAATTACTGGGCGTAAAGCGTGCGCAGGCGGTTATATAAGACAGATGTGAAATCCCCGGGCTCAACCTGGGAACTGCATTAGTGACTGTATAGCTGGAGTGCGGCAGAGGGGGATGGAATTCCGCGTGTAGCAGTGAAATGCGTAGATATGCGGAGGAACACCGATGGCGAAGGCAATCCCCTGGGCCTGCACTGACGCTCATGCACGAAAGCGTGGGGAGCAAACAGGATTAGATACCCTGGTAGTCCACGCCCTAAACGATGTCAACTGGTTGTTGGGAATTTACTTTCTCAGTAACGAAGCTAACGCGTGAAGTTGACCGCCTGGGGAGTACGGCCGCAAGGTTAAAACTCAAAGGAATTGACGGGGACCCGCACAAGCGGTGGATGATGTGGTTTAATTCGATGCAACGCGAAAAACCTTACCCACCTTTGACATGTATGGAAGATCGCAGAGACGCGATTGTGCTCGAAAGAGAGCCATAACACAGGTGCTGCATGGCTGTCGTCAGCTCGTGTCGTGAGATGTTGGGTTAAGTCCCGCAACGAGCGCAACCCTTGTCATTAGTTGCTACATTTAGTTGGGCACTCTAATGAGACTGCCGGTGACAAACCGGAGGAAGGTGGGGATGACGTCAAGTCCTCATGGCCCTTATAGGTGGGGCTACACACGTCATACAATGGCTGGTACAAAGGGTTGCCAACCCGCGAGGGGGAGCCAATCCCATAAAGCCAGTCGTAGTCCGGATCGTAGTCTGCAACTCGACTACGTGAAGTCGGAATCGCTAGTAATCGTGGATCAGAATGTCACGGTGAATACGTTCCCGGGTCTTGTACACACCGCCCGTCACACCATGGGAGCGGGTTCTGCCAGAAGTAGGTAGCCTAACCGTAAGGAGGGCGCT

>GW247-E3

AGCGCCCTCCTTACGGTTAGGCTACCTACTTCTGGCAGAACCCGCTCCCATGGTGTGACGGGCGGTGTGTACAAGACCCGGGAACGTATTCACCGTGACATTCTGATCCACGATTACTAGCGATTCCGACTTCACGTAGTCGAGTTGCAGACTACGATCCGGACTACGACTGGCTTTATGGGATTGGCTCCCCCTCGCGGGTTGGCAACCCTTTGTACCAGCCATTGTATGACGTGTGTAGCCCCACCTATAAGGGCCATGAGGACTTGACGTCATCCCCACCTTCCTCCGGTTTGTCACCGGCAGTCTCATTAGAGTGCCCAACTAAATGTAGCAACTAATGACAAGGGTTGCGCTCGTTGCGGGACTTAACCCAACATCTCACGACACGAGCTGACGACAGCCATGCAGCACCTGTGTTATGGCTCTCTTTCGAGCACAATCGCGTCTCTGCGATCTTCCATACATGTCAAAGGTGGGTAAGGTTTTTCGCGTTGCATCGAATTAAACCACATCATCCACCGCTTGTGCGGGTCCCCGTCAATTCCTTTGAGTTTTAACCTTGCGGCCGTACTCCCCAGGCGGTCAACTTCACGCGTTAGCTTCGTTACTGAGAAAGTAAATTCCCAACAACCAGTTGACATCGTTTAGGGCGTGGACTACCAGGGTATCTAATCCTGTTTGCTCCCCACGCTTTCGTGCATGAGCGTCAGTGCAGGCCCAGGGGATTGCCTTCGCCATCGGTGTTCCTCCGCATATCTACGCATTTCACTGCTACACGCGGAATTCCATCCCCCTCTGCCGCACTCCAGCTATACAGTCACTAATGCAGTTCCCAGGTTGAGCCCGGGGATTTCACATCTGTCTTATATAACCGCCTGCGCACGCTTTACGCCCAGTAATTCCGATTAACGCTTGCACCCTACGTATTACCGCGGCTGCTGGCACGTAGTTAGCCGGTGCTTATTCTTACGGTACCGTCATGAGCCTTCTTTATTAGAAAAGACCTTTTCGTTCCGTACAAAAGCAGTTTACAACCCGAAGGCCTTCATCCTGCACGCGGCATGGCTGGATCAGGCTTTCGCCCATTGTCCAAAATTCCCCACTGCTGCCTCCCGTAGGAGTCTGGGCCGTGTCTCAGTCCCAGTGTGGCTGGTCGTCCTCTCAGACCAGCTACAGATCGTCGGCTTGGTAAGCTTTTATCCCACCAACTACCTAATCTGCCATCGGCCGCTCCATGAGCGCAAGGCCTTGCGGTCCCCTGCTTTCATCCGTAGATCGTATGCGGTATTAGCACAGCTTTCGCTGCGTTATCCCCCACTCTCGGGCACGTTCCGATGTATTACTCACCCGTTCGCCACTCGTCAGCCACCGAAGTGCTGTTACCGTTCGACTGCA

>FW104-L2

GAAGGGAGCTTGCTCCCGGATGTTAGCGGCGGACGGGTGAGTAACACGTGGGTAACCTGCCTGTAAGACTGGGATAACTCCGGGAAACCGGAGCTAATACCGGATAGTTCCTTGAACCGCATGGTTCAAGGATGAAAGACGGTTTCGGCTGTCACTTACAGATGGACCCGCGGCGCATTAGCTAGTTGGTGGGGTAATGGCTCACCAAGGCGACGATGCGTAGCCGACCTGAGAGGGTGATCGGCCACACTGGGACTGAGACACGGCCCAGACTCCTACGGGAGGCAGCAGTAGGGAATCTTCCGCAATGGACGAAAGTCTGACGGAGCAACGCCGCGTGAGTGATGAAGGTTTTCGGATCGTAAAGCTCTGTTGTTAGGGAAGAACAAGTGCGAGAGTAACTGCTCGCACCTTGACGGTACCTAACCAGAAAGCCACGGCTAACTACGTGCCAGCAGCCGCGGTAATACGTAGGTGGCAAGCGTTGTCCGGAATTATTGGGCGTAAAGGGCTCGCAGGCGGTTTCTTAAGTCTGATGTGAAAGCCCCCGGCTCAACCGGGGAGGGTCATTGGAAACTGGGAAACTTGAGTGCAGAAGAGGAGAGTGGAATTCCACGTGTAGCGGTGAAATGCGTAGAGATGTGGAGGAACACCAGTGGCGAAGGCGACTCTCTGGTCTGTAACTGACGCTGAGGAGCGAAAGCGTGGGGAGCGAACAGGATTAGATACCCTGGTAGTCCACGCCGTAAACGATGAGTGCTAAGTGTTAGGGGGTTTCCGCCCCTTAGTGCTGCAGCTAACGCATTAAGCACTCCGCCTGGGGAGTACGGTCGCAAGACTGAAACTCAAAGGAATTGACGGGGGCCCGCACAAGCGGTGGAGCATGTGGTTTAATTCGAAGCAACGCGAAGAACCTTACCAGGTCTTGACATCCTCTGACAACCCTAGAGATAGGGCTTTCCCTTCGGGGACAGAGTGACAGGTGGTGCATGGTTGTCGTCAGCTCGTGTCGTGAGATGTTGGGTTAAGTCCCGCAACGAGCGCAACCCTTGATCTTAGTTGCCAGCATTCAGTTGGGCACTCTAAGGTGACTGCCGGTGACAAACCGGAGGAAGGTGGGGATGACGTCAAATCATCATGCCCCTTATGACCTGGGCTACMMMCGTGCTACAATGGACAGAACAAAGGGCTGCAAGACCGCAAGGTTTAGCCAATCCCATAAATCTGTTCTCAGTTCGGATCGCAGTCTGCAACTCGACTGCGTGAAGCTGGAATCGCTAGTAATCGCGGATCAGCATGCCGCGGTGAATACGTTCCCGGGCCTTGTACACACCGCCCGTCACACCACGAGAGTTTGCAACACCCGAAGTCGGTGAGGTAACCTTTA

>FW104-L5

TGGGTGGCGAGTGGCGGACGGGTGAGTAATGCATCGGGATCTACCCTGACGTGGGGGATAACCTCGGGAAACCGGGACTAATACCGCATACGTCCTACGGGAGAAAGCGGGGGACCTTCGGGCCTCGCGCGGCAGGACGAACCGATGTGCGATTAGCTAGTTGGCGGGGTAATGGCCCACCAAGGCGACGATCGCTAGCTGGTCTGAGAGGATGATCAGCCACACTGGGACTGAGACACGGCCCAGACTCCTACGGGAGGCAGCAGTGGGGAATATTGGACNAATGGGCGCAAGCCTGATCCAGCAATGCCGCGTGTGTGAAGAAGGCCTTCGGGTTGTAAAGCACTTTTATCAGGAGCGAAATACCACGGGTTAATACCCTATGGGGCTGACGGTACCTGAGGAATAAGCACCGGCTAACTTCGTGCCAGCAGCCGCGGTAATACGAAGGGTGCAAGCGTTAATCGGAATTACTGGGCGTAAAGGGTGCGTAGGCGGTTACTTAAGTCTGTCGTGAAATCCCCGGGCTCAACCTGGGAATGGCGATGGATACTGGGTGGCTAGAGTGTGTCAGAGGATGGTGGAATTCCCGGTGTAGCGGTGAAATGCGTAGAGATCGGGAGGAACATCAGTGGCGAAGGCGGCCATCTGGGACAACACTGACGCTGAAGCACGAAAGCGTGGGGAGCAAACAGGATTAGATACCCTGGTAGTCCACGCCCTAAACGATGCGAACTGGATGTTGGTCTCAACTCGGAGATCAGTGTCGAAGCTAACGCGTTAAGTTCGCCGCCTGGGGAGTACGGTCGCAAGACTGAAACTCAAAGGAATTGACGGGGGCCCGCACAAGCGGTGGAGTATGTGGTTTAATTCGATGCAACGCGAAGAACCTTACCTGGCCTTGACATGTCCGGAATCCTGCAGAGATGCGGGAGTGCCTTCGGGAATCGGAACACAGGTGCTGCATGGCTGTCGTCAGCTCGTGTCGTGAGATGTTGGGTTAAGTCCCGCAACGAGCGCAACCCTTGTCCTTAGTTGCCAGCACGTAATGGTGGGAACTCTAAGGAGACTGCCGGTGACAAACCGGAGGAAGGTGGGGATGACGTCAAGTCATCATGGCCCTTACGGCNAGGGCTACMCACGTACTACAATGGTCGGTACAGAGGGTTGCAATACCGCGAGGTGGAGCCAATCCCAGAAAGCCGATCCCAGTCCGGATTGGAGTCTGCAACTCGACTCCATGAAGTCGGAATCGCTAGTAATCGCGGATCAGCTATGCCGCGGTGAATACGTTCCCGGGCCTTGTACACACCGCCCGTCACACCATGGGAGTGGGTTGCTCCAGAAGGCGTTAGTCTAACCGCAAGGGGGACGA

>FW104-R4

TGCAGTCGAACGATGATCCCAGCTTGCTGGGGGATTAGTGGCGAACGGGTGAGTAACACGTGAGTAACCTGCCCTTGACTCTGGGATAAGCCTGGGAAACTGGGTCTAATACCGGATACGACCATTCCACGCATGTGGTGGTGGTGGAAAGCTTTTGTGGTTTTGGATGGACTCGCGGCCTATCAGCTTGTTGGTGGGGTAATGGCCTACCAAGGCGACGACGGGTAGCCGGCCTGAGAGGGTGACCGGCCACACTGGGACTGAGACACGGCCCAGACTCCTACGGGAGGCAGCAGTGGGGAATATTGCACAATGGGCGCAAGCCTGATGCAGCGACGCCGCGTGAGGGATGACGGCCTTCGGGTTGTAAACCTCTTTCAGTAGGGAAGAAGCGAAAGTGACGGTACCTGCAGAAGAAGCGCCGGCTAACTACGTGCCAGCAGCCGCGGTAATACGTAGGGCGCAAGCGTTATCCGGAATTATTGGGCGTAAAGAGCTCGTAGGCGGTTTGTCGCGTCTGCTGTGAAAGACCGGGGCTCAACTCCGGTTCTGCAGTGGGTACGGGCAGACTAGAGTGCAGTAGGGGAGACTGGAATTCCTGGTGTAGCGGTGAAATGCGCAGATATCAGGAGGAACACCGATGGCGAAGGCAGGTCTCTGGGCTGTAACTGACGCTGAGGAGCGAAAGCATGGGGAGCGAACAGGATTAGATACCCTGGTAGTCCATGCCGTAAACGTTGGGCACTAGGTGTGGGGGACATTCCACGTTTTCCGCGCCGTAGCTAACGCATTAAGTGCCCCGCCTGGGGAGTACGGCCGCAAGGCTAAAACTCAAAGGAATTGACGGGGGCCCGCACAAGCGGCGGAGCATGCGGATTAATTCGATGCAACGCGAAGAACCTTACCAAGGCTTGACATGAACCGGTAATACCTGGAAACAGGTGCCCCGCTTGCGGTCGGTTTACAGGTGGTGCATGGTTGTCGTCAGCTCGTGTCGTGAGATGTTGGGTTAAGTCCCGCAACGAGCGCAACCCTCGTTCTATGTTGCCAGCGGTTCGGCCGGGGACTCATAGGAGACTGCCGGGGTCAACTCGGAGGAAGGTGGGGACGACGTCAAATCATCATGCCCCTTATGTCTTGGGCTTCACGCATGCTACAATGGCCGGTACAAAGGGTTGCGATACTGTGAGGTGGAGCTAATCCCAAAAAGCCGGTCTCAGTTCGGATTGGGGTCTGCAACTCGACCCCATGAAGTCGGAGTCGCTAGTAATCGCAGATCAGCAACGCTGCGGTGAATACGTTCCCGGGCCTTGTACACACCGCCCGTCAAGTCACGAAAGTTGGTAACACCCGAAGCCGGT

>FW104-R5

TGGGTGGCGAGTGGCGGACGGGTGAGTAATGCATCGGGATCTACCCTGACGTGGGGGATAACCTCGGGAAACCGGGACTAATACCGCATACGTCCTACGGGAGAAAGCGGGGGACCTTCGGGCCTCGCGCGGCAGGACGAACCGATGTGCGATTAGCTAGTTGGCGGGGTAATGGCCCACCAAGGCGACGATCGCTAGCTGGTCTGAGAGGATGATCAGCCACACTGGGACTGAGACACGGCCCAGACTCCTACGGGAGGCAGCAGTGGGGAATATTGGMCAATGGGCGCAAGCCTGATCCAGCAATGCCGCGTGTGTGAAGAAGGCCTTCGGGTTGTAAAGCACTTTTATCAGGAGCGAAATACCACGGGTTAATACCCTATGGGGCTGACGGTACCTGAGGAATAAGCACCGGCTAACTTCGTGCCAGCAGCCGCGGTAATACGAAGGGTGCAAGCGTTAATCGGAATTACTGGGCGTAAAGGGTGCGTAGGCGGTTACTTAAGTCTGTCGTGAAATCCCCGGGCTCAACCTGGGAATGGCGATGGATACTGGGTGGCTAGAGTGTGTCAGAGGATGGTGGAATTCCCGGTGTAGCGGTGAAATGCGTAGAGATCGGGAGGAACATCAGTGGCGAAGGCGGCCATCTGGGACAACACTGACGCTGAAGCACGAAAGCGTGGGGAGCAAACAGGATTAGATACCCTGGTAGTCCACGCCCTAAACGATGCGAACTGGATGTTGGTCTCAACTCGGAGATCAGTGTCGAAGCTAACGCGTTAAGTTCGCCGCCTGGGGAGTACGGTCGCAAGACTGAAACTCAAAGGAATTGACGGGGGCCCGCACAAGCGGTGGAGTATGTGGTTTAATTCGATGCAACGCGAAGAACCTTACCTGGCCTTGACATGTCCGGAATCCTGCAGAGATGCGGGAGTGCCTTCGGGAATCGGAACACAGGTGCTGCATGGCTGTCGTCAGCTCGTGTCGTGAGATGTTGGGTTAAGTCCCGCAACGAGCGCAACCCTTGTCCTTAGTTGCCAGCACGTAATGGTGGGAACTCTAAGGARACTGCCGGTGACAAACCGGAGGAAGGTGGGGATGACGTCAAGTCATCATGGCCCTTACGGCCAGGGCTACACACGTACTACAATGGTCGGTACAGAGGGTTGCAATACCGCGAGGTGGAGCCAATCCCAGAAAGCCGATCCCAGTCCGGATTGGAGTCTGCAACTCGACTCCATGAAGTCGGAATCGCTAGTAATCGCGGATCAGCTATGCCGCGGTGAATACGTTCCCGGGCCTTGTACACACCGCCCGTCACACCATGGGAGTGGGTTGCTCCAGAAGGCGTTAGTCTAACCGCAAGGG

>FW104-R8

TGCAGTCGAACGGCAGCACAGCCGTAGCAATACGGTGGGTGGCGAGTGGCGGACGGGTGAGTAAAGCATCGGGACCTACCCAGACGTGGGGGATAACCTCGGGAAACCGGGACTAATACCGCATACGTCCTACGGGAGAAAGCGGGGGACCTTCGGGCCTCGCGCGGTTGGACGGACCGATGTGCGATTAGCTAGTTGGTAGGGTAATGGCCTACCAAGGCGACGATCGCTAGCTGGTCTGAGAGGATGATCAGCCACACTGGGACTGAGACACGGCCCAGAYTCCTACGGGAGGCAGCAGTGGGGAATATTGGMCAATGGGCGCAAGCCTGATCCAGCAATGCCGCGTGTGTGAAGAAGGCCTTCGGGTTGTAAAGCACTTTTATCAGGAGCGAAATACCACGGGTCAATACCCTGTGGGGCTGACGGTACCTGAGGAATAAGCACCGGCTAACTTCGTGCCAGCAGCCGCGGTAATACGAAGGGTGCAAGCGTTAATCGGAATTACTGGGCGTAAAGGGTGCGTAGGCGGTTACTTAAGTCTGTCGTGAAATCCCCGGGCTCAACCTGGGAATGGCGATGGATACTGGGTGGCTAGAGTGTGTCAGAGGATGGTGGAATTCCCGGTGTAGCGGTGAAATGCGTAGAGATCGGGAGGAACATCAGTGGCGAAGGCGGCCATCTGGGACAACACTGACGCTGAAGCACGAAAGCGTGGGGAGCAAACAGGATTAGATACCCTGGTAGTCCACGCCCTAAACGATGCGAACTGGATGTTGGTCTCAACTCGGAGATCAGTGTCGAAGCTAACGCGTTAAGTTCGCCGCCTGGGGAGTACGGTCGCAAGACTGAAACTCAAAGGAATTGACGGGGGCCCGCACAAGCGGTGGAGTATGTGGTTTAATTCGATGCAACGCGAAGAACCTTACCTGGCCTTGACATGTCCGGAATCCTGCAGAGATGCGGGAGTGCCTTCGGGAATCGGAACACAGGTGCTGCATGGCTGTCGTCAGCTCGTGTCGTGAGATGTTGGGTTAAGTCCCGCAACGAGCGCAACCCTTGTCCTTAGTTGCCAGCACGTAATGGTGGGAACTCTAAGGAGACTGCCGGTGACAAACCGGAGGAAGGTGGGGATGACGTCAAGTCATCATGGCCCTTACGGCCAGGGCTACMCACGTACTACAATGGTCGGTACAGAGGGTTGCAAGACCGCGAGGTGGAGCCAATCCCAGAAAGCCGATCCCAGTCCGGATTGGAGTCTGCAACTCGACTCCATGAAGTCGGAATCGCTAGTAATCGCGGATCAGCTATGCCGCGGTGAATACGTTCCCGGGCCTTGTACACACCGCCCGTCACACCATGGGAGTGGGTTGCTCCAGAAGGCGTTAGTCTAACCGCAAGG

>FW104-T1

AGTCGAGCGGACAGAAGGGAGCTTGCTCCCGGATGTTAGCGGCGGACGGGTGAGTAACACGTGGGTAACCTGCCTGTAAGACTGGGATAACTCCGGGAAACCGGAGCTAATACCGGATAGTTCCTTGAACCGCATGGTTCAAGGATGAAAGACGGTTTCGGCTGTCACTTACAGATGGACCCGCGGCGCATTAGCTAGTTGGTGGGGTAATGGCTCACCAAGGCGACGATGCGTAGCCGACCTGAGAGGGTGATCGGCCACACTGGGACTGAGACACGGCCCAGAYTCCTACGGGAGGCAGCAGTAGGGAATCTTCCGCAATGGACGAAAGTCTGACGGAGCAACGCCGCGTGAGTGATGAAGGTTTTCGGATCGTAAAGCTCTGTTGTTAGGGAAGAACAAGTGCGAGAGTAACTGCTCGCACCTTGACGGTACCTAACCAGAAAGCCACGGCTAACTACGTGCCAGCAGCCGCGGTAATACGTAGGTGGCAAGCGTTGTCCGGAATTATTGGGCGTAAAGGGCTCGCAGGCGGTTTCTTAAGTCTGATGTGAAAGCCCCCGGCTCAACCGGGGAGGGTCATTGGAAACTGGGAAACTTGAGTGCAGAAGAGGAGAGTGGAATTCCACGTGTAGCGGTGAAATGCGTAGAGATGTGGAGGAACACCAGTGGCGAAGGCGACTCTCTGGTCTGTAACTGACGCTGAGGAGCGAAAGCGTGGGGAGCGAACAGGATTAGATACCCTGGTAGTCCACGCCGTAAACGATGAGTGCTAAGTGTTAGGGGGTTTCCGCCCCTTAGTGCTGCAGCTAACGCATTAAGCACTCCGCCTGGGGAGTACGGTCGCAAGACTGAAACTCAAAGGAATTGACGGGGGCCCGCACAAGCGGTGGAGCATGTGGTTTAATTCGAAGCAACGCGAAGAACCTTACCAGGTCTTGACATCCTCTGACAACCCTAGAGATAGGGCTTTCCCTTCGGGGACAGAGTGACAGGTGGTGCATGGTTGTCGTCAGCTCGTGTCGTGAGATGTTGGGTTAAGTCCCGCAACGAGCGCAACCCTTGATCTTAGTTGCCAGCATTCAGTTGGGCACTCTAAGGTGACTGCCGGTGACAAACCGGAGGAAGGTGGGGATGACGTCAAATCATCATGCCCCTTATGACCTGGGCTACACACGTGCTACAATGGACAGAACAAAGGGCTGCAAGACCGCAAGGTTTAGCCAATCCCATAAATCTGTTCTCAGTTCGGATCGCAGTCTGCAACTCGACTGCGTGAAGCTGGAATCGCTAGTAATCGCGGATCAGCATGCCGCGGTGAATACGTTCCCGGGCCTTGTACACACCGCCCGTCACACCACGAGAGTTTGCAACACCCGAAGTCGGTGAGGTAACCTTTATGGAGCCAGCCGCCGAAGG

>FW104-T2

TGCAGTCGAGCGGACTTGATGAGAAGCTTGCTTCTCTGATGGTTAGCGGCGGACGGGTGAGTAACACGTAGGCAACCTGCCCTCAAGCTTGGGACAACTACCGGAAACGGTAGCTAATACCGAATATGTGCTTTCTTCGCCTGAAGGAAGCTGGAAAGACGGAGCAATCTGTCACTTGAGGATGGGCCTGCGGCGCATTAGCTAGTTGGTGAGGTAACGGCTCACCAAGGCGACGATGCGTAGCCGACCTGAGAGGGTGATCGGCCACACTGGGACTGAGACACGGCCCAGACTCCTACGGGAGGCAGCAGTAGGGAATCTTCCGCAATGGGCGAAAGCCTGACGGAGCAATGCCGCGTGAGTGATGAAGGTTTTCGGATCGTAAAGCTCTGTTGCCAGGGAAGAACGTCCTTGAGAGTAACTGCTCAAGGAGTGACGGTACCTGAGAAGAAAGCCCCGGCTAACTACGTGCCAGCAGCCGCGGTAATACGTAGGGGGCAAGCGTTGTCCGGAATTATTGGGCGTAAAGCGCGCGCAGGCGGTCATTTAAGTCTGGTGTTTAATCCCGGGGCTCAACCCCGGATCGCACTGGAAACTGGATGACTTGAGTGCAGAAGAGGAGAGTGGAATTCCACGTGTAGCGGTGAAATGCGTAGAGATGTGGAGGAACACCAGTGGCGAAGGCGACTCTCTGGGCTGTAACTGACGCTGAGGCGCGAAAGCGTGGGGAGCAAACAGGATTAGATACCCTGGTAGTCCACGCCGTAAACGATGAATGCTAGGTGTTAGGGGTTTCGATACCCTTGGTGCCGAAGTTAACACATTAAGCATTCCGCCTGGGGAGTACGGTCGCAAGACTGAAACTCAAAGGAATTGACGGGGACCCGCACAAGCAGTGGAGTATGTGGTTTAATTCGAAGCAACGCGAAGAACCTTACCAGGTCTTGACATCCGWMTGACCGKCGTAGAGATRTGSCTTTCCTTCGGGACAKACGAGACAGGTGGTGCATGGTTGTCGTCAGCTCGTGTCGTGAGATGTTGGGTTAAGTCCCGCAACGAGCGCAACCCTTGATCTTAGTTGCCAGCAYTTCGGGTGGGCACTCTAAGGTGACTGCCGGTGACAAACCGGAGGAAGGTGGGGATGACGTCAAATCATCATGCCCCTTATGACCTGGGCTACACACGTACTACAATGGCCGGTACAACGGGCAGTGAAGCCGCGAGGTGGAACGAATCCTAAAAAGCCGGTCTCAGTTCGGATTGCAGGCTGCAACTCGCCTGCATGAAGTCGGAATTGCTAGTAATCGCGGATCAGCATGCCGCGGTGAATACGTTCCCGGGTCTTGTACACACCGCCCGTCACACCACGAGAGTTTATAACACCCGAAGTCGGTGGGGTAACCGCAAGGAGCCAGCC

>FW104-T7

AGTCGAACGGCAGCACAGTCGTAGCAATACGATGGGTGGCGAGTGGCGGACGGGTGAGTAATGCATCGGGATCTACCCTGACGTGGGGGATAACCTCGGGAAACCGGGACTAATACCGCATACGTCCTACGGGAGAAAGCGGGGGACCTTCGGGCCTCGCGCGGCAGGACGAACCGATGTGCGATTAGCTAGTTGGCGGGGTAATGGCCCACCAAGGCGACGATCGCTAGCTGGTCTGAGAGGATGATCAGCCACACTGGGACTGAGACACGGCCCAGACTCCTACGGGAGGCAGCAGTGGGGAATATTGGMCAATGGGCGCAAGCCTGATCCAGCAATGCCGCGTGTGTGAAGAAGGCCTTCGGGTTGTAAAGCACTTTTATCAGGAGCGAAATACCACGGGTTAATACCCTATGGGGCTGACGGTACCTGAGGAATAAGCACCGGCTAACTTCGTGCCAGCAGCCGCGGTAATACGAAGGGTGCAAGCGTTAATCGGAATTACTGGGCGTAAAGGGTGCGTAGGCGGTTGTTTAAGTCTGCCGTGAAATCCCCGGGCTCAACCTGGGAATGGCGGTGGATACTGGGCAGCTAGAGTGTGTCAGAGGATGGTGGAATTCCCGGTGTAGCGGTGAAATGCGTAGAGATCGGGAGGAACATCAGTGGCGAAGGCGGCCATCTGGGACAACACTGACGCTGAAGCACGAAAGCGTGGGGAGCAAACAGGATTAGATACCCTGGTAGTCCACGCCCTAAACGATGCGAACTGGATGTTGGTCTCAACTCGGAGATCAGTGTCGAAGCTAACGCGTTAAGTTCGCCGCCTGGGGAGTACGGTCGCAAGACTGAAACTCAAAGGAATTGACGGGGGCCCGCACAAGCGGTGGAGTATGTGGTTTAATTCGATGCAACGCGAAGAACCTTACCTGGCCTTGACATGTCCGGAATCCTGCAGAGATGTGGGAGTGCCTTCGGGAATCGGAACACAGGTGCTGCATGGCTGTCGTCAGCTCGTGTCGTGAGATGTTGGGTTAAGTCCCGCAACGAGCGCAACCCTTGTCCTTAGTTGCCAGCACGTAATGGTGGNAACTCTAAGGAGACTGCCGGTGACAAACCGGAGGAAGGTGGGGATGACGTCAAGTCATCATGGCCCTTACGGCCAGGGCTACACACGTACTACAATGGTCGGTACAGAGGGTTGCCATACCGCGAGGTGGAGCCAATCCCAGAAAGCCGATCCCAGTCCGGATTGGAGTCTGCAACTCGACTCCATGAAGTCGGAATCGCTAGTAATCGCGGATCAGCTATGCCGCGGTGAATACGTTCCCGGGCCTTGTACACACCGCCCGTCACACCATGGGAGTGGGTTGCTCCAGAAGGCGTTAGTCTAACCGCAAGG

>GW247-B1

NNNNNNNNNNNNNNNNNNNNNNNNNNNCNCNNGGNTTGGGCNNNNNNNTTCNGGNGTNACCNACTTNNNNGACTTGANNGGNNGTGTGNANNANGNNNNGGAACGTANTCNCCNCNNCNNTGCTGATCTGCNATTACTANCGACTCCNACTTCNTGGGGTCNAGTTGCANACCCCAATCCGAACTGAGACCGGNTTTTTGGGATTCNCTCCNCCTCACGGCTTCNCAACCCTTTGTACCGGNCNTTGTANCATGNTTGAANCCCAAGANATAANGGGCATGANGANNTGACNTCNTCCCCNCCTTCCTCCNAGTTGACCCCGGNAGTCTCCTATGANTCCCCNCCATCACGTGCTGGCAACNNANAACGANGGTTGCGCTCGTTGCGGGACTTAACCCAACATCTCACGACACGAGCTGACGACAACCATGCACCACCTGTACACCAGTCCSAAGAAGCACCCNTCTCTGGATGTGTCCGGTGTATGTCAAGCCTTGGTAAGGTTCTTCGCGTTGCATCGAATTAATCCGCATGCTCCGCCGCTTGYGCGNGCCCCCGTCAATTCCTTTGAGTTTTAGCCTTGCGGCCGTACTCCCCAGGCGGGGCGCTTAATGCGTTARCTGCGGCACGGAACTCGTGGAATGAGCCCCACACCTAGCGCCCAACGTTTACGGCATGGACTACCAGGGTATCTAATCCTGTTCGCTCCCCATGCTTTCGCTTCTCAGCGTCAGTANGTGGCCCAGAGACCTGCCTTCGCCATCGGTGTTCCTCCTGATATCTGNGCATTCCACCGCTACACCAGGAATTCCAGTCTCYCCTACCACACTCTAGTCTGCCCGTACCCACTGCAAGTCCGGGGTTGAGCCCCGGATTTTCACAGCAGANGCGACAAACCGCCTACAAGCTCTTTACGCCCAATAATTCCGGACAACGYTCGCACCCTACGTATTACCGCGRCTGCTGGCACGTAGTTAGCCGGTGCTTCTTNTGCAGGTACCGTCACTTTCGCTTCTTCCCKGCTGAAAGAGGTTWACAACCCGAAGGCCGTCMTCCCTCACGCGGCGTCGCTGCATCAGGCTTCCGCCCATTGTGCAATATTCCCCACTGCTGCCTCCCGTAGGAGTCTGGGCCGTGTCTCAGTCCCAGTGTGGCCGGTCGCCCTCTCAGGCCGGCTACCCGTCGTCGCCTTGGTGAGCCACTACCTCACCAACAAGCTGATAGGCCGCGAGCCCATCCCAGACCGAAAAACTTTCCACCCCCAGCACATGCGTGCAGAGGTCATATCCGGTATTAGCTCCGGTTTCCCGGAGTTATCCCAGAGTCTGGGGCAGGTTACTCACGTGTTACTCACCCGTTCGCCACTGATCAGACAGGGCAAGCCCCATCATCACCGNNCGANNNNCANNNNTNAGNNNNNNNNNNNNN

>GW247-C2

NNNNNNNNNNNNNNNNNNNNNNNNNNNCNNNNNGTGNNGANGGGGCTTGNCCTGNCTGATNANTGGCGAACGGGTGAGTAACACGTGAGCANCCTGCCCCANACTCTGGGNTNNNTNNNGGAAACCGGAGNNNTTNCCGGATATGACCCTCGNACGCATGTGCTGGGGGTGGAAAGTTTTTCNNTNTNNNNNGGGNTCGCGGNCTNNNNTNNNGTTGGTGAGGTAGTGGCTCACCNNGGCGACNACGGGTAGCCGGNCTGAGAGGGCNACCGGNCACNCTGGGACTGANACACGGCCCANACTCCTACNGGAGGCAGCANTGGGGAATATTGCACAATGGGCGGAAGCCTGATGCACCGACCCCGCNNGAGGGATGACGGCCTTCGGGTTGTAAACCTCTTTCAGCAGGGAAGAAGCGAAAGTGACGGTACCTGCAGAAGAAGCACCGGCTAACTACGTGCCAGCAGCCGCGGTAATACGTAGGGTGCGAGCGTTGTCCGGAATTATTGGGCGTAAAGAGCTTGTAGGCGGTTTGTCGCGTCTGCTGTGAAAATCCGGGGCTCAACCCCGGACTTGCAGTGGGTACGGGCAGACTAGAGTGTGGTAGGGGAGACTGGAATTCCTGGTGTAGCGGTGGAATGCGCAGATATCAGGAGGAACACCGATGGCGAAGGCAGGTCTCTGGGCCACTACTGACGCTGASAAGCGAAAGCATGGGGAGCGAACAGGATTAGATACCCTGGTAGTCCATGCCGTAAACGTTGGGCGCTAGGTGTGGGGCTCATTCCACGAGTTCCGTGCCGCAGCTAACGCATTAAGCGCCCCGCCTGGGGAGTACGGCCGCAAGGCTAAAACTCAAANGAATTGACGGGGGCCCGCACAAGCGGCGGAGCATGCGGATTAATTCGATGCAACGCGAAGAACCTTACCAAGGCTTGACATACACCGGACACATCCAGAGATGGGTGCTTCTTCGGACTGGTGTACAGGTGGTGCATGGTTGTCGTCAGCTCGTGTCGTGAGATGTTGGGTTAAGTCCCGCAACGAGCGCAACCCTCGTTCTATGTTGCCAGCACGTGATGGTGGGGACTCATAGGAGACTGCCGGGGTCAACTCGGAGGAAGGTGGGGATGACGTCAAATCATCATGCCCCTTATGTCTTGGGCTTCACGCATGCTACAATGGCCGGTACAAAGGGTTGCGAAGCCGTGAGGTGGAGCGAATCCCAAAAAACCGGTCTCAGTTCGGATTGGGGTCTGCAACTCGACCCCATGAAGTCGGAGTCGCTAGTAATCGCAGATCAGCAACGCTGCGGTGAATACGTTCCCGGGCCTTGTACACACCGCCCGTCAAGTCACGAAAGTCGGTAACACCCGAAGCCGGTGGCCCAACCCTTGNGGGNGNAGCCNNNNNNNNNNNNNNNNN

>GW247-C5

NNNNNNNNNNNNNNNNNNNNNTGCANGTCGAACGGTGANGATGGNGCTTGCCCTGTCTGATCAGTGGCGANCGNNTNNNTAACACGTGAGCAACCTGCCCCAGACTCTGGGATAACTCCGGGAAACCGGANCTAATACCGGATATGACCCTCGTACGCNTGTGCTGGGGGTGGAAAGTTTTTCGGTCTGGGATGGGCTCGCGGCCTATCANCTTGTTGGTGAGGTAGTGGCTCACCNANGCGACNACNGGTAGCCGGCCTGANANGGCGACCGGCCNCACTGGGACTGANACACNGNCCANACTCCTACGGGAGGNNNCANTGGGGAATATTGCACAATGGGCGGAAGCCTGATGCAGCGACGCCGCGTGAGGGATGACGGCYTTSGGGTTGTAAACCTCTTTCAGCAGGGAANAAGCGAAAGTGACGGTACCTGCAGAAGAAGCNCCGGCTAACTACGTGCCAGCASCCGCNGTAATACNTAGGGTGCGAGCGTNGTCNGGAATTATTGGGCGTAAAGAGCTTGTAGGCGGTTTGTCGCGTCTGCTGTGAAAATCCGGGGCTCAACCCCGGACTTGCAGTGGGTACGGGCAGACTAGAGTGTGGTAGGGGAGACTGGAATTCCTGGTGTAGCGGTGGAATGCGCAGATATCAGGAGGAACACCGATGGNGAAGGCAGGTCTCTGGGCCACTACTGACGCTGAGAAGCGAAAGCATGGGGAGCGAACAGGATTAGATACCCTGGTARTCCATGCCGTAAACGTTGGGCGCTAGGTGTGGGGCTCATTCCACGAGTTCCGTGCCGCAGCTAACGCATTAAGCGCCCCGCCTGGNGAGTACGGCCGCAAGGNNAAAACTCAAAGGAATTGACGGGGGCCCGCACAANCGGCGGAGCATGCGGATTAATTCGATGCAACGCGAAGAACCTTACCAAGGCTTGACATACACCGGACACATCCAGAGATGGGTGCTTCTTCNNANTNNTGTACNGGTGGTGCATGGTTGTCGTCAGCTCGTGTCGTGAGATGTTGGGTTAAGTYCCGCAACGAGCGCAACCCTCGTTNTATGTTGCCAGCACGTGATGGTGGGGACTCATAGGAGACTGCCGGGGTCAANTNNNAGGAAGGNGGGGATGACGTCAAATCNTCATGCCCCCNATGTNNTGGGNTTCACNCATGTTACAATGGNCGGTACAAAGGGTTGNGAAGCCNTGAGGNNNANNNAATCCCNAANNNCNNNNNNNAGTTNNNATTGGGGTNTNCNNCTNNNCCCCANGAANTNGGAGTNNNNNNNNANNNNNGATCNNCNNNNNNNNNNNGAATNCNTTNCCGGGCNNTNNNCNCNCCNCCCNNNAAGNCNNNAAANTCGGTAACNCCCGAANNNGGNGGCCNNAACCNTNNNNNNNNNAGNNNTNNNNNNNNNNNNNNNNNNN

>GW247-D7

NNNNNNNNNCNNNNNANNNTGCNNGTCGAACGGTAACAGCACTTCNGTGGCTGACGAGTGGCGAACGGGTGAGTAATACATCGGAACGTGCCCGANAGTGGGGGATAACGCAGCGAAAGCTGTGCTAATACCGCATACGATCTACGGATGAAAGCAGGGGACCGCAAGGCCTTGCGCTCATGGAGCGGCCGATGGCAGATTANGTAGTTGGTGGGATAAAAGCTTACCAAGCCGACGATCTGTAGCTGGTCTGAGAGGACGACCAGCCACACTGGGACTGAGACACGGCCCANACTCCTACGGGAGGCAGCAGTGGGGAATTTTGGACAATGGGCGAAAGCCTGATCCAGCCATGCCGCGTGCAGGATGAAGGCCTTCGGGTTGTAAACTGCTTTTGTACGRAACGAAAAGGTCTTTTCTAATAAAGAAGGCTCATGACGGTACCGTAAGAATAAGCACCGGCTAACTACGTGCCAGCAGCCGCGGTAATACGTAGGGTGCAAGCGTTAATCGGAATTACTGGGCGTAAAGCGTGCGCAGGCGGTTATATAAGACAGATGTGAAATCCCCGGGCTCAACCTGGGAACTGCATTAGTGACTGTATAGCTGGAGTGCGGCAGAGGGGGATGGAATTCCGCGTGTAGCAGTGAAATGCGTAGATATGCGGAGGAACACCGATGGCGAAGGCAATCCCCTGGGCCTGCACTGACGCTCATGCACGAAAGCGTGGGGAGCAAACAGGATTAGATACCCTGGTAGTCCACGCCCTAAACGATGTCAACTGGTTGTTGGGAATTTACTTTCTCAGTAACGAAGCTAACGCGTGAAGTTGACCGCCTGGGGAGTACGGCCGCAAGGTTAAAACTCAANGGAATTGACGGGGACCCGCACAAGCGGTGGATGATGTGGTTTAATTCGATGCAACGCNAAAAACCTTACCCACNTTTGACATGTATNGAAGATCGCAGAGACGCGATTGTGCTNGAAAGAGAGCCATNACACNGGTGCTGCATGGCTGTCGTCAGCTCGTGTCGTGAGATGTTGGGTTAAGTCCCGCAACGAGCGCAACCCTTGTCATTAGTTGNTACATTTAGTTGGGCACTNTAATGAGACTGCCGGTGACAAACCGGAGGAAGGTGGGGATGACGTCAAGTCCTCATGGCCNTTATAGGTGGGGCTACACACGTCATACAATGGCTGGTACAAAGGGTTGCCAACCCGCGAGGGGGAGCCAATCCCATAAAGCCAGTCGTAGTCCGGATNGTAGTNTGCAACTCGACTACGNGAAGTCGGAATCGNTAGTAATCGTGGATCAGAATGTCACGGTGAATACGTTCCCGGGTNTTGTACACACCGCCCGTCACACCATGGGAGCGGGTTCTGCCAGAAGTAGGTAGCCTAACCGTAAGGANGGCGCNNNNNNNNNNNNNN

>GW247-E5

NNNNNNNNNNNNNNNGNNNNNNNNANNCNTGCNGTCGAACGGNGNCCTCNGGGCTTGCTTCNNGTGATCAGTGGCGAACGGGTGANTAACACGTGAGTAACCTGCCCCAGACTCTGGGATAACCCCGGGAAACCGGAGCTAATACCGGATATGACTCTCGCACGCATGTGCTGGGGGTGGAAAGTTTTTCGGTCTGGGATGGACTCGCGGCCTATCAGCTTGTTGGTGAGGTAGTGGCTCACCAAGGCGACGACGGGTAGCCGGCCTGAGAGGGCGACCGGCCACACTGGGACTGAGACACGGNCCAGWCTCCTACGGGAGGCAGCAGTGGGGAATATTGCACAATGGGCSCAAGCCTGATGCAGCGACGCCGCGTGAGGGATGANNGCCTTCGGGTTGTAAACCTCTTNCAGCAGGGAAGAAGCGAAAGTGACNGTACCTGCNGAAGAAGCACCNGCTAACTACNTGCCAGCAGCCGCGGTAATACGTAGGGTGCGAGCGTTGTCCGGAATTATTGGGCGTAAAGAGCTTGTAGGCGGTTTGTCGCGTCTGCTGTGAAAATCCGGGGCTCANCCCCGGACTTGCAGTGGGTACGGGCAGACTAGAGTGTGGTAGGGGAGACTGGAATTCCTGGTGTAGCGGTGGAATGCGCAGATATCAGGAGGAACACCGATGGCGAAGGCAGGTCTCTGGNCCACTACTGACGCTGAGAAGCGAAAGCATGGGGAGCGAACAGGATTAGATACCCTGGTAGTCCATGCCGTAAACGTTGGGCGCTAGGTGTGGGGCTCATTCCACGAGTTCCGTGCCGCAGCTAACGCATTAAGCGCCCCGCCTGGGGAGTACGGCCGCAAGGCTAAAACTCAAAGGAATTGACGGGGGCCCGCACNNAAGCGGCGGAGCATGCGGATTAATTMKAKSCAACGCGAAGAACCTTACCAAGGCTTGACATACACCGGACACATCCAGAGATGGGTGCTTCTTNGGNCTKGTGTACAGGTGGTGCATGGTTGTCGTCAGCTCGYGTCGTGAGATGTTGGGTTAAGTCCCGCAACGAGCGCAACCCTCGTTCTATGTTGCCAGCACGTGATGGTGGGGACTCATAGGAGACTGCCGGGGTCAACTCGGAGGAAGGTGGGGATGACGTCAAATCATCATGCCCCNTATGTCTTGGGCTTCACGCATGCTACAATGGCCGGTACAAAGGGCTGNGATACCGCGAGGTGGAGCGAATCCCAAAAAACCGGTNTCAGTTNGGANNGGGGTCTGCAACTCGACCCCATGAAGTCGGAGTCGCTAGTAATCGCAGATCAGCAACGCTGCGGTGAATACGTTCCCGGGCCTTGTACACACCNCCCGTCAAGTCNCGAAAGTCGGTAACACCCGAANCCGGNGGCCCAACNCCTTGTGNNNNNANCCNNNNNNNNNNNNNNNNNNNN

>GW531-E1

TGCAGTCGAACGATGATCCGGTGCTTGCACCGGGGATTAGTGGCGAACGGGTGAGTAACACGTGAGTAACCTGCCCTTGACTCTGGGATAAGCCTGGGAAACTGGGTCTAATACCGGATACGACCATTCCGCGCATGCGGTGGTGGTGGAAAGCTTTTGCGGTTTTGGATGGACTCGCGGCCTATCAGCTTGTTGGTGGGGTAATGGCCTACCAAGGCGACGACGGGTAGCCGGCCTGAGAGGGTGACCGGCCACACTGGGACTGAGACACGGCCCAGACTCCTACGGGAGGCAGCAGTGGGGAATATTGCACAATGGGCGCAAGCCTGATGCAGCGACGCCGCGTGAGGGATGACGGCCTTCGGGTTGTAAACCTCTTTCAGTAGGGAAGAAGCGAAAGTGACGGTACCTGCAGAAGAAGCGCCGGCTAACTACGTGCCAGCAGCCGCGGTAATACGTAGGGCGCAAGCGTTATCCGGAATTATTGGGCGTAAAGAGCTCGTAGGCGGTTTGTCGCGTCTGCTGTGAAAGACCGGGGCTCAACTCCGGTTCTGCAGTGGGTACGGGCAGACTAGAGTGATGTAGGGGAGACTGGAATTCCTGGTGTAGCGGTGAAATGCGCAGATATCAGGAGGAACACCGATGGCGAAGGCAGGTCTCTGGGCATTAACTGACGCTGAGGAGCGAAAGCATGGGGAGCGAACAGGATTAGATACCCTGGTAGTCCATGCCGTAAACGTTGGGCACTAGGTGTGGGGGACATTCCACGTTTTCCGCGCCGTAGCTAACGCATTAAGTGCCCCGCCTGGGGAGTACGGCCGCAAGGCTAAAACTCAAAGGAATTGACGGGGGCCCGCACAAGCGGCGGAGCATGCGGATTAATTCGATGCAACGCGAAGAACCTTACCAAGGCTTGACATGAACCGGTAACGCCTGGAAACAGGTGCCCCGCTTGCGGTCGGTTTACAGGTGGTGCATGGTTGTCGTCAGCTCGTGTCGTGAGATGTTGGGTTAAGTCCCGCAACGAGCGCAACCCTCGTTCTATGTTGCCAGCGGTTCGGCCGGGGACTCATAGGAGACTGCCGGGGTCAACTCGRAGGAAGGTGGGGACGACGTCAAATCATCATGCCCCTTATGTCTTGGGCTTCACGCATGCTACAATGGCCGGTACAAAGGGTTGCGATACTGTGAGGTGGAGCTAATCCCAAAAAGCCGGTCTCAGTTCGGATTGGGGTCTGCAACTCGACCCCATGAAGTCGGAGTCGCTAGTAATCGCAGATCAGCAACGCTGCGGTGAATACGTTCCCGGGCCTTGTACACACCGCCCGTCAAGTCACGAAAGTTGGTAACACCCGAAGCCGGTGGCCTAACCCT

>GW531-E2

AGTCGAACGGCAGCGCGGGAGCAATCCTGGCGGCGAGTGGCGAACGGGTGAGTAATACATCGGAACGTGCCCAATCGTGGGGGATAACGCAGCGAAAGCTGTGCTAATACCGCATACGATCTACGGATGAAAGCAGGGGATCGCAAGACCTTGCGCGAATGGAGCGGCCGATGGCAGATTAGGTAGTTGGTGAGGTAAAGGCTCMCCAAGCCTTCGATCTGTAGCTGGTCTGAGAGGACGMCCAGCCACACTGGGACTGAGACACGGCCCAGACTCCTACGGGAGGCAGCAGTGGGGAATTTTGGACAATGGGCGAAAGCCTGATCCAGCCATGCCGCGTGCAGGATGAAGGCCTTCGGGTTGTAAACTGCTTTTGTACGGAACGAAACGGTCTTTTCTAATACAGAAGGCTAATGACGGTACCGTAAGAATAAGCACCGGCTAACTACGTGCCAGCAGCCGCGGTAATACGTAGGGTGCAAGCGTTAATCGGAATTACTGGGCGTAAAGCGTGCGCAGGCGGTTATGTAAGACAGTTGTGAAATCCCCGGGCTCAACCTGGGAACTGCATCTGTGACTGCATAGCTAGAGTACGGTAGAGGGGGATGGAATTCCGCGTGTAGCAGTGAAATGCGTAGATATGCGGAGGAACACCGATGGCGAAGGCAATCCCCTGGACCTGTACTGACGCTCATGCACGAAAGCGTGGGGAGCAAACAGGATTAGATACCCTGGTAGTCCACGCCCTAAACGATGTCAACTGGTTGTTGGGTCTTCACTGACTCAGTAACGAAGCTAACGCGTGAAGTTGACCGCCTGGGGAGTACGGCCGCAAGGTTGAAACTCAAAGGAATTGACGGGGACCCGCACAAGCGGTGGATGATGTGGTTTAATTCGATGCAACGCGAAAAACCTTACCCACCTTTGACATGTACGGAATTCGCCAGAGATGGCTTAGTGCTCGAAAGAGAACCGTAACACAGGTGCTGCATGGCTGTCGTCAGCTCGTGTCGTGAGATGTTGGGTTAAGTCCCGCAACGAGCGCAACCCTTGTCATTAGTTGCTACATTCAGTTGGGCACTCTAATGAGACTGCCGGTGACAAACCGRAGGAAGGTGGGGATGACGTCAAGTCCTCATGGCCCTTATAGGTGGGGCTACMCACGTCATACAATGGCTGGTACAAAGGGTTGCCAACCCGCGAGGGGGAGCTAATCCCATAAAACCAGTCGTAGTCCGGATCGCAGTCTGCAACTCGACTGCGTGAAGTCGGAATCGCTAGTAATCGTGGATCAGAATGTCACGGTGAATACGTTCCCGGGTCTTGTACACACCGCCCGTCACACCATGGGAGCGGGTTCTGCCAGAAGTAGTTAGCTTAACCGCAAGGAGGGCGATACC

>GW531-L3

GGCAGCGCGGGAGCAATCCTGGCGGCGAGTGGCGAACGGGTGAGTAATACATCGGAACGTGCCCAATCGTGGGGGATAACGCAGCGAAAGCTGTGCTAATACCGCATACGATCTACGGATGAAAGCAGGGGATCGCAAGACCTTGCGCGAATGGAGCGGCCGATGGCAGATTAGGTAGTTGGTGAGGTAAAGGCTCACCAAGCCTTCGATCTGTAGCTGGTCTGAGAGGACGACCAGCCMCACTGGGACTGAGACACGGCCCAGAYTCCTACGGGAGGCAGCAGTGGGGAATTTTGGACAATGGGCGAAAGCCTGATCCAGCCATGCCGCGTGCAGGATGAAGGCCTTCGGGTTGTAAACTGCTTTTGTACGGAACGAAACGGTCTTTTCTAATACAGAAGGCTAATGACGGTACCGTAAGAATAAGCACCGGCTAACTACGTGCCAGCAGCCGCGGTAATACGTAGGGTGCAAGCGTTAATCGGAATTACTGGGCGTAAAGCGTGCGCAGGCGGTTATGTAAGACAGTTGTGAAATCCCCGGGCTCAACCTGGGAACTGCATCTGTGACTGCATAGCTAGAGTACGGTAGAGGGGGATGGAATTCCGCGTGTAGCAGTGAAATGCGTAGATATGCGGAGGAACACCGATGGCGAAGGCAATCCCCTGGACCTGTACTGACGCTCATGCACGAAAGCGTGGGGAGCAAACAGGATTAGATACCCTGGTAGTCCACGCCCTAAACGATGTCAACTGGTTGTTGGGTCTTCACTGACTCAGTAACGAAGCTAACGCGTGAAGTTGACCGCCTGGGGAGTACGGCCGCAAGGTTGAAACTCAAAGGAATTGACGGGGACCCGCACAAGCGGTGGATGATGTGGTTTAATTCGATGCAACGCGAAAAACCTTACCCACCTTTGACATGTACGGAATTCGCCAGAGATGGCTTAGTGCTCGAAAGAGAACCGTAACACAGGTGCTGCATGGCTGTCGTCAGCTCGTGTCGTGAGATGTTGGGTTAAGTCCCGCAACGAGCGCAACCCTTGTCATTAGTTGCTACATTCAGTTGGGCACTCTAATGAGACTGCCGGTGACAAACCGGAGGAAGGTGGGGATGACGTCAAGTCCTCATGGCCCTTATAGGTGGGGCTACACACGTCATACAATGGCTGGTACAAAGGGTTGCCAACCCGCGAGGGGGAGCTAATCCCATAAAACCAGTCGTAGTCCGGATCGCAGTCTGCAACTCGACTGCGTGAAGTCGGAATCGCTAGTAATCGTGGATCAGAATGTCACGGTGAATACGTTCCCGGGTCTTGTACACACCGCCCGTCACACCATGGGAGCGGGTTCTGCCAGAAGTAGTTAGCTTAACCGCAAGGAGGGCGAT

>GW531-L4

GTTGGGTGGCGAGTGGCGGACGGGTGAGTAATGCATCGGGATCTACCCTGACGTGGGGGATAACCTCGGGAAACCGGGACTAATACCGCATACGTCCTACGGGAGAAAGCGGGGGACCTTCGGGCCTCGCGCGGCAGGACGAACCGATGTGCGATTAGCTAGTTGGCGGGGTAATGGCCCACCAAGGCGACGATCGCTAGCTGGTCTGAGAGGATGATCAGCCACACTGGGACTGAGACACGGCCCAGACTCCTACGGGAGGCAGCAGTGGGGAATATTGGACAATGGGCGCAAGCCTGATCCAGCAATGCCGCGTGTGTGAAGAANGCCTTCGGGTTGTAAAGCACTTTTATCAGGAGCGAAATACCACGGGTTAATACCCTATGGGGCTGACGGTACCTGAGGAATAAGCACCGGCTAACTTCGTGCCAGCAGCCGCGGTAATACGAAGGGTGCAAGCGTTAATCGGAATTACTGGGCGTAAAGGGTGCGTAGGCGGTTACTTAAGTCTGTCGTGAAATCCCCGGGCTCAACCTGGGAATGGCGATGGATACTGGGTGGCTAGAGTGTGTCAGAGGATGGTGGAATTCCCGGTGTAGCGGTGAAATGCGTAGAGATCGGGAGGAACATCAGTGGCGAAGGCGGCCATCTGGGACAACACTGACGCTGAAGCACGAAAGCGTGGGGAGCAAACAGGATTAGATACCCTGGTAGTCCACGCCCTAAACGATGCGAACTGGATGTTGGTCTCAACTCGGAGATCAGTGTCGAAGCTAACGCGTTAAGTTCGCCGCCTGGGGAGTACGGTCGCAAGACTGAAACTCAAAGGAATTGACGGGGGCCCGCACAAGCGGTGGAGTATGTGGTTTAATTCGATGCAACGCGAAGAACCTTACCTGGCCTTGACATGTCCGGAATCCTGCAGAGATGCGGGAGTGCCTTCGGGAATCGGAACACAGGTGCTGCATGGCTGTCGTCAGCTCGTGTCGTGAGATGTTGGGTTAAGTCCCGCAACGAGCGCAACCCTTGTCCTTAGTTGCCAGCACGTAATGGTGGGAACTCTAAGGAGACTGCCGGTGACAAACCGGANGGAAGGTGGGGATGACGTCAAGTCATCATGGCCCTTACGGCCAGGGCTACACACGTACTACAATGGTCGGTACAGAGGGTTGCAATACCGCGAGGTGGAGCCAATCCCAGAAAGCCGATCCCAGTCCGGATTGGAGTCTGCAACTCGACTCCATGAAGTCGGAATCGCTAGTAATCGCGGATCAGCTATGCCGCGGTGAATACGTTCCCGGGCCTTGTACACACCGCCCGTCACACCATGGGAGTGGGTTGCTCCAGAAGGCGTTAGTCTAACCGCAAGGGGGACG

>GW531-R1

TGCAGTCGAGCGGTAGAGAGAAGCTTGCTTCTCTTGAGAGCGGCGGACGGGTGAGTAATGCCTAGGAATCTGCCTGGTAGTGGGGGATAACGTTCGGAAACGAACGCTAATACCGCATACGTCCTACGGGAGAAAGCAGGGGACCTTCGGGCCTTGCGCTATCAGATGAGCCTAGGTCGGATTAGCTAGTTGGTGGGGTAATGGCTCACCAAGGSGACGATCCGTAACTGGTCTGAGAGGATGATCAGTCMCACTGGAACTGAGACMCGGTCCAGACTCCTACGGGAGGCAGCAGTGGGGAATATTGGACAATGGGCGAAAGCCTGATCCAGCCATGCCGCGTGTGTGAAGAAGGTCTTCGGATTGTAAAGCACTTTAAGTTGGGAGGAAGGGCCATTACCTAATACGTGATGGTTTTGACGTTACCGACAGAATAAGCACCGGCTAACTCTGTGCCAGCAGCCGCGGTAATACAGAGGGTGCAAGCGTTAATCGGAATTACTGGGCGTAAAGCGCGCGTAGGTGGTTCGTTAAGTTGGATGTGAAATCCCCGGGCTCAACCTGGGAACTGCATTCAAAACTGTCGAGCTAGAGTATGGTAGAGGGTGGTGGAATTTCCTGTGTAGCGGTGAAATGCGTAGATATAGGAAGGAACACCAGTGGCGAAGGCGACCACCTGGACTGATACTGACACTGAGGTGCGAAAGCGTGGGGAGCAAACAGGATTAGATACCCTGGTAGTCCACGCCGTAAACGATGTCAACTAGCCGTTGGGAGCCTTGAGCTCTTAGTGGCGCAGCTAACGCATTAAGTTGACCGCCTGGGGAGTACGGCCGCAAGGTTAAAACTCAAATGAATTGACGGGGGCCCGCACAAGCGGTGGAGCATGTGGTTTAATTCGAAGCAACGCGAAGAACCTTACCAGGCCTTGACATCCAATGAACTTTCCAGAGATGGATTGGTGCCTTCGGGAACATTGAGACAGGTGCTGCATGGCTGTCGTCAGCTCGTGTCGTGAGATGTTGGGTTAAGTCCCGTAACGAGCGCAACCCTTGTCCTTAGTTACCAGCACGTWATGGTGGGCACTCTAAGGAGACTGCCGGTGACAAACCGGAGGAAGGTGGGGATGACGTCAAGTCATCATGGCCCTTACGGCCTGGGCTACMCACGTGCTACAATGGTCSGTACAGAGGGTTGCCAAGCCGCGAGGTGGAGCTAATCCCATAAAACCGATCGTAGTCCGGATCGCAGTCTGCAACTCGACTGCGTGAAGTCGGAATCGCTAGTAATCGCGAATCAGAATGTCGCGGTGAATACGTTCCCGGGCCTTGTACACACCGCCCGTCACACCATGGGAGTGGGTGCACCAGAAGTAGCTAGTCTAACCTTCGGGAGGACGGTAC

>GW531-T3

TGCAGTCGAACGGCAGCGCGGGAGCAATCCTGGCGGCGAGTGGCGAACGGGTGAGTAATACATCGGAACGTGCCCAATCGTGGGGGATAACGCAGCGAAAGCTGTGCTAATACCGCATACGATCTACGGATGAAAGCAGGGGATCGCAAGACCTTGCGCGAATGGAGCGGCCGATGGCAGATTAGGTAGTTGGTGAGGTAAAGGCTCACCAAGCCTTCGATCTGTAGCTGGTCTGAGAGGACGACCAGCCACACTGGGACTGAGACACGGNCCAGACTCCTACGGGAGGCAGCAGTGGGGAATTTTGGACAATGGGCGAAAGCCTGATCCAGCCATGCCGCGTGCAGGATGAAGGCCTTCGGGTTGTAAACTGCTTTTGTACGGAACGAAACGGTCTTTTCTAATACAGAAGGCTAATGACGGTACCGTAAGAATAAGCACCGGCTAACTACGTGCCAGCAGCCGCGGTAATACGTAGGGTGCAAGCGTTAATCGGAATTACTGGGCGTAAAGCGTGCGCAGGCGGTTATGTAAGACAGTTGTGAAATCCCCGGGCTCAACCTGGGAACTGCATCTGTGACTGCATAGCTAGAGTACGGTAGAGGGGGATGGAATTCCGCGTGTAGCAGTGAAATGCGTAGATATGCGGAGGAACACCGATGGCGAAGGCAATCCCCTGGACCTGTACTGACGCTCATGCACGAAAGCGTGGGGAGCAAACAGGATTAGATACCCTGGTAGTCCACGCCCTAAACGATGTCAACTGGTTGTTGGGTCTTCACTGACTCAGTAACGAAGCTAACGCGTGAAGTTGACCGCCTGGGGAGTACGGCCGCAAGGTTGAAACTCAAAGGAATTGACGGGGACCCGCACAAGCGGTGGATGATGTGGTTTAATTCGATGCAACGCGAAAAACCTTACCCACCTTTGACATGTACGGAATTCGCCAGAGATGGCTTAGTGCTCGAAAGAGAACCGTAACACAGGTGCTGCATGGCTGTCGTCAGCTCGTGTCGTGAGATGTTGGGTTAAGTCCCGCAACGAGCGCAACCCTTGTCATTAGTTGCTACATTCAGTTGGGCACTCTAATGAGACTGCCGGTGACAAACCGGAGGAAGGTGGGGATGACGTCAAGTCCTCATGGCCCTTATAGGTGGGGCTACACACGTCATACAATGGCTGGTACAAAGGGTTGCCAACCCGCGAGGGGGAGCTAATCCCATAAAACCAGTCGTAGTCCGGATCGCAGTCTGCAACTCGACTGCGTGAAGTCGGAATCGCTAGTAATCGTGGATCAGAATGTCACGGTGAATACGTTCCCGGGTCTTGTACACACCGCCCGTCACACCATGGGAGCGGGTTCTGCCAGAAGTAGTTAGCTTAACCGCAAGGAGGGCGAT

>GW531-T4

TGCAGTCGAGCGGTAGAGAGAAGCTTGCTTCTCTTGAGAGCGGCGGACGGGTGAGTAATGCCTAGGAATCTGCCTGGTAGTGGGGGATAACGTTCGGAAACGAACGCTAATACCGCATACGTCCTACGGGAGAAAGCAGGGGACCTTCGGGCCTTGCGCTATCAGATGAGCCTAGGTCGGATTAGCTAGTTGGTGGGGTAATGGCTCACCAAGGCGACGATCCGTAACTGGTCTGAGAGGATGATCAGTCACACTGGAACTGAGACACGGTCCAGACTCCTACGGGAGGCAGCAGTGGGGAATATTGGACAATGGGCGAAAGCCTGATCCAGCCATGCCGCGTGTGTGAAGAAGGTCTTCGGATTGTAAAGCACTTTAAGTTGGGAGGAAGGGCCATTACCTAATACGTGATGGTTTTGACGTTACCGACAGAATAAGCACCGGCTAACTCTGTGCCAGCAGCCGCGGTAATACAGAGGGTGCAAGCGTTAATCGGAATTACTGGGCGTAAAGCGCGCGTAGGTGGTTCGTTAAGTTGGATGTGAAATCCCCGGGCTCAACCTGGGAACTGCATTCAAAACTGTCGAGCTAGAGTATGGTAGAGGGTGGTGGAATTTCCTGTGTAGCGGTGAAATGCGTAGATATAGGAAGGAACACCAGTGGCGAAGGCGACCACCTGGACTGATACTGACACTGAGGTGCGAAAGCGTGGGGAGCAAACAGGATTAGATACCCTGGTAGTCCACGCCGTAAACGATGTCAACTAGCCGTTGGGAGCCTTGAGCTCTTAGTGGCGCAGCTAACGCATTAAGTTGACCGCCTGGGGAGTACGGCCGCAAGGTTAAAACTCAAATGAATTGACGGGGGCCCGCACAAGCGGTGGAGCATGTGGTTTAATTCGAAGCAACGCGAAGAACCTTACCAGGCCTTGACATCCAATGAACTTTCCAGAGATGGATTGGTGCCTTCGGGAACATTGAGACAGGTGCTGCATGGCTGTCGTCAGCTCGTGTCGTGAGATGTTGGGTTAAGTCCCGTAACGAGCGCAACCCTTGTCCTTAGTTACCAGCACGTAWTGGTGGNCACTCTAAGGAGACTGCCGGTGACAAACCGGAGGAAGGTGGGGATGACGTCAAGTCATCATGGCCCTTACGGCCTGGGCTACACACGTGCTACAATGGTCGGTACAGAGGGTTGCCAAGCCGCGAGGTGGAGCTAATCCCATAAAACCGATCGTAGTCCGGATCGCAGTCTGCAACTCGACTGCGTGAAGTCGGAATCGCTAGTAATCGCGAATCAGAATGTCGCGGTGAATACGTTCCCGGGCCTTGTACACACCGCCCGTCACACCATGGGAGTGGGTTGCACCAGAAGTAGCTAGTCTAACCTTCGGGAGGACGGT

>GW531-T5

TGCAGTCGAACGGCAGCACGGGAGCAATCCTGGTGGCGAGTGGCGAACGGGTGAGTAATACATCGGAACGTGCCCTGTCGTGGGGGATAACTAGTCGAAAGATTAGCTAATACCGCATACGACCTGAGGGTGAAAGCGGGGGACCGCAAGGCCTCGCGCGATAGGAGCGGCCGATGTCTGATTAGCTAGTTGGTGGGGTAAAGGCCCACCAAGGCGACGATCAGTAGCTGGTCTGAGAGGACGATCAGCCACACTGGGACTGAGACACGGCCCAGACTCCTACGGGAGGCAGCAGTGGGGAATTTTGGACAATGGGGGCAACCCTGATCCAGCAATGCCGCGTGTGTGAAGAAGGCCTTCGGGTTGTAAAGCACTTTTGTCCGGAAAGAAATCCCTTGCTCTAATACAGCGGGGGGATGACGGTACCGGAAGAATAAGCACCGGCTAACTACGTGCCAGCAGCCGCGGTAATACGTAGGGTGCGAGCGTTAATCGGAATTACTGGGCGTAAAGCGTGCGCAGGCGGTTTTGTAAGACAGGCGTGAAATCCCCGAGCTCAACTTGGGAATGGCGCTTGTGACTGCAAGGCTAGAGTATGTCAGAGGGGGGTAGAATTCCACGTGTAGCAGTGAAATGCGTAGAGATGTGGAGGAATACCGATGGCGAAGGCAGCCCCCTGGGACGTCACTGACGCTCATGCACGAAAGCGTGGGGAGCAAACAGGATTAGATACCCTGGTAGTCCACGCCCTAAACGATGTCAACTAGTTGTTGGGGATTCATTTCTTCAGTAACGTAGCTAACGCGTGAAGTTGACCGCCTGGGGAGTACGGTCGCAAGATTAAAACTCAAAGGAATTGACGGGGACCCGCACAAGCGGTGGATGATGTGGATTAATTCGATGCAACGCGAAAAACCTTACCTACCCTTGACATGCCACTAACGAAGCAGAGATGCATCAGGTGCCCGAAAGGGAAAGTGGACACAGGTGCTGCATGGCTGTCGTCAGCTCGTGTCGTGAGATGTTGGGTTAAGTCCCGCAACGAGCGCAACCCTTGTCTTTAGTTGCTACGCAAGAGCACTCTARARAGACTGCCNGTGACAAACCGGANGGAAGGTGGGGATGACGTCAAGTCCTCATGGCCCTTATGGGTAGGGCTTCACACGTCATACAATGGTGCGTACAGAGGGTTGCCAACCCGCGAGGGGGAGCTAATCCCAGAAAACGCATCGTAGTCCGGATCGTAGTCTGCAACTCGACTACGTGAAGCTGGAATCGCTAGTAATCGCGGATCAGCATGCCGCGGTGAATACGTTCCCGGGTCTTGTACACACCGCCCGTCACACCATGGGAGTGGGTTTTGCCAGAAGTAGTTAGCCTAACCGCAAGGAGGGCGAT

>GW531-T6

CGATGATCCCAGCTTGCTGGGGGATTAGTGGCGAACGGGTGAGTAACACGTGAGTAACCTGCCCTTGACTCTGGGATAAGCCTGGGAAACTGGGTCTAATACCGGATACGACCATTCCACGCATGTGGTGGTGGTGGAAAGCTTTTGTGGTTTTGGATGGACTCGCGGCCTATCAGCTTGTTGGTGGGGTAATGGCCTACCAAGGCGACGACGGGTAGCCGSCCTGAGAGGGTGACCGGCCNCACTGGGACTGAGACACGGNCCCAGAYTCCTACGGGAGGCAGCAGTGGGGAATATTGCMCAATGGGCGCAAGCCTGATGCAGCGACGCCGCGTGAGGGATGACGGCCTTCGGGTTGTAAACCTCTTTCAGTAGGGAAGAAGCGAAAGTGACGGTACCTGCAGAAGAAGCGCCGGCTAACTACGTGCCAGCAGCCGCGGTAATACGTAGGGCGCAAGCGTTATCCGGAATTATTGGGCGTAAAGAGCTCGTAGGCGGTTTGTCGCGTCTGCTGTGAAAGACCGGGGCTCAACTCCGGTTCTGCAGTGGGTACGGGCAGACTAGAGTGCAGTAGGGGAGACTGGAATTCCTGGTGTAGCGGTGAAATGCGCAGATATCAGGAGGAACACCGATGGCGAAGGCAGGTCTCTGGGCTGTAACTGACGCTGAGGAGCGAAAGCATGGGGAGCGAACAGGATTAGATACCCTGGTAGTCCATGCCGTAAACGTTGGGCACTAGGTGTGGGGGACATTCCACGTTTTCCGCGCCGTAGCTAACGCATTAAGTGCCCCGCCTGGGGAGTACGGCCGCAAGGCTAAAACTCAAAGGAATTGACGGGGGCCCGCACAAGCGGCGGAGCATGCGGATTAATTCGATGCAACGCGAAGAACCTTACCAAGGCTTGACATGAACCGGTAATACCTGGAAACAGGTGCCCCGCTTGCGGTCGGTTTACAGGTGGTGCATGGTTGTCGTCAGCTCGTGTCGTGAGATGTTGGGTTAAGTCCCGCAACGAGCGCAACCCTCGTTCTATGTTGCCAGCGGTTCGGCCGGGGACTCATAGGAGACTGCCGGGGTCAACTCGNAGGAAGGTGGGGACGACGTCAAATCATCATGCCCCTTATGTCTTGGGCTTCACGCATGCTACAATGGCCGGTACAAAGGGTTGCGATACTGTGAGGTGGAGCTAATCCCAAAAAGCCGGTCTCAGTTCGGATTGGGGTCTGCAACTCGACCCCATGAAGTCGGAGTCGCTAGTAATCGCAGATCAGCAACGCTGCGGTGAATACGTTCCCGGGCCTTGTACACACCGCCCGTCAAGTCACGAAAGTTGGTAACACCCGAAGCCGGTGGCCTAACCCTTG

>FW510-T10

TGCAGTCGACGATGAAGCCAGCTTGCTGGGTGGATTAGTGGCGAACGGGTGAGTAACACGTGAGTAACCTGCCCTTAACTCTGGGATAAGCCTGGGAAACTGGGTCTAATACCGGATAGGAGCGTCCACCGCATGGTGGGTGTTGGAAAGATTTATCGGTTTTGGATGGACTCGCGGCCTATCAGCTTGTTGGTGAGGTAATGGCTCACCAAGGCGACGACGGGTAGCCGGCCTGAGAGGGTGACCGGCCACACTGGGACTGAGACACGGCCCAGACTCCTACGGGAGGCAGCAGTGGGGAATATTGCACAATGGGCGCAAGCCTGATGCAGCGACGCCGCGTGAGGGATGACGGCCTTCGGGTTGTAAACCTCTTTCAGTAGGGAAGAAGCGAAAGTGACGGTACCTGCAGAAGAAGCACCGGCTAACTACGTGCCAGCAGCCGCGGTAATACGTAGGGTGCGAGCGTTATCCGGAATTATTGGGCGTAAAGAGCTCGTAGGCGGTTTGTCGCGTCTGTCGTGAAAGTCCGGGGCTTAACCCCGGATCTGCGGTGGGTACGGGCAGACTAGAGTGCAGTAGGGGAGACTGGAATTCCTGGTGTAGCGGTGGAATGCGCAGATATCAGGAGGAACACCGATGGCGAAGGCAGGTCTCTGGGCTGTAACTGACGCTGAGGAGCGAAAGCATGGGGAGCGAACAGGATTAGATACCCTGGTAGTCCATGCCGTAAACGTTGGGCACTAGGTGTGGGGACCATTCCACGGTTTCCGCGCCGCAGCTAACGCATTAAGTGCCCCGCCTGGGGAGTACGGCCGCAAGGCTAAAACTCAAAGGAATTGACGGGGGCCCGCACAAGCGGCGGAGCATGCGGATTAATTCGATGCAACGCGAAGAACCTTACCAAGGCTTGACATGTTCTCGATCGCCGTAGAGATACGGTTTCCCCTTTGGGGCGGGTTCACAGGTGGTGCATGGTTGTCGTCAGCTCGTGTCGTGAGATGTTGGGTTAAGTCCCGCAACGAGCGCAACCCTCGTTCCATGTTGCCAGCACGTAATGGTNGGGGACTCATGGGAGACTGCCGGGGTCAACTCGGAGGAAGGTGAGGACGACGTCAAATCATCATGCCCCTTATGTCTTGGGCTTCACGCATGCTACAATGGCCGGTACAATGGGTTGCGATACTGTGAGGTGGAGCTAATCCCAAAAAGCCGGTCTCAGTTCGGATTGGGGTCTGCAACTCGACCCCATGAAGTCGGAGTCGCTAGTAATCGCAGATCAGCAACGCTGCGGTGAATACGTTCCCGGGCCTTGTACACACCGCCCGTCAAGTCACGAAAGTCGGTAACACCCGAAGCCGGTGGCCTAACCCTTG

>FW510-T9

TGCAGTCGAGCGGACAATTAGGAGCTTGCTCCTAATTGTTAGCGGCGGACGGGTGAGTAACACGTGGGTAACCTGCCTGTAAGACTGGGATAACTCCGGGAAACCGGGGCTAATACCGGATAACTTCTTTTCTCGCATGAAGAAAGATTGAAAGATGGCTTCTGCTATCACTTACAGATGGACCCGCGGCGCATTAGCTAGTTGGTGAGGTAACGGCTCACCAAGGCAACGATGCGTAGCCGACCTGAGAGGGTGATCGGCCACACTGGGACTGAGACACGGCCCAGACTCCTACGGGAGGCAGCAGTAGGGAATCTTCCGCAATGGACGAAAGTCTGACGGAGCAACGCCGCGTGAGTGATGAAGGTTTTCGGATCGTAAAACTCTGTTGTTAGGGAAGAACAAGTACCGTTCGAATAGGGCGGTACCTTGACGGTACCTAACCAGAAAGCCACGGCTAACTACGTGCCAGCAGCCGCGGTAATACGTAGGTGGCAAGCGTTGTCCGGAATTATTGGGCGTAAAGCGCGCGCAGGCGGTTTCTTAAGTCTGATGTGAAAGCCCACGGCTCAACCGTGGAGGGTCATTGGAAACTGGGAGACTTGAGTGCAGAAGAGGAGAGTGGAATTCCACGTGTAGCGGTGAAATGCGTAGAGATGTGGAGGAACACCAGTGGCGAAGGCGACTCTCTGGTCTGTAACTGACGCTGAGGTGCGAAAGCGTGGGGAGCGAACAGGATTAGATACCCTGGTAGTCCACGCCGTAAACGATGAGTGCTAAGTGTTAGAGGGTTTCCGCCCTTTAGTGCTGCAGCTAACGCATTAAGCACTCCGCCTGGGGAGTACGGCCGCAAGGCTGAAACTCAAAGGAATTGACGGGGGCCCGCACAAGCGGTGGAGCATGTGGTTTAATTCGAAGCAACGCGAAGAACCTTACCAGGTCTTGACATCCTCTTGACCTCTCTAGAGATAGGGATTTCCCTTCGGGGACAAGAGTGACAGGTGGTGCATGGTTGTCGTCAGCTCGTGTCGTGAGATGTTGGGTTAAGTCCCGCAACGAGCGCAACCCTTGACCTTAGTTGCCAGCATTTAGTTGGGCACTCTAAGGTGACTGCCGGTGACAAACCGGAGGAAGGTGGGGATGACGTCAAATCATCATGCCCCTTATGACCTGGGCTACACACGTGCTACAATGGATGGTACAAAGGGCTGCGAGACCGCGAGGTTTAGCCAATCCCATAAAACCATTCTCAGTTCGGATTGTAGGCTGCAACTCGCCTACATGAAGCCGGAATCGCTAGTAATCGCGGATCAGCATGCCGCGGTGAATACGTTCCCGGGCCTTGTACACACCGCCCGTCACACCACGAGAGTTTGTAACACCCGAAGTCGGTGAGGTAACCTTTGGAGCCAGCCGCCGA

>FW510-R11

TGCAGTCGAACGGGCGTAGCAATACGTCAGTGGCAGACGGGTGAGTAACGCGTGGGAACGTACCTTTTGGTTCGGAACAACTGAGGGAAACTTCAGCTAATACCGGATAAGCCCTTACGGGGAAAGATTTATCGCCGAAAGATCGGCCCGCGTCTGATTAGCTAGTTGGTGAGGTAATGGCTCACCAAGGCGACGATCAGTAGCTGGTCTGAGAGGATGATCAGCCACATTGGGACTGAGACACGGCCCAAACTCCTACGGGAGGCAGCAGTGGGGAATATTGGACAATGGGCGAAAGCCTGATCCAGCCATGCCGCGTGAGTGATGAAGGCCCTAGGGTTGTAAAGCTCTTTTGTGCGGGAAGATAATGACGGTACCGCAAGAATAAGCCCCGGCTAACTTCGTGCCAGCAGCCGCGGTAATACGAAGGGGGCTAGCGTTGCTCGGAATCACTGGGCGTAAAGGGTGCGTAGGCGGGTCTTTAAGTCAGGGGTGAAATCCTGGAGCTCAACTCCAGAACTGCCTTTGATACTGAGGATCTTGAGTTCGGGAGAGGTGAGTGGAACTGCGAGTGTAGAGGTGAAATTCGTAGATATTCGCAAGAACACCAGTGGCGAAGGCGGCTCACTGGCCCGATACTGACGCTGAGGCACGAAAGCGTGGGGAGCAAACAGGATTAGATACCCTGGTAGTCCACGCCGTAAACGATGAATGCCAGCCGTTAGTGGGTTTACTCACTAGTGGCGCAGCTAACGCTTTAAGCATTCCGCCTGGGGAGTACGGTCGCAAGATTAAAACTCAAAGGAATTGACGGGGGCCCGCACAAGCGGTGGAGCATGTGGTTTAATTCGACGCAACGCGCAGAACCTTACCAGCCCTTGACATGTCCATGACCGGTCGCAGAGATGTGACCTTCTCTTCGGAGCATGGAGCACAGGTGCTGCATGGCTGTCGTCAGCTCGTGTCGTGAGATGTTGGGTTAAGTCCCGCAACGAGCGCAACCCCCGTCTTTAGTTGCTACCATTTAGTTGAGCACTCTAAAGAGACTGCCGGTGATAAGCCGCGAGGAAGGTGGGGATGACGTCAAGTCCTCATGGCCCTTACGGGCTGGGCTACACACGTGCTACAATGGCGGTGACAATGGGATGCAAAAGGGCAACCTCTAGCAAATCTCAAAAAGCCGTCTCAGTTCGGATTGGGCTCTGCAACTCGAGCCCATGAAGTTGGAATCGCTAGTAATCGTGGATCAGCATGCCACGGTGAATACGTTCCCGGGCCTTGTACACACCGCCCGTCACACCATGGGAGTTGGTTTTACCTGAAGGCGGTGCGCTAACCCGCAAGGGAGGCAGCCGACC

>FW510-R10

TGCAGTCGACGGCAGCATGAGAGAGCTTGCTCTCTTGATGGCGAGTGGCGGACGGGTGAGTAATACATCGGGACCTACCCATGACGTGGGGGATAACCTCGGGAAACCGGGACTAATACCGCATACGTCCTACGGGAGAAAGCGGGGGACCTTCGGGCCTCGCGCGTCTGGACGGGCCGATGTTCGATTAGCTTGTTGGTGAGGTAATGGCTCACCAAGGCGACGATCGATAGCTGGTCTGAGAGGATGATCAGCCACATTGGGACTGAGACACGGCCCAAACTCCTACGGGAGGCAGCAGTGGGGAATATTGGACAATGGGCGAAAGCCTGATCCAGCAATGCCGCGTGTGTGAAGAAGGCCTTCGGGTTGTAAAGCACTTTTATCAGGAACGAAATCGGCAAGGTTAATAACCGTGTCGTCTGACGGTACCTGAGGAATAAGCACCGGCTAACTTCGTGCCAGCAGCCGCGGTAATACGAAGGGTGCAAGCGTTAATCGGAATTACTGGGCGTAAAGGGTGCGTAGGCGGTTAGTTAAGTCTGTTGTGAAATCCCCGGGCTCAACCTGGGAATGGCAATGGATACTGGCTGGCTAGAGTGTGTCAGAGGATGGTGGAATTCCCGGTGTAGCGGTGAAATGCGTAGAGATCGGGAGGAACATCAGTGGCGAAGGCGGCCATCTGGGACAACACTGACGCTGAAGCACGAAAGCGTGGGGAGCAAACAGGATTAGATACCCTGGTAGTCCACGCCCTAAACGATGCGAACTGGACGTTGGTCTCAACTCGGAGATCAGTGTCGAAGCTAACGCGTTAAGTTCGCCGCCTGGGGAGTACGGTCGCAAGACTGAAACTCAAAGGAATTGACGGGGGCCCGCACAAGCGGTGGAGTATGTGGTTTAATTCGATGCAACGCGAAGAACCTTACCTGGCCTTGACATGTCGCGAATCCTGCAGAGATGCGGGAGTGCCTTCGGGAATGCGAACACAGGTGCTGCATGGCTGTCGTCAGCTCGTGTCGTGAGATGTTGGGTTAAGTCCCGCAACGAGCGCAACCCTTATCCTTAGTTGCCAGCACGTAATGGTGGGAACTCTAAGGAGACTGCCGGTGACAAACCGGAGGAAGGTGGGGATGACGTCAAGTCCTCATGGCCCTTACGGCCAGGGCTACACACGTACTACAATGGTCGGTACAGAGGGTTGCAATACCGCGAGGTGGAGCCAATCCCAGAAAGCCGATCCCAGTCCGGATTGGAGTCTGCAACTCGACTCCATGAAGTCGGAATCGCTAGTAATCGCGGATCAGCTATGCCGCGGTGAATACGTTCCCGGGCCTTGTACACACCGCCCGTCACACCATGGGAGTGGGTTGCTCCAGAAGGCGTTAGTCTAACCGCAAGGAGGACGACGCC

>GW247-26LB

TCGAACGGCAGCACGAAGAGCTTGCTCTTTGGTGGCGAGTGGCGAACGGGTGAGTAATATATCGGAACGTGCCCAGTAGCGGGGGATAACTGGCCGAAAGGTCAGCTAATACCGCATACGTCCTACGGGAGAAAGGGGGGGATCGCAAGACCTCTCACTATTGGAGCGGCCGATATCAGATTAGCTTGTTGGTGAGGTAAAGGCTCACCAAGGCCGCGATCTGTAGCTGGTTTGAGAGGACGACCAGCCACACTGGGACTGAGACACGGCCCAGACTCCTACGGGAGGCAGCAGTGGGGAATTTTGGACAATGGGGGCAACCCTGATCCAGCCATCCCGCGTGTGCGATGAAGGCCNTTCGGGTTGTAAAGCACTTTTGGCAGGGAAGAAAAGGSCTWGGATAATACCCTGGGCTCATGACGGTACCTGCAGAATAAGCANCGGCTAACTACGTGCCAGCAGCCGCGGTAATACGTAGGGTGCAAGSSTTAATYGGAATTACTGGGCGTAAAGCGTGCGCAGSCGGTTCGRAAAGAAAGGTGTGAAATCCCAGGNGCTTAACCTTGGAACTGCACTTTTAACTACCGGGCTAGAGTACGTCAGAGGGGGGTAGAATTCCACGTGTAGCAGTGAAATGCGTAGAGATGTGGAGGAATACCGATGGCGAAGGCAGCCCCCTGGGATGATACTGACGCTCATGCACGAAAGCGTGGGGAGCAAACAGGATTAGATACCCTGGTAGTCCACGCCCTAAACGATGTCAACTAGCTGTTGGGGTTTATTAACCTTAGTAGCGCAGCTAACGCGTGAAGTTGACCGCCTGGGGAGTACGGTCGCAAGATTAAAACTCAAAGGAATTGACGGGGACCCGCACAAGCGGTGGATGATGTGGATTAATTCGATGCAACGCGAAAAMCCTTACCTACCCTTGACATGTCTGGAAGATTCAAGAGATTGAATTGTGCTCGCNAGAGAACCGGAACACAGGTGCTGCATGGCTGTCGTCAGCTCGTGTCGTGAGATGTTGGGTTAAGTCCCGCAACGAGCGCAACCCTTGCCATTAGTTGCTACATTTAGTTGGGNCACTCTAATGGGACTGCCGGTGACAAMCSGGAGGAAGGTGGGGATGACGTCAAGYCCTCATGGCCCTTATGGGTAGGGCTTCACACGTCATACAATGGTCGGGACAGAGGGATGCCAAGCCGCGAGGTGGAGCCAATCTCAGAAACCCGATCGTAGTCCGGATCGCAGTCTGCAACTCGACTGCGTGAAGTCGGAATCGCTAGTAATCGCGGATCAGCATGTCGCGGTGAATACGTTCCCGGGTCTTGTACACACCGCCCGTCACACCATGGGAGTGGGTTTCACCAGAAGTAGGTAGCCTA

>FW510-T8

AGTCGAACGGCAGCACAGCCGTAGCAATACGGTGGGTGGCGAGTGGCGGACGGGTGAGTAAAGCATCGGGACCTACCCAGACGTGGGGGATAACCTCGGGAAACCGGGACTAATACCGCATACGTCCTACGGGAGAAAGCGGGGGACCTTCGGGCCTCGCGCGGTTGGACGGACCGATGTGCGATTAGCTAGTTGGTAGGGTAATGGCCTACCAAGGCGACGATCGCTAGCTGGTCTGAGAGGATGATCAGCCACACTGGGACTGAGACACGGCCCAGACTCCTACGGGAGGCAGCAGTGGGGAATATTGGMCAATGGGCGCAAGCCTGATCCAGCAATGCCGCGTGTGTGAAGAAGGCCTTCGGGTTGTAAAGCACTTTTATCAGGAGCGAAATACCACGGGTCAATACCCTGTGGGGCTGACGGTACCTGAGGAATAAGCACCGGCTAACTTCGTGCCAGCAGCCGCGGTAATACGAAGGGTGCAAGCGTTAATCGGAATTACTGGGCGTAAAGGGTGCGTAGGCGGTTACTTAAGTCTGTCGTGAAATCCCCGGGCTCAACCTGGGAATGGCGATGGATACTGGGTGGCTAGAGTGTGTCAGAGGATGGTGGAATTCCCGGTGTAGCGGTGAAATGCGTAGAGATCGGGAGGAACATCAGTGGCGAAGGCGGCCATCTGGGACAACACTGACGCTGAAGCACGAAAGCGTGGGGAGCAAACAGGATTAGATACCCTGGTAGTCCACGCCCTAAACGATGCGAACTGGATGTTGGTCTCAACTCGGAGATCAGTGTCGAAGCTAACGCGTTAAGTTCGCCGCCTGGGGAGTACGGTCGCAAGACTGAAACTCAAAGGAATTGACGGGGGCCCGCACAAGCGGTGGAGTATGTGGTTTAATTCGATGCAACGCGAAGAACCTTACCTGGCCTTGACATGTCCGGAATCCTGCAGAGATGCGGGAGTGCCTTCGGGAATCGGAACACAGGTGCTGCATGGCTGTCGTCAGCTCGTGTCGTGAGATGTTGGGTTAAGTCCCGCAACGAGCGCAACCCTTGTCCTTAGTTGCCAGCACGTAATGGTGGGAACTCTAAGGAGACTGCCGGTGACAAACCGGAGGAAGGTGGGGATGACGTCAAGTCATCATGGCCCTTACGGCCAGGGCTACACACGTACTACAATGGTCGGTACAGAGGGTTGCAAGACCGCGAGGTGGAGCCAATCCCAGAAAGCCGATCCCAGTCCGGATTGGAGTCTGCAACTCGACTCCATGAAGTCGGAATCGCTAGTAATCGCGGATCAGCTATGCCGCGGTGAATACGTTCCCGGGCCTTGTACACACCGCCCGTCACACCATGGGAGTGGGTTGCTCCAGAAGGCGTTAGTCTAACCGCAAGG

>FW510-R7

AGTCGAGCGCCCCGCAAGGGGAGCGGCAGACGGGTGAGTAACGCGTGGGAATCTACCCATCTCTACGGAATAACCCAGGGAAACTTGGACTAATACCGTATACGCCCTTCGGGGGAAAGATTTATCGGAGATGGATGAGCCCGCGTTGGATTAGCTAGTTGGTNGGGTAAAGNCCTACCAAGGCGACGATCCATAGCTGGTCTGAGAGGATGATCAGCCACATTGGGACTGAGACACGGCCCAAACTCCTACGGGAGGCAGCAGTGGGGAATATTGGMCAATGGGCGAAAGCCTGATCCAGCCATGCCGCGTGAGTGATGAAGGCCCTAGGGTTGTAAAGCTCTTTCAACGGTGAAGATAATGACGGTAACCGTAGAAGAAGCCCCGGCTAACTTCGTGCCAGCAGCCGCGGTAATACGAAGGGGGCTAGCGTTGTTCGGAATTACTGGGCGTAAAGCGCACGTAGGCGGATACTTAAGTTAGGGGTGAAATCCCGGGGCTCAACCCCGGAACTGCCTTTAATACTGGGTATCTCGAGTTCGAGAGAGGTGAGTGGAATTCCGAGTGTAGAGGTGAAATTCGTAGATATTCGGAGGAACACCAGTGGCGAAGGCGGCTCACTGGCTCGATACTGACGCTGAGGTGCGAAAGCGTGGGGAGCAAACAGGATTAGATACCCTGGTAGTCCACGCCGTAAACTATGAGAGCTAGCCGTCGGCAAGTTTACTTGTCGGTGGCGCAGCTAACGCATTAAGCTCTCCGCCTGGGGAGTACGGTCGCAAGATTAAAACTCAAAGGAATTGACGGGGGCCCGCACAAGCGGTGGAGCATGTGGTTTAATTCGAAGCAACGCGCAGAACCTTACCAGCCCTTGACATCCCGGTCGCGGTTTCCAGAGATGGATCCCTTCAGTTCGGCTGGACCGGTGACAGGTGCTGCATGGCTGTCGTCAGCTCGTGTCGTGAGATGTTGGGTTAAGTCCCGCAACGAGCGCAACCCTCGCCCTTAGTTGCCAGCATTCAGTTGGGCACTCTAAGGGGACTGCCGGTGATAAGCCGAGAGGAAGGTGGGGATGACGTCAAGTCCTCATGGCCCTTACGGGCTGGGCTACACACGTGCTACAATGGTGGTGACAGTGGGCAGCGAGACCGCNAGGTCRAGCTAATCTCCAAAAGCCATCTCAGTTCGGATTGCACTCTGCAACTCGAGTGCATGAAGTTGGAATCGCTAGTAATCGCAGATCAGCATGCTGCGGTGAATACGTTCCCGGGCCTTGTACACACCGCCCGTCACACCATGGGAGTTGGTTTTACCCGAAGGCGCTGCGCCAACCCGCAAGGAAGCAGGCGACC

>FW507-F4

GTTAAGCTACCTACTTCTGGTGAAACCCACTCCCATGGNGTGACGGGCGGNGTGNACAANANCCGGGAACGTATTCACCGCANCNTGNTGATCTGCGATTACTANCGATTCCGACTTCACNCANTCGAGTTGCAGACTGCGATCCGGACTACNATCGGCTTTNANGGATTANCTCCACCTCGCGGTTTGGCAACCCTCTGTACCGACCNNNNNNTGACGTGTGAAGCCCTACCCATAAKGGCCATGAGGACTTGACGTCATCCCCACCTNNNNNCGGTTTGTCACCGGCANTCTCGCTAAAGTGCCCAACTTAATGNNGGCAATTAGCGACAAGGGTKGCGCTCGTTGCGGGACTNAACCCAACATCTCACGACACGAGNTGACGACAGCCATGCAGCACCTGTGTCCTGGCTNCCGAAGGCACCAANTGATCTCTCACAAGTTCCAGGCATGTCAAGGGTAGGTAAGGTTTTTNSGTTGCATCGAATTAATCCACATCATCCACNGCTTGTGCGGGTNCCCGTCAATTCCNTTGAGTTTTAACCNTGCGGCCGTACTCCCCAGGCGGTCNACTTCACGCGTTANNTGCGTTACTCAGAAAGTTACCCTTCCGAACAACTAGTCGACATCGTTTAGGGCGTGGACTACCAGGGTATCTAATCCTGTTTGCTCCCCACGCTTTCGTGCATGAGCGTCAGTATCGGNCCAGGGGGCTGCCTTCGCCATCGGTGTTCCTCCACATATCTACGCATTTCACTGCTACACGTGGAATTCCACCCCCCTCTGCCGTACTCTANCCGTGCAGTCACAAGNGCAGTTCCCAGGTTAAGCCNNGNGATTTCACACCTGTCNTACACAACNGCCTGCGCACGCTTTACGCCCNGTAATTCCGATTAACGCTCGCACCCTACGTATTACCGCGGCTGCTGGCANGTAGTTAGCCGGTGCTTCTTCTGACAGTACCGTCATCCGCCTCCCGTATTAGGAGAAGCNTTTTCTTTCCGTCTGAAAGAGCTTTACAACCCGAAGGCNTTCTTCACTCACGCGGCATNGNTGGATCANNNNTGCCCCCATNGTCCAAAATTCCCCANTGCTGCCTCCGGNNGGAGTNTGGGCCGTGTNTCAGTCCCAGTGNGGNCGGATCATCCTNTCAGACCCGNTACSGATCGTCGCCNNGGTAGGCCTTTACCCCNCCAANTAGNTAATCNGACATCGGCCGCTCCAATCACGNGAGGTCTTGCGATCCCCCGCTTTCCCCCTCAGGGNGTATGCGGTATNAGNTACGCTTTCGNGTAGTTATCCCCCATGACNGGGTACGTTCNGATGCATTACTCACCCGTTCGCCACTCGCCGGNGGGCNGAAGCCCCCGC

>FW300-N2A3

ACCGTCCTCCCGAAGGNTAGACTAGCTACTTCTGGTGCAACCCACTCCCATGGTGTGACGGGCGGTGTGTACAAGGCCCGGGAACGTATTCACCGCGACATTCTGATTCGCGATTACTAGCGATTCCGACTTCACGCAGTCGAGTTGCAGACTGCGATCCGGACTACGATCGGTTTTATGGGATTAGCTCCACCTCGCGGCTTGGCAACCCTCTGTACCRACCATTGTAGCACGTGTGTNNNCCAGGCCGTAAGGGCCMTGATGACTTSACGTCATCYCCACCTTCCTCCGGTTTGTCACCGGCAGTCTCCTTAGAGTGCCCACNNTAACGTGCTGGTAACNAAGGACAANGGTTGCGCTCGTNNMGGGACTTAACCCAACATCTCACGACACGAGCTGANGACAGCCATGCAGCNCCTGTCTCAATGTNNNCGAAGGCACCAWTCNNNCTCTGGAAAGTNNATTGGATGTCAANGCCTGGTAAGGTNCTTCGNGTTGCTTCGAATTAAACCACATGCTCCACCGCTTGTGCGGNNCCCCGTCAATTCATTTGAGTTTTAACCTTGCGGNCGTACTCCCCAGGCGGTCAACTTAATGCGTTAGCTGCGCCACTAAGAGCTCAAGGCTCCCAACGGCTAGTTGACATCGTTTACGGCGTGGACTACCAGGNGTATCTAATCCTGTTTGCTCCCCACGCTTTCGCACCTCAGTGTCAGTATCAGTCCANGGTGGTCGCCTTCGNCCACTGGTGTTCCTTCCNTATATCTACGCATTTCACCGCTACACAGGNAAATTCCANCCACCCTCTACCATACTCTAGCTCGACAGTTTTGAATGCAGTTCCCNGGTTGAGCCCGGGGATTTCACATCCAACTTAACGAACCACCTACGCGCGCTTTACGCCCAGTAATTCCGATTAACGCTTGCACCCTCTGTATTACCGCGGCTGCTGGCACAGAGTTAGNCGGTGCTTANTCTGTCGGTAACGTCAAAACCATCACGTATTAGGNAAKGNCCNTTCCTCCCAANNTWAAGTGCTTTACATNCGA

>FW300-N2E3

TGCAGTCGAGCGGTAGAGAGAAGCTTGCTTCTCTTGAGAGCGGCGGACGGGTGAGTAATGCCTAGGAATCTGCCTGGTAGTGGGGGATAACGTTCGGAAACGAACGCTAATACCGCATACGTCCTACGGGAGAAAGCAGGGGACCTTCGGGCCTTGCGCTATCAGATGAGCCTAGGTCGGATTAGCTAGTTGGTGGGGTAATGGCTCACCAAGGCGACGATCCGTAACTGGTCTGAGAGGATGATCAGTCACACTGGAACTGAGACACGGTCCAGACTCCTACGGGAGGCAGCAGTGGGGAATATTGGACAATGGGCGAAAGCCTGATCCAGCCATGCCGCGTGTGTGAAGAAGGTCTTCGGATTGTAAAGCACTTTAAGTTGGGAGGAAGGGCCATTACCTAATACGTGATGGTTTTGACGTTACCGACAGAATAAGCACCGGCTAACTCTGTGCCAGCAGCCGCGGTAATACAGAGGGTGCAAGCGTTAATCGGAATTACTGGGCGTAAAGCGCGCGTAGGTGGTTCGTTAAGTTGGATGTGAAATCCCCGGGCTCAACCTGGGAACTGCATTCAAAACTGTCGAGCTAGAGTATGGTAGAGGGTGGTGGAATTTCCTGTGTAGCGGTGAAATGCGTAGATATAGGAAGGAACACCAGTGGCGAAGGCGACCACCTGGACTGATACTGACACTGAGGTGCGAAAGCGTGGGGAGCAAACAGGATTAGATACCCTGGTAGTCCACGCCGTAAACGATGTCAACTAGCCGTTGGGAGCCTTGAGCTCTTAGTGGCGCAGCTAACGCATTAAGTTGACCGCCTGGGGAGTACGGCCGCAAGGTTAAAACTCAAATGAATTGACGGGGGCCCGCACAAGCGGTGGAGCATGTGGTTTAATTCGAAGCAACGCGAMGAAMCTTACCAGGCCWTGACATCCAATGAACTTTCCAGAGATGGATTGGTGCCTTCGGGAACATTGAGACAGGTGCTGCATGGCTGTCGTCAGCTCGTGTCGTGAGATGTTGGGTTAAGTCCCGTAACGAGCGCAACCCTTGTCCTTAGTTACCAGCACGTTATGGTGGGCACTCTAAGGAGACTGCCGGTGACAAACCGGAGGAAGGTGGGGATGACGTCAAGTCATCATGGCCCTTACGGCCTGGGCTACACACGTGCTACAATGGTCGGTACAGAGGGTTGCCAAGCCGCGAGGTGGAGCTAATCCCATAAAACCGATCGTAGTCCGGATCGCAGTCTGCAACTCGACTGCGTGAAGTCGGAATCGCTAGTAATCGCGAATCAGAATGTCGCGGTGAATACGTTCCCGGGCCTTGTACACACCGCCCGTCACACCATGGGAGTGGGTTGCACCAGAAGTAGCTAGTCTAACCTTCGGGAGGACGGT

>FW507-F1

CATGCAGTCGAACGGTAACAGGGGCTTCGGCCCGCTGACGAGTGGCGAACGGGTGAGTAATGCGTCGGAACGCACCGAGTAATGGGGGATAACGCAGCGAAAGCTGTGCTAATACCGCATACGCTCCGAGGAGGAAAGCAGGGGATCGTAAGACCTTGCGTTATTCGAGCGGCCGACGTCTGATTAGCTAGTTGGTGGGGTAAAGGCCCACCAAGGCGACGATCAGTAGCGGGTCTGAGAGGATGATCCGCCACACTGGGACTGAGACACGGCCCAGACTCCTACGGGAGGCAGCAGTGGGGAATTTTGGACAATGGGCGCAAGCCTGATCCAGCCATGCCGCGTGTCTGAAGAAGGCCTTCGGGTTGTAAAGGACTTTTGTCCGGGAGCAAATGCCGGTGGCTAATATCCACTGGAGCTGAGAGTACCGGAAGAATAAGCACCGGCTAACTACGTGCCAGCAGCCGCGGTAATACGTAGGGTGCAAGCGTTAATCGGAATTACTGGGCGTAAAGCGTGCGCAGGCGGTTGTGTAAGTCTGATGTGAAAGCCCCGGGCTCAACCTGGGAACGGCATTGGAGACTGCACAGCTAGAGTGCGTCAGAGGGGGGTAGAATTCCACGTGTAGCAGTGAAATGCGTAGAGATGTGGAGGAATACCGATGGCGAAGGCAGCCCCCTGGGATGACACTGACGCTCATGCACGAAAGCGTGGGGAGCAAACAGGATTAGATACCCTGGTAGTCCACGCCCTAAACGATGTCAATTAGCTGTTGGGGGTTTGAATCCTTGGTAGCGTAGCTAACGCGTGAAATTGACCGCCTGGGGAGTACGGCCGCAAGGTTAAAACTCAAAGGAATTGACGGGGACCCGCACAAGCGGTGGATGATGTGGATTAATTCGATGCAACGCGAAAAACCTTACCTGCTCTTGACATGTCCGGAACCTCGCAGAGACGCGAGGGTGCCCGAAAGGGAGCCGGAACACAGGTGCTGCATGGCTGTCGTCAGCTCGTGTCGTGAGATGTTGGGTTAAGTCCCGCAACGAGCGCAACCCTTGTCATTAGTTGCCATCATTTAGTTGGGCACTCTAATGAGACTGCCGGTGACAAACCGGAGGAAGGTGGGGATGACGTCAAGTCCTCATGGCCCTTATGAGCAGGGCTTCACACGTCATACAATGGTCGGTACAGAGGGTCGCCAAGCCGCGAGGTGGAGCCAATCTCATAAAACCGATCGTAGTCCGGATCGCACTCTGCAACTCGAGTGCGTGAAGTCGGAATCGCTAGTAATCGCAGATCAGCATGCTGCGGTGAATACGTTCCCGGGTCTTGTACACACCGCCCGTCACACCATGGGAGTGGGGGATACCAGAAGTGGCTAGGATANCCTTCGGGAGTCCGGT

>FW507-E7

TGCAGTCGAACGGTAACAGCACTTCGGTGGCTGACGAGTGGCGAACGGGTGAGTAATACATCGGAACGTGCCCGAGAGTGGGGGATAACGCAGCGAAAGCTGTGCTAATACCGCATACGATCTACGGATGAAAGCAGGGGACCGCAAGGCCTTGCGCTCATGGAGCGGCCGATGGCAGATTAGGTAGTTGGTGGGATAAAAGCTTACCAAGCCGACGATCTGTAGCTGGTCTGAGAGGACGACCAGCCACACTGGGACTGAGACACGGCCCAGACTCCTACGGGAGGCAGCAGTGGGGAATTTTGGACAATGGGCGAAAGCCTGATCCAGCCATGCCGCGTGCAGGATGAAGGCCTTCGGGTTGTAAACTGCTTTTGTACGGAACGAAAAGGTCTTTTCTAATAAAGAAGGCTCATGACGGTACCGTAAGAATAAGCACCGGCTAACTACGTGCCAGCAGCCGCGGTAATACGTAGGGTGCAAGCGTTAATCGGAATTACTGGGCGTAAAGCGTGCGCAGGCGGTTATATAAGACAGATGTGAAATCCCCGGGCTCAACCTGGGAACTGCATTAGTGACTGTATAGCTAGAGTGCGGCAGAGGGGGATGGAATTCCGCGTGTAGCAGTGAAATGCGTAGATATGCGGAGGAACACCGATGGCGAAGGCAATCCCCTGGGCCTGCACTGACGCTCATGCACGAAAGCGTGGGGAGCAAACAGGATTAGATACCCTGGTAGTCCACGCCCTAAACGATGTCAACTGGTTGTTGGGAATTTACTTTCTCAGTAACGAAGCTAACGCGTGAAGTTGACCGCCTGGGGAGTACGGCCGCAAGGTTAAAACTCAAAGGAATTGACGGGGACCCGCACAAGCGGTGGATGATGTGGTTTAATTCGATGCAACGCGAAAAACCTTACCCACCTTTGACATGTATGGAAGATCGCAGAGACGCGATTGTGCTCGAAAGAGAGCCATAACACAGGTGCTGCATGGCTGTCGTCAGCTCGTGTCGTGAGATGTTGGGTTAAGTCCCGCAACGAGCGCAACCCTTGTCATTAGTTGCTACATTTAGTTGGGCACTCTAATGAGACTGCCGGTGACAAACCGGAGGAAGGTGGGGATGACGTCAAGTCCTCATGGCCCTTATAGGTGGGGCTACACACGTCATACAATGGCTGGTACAAAGGGTTGCCAACCCGCGAGGGGGAGCCAATCCCATAAAGCCAGTCGTAGTCCGGATCGCAGTCTGCAACTCGACTGCGTGAAGTCGGAATCGCTAGTAATCGTGGATCAGAATGTCACGGTGAATACGTTCCCGGGTCTTGTACACACCGCCCGTCACACCATGGGAGCGGGTTCTGCCAGAAGTAGGTAGCCTAACCGCAAGGAGGGCGCT

>FW300-N2E2

TGCAAGTCGAGCGGTAGAGAGAAGCTTGCTTCTCTTGAGAGCGGCGGACGGGTGAGTAATGCCTAGGAATCTGCCTGGTAGTGGGGGATAACGCTCGGAAACGGACGCTAATACCGCATACGTCCTACGGGAGAAAGCAGGGGACCTTCGGGCCTTGCGCTATCAGATGAGCCTAGGTCGGATTAGCTAGTTGGTGAGGTAATGGCTCACCAAGGCGACGATCCGTAACTGGTCTGAGAGGATGATCAGTCACACTGGAACTGAGACACGGTCCAGACTCCTACGGGAGGCAGCAGTGGGGAATATTGGACAATGGGCGAAAGCCTGATCCAGCCATGCCGCGTGTGTGAAGAAGGTCTTCGGATTGTAAAGCACTTTAAGTTGGGAGGAAGGGCATTAACCTAATACGTTAGTGTTTTGACGTTACCGACAGAATAAGCACCGGCTAACTCTGTGCCAGCAGCCGCGGTAATACAGAGGGTGCAAGCGTTAATCGGAATTACTGGGCGTAAAGCGCGCGTAGGTGGTTCGTTAAGTTGGATGTGAAAGCCCCGGGCTCAACCTGGGAACTGCATTCAAAACTGTCGAGCTAGAGTATGGTAGAGGGTGGTGGAATTTCCTGTGTAGCGGTGAAATGCGTAGATATAGGAAGGAACACCAGTGGCGAAGGCGACCACCTGGACTGATACTGACACTGAGGTGCGAAAGCGTGGGGAGCAAACAGGATTAGATACCCTGGTAGTCCACGCCGTAAACGATGTCAACTAGCCGTTGGGAGCCTTGAGCTCTTAGTGGCGCAGCTAACGCATTAAGTTGACCGCCTGGGGAGTACGGCCGCAAGGTTAAAACTCAAATGAATTGACGGGGGCCCGCACAAGCGGTGGAGCATGTGGTTTAATTCGAAGCAACGCGAAGAACCTTACCAGGCCTTGACATCCAATGAACTTTCCAGAGATGGATTGGTGCCTTCGGGAACATTGAGACAGGTGCTGCATGGCTGTCGTCAGCTCGTGTCGTGAGATGTTGGGTTAAGTCCCGTAACGAGCGCAACCCTTGTCCTTAGTTACCAGCACGTTATGGTGGGCACTCTAAGGAGACTGCCGGTGACAAACCGGAGGAAGGTGGGGATGACGTCAAGTCATCATGGCCCTTACGGCCTGGGCTACACACGTGCTACAATGGTCGGTACAGAGGGTTGCCAAGCCGCGAGGTGGAGCTAATCCCACAAAACCGATCGTAGTCCGGATCGCAGTCTGCAACTCGACTGCGTGAAGTCGGAATCGCTAGTAATCGCGAATCAGAATGTCGCGGTGAATACGTTCCCGGGCCTTGTACACACCGCCCGTCACACCATGGGAGTGGGTTGCACCAGAAGTAGCTAGTCTAACCNTCGGGGNGACGGT

>FW300-N2A6

TGCAGTCGAACGGCAGCGCGGGGCAACCTGGCGGCGAGTGGCGAACGGGTGAGTAATATATCGGAACGTACCCTGGAGTGGGGGATAACGTAGCGAAAGTTACGCTAATACCGCATACGATCTAAGGATGAAAGCAGGGGACCGCAAGGCCTTGTGCTCCTGGAGCGGCCGATATCTGATTAGCTAGTTGGTGGGGTAAAAGCCNACCAAGGCATCGATCAGTAGCTGGTCTGAGAGGACGACCAGCCACACTGGAACTGAGACACGGTCCAGACTCCTACGGGAGGCAGCAGTGGGGAATTTTGGACAATGGGCGCAAGCCTGATCCAGCAATGCCGCGTGAGTGAAGAAGGCCTTCGGGTTGTAAAGCTCTTTTGTCAGGGAAGAAACGGCCTGTTCTAATACAGTGGGCTAATGACGGTACCTGAAGAATAAGCACCGGCTAACTACGTGCCAGCAGCCGCGGTAATACGTAGGGTGCAAGCGTTAATCGGAATTACTGGGCGTAAAGCGTGCGCAGGCGGTTTTGTAAGACTGTCGTGAAATCCCCGGGCTCAACCTGGGAATGGCGATGGTGACTGCAAGGCTAGAGTTTGGCAGAGGGGGGTAGAATTCCACGTGTAGCAGTGAAATGCGTAGATATGTGGAGGAACACCGATGGCGAAGGCAGCCCCCTGGGTCAAAACTGACGCTCATGCACGAAAGCGTGGGGAGCAAACAGGATTAGATACCCTGGTAGTCCACGCCCTAAACGATGTCTACTAGTTGTCGGGTCTTAATTGACTTGGTAACGCAGCTAACGCGTGAAGTAGACCGCCTGGGGAGTACGGTCGCAAGATTAAAACTCAAAGGAATTGACGGGGACCCGCACAAGCGGTGGATGATGTGGATTAATTCGATGCAACGCGAAAAACCTTACCTACCCTTGACATGGCTGGAATCCCCGAGAGATTGGGGAGTGCTCGAAAGAGAACCAGTACACAGGTGCTGCATGGCTGTCGTCAGCTCGTGTCGTGAGATGTTGGGTTAAGTCCCGCAACGAGCGCAACCCTTGTCATTAGTTGCTACGAAAGGGCACTCTAATGAGACTGCCGGTGACAAACCGGAGGAAGGTGGGGATGACGTCAAGTCCTCATGGCCCTTATGGGTAGGGCTTCACACGTCATACAATGGTACATACAGAGGGCCGCCAACCCGCGAGGGGGAGCTAATCCCAGAAAGTGTATCGTAGTCCGGATTGTAGTCTGCAACTCGACTGCATGAAGTTGGAATCGCTAGTAATCGCGGATCAGCATGTCGCGGTGAATACGTTCCCGGGTCTTGTACACACCGCCCGTCACACCATGGGAGCGGGTTTTACCAGAAGTAGGTAGCTTAACCGCAAGGAGGGCGCT

>FW300-N2C2

TGCAGTCGAGCGGTAGAGAGAAGCTTGCTTCTCTTGAGAGCGGCGGACGGGTGAGTAATGCCTAGGAATCTGCCTGGTAGTGGGGGATAACGCTCGGAAACGGACGCTAATACCGCATACGTCCTACGGGAGAAAGCAGGGGACCTTCGGGCCTTGCGCTATCAGATGAGCCTAGGTCGGATTAGCTAGTTGGTGAGGTAATGGCTCACCAAGGCGACGATCCGTAACTGGTCTGAGAGGATGATCAGTCACACTGGAACTGAGACACGGTCCAGACTCCTACGGGAGGCAGCAGTGGGGAATATTGGACAATGGGCGAAAGCCTGATCCAGCCATGCCGCGTGTGTGAAGAAGGTCTTCGGATTGTAAAGCACTTTAAGTTGGGAGGAAGGGCANTAAATTAATACTTTGCTGTTTTGACGTTACCGACAGAATAAGCACCGGCTAACTCTGTGCCAGCAGCCGCGGTAATACAGAGGGTGCAAGCGTTAATCGGAATTACTGGGCGTAAAGCGCGCGTAGGTGGTTTGTTAAGTTGGATGTGAAATCCCCGGGCTCAACCTGGGAACTGCATTCAAAACTGACAAGCTAGAGTATGGTAGAGGGTGGTGGAATTTCCTGTGTAGCGGTGAAATGCGTAGATATAGGAAGGAACACCAGTGGCGAAGGCGACCACCTGGACTGATACTGACACTGAGGTGCGAAAGCGTGGGGAGCAAACAGGATTAGATACCCTGGTAGTCCACGCCGTAAACGATGTCAACTAGCCGTTGGGAGCCTTGAGCTCTTAGTGGCGCAGCTAACGCATTAAGTTGACCGCCTGGGGAGTACGGCCGCAAGGTTAAAACTCAAATGAATTGACGGGGGCCCGCACAAGCGGTGGAGCATGTGGTTTAATTCGAAGCAACGCGAAGAACCTTACCAGGCCTTGACATCCAATGAACTTTCCAGAGATGGATTGGTGCCTTCGGGAACATTGAGACAGGTGCTGCATGGCTGTCGTCAGCTCGTGTCGTGAGATGTTGGGTTAAGTCCCGTAACGAGCGCAACCCTTGTCCTTAGTTACCAGCACGTAATGGTGGGCACTCTAAGGAGACTGCCGGTGACAAACCGGAGGAAGGTGGGGATGACGTCAAGTCATCATGGCCCTTACGGCCTGGGCTACACACGTGCTACAATGGTCGGTACAGAGGGTTGCCAAGCCGCGAGGTGGAGCTAATCCCATAAAACCGATCGTAGTCCGGATCGCAGTCTGCAACTCGACTGCGTGAAGTCGGAATCGCTAGTAATCGCGAATCAGAATGTCGCGGTGAATACGTTCCCGGGCCTTGTACACACCGCCCGTCACACCATGGGAGTGGGTTGCACCAGAAGTAGCTAGTCTAACCTTCGGGAGGACGGT

>FW300-N1A5

TAGACTAGCTACTTCTGGTGCAACCCACTCCCATGGTGTGACGGGCGGTGTGTACAAGGCCCGGGAACGTATTCACCGCGACATTCTGATTCGCGATTACTAGCGATTCCGACTTCACGCAGTCGAGTTGCAGACTGCGATCCGGACTACGATCGGTTTTCTGGGATTAGCTCCACCTCGCGGCTTGGCAACCCTCTGTACCGACCATTGTAGCACGTGTGTAGCCCAGGCCGTAAGGGCCATGATGACTTGACGTCATCCCCACCTTCCTCCGGTTTGTCACCGGCAGTCTCCTTAGAGTGCCCACCATAACGTGCTGGTAACTAAGGACAAGGGTTGCGCTCGTTACGGGACTTAACCCAACATCTCACGACACGAGCTGACGACAGCCATGCAGCACCTGTCTCAATGTTCCCGAAGGCACCAATCCATCTCTGGAAAGTTCATTGGATGTCAAGGCCTGGTAAGGTTCTTCGCGTTGCTTCGAATTAAACCACATGCTCCACCGCTTGTGCGGGCCCCCGTCAATTCATTTGAGTTTTAACCTTGCGGCCGTACTCCCCAGGCGGTCAACTTAATGCGTTAGCTGCGCCACTAAGAGCTCAAGGCTCCCAACGGCTAGTTGACATCGTTTACGGCGTGGACTACCAGGGTATCTAATCCTGTTTGCTCCCCACGCTTTCGCACCTCAGTGTCAGTATCAGTCCAGGTGGTCGCCTTCGCCACTGGTGTTCCTTCCTATATCTACGCATTTCACCGCTACACAGGAAATTCCACCACCCTCTACCATACTCTAGCTCGTCAGTTTTGAATGCAGTTCCCNGGTTGAGCCCGGGGATTTCACATCCAACTTAACGAACCACCTACGCGCGCTTTACGCCCAGTAATTCCGATTAACGCTTGCACCCTCTGTATTACCGCGGCTGCTGGCACAGAGTTAGCCGGTGCTTATTCTGTCGGTAACGTCAAAACAATTACGTATTAGGTAANTGCCCTTCCTCCCAACTTAAAGTGCTTTACAATCCGAAGACCTTCTTCACACACGCGGCATGGCTGGATCAGGCTTTCGCCCATTGTCCAATATTCCCCACTGCTGCCTCCCGTAGGAGTCTGGACCGTGTCTCAGTTCCAGTGTGACTGATCATCCTCTCAGACCAGTTACGGATCGTCGCCTTGGTGAGCCATTACCTCACCAACTAGCTAATCCGACCTAGGCTCATCTGATAGCGCAAGGCCCGAAGGTCCCCTGCTTTCTCCCGTAGGACGTATGCGGTATTAGCGTCCGTTTCCGAGCGTTATCCCCCACTACCAGGCAGATTCCTAGGCATTACTCACCCGTCCGCCGCTCTCAAGAGGTGCAAGCACCTCTCTACCGCTCGACTGCA

>FW507-AN12LB

CTAANAGGNTAGCTAACCGGCTTCGGGCGCCCCCAACTTCCATGGTGTGACGGGCGGTGTGTACAAGACCCGGGAACGCATTCACCGCAGCATTCTGATCTGCGATTACTAGTAACTCCAGCTTCATGTAGGCGAGTTTCAGCCTACAATCCGAACTGAGAATGGCTTTAAGGGATTAGCTCCACCTCGCGGCTTGGCAACCCTCTGTACCACCCATTGTAGCACGTGTGTAGCCCTAAGCATAAGGGGCATGATGATTTGACGTCATCCCCACCTTCCTCCGAGTTATCCTCGGCAGTCCCTCTAGAGTGCCCAACTTAATGCTGGCAACTAAAGGCAAGGGTTGCGCTCGTTGCGGGACTTAACCCAACATCTCACGACACGAGCTGACGACAACCATGCACCACCTGTCACCACTGTCCCCGAAGGGAAATCTCCGATTAGGGAGAGGTCAGTGGGATGTCAAGCTTAGGTAAGGTTCTTCGCGTTGCTTCGAATTAAACCACATGCTCCGCTACTTGTGCGGGTCCCCGTCAATTCCTTTGAGTTTCACTCTTGCGAGCGTACTTCCCAGGCGGAGTACTTAATGCGTTAGCTGCGGCACCGAGGGGGGTAACCCCCGACACCTAGTACTCATCGTTTACGGCGTGGACTACCAGGGTATCTAATCCTGTTTGCTCCCCACGCTTTCGTGCCTCAGTGTCAGTTACAGTCCAGAGAGCCGCCTTCGCTACTGGTATTCCTCCTAATATCTACGCATTTCACCGCTACACTAGGAATTCTACTCTCCTCTCCTGCACTCAAGTTCTCTAGTTTCAAAAGCTTACTACGGTTGAGCCGTAGCCTTTCACTTCTGACTTAAAAAACCACCTACGCACCCTTTACGCCCAGTAATTCCGGATAACGCTAGCCCCCTACGTATTACCGCGGCTGCTGGCACGTAGTTAGCCGGGGCTTCCTCCTCAAGTACCGTCATTATCTTCCTTGAGGACAGAGCTTTACGACCCGAAGGCCTTCATCGCTCACGCGGCGTTGCTGCATCAGGCTTTCGCCCATTGTGCAATATTCCCCACTGCTGCCTCCCGTAGGAGTCTGGACCGTGTCTCAGTTCCAGTGTGGCCGATCACCCTCTCAGGTCGGCTACTGATCGTTGCCTTGGTAAGCCATTACCTTACCAACTAGCTAATCAGACGCGGGTCCATCCTGTACCGCCGGAGCTTTGATACAAAAGCCATGCGACTTTCNTATGTTATCCCGTATTAGTATACCTTTCGGTATGTTATCCGTGTGTACAGGGCAGGTTACCCACGCGTTACTCACCCGTCCGCCGCTCTCTCCGAAGAGATCGCTCGACTGCATG

>FW303-B3

TCGAACGGCAGCACGGGCTTCGGCCTGGTGGCGAGTGGCGAACGGGTGAGTAATACATCGGAACGTGCCCTGTAGTGGGGGATAACTAGTCGAAAGATTAGCTAATACCGCATACGACCTGAGGGTGAAAGCGGGGGACCGCAAGGCCTCGCGCTACAGGAGCGGCCGATGTCTGATTAGCTAGTTGGTGGGGTAAAAGCCTACCAAGGCGACGATCAGTAGCTGGTCTGAGAGGACGATCAGCCACACTGGGACTGAGACACGGCCCAGACTCCTACGGGAGGCAGCAGTGGGGAATTTTGGACAATGGGGGCAACCCTGATCCAGCAATGCCGCGTGTGTGAAGAAGGCCTTCGGGTTGTAAAGCACTTTTGTCCGGAAAGAAATGGCTCTGGTTAATACCCGGGGTCGATGACGGTACCGGAAGAATAAGCACCGGCTAACTACGTGCCAGCAGCCGCGGTAATACGTAGGGTGCGAGCGTTAATCGGAATTACTGGGCGTAAAGCGTGCGCAGGCGGTTTTGTAAGACAGGCGTGAAATCCCCGAGCTCAACTTGGGAATGGCGCTTGTGACTGCAAGGCTAGAGTATGTCAGAGGGGGGTAGAATTCCACGTGTAGCAGTGAAATGCGTAGAGATGTGGAGGAATACCGATGGCGAAGGCAGCCCCCTGGGACGTCACTGACGCTCATGCACGAAAGCGTGGGGAGCAAACAGGATTAGATACCCTGGTAGTCCACGCCCTAAACGATGTCAACTAGTTGTTGGGGATTCATTTCTTCAGTAACGTAGCTAACGCGTGAAGTTGACCGCCTGGGGAGTACGGTCGCAAGATTAAAACTCAAAGGAATTGACGGGGACCCGCACAAGCGGTGGATGATGTGGATTAATTCGATGCAACGCGAAAAACCTTACCTACCCTTGACATGCCACTAACGAAGCAGAGATGCATCAGGTGCCCGAAAGGGAAAGTGGACACAGGTGCTGCATGGCTGTCGTCAGCTCGTGTCGTGAGATGTTGGGTTAAGTCCCGCAACGAGCGCAACCCTTGTCTCTAGTTGCTACGAAAGGGCACTCTAGAGAGACTGCCGGTGACAAACCGGAGGAAGGTGGGGATGACGTCAAGTCCTCATGGCCCTTATGGGTAGGGCTTCACACGTCATACAATGGTGCGTACAGAGGGTTGCCAACCCGCGAGGGGGAGCTAATCCCAGAAAACGCATCGTAGTCCGGATCGTAGTCTGCAACTCGACTACGTGAAGCTGGAATCGCTAGTAATCGCGGATCAGCATGCCGCGGTGAATACGTTCCCGGGTCTTGTACACACCGCCCGTCACACCATGGGAGTGGGTTTTGCCAGAAGTAGTTAGNCTAACCGCAAGGAGGGCGAT

>FW300-N1A1

TGCAAGTCGAGCGGTAGAGAGAAGCTTGCTTCTCTTGAGAGCGGCGGACGGGTGAGTAATGCCTAGGAATCTGCCTGGTAGTGGGGGATAACGCTCGGAAACGGACGCTAATACCGCATACGTCCTACGGGAGAAAGCAGGGGACCTTCGGGCCTTGCGCTATCAGATGAGCCTAGGTCGGATTAGCTAGTTGGTGAGGTAATGGCTCACCAAGGCGACGATCCGTAACTGGTCTGAGAGGATGATCAGTCACACTGGAACTGAGACACGGTCCAGACTCCTACGGGAGGCAGCAGTGGGGAATATTGGACAATGGGCGAAAGCCTGATCCAGCCATGCCGCGTGTGTGAAGAAGGTCTTCGGATTGTAAAGCACTTTAAGTTGGGAGGAAGGGCAGTAAATTAATACTTTGCTGTTTTGACGTTACCGACAGAATAAGCACCGGCTAACTCTGTGCCAGCAGCCGCGGTAATACAGAGGGTGCAAGCGTTAATCGGAATTACTGGGCGTAAAGCGCGCGTAGGTGGTTTGTTAAGTTGGATGTGAAATCCCCGGGCTCAACCTGGGAACTGCATTCAAAACTGACAAGCTAGAGTATGGTAGAGGGTGGTGGAATTTCCTGTGTAGCGGTGAAATGCGTAGATATAGGAAGGAACACCAGTGGCGAAGGCGACCACCTGGACTGATACTGACACTGAGGTGCGAAAGCGTGGGGAGCAAACAGGATTAGATACCCTGGTAGTCCACGCCGTAAACGATGTCAACTAGCCGTTGGGAGCCTTGAGCTCTTAGTGGCGCAGCTAACGCATTAAGTTGACCGCCTGGGGAGTACGGCCGCAAGGTTAAAACTCAAATGAATTGACGGGGGCCCGCACAAGCGGTGGAGCATGTGGTTTAATTCGAAGCAACGCGAAGAACCTTACCAGGCCTTGACATCCAATGAACTTTCCAGAGATGGATTGGTGCCTTCGGGAACATTGAGACAGGTGCTGCATGGCTGTCGTCAGCTCGTGTCGTGAGATGTTGGGTTAAGTCCCGTAACGAGCGCAACCCTTGTCCTTAGTTACCAGCACGTAATGGTGGGCACTCTAAGGAGACTGCCGGTGACAAACCGGAGGAAGGTGGGGATGACGTCAAGTCATCATGGCCCTTACGGCCTGGGCTACACACGTGCTACAATGGTCGGTACAGAGGGTTGCCAAGCCGCGAGGTGGAGCTAATCCCATAAAACCGATCGTAGTCCGGATCGCAGTCTGCAACTCGACTGCGTGAAGTCGGAATCGCTAGTAATCGCGAATCAGAATGTCGCGGTGAATACGTTCCCGGGCCTTGTACACACCGCCCGTCACACCATGGGAGTGGGTTGCACCAGAAGTAGCTAGTCTAACCTTCGGGGGGACGGT

>FW303-A3

TGCAGTCGANCGGCAGCGCGGGAGCAATCCTGGCGGCGAGTGGCGAACGGGTGAGTAATATATCGGAACGTGCCCTGGAGTGGGGGATAACTAGTCGAAAGACTAGCTAATACCGCATACGATCTACGGATGAAAGTGGGGGATCGCAAGACCTCATGCTCCTGGAGCGGCCGATATCTGATTAGCTAGTTGGTGGGGTAAAAGCCTACCAAGGCGACGATCAGTAGCTGGTCTGAGAGGACGACCAGCCACACTGGGACTGAGACACGGCCCAGACTCCTACGGGAGGCAGCAGTGGGGAATTTTGGACAATGGGGGCAACCCTGATCCAGCAATGCCGCGTGTGTGAAGAAGGCCTTCGGGTTGTAAAGCACTTTTGTTCGGAAAGAAATCGCCTGGGCTAATACCCTGGGTGGATGACGGTACCGGAAGAATAAGCACCGGCTAACTACGTGCCAGCAGCCGCGGTAATACGTAGGGTGCAAGCGTTAATCGGAATTACTGGGCGTAAAGCGTGCGCAGGCGGTTGTGCAAGACAGATGTGAAATCCCCGGGCTTAACCTGGGAACTGCATTTGTGACTGCACGGCTAGAGTGTGTCAGAGGGGGGTAGAATTCCACGTGTAGCAGTGAAATGCGTAGATATGTGGAGGAATACCGATGGCGAAGGCAGCCCCCTGGGATAACACTGACGCTCATGCACGAAAGCGTGGGGAGCAAACAGGATTAGATACCCTGGTAGTCCACGCCCTAAACGATGTCAACTAGTTGTCGGGTCTTAATTGACTTGGTAACGCAGCTAACGCGTGAAGTTGACCGCCTGGGGAGTACGGTCGCAAGATTAAAACTCAAAGGAATTGACGGGGACCCGCACAAGCGGTGGATGATGTGGATTAATTCGATGCAACGCGAAAAACCTTACCTACCCTTGACATGGTCGGAATCCTGGAGAGATCTGGGAGTGCTCGAAAGAGAACCGGCGCACAGGTGCTGCATGGCTGTCGTCAGCTCGTGTCGTGAGATGTTGGGTTAAGTCCCGCAACGAGCGCAACCCTTGTCATTAGTTGCTACGAAAGGGCACTCTAATGAGACTGCCGGTGACAAACCGGAGGAAGGTGGGGATGACGTCAAGTCCTCATGGCCCTTATGGGTAGGGCTTCACACGTCATACAATGGTACATACAGAGGGCCGCCAACCCGCGAGGGGGAGCTAATCCCAGAAAGTGTATCGTAGTCCGGATTGTAGTCTGCAACTCGACTGCATGAAGTTGGAATCGCTAGTAATCGCGGATCAGCATGTCGCGGTGAATACGTTCCCGGGTCTTGTACACACCGCCCGTCACACCATGGGAGCGGGTTTTACCAGAAGTAGGTAGCCTAACCGCAAGGAGGGCGCT

>FW300-N1B4

TGCAGTCGAGCGGTAGAGAGGTGCTTGCACCTCTTGAGAGCGGCGGACGGGTGAGTAATGCCTAGGAATCTGCCTGGTAGTGGGGGATAACGCTCGGAAACGGACGCTAATACCGCATACGTCCTACGGGAGAAAGCAGGGGACCTTCGGGCCTTGCGCTATCAGATGAGCCTAGGTCGGATTAGCTAGTTGGTGAGGTAATGGCTCACCAAGGCGACGATCCGTAACTGGTCTGAGAGGATGATCAGTCACACTGGAACTGAGACACGGTCCAGACTCCTACGGGAGGCAGCAGTGGGGAATATTGGACAATGGGCGAAAGCCTGATCCAGCCATGCCGCGTGTGTGAAGAAGGTCTTCGGATTGTAAAGCACTTTAAGTTGGGAGGAAGGGCAGTTACCTAATACGTAATTGTTTTGACGTTACCGACAGAATAAGCACCGGCTAACTCTGTGCCAGCAGCCGCGGTAATACAGAGGGTGCAAGCGTTAATCGGAATTACTGGGCGTAAAGCGCGCGTAGGTGGTTCGTTAAGTTGGATGTGAAATCCCCGGGCTCAACCTGGGAACTGCATTCAAAACTGACGAGCTAGAGTATGGTAGAGGGTGGTGGAATTTCCTGTGTAGCGGTGAAATGCGTAGATATAGGAAGGAACACCAGTGGCGAAGGCGACCACCTGGACTGATACTGACACTGAGGTGCGAAAGCGTGGGGAGCAAACAGGATTAGATACCCTGGTAGTCCACGCCGTAAACGATGTCAACTAGCCGTTGGGAGCCTTGAGCTCTTAGTGGCGCAGCTAACGCATTAAGTTGACCGCCTNGGGGAGTACGGCCGCAAGGTTAAAACTCAAATGAATTGACGGGGGCCCGCACAAGCGGTGGAGCATGTGGTTTAATTCGAAGCAACGCGAAGAACCTTACCAGGCCTTGACATCCAATGAACTTTCCAGAGATGGATTGGTGCCTTCGGGAACATTGAGACAGGTGCTGCATGGCTGTCGTCAGCTCGTGTCGTGAGATGTTGGGTTAAGTCCCGTAACGAGCGCAACCCTTGTCCTTAGTTACCAGCACGTAATGGTGGGCACTCTAAGGAGACTGCCGGTGACAAACCGGAGGAAGGTGGGGATGACGTCAAGTCATCATGGCCCTTACGGCCTGGGCTACACACGTGCTACAATGGTCGGTACAGAGGGTTGCCAAGCCGCGAGGTGGAGCTAATCCCAGAAAACCGATCGTAGTCCGGATCGCAGTCTGCAACTCGACTGCGTGAAGTCGGAATCGCTAGTAATCGCGAATCAGAATGTCGCGGTGAATACGTTCCCGGGCCTTGTACACACCGCCCGTCACACCATGGGAGTGGGTTGCACCAGAAGTAGCTAGTCTAACCTTCGGGAGGACG

>GW247-27LB

CTTCCTCCCTTGCGGGTTAGAGCACTGCCTTCGGGTGAAACCAACTCCCATGGTGTGACGGGCGGTGTGTACAAGGCCTGGGAACGTATTCACCGCGGCATGCTGATCCGCGATTACTAGCGATTCCGCCTTCATGCTCTCGAGTTGCAGAGAACAATCCGAACTGAGACGGCTTTTGGAGATTAGCTACCCCTCGCGGGGTTGCTGCCCACTGTCACCGCCATTGTAGCACGTGTGTAGCCCAGCGCGTAAGGGCCATGAGGACTTGACGTCATCCCCACCTTCCTCCGGCTTATCACCGGCAGTTTCCTTAGAGTGCCCAACTGAATGATGGCAACTAGGGATGAGGGTTGCGCTCGTTGCGGGACTTAACCCAACATCTCACGACACGAGCTGACGACAGCCATGCAGCACCTGTCACTGATCCAGCCGAACTGAAGGAAAAGATCTCTCTAATCCGCGATCAGGATGTCAAACGCTGGTAAGGTTCTGCGCGTTGCTTCGAATTAAACCACATGCTCCACCGCTTGTGCAGGCCCCCGTCAATTCCTTTGAGTTTTAATCTTGCGACCGTACTCCCCAGGCGGATAACTTAATGCGTTAGCTGCGCCACCCAAACTCTATGAGTCCGGACAGCTAGTTATCATCGTTTACGGCGTGGACTACCAGGGTATCTAATCCTGTTTGCTCCCCACGCTTTCGCACCTCAGCGTCAATACTTGTCCAGTCAGTCGCCTTCGCCACTGGTGTTCTTCCGAATATCTACGAATTTCACCTCTACACTCGGAATTCCACTGACCTCTCCAAGATTCAAGTCTTCCAGTTTCAAAGGCTATTCCGGAGTTGAGCTCCGGGCTTTCACCCCTGACTTAAAAAACCGCCTACGCGCGCTTTACGCCCAGTAATTCCGAACAACGCTAGCTCCCTCCGTATTACCGCGGCTGCTGGCACGGAGTTAGCCGGAGCTTATTCTCCCGGTACTGTCATTATCATCCCGGGTAAAAGAGCTTTACAACCCTAGGGCCTTCATCACTCACGCGGCATTGCTGGATCAGGCTTTCGCCCATTGTCCAATATTCCCCACTGCTGCCTCCCGTAGGAGTCTGGGCCGTGTCTCAGTCCCAGTGTGGCTGATCATCCTCTCAGACCAGCTAAAGATCGTCGCCTTGGTAGGCTTTTACCCCACCAACTAGCTAATCTTACGCGGGCTCATCCTTGGGCGATAAATCTTTGGACCGAAGTCATTATACGGTATTAGCACAAATTTCTCTGAGTTATTCCGTACCCAAGGGCAGATTCCCACGCGTTACGCACCCGTGCGCCACTAACTCCGAAGAGTTCGTNCGACTTGCA

>FW507-E2

TGCAGTCGAACGGTAACAGGGGCTTCGGCCCGCTGACGAGTGGCGAACGGGTGAGTAATGCGTCGGAACGCACCGAGTAATGGGGGATAACGCAGCGAAAGCTGTGCTAATACCGCATACGCTCCGAGGAGGAAAGCAGGGGATCGTAAGACCTTGCGTTATTCGAGCGGCCGACGTCTGATTAGCTAGTTGGTGGGGTAAAGGCCCACCAAGGCGACGATCAGTAGCGGGTCTGAGAGGATGATCCGCCACACTGGGACTGAGACACGGCCCAGACTCCTACGGGAGGCAGCAGTGGGGAATTTTGGACAATGGGCGCAAGCCTGATCCAGCCATGCCGCGTGTCTGAAGAAGGCCTTCGGGTTGTAAAGGACTTTTGTCCGGGAGCAAATGCCGGTGGCTAATATCCACTGGAGCTGAGAGTACCGGAAGAATAAGCACCGGCTAACTACGTGCCAGCAGCCGCGGTAATACGTAGGGTGCAAGCGTTAATCGGAATTACTGGGCGTAAAGCGTGCGCAGGCGGTTGTGTAAGTCTGATGTGAAAGCCCCGGGCTCAACCTGGGAACGGCATTGGAGACTGCACAGCTAGAGTGCGTCAGAGGGGGGTAGAATTCCACGTGTAGCAGTGAAATGCGTAGAGATGTGGAGGAATACCGATGGCGAAGGCAGCCCCCTGGGATGACACTGACGCTCATGCACGAAAGCGTGGGGAGCAAACAGGATTAGATACCCTGGTAGTCCACGCCCTAAACGATGTCAATTAGCTGTTGGGGGTTTGAATCCTTGGTAGCGTAGCTAACGCGTGAAATTGACCGCCTGGGGAGTACGGCCGCAAGGTTAAAACTCAAAGGAATTGACGGGGACCCGCACAAGCGGTGGATGATGTGGATTAATTCGATGCAACGCGAAAAACCTTACCTGCTCTTGACATGTCCGGAACCTCGCAGAGACGCGAGGGTGCCCGAAAGGGAGCCGGAACACAGGTGCTGCATGGCTGTCGTCAGCTCGTGTCGTGAGATGTTGGGTTAAGTCCCGCAACGAGCGCAACCCTTGTCATTAGTTGCCATCATTTAGTTGGGCACTCTAATGAGACTGCCGGTGACAAACCGGAGGAAGGTGGGGATGACGTCAAGTCCTCATGGCCCTTATGAGCAGGGCTTCACACGTCATACAATGGTCGGTACAGAGGGTCGCCAAGCCGCGAGGTGGAGCCAATCTCATAAAACCGATCGTAGTCCGGATCGCACTCTGCAACTCGAGTGCGTGAAGTCGGAATCGCTAGTAATCGCAGATCAGCATGCTGCGGTGAATACGTTCCCGGGTCTTGTACACACCGCCCGTCACACCATGGGAGTGGGGGATACCAGAAGTGGCTAGGATAACCTTCGGGAGTCCGGT

>FW303-C2

TGCAGTCGAACGGCAGCACGGGGGCAACCCTGGTGGCGAGTGGCGAACGGGTGAGTAATACATCGGAACGTGCCCAGTCGTGGGGGATAACGTAGCGAAAGTTACGCTAATACCGCATACGATCTATGGATGAAAGCGGGGGACCGTAAGGCCTCGCGCGATTGGAGCGGCCGATGTCAGATTAGGTAGTTGGTGGGGTAAAGGCTCACCAAGCCGACGATCTGTAGCTGGTCTGAGAGGACGACCAGCCACACTGGGACTGAGACACGGCCCAGACTCCTACGGGAGGCAGCAGTGGGGAATTTTGGACAATGGGCGCAAGCCTGATCCAGCCATTCCGCGTGCAGGATGAAGGCCCTCGGGTTGTAAACTGCTTTTGTACGGAACGAAAAGGCTCTGGTTAATACCCGGGGCTCATGACGGTACCGTAAGAATAAGCACCGGCTAACTACGTGCCAGCAGCCGCGGTAATACGTAGGGTGCAAGCGTTAATCGGAATTACTGGGCGTAAAGCGTGCGCAGGCGGTGATGTAAGACAGATGTGAAATCCCCGGGCTCAACCTGGGAACTGCATTTGTGACTGCATCGCTGGAGTACGGCAGAGGGGGATGGAATTCCGCGTGTAGCAGTGAAATGCGTAGATATGCGGAGGAACACCGATGGCGAAGGCAATCCCCTGGGCCTGTACTGACGCTCATGCACGAAAGCGTGGGGAGCAAACAGGATTAGATACCCTGGTAGTCCACGCCCTAAACGATGTCAACTGGTTGTTGGGTCTTCACTGACTCAGTAACGAAGCTAACGCGTGAAGTTGACCGCCTGGGGAGTACGGCCGCAAGGTTGAAACTCAAAGGAATTGACGGGGACCCGCACAAGCGGTGGATGATGTGGTTTAATTCGATGCAACGCGAAAAACCTTACCCACCTTTGACATGTACGGAAGTTGCCAGAGATGGCTTCGTGCTCGAAAGAGAACCGTAACACAGGTGCTGCATGGCTGTCGTCAGCTCGTGTCGTGAGATGTTGGGTTAAGTCCCGCAACGAGCGCAACCCTTGTCATTAGTTGCTACGAAAGGGCACTCTAATGAGACTGCCGGTGACAAACCGGAGGAAGGTGGGGATGACGTCAAGTCCTCATGGCCCTTATAGGTGGGGCTACACACGTCATACAATGGCTGGTACAGAGGGTTGCCAACCCGCGAGGGGGAGCTAATCCCATAAAACCAGTCGTAGTCCGGATCGCAGTCTGCAACTCGACTGCGTGAAGTCGGAATCGCTAGTAATCGCGGATCAGAATGTCGCGGTGAATACGTTCCCGGGTCTTGTACACACCGCCCGTCACACCATGGGAGCGGGTTCTGCCAGAAGTAGTTAGCCTAACCGCAAGGAGGGCGAT

>FW301-B2

TGCAAGTCGAACGGTAACAGGTTAAGCTGACGAGTGGCGAACGGGTGAGTAATGTATCGGAACGTGCCCAGTTGTGGGGGATAACTACTCGAAAGAGTGGCTAATACCGCATACGACCTGAGGGTGAAAGCGGGGGATCGCAAGACCTCGCGCAATTGGAGCGGCCGATATCAGATTAGGTAGTTGGTGGGGTAAAGGCTCACCAAGCCAACGATCTGTAGCTGGTCTGAGAGGACGACCAGCCACACTGGGACTGAGACACGGCCCAGACTCCTACGGGAGGCAGCAGTGGGGAATTTTGGACAATGGACGCAAGTCTGATCCAGCCATGCCGCGTGCGGGAAGAAGGCCTTCGGGTTGTAAACCGCTTTTGTCAGGGAAGAAACGGCTTTCTCTAATACAGAGGGCTAATGACGGTACCTGAAGAATAAGCACCGGCTAACTACGTGCCAGCAGCCGCGGTAATACGTAGGGTGCAAGCGTTAATCGGAATTACTGGGCGTAAAGCGTGCGCAGGCGGTTATGCAAGACAGAGGTGAAATCCCCGGGCTCAACCTGGGAACTGCCTTTGTGACTGCATGGCTAGAGTACGGTAGAGGGGGATGGAATTCCGCGTGTAGCAGTGAAATGCGTAGATATGCGGAGGAACACCGATGGCGAAGGCAATCCCCTGGACCTGTACTGACGCTCATGCACGAAAGCGTGGGGAGCAAACAGGATTAGATACCCTGGTAGTCCACGCCCTAAACGATGTCAACTGGTTGTTGGGAGGGTTTCTTCTCAGTAACGTAGCTAACGCGTGAAGTTGACCGCCTGGGGAGTACGGCCGCAAGGTTGAAACTCAAAGGAATTGACGGGGACCCGCACAAGCGGTGGATGATGTGGTTTAATTCGATGCAACGCGAAAAACCTTACCTACCCTTGACATGTCAGGAATCCTGAAGAGATTTGGGAGTGCTCGAAAGAGAACCTGAACACAGGTGCTGCATGGCCGTCGTCAGCTCGTGTCGTGAGATGTTGGGTTAAGTCCCGCAACGAGCGCAACCCTTGTCATTAGTTGCTACGAAAGGGCACTCTAATGAGACTGCCGGTGACAAACCGGAGGAAGGTGGGGATGACGTCAGGTCATCATGGCCCTTATGGGTAGGGCTACACACGTCATACAATGGCCGGTACAGAGGGCTGCCAACCCGCGAGGGGGAGCTAATCCCAGAAAACCGGTCGTAGTCCGGATCGTAGTCTGCAACTCGACTGCGTGAAGTCGGAATCGCTAGTAATCGCGGATCAGCTTGCCGCGGTGAATACGTTCCCGGGTCTTGTACACACCGCCCGTCACACCATGGGAGCGGGTTCTGCCAGAAGTAGTTAGCCTAACCGCAAGGAGGGCG

>FW300-E2

TGCAGTCGAACGGTAACNGGTTAAGCTGACGAGTGGCGAACGGGTGAGTAATGTATCGGAACGTGCCCAGTTGTGGGGGATAACTACTCGAAAGAGTGGCTAATACCGCATACGACCTGAGGGTGAAAGCGGGGGATCGCAAGACCTCGCGCAATTGGAGCGGCCGATATCAGATTAGGTAGTTGGTGGGGTAAAGGCTCACCAAGCCAACGATCTGTAGCTGGTCTGAGAGGACGACCAGCCACACTGGGACTGAGACACGGCCCAGACTCCTACGGGAGGCAGCAGTGGGGAATTTTGGACAATGGACGCAAGTCTGATCCAGCCATGCCGCGTGCGGGAAGAAGGCCTTCGGGTTGTAAACCGCTTTTGTCAGGGAAGAAACGGCTTTCTCTAATACAGAGGGCTAATGACGGTACCTGAAGAATAAGCACCGGCTAACTACGTGCCAGCAGCCGCGGTAATACGTAGGGTGCAAGCGTTAATCGGAATTACTGGGCGTAAAGCGTGCGCAGGCGGTTATGCAAGACAGAGGTGAAATCCCCGGGCTCAACCTGGGAACTGCCTTTGTGACTGCATGGCTAGAGTACGGTAGAGGGGGATGGAATTCCGCGTGTAGCAGTGAAATGCGTAGATATGCGGAGGAACACCGATGGCGAAGGCAATCCCCTGGACCTGTACTGACGCTCATGCACGAAAGCGTGGGGAGCAAACAGGATTAGATACCCTGGTAGTCCACGCCCTAAACGATGTCAACTGGTTGTTGGGAGGGTTTCTTCTCAGTAACGTAGCTAACGCGTGAAGTTGACCGCCTGGGGAGTACGGCCGCAAGGTTGAAACTCAAAGGAATTGACGGGGACCCGCACAAGCGGTGGATGATGTGGTTTAATTCGATGCAACGCGAAAAACCTTACCTACCCTTGACATGTCAGGAATCCTGAAGAGATTTGGGAGTGCTCGAAAGAGAACCTGAACACAGGTGCTGCATGGCCGTCGTCAGCTCGTGTCGTGAGATGTTGGGTTAAGTCCCGCAACGAGCGCAACCCTTGTCATTAGTTGCTACGAAAGGGCACTCTAATGAGACTGCCGGTGACAAACCGGAGGAAGGTGGGGATGACGTCAGGTCATCATGGCCCTTATGGGTAGGGCTACACACGTCATACAATGGCCGGTACAGAGGGCTGCCAACCCGCGAGGGGGAGCTAATCCCAGAAAACCGGTCGTAGTCCGGATCGTAGTCTGCAACTCGACTGCGTGAAGTCGGAATCGCTAGTAATCGCGGATCAGCTTGCCGCGGTGAATACGTTCCCGGGTCTTGTACACACCGCCCGTCACACCATGGGAGCGGGTTCTGCCAGAAGTAGTTAGCCTAACCGCAAGGAGGGCGAT

>GW247-5R2A

TACCTCACCGACTTCGGGTGTTACAAACTCTCGTGGTGTGACGGGCGGTGTGTACAAGGCCCGGGAACGTATTCACCGCGGCATGCTGATCCGCGATTACTAGCGATTCCGGCTTCATGTAGGCGAGTTGCAGCCTACAATCCGAACTGAGAACGACTTTATCGGATTAGCTCCCTCTCGCGAGTTGGCAACCGTTTGTATCGTCCATTGTAGCACGTGTGTAGCCCAGGTCATAAGGGGCATGATGATTTGACGTCATCCCCACCTTCCTCCGGTTTGTCACCGGCAGTCACCTTAGAGTGCCCAACTAAATGATGGCAACTAAGATCAAGGGTTGCGCTCGTTGCGGGACTTAACCCAACATCTCACGACACGAGCTGACGACAACCATGCACCACCTGTCACCGTTGCCCCCGAAGGGGAAACTATATCTCTACAGTGGTCAACGGGATGTCAAGACCTGGTAAGGTTCTTCGCGTTGCTTCGAATTAAACCACATGCTCCACCGCTTGTGCGGGCCCCCGTCAATTCCTTTGAGTTTCAGTCTTGCGACCGTACTCCCCAGGCGGAGTGCTTAATGCGTTAGCTGCAGCACTAAGGGGCGGAAACCCCCTAACACTTAGCACTCATCGTTTACGGCGTGGACTACCAGGGTATCTAATCCTGTTTGCTCCCCACGCTTTCGCGCCTCAGTGTCAGTTACAGACCAGATAGTCGCCTTCGCCACTGGTGTTCCTCCAAATCTCTACGCATTTCACCGCTACACTTGGAATTCCACTATCCTCTTCTGCACTCAAGTCTCCCAGTTTCCAATGACCCTCCACGGTTGAGCCGTGGGCTTTCACATCAGACTTAAGAAACCACCTGCGCGCGCTTTACGCCCAATAATTCCGGACAACGCTTGCCACCTACGTATTACCGCGGCTGCTGGCACGTAGTTAGCCGTGGCTTTCTAATAAGGTACCGTCAAGGTACAGCCAGTTACTACTGTACTTGTTCTTCCCTTACAACAGAGTTTTACGAACCGAAATCCTTCTTCACTCACGCGGCGTTGCTCCATCAGGCTTTCGCCCATTGTGGAAGATTCCCTACTGCTGCCTCCCGTAGGAGTCTGGGCCGTGTCTCAGTCCCAGTGTGGCCGATCACCCTCTCAGGTCGGCTACGCATCGTCGCCTTGGTGAGCCGTTACCTCACCAACTAGCTAATGCGCCGCGGGCCCATCCTATAGCGACAGCGAGATGCCGTCTTTCAGTGTTTCACCATGAGGTGAAACAGATTATTCGGTATTAGCCCCGGTTTCCCGGAGTTATCCCAAACTATAAGGTAGGTTGCCCACGTGTTACTCACCCGTCCGCCGCTAACGTCGAAGGAGCAAGCTCCTTCTCTGTTCGCTCGACTTGCA

>FW507-AN21R2A

TGCAAGTCGAGCGATGAAGCTCCTTCGGGAGTGGATTAGCGGCGGACGGGTGAGTAACACGTGGGTAACCTGCCTCATAGAGGGGAATAGCCTTCCGAAAGGAAGATTAATACCGCATAAGATTGTAGTTCCGCATGGAATAGCAATTAAAGGAGCAATCCGCTATGAGATGGACCCGCGTCGCATTAGCTAGTTGGTGAGGTAACGGCTCACCAAGGCGACGATGCGTAGCCGACCTGAGAGGGTGATCGGCCACATTGGGACTGAGACACGGCCCAGACTCCTACGGGAGGCAGCAGTGGGGAATATTGCACAATGGGGGAAACCCTGATGCAGCAACGCCGCGTGAGTGATGACGGTCTTCGGATTGTAAAACTCTGTCTTTGGGGACGATAATGACGGTACCCAAGGAGGAAGCCACGGCTAACTACGTGCCAGCAGCCGCGGTAATACGTAGGTGGCAAGCGTTGTCCGGATTTACTGGGCGTAAAGGGAGCGTAGGTGGATATTTAAGTGGGATGTGAAATACTCGGGCTTAACCTGAGTGCTGCATTCCAAACTGGATATCTAGAGTGCAGGAGAGGAAAGTAGAATTCCTAGTGTAGCGGTGAAATGCGTAGAGATTAGGAAGAATACCAGTGGCGAAGGCGACTTTCTGGACTGTAACTGACACTGAGGCTCGAAAGCGTGGGGAGCAAACAGGATTAGATACCCTGGTAGTCCACGCCGTAAACGATGAATACTAGGTGTGGGGGTTGTCATGACCTCCGTGCCGCCGCTAACGCATTAAGTATTCCGCCTGGGGAGTACGGTCGCAAGATTAAAACTCAAAGGAATTGACGGGGGCCCGCACAAGCAGCGGAGCATGTGGTTTAATTCGAAGCAACGCGAAGAACCTTACCTAGACTTGACATCTCCTGAATTACTCTTAATCGAGGAAGTCACTTCGGTGACAGGAAGACAGGTGGTGCATGGTTGTCGTCAGCTCGTGTCGTGAGATGTTGGGTTAAGTCCCGCAACGAGCGCAACCCCTATTGTTAGTTGCTACCATTTAGTTGAGCACTCTAGCGAGACTGCCCGGGTTAACCGGGAGGAAGGTGGGGATGACGTCAAATCATCATGCCCCTTATGTCTAGGGCTACACACGTGCTACAATGGCTGGTACAGAGAGATGCAATACCGTGAGGTGGAGCCAAACTTCAAAACCAGTCTCAGTTCGGATTGTAGGCTGAAACTCGCCTACATGAAGCTGGAGTTGCTAGTAATCGCGAATCAGAATGTCGCGGTGAATACGTTCCCGGGCCTTGTACACACCGCCCGTCACACCATGAGAGTTGGCAATACCCAAAGTTCGTGAGCTAACGCGTAAGCGAGGCAGCGAC

>FW507-AN13LB

TGCAGTCGAGCGANCTCTTCGGAGAGAGCGGCGGACGGGTGAGTAACGCGTGGGTAACCTGCCCTGTACACACGGATAACATACCGAAAGGTATACTAATACGGGATAACATATGAAAGTCGCATGGCTTTTGTATCAAAGCTCCGGCGGTACAGGATGGACCCGCGTCTGATTAGCTAGTTGGTAAGGTAATGGCTTACCAAGGCAACGATCAGTAGCCGACCTGAGAGGGTGATCGGCCACACTGGAACTGAGACACGGTCCAGACTCCTACGGGAGGCAGCAGTGGGGAATATTGCACAATGGGCGAAAGCCTGATGCAGCAACGCCGCGTGAGCGATGAAGGCCTTCGGGTCGTAAAGCTCTGTCCTCAAGGAAGATAATGACGGTACTTGAGGAGGAAGCCCCGGCTAACTACGTGCCAGCAGCCGCGGTAATACGTAGGGGGCTAGCGTTATCCGGAATTACTGGGCGTAAAGGGTGCGTAGGTGGTTTTTTAAGTCAGAAGTGAAAGGCTACGGCTCAACCGTAGTAAGCTTTTGAAACTAGAGAACTTGAGTGCAGGAGAGGAGAGTAGAATTCCTAGTGTAGCGGTGAAATGCGTAGATATTAGGAGGAATACCAGTAGCGAAGGCGGCTCTCTGGACTGTAACTGACACTGAGGCACGAAAGCGTGGGGAGCAAACAGGATTAGATACCCTGGTAGTCCACGCCGTAAACGATGAGTACTAGGTGTCGGGGGTTACCCCCCTCGGTGCCGCAGCTAACGCATTAAGTACTCCGCCTGGGAAGTACGCTCGCAAGAGTGAAACTCAAAGGAATTGACGGGGACCCGCACAAGTAGCGGAGCATGTGGTTTAATTCGAAGCAACGCGAAGAACCTTACCTAAGCTTGACATCCCACTGACCTCTCCCTAATCGGAGATTTCCCTTCGGGGACAGTGGTGACAGGTGGTGCATGGTTGTCGTCAGCTCGTGTCGTGAGATGTTGGGTTAAGTCCCGCAACGAGCGCAACCCTTGCCTTTAGTTGCCAGCATTAAGTTGGGCACTCTAGAGGGACTGCCGAGGATAACTCGGAGGAAGGTGGGGATGACGTCAAATCATCATGCCCCTTATGCTTAGGGCTACACACGTGCTACAATGGGTGGTACAGAGGGTTGCCAAGCCGCGAGGTGGAGCTAATCCCTTAAAGCCATTCTCAGTTCGGATTGTAGGCTGAAACTCGCCTACATGAAGCTGGAGTTACTAGTAATCGCAGATCAGAATGCTGCGGTGAATGCGTTCCCGGGTCTTGTACACACCGCCCGTCACACCATGGAAGTTGGGGGCGCCCGAAGCCGGTTAGCTANCCTTTTAGGAAGCGGCCGTCGA

>FW507-AN11LB

TGCAGTCGAGCGATGAAACCCTTCGGGGTGANTTAGCGGCGGACGGGTGAGTAACACGTGGGTAACCTGCCTCATAGAGGGGGATAGCCTCCCGAAAGGGAGATTAATACCGCATAACATATTTCTGCTGCATGGTAGAAATATCAAAGGAGTAATCCGCTATGAGATGGACCCGCGGCGCATTAGCTAGTTGGTGAGGTAAGGGCTCACCAAGGCGACGATGCGTAGCCGACCTGAGAGGGTGATCGGCCACATTGGAACTGAGACACGGTCCAGACTCCTACGGGAGGCAGCAGTGGGGAATATTGCGCAATGGGGGAAACCCTGACGCAGCAACGCCGCGTGAATGAAGAAGGCCTTAGGGTTGTAAAGTTCTGTTTTCTGGGACGATAATGACGGTACCAGAGGAGGAAGCCACGGCTAACTACGTGCCAGCAGCCGCGGTAATACGTAGGTGGCAAGCGTTGTCCGGATTTACTGGGCGTAAAGGATGCGTAGGCGGATGTTTAAGTGAGATGTGAAATACCCGGGCTCAACTTGGGTGCTGCATTTCAAACTGGATATCTAGAGTGTCGGAGAGGTAAGTGGAATTCCTAGTGTAGCGGTGAAATGCGTAGAGATTAGGAAGAACACCAGTGGCGAAGGCGACTTACTGGACGATAACTGACGCTGAGGCATGAAAGCGTGGGGAGCAAACAGGATTAGATACCCTGGTAGTCCACGCCGTAAACGATGAATACTAGGTGTAGGAGGTATCGACCCCTTCTGTGCCGCAGTTAACACAATAAGTATTCCGCCTGGGGAGTACGATCGCAAGATTAAAACTCAAAGGAATTGACGGGGGCCCGCACAAGCAGCGGAGCATGTGGTTTAATTCGAAGCAACGCGAAGAACCTTACCTAGACTTGACATCTCCTGAATTACCCTTAACCGGGGAAGCCCTTCGGGGCAGGAAGACAGGTGGTGCATGGTTGTCGTCAGCTCGTGTCGTGAGATGTTGGGTTAAGTCCCGCAACGAGCGCAACCCTTATCATTAGTTGCTACCATTAAGTTGAGCACTCTAGTGAGACTGCCAGGGTTAACCTGGAGGAAGGTGGGGATGACGTCAAATCATCATGCCCCTTATGTCTAGGGCTACACACGTGCTACAATGGTGAATACAACGAGATGCAATACCGCGAGGTGGAGCCAAACTCAAAAATTCATCCCAGTTCGGATTGTAGTCTGAAACTCGACTACATGAAGCCGGAGTTGCTAGTAATCGCGAATCAGCATGTCGCGGTGAATACGTTCCCGGGCCTTGTACACACCGCCCGTCACACCATGAGAGCTGGTAACACCCGAAGTCCGTGAGGTAACCGTAAGGAGCCAGCG

>FW507-AN2TSA

TGCAGTCGAGCGAATGGATTAAGAGCTTGCTCTTATGAAGTTAGCGGCGGACGGGTGAGTAACACGTGGGTAACCTGCCCATAAGACTGGGATAACTCCGGGAAACCGGGGCTAATACCGGATAACATTTTGCACCGCATGGTGCGAAATTCAAAGGCGGCTTCGGCTGTCACTTATGGATGGACCCGCGTCGCATTAGCTAGTTGGTGAGGTAACGGCTCACCAAGGCAACGATGCGTAGCCGACCTGAGAGGGTGATCGGCCACACTGGGACTGAGACACGGCCCAGACTCCTACGGGAGGCAGCAGTAGGGAATCTTCCGCAATGGACGAAAGTCTGACGGAGCAACGCCGCGTGAGTGATGAAGGCTTTCGGGTCGTAAAACTCTGTTGTTAGGGAAGAACAAGTGCTAGTTGAATAAGCTGGCACCTTGACGGTACCTAACCAGAAAGCCACGGCTAACTACGTGCCAGCAGCCGCGGTAATACGTAGGTGGCAAGCGTTATCCGGAATTATTGGGCGTAAAGCGCGCGCAGGTGGTTTCTTAAGTCTGATGTGAAAGCCCACGGCTCAACCGTGGAGGGTCATTGGAAACTGGGAGACTTGAGTGCAGAAGAGGAAAGTGGAATTCCATGTGTAGCGGTGAAATGCGTAGAGATATGGAGGAACACCAGTGGCGAAGGCGACTTTCTGGTCTGTAACTGACACTGAGGCGCGAAAGCGTGGGGAGCAAACAGGATTAGATACCCTGGTAGTCCACGCCGTAAACGATGAGTGCTAAGTGTTAGAGGGTTTCCGCCCTTTAGTGCTGAAGTTAACGCATTAAGCACTCCGCCTGGGGAGTACGGCCGCAAGGCTGAAACTCAAAGGAATTGACGGGGGCCCGCACAAGCGGTGGAGCATGTGGTTTAATTCGAAGCAACGCGAAGAACCTTACCAGGTCTTGACATCCTCTGACAACCCTAGAGATAGGGCTTCCCCTTCGGGGGCAGAGTGACAGGTGGTGCATGGTTGTCGTCAGCTCGTGTCGTGAGATGTTGGGTTAAGTCCCGCAACGAGCGCAACCCTTGATCTTAGTTGCCATCATTAAGTTGGGCACTCTAAGGTGACTGCCGGTGACAAACCGGAGGAAGGTGGGGATGACGTCAAATCATCATGCCCCTTATGACCTGGGCTACACACGTGCTACAATGGACGGTACAAAGAGCTGCAAGACCGCGAGGTGGAGCTAATCTCATAAAACCGTTCTCAGTTCGGATTGTAGGCTGCAACTCGCCTACATGAAGCTGGAATCGCTAGTAATCGCGGATCAGCATGCCGCGGTGAATACGTTCCCGGGCCTTGTACACACCGCCCGTCACACCACGAGAGTTTGTAACACCCGAAGTCGGTGGGGTA

>FW507-15TSA

TGCAGTCGAACGGCAGCACGGGAGCAATCCTGGTGGCGAGTGGCGAACGGGTGAGTAATACATCGGAACGTGCCCTGTCGTGGGGGATAACTAGTCGAAAGATTAGCTAATACCGCATACGACCTGAGGGTGAAAGCGGGGGACCGTAAGGCCTCGCGCGATAGGAGCGGCCGATGTCTGATTAGCTAGTTGGTGGGGTAAAGGCCCACCAAGGCGACGATCAGTAGCTGGTCTGAGAGGACGATCAGCCACACTGGGACTGAGACACGGCCCAGACTCCTACGGGAGGCAGCAGTGGGGAATTTTGGACAATGGGGGCAACCCTGATCCAGCAATGCCGCGTGTGTGAAGAAGGCCTTCGGGTTGTAAAGCACTTTTGTCCGGAAAGAAATCCCTTGCTCTAATACAGCGGGGGGATGACGGTACCGGAAGAATAAGCACCGGCTAACTACGTGCCAGCAGCCGCGGTAATACGTAGGGTGCGAGCGTTAATCGGAATTACTGGGCGTAAAGCGTGCGCAGGCGGTTTTGTAAGACAGGCGTGAAATCCCCGAGCTCAACTTGGGAATGGCGCTTGTGACTGCAAGGCTAGAGTATGTCAGAGGGGGGTAGAATTCCACGTGTAGCAGTGAAATGCGTAGAGATGTGGAGGAATACCGATGGCGAAGGCAGCCCCCTGGGACGTCACTGACGCTCATGCACGAAAGCGTGGGGAGCAAACAGGATTAGATACCCTGGTAGTCCACGCCCTAAACGATGTCAACTAGTTGTTGGGGATTCATTTCTTCAGTAACGTAGCTAACGCGTGAAGTTGACCGCCTGGGGAGTACGGTCGCAAGATTAAAACTCAAAGGAATTGACGGGGACCCGCACAAGCGGTGGATGATGTGGATTAATTCGATGCAACGCGAAAAACCTTACCTACCCTTGACATGCCACTAACGAAGCAGAGATGCATTAGGTGCNNGAAAGGGAAAGTGGACACAGGTGCTGCATGGCTGTCGTCAGCTCGTGTCGTGAGATGTTGGGTTAAGTCCCGCAACGAGCGCAACCCTTGTCTTTAGTTGCTACGCAAGAGCACTCTAGAGAGACTGCCGGTGACAAACCGGAGGAAGGTGGGGATGACGTCAAGTCCTCATGGCCCTTATGGGTAGGGCTTCACACGTCATACAATGGTGCGTACAGAGGGTTGCCAACCCGCGAGGGGGAGCTAATCCCAGAAAACGCATCGTAGTCCGGATCGTAGTCTGCAACTCGACTACGTGAAGCTGGAATCGCTAGTAATCGCGGATCAGCATGCCGCGGTGAATACGTTCCCGGGTCTTGTACACACCGCCCGTCACACCATGGGAGTGGGTTTTGCCAGAAGTAGTTAGCCTAACCGCAAGGGGGGCGAT

>FW507-14TSA

GTCGAGCGGANGAAGTGAGCTTGCTCACGGATTTAGCGGCGGACGGGTGAGTAATGCCTAGGAATCTGCCTGGTAGTGGGGGATAACGTTTCGAAAGGGACGCTAATACCGCATACGTCCTACGGGAGAAAGCAGGGGACCTTCGGGCCTTGCGCTATCAGATGAGCCTAGGTCGGATTAGCTAGTTGGTGAGGTAATGGCTCACCAAGGCGACGATCCGTAACTGGTCTGAGAGGATGATCAGTCACACTGGAACTGAGACACGGTCCAGACTCCTACGGGAGGCAGCAGTGGGGAATATTGGACAATGGGCGAAAGCCTGATCCAGCCATGCCGCGTGTGTGAAGAAGGTCTTCGGATTGTAAAGCACTTTAAGTTGGGAGGAAGGGCAGTAAGCTAATATCTTGCTGTTTTGACGTTACCGACAGAATAAGCACCGGCTAACTCTGTGCCAGCAGCCGCGGTAATACAGAGGGTGCAAGCGTTAATCGGAATTACTGGGCGTAAAGCGCGCGTAGGTGGTTTGTTAAGTTGGATGTGAAAGCCCCGGGCTCAACCTGGGAACTGCATCCAAAACTGGCAAGCTAGAGTATGGTAGAGGGTGGTGGAATTTCCTGTGTAGCGGTGAAATGCGTAGATATAGGAAGGAACACCAGTGGCGAAGGCGACCACCTGGACTGATACTGACACTGAGGTGCGAAAGCGTGGGGAGCAAACAGGATTAGATACCCTGGTAGTCCACGCCGTAAACGATGTCAACTAGCCGTTGGGGTCCTTGAGACTTTAGTGGCGCAGCTAACGCATTAAGTTGACCGCCTGGGGAGTACGGCCGCAAGGTTAAAACTCAAATGAATTGACGGGGGCCCGCACAAGCGGTGGAGCATGTGGTTTAATTCGAAGCAACGCGAAGAACCTTACCAGGCCTTGACATGCAGAGAACTTTCCAGAGATGGATTGGTGCCTTCGGGAACTCTGACACAGGTGCTGCATGGCTGTCGTCAGCTCGTGTCGTGAGATGTTGGGTTAAGTCCCGTAACGAGCGCAACCCTTGTCCTTAGTTACCAGCACGTTATGGTGGGCACTCTAAGGAGACTGCCGGTGACAAACCGGAGGAAGGTGGGGATGACGTCAAGTCATCATGGCCCTTACGGCCTGGGCTACACACGTGCTACAATGGTCGGTACAGAGGGTTGCCAAGCCGCGAGGTGGAGCTAATCCCACAAAACCGATCGTAGTCCGGATCGCAGTCTGCAACTCGACTGCGTGAAGTCGGAATCGCTAGTAATCGCGAATCAGAATGTCGCGGTGAATACGTTCCCGGGCCTTGTACACACCGCCCGTCACACCATGGGAGTGGGTTGCACCAGAAGTAGCTAGTCTAACCTTCGGGAGGAC

>FW507-12TSA

AGTCGAGCGGANGAAGTGAGCTTGCTCACGGATTTAGCGGCGGACGGGTGAGTAATGCCTAGGAATCTGCCTGGTAGTGGGGGATAACGTTTCGAAAGGGACGCTAATACCGCATACGTCCTACGGGAGAAAGCAGGGGACCTTCGGGCCTTGCGCTATCAGATGAGCCTAGGTCGGATTAGCTAGTTGGTGAGGTAATGGCTCACCAAGGCGACGATCCGTAACTGGTCTGAGAGGATGATCAGTCACACTGGAACTGAGACACGGTCCAGACTCCTACGGGAGGCAGCAGTGGGGAATATTGGACAATGGGCGAAAGCCTGATCCAGCCATGCCGCGTGTGTGAAGAAGGTCTTCGGATTGTAAAGCACTTTAAGTTGGGAGGAAGGGCAGTAAGCTAATATCTTGCTGTTTTGACGTTACCGACAGAATAAGCACCGGCTAACTCTGTGCCAGCAGCCGCGGTAATACAGAGGGTGCAAGCGTTAATCGGAATTACTGGGCGTAAAGCGCGCGTAGGTGGTTTGTTAAGTTGGATGTGAAAGCCCCGGGCTCAACCTGGGAACTGCATCCAAAACTGGCAAGCTAGAGTATGGTAGAGGGTGGTGGAATTTCCTGTGTAGCGGTGAAATGCGTAGATATAGGAAGGAACACCAGTGGCGAAGGCGACCACCTGGACTGATACTGACACTGAGGTGCGAAAGCGTGGGGAGCAAACAGGATTAGATACCCTGGTAGTCCACGCCGTAAACGATGTCAACTAGCCGTTGGGGTCCTTGAGACTTTAGTGGCGCAGCTAACGCATTAAGTTGACCGCCTGGGGAGTACGGCCGCAAGGTTAAAACTCAAATGAATTGACGGGGGCCCGCACAAGCGGTGGAGCATGTGGTTTAATTCGAAGCAACGCGAAGAACCTTACCAGGCCTTGACATGCAGAGAACTTTCCAGAGATGGATTGGTGCCTTCGGGAACTCTGACACAGGTGCTGCATGGCTGTCGTCAGCTCGTGTCGTGAGATGTTGGGTTAAGTCCCGTAACGAGCGCAACCCTTGTCCTTAGTTACCAGCACGTTATGGTGGGCACTCTAAGGAGACTGCCGGTGACAAACCGGAGGAAGGTGGGGATGACGTCAAGTCATCATGGCCCTTACGGCCTGGGCTACACACGTGCTACAATGGTCGGTACAGAGGGTTGCCAAGCCGCGAGGTGGAGCTAATCCCACAAAACCGATCGTAGTCCGGATCGCAGTCTGCAACTCGACTGCGTGAAGTCGGAATCGCTAGTAATCGCGAATCAGAATGTCGCGGTGAATACGTTCCCGGGCCTTGTACACACCGCCCGTCACACCATGGGAGTGGGTTGCACCAGAAGTAGCTAGTCTAACCTTCGGGAGGACGGT

>FW507-11TSA

AGTCGAACGGCAGCACGGGAGCAATCCTGGTGGCGAGTGGCGAACGGGTGAGTAATACATCGGAACGTGCCCTGTCGTGGGGGATAACTAGTCGAAAGATTAGCTAATACCGCATACGACCTGAGGGTGAAAGCGGGGGACCGTAAGGCCTCGCGCGATAGGAGCGGCCGATGTCTGATTAGCTAGTTGGTGGGGTAAAGGCCCACCAAGGCGACGATCAGTAGCTGGTCTGAGAGGACGATCAGCCACACTGGGACTGAGACACGGCCCAGACTCCTACGGGAGGCAGCAGTGGGGAATTTTGGACAATGGGGGCAACCCTGATCCAGCAATGCCGCGTGTGTGAAGAAGGCCTTCGGGTTGTAAAGCACTTTTGTCCGGAAAGAAATCCCTTGCTCTAATACAGCGGGGGGATGACGGTACCGGAAGAATAAGCACCGGCTAACTACGTGCCAGCAGCCGCGGTAATACGTAGGGTGCGAGCGTTAATCGGAATTACTGGGCGTAAAGCGTGCGCAGGCGGTTTTGTAAGACAGGCGTGAAATCCCCGAGCTCAACTTGGGAATGGCGCTTGTGACTGCAAGGCTAGAGTATGTCAGAGGGGGGTAGAATTCCACGTGTAGCAGTGAAATGCGTAGAGATGTGGAGGAATACCGATGGCGAAGGCAGCCCCCTGGGACGTCACTGACGCTCATGCACGAAAGCGTGGGGAGCAAACAGGATTAGATACCCTGGTAGTCCACGCCCTAAACGATGTCAACTAGTTGTTGGGGATTCATTTCTTCAGTAACGTAGCTAACGCGTGAAGTTGACCGCCTGGGGAGTACGGTCGCAAGATTAAAACTCAAAGGAATTGACGGGGACCCGCACAAGCGGTGGATGATGTGGATTAATTCGATGCAACGCGAAAAACCTTACCTACCCTTGACATGCCACTAACGAAGCAGAGATGCATTAGGTGCCNGAAAGGGAAAGTGGACACAGGTGCTGCATGGCTGTCGTCAGCTCGTGTCGTGAGATGTTGGGTTAAGTCCCGCAACGAGCGCAACCCTTGTCTTTAGTTGCTACGCAAGAGCACTCTAGAGAGACTGCCGGTGACAAACCGGAGGAAGGTGGGGATGACGTCAAGTCCTCATGGCCCTTATGGGTAGGGCTTCACACGTCATACAATGGTGCGTACAGAGGGTTGCCAACCCGCGAGGGGGAGCTAATCCCAGAAAACGCATCGTAGTCCGGATCGTAGTCTGCAACTCGACTACGTGAAGCTGGAATCGCTAGTAATCGCGGATCAGCATGCCGCGGTGAATACGTTCCCGGGTCTTGTACACACCGCCCGTCACACCATGGGAGTGGGTTTTGCCAGAAGTAGTTAGCCTAACCGCAAGGGGGGCGAT

>GW247-6R2A

GTCGAGCGAACAGAGAAGGAGCTTGCTCCTTCGACGTTAGCGGCGGACGGGTGAGTAACACGTGGGCAACCTACCTTATAGTTTGGGATAACTCCGGGAAACCGGGGCTAATACCGAATAATCTGTTTCACCTCATGGTGAAACACTGAAAGACGGCATCTCGCTGTCGCTATAGGATGGGCCCGCGGCGCATTAGCTAGTTGGTGAGGTAACGGCTCACCAAGGCGACGATGCGTAGCCGACCTGAGAGGGTGATCGGCCACACTGGGACTGAGACACGGCCCAGACTCCTACGGGAGGCAGCAGTAGGGAATCTTCCACAATGGGCGAAAGCCTGATGGAGCAACGCCGCGTGAGTGAAGAAGGATTTCGGTTCGTAAAACTCTGTTGTAAGGGAAGAACAAGTACAGTAGTAACTGGCTGTACCTTGACGGTACCTTATTAGAAAGCCACGGCTAACTACGTGCCAGCAGCCGCGGTAATACGTAGGTGGCAAGCGTTGTCCGGAATTATTGGGCGTAAAGCGCGCGCAGGTGGTTTCTTAAGTCTGATGTGAAAGCCCACGGCTCAACCGTGGAGGGTCATTGGAAACTGGGAGACTTGAGTGCAGAAGAGGATAGTGGAATTCCAAGTGTAGCGGTGAAATGCGTAGAGATTTGGAGGAACACCAGTGGCGAAGGCGACTATCTGGTCTGTAACTGACACTGAGGCGCGAAAGCGTGGGGAGCAAACAGGATTAGATACCCTGGTAGTCCACGCCGTAAACGATGAGTGCTAAGTGTTAGGGGGTTTCCGCCCCTTAGTGCTGCAGCTAACGCATTAAGCACTCCGCCTGGGGAGTACGGTCGCAAGACTGAAACTCAAAGGAATTGACGGGGGCCCGCACAAGCGGTGGAGCATGTGGTTTAATTCGAAGCAACGCGAAGAACCTTACCAGGTCTTGACATCCCGTTGACCACTGTAGAGATATAGTTTCCCCTTCGGGGGCAACGGTGACAGGTGGTGCATGGTTGTCGTCAGCTCGTGTCGTGAGATGTTGGGTTAAGTCCCGCAACGAGCGCAACCCTTGATCTTAGTTGCCATCATTTAGTTGGGCACTCTAAGGTGACTGCCGGTGACAAACCGGAGGAAGGTGGGGATGACGTCAAATCATCATGCCCCTTATGACCTGGGCTACACACGTGCTACAATGGACGATACAAACGGTTGCCAACTCGCGAGAGGGAGCTAATCCGATAAAGTCGTTCTCAGTTCGGATTGTAGGCTGCAACTCGCCTACATGAAGCCGGAATCGCTAGTAATCGCGGATCAGCATGCCGCGGTGAATACGTTCCCGGGCCTTGTACACACCGCCCGTCACACCACGAGAGTTTGTAACACCCGAAGTCGGTGAGGTANCCTTTGGAGCCAGCC

>GW247-4R2A

ACGGTAACAGCACTTCGGTGGCTGACGAGTGGCGAACGGGTGAGTAATACATCGGAACGTGCCCGAGAGTGGGGGATAACGCAGCGAAAGCTGTGCTAATACCGCATACGATCTACGGATGAAAGCAGGGGACCGCAAGGCCTTGCGCTCATGGAGCGGCCGATGGCAGATTAGGTAGTTGGTGGGATAAAAGCTTACCAAGCCGACGATCTGTAGCTGGTCTGAGAGGACGACCAGCCACACTGGGACTGAGACACGGCCCAGACTCCTACGGGAGGCAGCAGTGGGGAATTTTGGACAATGGGCGAAAGCCTGATCCAGCCATGCCGCGTGCAGGATGAAGGCCTTCGGGTTGTAAACTGCTTTTGTACGGAACGAAAAGGTCTTTTCTAATAAAGAAGGCTCATGACGGTACCGTAAGAATAAGCACCGGCTAACTACGTGCCAGCAGCCGCGGTAATACGTAGGGTGCAAGCGTTAATCGGAATTACTGGGCGTAAAGCGTGCGCAGGCGGTTATATAAGACAGATGTGAAATCCCCGGGCTCAACCTGGGAACTGCATTAGTGACTGTATAGCTAGAGTGCGGCAGAGGGGGATGGAATTCCGCGTGTAGCAGTGAAATGCGTAGATATGCGGAGGAACACCGATGGCGAAGGCAATCCCCTGGGCCTGCACTGACGCTCATGCACGAAAGCGTGGGGAGCAAACAGGATTAGATACCCTGGTAGTCCACGCCCTAAACGATGTCAACTGGTTGTTGGGAATTTACTTTCTCAGTAACGAAGCTAACGCGTGAAGTTGACCGCCTGGGGAGTACGGCCGCAAGGTTAAAACTCAAAGGAATTGACGGGGACCCGCACAAGCGGTGGATGATGTGGTTTAATTCGATGCAACGCGAAAAACCTTACCCACCTTTGACATGTATGGAAGATCGCAGAGACGCGATTGTGCTCGAAAGAGAGCCATAACACAGGTGCTGCATGGCTGTCGTCAGCTCGTGTCGTGAGATGTTGGGTTAAGTCCCGCAACGAGCGCAACCCTTGTCATTAGTTGCTACATTTAGTTGGGCACTCTAATGAGACTGCCGGTGACAAACCGGAGGAAGGTGGGGATGACGTCAAGTCCTCATGGCCCTTATAGGTGGGGCTACACACGTCATACAATGGCTGGTACAAAGGGTTGCCAACCCGCGAGGGGGAGCCAATCCCATAAAGCCAGTCGTAGTCCGGATCGCAGTCTGCAACTCGACTGCGTGAAGTCGGAATCGCTAGTAATCGTGGATCAGAATGTCACGGTGAATACGTTCCCGGGTCTTGTACACACCGCCCGTCACACCATGGGAGCGGGTTCTGCCAGAAGTAGGTAGCCTAACCGCAAGGAGGGCGCT

>GW247-3R2A

AAGTCGANCGAACTCTTCGGAGTTAGTGGCGCACGGGTGCGTAACGCGTGGGAATCTGCCCTTGGGTACGGAATAACTCAGAGAAATTTGTGCTAATACCGTATAATGACTTCGGTCCAAAGATTTATCGCCCAAGGATGAGCCCGCGTAAGATTAGCTAGTTGGTGGGGTAAAAGCCTACCAAGGCGACGATCTTTAGCTGGTCTGAGAGGATGATCAGCCACACTGGGACTGAGACACGGCCCAGACTCCTACGGGAGGCAGCAGTGGGGAATATTGGACAATGGGCGAAAGCCTGATCCAGCAATGCCGCGTGAGTGATGAAGGCCCTAGGGTTGTAAAGCTCTTTTACCCGGGATGATAATGACAGTACCGGGAGAATAAGCTCCGGCTAACTCCGTGCCAGCAGCCGCGGTAATACGGAGGGAGCTAGCGTTGTTCGGAATTACTGGGCGTAAAGCGCGCGTAGGCGGTTTTTTAAGTCAGGGGTGAAAGCCCGGAGCTCAACTCCGGAATAGCCTTTGAAACTGGAAGACTTGAATCTTGGAGAGGTCAGTGGAATTCCGAGTGTAGAGGTGAAATTCGTAGATATTCGGAAGAACACCAGTGGCGAAGGCGACTGACTGGACAAGTATTGACGCTGAGGTGCGAAAGCGTGGGGAGCAAACAGGATTAGATACCCTGGTAGTCCACGCCGTAAACGATGATAACTAGCTGTCCGGACTCATAGAGTTTGGGTGGCGCAGCTAACGCATTAAGTTATCCGCCTGGGGAGTACGGTCGCAAGATTAAAACTCAAAGGAATTGACGGGGGCCTGCACAAGCGGTGGAGCATGTGGTTTAATTCGAAGCAACGCGCAGAACCTTACCAGCGTTTGACATCCTGATCGCGGATTAGAGAGATCTTTTCCTTCAGTTCGGCTGGATCAGTGACAGGTGCTGCATGGCTGTCGTCAGCTCGTGTCGTGAGATGTTGGGTTAAGTCCCGCAACGAGCGCAACCCTCATCCCTAGTTGCCATCATTCAGTTGGGCACTCTAAGGAAACTGCCGGTGATAAGCCGGAGGAAGGTGGGGATGACGTCAAGTCCTCATGGCCCTTACGCGCTGGGCTACACACGTGCTACAATGGCGGTGACAGTGGGCAGCAACCCCGCGAGGGGTAGCTAATCTCCAAAAGCCGTCTCAGTTCGGATTGTTCTCTGCAACTCGAGAGCATGAAGGCGGAATCGCTAGTAATCGCGGATCAGCATGCCGCGGTGAATACGTTCCCAGGCCTTGTACACACCGCCCGTCACACCATGGGAGTTGGTTTCACCCGAAGGCAGTGCTCTAACCCGCAAGGGA

>GW247-2R2A

TGCAGTCGAACGAACTCTTCGGAGTTAGTGGCGCACGGGTGCGTAACGCGTGGGAATCTGCCCTTGGGTACGGAATAACTCAGAGAAATTTGTGCTAATACCGTATAATGACTTCGGTCCAAAGATTTATCGCCCAAGGATGAGCCCGCGTAAGATTAGCTAGTTGGTGGGGTAAAAGCCTACCAAGGCGACGATCTTTAGCTGGTCTGAGAGGATGATCAGCCACACTGGGACTGAGACACGGCCCAGACTCCTACGGGAGGCAGCAGTGGGGAATATTGGACAATGGGCGAAAGCCTGATCCAGCAATGCCGCGTGAGTGATGAAGGCCCTAGGGTTGTAAAGCTCTTTTACCCGGGATGATAATGACAGTACCGGGAGAATAAGCTCCGGCTAACTCCGTGCCAGCAGCCGCGGTAATACGGAGGGAGCTAGCGTTGTTCGGAATTACTGGGCGTAAAGCGCGCGTAGGCGGTTTTTTAAGTCAGGGGTGAAAGCCCGGAGCTCAACTCCGGAATAGCCTTTGAAACTGGAAGACTTGAATCTTGGAGAGGTCAGTGGAATTCCGAGTGTAGAGGTGAAATTCGTAGATATTCGGAAGAACACCAGTGGCGAAGGCGACTGACTGGACAAGTATTGACGCTGAGGTGCGAAAGCGTGGGGAGCAAACAGGATTAGATACCCTGGTAGTCCACGCCGTAAACGATGATAACTAGCTGTCCGGACTCATAGAGTTTGGGTGGCGCAGCTAACGCATTAAGTTATCCGCCTGGGGAGTACGGTCGCAAGATTAAAACTCAAAGGAATTGACGGGGGCCTGCACAAGCGGTGGAGCATGTGGTTTAATTCGAAGCAACGCGCAGAACCTTACCAGCGTTTGACATCCTGATCGCGGATTAGAGAGATCTTTTCCTTCAGTTCGGCTGGATCAGTGACAGGTGCTGCATGGCTGTCGTCAGCTCGTGTCGTGAGATGTTGGGTTAAGTCCCGCAACGAGCGCAACCCTCATCCCTAGTTGCCATCATTCAGTTGGGCACTCTAAGGAAACTGCCGGTGATAAGCCGGAGGAAGGTGGGGATGACGTCAAGTCCTCATGGCCCTTACGCGCTGGGCTACACACGTGCTACAATGGCGGTGACAGTGGGCAGCAACCCCGCGAGGGGTAGCTAATCTCCAAAAGCCGTCTCAGTTCGGATTGTTCTCTGCAACTCGAGAGCATGAAGGCGGAATCGCTAGTAATCGCGGATCAGCATGCCGCGGTGAATACGTTCCCAGGCCTTGTACACACCGCCCGTCACACCATGGGAGTTGGTTTCACCCGAAGGCAGTGCTCTAACCCGCAAGGGAGGAAGC

>GW247-1R2A

TGCAGTCGAACGAACTCTTCGGAGTTAGTGGCGCACGGGTGCGTAACGCGTGGGAATCTGCCCTTGGGTACGGAATAACTCAGAGAAATTTGTGCTAATACCGTATAATGACTTCGGTCCAAAGATTTATCGCCCAAGGATGAGCCCGCGTAAGATTAGCTAGTTGGTGGGGTAAAAGCCTACCAAGGCGACGATCTTTAGCTGGTCTGAGAGGATGATCAGCCACACTGGGACTGAGACACGGCCCAGACTCCTACGGGAGGCAGCAGTGGGGAATATTGGACAATGGGCGAAAGCCTGATCCAGCAATGCCGCGTGAGTGATGAAGGCCCTAGGGTTGTAAAGCTCTTTTACCCGGGATGATAATGACAGTACCGGGAGAATAAGCTCCGGCTAACTCCGTGCCAGCAGCCGCGGTAATACGGAGGGAGCTAGCGTTGTTCGGAATTACTGGGCGTAAAGCGCGCGTAGGCGGTTTTTTAAGTCAGGGGTGAAAGCCCGGAGCTCAACTCCGGAATAGCCTTTGAAACTGGAAGACTTGAATCTTGGAGAGGTCAGTGGAATTCCGAGTGTAGAGGTGAAATTCGTAGATATTCGGAAGAACACCAGTGGCGAAGGCGACTGACTGGACAAGTATTGACGCTGAGGTGCGAAAGCGTGGGGAGCAAACAGGATTAGATACCCTGGTAGTCCACGCCGTAAACGATGATAACTAGCTGTCCGGACTCATAGAGTTTGGGTGGCGCAGCTAACGCATTAAGTTATCCGCCTGGGGAGTACGGTCGCAAGATTAAAACTCAAAGGAATTGACGGGGGCCTGCACAAGCGGTGGAGCATGTGGTTTAATTCGAAGCAACGCGCAGAACCTTACCAGCGTTTGACATCCTGATCGCGGATTAGAGAGATCTTTTCCTTCAGTTCGGCTGGATCAGTGACAGGTGCTGCATGGCTGTCGTCAGCTCGTGTCGTGAGATGTTGGGTTAAGTCCCGCAACGAGCGCAACCCTCATCCCTAGTTGCCATCATTCAGTTGGGCACTCTAAGGAAACTGCCGGTGATAAGCCGGAGGAAGGTGGGGATGACGTCAAGTCCTCATGGCCCTTACGCGCTGGGCTACACACGTGCTACAATGGCGGTGACAGTGGGCAGCAACCCCGCGAGGGGTAGCTAATCTCCAAAAGCCGTCTCAGTTCGGATTGTTCTCTGCAACTCGAGAGCATGAAGGCGGAATCGCTAGTAATCGCGGATCAGCATGCCGCGGTGAATACGTTCCCAGGCCTTGTACACACCGCCCGTCACACCATGGGAGTTGGTTTCACCCGAAGGCAGTGCTCTAACCCGCAAGGGAG

>FW300-N2E4

TGCAGTCGAACGGCAGCGCGGGGCAACCTGGCGGCGAGTGGCGAACGGGTGAGTAATATATCGGAACGTACCCTGGAGTGGGGGATAACGTAGCGAAAGTTACGCTAATACCGCATACGATCTAAGGATGAAAGCAGGGGACCGCAAGGCCTTGTGCTCCTGGAGCGGCCGATATCTGATTAGCTAGTTGGTGGGGTAAANGCCNACCAAGGCATCGATCAGTAGCTGGTCTGAGAGGACGACCAGCCACACTGGAACTGAGACACGGTCCAGACTCCTACGGGAGGCAGCAGTGGGGAATTTTGGACAATGGGCGCAAGCCTGATCCAGCAATGCCGCGTGAGTGAAGAAGGCCTTCGGGTTGTAAAGCTCTTTTGTCAGGGAAGAAACGGCCTGTTCTAATACAGTGGGCTAATGACGGTACCTGAAGAATAAGCACCGGCTAACTACGTGCCAGCAGCCGCGGTAATACGTAGGGTGCAAGCGTTAATCGGAATTACTGGGCGTAAAGCGTGCGCAGGCGGTTTTGTAAGACTGTCGTGAAATCCCCGGGCTCAACCTGGGAATGGCGATGGTGACTGCAAGGCTAGAGTTTGGCAGAGGGGGGTAGAATTCCACGTGTAGCAGTGAAATGCGTAGATATGTGGAGGAACACCGATGGCGAAGGCAGCCCCCTGGGTCAAAACTGACGCTCATGCACGAAAGCGTGGGGAGCAAACAGGATTAGATACCCTGGTAGTCCACGCCCTAAACGATGTCTACTAGTTGTCGGGTCTTAATTGACTTGGTAACGCAGCTAACGCGTGAAGTAGACCGCCTGGGGAGTACGGTCGCAAGANTAAAACTCAAAGGAATTGACGGGGACCCGCACAAGCGGTGGATGATGTGGATTAATTCGATGCAACGCGAAAAACCTTACCTACCCTTGACATGGNTGGAATC

>FW300-N2C3

GCGGCAGCACGGGTACTTGTACCTGGTGGCGNNGCGGCGGACGGGTGAGNAATGCCTAGGAATCTGCCTGGTAGTGGGGGATAACGTTCGGAAACGAACGCTAATACCGCATACGTCCTACGGGAGAAAGCAGGGGACCTTCGGGCCTTGCGCTATCAGATGAGCCTAGGTCGGATTAGCTAGTTGGTGAGGTAATGGCTCACCAAGGCGACGATCCGTAACTGGTCTGAGAGGATGATCAGTCACACTGGAACTGAGACACGGTCCAGACTCCTACGGGAGGCAGCAGTGGGGAATATTGGACAATGGGCGAAAGCCTGATCCAGCCATGCCGCGTGTGTGAAGAAGGTCTTCGGATTGTAAAGCACTTTAAGTTGGGAGGAAGGGCAGTTACCTAATACGTAATTGTTTTGACGTTACCGACAGAATAAGCACCGGCTAACTCTGTGCCAGCAGCCGCGGTAATACAGAGGGTGCAAGCGTTAATCGGAATTACTGGGCGTAAAGCGCGCGTAGGTGGTTCGTTAAGTTGGATGTGAAATCCCCGGGCTCAACCTGGGAACTGCATTCAAAACTGTCGAGCTAGAGTATGGTAGAGGGTNGGTGGAATTTCCTGTGTAGCGGTGAAATGCGTAGATATAGGAAGGAACACCAGTGGCGAAGGCGACCACCTGGACTGATACTGACACTGAGGTGCGAAAGCGTGGGGAGCAAACAGGATTAGATACCCTGGTAGTCCACGCCGTAANCGATGTCAACTAGCCGTTGGGAGCCTTGAGCTCTTAGTGGCGCAGCTAACGCATTAAGTTGACCGCCTGGGGAGTACGGCCGCAAGGTTAAAACTCAAATGAATTGACGNGGGCCCGCACAAGCGGTGGAGCATGTGGTTTAATTCGAAGCAACGCGAAGAACCTTACCAGGCCTTGACATCCAATGAACTTTCCAGAGATGGATTGGTGCCTTCGGGAACATTGAGACAGGTGCTGCATGGCTGTCGTCAGCTCGTGTCGTGAGATGTTGGGTTAAGTCCCGTAACGAGCGCAACCCTTGTCCTTAGTTACCAGCACGTAATGGTGGGCACTCTAAGGAGACTGCCGGTGACAAACCGGAGGAAGGTGGGGATGACGTCAAGTCATCATGGCCCTTACGGCCTGGGCTACACACGTGCTACAATGGTCGGTACAGAGGGTTGCCAAGCCGCGAGGTGGAGCTAATCCCATAAAACCGATCGTAGTCCGGATCGCAGTCTGCAACTCGACTGCGTGAAGTCGGAATCGCTAGTAATCGCGAATCAGAATGTCGCGGTGAATACGTTCCCGGGCCTTGTACACACCGCCCGTCACACCATGGGAGTGGGTTGCACCAGAAGTAGCTAGTCTAACCTTCGGGG

>FW300-N2C1

TGCAGTCGAGCGGTAGAGAGAANCTTGCTTCTCTTGANAGCGGCGGACGGGTGAGTAATGNCNNNGAATCTGCCTGGNANTGGGGGATAACGCTCGGAAACGGACGCTAATACCGCATACGTCCTACGGGAGAAAGCAGGGGACCTTCNGGCCTTGCGCTATCNNATGAGCCTANGTCGGATTANCTAGTTGGTGAGGTAATGGCTCACCAAGGCGACNATCCNTAACTGGTCTGAGAGGATGATCANTCACACTGGAACTGAGACACGGTCCANACTCCTACNGGAGGCANCNGTGGGGAATATTGGACAATGGGCGAAAGCCTGATCCANCCNTGCCNCGTGTGTGAANAANGTCTTCNGATTGTAAAGCACTTTANNTTGGGAGGAAGGGNNNTAAATTAATACTTTGCTGTTTTGACGTTACCGACAGAATAANCACCGGCTAACTCTGTGCCANCANCCGCGGTAATACANAGGGTGCAAGCGTTAATCGGAATTACTGGGCGTAAAGCGCGCGTANGTGGTTTGTTAAGTTGGATGTGAAATCCCCGGGCTCAACCTGGGAACTGCATTCAAAACTGACNAGCTANAGTATGGTAGAGGGNGGTGGAATTTNNNGTGTANCGGTGAAATGCGTANATATANGAAGGAACACCANTGGCGAANGCGACCACCTGGACTGATACTGACACTGAGGTGCGAAAGCGTGNGGAGCANACANGATTANATACCCTGNTANTCCACGCCNTANACNATGTCNACTANCCNTTGGGAGCCTTGAGCTCTTANTGNNGCANCTAACNCATTAANTTGANNNCCTGGGGAGTACNGNCGCNAGGNNANNACTCANATGAATTGACNGGGGCCCNCACAANCGGTGGAGNNTGTGGNTTTNNNTCTAAGCANCNCG

>FW104-15D2A

TACGACGGCTRGCTCCTTACGGTTACTCCACCGRCTTCGGGTGTTACAAACTCTCGTGGTGTGACGGGCGGTGTGTACAAGRCCCGGGAACGTATTCACCGYRGCATGCTGATCYRCGATTACTAGCGATTCCRGCTTCATGYAGKCGAGTTGCAGMCTRCAATCCGAACTGAGAAYRRYTTTATGGGATTKGCTTSACCTCGCGGYTTCGCTRCCCTTTGTAYYRTCCATTGTAGCACGTGTGTAGCCCARRTCATAAGGGGCATGATGATTTGACGTCATCCCCACCTTCCTCCGGTTTGTCACCGGCAGTCAMCTTAGAGTGCCCAACTKAATGMTGGCAACTAAGMTYAAGGGTTGCGCTCGTTGCGGGACTTAACCCAACATCTCACGACACGAGCTGACGACAACCATGCACCACCTGTCACTCTGTCCCCCGAAGGGGAAMRCYCTATCTCTAGRGKTGTCAGAGGATGTCAAGAYYTGGTAAGGTTCTTCGCGTTGCTTCGAATTAAACCACATGCTCCACCGCTTGTGCGGGYCCCCGTCAATTCCTTTGAGTTTCARCCTTGCGGYCGTACTCCCCAGGCGGAGTGCTTAATGCGTTAGCTGCAGCACTAARGGGCGGAAACCCYCTAACACTTAGCACTCATCGTTTACGGCGTGGACTACCAGGGTATCTAATCCTGTTTGMTCCCCACGCTTTCGCRCMTCAGCGTCAGTTACAGACCAGAAAGTCGCCTTCGCCACTGGTGTTCCTCCAYATCTCTRCGCATTTCACCGCTACACRTGGAATTCCACTTTCCTCTTCTGCACTCAAGTYYYCCAGTTTCCAATGACCCTCCACGGTTGAGCCGTGGGCTTTCACATCAGACTTAAARRACCGCCTRCGCGCGCTTTACGCCCAATAATTCCGGACAACGCTTGCCACCTACGTATTACCGCGGCTGCTGGCACGTAGTTAGCCGTGGCTTTCTGRTYAGGTACCGTCAAGGTACCGGCATAGTTACTYMGGYACTTGTTCTTCCCTRAYAACAGAGYTTTACGATCCGAAGGCCTTCATCRCTCACGCGGCGTTGCTCCGTCAGRCTTTCGYCCATTGCGGAANATTCCCTACTGCTGCCTCCCGTAGGNNTCTGGACCG

>FW104-15D2B

ATGCAGTCGAGCNCACCTTTCGGGGTGAGCGGCGGACGGGTGAGTAACGCGTGGGAATATGCCCTATGGTGCGGAACAACTGAGGGAAACTTCAGCTAATACCGCATGTGCCCTACGGGGGAAAGATTTATCGCCATAGGAGTAGCCCGCGTTGGATTAGTTTGTTGGTGGGGTAATGGCTCACCAAGACTACGATCCATAGCTGGTCTGAGAGGATGATCAGCCACACTGGGACTGAGACACGGCCCAGACTCCTACGGGAGGCAGCAGTAGGGAATCTTGCGCAATGGGCGAAAGCCTGACGCAGCCATGCCGCGTGAATGATGAAGGTCTTAGGATTGTAAAATTCTTTCACCGGGGACGATAATGACGGTACCCGGAGAAGAAGCTCCGGCTAACTTCGTGCCAGCAGCCGCGGTAATACGAAGGGGGCTAGCGTTGCTCGGAATTACTGGGCGTAAAGGGCGCGTAGGCGGACAGTTTAGTCAGAGGTGAAAGCCCAGGGCTCAACCTTGGAATAGCCTTTGATACTGACTGTCTTGAGTACGGGAGAGGTGTGTGGAACTCCGAGTGTAGAGGTGAAATTCGTAGATATTCGGAAGAACACCAGTGGCGAAGGCGACACACTGGCCCGTTACTGACGCTGAGGCGCGAAAGCGTGGGGAGCAAACAGGATTAGATACCCTGGTAGTCCACGCTGTAAACGATGAGTGCTAGTTGTCGGCATGCATGCATGTCGGTGACGCAGCTAACGCATTAAGCACTCCGCCTGGGGAGTACGGTCGCAAGATTAAAACTCAAAGGAATTGACGGGGGCCCGCACAAGCGGTGGAGCATGTGGTTTAATTCGAAGCAACGCGCAGAACCTTACCACCTTTTGACATGCCCGGCTCGCCAGAGAGATCTGGTTTTCCCTTCGGGGACCGGGACACAGGTGCTGCATGGCTGTCGTCAGCTCGTGTCGTGAGATGTTGGGTTAAGTCCCGCAACGAGCGCAACCCTCGCCATTAGTTGCCATCATTAAGTTGGGCACTCTAGTGGGACCGCCGGTGGTAAGCCGGAGGAAGGTGGGGATGACGTCAAGTCCTCATGGCCCTTACGGGGTGGGCTACACACGTGCTACAATGGCGACTACAGAGGGCAGCAAGCCAGCGATGGGGAGCTAATCCCTAAAAGTCGTCTCAGTTCGGATTGCACTCTGCAACTCGAGTGCATGAAGTCGGAATCGCTAGTAATCGCGGATCAGCATGCCGCGGTGAATACGTTCCCGGGCCTTGTACACACCGCCCGTCACACCATGGGAGTTGGCTTTACCCGAAGGCGGTGCGCTAACCAGCAATGGAGGCA

>FW104-15G4A

TGCAGTCGAACGATGAAGCCTAGCTTGCTGGGTGGATTAGTGGCGAACGGGTGAGTAACACGTGAGCAACCTGCCCCTGACTCTGGGATAAGCGCTGGAAACGGCGTCTAATACTGGATATGAGCCTTCCACGCATGTGGGGGGTTGGAAAGATTTTTTGGTTGGGGATGGGCTCGCGGCCTATCAGCTTGTTGGTGAGGTAATGGCTCACCAAGGCGTCGACGGGTAGCCGGCCTGAGAGGGTGACCGGCCACACTGGGACTGAGACACGGCCCAGACTCCTACGGGAGGCAGCAGTGGGGAATATTGCACAATGGGCGGAAGCCTGATGCAGCAACGCCGCGTGAGGGATGACGGCCTTCGGGTTGTAAACCTCTTTTAGCAAGGAAGAAGCGAAAGTGACGGTACTTGCAGAAAAAGCGCCGGCTAACTACGTGCCAGCAGCCGCGGTAATACGTAGGGCGCAAGCGTTATCCGGAATTATTGGGCGTAAAGAGCTCGTAGGCGGTTTGTCGCGTCTGCTGTGAAATCNCCGAGGCTCAACCTCGGGCCTGCAGTGGGTACGGGCAGACTAGAGTGCGGTAGGGGAGATTGGAATTCCTGGTGTAGCGGTGGAATGCGCAGATATCAGGAGGAACACCGATGGCGAAGGCAGATCTCTGGGCCGTAACTGACGCTGAGGAGCGAAAGGGTGGGGAGCAAACAGGCTTAGATACCCTGGTAGTCCACCCCGTAAACGTTGGGAACTAGTTGTGGGGTCCTTTCCACGGATTCCGTGACGCAGCTAACGCATTAAGTTCCCCGCCTGGGGAGTACGGCCGCAAGGCTAAAACTCAAAGGAATTGACGGGGACCCGCACAAGCGGCGGAGCATGCGGATTAATTCGATGCAACGCGAAGAACCTTACCAAGGCTTGACATACACGAGAACACCCTAGAAATAGGGGACTCTTTGGACACTCGTGAACAGGTGGTGCATGGTTGTCGTCAGCTCGTGTCGTGAGATGTTGGGTTAAGTCCCGCAACGAGCGCAACCCTCGTTCTATGTTGCCAGCACGTAATGGTGGGAACTCATNGGGATACTNGCCGGGGTCAACTNCGGAGGAAGGTGGGGATGACGTCAAATCATCATGCCCCTTATGTCTTGGGCTTCACGCATGCTACAATGGCCGGTACAAAGGGCTGCAATACCGTGAGGTGGAGCGAATCCCAAAAAGCCGGTCCCAGTTCGGATTGAGGTCTGCAACTCGACCTCATGAAGTCGGAGTCGCTAGTAATCGCAGATCAGCAACGCTGCGGTGAATACGTTCCCGGGTCTTGTACACACCGCCCGTCAAGTCATGAAAGTCGGTAACACCTGAAGCCGGTGGCCCAACCCTTGTGGANGGANC

>FW104-15G4B

TGCAGTCGANCGGTGATGGCCNAGCTTGCTTGGTCTGATCAGTGGCGAACGGGTGAGTAACACGTGAGTAACCTGCCCCAGACTCTGGGATAACCCCGGGAAACCGGAGCTAATACCGGATATGACACCAATCCGCATGGGTATGGTGTGGAAAGTTTTTCGGTCTGGGATGGGCTCGCGGCCTATCAGCTTGTTGGTGAGGTAGTGGCTCACCAAGGCGACGACGGGTAGCCGGCCTGAGAGGGCGACCGGCCACACTGGGACTGAGACACGGCCCAGACTCCTACGGGAGGCAGCAGTGGGGAATATTGCACAATGGGCGGAAGCCTGATGCAGCGACGCCGCGTGAGGGATGACGGCCTTCGGGTTGTAAACCTCTTTCAGCAGGGAAGAAGCGCAAGTGACGGTACCTGCAGAAGAAGCACCGGCTAACTACGTGCCAGCAGCCGCGGTAATACGTAGGGTGCGAGCGTTGTCCGGAATTATTGGGCGTAAAGAGCTTGTAGGTGGTTTGTCGCGTCTGCTGTGAAAATCCGAGGCTCAACCTCGGACTTGCAGTGGGTACGGGCAGACTAGAGTGTGGTAGGGGAGACTGGAATTCCTGGTGTAGCGGTGGAATGCGCAGATATCAGGAGGAACACCGATGGCGAAGGCAGGTCTCTGGGCCATTACTGACACTGAGAAGCGAAAGCATGGGGAGCGAACAGGATTAGATACCCTGGTAGTCCATGCCGTAANCGTTGGGAACTAGGTGTGGGTCTCATTCCACGAGATCCGTGCCGCAGCTAACGCATTAAGTTCCCCGCCTGGGGAGTACGGCCGCAAGGCTAAAACTCAAAGGAATTGACGGGGGCCCGCACAAGCGGCGGAGCATGCGGATTAATTCGATGCAACGCGAAGAACCTTACCAAGGCTTGACATACACCGGAATCACTCAGAGATGGGTGCGTCTTCGGACTGGTGTACAGGTGGTGCATGGTTGTCGTCAGCTCGTGTCGTGAGATGTTGGGTTAAGTCCCGCAACGAGCGCAACCCTCGTTCTATGTTGCCAGCACGTGATGGTGGGGACTCATAGGAGACTGCCGGGGTCAACTCGGAGGAAGGTGGGGATGACGTCAAATCATCATGNCCCCTTATGTCTTGGGCTTNCACGCATGCTACAATGGCCGGTACAAAGGGCTGCGATACCGCGAGGTGGAGCGAATCCCAAAAAACCGGTCTCAGTTCGGATTGGGGTCTGCAACTCGACCCCATGAAGTCGGAGTCGCTAGTAATCGCAGATCAGCAACGCTGCGGTGAATACGTTCCCGGGCCTTGTACACACCGCCCGTCAAGTCACGAAAGTCGGTAACACCCGAAGCCGGTGGCCCAACTGTTACAGA

>FW104-15G4D

TTCGACGGCTAGCTCCNAAAGGNTACTCCACCGGCTTCGGGTGTTACAAACTCTCGTGGTGTGACGGGCGGTGTGTACAAGACCCGGGAACGTATTCACCGTAGCATGCTGATCTACGATTACTAGCGATTCCAGCTTCATGTAGTCGAGTTGCAGACTACAATCCGAACTGAGAACAACTTTATGGGATTTGCTTGACCTCGCGGTTTCGCTACCCTTTGTATTGTCCATTGTAGCACGTGTGTAGCCCAAATCATAAGGGGCATGATGATTTGACGTCATNCCCCACCTTCCTCCGGTTTGTCACCGGCAGTCAACTTAGAGTGCCCAACTTAATGATGGCAACTAAGCTTAAGGGTTGCGCTCGTTGCGGGACTTAACCCAACATCTCACGACACGAGCTGACGACAACCATGCACCACCTGTCACTCTGTCCCCCGAAGGGGAAAACTCTATCTCTAGAGGGGTCAGAGGATGTCAAGATTTGGTAAGGTTCTTCGCGTTGCTTCGAATTAAACCACATGCTCCACCGCTTGTGCGGGTCCCCGTCAATTCCTTTGAGTTTCAACCTTGCGGTCGTACTCCCCAGGCGGAGTGCTTAATGCGTTAGCTGCAGCACTAAGGGGCGGAAACCCCCTAACACTTAGCACTCATCGTTTACGGCGTGGACTACCAGGGTATCTAATCCTGTTTGATCCCCACGCTTTCGCACATCAGCGTCAGTTACAGACCAGAAAGTCGCCTTCGCCACTGGTGTTCCTCCATATCTCTGCGCATTTCACCGCTACACATGGAATTCCACTTTCCTCTTCTGCACTCAAGTTTTCCAGTTTCCAATGACCCTCCACGGTTGAGCCGTGGGCTTTCACATCAGACTTAAAAAACCGCCTACGCGCGCTTTACGCCCAATAATTCCGGATAACGCTTGCCACCTACGTATTACCGCGGCTGCTGGCACGTAGTTAGCCGTGGCTTTCTGATTAGGTACCGTCAAGACGTGCATAGTTACTTACACATTTGTTCTTCCCTAATAACAGAGTTTTACGATCCGAAGACCTTCTTCACTCACGCGGCGTTGCTCCGTCAGGCTTTCGCCCATTGCGGAAGATTCCCTACTGCTGCCTCCCGTAGGAGTCTGGACCGTGTCTCAGTTCCAGTGTGGCCGATCACCCTCTCAGGTCGGCTACGCATCGTTGCCTTGGTAAGCCGTTACCTTACCAACTAGCTAATGCGGCGCGGATCCATCTATAAGTGACAGCAAGACCGTCTTTCACTGTTGAACCATGCGGTTCAACATGTTATCCGGTATTAGCTCCGGTTTCCCGAAGTTATCCCAGTCTTATAGGTAGGTTATCCACGTGTTACTCACCCGTCCGCCGCTAACGTCAGAGGAGCAAGCTCCTCGTCTGTTCGCTCGACTGCANGTATAG

>FW104-7B9A

CAGCCAGCAGGTAATCGTGGGAACTCTAAGGAGAGTACAGGTGCCAACCCGGAGGAAGGTGGGGATGACGTCAAGTCATCATGGCCCTTACGCCCAGGGCTACACCCGTCCTACAATGGTCGGTACAGAGGGTTGCAATACCCCGAGGTGGAGCCAATCCCAGAAAGCCGATCCCAGTCCGGATTGGAGTCTGCAACTCGACTCCATGAAGTCGGAATCGCTAGTAATCGCGGATCAGCTATGCCGCGGTGAATACGTTCCCGGGCCTTGTACACACCGCCCGTCACACCATGGGAGTGGGTTGCTCCAGAAGGCGTTAGTCTAACCGCAAGGAGGACGTGCAGTCGANCGGCAGCACAGNTGGGTGGCGAGTGGCGGACGGGTGAGTAATGCATCGGGATCTACCCTGACGTGGGGGATAACCTCGGGAAACCGGGACTAATACCGCATACGTCCTACGGGAGAAAGCGGGGGACCTTCGGGCCTCGCGCGGCAGGACGAACCGATGTGCGATTAGCTAGTTGGCGGGGTAATGGCCCACCAAGGCGACGATCGCTAGCTGGTCTGAGAGGATGATCAGCCACACTGGGACTGAGACACGGCCCAGACTCCTACGGGAGGCAGCAGTGGGGAATATTGGACAATGGGCGCAAGCCTGATCCAGCAATGCCGCGTGTGTGAAGAAGGCCTTCGGGTTGTAAAGCACTTTTATCAGGAGCGAAATACCACGGGTTAATACCCTATGGGGCTGACGGTACCTGAGGAATAAGCACCGGCTAACTTCGTGCCAGCAGCCGCGGTAATACGAAGGGTGCAAGCGTTAATCGGAATTACTGGGCGTAAAGGGTGCGTAGGCGGTTACTTAAGTCTGTCGTGAAATCCCCGGGCTCAACCTGGGAATGGCGATGGATACTGGGTGGCTAGAGTGTGTCAGAGGATGGTGGAATTCCCGGTGTAGCGGTGAAATGCGTAGAGATCGGGAGGAACATCAGTGGCGAAGGCGGCCATCTGGGACAACACTGACGCTGAAGCACGAAAGCGTGGGGAGCAAACAGGATTAGATACCCTGGTAGTCCACGCCCTAANCGATGCGAACTGGATGTTGGTCTCAACTCGGAGATCAGTGTCGAAGCTAACGCGTTAAGTTCGCCGCCTGGGGAGTACGGTCGCAAGACTGAANCTCAANGGAATTGACGGGGGCCCGCACAAGCGGTGGAGTATGTGNNTTAATTCGATGCAACGCNANGAACCTTACCTGGCNTTGACATGTCCGGAATCCTNNANACATGCGGGAGTGCCTTCGGNAATCGGAA

>FW104-7B9B

TGCAGTCGANCGATGAAGCCCAGCTTGCTGGGTGGATTAGTGGCGAACGGGTGAGTAACACGTGAGTAACCTGCCCTTGACTCTGGGATAAGCGCTGGAAACGGCGTCTAATACCGGATACGACCTGCCCCGGCATCGGGTGCGGGTGGAAAGTTTTTCGGTCAAGGATGGACTCGCGGCCTATCAGCTAGTTGGTGAGGTAATGGCTCACCAAGGCGACGACGGGTAGCCGGCCTGAGAGGGTGACCGGCCACACTGGGACTGAGACACGGCCCAGACTCCTACGGGAGGCAGCAGTGGGGAATATTGCACAATGGGCGAAAGCCTGATGCAGCAACGCCGCGTGGGGGATGACGGCCTTCGGGTTGTAAACCTCTTTTAGTAGGGAAGAAGGGCTTCGGCTTGACGGTACCTGCAGAAAAAGCACCGGCTAACTACGTGCCAGCAGCCGCGGTAATACGTAGGGTGCAAGCGTTGTCCGGAATTATTGGGCGTAAAGAGCTCGTAGGCGGTTTGTCGCGTCTGCTGTGAAAACCCGAGGCTCAACCTCGGGCCTGCAGTGGGTACGGGCAGACTAGAGTGCGGTAGGGGAGAATGGAATTCCTGGTGTAGCGGTGGAATGCGCAGATATCAGGAGGAACACCGATGGCGAAGGCAGTTCTCTGGGCCGTTACTGACGCTGAGGAGCGAAAGCGTGGGGAGCGAACAGGATTAGATACCCTGGTAGTCCACGCCGTAAACGTTGGGAACTAGATGTGGGGACCTTTCCACGGTCTCCGTGTCGCAGCTAACGCATTAAGTTCCCCGCCTGGGGAGTACGGCCGCAAGGCTAAAACTCAAAGGAATTGACGGGGGCCCGCACAAGCGGCGGAGCATGCGGATTAATTCGATGCAACGCGAAGAACCTTACCAAGGCTTGACATATACCGGAAACTTCCAGAAATGGTTGCCCCGCAAGGTCGGTATACAGGTGGTGCATGGTTGTCGTCAGCTCGTGTCGTGAGATGTTGGGTTAAGTCCCGCAACGAGCGCAACCCTCGTTCTATGTTGCCAGCACGTCATGGTGGGAACTCATAGGAGACTGCCGGGGTCAACTCNGGAGGAAGGTGGGGATGACGTCAAATCATCATGCCCCTTATGTCTTGGGCTTCACGCATGCTACAATNGGCCGGTACAAAGGGCTGCGATACCGTAAGGTGGAGCGAATCCCAAAAAGCCGGTCTCAGTTCGGATTGAGGTCTGCAACTCGACCTCATGAAGTCGGAGTCGCTAGTAATCGCAGATCAGCAACGCTGCGGTGAATACGTTCCCGGGCCTTGTACACACCGCCCGTCAAGTCATGAAAGTCGGTAACACCCGAAGCCANTNNCCTAACCGCAAGGAGGGAGC

>FW104-7C8
[truncated: 490,584 more chars]
