# Supplementary material for: Probing the active fraction of soil microbiomes using BONCAT-FACS
Source: Nat Commun. 2019 Jun 24;10:2770. doi: 10.1038/s41467-019-10542-0 (PMC6591230; doi:10.1038/s41467-019-10542-0)
Supplement: Supplementary file 4 — Reporting Summary [file 41467_2019_10542_MOESM4_ESM.pdf]

natureresearch

Corresponding author(s): Estelle Couradeau
Trent R. Northen

Last updated by author(s): Apr 15, 2019

Reporting Summary

Nature Research wishes to improve the reproducibility of the work that we publish. This form provides structure for consistency and transparency in reporting. For further information on Nature Research policies, see Authors & Referees and the Editorial Policy Checklist.

Statistics

For all statistical analyses, confirm that the following items are present in the figure legend, table legend, main text, or Methods section.

n/a	Confirmed	
☐	☒	The exact sample size (*n*) for each experimental group/condition, given as a discrete number and unit of measurement
☐	☒	A statement on whether measurements were taken from distinct samples or whether the same sample was measured repeatedly
☐	☒	The statistical test(s) used AND whether they are one- or two-sided<br>*Only common tests should be described solely by name; describe more complex techniques in the Methods section.*
☒	☐	A description of all covariates tested
☒	☐	A description of any assumptions or corrections, such as tests of normality and adjustment for multiple comparisons
☐	☒	A full description of the statistical parameters including central tendency (e.g. means) or other basic estimates (e.g. regression coefficient) AND variation (e.g. standard deviation) or associated estimates of uncertainty (e.g. confidence intervals)
☐	☒	For null hypothesis testing, the test statistic (e.g. F, t, r) with confidence intervals, effect sizes, degrees of freedom and P value noted<br>*Give P values as exact values whenever suitable.*
☒	☐	For Bayesian analysis, information on the choice of priors and Markov chain Monte Carlo settings
☒	☐	For hierarchical and complex designs, identification of the appropriate level for tests and full reporting of outcomes
☐	☒	Estimates of effect sizes (e.g. Cohen's d, Pearson's r), indicating how they were calculated

Our web collection on statistics for biologists contains articles on many of the points above.

Software and code

Policy information about availability of computer code

Data collection	Flow cytometer data were collected on a BD-Influx cytometer with the BD-Influx software (https://www.bdbiosciences.com/us/instruments/research/cell-sorters/bd-influx/m/744777/overview)<br>Sequencing Data were acquired on q MiSeq with the instrument software (https://www.illumina.com/systems/sequencing-platforms/miseq.html)<br>LCMS data were acquired on a Q Exactive Hybrid Quadrupole-Orbitrap with the ThermoFisher software (https://www.thermofisher.com/order/catalog/product/IQLAAEGAAPFALGMAZR)<br>Please see the method section for more details
Data analysis	16S rDNA were analyzed using Qiime2 (https://qiime2.org) and Geneious R9 (https://www.geneious.com)<br>Flow cytometer data were then analyzed in FlowJo (https://www.flowjo.com)<br>LCMS data were analyzed using Mzmine (http://mzmine.github.io) and Metatlas (https://github.com/biorack/metatlas)<br>Please see the Methods section for more details

For manuscripts utilizing custom algorithms or software that are central to the research but not yet described in published literature, software must be made available to editors/reviewers. We strongly encourage code deposition in a community repository (e.g. GitHub). See the Nature Research guidelines for submitting code & software for further information.

Data

Policy information about availability of data

All manuscripts must include a data availability statement. This statement should provide the following information, where applicable:

- Accession codes, unique identifiers, or web links for publicly available datasets
- A list of figures that have associated raw data
- A description of any restrictions on data availability

The datasets generated during the current study are available in public repository or are directly included in the supplementary information file attached. The 16S rRNA gene sequences from the libraries constructed for this study have been deposited to Genebank under the Bioproject ID PRJNA475109. The 16S rRNA genes

from the ENIGMA culture collection are included in the supplementary file. The raw flow cytometer data collected are displayed in Figure S4 and Figure S5. The LCMS data and analysis are publicly available from the Joint Genome Institute Genome Portal: https://genome.jgi.doe.gov/portal/201CAT_FD/201CAT_FD.info.html. The data file is #1207416 and the analysis file is #1207417.

Field-specific reporting

Please select the one below that is the best fit for your research. If you are not sure, read the appropriate sections before making your selection.

☐ Life sciences ☐ Behavioural & social sciences ☒ Ecological, evolutionary & environmental sciences

For a reference copy of the document with all sections, see nature.com/documents/nr-reporting-summary-flat.pdf

Ecological, evolutionary & environmental sciences study design

All studies must disclose on these points even when the disclosure is negative.

Study description	This study presents data collected on two soil cores that were collected at the Oak Ridge National Lab site. Biological triplicate of 1g of soil
Research sample	Soil samples - unfrozen / undisturbed
Sampling strategy	The soil were cored horizontally at the field site and then subsample in the lab using sterile spatula in a biohood. The exterior part of the core (touching the tube) was avoided. See Fig 1A
Data collection	Dr. Estelle Couradeau participated in the collection of all the data but the field section. Flow cytometry data were collected by Danielle Goudeau. 16S library prep were realiszd by Nandita Nath. The samples were sequenced were acquired by the Joint Genome Institute production pipeline. The LCMS data were acquired by the Northen group production staff.
Timing and spatial scale	All the details regarding when the samples were incubated, sorted, sequenced, pooled can be found in this table: https://docs.google.com/spreadsheets/d/1N8N17uPxgsyRvTBRSE-NJlQ7QCHyrTv9uN7JZwEEt9s/edit?usp=sharing
Data exclusions	No data were excluded
Reproducibility	The data analyzed come from biological triplicates. The BONCAT labelling of our samples under the incubation condition was always consistent including in the pilot study (that did not have replications)
Randomization	The samples with lower percentage of BONCAT + cells were always analyzed first at the cell sorter in order to limit possible cross contamination between samples.
Blinding	The 16rDNA received a 6 digit ID number and were analyzed under this ID on the Qiime 2 pipeline, the origin of the samples was only matched at the very end on the output figures.

Did the study involve field work? ☒ Yes ☐ No

Field work, collection and transport

Field conditions	Collection on January 24th 2017 from a silt loam area. Surface temperature was +6 deg C / no precipitation
Location	GPS 35.941133, -84.336504, Oak Ridge, TN, USA<br>Cores sampled at 30 cm & 76 cm depth
Access and import/export	Samples were collected by Dominique Joyner (Hazen lab, ORNL), and placed in a cooler with ice packs and shipped overnight to the Joint Genome Institute under the soil permit P330-15-00108_20150413. It is a regulated area and therefore all the material produced directly or indirectly by the experimentation has been treated as biohazard
Disturbance	The soil was sub-sampled with a spatula trying to respect the soil structure and to not break soil aggregates. The incubation was performed in still condition to try to preserve the soil structure.

Reporting for specific materials, systems and methods

We require information from authors about some types of materials, experimental systems and methods used in many studies. Here, indicate whether each material, system or method listed is relevant to your study. If you are not sure if a list item applies to your research, read the appropriate section before selecting a response.

Materials & experimental systems

n/a	Involved in the study	
☒	☐	Antibodies
☒	☐	Eukaryotic cell lines
☒	☐	Palaeontology
☒	☐	Animals and other organisms
☒	☐	Human research participants
☒	☐	Clinical data

Methods

n/a	Involved in the study	
☒	☐	ChIP-seq
☐	☒	Flow cytometry
☒	☐	MRI-based neuroimaging

Flow Cytometry

Plots

Confirm that:

☒ The axis labels state the marker and fluorochrome used (e.g. CD4-FITC).

☒ The axis scales are clearly visible. Include numbers along axes only for bottom left plot of group (a 'group' is an analysis of identical markers).

☒ All plots are contour plots with outliers or pseudocolor plots.

☒ A numerical value for number of cells or percentage (with statistics) is provided.

Methodology

Sample preparation	Cells were detached from the soil by vortexing it 5 min in PBS + 0.1% Tween. Samples were spined down at slow speed and the supernatant was collected.
Instrument	BD-Influx (BD-Bioscience) sterilized, sheath fluid was 1X PBS
Software	Data were collected with the BD Influx software and analyzed in FlowJo (https://www.flowjo.com)
Cell population abundance	Cell counts were performed using 1X SYBR staining, after 5 min incubation at room temperature. Cells were counted for each biological replicate. Cells were also counted water incubated samples as a control.
Gating strategy	The gating strategy is described in details in the method section and in supplementary figure S1. Briefly, the cells were primary gated based on a DNA dye (SYTO 58) and secondary gated based on the BONCAT fluorescent dye (FAM pycolyl azide). Both BONCAT positive and BONCAT - cells were collected. BONCAT positive gate was such that <0.5% of negative control (water incubated) cells were in it. Cytometry data are displayed in supplementary figures 4 and 5

☒ Tick this box to confirm that a figure exemplifying the gating strategy is provided in the Supplementary Information.
